# Kratom (*Mitragyna speciosa*) as a Phytochemical-Based Natural Product Exhibiting Opioid-like Analgesic Effects with Reduced Tolerance and Dependence Liability via TLR4-Associated Neuroimmune Modulation

**DOI:** 10.3390/molecules31091428

**Published:** 2026-04-26

**Authors:** Fajar Prasetya, Niken Indriyanti, Nurul Muhlisa Mus, Mentarry Bafadal, Raisa Fadilla, Yuli Widiyastuti, Chaidir Chaidir, Hadi Kuncoro, Sofa Fajriah, Rudi Heryanto, Angga Cipta Narsa, Onny Ziasti Fricillia, Yurika Sastyarina, Victoria Yulita Fitriani, Siti Rouchmana, Nurus Sobah, Zulhaerana Bahar, Nur Rezky Khairun Nisaa, Helmi Helmi, Hady Anshory

**Affiliations:** 1 Faculty of Pharmacy, University of Mulawarman, Samarinda 75119, Indonesia; 2 Medicinal Plants and Traditional Medicines Research and Development Centre, Tawangmangu 57792, Indonesia; 3 Tropical Biopharmaca Research Center, Bogor Agricultural Institute, IPB University, Bogor 16680, Indonesia; 4 Department of Pharmacy, Faculty of Mathematics and Natural Sciences, Universitas Islam Indonesia, Yogyakarta 55584, Indonesia

**Keywords:** *Mitragyna speciosa*, kratom, Toll-Like Receptor 4, pain management, analgesics, opioid, chromatography, high-pressure liquid, molecular docking simulation, flow cytometry, substance withdrawal syndrome

## Abstract

Kratom (*Mitragyna speciosa*) is a botanical candidate for pain management with potentially reduced opioid-related risks, partly through modulation of neuroimmune pathways involving Toll-Like Receptor 4 (TLR4). This study aimed to characterize the phytochemical profile of kratom ethanol extract and evaluate its effects on TLR4 signalling, neuroinflammatory cytokines, analgesic activity, withdrawal behaviours, and organ safety in morphine-dependent mice. Metabolite profiling was conducted using UHPLC–Q-Exactive Orbitrap HRMS, followed by molecular docking of major constituents to the TLR4 complex. In vivo assessments included flow cytometry and gene expression analyses of TLR4-mediated cytokines (NF-κB, IL-1β, IL-6), behavioural assays for antinociception, endurance, and withdrawal symptoms, and histopathological and biochemical evaluation of liver, kidney, and spleen tissues. More than 100 metabolites were identified, including mitragynine and flavonoids such as rutin and isoquercetin, which showed interactions with key TLR4 residues. Selected fractions suppressed pro-inflammatory cytokine expression, increased tail-pinch latency comparable to morphine, reduced withdrawal manifestations, and demonstrated nephroprotective and immunomodulatory effects, although mild reversible hepatic alterations were observed in specific fractions. Overall, kratom ethanol extract exhibited fraction-dependent analgesic and anti-neuroinflammatory activities associated with TLR4 modulation, supporting its potential as a botanical analgesic candidate while emphasizing the importance of safety optimization and standardized fraction development.

## 1. Introduction

The global opioid crisis has intensified the demand for safer, non-addictive analgesics. Traditional opioids, while effective in managing pain, are associated with severe risks, including dependence, tolerance, and fatal respiratory depression [1]. Amid the search for alternative analgesics, *Mitragyna speciosa*, commonly known as Kratom (Indonesia), has gained scientific interest for its unique pharmacological profile. Kratom (*Mitragyna speciosa*) or Kǎ tòng shù/卡痛树 (China) is a plant native to Southeast Asia and has long been consumed by local communities to support endurance and physical activity [2]. In recent decades, the plant has gained substantial attention across biomedical, economic, and sociocultural disciplines due to the broad array of bioactive constituents it contains.

Being a tropical evergreen member of the *Rubiaceae* family, Kratom is distributed across Indonesia, Malaysia, Thailand, and Papua New Guinea. Its leaves produce stimulant, analgesic, and opioid-like effects, contributing to the growing international interest in the plant. Modern investigations further underscore Kratom’s potential applications in pain control, mitigation of opioid withdrawal symptoms, and mood regulation [2,3,4]. In its native regions, Kratom extract has also been employed as a home remedy for conditions such as fever, diarrhea, cough, hypertension, and pain. In Western countries, however, its use is more commonly linked to self-management of chronic pain, opioid withdrawal, depression, and anxiety [5,6].

Kratom’s extract effects are dose-dependent. At low doses (1–5 g), kratom tends to produce stimulant-like effects such as increased energy, alertness, and sociability. At moderate to high doses (5–15 g), it induces sedation, euphoria, and pain relief, mimicking the effects of opioids [7]. To improve reproducibility and cross-study comparability, all doses in this study are expressed in mg/kg body weight (BW). However, it is important to note that the administered doses refer to crude extract or fraction weight rather than isolated active constituents. Given variability in alkaloid content due to extraction efficiency and plant origin, normalization to active compounds such as mitragynine (µg/kg BW) remains an important direction for future studies.

This dual action makes kratom unique among herbal psychoactive substances. User surveys and anecdotal reports suggest that kratom may help reduce the use of prescription opioids and mitigate withdrawal symptoms, although robust clinical trials are lacking. The pharmacological effects of kratom extract are largely attributed to its diverse array of alkaloids, of which more than 40 have been identified. The most abundant and pharmacologically active are mitragynine and 7-hydroxymitragynine. Mitragynine is the major alkaloid found in kratom leaves, accounting for approximately 66% of the total alkaloid content [8]. This compound has been reported to act as an agonist with affinity for opioid receptors, particularly those associated with dopaminergic and GABAergic interneurons.

Kratom extract exhibits agonist activity at µ-, δ-, and κ-opioid receptors [9]. Mitragynine shows a high affinity for the µ-opioid receptor, which mediates analgesia, respiratory depression, and euphoria. Its antinociceptive activity is primarily mediated through µ- and δ-opioid receptor subtypes. This mechanism underlies the use of kratom as a substitute for opium, as well as its ability to reduce opium addiction and alleviate withdrawal symptoms [10]. Mitragynine is a partial agonist at the μ-opioid receptor (MOR) and also exhibits activity at adrenergic and serotonergic receptors, contributing to its complex pharmacodynamics. 7-hydroxymitragynine, although present in lower concentrations, is significantly more potent and is believed to be the primary compound responsible for kratom’s analgesic effects [11,12]. However, unlike classical opioids such as morphine, Kratom alkaloids appear to have a lower propensity to induce respiratory depression and dependence-related behaviours in both preclinical and clinical studies [13,14].

Furthermore, administration of kratom ethanol extract at a dose of 4 mg/kgBW to mice was able to suppress withdrawal syndrome symptoms in the morphine addiction model [15]. One of the mechanisms potentially underlying this favourable profile is Kratom’s interaction with non-opioid targets, including the TLR4 pathway. The role of TLR4 in modulating opioid dependence and tolerance through natural products involves mitigating the receptor’s activation pathways linked to inflammation and glial cell activation in the central nervous system. Natural products targeting TLR4 could offer therapeutic benefits in addressing opioid-related issues by disrupting pro-inflammatory signalling. TLR4 activation is implicated in the processes of opioid dependence and the development of tolerance. It is known to activate glial cells, which then release pro-inflammatory cytokines contributing to the substance’s reinforcing properties [16]. Natural products, some derived from bacteria and plants, have shown efficacy in acting as TLR4 antagonists, thereby reducing the inflammatory processes involved in these pathways [17]. By attenuating TLR4-induced signalling, these products could potentially alleviate the development of opioid tolerance and dependence [16]. Furthermore, blocking TLR4 has been shown to decrease the conditioned place preference, which is a behavioural measure of drug reward, thus indicating a reduction in the rewarding effects of opioids [18]. This suggests that natural TLR4 antagonists could disrupt the reinforcing properties of opioids, thereby providing a novel pathway for managing opioid addiction and improving the clinical management of opioid-induced side effects. The continued investigation into TLR4 as a target has highlighted its potential for separating the analgesic effects of opioids from unwanted side effects like tolerance and dependence, which are significant concerns in clinical settings [16].

TLR4, a key component of the innate immune system, has been implicated in the development of opioid tolerance, hyperalgesia, and dependence [19]. Opioid drugs like morphine have been shown to activate TLR4, leading to neuroinflammation and adverse neuroplastic adaptations that contribute to their addictive properties. Inhibiting TLR4 has been proposed as a strategy to reduce these effects. Emerging evidence suggests that mitragynine may inhibit TLR4-mediated signalling, which could partly explain Kratom’s reduced liability for tolerance and dependence [20,21].

Furthermore, behavioural studies using rodent models have provided insight into Kratom extract’s potential to attenuate morphine-induced motor and coordination impairments. These models are crucial for understanding the neuromodulatory and behavioural effects of Kratom extract in the context of opioid addiction. For example, morphine-addicted mice typically exhibit significant deficits in coordination and increased signs of withdrawal, both of which have been mitigated in preclinical studies through administration of mitragynine [22].

Despite its promise, the regulatory status of Kratom remains controversial. The U.S. Food and Drug Administration has raised concerns over safety and quality control, citing reports of toxicity and contamination. However, growing preclinical evidence supports a re-evaluation of Kratom’s potential, especially its role in harm reduction and as an adjunct in opioid withdrawal management [6]. As such, detailed mechanistic studies particularly those exploring TLR4 inhibition and behavioural outcomes are urgently needed to validate Kratom’s therapeutic benefits while addressing safety concerns. This study aims to investigate the role of Kratom extract as a non-addictive opioid analgesic by focusing on its inhibition of TLR4 (in silico and in vivo) and its effects on coordination behaviour in morphine-addicted mice. Understanding these mechanisms may advance the development of novel analgesics with reduced risk of addiction and enhance therapeutic strategies for opioid use disorder.

## 2. Results and Discussion

### 2.1. Extraction and Fractionation Yields of Mitragyna speciosa Leaves

The extraction of plant material using 96% ethanol resulted in a yield of 12.6%, demonstrating efficient recovery of ethanol-soluble constituents from the plant matrix. Ethanol, being a polar protic solvent, is widely reported to efficiently extract a broad range of secondary metabolites, including alkaloids, phenolics, and semi-polar terpenoids. Subsequent fractionation using solvents of varying polarity produced distinct yield patterns that reflect the distribution of metabolites according to their polarity. Among the chloroform–methanol fractions, the highest yields were observed in Fraction IV (70:30, 25.8%) and Fraction V (50:50, 22.46%), followed by Fraction III (95:5, 10.3%). This pattern indicates that a substantial portion of the extractable metabolites are of intermediate polarity, consistent with the enrichment of alkaloids, phenolics, and semi-polar terpenoids typically found in Mitragyna species [23]. In contrast, the n-hexane–ethyl acetate fractions yielded lower amounts, with Fraction I (70:30) at 5.4% and Fraction II (30:70) at 10.64%, indicating the presence of non-polar to mildly polar constituents such as lipophilic alkaloids, sterols, and fatty acid derivatives [24]. The methanolic fractions, Fraction VI (100% MeOH) and Fraction VII (60% MeOH), yielded 8.4% and 3.9%, respectively, suggesting that highly polar metabolites including sugars, glycosides, and very polar phenolics constitute a relatively minor portion of the overall extractable matrix [25]. Overall, the fractionation profile demonstrates that the plant material predominantly contains semi-polar metabolites, with the highest enrichment occurring in chloroform–methanol fractions containing higher methanol proportions. This distribution provides a rational basis for downstream bioassays, as fractions with higher yields often correlate with greater phytochemical diversity and potential pharmacological activity [23].

### 2.2. Metabolites Identification of Kratom (Mitragyna speciosa) Leaf Ethanol Extract Through UHPLC-Q-Exactive Plus Orbitrap HRMS

Metabolite identification was performed using UHPLC-Q-Exactive Orbitrap HRMS and should be considered as putative (Level 2 identification according to the Metabolomics Standards Initiative), based on accurate mass and database matching without full confirmation by reference standards. The chemical composition of Kratom is complex and varies depending on geographical origin, harvesting methods, and extraction techniques. Over 40 alkaloids have been isolated from Kratom leaves, along with flavonoids, terpenoids, saponins, and other phytochemicals [26,27]. Among these, mitragynine accounts for up to 66% of total alkaloid content in Thai Kratom, while 7-hydroxymitragynine, although present in lower concentrations, is significantly more potent at opioid receptors [28]. Given the growing medicinal interest and regulatory scrutiny, the need for comprehensive metabolite profiling of *M. speciosa* is paramount. Advanced analytical platforms such as ultra-high-performance liquid chromatography coupled with high-resolution mass spectrometry (UHPLC-HRMS) enable the untargeted and sensitive identification of plant secondary metabolites, including those present in trace amounts [29]. The Q-Exactive Plus Orbitrap HRMS, in particular, offers excellent mass accuracy, resolution, and fragmentation capabilities that are essential for dereplication, structural elucidation, and compound annotation in complex botanical matrices. Previous studies have often focused on targeted quantification of major alkaloids in Kratom using LC-MS/MS. However, comprehensive metabolomics using full-scan HRMS combined with data-dependent acquisition (DDA) allows for a more holistic understanding of the phytochemical landscape, including minor or previously unidentified metabolites that may contribute to pharmacological or toxicological outcomes [30]. In this study, we employed UHPLC-Q-Exactive Plus Orbitrap HRMS to profile and annotate the ethanol extract metabolites of *M. speciosa* leaves (Table 1). Ethanol was selected as the extraction solvent due to its ability to extract a broad spectrum of both polar and non-polar secondary metabolites.

Compounds sharing identical molecular formulas but different retention times (e.g., entries **1**, **2**, **7**, and **12**) are interpreted as putative structural isomers or analogues rather than identical compounds. Additionally, stereochemical configurations (e.g., compounds **21**, **27**, **52**, and **65**) cannot be resolved using HRMS alone and require further structural elucidation such as NMR or chiral chromatography. It is important to emphasize that alkaloids, particularly mitragynine, represent the dominant bioactive constituents of Mitragyna speciosa, as confirmed by peak area distribution in Table 1. Flavonoids such as rutin, isoquercetin, and cianidanol were detected in comparatively lower abundance. Their discussion in the molecular docking section does not imply primary pharmacological dominance, but rather reflects their specific binding orientation within key MD2 residues associated with TLR4 modulation. Thus, alkaloids remain the principal drivers of opioid receptor–mediated activity, while flavonoids may contribute secondary neuroimmune modulatory effects. No internal standards were applied in the untargeted LC-HRMS analysis, as the primary objective was qualitative metabolite profiling. Any reference to internal standards pertains to separate targeted analytical workflows. Detailed chromatographic conditions and retention time data are provided in the Supplementary Materials.

### 2.3. Molecular Investigating the Binding Affinity of Kratom (Mitragyna speciosa) Leaf Ethanol Extract Constituents to TLR4 Protein (PDB ID: 4G8A)

Morphine is known to activate TLR4 signalling, whereas kratom-derived compounds are hypothesized to modulate or attenuate this activation rather than directly activate the receptor. The major alkaloids found in Kratom, particularly mitragynine and 7-hydroxymitragynine, exhibit partial agonist activity at μ-opioid receptors, while recent studies suggest they may also influence neuroinflammatory pathways through non-opioid targets such as TLR4 [21]. Given the role of TLR4 in mediating opioid-induced hyperalgesia and tolerance, it is hypothesized that Kratom alkaloids may contribute to analgesia without addiction by interacting with and inhibiting TLR4 signalling. In silico molecular docking provides a powerful and cost-effective approach to assess the binding affinity and interaction patterns of small molecules with target proteins. The Maestro Schrödinger Glide module offers high-precision docking using advanced scoring functions to predict ligand–receptor binding with atomic-level accuracy.

This study employed Maestro (Schrödinger Release 2025-1, Schrödinger, LLC, New York, NY, USA), including the Glide module, to investigate the molecular interactions between key alkaloids and flavonoids from 96% ethanol extract of kratom leaves and the TLR4/MD2 receptor complex (PDB ID: 4G8A) to elucidate their role in the modulation of TLR4 signalling pathways. The docking workflow consisted of protein preparation, ligand optimization using LigPrep (Schrödinger Release 2025-1), receptor grid generation, and docking in both Standard Precision (SP) and Extra Precision (XP) modes, with docking scores and binding poses evaluated to predict inhibitory potential. This in silico approach was designed to identify phytoconstituents capable of binding to TLR4 and to clarify the mechanistic basis of Kratom’s reported analgesic effects with lower addiction liability compared to classical opioids. The computational findings provide an initial framework for subsequent pharmacological validation and the development of novel non-opioid analgesics that may circumvent the adverse effects of traditional opioid therapy.

Based on the docking results of compounds found in Kratom extracts obtained using 96% ethanol solvents (Table 2), most ligands demonstrated the ability to interact with the active site of the TLR4/MD2 complex, and this aligns with earlier computational reports showing that non-lipopolysaccharide (non-LPS) small molecules can engage the MD-2 cavity while also influencing receptor conformation and signalling behaviour [31]. MD2 functions here as an adaptor that facilitates ligand recognition and initiates TLR4 signalling. The results further indicate that the tested compounds in this study exhibit a meaningful potential to modulate TLR4 signalling, reinforcing the rationale for developing safer non-opioid analgesics with reduced risk of tolerance and addiction. Among the compounds evaluated, Rutin and Isoquercetin emerged as strong candidates for continued investigation. These two flavonoids are widely known for their anti-inflammatory effects and, in this study, also demonstrated direct interactions with essential MD2 residues such as GLU92, SER120, and TYR102 residues previously implicated in stabilizing ligand engagement or antagonistic activity in structural studies of the TLR4/MD2 complex [32].

Rutin showed a docking score of −5.36, indicating a favourable binding affinity, alongside interactions with residues critical for initiating TLR4 signalling modulation. Isoquercetin, with a docking score of −5.237, also exhibited stable interactions involving TYR102, GLU92, VAL93, and SER120, consistent with prior reports describing the capacity of flavonoids such as Rutin to modulate TLR4-mediated inflammatory signalling [33]. When integrating these findings docking scores, interaction sites, and existing pharmacological evidence the data reinforce the strategic value of prioritizing Rutin and Isoquercetin for further development as TLR4 modulators, particularly given their alignment with known structural determinants of ligand accommodation within MD2. Their potential now warrants validation through in vitro assays, crystallographic studies, and broader biological activity evaluations. Overall, the docking scores offer quantitative support for the TLR4 signalling modulation hypothesis by estimating the affinity of candidate ligands for key functional regions of the TLR4/MD2 complex, strengthening the mechanistic premise that strong binding within regulatory pockets may prevent engagement by native ligands such as LPS and thereby attenuate downstream pro-inflammatory signalling, ultimately supporting the pursuit of safer analgesic alternatives with lower risks of tolerance and addiction.

The primary compound in Kratom, Mitragynine, recorded the lowest docking score at −6.341, indicating high binding affinity; however, its interactions were limited to PHE76 and PHE151, which are not part of the core active site of TLR4. Therefore, despite its strong affinity, its binding position may be less relevant to the modulation of the TLR4 signalling pathway, which is the primary focus of this study. To improve structural interpretation, the docking pose of mitragynine was further visualized to show its orientation within the MD-2 binding cavity and its proximity to key residues associated with ligand accommodation. This presentation helps distinguish high binding affinity from biologically meaningful pocket occupancy.

Molecular docking provides an estimation of binding affinity and interaction patterns but does not determine whether a compound acts as an agonist or antagonist. Functional effects require experimental validation. In addition to direct binding at the MD-2 active site, it is important to consider that small molecules may exert indirect or allosteric effects on TLR4 signalling, including modulation of receptor dimerization or conformational dynamics. Therefore, the observed docking interactions may not solely reflect competitive inhibition but could also involve alternative regulatory mechanisms. Reference ligands such as lipopolysaccharide (LPS, TLR4 agonist) and naloxone (reported TLR4 modulator) were not included in the docking simulation; therefore, docking scores should be interpreted qualitatively rather than comparatively.

### 2.4. Flow Cytometric Profiling of In Vivo TLR4-Inhibited Glial Cytokine Responses: Implications for Opioid Addiction, Dependence, and Tolerance

In this study, ‘bioactivity’ refers to general biological effects observed at the molecular or cellular level, whereas ‘pharmacological activity’ denotes mechanism-specific effects demonstrated in vivo. All in vivo experiments were conducted in morphine-dependent mice. The analyzed cell population primarily represents microglial cells (CD68^+^), although contributions from other glial subtypes cannot be excluded. The development of opioid analgesics that provide effective pain relief without triggering addiction, dependence, or tolerance remains a major challenge in neuropharmacology. Recent studies have highlighted the critical role of neuroimmune interactions, particularly the activation of TLR4 on glial cells, in mediating the adverse neurological adaptations associated with chronic opioid use. TLR4 activation leads to the release of pro-inflammatory cytokines such as TNF-α, IL-1β, and IL-6, contributing to neuroinflammation, opioid tolerance, and reward pathway sensitization [31] [34,35,36,37]. Unlike classical opioids such as morphine, certain alkaloids in Kratom particularly mitragynine and 7-hydroxymitragynine are believed to exert analgesic effects with minimal recruitment of β-arrestin-2 [12] and limited activation of glial-mediated TLR4 signalling [38]. This may suggest that Kratom may suppress pro-inflammatory cytokine release from glial cells, mitigating the immune-related mechanisms that underlie opioid-induced addiction and tolerance. To investigate this hypothesis, flow cytometry was employed to profile cytokine responses in glial cells following exposure to Kratom fraction in the presence or absence of TLR4 inhibition. This approach allows for high-resolution quantification of intracellular cytokine expression, enabling the elucidation of immune-modulatory effects at the cellular level. The resulting data provide insight into how TLR4 blockade either pharmacologically or through Kratom bioactivity may attenuate glial inflammatory signalling and offer a novel therapeutic pathway for opioid analgesia without the typical liabilities of dependence and tolerance.

**Figure 1:**
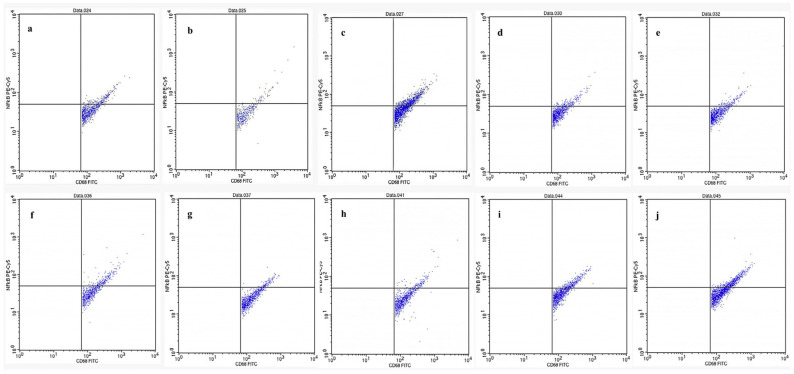
Distribution of CD68^+^NF-κB expression in mouse brain microglial cells from normal, control, and treatment groups following administration of kratom extract fractions. (**a**) Normal control (NaCMC 0.1%, p.o.); (**b**) Positive control (naloxone, 0.00104 mg/20 g BW, s.c.); (**c**) Negative control (morphine, 10–30 mg/kg BW, p.o., with gradually increasing doses); (**d**–**j**) treatment groups receiving different kratom fractions. Cells were stained with PE-conjugated antibodies and analyzed using a BD FACS flow cytometer. Data are presented as mean ± SD (*n* = 5). Statistical significance is indicated as *p* < 0.05, *p* < 0.01, and *p* < 0.001 compared with the designated control group.

Figure 1 presents flow cytometric analysis of glial cells isolated from the brains of mice in various experimental groups, including normal control, negative control (morphine-induced), positive control (naloxone-treated), and treatment groups, and treatment groups receiving different fractions of kratom (*Mitragyna speciosa*) ethanol extract. Cells were stained with phycoerythrin (PE)-conjugated antibodies targeting CD68 and NF-κB to assess microglial activation and pro-inflammatory signalling. CD68 is a marker of activated microglia, while NF-κB is a transcription factor involved in initiating inflammatory responses. The dual expression of CD68 and NF-κB indicates an activated inflammatory state in glial cells. Flow cytometric analysis demonstrated that several kratom fractions reduced CD68^+^NF-κB^+^ cell populations compared to the negative control group (morphine-treated), indicating attenuation of morphine-induced glial activation.

The present study demonstrates that kratom fractions are capable of downregulating the expression of key pro-inflammatory cytokine genes in glial cells, indicating a suppressive effect on neuroinflammatory signalling pathways in morphine-dependent animal models. This downregulation is reflected by reduced expression of NF-κB, IL-1, and IL-6, which are typically upregulated during glial activation in response to opioid exposure. As a major transcription factor, NF-κB plays a pivotal role in regulating the expression of various inflammatory mediators, including IL-1 and IL-6.

**Figure 2:**
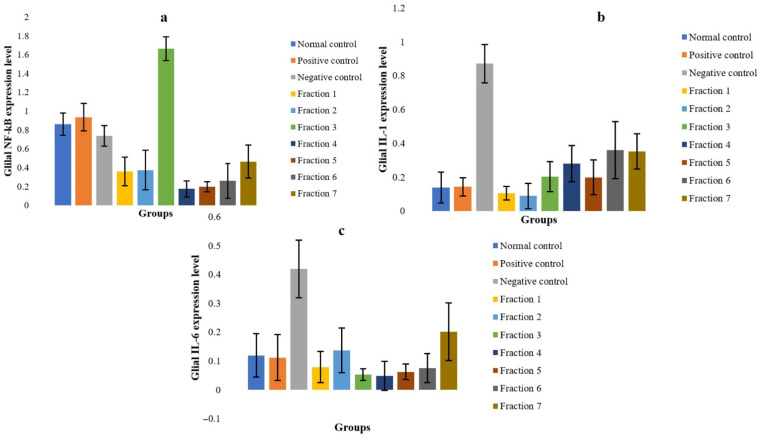
Effect of kratom on the cytokines pro-inflammatory gene expression in the glial cells. (**a**) NF-kB expression level, (**b**) interleukin-1 (IL-1) expression level and (**c**) Interleukin-6 (IL-6) expression level. Data are presented as mean ± SD (*n* = 5). Normal group (NaCMC 0.1%) (p.o), Positive group (Naloxone) subcutaneously (s.c) (0.00104 mg/20 g body weight) at the end of the test, Negative group (Morphine) (p.o) (10–30 mg/kg body weight with gradually increasing doses).

According to Figure 2a, administration of kratom fractions suppressed NF-κB expression, with the most pronounced inhibition observed in fractions 4 and 5, suggesting that kratom interferes with upstream signalling pathways involved in glial activation. Interestingly, Fraction 3 exhibited relatively elevated NF-κB expression compared to other fractions. This may reflect a complex immunomodulatory response rather than a purely inhibitory effect, potentially involving transient activation or feedback regulation mechanisms. Further studies are required to clarify this observation. This anti-inflammatory modulation likely contributes to the observed behavioural improvements and may provide neuroprotective effects during opioid withdrawal. The absence of statistically significant differences in behavioural parameters may be attributed to the limited sample size and low incidence of observable withdrawal symptoms in certain groups. This limitation highlights the importance of integrating behavioural observations with molecular and immunological endpoints. In this study, flow cytometry data targeting TLR4-associated neuroimmune signalling provided more sensitive and mechanistically relevant evidence of kratom’s effects. IL-1, a potent pro-inflammatory cytokine primarily produced by activated microglia, is known to increase during opioid withdrawal and is associated with neurotoxicity, emotional dysregulation, and heightened pain sensitivity [34,35,39]. In the withdrawal model, IL-1 expression was elevated, reflecting glial activation. However, treatment with kratom fractions markedly reduced IL-1 expression, with optimal inhibition observed in fraction 2 (Figure 2b), indicating that kratom can modulate glial-mediated cytokine release, potentially via mitragynine or other alkaloids that interfere with TLR4 signalling.

Similarly, IL-6, another pro-inflammatory cytokine elevated during opioid withdrawal and linked to malaise, fatigue, and depressive-like behaviours [40], was suppressed by kratom fractions, particularly in fractions 3 and 4 (Figure 2c), further supporting kratom’s role in mitigating glial inflammation, stabilizing neuroimmune homeostasis, and reducing withdrawal-associated behavioural abnormalities.

**Figure 3:**
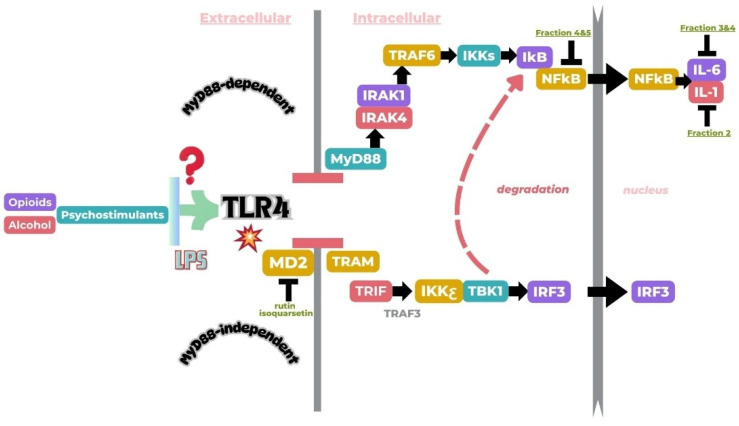
Pharmacological Modulation of TLR4 Signalling by Bioactive Fractions: Inhibition of NF-κB and Cytokine Pathways in Drug Addiction.

Collectively, these results indicate that kratom fractions can interfere with the MyD88-dependent signalling cascade downstream of TLR4, thereby reducing the transcription of inflammatory mediators via NF-κB inhibition (Figure 3). It should be noted that the measurement of total NF-κB expression does not fully reflect its transcriptional activity, as nuclear localization is a more direct indicator of NF-κB activation. Therefore, the present results should be interpreted as indicative of relative expression rather than definitive activation status. This anti-inflammatory effect is particularly relevant in opioid dependence, where chronic morphine exposure triggers glial sensitization and prolonged cytokine release, contributing to tolerance and addictive behaviours. By modulating glial immune responses, kratom shows potential as a novel analgesic with lower risk of neuroimmune-driven addiction, supporting its development as a TLR4-targeted therapy. The observed decrease in NF-κB, IL-1β, and IL-6 expression indicates that kratom primarily acts on central neuroimmune pathways, inhibiting microglial activation and pro-inflammatory transcription. This action may complement kratom’s partial μ-opioid receptor activity, alleviating both emotional and physical symptoms associated with opioid withdrawal. Meanwhile, based on the data (Figure 2), morphine substantially increases the production of the cytokines IL-1 and IL-6 [41].

### 2.5. Kratom (Mitragyna speciosa) Demonstrated an Improvement in the Physical Symptoms Associated with Opioid Withdrawal Syndrome in Morphine-Addicted Mice

Preclinical investigations into kratom (*Mitragyna speciosa*) have demonstrated its potential in alleviating the physical symptoms of opioid withdrawal syndrome in morphine-dependent murine models. In behavioural assays conducted during naloxone-precipitated withdrawal, Kratom-treated mice exhibited a reduction in characteristic withdrawal behaviours such as jumping, paw tremors, teeth chattering, and wet dog shakes, when compared to vehicle-treated controls. These improvements suggest that Kratom may mitigate opioid-induced neuronal hyperexcitability. Mechanistically, the observed effects are likely attributable to the partial μ-opioid receptor agonism of Kratom’s primary alkaloids, such as mitragynine, as well as its ability to modulate neuroimmune pathways. Notably, Kratom has been shown to downregulate pro-inflammatory cytokine expression in glial cells through Toll-like Receptor 4 (TLR4) inhibition, which may further contribute to the attenuation of withdrawal-related neuroinflammation [42,43]. Together, these findings highlight Kratom’s dual neuromodulatory and anti-inflammatory mechanisms as promising therapeutic strategies in the management of opioid withdrawal, warranting further translational studies.

**Figure 4:**
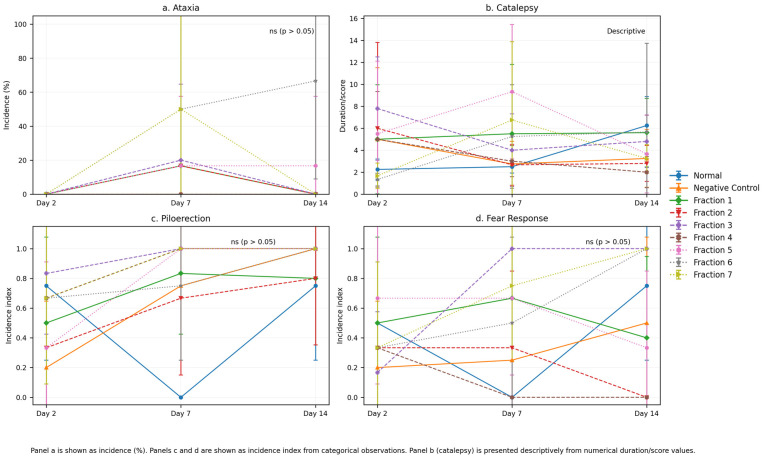
Effect of kratom on physical withdrawal symptoms, including (**a**) ataxia, (**b**) catalepsy, (**c**) piloerection, and (**d**) fear response, evaluated on days 2, 7, and 14. Data are presented as mean ± SD based on available observations in each group. Panel (**a**) is presented as incidence (%). Behavioural data (ataxia, piloerection, and fear response) were analyzed using Fisher’s exact test due to their categorical nature. No statistically significant differences were observed between groups at all time points (ns, *p* > 0.05). Catalepsy data, recorded as numerical duration/score, are presented descriptively. Raw data are provided in the Supplementary Materials.

In the morphine-addicted mice, withdrawal triggers a range of physical symptoms due to the sudden cessation of opioid signalling in the central nervous system. As shown in Figure 4, The effects of kratom fractions on withdrawal-related behavioural parameters showed variability across treatment groups. While some fractions demonstrated improvements in specific parameters, these effects were not consistently observed across all endpoints. More consistent trends were observed in selected fractions, statistical analysis of behavioural parameters (ataxia, catalepsy, piloerection, and fear response) was performed to evaluate differences between treatment groups. Due to the categorical nature and low incidence of most behavioural endpoints, Fisher’s exact test was applied for ataxia, piloerection, and fear response. The results showed no statistically significant differences between groups at all observed time points (day 2: *p* = 1.000; day 7: *p* = 0.561; day 14: *p* = 1.000). Although some variations in behavioural responses were observed across treatment groups, these differences did not reach statistical significance and are therefore interpreted as descriptive trends rather than statistically confirmed effects. Catalepsy, recorded as numerical duration/score, is presented descriptively due to variability and limited sample size. These findings suggest that while kratom fractions may exhibit mild modulatory effects on withdrawal-related behaviours, the behavioural data primarily serve as supportive evidence, complementing the more robust immunopharmacological findings derived from flow cytometry analysis.

Withdrawal symptoms such as ataxia, catalepsy, piloerection, and enhanced index of fear responsiveness reflect disturbances in central nervous system (CNS) activity and autonomic imbalance [44]. Treatment with kratom fractions improved these parameters, indicating a neurophysiological stabilizing effect likely mediated by kratom’s active compounds. According to Figure 4a, ataxia observed in the morphine-withdrawn control group was characterized by motor discoordination and unsteady movement, aligning with previous findings on opioid withdrawal-induced motor impairment. On the day 14, mice receiving kratom fractions showed a reduction in the ataxia duration, especially in the fraction 1, 3 and 7 groups. This finding suggesting a protective effect on motor control and may be attributed to mitragynine’s partial agonist activity at μ-opioid receptors, which helps to attenuate withdrawal severity without inducing full opioid effects [45].

Catalepsy, defined by prolonged immobility and resistance to external movement, was evident in the withdrawal group. In contrast, kratom-treated animals exhibited a marked reduction in cataleptic behaviour (Figure 4b), especially in the fraction 5 and 7 groups indicating that kratom does not exacerbate CNS rigidity and may in fact modulate dopaminergic or GABAergic systems, which are implicated in cataleptic responses [46,47].

Piloerection is a classic autonomic symptom of opioid withdrawal, indicative of sympathetic overactivity. In this study, mice treated with kratom fractions showed a decrease in the frequency of piloerection episodes (Figure 4c). This suggests that kratom may exert a modulatory effect on noradrenergic pathways, which are typically hyperactive during withdrawal. Enhanced fear or anxiety-like behaviours such as freezing, increased startle reflexes, and avoidance behaviour were attenuated in the kratom-treated groups (Figure 4d). These findings support the anxiolytic potential of kratom, possibly due to its influence on serotonergic and adrenergic receptors, both of which play roles in stress and emotional regulation [13]. Collectively, the improvement across all four behavioural and physical parameters suggests that kratom fractions exert multi-target effects on the neurobiological systems involved in opioid withdrawal. The combination of mild opioid receptor activation and modulation of monoaminergic systems may underlie the observed therapeutic benefits.

### 2.6. Kratom (Mitragyna speciosa) Exhibits Analgesic Effects Based on the Tail Pinch Test in Morphine-Addicted Mice

The tail-pinch test is a classical behavioural assay widely used in rodents to assess nociceptive sensitivity and analgesic efficacy of pharmacological agents. In this test, a mild mechanical stimulus (pinch) is applied to the distal tail, and the latency to exhibit a behavioural response such as vocalization, tail withdrawal, biting, or body movement is measured. The longer the latency, the greater the analgesic effect, whereas shorter latencies indicate either no analgesic effect or heightened nociceptive sensitivity. This assay is particularly valuable for evaluating both centrally acting analgesics, such as opioids, and plant-derived compounds with potential antinociceptive properties.

**Figure 5:**
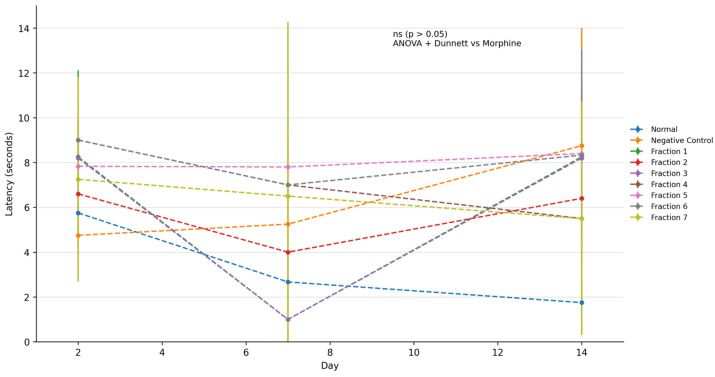
Analgesic activity of kratom fractions evaluated using the tail pinch test in morphine-dependent mice on days 2, 7, and 14. Data are presented as mean ± SD (*n* = 5). Statistical analysis was performed using one-way ANOVA followed by Dunnett’s post hoc test against the negative control group (morphine-treated). No statistically significant differences were observed between groups at all time points (ns, *p* > 0.05). Raw data are provided in the Supplementary Materials.

The tail pinch test was conducted to evaluate nociceptive response in morphine-dependent mice following treatment with kratom extract fractions. Statistical analysis using one-way ANOVA followed by Dunnett’s post hoc test revealed no statistically significant differences between treatment groups and the negative control group at all observed time points (Day 2, Day 7, and Day 14; *p* > 0.05). Although some groups exhibited increased latency values compared to the negative control, these differences did not reach statistical significance and are therefore interpreted as descriptive trends. The relatively large variability within groups, as indicated by overlapping standard deviations, may have contributed to the absence of statistically significant findings. These results suggest that the tail pinch test data should be interpreted cautiously and primarily as supportive observations. Due to overlapping variability (SD) across groups, the observed differences should be interpreted as descriptive trends rather than statistically confirmed differences. The tail-pinch response integrates spinal and supraspinal nociceptive processing, involving ascending sensory pathways and descending modulatory systems, including opioidergic, monoaminergic, and GABAergic circuits. Therefore, changes in tail-pinch latency can reflect the efficacy of an analgesic at multiple levels of the central nervous system. According to Figure 5, mice treated with kratom fraction showed a trend toward increased latency in tail-pinch latency compared to the normal control group, which did not receive any test substance. The latency observed in the kratom-treated group approached that of the morphine-treated group, indicating a comparable level of antinociceptive effect [25,48]. This finding suggests that kratom possesses analgesic properties capable of modulating nociceptive responses, potentially through its action on central opioid receptors [46,47]. The prolonged latency may be attributed to mitragynine, the primary alkaloid in kratom, which has been reported to act as a partial μ-opioid receptor agonist, thereby increasing pain threshold without eliciting the full spectrum of effects associated with morphine [3,45].

Although kratom fractions demonstrated significant antinociceptive effects under the experimental conditions employed, the present study did not perform equianalgesic dose comparisons or determine ED50 values relative to morphine. Therefore, the findings should be interpreted as demonstrating analgesic potential within a morphine-dependence framework rather than establishing pharmacodynamic equivalence with classical opioids. The absence of statistically significant differences in the tail pinch test may be attributed to variability within treatment groups and the limited sample size. These findings highlight the importance of interpreting behavioural data in conjunction with molecular and immunological endpoints. In this study, flow cytometry analysis targeting TLR4-associated neuroimmune signalling provides more robust and mechanistically relevant evidence of kratom’s effects, while behavioural outcomes serve as supportive observations. Kratom (*Mitragyna speciosa*) leaves contain the indole alkaloids mitragynine and 7-hydroxymitragynine, which have been shown in vitro and in vivo to bind to μ- (MOR), δ- (DOR), and κ-opioid receptors (KOR) [20]. Detailed receptor-binding and functional studies demonstrate that these alkaloids act as partial agonists at the μ-opioid receptor with measurable affinity for δ and κ receptors, and that their antinociceptive effects in animal models are reversed by opioid antagonists indicating that activation of opioid receptors, particularly MOR, is the primary molecular mechanism underlying kratom’s opioid-like analgesic effects [43]. The observed variation in response time in the control group may be attributed to behavioural adaptation, stress, or learning effects rather than pharmacological influence. These results support studies demonstrating the analgesic of kratom and highlight its capacity to modulate both spinal and supraspinal nociceptive pathways [49].

### 2.7. Kratom (Mitragyna speciosa) Led to an Increase in Endurance, as Evidenced by Improved Performance in Both the Forced Swimming Test and the Traction Test in Morphine-Addicted Mice

Preclinical behavioural assays revealed that administration of Kratom (*Mitragyna speciosa*) enhanced physical endurance and neuromuscular coordination in murine models. In the forced swimming test, Kratom-treated mice exhibited a delayed onset of immobility and prolonged swimming duration, indicating improved stress resilience and energy capacity under fatigue-inducing conditions [2]. Additionally, in the traction test, animals demonstrated enhanced grip strength and motor coordination, suggesting a stimulatory effect on the neuromuscular system [50]. These enhancements in performance may be attributed to Kratom’s complex pharmacological profile, including partial agonism at μ-opioid receptors and modulation of dopaminergic and adrenergic pathways [3]. Furthermore, Kratom’s anti-inflammatory properties, mediated by TLR4 inhibition and downregulation of pro-inflammatory cytokines, may contribute to improved mitochondrial function and reduced central fatigue [51]. Collectively, these findings position Kratom as a potential phytotherapeutic agent with adaptogenic properties relevant for fatigue-related conditions or opioid withdrawal-induced weakness.

The present study provides compelling evidence that kratom fractions exhibit therapeutic effects in morphine-addicted mice, particularly in improving behavioural and motor performance as assessed by the Forced Swimming Test (FST) and the Traction Test. Both tests serve as important indicators of physical and emotional withdrawal severity, reflecting motor endurance, neuromuscular strength, and depressive-like behaviours that are commonly exacerbated during opioid withdrawal.

**Figure 6:**
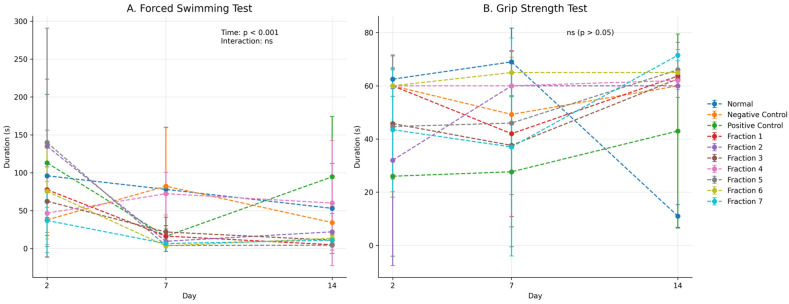
Effect of kratom on the endurance in the forced swimming test and traction test with parameter (**A**) duration of swimming and (**B**) duration of grip. Data are presented as mean ± SD (*n* = 5). Normal group (NaCMC 0.1%) (p.o), Positive group (Naloxone) subcutaneously (s.c) (0.00104 mg/20 g body weight) at the end of the test, Negative group (Morphine) (p.o) (10–30 mg/kg body weight with gradually increasing doses).

In morphine-addicted mice undergoing withdrawal, the duration of active swimming is typically reduced, reflecting behavioural despair or lethargy, a hallmark of depressive-like states during opioid withdrawal. In this study, animals in the withdrawal (negative control) group exhibited variable swimming durations across time points, without a consistent decreasing trend; the mean value at Day 14 was slightly lower than Day 2, but with substantial variability (Figure 6A), confirming the onset of opioid-induced behavioural depression. Interestingly, treatment with kratom fractions increased the swimming duration in the all treated-groups, except in the fraction 2 and 4 groups (Figure 6A). This indicating a reversal of withdrawal-induced depressive-like behaviour and suggests that kratom may possess mild antidepressant or psychostimulant properties, possibly through interaction with the monoaminergic system, particularly serotonin and dopamine pathways. Mitragynine, the major alkaloid in kratom, has been shown in other studies to modulate mood and arousal, supporting this hypothesis [46,47]. Although selected fractions (notably fractions 2 and 4) showed improved performance in traction and endurance tests, overlapping variability indicates that these findings should be interpreted as trends rather than statistically confirmed effects.

The traction test evaluates muscle strength and neuromuscular coordination, both of which are often impaired during opioid withdrawal due to autonomic dysregulation and reduced motor tone. In the traction test, morphine-withdrawal (negative control) mice exhibited reduced grip duration, indicating impaired motor performance. Kratom-treated groups showed improved grip duration, particularly fractions 2 and 4 (Figure 6B), suggesting a potential attenuation of withdrawal-associated motor deficits and a recovery trend in motor function. This improvement may be attributed to kratom’s partial agonism at μ-opioid receptors, which helps attenuate withdrawal severity without causing sedation [45]. Additionally, the fraction’s influence on central dopaminergic and noradrenergic activity may contribute to enhanced motor performance.

Together, the improvement in swimming activity and grip strength reflects a broader alleviation of withdrawal-induced behavioural and physical impairments. The observed effects indicate that kratom extract fractions can counteract both central (mood-related) and peripheral (muscular) symptoms of opioid withdrawal. These findings align with earlier studies suggesting kratom’s utility as a transitional agent in opioid detoxification, offering a potential reduction in withdrawal severity while avoiding the intense psychoactive effects of full opioid agonists [6,45].

### 2.8. Histopathological and Immunopharmacological Evaluation of Mitragyna speciosa Fractions: Organ-Protective, Immunomodulatory, and TLR4-Inhibitory Effects Against Morphine-Induced Toxicity

Histopathological examination of tissue sections from Kratom-treated subjects revealed an increase in the lymphoid index, characterized by elevated infiltration of lymphocytes in specific regions, such as the spleen, lymph nodes, or brain parenchyma, depending on the tissue analyzed. This lymphocytic accumulation may indicate an immunomodulatory response triggered by Kratom’s bioactive constituents, possibly involving both adaptive and innate immune pathways. Such changes could reflect a physiological reaction to phytochemical stimulation or a compensatory response to underlying inflammatory regulation. In the context of opioid exposure or neuroinflammation, increased lymphoid presence may also represent an effort to restore immune homeostasis, particularly if Kratom’s TLR4-inhibiting effects shift the local cytokine environment toward a less pro-inflammatory state. While the precise implications of elevated lymphoid indices require further investigation, these findings support the hypothesis that Kratom influences immune architecture and should be evaluated for its broader immunopharmacological properties.

Morphine-induced organ toxicity is a well-documented pathological consequence of prolonged opioid exposure, primarily mediated by oxidative stress, inflammation, and mitochondrial dysfunction. In this study, histopathological evaluation was conducted on kidney, spleen, and liver tissues from mice treated with morphine and various ethanol fractions of *Mitragyna speciosa* (kratom) leaf extract. Data were compared to normal, negative (morphine), and positive (morphine + naloxone) controls to determine protective or adverse effects.

#### 2.8.1. Splenic Histopathology and Immunomodulatory Response

**Figure 7:**
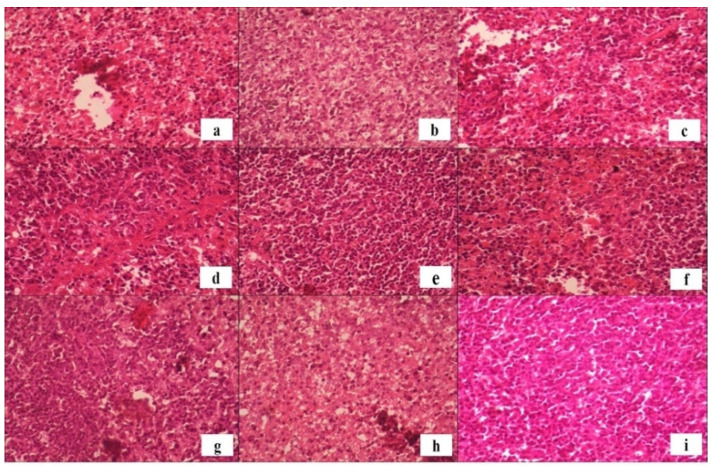
Effect of kratom in the Splenic histopathology analysis. (**a**) normal control, (**b**) negative control, (**c**) fraction 1, (**d**) fraction 2, (**e**) fraction 3, (**f**) fraction 4, (**g**) fraction 5, (**h**) fraction 6 and (**i**) fraction 7 with microscopic magnification of 40×. Normal group (NaCMC 0.1%) (p.o), Positive group (Naloxone) subcutaneously (s.c) (0.00104 mg/20 g body weight) at the end of the test, and Negative group (Morphine) (p.o) (10–30 mg/kg body weight with gradually increasing doses).

Morphine exposure is known to suppress immune function and promote systemic inflammation through glucocorticoid and cytokine imbalance [52]. In the negative control group, splenic edema (1.40%) and splenic necrosis (2.6%) were observed (Table 3), confirming morphine-induced immune dysregulation [53,54,55,56]. Conversely, Fraction 7 markedly reduced edema (0.65%) and splenic necrosis (0.87%), indicating potent anti-inflammatory effects. Histopathological analysis revealed an increase in lymphoid cell infiltration following kratom administration, signifying enhanced immune activity or localized immunomodulation. Elevated lymphoid indices—particularly in the spleen and lymph nodes—may reflect the activation of both adaptive and innate pathways through bioactive alkaloid-mediated modulation of TLR4 signalling. Such responses likely represent compensatory immune restoration following opioid-induced suppression. Figure 7b,c,h,i show preserved red and white pulp structures in kratom-treated groups, with intact sinusoids and periarteriolar lymphoid sheaths. These indicate maintenance of normal hemodynamic and immune function. Fractions 4 and 5 displayed mild architectural alterations, including disproportionate white pulp expansion and partial lymphoid disruption, which may suggest localized lymphoproliferative responses at certain dose thresholds.

Histopathological examination of lymph tissue in the experimental groups revealed preserved architecture of both red pulp and white pulp, which are key indicators of lymph immune function. The red pulp, characterized by abundant blood-filled sinusoids and splenic cords, remained intact, indicating normal hemodynamic and filtering activity. Similarly, the white pulp, which contains densely packed lymphocytes organized around central arterioles (periarteriolar lymphoid sheaths), showed no signs of atrophy or disruption [57]. Notably, in Figure 7b,c,h,i, the histological profile of the lymph from mice administered various fractions of *Mitragyna speciosa* extract appeared comparable to that of the normal control group. These observations suggest that Kratom extract did not compromise the immune microarchitecture or lymphoid function of the spleen, even in mice previously subjected to morphine-induced immunosuppression. Therefore, it can be inferred that the administration of Kratom fractions preserved splenic immune integrity, supporting its safety in terms of immune function during opioid withdrawal conditions.

However, Figure 7d,f presented altered lymph histoarchitecture, characterized by abnormal predominance of white pulp and evidence of lymphoid tissue disruption. This disproportionate expansion of white pulp may indicate localized lymphoproliferative activity, immune dysregulation, or potential inflammatory response. Additionally, fragmentation and irregular borders within lymphoid nodules suggest structural compromise of the immune microenvironment. These findings highlight the possibility that certain Kratom fractions, or dosing thresholds, may induce unfavourable immunological changes and warrant further investigation to delineate dose-dependent effects and histological correlates.

The spleen organ index, representing spleen-to-body weight ratio, is an established biomarker of immunological activity [58]. As shown in Figure 7, all morphine-exposed groups exhibited elevated spleen indices compared to the normal control, indicating increased immunological demand. Fraction 3 demonstrated the smallest increase, suggesting balanced immunomodulation. This fraction may exert anti-inflammatory or homeostatic effects that mitigate morphine-induced splenic hyperactivation.

**Figure 8:**
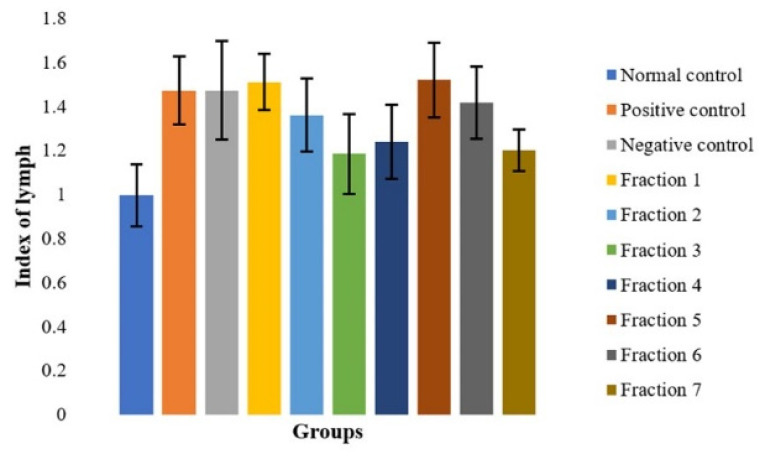
Spleen Organ Index in morphine-addicted mice following treatment with fractions of Kratom extract. Data are presented as mean ± SD (n = 5). Normal group (NaCMC 0.1%) (p.o), Positive group (Naloxone) subcutaneously (s.c) (0.00104 mg/20 g body weight) at the end of the test, Negative group (Morphine) (p.o) (10–30 mg/kg body weight with gradually increasing doses).

Figure 8 presents the spleen organ index measurements in mice after administration of morphine and subsequent treatment with various fractions of *Mitragyna speciosa* extract. Microscopic evaluation of spleen tissues (Figure 7) revealed morphological changes consistent with immune activation in mice exposed to chronic morphine administration. These histopathological findings are corroborated by the spleen organ index data presented in Figure 8. The spleen index, which reflects relative organ weight normalized to body weight, is a commonly used indicator of immunological and hematological responses to pharmacological interventions. All groups that received morphine, followed by treatment with fractions of Kratom (*Mitragyna speciosa*) extract, demonstrated a notable increase in spleen weight compared to the normal control group. This increase is indicative of splenic hyperactivity, likely due to enhanced immunological demand and the systemic effects of morphine-induced stress. Among the treatment groups, the Fraction 3 group exhibited the lowest increase in spleen index, suggesting a potentially more balanced or less stimulatory impact on splenic function compared to other extract fractions.

These findings imply that certain kratom fractions may modulate the immune response to morphine-induced physiological stress differently. The reduced spleen enlargement observed in the Fraction 3 group could be attributed to its anti-inflammatory or immunomodulatory properties, which may have mitigated the overactivation of the spleen often associated with chronic opioid exposure. All treatment groups exhibited higher spleen index values compared to the normal group, with Fraction 3 showing relatively lower values; however, these differences should be interpreted cautiously due to data variability. Further investigation into the specific bioactive compounds present in Fraction 3 may elucidate mechanisms contributing to this observed effect.

#### 2.8.2. Hepatic Histopathology and Biochemical Markers

Evaluation of biochemical, morphometric, and histopathological liver (Table 4) parameters demonstrated that most Kratom fractions did not induce a significant pattern of hepatotoxicity. The liver index across all treatment groups remained within the physiological range (3–5%) [59], indicating that exposure to these fractions did not cause hepatomegaly or hepatic atrophy in the majority of animals.

Serum SGOT and SGPT levels (Table 4) were also within the normal physiological range for male Swiss Webster mice, with SGOT at 40–100 IU/L and SGPT at 20–60 IU/L [60,61]. Elevations above the normal limits were observed only in a single mouse each in Fraction 6 and Fraction 7 groups. These responses were idiosyncratic and likely attributable to individual susceptibility or metabolic variability [62]. Macroscopic examination further revealed darkened hepatic lobes in the Fraction 6 mouse with a markedly elevated SGOT value. However, the mean SGOT level of the group and the Ishak score (2) remained within the safe range, providing no evidence of systemic hepatotoxicity.

**Figure 9:**
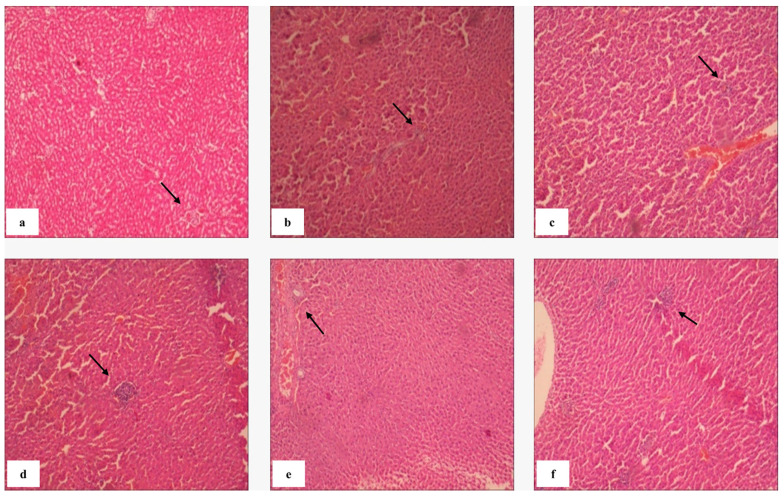
Histopathology of liver (**a**) normal control, (**b**) negative control, (**c**) fraction 2, (**d**) fraction 4, (**e**) fraction 6, (**f**) fraction 7, with microscopic magnification of 10×. Arrows indicate areas of hepatic fibrosis, characterized by fibrous tissue deposition within hepatic architecture.

Fibrosis analysis provided a more comprehensive assessment of long-term hepatic safety. The Ishak system classifies fibrosis into no fibrosis (0), mild (1–2), moderate (3–4), and advanced (5–6) [63]. In this study, Fractions 2, 4, and 7 exhibited an Ishak score of 1 (Figure 9), indicating mild fibrosis that is reversible and non-progressive once exposure is discontinued [64]. In contrast, the positive control (morphine) group showed the most severe injury, characterized by 5.05% centrilobular necrosis, 2.21% fibrosis, and an Ishak score of 3 (moderate fibrosis). This outcome is consistent with literature describing morphine-induced oxidative stress, Kupffer cell activation, and hepatic inflammation leading to substantial structural damage [64].

Interestingly, the Kratom fractions reduced necrosis (Fractions 2, 4, 6, and 7: 4.17%, 3.59%, 2.75%, and 4.02%, respectively), inflammation (Fractions 2, 4, 6, and 7: 0.01%, 0.68%, 0.45%, and 0.29%, respectively), and fibrosis (Fractions 2, 4, and 7: 1.23%, 1.23%, and 1.12%, respectively) compared with the morphine-exposed group, suggesting a relative hepatoprotective effect or at least an absence of exacerbation of morphine-induced injury. For comparison, the normal group exhibited necrosis, inflammation, and fibrosis levels of 0.01%, 3.96%, and 0.58%, respectively. These findings are aligned with reports indicating that various phytochemicals attenuate morphine-induced toxicity through modulation of oxidative stress and inflammatory pathways [64].

Importantly, SGOT levels do not necessarily correlate with the degree of fibrosis, particularly at early stages (scores 1–2), as early collagen deposition may occur without marked elevations in transaminases [64]. Therefore, combining SGOT and SGPT levels with Ishak scores, liver index, and histopathological findings provides a more accurate profile of hepatic toxicity. In this study, the integrated assessment indicated that the liver injury observed was mild, non-progressive, and not accompanied by active neuroinflammatory processes. Overall, despite isolated findings of elevated SGOT or focal necrosis, the Kratom fractions tested did not provide strong evidence of systemic hepatotoxicity. The very low levels of inflammation (<1%) [65,66,67], necrosis below 5% [68], and minimal fibrosis indicate that hepatic injury was mild and likely reversible. From a toxicological perspective, fractions presenting mild SGOT/SGPT elevations and low necrosis should be considered potential hepatotoxic candidates for further evaluation, such as collagen staining, immunohistochemistry of stellate cell activation, or long-term exposure studies. Conversely, fractions demonstrating normal biochemical and histopathological parameters may be regarded as relatively safe for short-term use in animal models.

#### 2.8.3. Renal Histopathology (Nephroprotective Activity)

Morphine administration resulted in pronounced glomerular lesions (33.33%) and tubular degeneration (597), confirming nephrotoxic injury consistent with elevated oxidative stress markers and suppressed antioxidant enzymes [63]. Treatment with fractions notably Fractions 2, 5, and 6 completely abolished glomerular lesions (0%) and preserved tubular integrity, suggesting nephroprotective activity (Table 5) [69].

**Figure 10:**
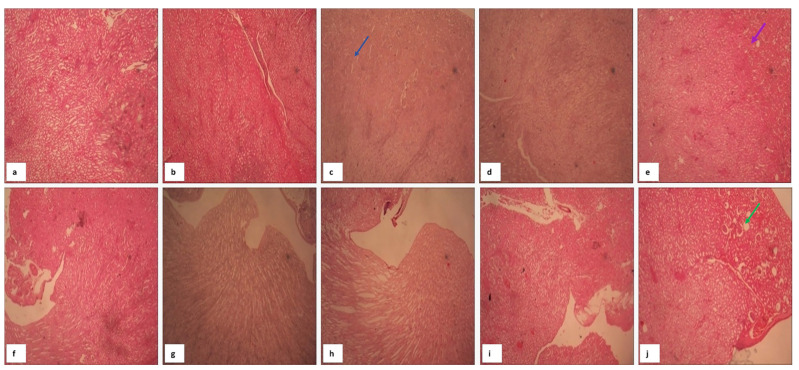
Effect of kratom on the renal histopathology analysis. (**a**) normal control, (**b**) negative control, (**c**) fraction 1, (**d**) fraction 2, (**e**) fraction 3, (**f**) fraction 4, (**g**) fraction 5, (**h**) fraction 6, (**i**) fraction 7, and (**j**) positive control with microscopic magnification of 40×. Data are presented were analyzed descriptively. Note, blue arrow: glomerulus; green arrow: Bowman’s capsule; purple arrow: tubules.

Histopathological analysis of kidney tissue across all experimental groups revealed no substantial alterations in renal architecture (Figure 10). The microscopic structures of the glomeruli, Bowman’s capsules, and renal tubules remained intact and well-defined in all samples. There were no indications of necrosis, interstitial inflammation, or thickening of the glomerular or tubular basement membranes. Additionally, the epithelial lining of the tubules appeared normal, without evidence of cellular degeneration, swelling, or detachment. These findings suggest that administration of the kratom extract fractions, including in morphine-induced animals, did not induce observable nephrotoxicity under the study conditions. The absence of structural renal damage supports the renal safety profile of the tested fractions, at least at the administered doses and durations. This aligns with previous reports indicating that certain natural compounds with immunomodulatory or analgesic properties may have low nephrotoxic potential when used appropriately [61,70]. Further biochemical assessments, such as BUN and creatinine levels, would be valuable to complement these histological observations and confirm renal function preservation.

## 3. Materials and Methods

### 3.1. Materials

#### 3.1.1. Plant Material

The Kratom (*Mitragyna speciosa*) leaves were collected from Kota Bangun, East Kalimantan, Indonesia in August 2024. Furthermore, the plant was identified by Prof. Dr. Paulus Matius, M.Sc, Head of Mulawarman Herbarium, Laboratory of Ecology and Conservation of Tropical Forest Biodiversity, Faculty of Forestry, Mulawarman University, Samarinda, East Kalimantan, Indonesia. Finally, a voucher specimen (No. 197/UN17.4.08/LL/2024) was deposited at the Herbarium.

#### 3.1.2. Reagents

Phosphate-buffered saline (PBS), pH 7.4; Hank’s Balanced Salt Solution (HBSS); Collagenase Type IV (1 mg/mL; Sigma-Aldrich, St. Louis, MO, USA); DNase I (0.1 mg/mL; Sigma-Aldrich); Percoll™ (GE Healthcare, Uppsala, Sweden) or Ficoll-Paque™ for gradient centrifugation; RPMI-1640 or DMEM (with 10% FBS, 1% penicillin/streptomycin); Fluorochrome-conjugated monoclonal antibodies (e.g., anti-CD11b, CD45, GFAP, TLR4, IL-1β, IL-6); Fixation/Permeabilization solution (e.g., BD Cytofix/Cytoperm™); Fc block (anti-CD16/CD32; BD Biosciences, San Jose, CA, USA); Flow cytometry tubes and 40 µm cell strainers; Viability dye (e.g., 7-AAD or Zombie Aqua™); BD FACSVerse™ (San Jose, CA, USA), BD LSRFortessa™ (San Jose, CA, USA), or equivalent flow cytometer; FlowJo™ (v10) or FCS Express software (v7.28.0035) for analysis.

#### 3.1.3. Animals

Male Swiss Webster mice (weighing 20–25 g, age 2–3 months) were obtained and acclimatized under standard laboratory conditions (25 ± 2 °C, 12 h light/dark cycle) with free access to food and water. All experimental procedures were approved by Health Research Ethics Commission, Faculty of Pharmacy, Mulawarman University, Samarinda, East Kalimantan, Indonesia with Description of Ethical Exemption Voucher Number: 179/KEPK-FFUNMUL/EC/EXP/11/2023, signed by Chairperson: Dr. apt. Riski Sulistiarini, M.Si.

### 3.2. Methods

#### 3.2.1. Preparation of Plant Extracts

Dried leaf material of Kratom (*Mitragyna speciosa)* was subjected to extraction using the maceration method with 96% ethanol as the solvent. Briefly, the dried leaves were ground into a fine powder using a mechanical grinder and passed through a 40-mesh sieve to ensure uniform particle size. A total of 500 g of the powdered material was weighed and transferred into a clean, sterile glass container. Subsequently, 2.5 L of 96% ethanol (1:5 *w*/*v* ratio) was added to fully immerse the plant material. The mixture was sealed and allowed to macerate at room temperature (25–28 °C) for 72 h with intermittent shaking every 12 h to ensure maximum solvent penetration and diffusion of phytochemicals. After 72 h, the mixture was filtered using muslin cloth followed by Whatman No. 1 filter paper to separate the liquid extract from the plant residue. The marc (plant residue) was subjected to two additional maceration cycles under the same conditions to ensure complete extraction. All filtrates were pooled and concentrated under reduced pressure using a rotary evaporator at 40 °C to remove ethanol, yielding a viscous crude extract. The resulting extract was stored at 4 °C in an amber-coloured bottle until further fractionation. Detailed procedures for plant material processing, extraction, and fractionation are provided in Appendix A.1.

#### 3.2.2. Fractionation of Kratom Leaf Ethanol Extract by Vacuum Liquid Chromatography (VLC)

The crude ethanol extract of *Mitragyna speciosa* leaves was subjected to fractionation using Vacuum Liquid Chromatography (VLC) to separate compounds based on polarity. The stationary phase used was silica gel 60 (Merck 1.07734.1000, Darmstadt, Germany), packed into a VLC column under vacuum to ensure even distribution and compaction. The extract was applied onto the top of the silica bed, and elution was carried out sequentially using a gradient system of increasing polarity. The mobile phase consisted of a series of gradient solvent systems, beginning with 100% n-hexane (F0), which was discarded due to negligible compound elution. Subsequent fractions were collected using the following solvent systems: F1: n-Hexane:Ethyl acetate (70:30, *v*/*v*); F2: n-Hexane:Ethyl acetate (50:50, *v*/*v*); F3: Chloroform:Methanol (70:30, *v*/*v*); F4: Chloroform:Methanol (50:50, *v*/*v*); F5: Chloroform:Methanol (30:70, *v*/*v*); F6: Methanol (100%); F7: Methanol (80%). Each fraction was collected in clean, labelled glass tubes, evaporated under reduced pressure using a rotary evaporator at 40 °C, and stored at 4 °C for further analysis. Monitoring of compound separation was performed using Thin-Layer Chromatography (TLC) on silica gel 60 F254 plates. The mobile phase for TLC was n-Hexane:Ethyl acetate (60:40, *v*/*v*). Plates were visualized under UV light at 254 nm and 366 nm and sprayed with appropriate reagents for detection of phytoconstituents (e.g., Dragendorff’s reagent for alkaloids). Collected fractions were further characterized using UV–Visible Spectrophotometry (UV-Vis) to detect chromophoric groups and High-Performance Liquid Chromatography (HPLC) to confirm compound identity and purity. HPLC analysis was carried out with reference standards such as mitragynine, using a C18 reverse-phase column and appropriate gradient elution conditions. A tabulated summary of the solvent systems used in VLC fractionation is provided in the Supplementary Materials.

#### 3.2.3. Metabolites Identification Through UHPLC-Q-Exactive Plus Orbitrap HRMS

Sample preparation: 5 mg of fraction was dissolved in 1 mL of MeOH, then filtered with a 0.2 μm Nylon membrane; Method used—LC-MS: UHPLC Vanquish Tandem Q Exactive Plus Orbitrap HRMS ThermoScientific; Column: Accucore C18, 100 × 2.1 mm, 1.5 μm (ThermoScientific); Flow Rate: 0.2 mL/min; Eluent: H2O + 0.1% formic acid (A) and acetonitrile + 0.1% formic acid (B); Gradient: 0–1 min (5% B), 1–25 min (5–95% B), 25–28 min (95% B), and 28–33 min (5% B); Column temperature: 30 °C; Injection Volume: 2 μL; Mass range: 100–1500 *m*/*z*; Ionization mode: positive and negative; Data processing: using online databases from ChemSpider and mzCloud. The detailed UHPLC–HRMS analytical conditions are described in Appendix A.2.

#### 3.2.4. Molecular Investigating the Binding Affinity to TLR4 Protein (PDB ID: 4G8A)

The molecular interactions between key Kratom alkaloids and TLR4 were investigated using Maestro Glide (Schrödinger Suite) to evaluate their potential as TLR4 inhibitors. The TLR4/MD2 complex structure (PDB ID: 4G8A) was employed as the receptor for docking studies. Protein preparation was performed using the Protein Preparation Wizard, which included the removal of water molecules, addition of hydrogen atoms, optimization of the protein structure, and elimination of ions, solvents, and co-ligands. The three-dimensional structures of Kratom alkaloids were prepared using LigPrep at pH 7.0 ± 0.5. The active site of TLR4 was defined based on literature-reported key residues (PHE126, PHE119, ILE124, TYR102, SER120, HIS155, ARG90, and GLU92) and used to generate the receptor grid. Docking simulations were conducted using the Glide module in both Standard Precision (SP) and Extra Precision (XP) modes. Docking results were evaluated based on GlideScore values and the nature of ligand–receptor interactions. The binding poses and molecular interactions of the ligand–protein complexes were visualized and analyzed using Maestro ((Schrödinger Release 2025-1) and PyMOL (version 3.1, Schrödinger, LLC) to assess the orientation of the ligands and the residues involved in binding.

#### 3.2.5. In Vivo Evaluation of *Mitragyna speciosa* (Kratom) as a Non-Tolerant and Non-Dependent Opioid Analgesic in Morphine-Addicted Mice

1. Preparation of test solutions

Morphine Sulphate was dissolved in distilled water; Naloxone Hydrochloride (injection solution, 0.4 mg/mL) was diluted to 0.4 mg/50 mL using sterile Water for Injection (WFI). Based on a target dose of 0.00104 mg per 20 g BW, the injection volume was adjusted to 0.3 mL per 20 g BW.

2. Dose determination

All administered doses were calculated in mg/kg BW and represent crude extract or fraction weight without normalization to individual alkaloids. The dose selection for the Kratom fractions in this study was grounded in an ethnopharmacological framework that reflects the traditional consumption patterns of Kratom in Southeast Asia. Contemporary ethnobotanical surveys report that habitual users typically consume 5–10 fresh leaves per intake, corresponding to approximately 2–8 g of dried leaf material per day, depending on the method of preparation (chewing, brewing, or powder ingestion) and frequency of use [71,72]. To ensure translational relevance, the estimated human daily consumption (0.03–0.1 g/kg BW of dried leaf equivalent) was converted to a murine equivalent dose using the standard body surface area (BSA) scaling factor (Km ratio: human 37; mouse 3). Based on this interspecies conversion, the equivalent murine exposure corresponds to 3–10 mg/kg BW of extract-equivalent material. Consequently, the selected dose of 4 mg/kg BW for each Kratom fraction falls within this physiologically relevant bracket, representing a moderate and non-toxic range in alignment with real-world human usage. The selected dosage reflects chronic low-to-moderate exposure, consistent with traditional consumption patterns rather than acute high-dose intake, thereby increasing ecological validity.

While the selected dose (4 mg/kg BW) was derived using body surface area conversion to reflect ethnopharmacological relevance, the present study did not establish concentration–response relationships or determine receptor-specific IC50 values. Furthermore, molecular docking scores represent theoretical binding affinities and do not directly equate to pharmacologically active concentrations in vivo. Future studies incorporating receptor-binding assays and dose–response analyses are warranted to define precise pharmacodynamic thresholds.

3. Experimental Protocol for Evaluating Kratom’s Effects

Mice were randomly assigned into ten groups (n = 5–6 per group) as follows: Group 1 (Normal/Vehicle Control) received 0.1% NaCMC (p.o.) which is widely used as a standard vehicle in preclinical studies due to its excellent safety profile and physiological inertness [73,74,75]; Groups 2–8 (Test Groups 1–7) received Kratom extract Fractions 1–7, respectively, at 4 mg/kg body weight (BW), p.o.; Group 9 (Positive Control) received naloxone at 0.00104 mg/20 g BW subcutaneously (s.c.); and Group 10 (Negative Control) received morphine sulphate at 10–30 mg/kg BW, p.o., administered in an escalating regimen to confirm the establishment of dependency. The study was conducted for 14 days, during which all test groups and the positive control were subjected to morphine induction twice daily following an escalating dosing protocol to induce dependence (10 mg/kg BW on days 1–5, 20 mg/kg BW on days 6–10, and 30 mg/kg BW on days 11–14). Orally administered substances were delivered at 0.5 mL/20 g BW using 0.1% NaCMC as the vehicle, and body weight was monitored daily. All animals were monitored for behavioural changes indicative of opioid dependence and withdrawal symptoms (including ataxia, catalepsy, piloerection, and index of fear responsiveness), as well as behavioural despair, endurance, and muscular strength (using the Forced Swimming Test/FST and the Traction Test), and acute nociceptive responses (Tail Pinch Test) throughout the experimental period, with observations conducted on days 2, 7, and 14.

Statistical Analysis of Behavioural Data. Behavioural parameters including ataxia, piloerection, and fear response were recorded as categorical variables (presence/absence). Due to the binary nature of these data and low event frequency, Fisher’s exact test was used to evaluate differences between groups at each observation time point (days 2, 7, and 14). Catalepsy data, recorded as numerical duration/score, were analyzed descriptively. Tail pinch test data, recorded as numerical latency values (seconds), were analyzed using one-way ANOVA for each observation time point (days 2, 7, and 14), followed by Dunnett’s post hoc test using the negative control (morphine-treated group) as the reference comparator. Statistical significance was defined as *p* < 0.05.

On day 15, all animals were euthanized; brains were collected for cytokine quantification via flow cytometry, and the liver, kidneys, and spleen were harvested for histopathological examination and for the measurement of organ indices. Blood samples were obtained via intracardiac puncture for SGOT and SGPT analysis.

4. Behavioural despair, endurance, and muscular strength assessment

The Forced Swimming Test (FST) and the Traction Test were conducted to assess behavioural despair, endurance, and muscular strength in mice. In the FST, each mouse was individually placed in a transparent cylindrical glass container (height: 25 cm, diameter: 10 cm) filled with water to a depth of 15 cm (25 ± 1 °C) to prevent tail contact with the bottom and underwent a single 6 min trial. Total immobility time, defined as the period during which the animal ceased struggling and floated with minimal movement to keep its head above water, was recorded during the final 4 min following a 2 min acclimatization period. Increased immobility was interpreted as behavioural despair or reduced endurance, whereas decreased immobility indicated higher stamina or a potential antidepressant-like effect. Water was changed between trials to prevent olfactory cues from influencing performance. In the Traction Test, used to evaluate muscular strength and coordination often impaired during opioid withdrawal, each mouse was suspended by its forepaws on a horizontally placed metal wire (1 mm diameter) stretched between two vertical stands at a height of 30 cm. The test was initiated by gently placing the mouse’s forelimbs on the wire, allowing it to hang freely, and a positive response was recorded if the mouse could raise at least one hind limb and reach the wire within 5 s; failure to do so or falling from the wire was recorded as a negative response. Traction performance was calculated as the percentage of animals showing a positive response in each group. Both tests were conducted on Day 14 of morphine exposure and repeated on Day 21 after sample administration under blinded conditions to eliminate observer bias. Behavioural testing and scoring were performed by two independent observers blinded to treatment allocation. The behavioural assessment parameters are summarized in Appendix A (Table A1).

5. Acute Nociceptive Response Assessment

Acute nociceptive responses were evaluated using the tail pinch test. Mice were acclimatized in a quiet room for 30 min prior to testing. Each mouse was gently restrained, and the distal third of the tail was pinched using calibrated forceps with sufficient pressure to elicit nociceptive stimulation without causing tissue injury. Nociceptive response, defined as rapid tail withdrawal, flicking, or vigorous movement, was recorded as the latency from stimulus onset to response using a digital stopwatch.

6. Isolation and Flow Cytometric Analysis of Mouse Brain Cells

Mice were euthanized in accordance with institutional ethical guidelines, and brains were rapidly dissected and placed in cold Hank’s Balanced Salt Solution (HBSS). The tissue was minced into approximately 1 mm^3^ fragments and enzymatically digested in a solution containing Collagenase IV (1 mg/mL) and DNase I (0.1 mg/mL) for 30–45 min at 37 °C with gentle shaking, followed by mechanical dissociation using a fire-polished Pasteur pipette or a gentleMACS™ dissociator (Miltenyi Biotec, Germany). The homogenate was filtered through a 40 µm cell strainer into a 15 mL conical tube, and mononuclear cells were isolated by density gradient centrifugation using 30%/70% Percoll™ at 800× *g* for 30 min without brake, with the interface layer subsequently collected. Cells were counted using a haemocytometer or an automated cell counter, and viability was assessed by Trypan Blue exclusion or using a viability dye such as Zombie Aqua™ or 7-AAD. Aliquots of 1–2 × 10^6^ cells were transferred to FACS tubes, and Fc receptors were blocked using anti-CD16/CD32 antibodies for 10 min at 4 °C. Cells were stained for surface antigens with appropriate fluorochrome-conjugated antibodies (e.g., CD11b, CD45, TLR4) in FACS buffer (PBS supplemented with 2% FBS) for 30 min at 4 °C in the dark, followed by two washes with FACS buffer. For intracellular cytokine staining (IL-6 and IL-1β), cells were fixed and permeabilized using BD Cytofix/Cytoperm™ solution according to the manufacturer’s instructions and incubated with intracellular antibodies for 30 min at room temperature, after which cells were washed and resuspended in 300 µL of FACS buffer for acquisition. Flow cytometric acquisition was performed on a BD flow cytometer, collecting a minimum of 10,000–50,000 events per sample, with unstained, single-stained, and fluorescence-minus-one (FMO) controls used to set gates and compensation. Data were analyzed using FlowJo™ or equivalent software, gating on single, live cells to identify immune and glial populations (e.g., CD11b^+^CD45^+^ for microglia/macrophages) and to quantify expression levels of TLR4, TNF-α, IL-1β, and other relevant markers. The gating strategy and analysis workflow are provided in Appendix A.3.

7. Histopathological Analysis

At the end of the experimental period, mice were euthanized under appropriate anesthesia in accordance with institutional ethical guidelines, and organs of interest, including the spleen, brain, kidneys, and liver, were carefully dissected and immediately immersed in 10% neutral-buffered formalin (NBF) for 24–48 h at room temperature to ensure complete fixation. Fixed tissues were washed with running tap water, dehydrated through a graded ethanol series (70%, 80%, 90%, 95%, and absolute ethanol), cleared in xylene, and embedded in molten paraffin wax using a standard tissue processor. Tissue blocks were trimmed and sectioned into 4–5 µm slices using a rotary microtome. Paraffin sections were mounted on glass slides, deparaffinized in xylene, and rehydrated through a descending ethanol series to distilled water, followed by Haematoxylin and Eosin (H&E) staining to visualize general tissue architecture. After staining, slides were dehydrated, cleared, and mounted with DPX mounting medium for light microscopy. Stained sections were examined under a light microscope at magnifications of 10× and 40×, and histopathological features were evaluated by a trained pathologist blinded to treatment allocation. Representative photomicrographs were captured using a digital camera mounted on the microscope, processed with ImageJ software 1.54k for clarity and scale calibration, and annotated to highlight key features for inclusion in the results section. The histopathological assessment, performed using the Optilab Viewer 4.0 application, included Ishak score, percentage of necrosis, inflammation, and fibrosis for the liver; percentage of edema and necrosis for the spleen; and the number of renal tubules and percentage of glomerular lesions for the kidneys in only one replication.

8. Experimental Procedures

All experimental procedures were conducted following standardized laboratory protocols to ensure reproducibility and methodological transparency. Detailed descriptions of extraction, fractionation, analytical profiling, molecular docking parameters, animal handling, behavioural testing, flow cytometry gating strategies, and histopathological scoring are provided to facilitate independent replication.

9. Limitations and Translational Considerations

Several limitations should be acknowledged. This study is limited by the withdrawal animal model employed, as the stepwise morphine dose-escalation protocol could not be extended beyond 14 days. Beyond this period, withdrawal symptoms intensified markedly and were associated with a high risk of mortality, thereby restricting observations to the acute and subacute phases of opioid withdrawal. Consequently, the findings may not fully capture the characteristics or neurobiological consequences associated with prolonged or chronic opioid withdrawal states. The observed cytokine modulation primarily reflects microglial activity; however, additional studies are required to distinguish contributions from other glial cell populations. Formal evaluation of reinforcing properties requires additional paradigms, including self-administration studies, conditioned place preference, and reinforcement-based behavioural testing, which were beyond the scope of the present study. Additionally, molecular docking provides predictive interaction modelling that requires biochemical validation through receptor-binding assays or functional TLR4 inhibition studies. Therefore, the present findings establish mechanistic plausibility for neuroimmune modulation associated with kratom fractions but do not constitute definitive evidence regarding addiction liability or long-term therapeutic equivalence to classical opioid analgesics. The present study focuses on functional and mechanistic evaluation of tolerance- and dependence-related outcomes based on integrated behavioural, neuroimmune, and histopathological evidence. While pharmacokinetic interactions, including those involving cytochrome P450 isoenzymes, may influence drug disposition under certain conditions, they were not directly evaluated in this study. Therefore, additional investigations addressing pharmacokinetic interactions and long-term safety under different exposure conditions are warranted.

## 4. Conclusions

The present investigation provides a comprehensive, multi-level evaluation of *Mitragyna speciosa* (Kratom) ethanol extract, integrating metabolomic, molecular docking, immunopharmacological, behavioural, and histopathological analyses to elucidate its therapeutic potential and safety profile. Metabolomic profiling identified multiple alkaloids and flavonoids most notably mitragynine, 7-hydroxymitragynine, rhynchophylline, speciogynine, quinine ethyl carbonate, deacetylvindoline, rutin, and isoquercetin as key bioactive constituents capable of modulating TLR4-mediated neuroinflammatory pathways central to opioid dependence and tolerance.

Molecular docking studies demonstrated that rutin and isoquercetin exhibited the strongest and most functionally relevant interactions with the TLR4/MD2 complex, engaging critical residues such as GLU92, TYR102, and SER120, thus supporting Kratom’s proposed anti-neuroinflammatory and TLR4-inhibitory mechanisms. In contrast, mitragynine, while showing a higher binding affinity, interacted with non-essential residues (PHE76, PHE151), indicating limited contribution to direct TLR4 inhibition.

At the cellular level, flow cytometric and gene expression profiling confirmed that specific Kratom fractions downregulated pro-inflammatory cytokines (NF-κB, IL-1β, and IL-6) in glial cells, demonstrating suppression of microglial activation. Mice treated with a kratom fraction showed significantly increased tail-pinch response latency, demonstrating antinociceptive activity comparable to morphine. This effect aligns with evidence that mitragynine and 7-hydroxymitragynine act as partial μ-opioid receptor agonists, indicating kratom’s potential as a centrally acting analgesic.

Among the evaluated fractions, Fraction 3 appears to represent the most promising candidate, as it demonstrated a relatively consistent and balanced profile across multiple experimental parameters, including suppression of pro-inflammatory cytokines (NF-κB, IL-1β, and IL-6), improvement in behavioural outcomes in morphine-dependent mice, and a stable immunohistopathological profile. While other fractions, particularly Fraction 7, exhibited strong effects in specific assays such as attenuation of withdrawal symptoms and splenic protection, these effects were less consistent across all evaluated endpoints. Nevertheless, these findings should be interpreted within the limitations of the current study, and further validation through targeted compound quantification, mechanistic assays, and dose-standardized evaluations is required to confirm the therapeutic potential of the identified fractions. At the organ level, fractions 2, 5, and 6 provided notable nephroprotective effects, preserving renal architecture and tubular integrity, while fraction 3 maintained immune homeostasis with minimal splenic hyperactivation. Conversely, fractions 2, 4, and 7 exhibited mild, reversible hepatic alterations, including localized necrosis and low-grade fibrosis (Ishak score 1–2), indicating the need for dose optimization and long-term toxicity monitoring. Histopathological observations of increased lymphoid cell infiltration further suggest an immunomodulatory role, reflecting Kratom’s capacity to restore immune balance following morphine-induced suppression.

Collectively, these findings confirm that Kratom’s pharmacological and safety profiles are fraction-dependent. Specific fractions exert potent TLR4 inhibitory, anti-inflammatory, and immunorestorative actions that may alleviate opioid withdrawal related neuroinflammation, whereas others may induce mild, reversible organ stress at higher doses. Functionally, the extract’s dual mechanism combining partial μ-opioid receptor agonism with TLR4 inhibition underpins its unique potential as a non-addictive opioid analgesic alternative.

This integrative evidence establishes a strong mechanistic basis for Kratom’s use in pain management and opioid withdrawal therapy, emphasizing the importance of fraction-based standardization, mechanistic validation, and translational safety profiling. Further clinical studies are warranted to define optimal dosing parameters, verify long-term safety, and enable the development of Kratom-derived therapeutic agents capable of addressing the global opioid dependence crisis with reduced risk of addiction and tolerance.

## Figures and Tables

**Table 1:** Metabolite Profile of Kratom (*Mitragyna speciosa*) Leaf Ethanol Extract.

No	Name	Formula	Annot. Delta Mass [ppm]	Calc. MW	RT [min]	Area Sample
**1**	Mitragynine	C23 H30 N2 O4	−2.5	398.2196	11.843	88,171,780,539
**2**	Mitragynine	C23 H30 N2 O4	−2.5	398.2196	12.649	36,906,641,389
**3**	Rhynchophylline	C22 H28 N2 O4	−3.09	384.2037	10.14	25,954,534,995
**4**	Oxospeciogynine (Methyl 2beta,3beta,4beta,5alpha,12beta,19alpha)-3,4,16-trihydroxy-1-methyl-6,7-didehydroaspidospermidine-3-carboxylate)	C22 H28 N2 O5	−2.68	400.1988	9.571	25,713,232,423
**5**	Quinine ethyl carbonate (Ethyl (4beta,8alpha,9R)-6′-methoxycinchonan-9-yl carbonate)	C23 H28 N2 O4	−2.68	396.2038	13.295	17,637,557,433
**6**	Oxospeciogynine (Methyl 2beta,3beta,4beta,5alpha,12beta,19alpha)-3,4,16-trihydroxy-1-methyl-6,7-didehydroaspidospermidine-3-carboxylate)	C22 H28 N2 O5	−2.68	400.1988	9.897	15,019,150,043
**7**	Mitragynine	C23 H30 N2 O4	−2.5	398.2196	12.394	13,698,326,168
**8**	Rhynchophylline	C22 H28 N2 O4	−3.09	384.2037	10.707	10,137,779,684
**9**	Deacetylvindoline	C23 H30 N2 O5	−2.36	414.2145	11.188	7,950,391,710
**10**	Oxospeciogynine (Methyl (2beta,3beta,4beta,5alpha,12beta,19alpha)-3,4,16-trihydroxy-1-methyl-6,7-didehydroaspidospermidine-3-carboxylate	C22 H28 N2 O5	−2.68	400.1988	9.236	6,553,914,659
**11**	Cianidanol	C15 H14 O6	−1.12	290.0787	7.242	5,513,117,072
**12**	Mitragynine	C23 H30 N2 O4	−2.5	398.2196	13.074	4,522,475,648
**13**	Deacetylvindoline	C23 H30 N2 O5	−2.36	414.2145	11.004	3,634,828,390
**14**	-	C17 H23 N10 P	−2.71	398.1834	9.305	3,348,871,466
**15**	Rutin	C27 H30 O16	−0.93	610.1528	8.481	2,994,147,274
**16**	Quinic acid (1,3,4,5-Tetrahydroxycyclohexanecarboxylic acid)	C7 H12 O6	−3.51	192.0627	1.128	2,715,463,523
**17**	6beta-hydroxygeniposide	C17 H24 O11	−0.63	404.1316	7.672	2,493,713,228
**18**	Chlorogenic acid	C16 H18 O9	−0.89	354.0948	6.136	2,295,478,416
**19**	Tofisopam	C22 H26 N2 O4	−1.2	382.1888	10.028	2,271,401,681
**20**	Deacetylvindoline	C23 H30 N2 O5	−2.36	414.2145	10.559	2,104,129,222
**21**	(-)-Brucine	C23 H26 N2 O4	−0.93	394.1889	13.425	2,081,162,162
**22**	Choline	C5 H13 N O	1.96	103.0999	1.047	2,068,900,456
**23**	Pheophorbide A	C35 H36 N4 O5	−1.07	592.2679	26.628	2,005,647,331
**24**	-	C23 H28 N2 O5	−3.64	412.1983	10.138	1,913,601,179
**25**	Isoquercetin	C21 H20 O12	−0.76	464.0951	8.786	1,890,449,637
**26**	D-Gluconic acid	C6 H12 O7	−3.27	196.0577	1.068	1,877,954,476
**27**	(+)-Procyanidin B2	C30 H26 O12	−0.93	578.1419	6.829	1,809,608,510
**28**	hirsuteine	C22 H26 N2 O3	−2.55	366.1934	12.453	1,318,649,752
**29**	Tofisopam	C22 H26 N2 O4	−1.2	382.1888	9.593	1,234,706,155
**30**	-	C23 H28 N2 O5	−1.2	412.1993	10.836	1,223,319,317
**31**	Citric acid	C6 H8 O7	−1.73	192.0267	1.125	979,561,379.1
**32**	(-)-Brucine	C23 H26 N2 O4	−0.93	394.1889	13.129	969,488,507.7
**33**	Mitraphylline (Methyl (2beta,3beta,4beta,5alpha,12beta,19alpha)-3,4,16-trihydroxy-1-methyl-6,7-didehydroaspidospermidine-3-carboxylate)	C22 H28 N2 O5	−2.68	400.1988	11.272	919,002,547.8
**34**	-	C36 H38 N4 O5	−1.3	606.2834	28.906	893,998,933.7
**35**	beta-d-glucose pentaacetate	C16 H22 O11	−0.23	390.1161	6.158	822,824,505.1
**36**	Gambiriin A1	C30 H28 O12	−0.09	580.158	7.239	817,228,454.8
**37**	-	C22 H28 N2 O6	−1.35	416.1942	10.628	814,896,855.5
**38**	Genipinic acid (2-(3-Hydroxy-3,4,5,6-tetrahydro-1H-cyclopenta[c]furan-4-yl)-3-methoxy-3-xopropanoic acid)	C11 H14 O6	−1.87	242.0786	7.672	746,351,521.8
**39**	-	C46 H60 N4 O8	−0.8	796.4405	11.833	663,910,299.2
**40**	5-Caffeoylshikimic Acid	C16 H16 O8	−0.09	336.0845	7.241	609,735,251.4
**41**	(E)-p-coumaric acid	C9 H8 O3	−1.27	164.0471	7.681	571,213,729.6
**42**	Phenylglyoxylic acid	C8 H6 O3	−1.32	150.0315	7.672	514,246,094.6
**43**	1-O-acetyl-alpha-maltose	C14 H24 O12	−0.22	384.1267	1.068	512,845,764.2
**44**	-	C14 H20 N4 O9	−3.61	388.1216	1.066	511,073,073.8
**45**	Sinapinic acid	C11 H12 O5	−1.86	224.0681	7.672	489,734,626.7
**46**	-	C44 H64 N4 O13	−2.37	856.445	10.761	479,571,997.4
**47**	-	C7 H12 O8	−1.75	224.0528	1.142	475,859,275.4
**48**	Samidorphan	C21 H26 N2 O4	−1.82	370.1886	8.42	474,441,474.6
**49**	Hexaric acid	C6 H10 O8	−2.41	210.0371	1.107	460,928,420.1
**50**	Procyanidin C1	C45 H38 O18	0.08	866.2059	7.554	409,371,560.4
**51**	-	C23 H40 N5 O6 P3	−1.56	575.2183	8.759	407,148,768.1
**52**	(+)-Procyanidin B2	C30 H26 O12	−0.94	578.1419	8.631	386,300,859.1
**53**	γ-Mangostin (gamma-mangostin) (6,10,11,13-Tetrahydroxy-9-isopropenyl-3,3-dimethyl-8,9-dihydro-3H,7H-benzo[c]pyrano[3,2-h]xanthen-7-one)	C25 H22 O7	−0.51	434.1363	18.305	361,706,572.3
**54**	Artonin E	C25 H24 O7	−0.51	436.152	18.785	347,849,605.4
**55**	3-Oxoglycyrrhetic acid	C30 H44 O4	−1.04	468.3235	11.956	340,221,423.8
**56**	Furfuryl thioalkenyl aldehyde derivative (2Z)-3-{5-[(4Z)-5-(Methylsulfanyl)-4-penten-2-yn-1-yl]-2-furyl}acrylaldehyde)	C13 H12 O2 S	0.28	232.0559	1.161	335,370,299.7
**57**	3-BHA	C11 H16 O2	−0.72	180.1149	13.965	328,966,325.5
**58**	Pheophorbide A	C35 H36 N4 O5	−1.08	592.2679	27.243	328,749,015.9
**59**	Hex-2-ulose	C6 H12 O6	−3.75	180.0627	1.067	328,029,299.7
**60**	Folic Acid (N-(4-{[(2-Amino-4-oxo-1,4,5,6,7,8-hexahydro-6-pteridinyl)methyl]amino}benzoyl)-gamma-glutamylglutamic acid)	C24 H30 N8 O9	4.79	574.2163	8.757	320,125,597.7
**61**	Ensulizole	C13 H10 N2 O3 S	−0.16	274.0412	5.996	312,749,198.9
**62**	13-KODE	C18 H30 O3	−1.33	294.2191	20.709	312,657,191.2
**63**	Trilaurylamine	C36 H75 N	−1.87	521.589	30.435	290,135,958
**64**	-	C38 H53 N3 O	−0.31	567.4187	29.439	288,775,423.5
**65**	L-(+)-Valine	C5 H11 N O2	1.13	117.0791	1.089	279,866,001.4
**66**	13-KODE	C18 H30 O3	0.63	294.2197	18.981	277,401,129.4
**67**	-	C41 H69 N4 O10 P S	3.36	840.45	11.953	266,722,735.5
**68**	Protoporphyrin IX (3,3′,3″-(3,8,13,17-Tetramethyl-12-vinyl-2,7,18-porphyrintriyl)tripropanoic acid)	C35 H36 N4 O6	−2.12	608.2622	26.076	263,145,385.9
**69**	Quinaprilat	C23 H26 N2 O5	−2.04	410.1833	13.348	263,064,430.2
**70**	Quillaic Acid	C30 H46 O5	−1.2	486.3339	10.759	253,864,323.5
**71**	Vanilloyl-lupanine (7alpha,13beta)-2-Oxospartein-13-yl 3,5-dihydroxy-4-methoxybenzoate)	C23 H30 N2 O6	−1.3	430.2098	11.712	251,476,065.2
**72**	MFCD00036904	C24 H50 N O7 P	−0.83	495.3321	19.834	242,049,757
**73**	-	C15 H18 N6 O9	−0.53	426.1133	7.67	239,811,663.2
**74**	Samidorphan	C21 H26 N2 O4	−1.82	370.1886	8.788	237,701,814
**75**	-	C37 H32 N4 O15	−1.77	772.1851	10.138	237,309,137.3
**76**	-	C13 H24 O12	−0.23	372.1267	1.091	229,355,522.7
**77**	Nictoflorin	C27 H30 O15	0.09	594.1585	8.861	229,141,554.9
**78**	Zingerol	C11 H16 O3	−0.72	196.1098	9.123	228,927,714.2
**79**	IN00458	C9 H6 O3	−1.88	162.0314	6.135	22,663,5806
**80**	Nictoflorin	C27 H30 O15	0.08	594.1585	9.078	226,266,246.3
**81**	Vanilloyl Lupanine (7alpha,13beta)-2-Oxospartein-13-yl 3,5-dihydroxy-4-methoxybenzoate)	C23 H30 N2 O6	−1.3	430.2098	9.773	221,643,600.6
**82**	-	C14 H18 N4 O8	−4.02	370.111	1.121	218,873,560.3
**83**	Mosapride	C21 H25 Cl F N3 O3	2.46	421.1579	7.671	212,470,446
**84**	-	C22 H26 N2 O5	−1.95	398.1834	10.385	209,483,402.1
**85**	Diphenhydramine N-glucuronide (3R,4S,5S,6S)-6-Carboxy-N-[2-(diphenylmethoxy)ethyl]-3,4,5-trihydroxy-N,N-dimethyltetrahydro-2H-pyran-2-aminium (non-preferred name)	C23 H30 N O7	−6.08	432.1996	7.005	207,451,235.8
**86**	O-DESMETHYLCARVEDILOL	C23 H24 N2 O4	−1.26	392.1731	13	207,316,788.9
**87**	Adenine	C5 H5 N5	0.08	135.0545	1.125	204,609,543.2
**88**	Oxospeciogynine (Methyl (2beta,3beta,4beta,5alpha,12beta,19alpha)-3,4,16-trihydroxy-1-methyl-6,7-didehydroaspidospermidine-3-carboxylate)	C22 H28 N2 O5	−2.68	400.1988	8.872	203,143,262.2
**89**	Methyl 14-hydroxy-14,15-dihydroeburnamenine-14-carboxylate	C21 H26 N2 O3	−2.12	354.1936	8.549	197,611,617.8
**90**	Boldenone Undecylenate	C30 H44 O3	−1.25	452.3285	17.935	197,062,096.5
**91**	-	C46 H60 N4 O8	−0.8	796.4405	12.66	195,206,004.1
**92**	3-O-feruloyl-D-quinic acid	C17 H20 O9	−0.11	368.1107	8.169	194,758,987
**93**	-	C23 H43 N4 O P	1.04	422.3179	10.758	194,300,041.5
**94**	Phenylglyoxylic acid	C8 H6 O3	−1.32	150.0315	6.156	192,676,000.5
**95**	-	C20 H30 N4 O13	−2.77	534.1795	1.087	190,099,054.6
**96**	D-Glucono-delta-lactone	C6 H10 O6	−2.94	178.0472	1.147	189,658,308
**97**	DL-Malic acid	C4 H6 O5	−6.78	134.0206	1.148	185,254,990.3
**98**	3-O-feruloyl-D-quinic acid	C17 H20 O9	−0.13	368.1107	7.663	185,006,972.9
**99**	Silydianin (3-Hydroxy-10-(4-hydroxy-3-methoxyphenyl)-8-(3,5,7-trihydroxy-4-oxo-3,4-dihydro-2H-chromen-2-yl)-4 oxatricyclo[4.3.1.0~3,7~]decan-2-one)	C25 H24 O10	0.6	484.1372	8.987	184,176,432.7
**100**	MUD	C16 H18 O8	0.18	338.1002	7.194	184,142,484.4

**Table 2:** Molecular Docking of Kratom (*Mitragyna speciosa*) Leaf Ethanol Extract Compounds with TLR4 protein (PDB ID: 4G8A).

No.	Ligand	Docking Score	Interaction	Active Site (Reference)	Image
**1**	Mitragynine	−6.341	PHE76, PHE151	PHE126, PHE 119, ILE124, TYR102, SER120, HIS155, ARG90, GLU92	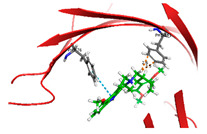
					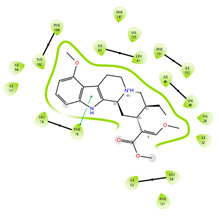
					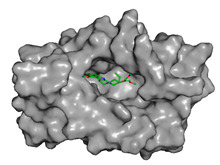
**2**	Hirsuteine	−5.579	CYS133, LEU61, PHE 151	PHE126, PHE 119, ILE124, TYR102, SER120, HIS155, ARG90, GLU92	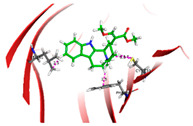
**3**	Rutin	−5.36	GLU92, SER120, TYR102	PHE126, PHE 119, ILE124, TYR102, SER120, HIS155, ARG90, GLU92	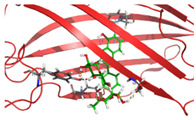
**4**	Gambiriin A1	−5.335	TYR102, ASP101, GLU92, VAL93, ILE94	PHE126, PHE 119, ILE124, TYR102, SER120, HIS155, ARG90, GLU92	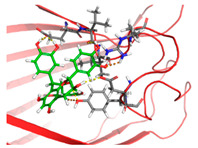
**5**	Isoquercetin	−5.237	TYR102, VAL93, GLU92, SER120	PHE126, PHE 119, ILE124, TYR102, SER120, HIS155, ARG90, GLU92	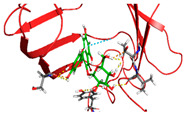
**6**	(+)-Procyanidin B2	−4.808	VAL93, SER120, SER118	PHE126, PHE 119, ILE124, TYR102, SER120, HIS155, ARG90, GLU92	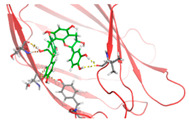
**7**	Cianidanol	−4.697	TYR 102	PHE126, PHE 119, ILE124, TYR102, SER120, HIS155, ARG90, GLU92	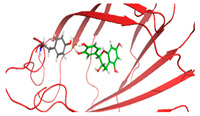
**8**	Rhynchophylline	−4.338	SER120	PHE126, PHE 119, ILE124, TYR102, SER120, HIS155, ARG90, GLU92	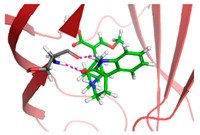
**9**	6beta-hydroxygeniposide	−4.05	GLU92, ASP101, TYR102	PHE126, PHE 119, ILE124, TYR102, SER120, HIS155, ARG90, GLU92	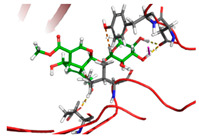
**10**	1-O-acetyl-alpha-maltose	−3.965	SER118, SER120, TYR120, PHE119	PHE126, PHE 119, ILE124, TYR102, SER120, HIS155, ARG90, GLU92	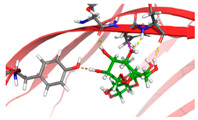
**11**	Ethyl (4beta,8alpha,9R)-6′-methoxycinchonan-9-yl carbonate	−3.937	PHE121, GLU92	PHE126, PHE 119, ILE124, TYR102, SER120, HIS155, ARG90, GLU92	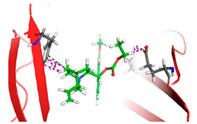
**12**	Chlorgenic acid	−3.536	TYR102, SER120	PHE126, PHE 119, ILE124, TYR102, SER120, HIS155, ARG90, GLU92	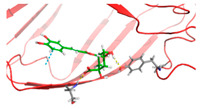
**13**	Hex-2-ulose	−2.484	TYR102, ILE117	PHE126, PHE 119, ILE124, TYR102, SER120, HIS155, ARG90, GLU92	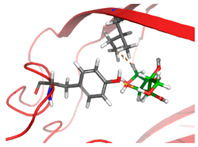
**14**	D-gluconic acid	−2.129	SER120, TYR102	PHE126, PHE 119, ILE124, TYR102, SER120, HIS155, ARG90, GLU92	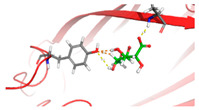

**Table 3:** Percentage of Edema, Necrosis, and Spleen Index in Mice Following Treatment with different *Mitragyna speciosa* leaf fractions.

Group	Spleen *		
	% Edema	% Necrosis	Spleen Index (%)
Fraction 1	1.31	1.57	1.51 ± 0.53
Fraction 2	0.99	1.41	1.36 ± 0.17
Fraction 3	0.68	1.17	1.18 ± 0.18
Fraction 4	1.00	1.38	1.24 ± 0.17
Fraction 5	1.02	1.59	1.52 ± 0.17
Fraction 6	1.15	0.00	1.42 ± 0.26
Fraction 7	0.65	0.87	1.2 ± 0.1
Negative Control	1.40	2.65	1.47 ± 0.22
Normal Control	0.65	1.14	0.99 ± 0.64
Positive Control	-	-	1.47 ± 0.26

* Edema and necrosis values represent semi-quantitative histopathological scoring; therefore, standard deviations are not applicable.

**Table 4:** Liver index (%) and serum liver enzyme levels (SGOT and SGPT) in mice treated with different *Mitragyna speciosa* leaf fractions.

Group	Liver		
	Liver Index (%)	SGOT (IU/L)	SGPT (IU/L)
Fraction 1	4.3 ± 1.32	55.5 ± 39.7	60.45 ± 16.97
Fraction 2	4.6 ± 0.3	65.65 ± 23.5	23.05 ± 7.93
Fraction 3	4.7 ± 0.6	37.67 ± 13.6	25.23 ± 11.57
Fraction 4	4.7 ± 0.3	45.33 ± 23.12	36.31 ± 14.50
Fraction 5	5.4 ± 0.8	71.16 ± 18.05	58.93 ± 18.97
Fraction 6	3.7 ± 0.24	95.11 ± 74.6	43.25 ± 20.83
Fraction 7	4.2 ± 0.1	80.12 ± 45.9	63.36 ± 12.63
Negative Control	4.7 ± 0.6	47.45 ± 5.24	24.90 ± 15.84
Normal Control	4.6 ± 0.2	66.7 ± 2.84	24.77 ± 12.25
Positive Control	4.7 ± 0.6	95.27 ± 2.6	34.02 ± 5.59

**Table 5:** Percentage of Glomerular Lesion, Number of Tubules, and Kidney Index in Mice Following Treatment with different *Mitragyna speciosa* leaf fractions.

Group	Kidney		
	No. Tubules	% Glomerular Lesion	Kidney Index (%)
Fraction 1	295	17.5	1.5 ± 0.53
Fraction 2	373	0	1.36 ± 0.17
Fraction 3	431	10	1.36 ± 0.18
Fraction 4	413	10	1.18 ± 0.17
Fraction 5	320	0	1.24 ± 0.17
Fraction 6	269	0	1.52 ± 0.26
Fraction 7	538	12.5	1.42 ± 0.09
Negative Control	597	33.33	1.47 ± 0.22
Normal Control	364	0	1.24 ± 0.22
Positive Control	364	21.05	1.47 ± 0.26

**Figure A1:**
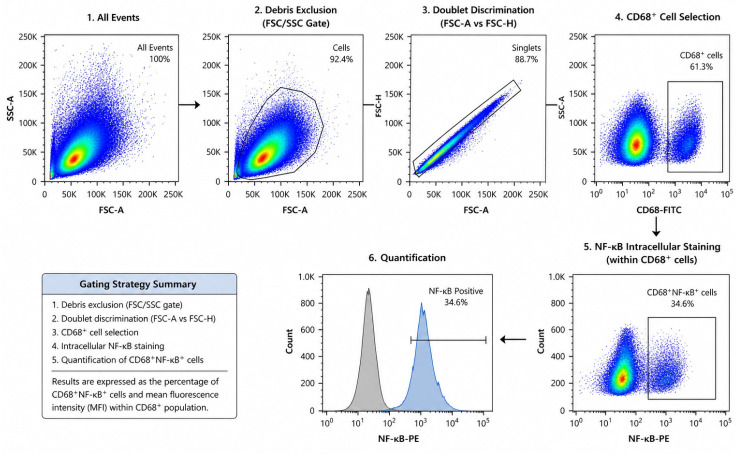
Representative flow cytometry gating strategy for identification of CD68^+^NF-κB^+^ cells.

## Data Availability

The datasets generated and analyzed during this study are available from the corresponding author upon reasonable request. The data supporting the findings, including behavioural assessment data, flow cytometry analyses, histopathological evaluations, and metabolomic profiling results, are not publicly available due to institutional data management policies and ethical considerations related to animal research but can be provided for scientific verification and reproducibility purposes upon request. No publicly archived datasets were used in this study.

## Supplementary Materials

The following supporting information can be downloaded at: https://www.mdpi.com/article/10.3390/molecules31091428/s1.

## Abbreviations

The following abbreviations are used in this manuscript:

BSA	Body Surface Area
CD68	Cluster of Differentiation 68
CNS	Central Nervous System
DDA	Data-Dependent Acquisition
DOR	Delta-Opioid Receptor
FACS	Fluorescence-Activated Cell Sorting
FST	Forced Swimming Test
GABA	Gamma-Aminobutyric Acid
HRMS	High-Resolution Mass Spectrometry
IL-1β	Interleukin-1 beta
IL-6	Interleukin-6
Km	Body Surface Area Conversion Factor
KOR	Kappa-Opioid Receptor
LPS	Lipopolysaccharide
MD-2	Myeloid Differentiation Protein-2
MOR	Mu-Opioid Receptor
*M. speciosa*	*Mitragyna speciosa*
NF-κB	Nuclear Factor kappa B
NaCMC	Sodium Carboxymethyl Cellulose
p.o.	Per Oral (oral administration)
s.c.	Subcutaneous
SGOT	Serum Glutamic Oxaloacetic Transaminase (AST)
SGPT	Serum Glutamic Pyruvic Transaminase (ALT)
SP	Standard Precision (Docking Mode)
TLR4	Toll-Like Receptor 4
UHPLC	Ultra-High-Performance Liquid Chromatography
UHPLC–HRMS	Ultra-High-Performance Liquid Chromatography–High-Resolution Mass Spectrometry
XP	Extra Precision (Docking Mode)

## Appendix A. Experimental and Analytical Supplementary Details

### Appendix A.1. Plant Material Processing and Extraction Procedure

Dried leaves of *Mitragyna speciosa* were pulverized into a fine powder prior to extraction. The plant material was extracted by maceration using 96% ethanol at room temperature for 72 h with intermittent agitation. The solvent-to-sample ratio was maintained at 10:1 (*v*/*w*). The extract was filtered and concentrated under reduced pressure using a rotary evaporator at temperatures below 45 °C to prevent thermal degradation of alkaloids. The crude extract was subjected to Vacuum Liquid Chromatography (VLC) using silica gel as stationary phase. Elution was performed using gradient solvent systems of increasing polarity consisting of n-hexane–ethyl acetate followed by chloroform–methanol mixtures. Fractions were pooled based on TLC similarity.

### Appendix A.2. UHPLC–HRMS Analytical Parameters

Metabolite profiling was performed using UHPLC coupled with Q-Exactive Orbitrap High-Resolution Mass Spectrometry under the following conditions:

1. Column: C18 reverse-phase (2.1 × 100 mm, 1.7 μm);
2. Mobile phase A: Water with 0.1% formic acid;
3. Mobile phase B: Acetonitrile with 0.1% formic acid;
4. Flow rate: 0.3 mL min^−1^;
5. Injection volume: 5 μL;
6. Ionization: Electrospray ionization (ESI+);
7. Scan range: *m*/*z* 100–1000;
8. Acquisition mode: Data-Dependent Acquisition (DDA).

Compound annotation was performed using accurate mass measurements and fragmentation matching against available spectral databases.

### Appendix A.3. Flow Cytometry Analysis and Gating Strategy

Single-cell suspensions were analyzed using flow cytometry following exclusion of debris via forward scatter (FSC) and side scatter (SSC) gating. Microglial populations were identified based on CD68 expression, followed by intracellular staining for NF-κB.

The gating workflow consisted of:

1. Debris exclusion (FSC/SSC gate);
2. Doublet discrimination;
3. CD68-positive cell selection;
4. Quantification of NF-κB fluorescence intensity.

**Table A1:** Behavioural Assessment Parameters Used in the Study.

Assay	Measured Variable	Endpoint	Interpretation
Tail Pinch Test	Latency response	Seconds	Antinociceptive effect
Forced Swimming Test	Swimming duration	Immobility time	Endurance response
Traction Test	Grip duration	Holding time	Motor coordination
Withdrawal Score	Behavioural signs	Composite index	Withdrawal severity

## Appendix B. Supplementary Computational and Translational Notes

### Appendix B.1. Molecular Docking Computational Settings

Molecular docking was conducted using the Schrödinger Glide (Schrödinger Release 2025-1) module with Standard Precision (SP) and Extra Precision (XP) modes. The crystal structure of the TLR4/MD2 complex (PDB ID: 4G8A) was prepared using Protein Preparation Wizard including hydrogen addition, bond order assignment, and energy minimization.

Grid generation was centred on the MD2 binding pocket. Ligands were optimized using LigPrep (Schrödinger Release 2025-1) prior to docking. Docking scores are reported as GlideScore values representing predicted binding affinity.

### Appendix B.2. Dose Conversion Method

The experimental dose (4 mg kg^−1^ BW) was determined using body surface area (BSA) conversion according to standard interspecies scaling:

Animal Dose = Human Equivalent Dose × (Km_human_/Km_mouse_)

where Km factors follow FDA-recommended conversion values.

### Appendix B.3. Experimental Model Limitations

The morphine dose-escalation protocol was limited to 14 days due to increased withdrawal severity and mortality risk beyond this period. Therefore, experimental observations represent acute to subacute withdrawal phases. Long-term dependence models were not evaluated within the present study.
