# Supplementary material for: Kratom (Mitragyna speciosa) as a Phytochemical-Based Natural Product Exhibiting Opioid-like Analgesic Effects with Reduced Tolerance and Dependence Liability via TLR4-Associated Neuroimmune Modulation
Source: Molecules. 2026 Apr 26;31(9):1428. doi: 10.3390/molecules31091428 (PMC13164666; doi:10.3390/molecules31091428)
Supplement: Supplementary file 1 [file molecules-31-01428-s001.zip › Behavioral_Observations_English_Complete.pdf]

Behavioral Observations

Complete English Translation of Tables and Data Interpretations

All numerical values are preserved exactly as in the source document.

1. Ataxia

Group	Day 0	Day 7 (sec)	Day 14 (sec)
Fraction 1	0	0,2 ± 0,5	0
Fraction 2	0	0	0
Fraction 3	0	0,2 ± 0,5	0
Fraction 4	0	0	0
Fraction 5	0	0,2 ± 0,4	0,2 ± 0,4
Fraction 6	0	0,3 ± 0,6	0,7 ± 0,6
Fraction 7	0	0,5 ± 0,6	0
Normal	0	0	0
Positive Control	0	0,6 ± 0,5	0,2 ± 0,4
Negative Control	0	0	0

Data interpretation:

- Ataxia symptoms were detected on Day 7 in the positive control group, Fraction 1, Fraction 3, Fraction 5, Fraction 6, and Fraction 7. These symptoms persisted only in Fraction 5, Fraction 6, and the positive control group.
- It is provisionally assumed that the ataxia symptoms emerged as a result of morphine induction. These symptoms may have disappeared due to multicompound treatment in Fractions 1, 3, and 7.
- Treatment with Fractions 2 and 4 was able to prevent ataxia, as no such symptoms were detected on Days 7 and 14.

2. Grip Strength Test

Group	Day 2 (sec)	Day 7 (sec)	Day 14 (sec)
Fraction 1	60,00 ± 0,00	49,20 ± 24,1	54,60 ± 19,40
Fraction 2	41,80 ± 26,00	60,00 ± 0,00	60,00±0,00
Fraction 3	45,80 ± 20,81	37,60 ± 30,67	62,6 ± 2,41
Fraction 4	46,75 ± 26,50	60,00 ± 0,00	61,5 ± 3,00
Fraction 5	45,33 ±26,94	49,83 ± 21,22	64 ± 7,27
Fraction 6	70,00 ± 0,00	63,33 ± 2,89	66,33 ± 2,52
Fraction 7	54,25 ± 18,77	52,75 ± 30,48	71,5 ± 2,12
Normal	62,50 ± 2,89	66,55 ± 8,54	68,00 ± 31,54
Positive Control	29,60 ± 28,08	40,60 ± 26,68	41,80 ± 29,47
Negative Control	60,00 ± 0,00	49,25 ± 21,5	60,00 ± 0,00

Data interpretation:

- Neuromuscular coordination in mice can be measured using the traction test. The non-induced normal group maintained consistent grip strength for 1 minute on Days 2, 7, and 14.
- Fraction 1 was able to maintain grip strength on Day 2, showed a slight decline on Day 7, and improved again on Day 14.
- Fractions 2 and 4 showed reduced grip strength on Day 2, but recovered on Days 7 and 14.
- Fractions 3, 5, and 7 showed reduced grip strength on Days 2 and 7, but recovered on Day 14.
- Fraction 6 was able to maintain grip strength at all three observed time points.
- In contrast, the induced untreated control group showed reduced grip strength at all three observed time points.

3. Forced Swimming Test

Group	Day 2 (sec)	Day 7 (sec)	Day 14 (sec)
Fraction 1	66,75±16,15	17,15±11,50	13,40±11,87
Fraction 2	83,4±54,47	47,66±66,11	22,00±24,04
Fraction 3	71,8±32,16	22,00±19,48	11,20±6,42
Fraction 4	46,75±33,99	72,33±28,02	61,25±67,43
Fraction 5	58,00±16,82	12,50±14,25	13,00 ± 11,29
Fraction 6	80,00±45,17	4,33±1,53	13,50±4,95
Fraction 7	30,75±29,66	11,00±8,19	11,00±0,00
Normal	65,33±39,26	42,00±63,01	67,00±48,50
Positive Control	51,75±10,21	18,00±8,00	94,67±79,74
Negative Control	37,75±16,54	82,25±77,44	34,25±21,64

Data interpretation:

- Endurance on Day 14 was lower in Groups 4 and 2.

4. Tail Suspension Test

Group	Day 2 (sec)	Day 7 (sec)	Day 14 (sec)
Fraction 1	12,60±5,59	7,75±5,50	2,33±1,53
Fraction 2	8,20±4,21	4,33±1,52	5,00±2,30
Fraction 3	9,20±8,93	3,60±1,82	3,20±2,17
Fraction 4	8,75±7,04	7,33±1,52	3,33±2,31
Fraction 5	10,00±7,03	5,67±4,96	3,33±0,58
Fraction 6	6,67±2,89	3,33±1,15	3,50±0,71
Fraction 7	7,25±8,62	7,00±6,92	2,50±1,71
Normal	11,25±7,68	6,00±2,65	3,67±1,52

Group	Day 2 (sec)	Day 7 (sec)	Day 14 (sec)
Positive Control	7,80±3,49	3,40±1,14	4,33±2,08
Negative Control	10,00±1,63	5,5±2,08	6,00±3,46

Data interpretation:

- On Day 14, Groups 1 and 7 showed faster responses than the normal group. Groups 3, 4, and 5 were close to normal.

5. Body Weight (g)

Group	Day 1	2	3	4	5	6	7	8	9	10	11	12	13	14
Fraction 1	30,04±3,85	27,40±3,29	31,40± 2,88	29,60±2,41	28,80±3,27	27,60±3,05	27,20±3,56	26,40±3,36	25,80±3,83	25,80±3,83	25,20±4,32	24,80±4,99	24,60±5,13	24,60±4,72
Fraction 2	29,20±2,95	29,80±2,78	27,60±4,93	30,80±2,49	30,80±2,59	30,80±2,59	30,20±2,49	30,60±2,88	30,00±2,88	29,60±3,21	29,80±3,19	29,60±3,91	29,40±3,91	29,00±3,94
Fraction 3	28,00±4,00	27,80±4,32	25,00±2,31	28,20±4,32	29,20±4,32	28,60±4,16	28,20±4,44	28,80±4,66	28,80±4,66	28,80±4,02	29,20±4,32	28,60±3,85	28,40±4,83	28,40±4,16
Fraction 4	24,75±2,63	25,25±2,63	27,33±5,05	25,75±2,63	25,50±2,38	26,00±2,58	26,75±2,99	27,50±3,11	27,5±3,11	27,50±2,08	27,50±2,08	27,00±2,45	26,00±2,71	26,00±2,94
Fraction 5	29,00±6,36	27,83±5,64	28,00±00	28,00±4,82	27,83±5,64	28,33±5,78	27,83±4,99	28,83±5,60	28,83±5,60	28,33±6,31	27,83±6,73	27,33±7,12	28,17±7,47	26,83±6,85
Fraction 6	26,00±2,83	23,50±2,12	31,00±5,29	26,33±3,79	24,33±4,62	23,00±4,36	22,33±4,62	22,00±5,20	22,00±5,29	22,00±5,20	21,00±5,20	20,67±5,77	20,67±5,77	19,67±5,77
Fraction 7	31,25±5,25	31,25±5,50	31,00±5,29	30,50±5,80	32,00±4,97	32,25±5,31	31,50±5,19	31,50±5,20	32,00±4,97	30,75±4,65	31,00±4,97	31,25±4,86	31,00±4,76	30,50±4,93
Normal	28,75±5,06	28,75±5,06	29,25±5,62	31,25±4,99	30,50±5,26	30,25±4,57	28,75±4,03	31,25±4,03	31,75±4,11	32,00±4,24	31,75±3,86	31,75±2,06	32,25±2,22	31,75±2,06
Positive Control	26,00±5,10	26,00±5,10	27,00±4,74	27,00±5,61	28,00±5,79	28,00±5,79	28,60±5,12	28,00±5,48	28,20±5,12	28,00±4,64	26,40±5,27	27,20±4,71	26,40±5,41	27,20±4,32
Negative Control	29,00±6,00	29,00±6,00	28,75±6,95	30,25±7,72	31,75±7,13	31,75±7,13	32,00±7,62	32,25±7,18	31,25±6,40	31,25±5,62	32,25±5,06	32,50±5,20	31,00±6,05	30,75±3,59

Data interpretation:

- Body weight stability was generally good in all groups, except Group 6, in which a marked toxic effect on the liver was confirmed. Therefore, Group 6 was excluded from the selection of potentially promising fractions due to this toxic effect.

6. Aggressive Behavior

Group	Day 2	Day 7	Day 14
Fraction 1	0,50±0,71	1,00±0,00	1,00±0,00
Fraction 2	0,50±0,71	1,00±0,00	1,00±0,00
Fraction 3	0,80±0,45	1,00±0,00	0,80±0,45
Fraction 4	1,00±0,00	1,00±0,00	1,00±0,00
Fraction 5	0,67±0,58	0,67±0,52	1,00±0,00
Fraction 6	1,00±0,00	1,00±0,00	0,67±0,58
Fraction 7	1,00±0,00	0,75±0,50	0,75±0,50
Normal	1,00±0,00	1,00±0,00	1,00±0,00
Positive Control	1,00±0,00	0,60±0,54	0,80±0,45
Negative Control	1,00±0,00	1,00±0,00	0,75±0,50

Data interpretation:
• Fraction 6 caused the mice to become more aggressive and was therefore excluded.
• There was no significant difference in aggressive behavior between the control and fraction-treated groups.

7. Active Behavior

Group	Day 2	Day 7	Day 14
Fraction 1	0,60±0,55	1,00±0,00	1,00±0,00
Fraction 2	0,60±0,55	1,00±0,00	1,00±0,00
Fraction 3	0,80±0,45	1,00±0,00	0,80±0,45
Fraction 4	0,75±0,50	1,00±0,00	0,75±0,50
Fraction 5	0,83±0,41	0,83±0,41	0,80±0,45
Fraction 6	0,50±0,71	1,00±0,00	0,67±0,58
Fraction 7	1,00±0,00	0,67±0,58	1,00±0,00
Normal	0,83±0,41	1,00±0,00	1,00±0,00
Positive Control	1,00±0,00	0,60±0,55	0,80±0,45
Negative Control	1,00± 0,00	1,00±0,00	0,75±0,50

Data interpretation:
• Fraction 6 caused the mice to become more active and was therefore excluded.
• There was no significant difference in active behavior between the control and fraction-treated groups.

8. Diarrhea

Group	Day 2	Day 7	Day 14
Fraction 1	0,40±0,55	1,00±0,00	0,60±0,55
Fraction 2	0,80±0,45	1,00±0,00	0,60±0,55

Group	Day 2	Day 7	Day 14
Fraction 3	0,80±0,45	0,80±0,45	1,00±0,00
Fraction 4	0,33±0,57	0,75±0,50	1,00±0,00
Fraction 5	0,83±0,41	1,00±0,00	0,80±0,45
Fraction 6	0,67±0,58	0,67±0,58	0,67±0,58
Fraction 7	1,00±0,00	1,00±0,00	0,50±0,58
Normal	0,75±0,50	1,00±0,00	0,75±0,50
Positive Control	0,80±0,45	1,00±0,00	0,20±0,45
Negative Control	1,00±0,00	0,25±0,50	1,00±0,00

Data interpretation:
• There was a potential increase in peristaltic activity in Groups 3, 4, and the negative control group.

9. Piloerection (Hair Standing)

Group	Day 2	Day 7	Day 14
Fraction 1	0,60±0,55	0,75±0,50	0,80±0,45
Fraction 2	0,50±0,58	0,25±0,50	0,80±0,45
Fraction 3	0,80±0,45	1,00±0,00	1,00±0,00
Fraction 4	0,75±0,50	1,00±0,00	1,00±0,00
Fraction 5	0,40±0,55	1,00±0,00	1,00±0,00
Fraction 6	0,67±0,58	1,00±0,00	1,00±0,00
Fraction 7	0,50±0,58	1,00±0,00	1,00±0,00
Normal	0,75±0,50	0,00±0,00	0,50±0,58
Positive Control	0,60±0,55	1,00±0,00	0,80±0,45
Negative Control	0,25±0,50	0,75±0,50	1,00±0,00

Data interpretation:
• There was no difference between the control and treatment groups.

10. Straub Response (Erect Tail)

Group	Day 2	Day 7	Day 14
Fraction 1	0,20±0,45	0,00±0,00	0,40±0,55
Fraction 2	0,20±0,45	0,67±0,58	0,40±0,55
Fraction 3	0,40±0,55	0,80±0,45	0,20±0,45
Fraction 4	0,25±0,50	1,00±0,00	0,75±0,50
Fraction 5	1,00±0,00	1,00±0,00	0,67±0,52
Fraction 6	0,33±0,58	0,33±0,58	0,33±0,58

Group	Day 2	Day 7	Day 14
Fraction 7	0,75±0,50	0,33±0,58	0,25±0,50
Normal	0,75±0,50	0,67±0,58	0,75±0,50
Positive Control	0,60±0,55	0,20±0,45	0,80±0,45
Negative Control	1,00±0,00	0,50±0,58	0,25±0,50

Data interpretation:

- The Straub response, an opiate-like effect, appeared more frequently in the normal and positive control groups. This was considered irrational; therefore, these data were disregarded.

11. Rapid Tail Rotation

Group	Day 2	Day 7	Day 14
Fraction 1	0,00±0,00	0,00±0,00	0,00±0,00
Fraction 2	0,00±0,00	0,00±0,00	0,20±0,45
Fraction 3	0,00±0,00	0,00±0,00	0,00±0,00
Fraction 4	0,00±0,00	0,00±0,00	0,00±0,00
Fraction 5	0,17±0,41	0,00±0,00	0,00±0,00
Fraction 6	0,00±0,00	0,00±0,00	0,00±0,00
Fraction 7	0,00±0,00	0,00±0,00	0,00±0,00
Normal	0,00±0,00	0,33±0,58	0,00±0,00
Positive Control	0,00±0,00	0,00±0,00	0,00±0,00
Negative Control	0,00±0,00	0,00±0,00	0,00±0,00

Data interpretation:

- There was no difference.

12. Grooming / Face-Rubbing Response

Group	Day 2	Day 7	Day 14
Fraction 1	0,20±0,45	0,00±0,00	0,00±0,00
Fraction 2	0,20±0,45	0,67±0,58	0,2±0,45
Fraction 3	0,40±0,55	0,40±0,55	0,20±0,45
Fraction 4	0,75±0,50	0,67±0,58	0,75±0,50
Fraction 5	0,50±0,55	0,17±0,41	0,00±0,00
Fraction 6	0,00±0,00	0,33±0,58	0,00±0,00
Fraction 7	0,25±0,50	0,00±0,00	0,00±0,00
Normal	0,75±0,5	0,67±0,58	0,25±0,50
Positive Control	0,40±0,55	0,20±0,45	0,00±0,00
Negative Control	0,50±0,58	0,75±0,50	0,25±0,50

Data interpretation:
• Fractions 1, 5, 6, and 7 did not induce grooming behavior.

13. Circular Movement

Group	Day 2	Day 7	Day 14
Fraction 1	0,20±0,45	0,00±0,00	0,00±0,00
Fraction 2	0,20±0,45	0,33±0,58	0,00±0,00
Fraction 3	0,00±0,00	0,00±0,00	0,20±0,45
Fraction 4	0,50±0,58	0,00±0,00	0,00±0,00
Fraction 5	0,17±0,41	0,00±0,00	0,00±0,00
Fraction 6	0,00± 0,00	0,00±0,00	0,00±0,00
Fraction 7	0,25±0,50	0,00±0,00	0,00±0,00
Normal	0,50±0,58	0,00±0,00	0,25±0,50
Positive Control	0,20± 0,45	0,00±0,00	0,20±0,45
Negative Control	0,25±0,50	0,00±0,00	0,00±0,00

Data interpretation:
• There was no difference.

14. Skin Color

Group	Day 2	Day 7	Day 14
Fraction 1	0,00±0,00	0,00±0,00	0,00±0,00
Fraction 2	0,00±0,00	0,33±0,58	0,20±0,45
Fraction 3	0,20±0,45	0,00±0,00	0,00±0,00
Fraction 4	0,00±0,00	0,33±0,58	0,50±0,58
Fraction 5	0,17±0,41	0,00±0,00	0,40±0,55
Fraction 6	0,00±0,00	0,00±0,00	1,00±0,00
Fraction 7	0,00±0,00	0,00±0,00	0,00±0,00
Normal	0,00±0,00	0,00±0,00	0,00±0,00
Positive Control	0,40±0,55	0,20±0,45	0,40±0,55
Negative Control	0,25±0,50	0,25±0,50	1,00±0,00

Data interpretation:
• Fractions 1, 3, and 7 did not cause reddish discoloration of the skin.

15. Catalepsy

Group	Day 2 (sec)	Day 7 (sec)	Day 14 (sec)

Group	Day 2 (sec)	Day 7 (sec)	Day 14 (sec)
Fraction 1	4,20±4,49	8,00±6,58	4,60±2,51
Fraction 2	4,40±5,98	1,67±0,58	4,80±1,30
Fraction 3	6,00±2,83	4,00±3,32	4,8±2,39
Fraction 4	4,25±3,86	2,33±0,58	5,25±6,65
Fraction 5	7,50±7,59	4,67±3,20	3,00±1,41
Fraction 6	2,67±2,08	3,67±1,53	5,67±8,08
Fraction 7	1,00±0,00	8,67±7,37	3,25±1,50
Normal	7,75±9,07	2,33±0,58	3,50±2,38
Positive Control	13,80±19,96	4,20±1,48	7,80±7,50
Negative Control	6,00±7,07	2,75±2,06	3,25±2,63

Data interpretation:

• Groups 5 and 7 were close to the normal condition.

16. Convulsion

Group	Day 2	Day 7	Day 14
Fraction 1	0,00±0,00	0,00±0,00	0,00±0,00
Fraction 2	0,00±0,00	0,00±0,00	0,00±0,00
Fraction 3	0,20±0,45	0,00±0,00	0,00±0,00
Fraction 4	0,00±0,00	0,00±0,00	0,00±0,00
Fraction 5	0,00±0,00	0,00±0,00	0,00±0,00
Fraction 6	0,00±0,00	0,00±0,00	0,00±0,00
Fraction 7	0,00±0,00	0,00±0,00	0,00±0,00
Normal	0,00±0,00	0,00±0,00	0,00±0,00
Positive Control	0,40±0,55	0,00±0,00	0,20±0,45
Negative Control	0,00±0,00	0,00±0,00	0,00±0,00

Data interpretation:

• There was no difference.

17. Muscle Twitching

Group	Day 2	Day 7	Day 14
Fraction 1	0,80±0,45	0,75±0,50	0,60±0,55
Fraction 2	0,60±0,55	0,67±0,58	0,60±0,55
Fraction 3	0,00±0,00	0,80±0,45	0,60±0,55
Fraction 4	0,00±0,00	0,67±0,58	0,50±0,58

Group	Day 2	Day 7	Day 14
Fraction 5	0,50±0,55	1,00±0,00	0,67±0,52
Fraction 6	0,00±0,00	0,67±0,58	0,67±0,58
Fraction 7	0,25±0,50	0,67±0,58	0,50±0,58
Normal	0,00±0,00	1,00±0,00	0,75±0,50
Positive Control	0,20±0,45	1,00±0,00	0,80±0,45
Negative Control	0,00±0,00	1,00±0,00	1,00±0,00

Data interpretation:
• There was no difference.

18. Fear Response

Group	Day 2	Day 7	Day 14
Fraction 1	0,60±0,55	1,00±0,00	0,20±0,45
Fraction 2	0,60±0,55	0,00±0,00	0,40±0,55
Fraction 3	0,00±0,00	1,00±0,00	1,00±0,00
Fraction 4	0,25±0,50	0,00±0,00	0,25±0,55
Fraction 50,50	0,50±0,55	0,67±0,52	0,60±0,55
Fraction 6	0,33±0,58	0,33±0,58	1,00±0,00
Fraction 7	0,50±0,58	1,00±0,00	0,75±0,50
Normal	0,25±0,50	0,33±0,58	0,50±0,58
Positive Control	0,40±0,55	0,60±0,55	1,00±0,00
Negative Control	0,25±0,50	0,25±0,50	0,50±0,58

Data interpretation:
• Groups 1 and 2 were more responsive.

19. Lacrimation

Group	Day 2	Day 7	Day 14
Fraction 1	0,00±0,00	0,00±0,00	0,00±0,00
Fraction 2	0,00±0,00	0,00±0,00	0,00±0,00
Fraction 3	0,00±0,00	0,00±0,00	0,00±0,00
Fraction 4	0,00±0,00	0,00±0,00	0,00±0,00
Fraction 5	0,00±0,00	0,00±0,00	0,00±0,00
Fraction 6	0,00±0,00	0,00±0,00	0,00±0,00
Fraction 7	0,00±0,00	0,00±0,00	0,00±0,00
Normal	0,00±0,00	0,00±0,00	0,00±0,00

Group	Day 2	Day 7	Day 14
Positive Control	0,00±0,00	0,20±0,45	0,00±0,00
Negative Control	0,00±0,00	0,00±0,00	0,00±0,00

Data interpretation:
• There was no response.

20. Writhing Response

Group	Day 2	Day 7	Day 14
Fraction 1	0,00±0,00	0,00±0,00	0,00±0,00
Fraction 2	0,00±0,00	0,00±0,00	0,00±0,00
Fraction 3	0,00±0,00	0,00±0,00	0,00±0,00
Fraction 4	0,00±0,00	0,00±0,00	0,00±0,00
Fraction 5	0,00±0,00	0,00±0,00	0,00±0,00
Fraction 6	0,00±0,00	0,00±0,00	0,00±0,00
Fraction 7	0,00±0,00	0,00±0,00	0,00±0,00
Normal	0,00±0,00	0,00±0,00	0,00±0,00
Positive Control	0,00±0,00	0,00±0,00	0,00±0,00
Negative Control	0,00±0,00	0,00±0,00	0,00±0,00

Data interpretation:
• There was no pain response.

21. Rapid Breathing (Number of Breaths/Minute)

Group	Day 2	Day 7	Day 14
Fraction 1	144,6±32,42	153,33±5,77	148±2,34
Fraction 2	148±19,23	149,33±15,04	148,4±5,68
Fraction 3	136±15,16	153±9,75	144,4±13,67
Fraction 4	126,75±22,11	133,33±11,72	147,25±2,98
Fraction 5	143,83±	139,67±0,82	140,2±6,61
Fraction 6	141,33±32,33	151,67±5,77	150,33±8,39
Fraction 7	155±17,32	140±10	157±6,83
Normal	129,5±7,72	142,33±2,52	156,5±7,59
Positive Control	143,2±19,52	138±13,94	148,8±2,86
Negative Control	137,33±20,03	150±10	152,75±5,12

Data interpretation:
• There was no significant difference.

22. Pallor

Group	Day 2	Day 7	Day 14
Fraction 1	0,00±0,00	0,00±0,00	0,00±0,00
Fraction 2	0,00±0,00	0,00±0,00	0,00±0,00
Fraction 3	0,00±0,00	0,00±0,00	0,00±0,00
Fraction 4	0,00±0,00	0,00±0,00	0,00±0,00
Fraction 5	0,00±0,00	0,17±0,41	0,40±0,55
Fraction 6	0,00±0,00	0,50±0,71	0,00±0,00
Fraction 7	0,00±0,00	0,00±0,00	0,00±0,00
Normal	0,00±0,00	0,00±0,00	0,00±0,00
Positive Control	0,00±0,00	0,00±0,00	0,00±0,00
Negative Control	0,00±0,00	0,25±0,50	0,00±0,00

Data interpretation:

• Observed in Fraction 5.

23. Passive Behavior

Group	Day 2	Day 7	Day 14
Fraction 1	0,00±0,00	0,00±0,00	0,20±0,45
Fraction 2	0,00±0,00	0,00±0,00	0,00±0,00
Fraction 3	0,20±0,45	0,20±0,45	0,40±0,55
Fraction 4	0,00±0,00	0,00±0,00	0,25±0,50
Fraction 5	0,00±0,00	0,17±0,41	0,17±0,41
Fraction 6	0,33±0,58	0,00±0,00	0,33±0,58
Fraction 7	0,00±0,00	0,00±0,00	0,25±0,50
Normal	0,00±0,00	0,00±0,00	0,00±0,00
Positive Control	0,00±0,00	0,20±0,45	0,40±0,55
Negative Control	0,00±0,00	0,00±0,00	0,00±0,00

Data interpretation:

• There was no difference.

24. Tremor

Group	Day 2	Day 7	Day 14
Fraction 1	0,00±0,00	0,00±0,00	0,20±0,45
Fraction 2	0,00±0,00	0,00±0,00	0,20±0,45

Group	Day 2	Day 7	Day 14
Fraction 3	0,00±0,00	0,00±0,00	0,00±0,00
Fraction 4	0,00±0,00	0,00±0,00	0,00±0,00
Fraction 5	0,00±0,00	0,00±0,00	0,00±0,00
Fraction 6	0,00±0,00	0,00±0,00	0,00±0,00
Fraction 7	0,00±0,00	0,00±0,00	0,00±0,00
Normal	0,00±0,00	0,00±0,00	0,00±0,00
Positive Control	0,00±0,00	0,00±0,00	0,00±0,00
Negative Control	0,00±0,00	0,00±0,00	0,00±0,00

Data interpretation:

• There was no response.

25. Paralysis

Group	Day 2	Day 7	Day 14
Fraction 1	0,00±0,00	0,25±0,50	0,00±0,00
Fraction 2	0,00±0,00	0,00±0,00	0,20±0,45
Fraction 3	0,00±0,00	0,20±0,45	0,00±0,00
Fraction 4	0,00±0,00	0,00±0,00	0,00±0,00
Fraction 5	0,00±0,00	0,17±0,41	0,00±0,00
Fraction 6	0,00±0,00	0,00±0,00	0,00±0,00
Fraction 7	0,00±0,00	0,00±0,00	0,00±0,00
Normal	0,00±0,00	0,00±0,00	0,00±0,00
Positive Control	0,00±0,00	0,20±0,45	0,00±0,00
Negative Control	0,00±0,00	0,00±0,00	0,00±0,00

Data interpretation:

• There was no response.

26. Tail Pinch Response Time

Group	Day 2 (sec)	Day 7 (sec)	Day 14 (sec)
Fraction 1	8,25±3,86	1,00±0,00	8,25±3,50
Fraction 2	6,60±2,97	4,00±5,20	6,40±4,93
Fraction 3	8,20±2,17	1,00±0,00	8,20±4,02
Fraction 4	9,00±0,82	7,00±1,73	5,50±5,20
Fraction 5	7,83±2,23	7,80±4,09	8,40±4,16
Fraction 6	9,00±1,73	7,00±7,00	8,33±4,72

Group	Day 2 (sec)	Day 7 (sec)	Day 14 (sec)
Fraction 7	7,25±4,57	6,50±7,78	5,50±5,20
Normal	5,75±2,98	2,67±2,08	1,75±0,96
Positive Control	7,60±2,30	4,60±4,93	4,60±4,92
Negative Control	4,75±0,50	5,25±4,92	8,75±5,25

Data interpretation:

• Groups 4 and 7 responded the fastest.

• Groups 1, 3, 5, and 6 were close to normal.

27. Ear Pain Reflex Response Time

Group	Day 2 (sec)	Day 7 (sec)	Day 14 (sec)
Fraction 1	3,80±3,56	3,25±4,50	1,00±0,00
Fraction 2	4,20±3,56	2,00±1,73	4,60±4,93
Fraction 3	2,60±1,51	1,00±0,00	6,40±4,93
Fraction 4	4,25±3,86	2,67±2,08	2,00±2,00
Fraction 5	4,33±3,88	8,17±3,60	3,00±3,94
Fraction 6	5,67±5,51	1,67±1,15	4,00±5,20
Fraction 7	5,50±4,97	1,00±0,00	1,00±0,00
Normal	5,00±4,97	1,00±0,00	1,00±0,00
Positive Control	3,40±3,71	2,80±4,02	3,80±4,76
Negative Control	1,25±0,50	1,00±0,00	2,00±2,00

Data interpretation:

• Fractions 1 and 7 were close to normal.

28. Startle Response Time

Group	Day 2 (sec)	Day 7 (sec)	Day 14 (sec)
Fraction 1	1,00±0,00	2,25±2,50	2,20±1,79
Fraction 2	2,60±1,52	1,00±0,00	1,60±1,34
Fraction 3	1,00±0,00	1,60±1,34	1,00±0,00
Fraction 4	3,75±2,75	2,33±2,31	1,00±0,00
Fraction 5	1,50±0,84	1,17±0,41	2,00±1,73
Fraction 6	1,50±0,71	1,00±0,00	1,00±0,00
Fraction 7	1,50±1,00	2,67±2,89	2,75±2,06
Normal	1,75±1,50	1,00±0,00	2,00± 2,00
Positive Control	3,60±3,97	1,00±0,00	1,20±0,45

Group	Day 2 (sec)	Day 7 (sec)	Day 14 (sec)
Negative Control	7,00±6,93	1,00±0,00	1,00±0,00

Data interpretation:

• Fractions 1, 2, and 5 were close to normal.

29. Urination

Group	Day 2	Day 7	Day 14
Fraction 1	0,20±0,45	0,00±0,00	0,00±0,00
Fraction 2	0,40±0,55	0,33±0,58	0,00±0,00
Fraction 3	0,00±0,00	0,20±0,45	0,20±0,45
Fraction 4	0,00±0,00	0,33±0,58	0,50±0,58
Fraction 5	0,17±0,41	0,17±0,41	0,20±0,45
Fraction 6	0,00±0,00	0,00±0,00	0,00±0,00
Fraction 7	0,00±0,00	0,67±0,58	0,25±0,50
Normal	0,00±0,00	0,00±0,00	0,00±0,00
Positive Control	0,40±0,55	0,20±0,45	0,20±0,45
Negative Control	0,25±0,50	0,00±0,00	0,25±0,50

Data interpretation:

• Fractions 1, 2, and 6 were similar to normal.

30. SGOT Level

Group	Day 14 (mg/dL)
Fraction 1	68,42±28,19
Fraction 2	51,65±23,75
Fraction 3	63,21±50,09
Fraction 4	44,92±21,28
Fraction 5	62,66±19,47
Fraction 6	95,10±74,56
Fraction 7	80,11±45,89
Normal	84,30±30,20
Positive Control	65,22±35,58
Negative Control	52,98±11,89

Data interpretation:

• Groups 1, 3, and 5 were close to the positive control and below the normal group.

• Fraction 6 was above normal.

31. SGPT Level

Group	Day 14 (mg/dL)
Fraction 1	60,45±16,97
Fraction 2	23,05±7,93
Fraction 3	25,23±11,57
Fraction 4	36,31±14,50
Fraction 5	58,93±18,97
Fraction 6	43,25±20,83
Fraction 7	63,36±12,63
Normal	24,77±12,25
Positive Control	34,02±5,59
Negative Control	24,90±15,84

Data interpretation:
• Groups 2 and 3 were close to normal.
• Groups 1, 3, 5, and 7 were far above normal.

32. Liver Organ Index

Group	Mean	SD
NORMAL	4,616	0,233
Positive Control	4,656	0,508
Negative Control	4,782	0,566
Fraction 1	4,259	1,317
2	4,592	0,301
3	4,650	0,640
4	4,741	0,337
5	5,397	0,826
6	3,747	0,350
7	4,245	0,141

Data interpretation:
• Groups 2 and 3 were close to normal.

33. Kidney Organ Index

Group	Mean	SD
NORMAL	4,616	0,233
Positive Control	4,656	0,508
Negative Control	4,782	0,566

Group	Mean	SD
Fraction 1	4,259	1,317
2	4,592	0,301
3	4,592	0,640
4	4,650	0,337
5	4,741	0,826
6	5,397	0,350
7	3,747	0,141

Data interpretation:
• Groups 2, 3, 4, and 5 were close to normal.

34. Spleen Organ Index

Group	Mean	SD
NORMAL	0,996	0,642
Positive Control	1,472	0,256
Negative Control	1,471	0,224
Fraction 1	1,510	0,528
2	1,359	0,166
3	1,183	0,182
4	1,239	0,169
5	1,519	0,170
6	1,417	0,264
7	1,200	0,095

Data interpretation:
• All groups were above normal.

Next: flow cytometry data and histopathological analysis
Video link for 1st JICE, Day 1: https://youtu.be/XFNr_b37Ikc?si=Z4i1EPvwXkLfVhVY
Video link for 1st JICE, Day 2: https://youtu.be/JvVPODOMRV4?si=KDhoEEOoQH-ED-3v
