# Supplementary material for: Kratom (Mitragyna speciosa) as a Phytochemical-Based Natural Product Exhibiting Opioid-like Analgesic Effects with Reduced Tolerance and Dependence Liability via TLR4-Associated Neuroimmune Modulation
Source: Molecules. 2026 Apr 26;31(9):1428. doi: 10.3390/molecules31091428 (PMC13164666; doi:10.3390/molecules31091428)
Supplement: Supplementary file 1 [file molecules-31-01428-s001.zip › Ethical Clearance.pdf]

KOMISI ETIK PENELITIAN KESEHATAN
HEALTH RESEARCH ETHICS COMMISSION
FAKULTAS FARMASI UNIVERSTAS MULAWARMAN
FAKULTAS FARMASI UNIVERSITAS MULAWARMAN

KETERANGAN LAYAK ETIK
DESCRIPTION OF ETHICAL EXEMPTION
"ETHICAL EXEMPTION"

No.179/KEPK-FFUNMUL/EC/EXP/11/2023

Protokol penelitian versi 1 yang diusulkan oleh :
The research protocol proposed by

Peneliti utama : apt. Fajar Prasetya, M.Si., Ph.D
Principal In Investigator

Nama Institusi : Universitas Mulawarman
Name of the Institution

Dengan judul:
Title

"KAJIAN DAN REGIMENTASI DOSIS KRATOM (MITRAGYNA SPECIOSA) DALAM PENGHAMBATAN GLIAL ACTIVATION (TOLL LIKE RECEPTOR-4) SEBAGAI ANALGETIK (NON-OPIAT) TANPA TOLERANSI DAN DEPENDANSI OPIOID PEMBANGKIT KEMANDIRIAN EKONOMI DAN KESEHATAN BERBASIS KEKAYAAN ALAM DAN BUDAYA LOKAL"

"KAJIAN DAN REGIMENTASI DOSIS KRATOM (MITRAGYNA SPECIOSA) DALAM PENGHAMBATAN GLIAL ACTIVATION (TOLL LIKE RECEPTOR-4) SEBAGAI ANALGETIK (NON-OPIAT) TANPA TOLERANSI DAN DEPENDANSI OPIOID PEMBANGKIT KEMANDIRIAN EKONOMI DAN KESEHATAN BERBASIS KEKAYAAN ALAM DAN BUDAYA LOKAL"

Dinyatakan layak etik sesuai 7 (tujuh) Standar WHO 2011, yaitu 1) Nilai Sosial, 2) Nilai Ilmiah, 3) Pemerataan Beban dan Manfaat, 4) Risiko, 5) Bujukan/Eksploitasi, 6) Kerahasiaan dan Privacy, dan 7) Persetujuan Setelah Penjelasan, yang merujuk pada Pedoman CIOMS 2016. Hal ini seperti yang ditunjukkan oleh terpenuhinya indikator setiap standar.

Declared to be ethically appropriate in accordance to 7 (seven) WHO 2011 Standards, 1) Social Values, 2) Scientific Values, 3) Equitable Assessment and Benefits, 4) Risks, 5) Persuasion/Exploitation, 6) Confidentiality and Privacy, and 7) Informed Concent, referring to the 2016 CIOMS Guidelines. This is as indicated by the fulfillment of the indicators of each standard.

Pernyataan Laik Etik ini berlaku selama kurun waktu tanggal 23 November 2023 sampai dengan tanggal 31 Desember 2023.

This declaration of ethics applies during the period November 23, 2023 until December 31, 2023.

November 23, 2023
Professor and Chairperson,

Dr. Apt. Riski Sulistiarini, M.Si

Anggota Peneliti : Dr. apt. Niken Indriyanti, M.Si
