# Supplementary material for: Kratom (Mitragyna speciosa) as a Phytochemical-Based Natural Product Exhibiting Opioid-like Analgesic Effects with Reduced Tolerance and Dependence Liability via TLR4-Associated Neuroimmune Modulation
Source: Molecules. 2026 Apr 26;31(9):1428. doi: 10.3390/molecules31091428 (PMC13164666; doi:10.3390/molecules31091428)
Supplement: Supplementary file 1 [file molecules-31-01428-s001.zip › Kratom_Flow Cytometri Results.pdf]

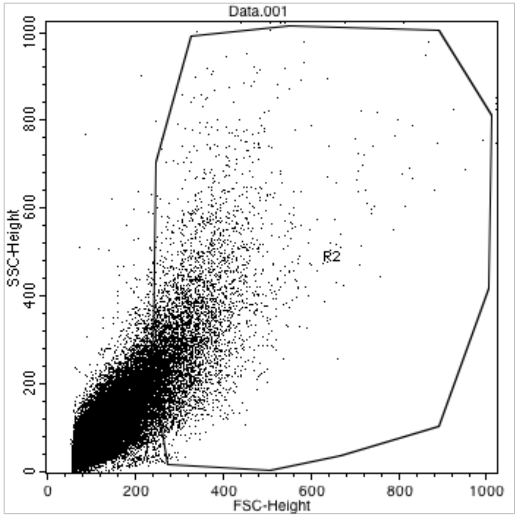

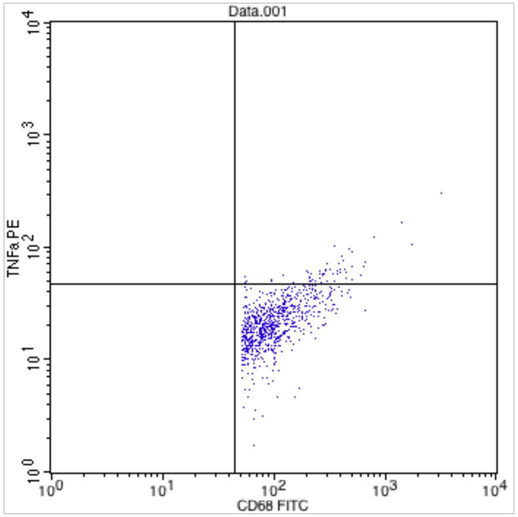

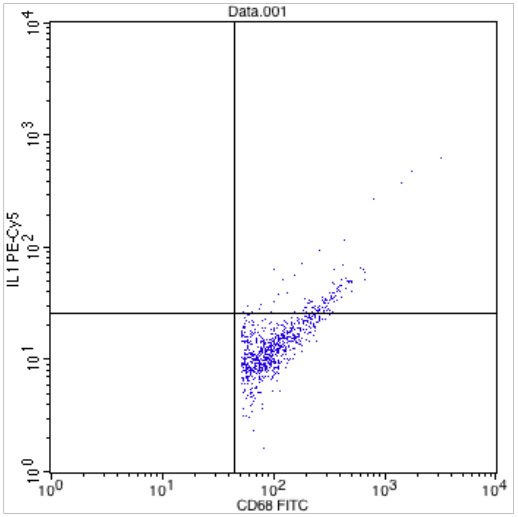

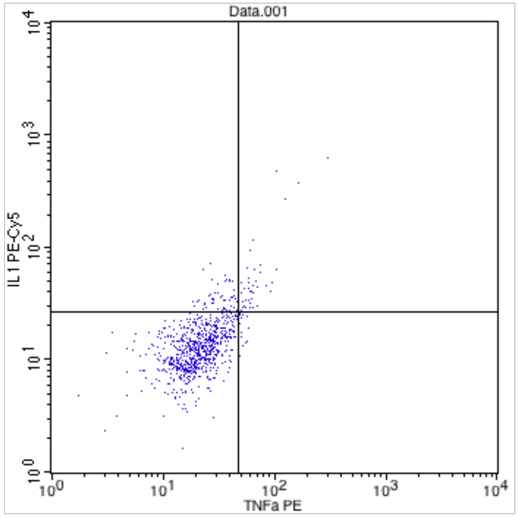

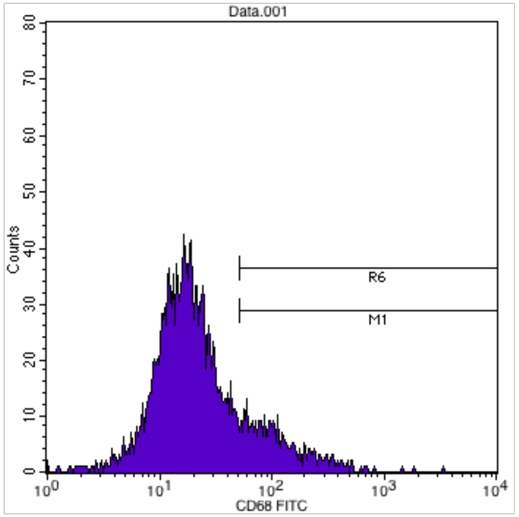

Quadrant Statistics

File: Data.001
Acquisition Date: 16-Oct-24
Gated Events: 833
X Parameter: CD68 FITC (Log)
Quad Location: 45, 47

Sample ID: Norm.1
Gate: G7
Total Events: 71712
Y Parameter: TNFa PE (Log)

Quad	Events	% Gated	% Total
UL	0	0.00	0.00
UR	56	6.72	0.08
LL	0	0.00	0.00
LR	777	93.28	1.08

Quadrant Statistics

File: Data.001
Acquisition Date: 16-Oct-24
Gated Events: 833
X Parameter: CD68 FITC (Log)
Quad Location: 45, 26

Sample ID: Norm.1
Gate: G7
Total Events: 71712
Y Parameter: IL1 PE-Cy5 (Log)

Quad	Events	% Gated	% Total
UL	0	0.00	0.00
UR	102	12.24	0.14
LL	0	0.00	0.00
LR	731	87.76	1.02

Quadrant Statistics

File: Data.001
Acquisition Date: 16-Oct-24
Gated Events: 833
X Parameter: TNFa PE (Log)
Quad Location: 47, 26

Sample ID: Norm.1
Gate: G7
Total Events: 71712
Y Parameter: IL1 PE-Cy5 (Log)

Quad	Events	% Gated	% Total
UL	58	6.96	0.08
UR	40	4.80	0.06
LL	721	86.55	1.01
LR	14	1.68	0.02

Histogram Statistics

File: Data.001
Acquisition Date: 16-Oct-24
Gated Events: 5249
X Parameter: CD68 FITC (Log)

Sample ID: Norm.1
Gate: G2
Total Events: 71712

Marker	Left, Right	Events	% Gated	% Total
All	1, 9910	5249	100.00	7.32
M1	51, 9910	833	15.87	1.16

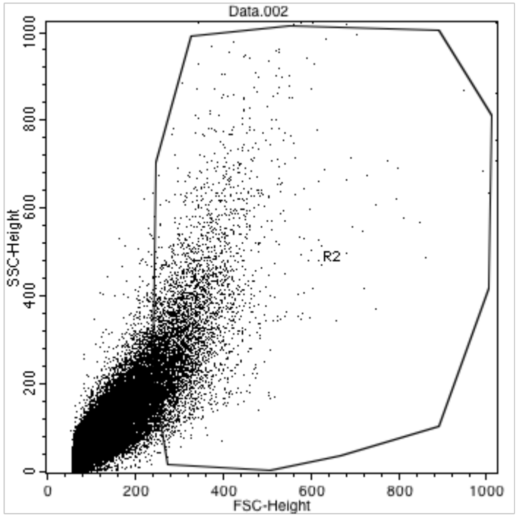

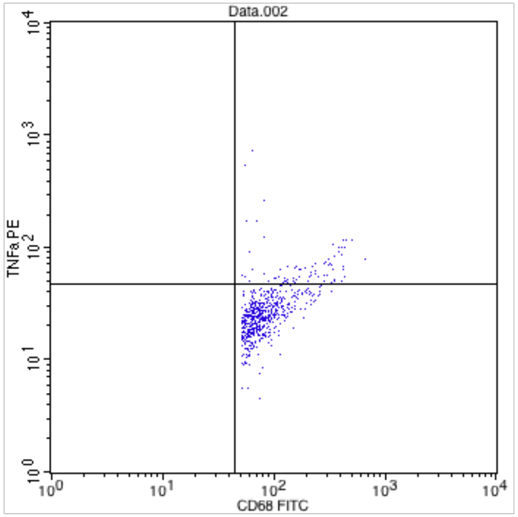

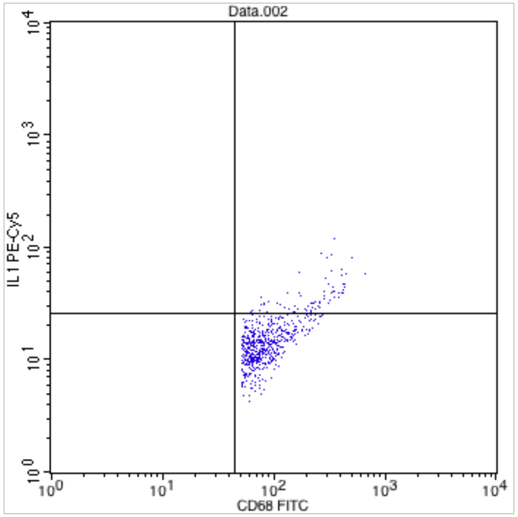

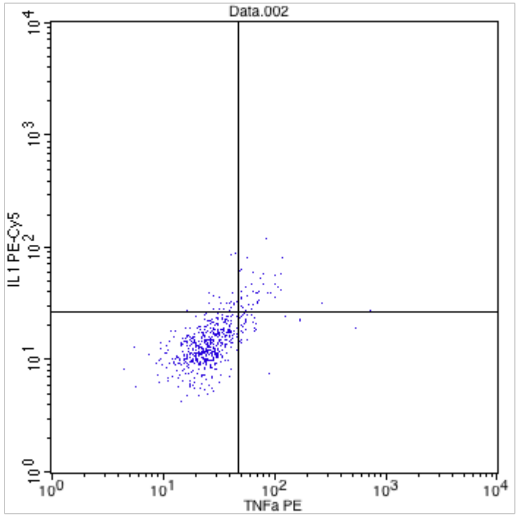

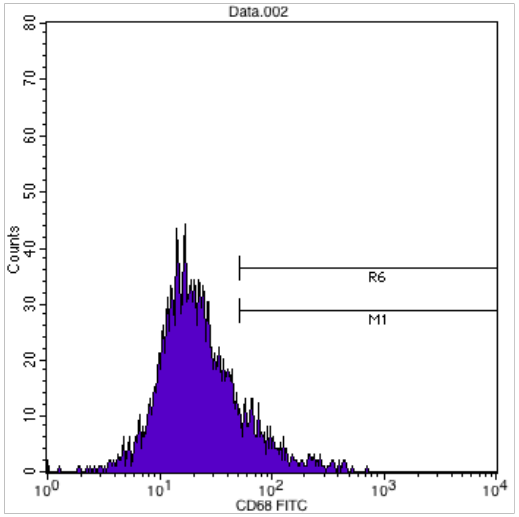

Quadrant Statistics

File: Data.002
Acquisition Date: 16-Oct-24
Gated Events: 606
X Parameter: CD68 FITC (Log)
Quad Location: 45, 47
Sample ID: +.1
Gate: G7
Total Events: 74199
Y Parameter: TNFa PE (Log)

Quad	Events	% Gated	% Total
UL	0	0.00	0.00
UR	75	12.38	0.10
LL	0	0.00	0.00
LR	531	87.62	0.72

Quadrant Statistics

File: Data.002
Acquisition Date: 16-Oct-24
Gated Events: 606
X Parameter: CD68 FITC (Log)
Quad Location: 45, 26
Sample ID: +.1
Gate: G7
Total Events: 74199
Y Parameter: IL1 PE-Cy5 (Log)

Quad	Events	% Gated	% Total
UL	0	0.00	0.00
UR	66	10.89	0.09
LL	0	0.00	0.00
LR	540	89.11	0.73

Quadrant Statistics

File: Data.002
Acquisition Date: 16-Oct-24
Gated Events: 606
X Parameter: TNFa PE (Log)
Quad Location: 47, 26
Sample ID: +.1
Gate: G7
Total Events: 74199
Y Parameter: IL1 PE-Cy5 (Log)

Quad	Events	% Gated	% Total
UL	25	4.13	0.03
UR	39	6.44	0.05
LL	509	83.99	0.69
LR	33	5.45	0.04

Histogram Statistics

File: Data.002
Acquisition Date: 16-Oct-24
Gated Events: 5054
X Parameter: CD68 FITC (Log)
Sample ID: +.1
Gate: G2
Total Events: 74199

Marker	Left, Right	Events	% Gated	% Total
All	1, 9910	5054	100.00	6.81
M1	51, 9910	606	11.99	0.82

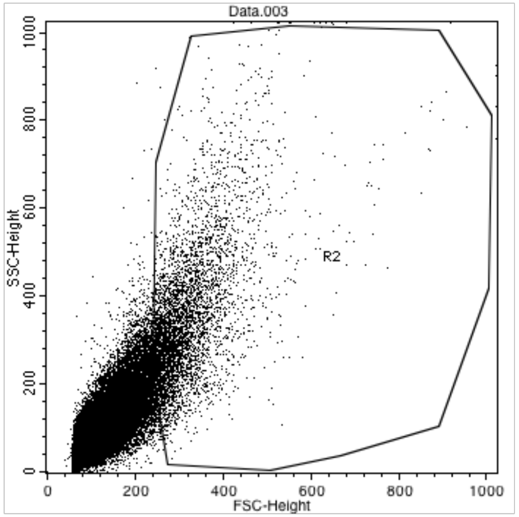

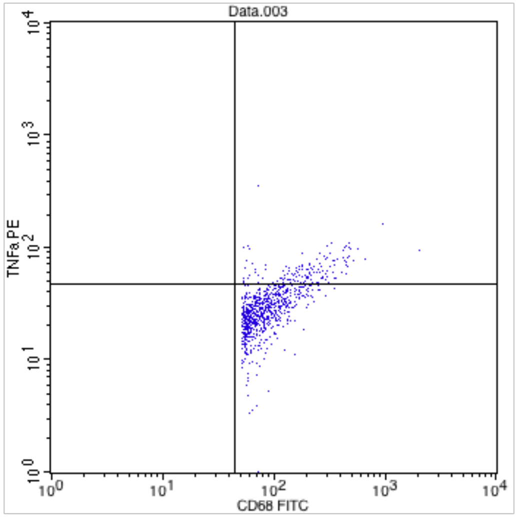

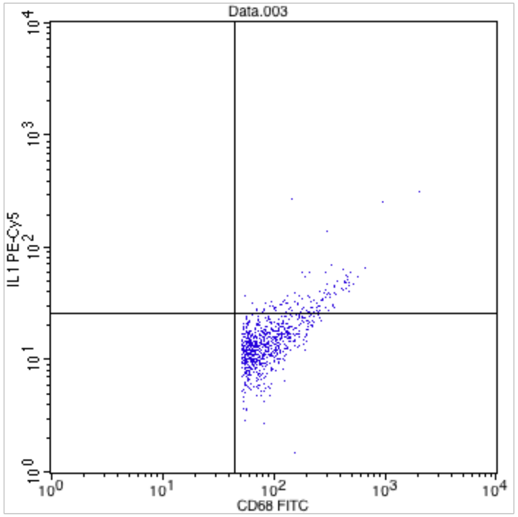

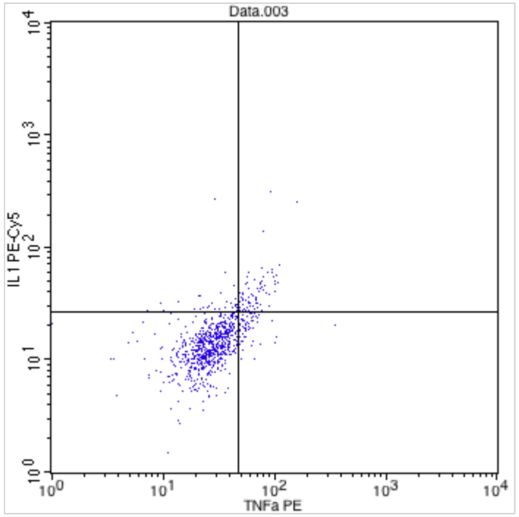

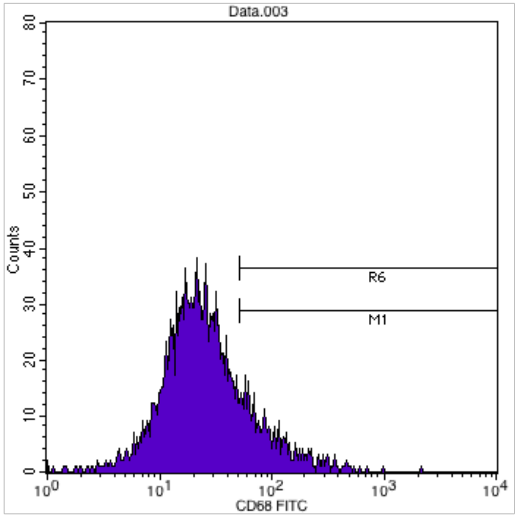

Quadrant Statistics

File: Data.003 Sample ID: +.2
Acquisition Date: 16-Oct-24 Gate: G7
Gated Events: 848 Total Events: 69850
X Parameter: CD68 FITC (Log) Y Parameter: TNFa PE (Log)
Quad Location: 45, 47

Quad	Events	% Gated	% Total
UL	0	0.00	0.00
UR	133	15.68	0.19
LL	0	0.00	0.00
LR	715	84.32	1.02

Quadrant Statistics

File: Data.003 Sample ID: +.2
Acquisition Date: 16-Oct-24 Gate: G7
Gated Events: 848 Total Events: 69850
X Parameter: CD68 FITC (Log) Y Parameter: IL1 PE-Cy5 (Log)
Quad Location: 45, 26

Quad	Events	% Gated	% Total
UL	0	0.00	0.00
UR	111	13.09	0.16
LL	0	0.00	0.00
LR	737	86.91	1.06

Quadrant Statistics

File: Data.003 Sample ID: +.2
Acquisition Date: 16-Oct-24 Gate: G7
Gated Events: 848 Total Events: 69850
X Parameter: TNFa PE (Log) Y Parameter: IL1 PE-Cy5 (Log)
Quad Location: 47, 26

Quad	Events	% Gated	% Total
UL	35	4.13	0.05
UR	71	8.37	0.10
LL	682	80.42	0.98
LR	60	7.08	0.09

Histogram Statistics

File: Data.003 Sample ID: +.2
Acquisition Date: 16-Oct-24 Gate: G2
Gated Events: 5013 Total Events: 69850
X Parameter: CD68 FITC (Log)

Marker	Left, Right	Events	% Gated	% Total
All	1, 9910	5013	100.00	7.18
M1	51, 9910	848	16.92	1.21

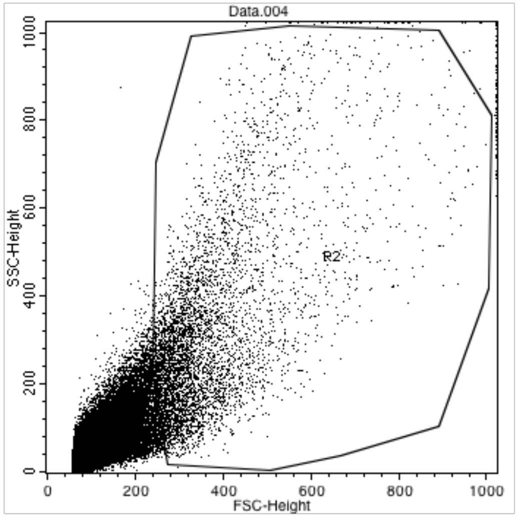

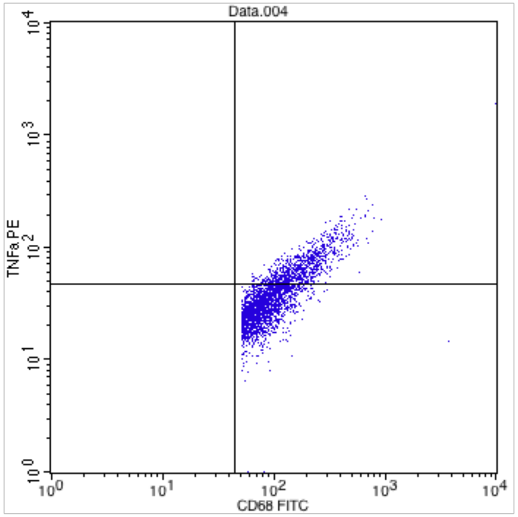

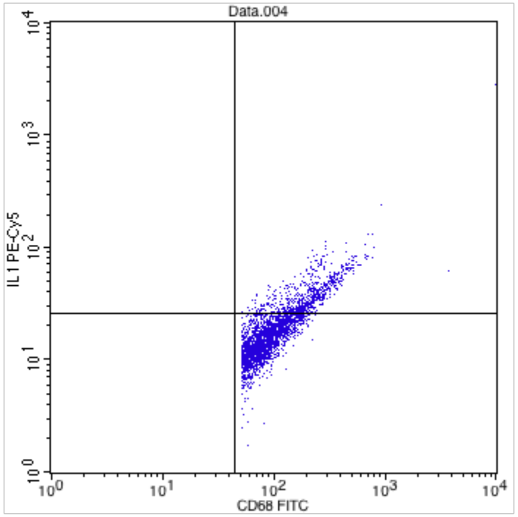

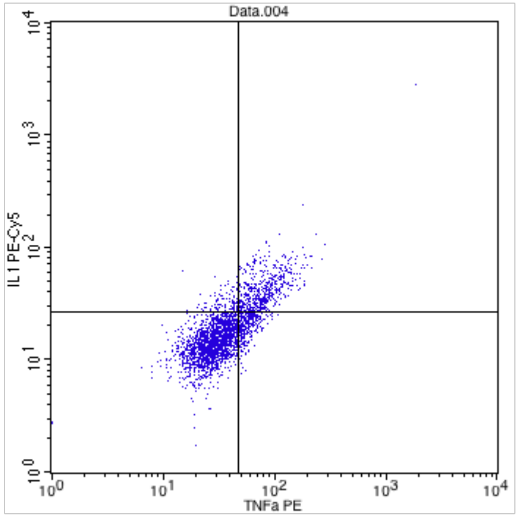

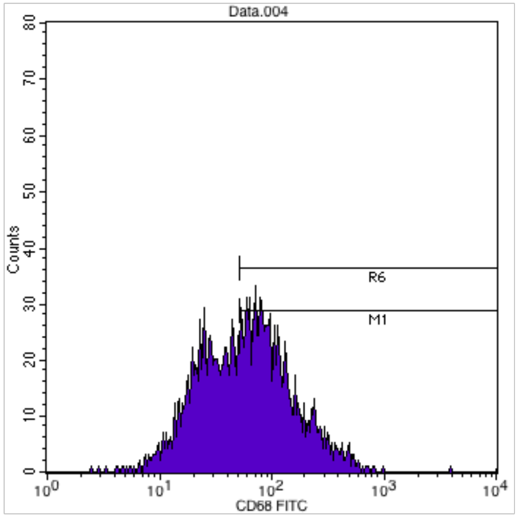

Quadrant Statistics

File: Data.004
Acquisition Date: 16-Oct-24
Gated Events: 2944
X Parameter: CD68 FITC (Log)
Quad Location: 45, 47

Sample ID: -.1
Gate: G7
Total Events: 51989
Y Parameter: TNFa PE (Log)

Quad	Events	% Gated	% Total
UL	0	0.00	0.00
UR	911	30.94	1.75
LL	0	0.00	0.00
LR	2033	69.06	3.91

Quadrant Statistics

File: Data.004
Acquisition Date: 16-Oct-24
Gated Events: 2944
X Parameter: CD68 FITC (Log)
Quad Location: 45, 26

Sample ID: -.1
Gate: G7
Total Events: 51989
Y Parameter: IL1 PE-Cy5 (Log)

Quad	Events	% Gated	% Total
UL	0	0.00	0.00
UR	715	24.29	1.38
LL	0	0.00	0.00
LR	2229	75.71	4.29

Quadrant Statistics

File: Data.004
Acquisition Date: 16-Oct-24
Gated Events: 2944
X Parameter: TNFa PE (Log)
Quad Location: 47, 26

Sample ID: -.1
Gate: G7
Total Events: 51989
Y Parameter: IL1 PE-Cy5 (Log)

Quad	Events	% Gated	% Total
UL	130	4.42	0.25
UR	548	18.61	1.05
LL	1916	65.08	3.69
LR	350	11.89	0.67

Histogram Statistics

File: Data.004
Acquisition Date: 16-Oct-24
Gated Events: 5425
X Parameter: CD68 FITC (Log)

Sample ID: -.1
Gate: G2
Total Events: 51989

Marker	Left, Right	Events	% Gated	% Total
All	1, 9910	5425	100.00	10.43
M1	51, 9910	2944	54.27	5.66

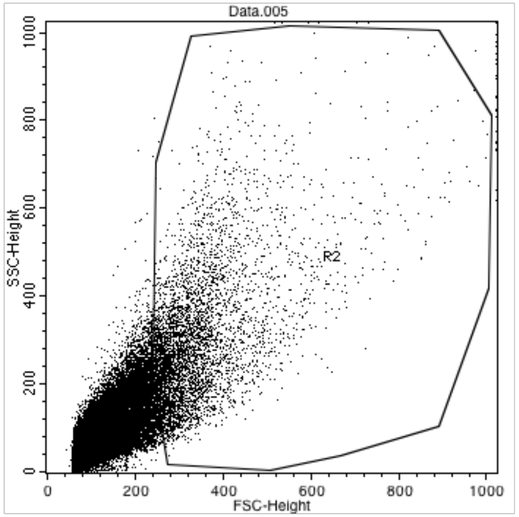

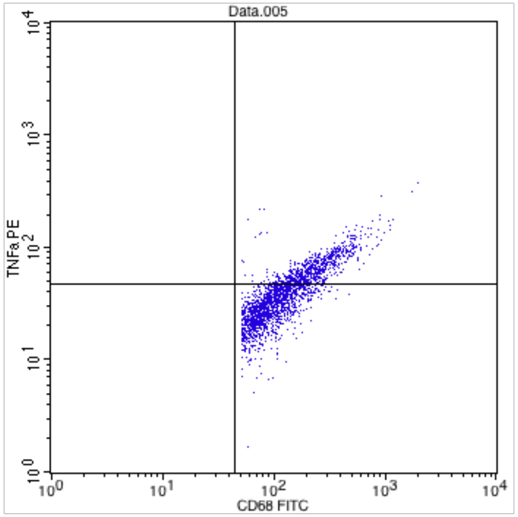

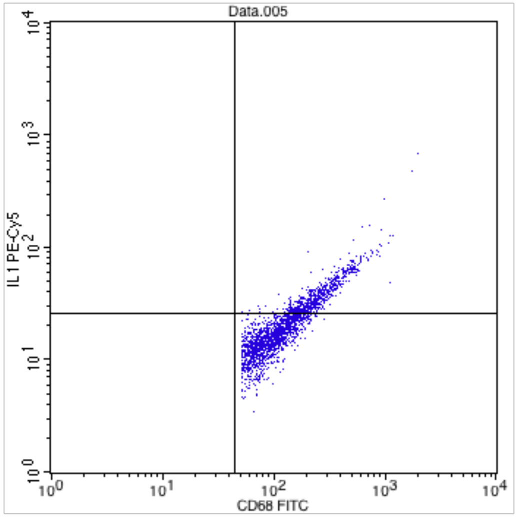

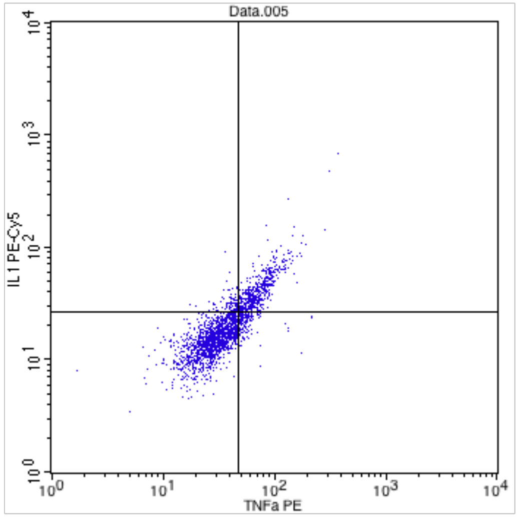

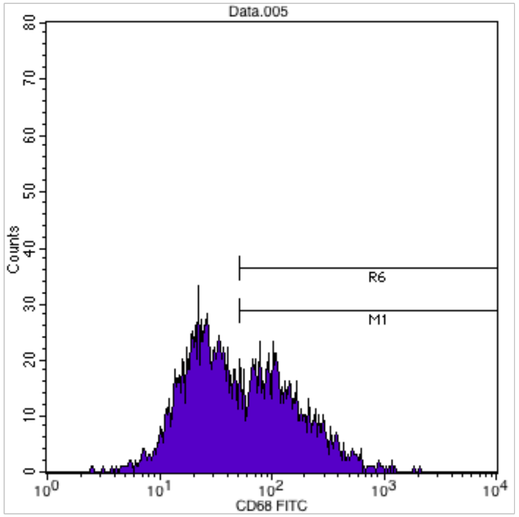

Quadrant Statistics

File: Data.005
Acquisition Date: 16-Oct-24
Gated Events: 2320
X Parameter: CD68 FITC (Log)
Quad Location: 45, 47
Sample ID: -.2
Gate: G7
Total Events: 67195
Y Parameter: TNFa PE (Log)

Quad	Events	% Gated	% Total
UL	0	0.00	0.00
UR	707	30.47	1.05
LL	0	0.00	0.00
LR	1613	69.53	2.40

Quadrant Statistics

File: Data.005
Acquisition Date: 16-Oct-24
Gated Events: 2320
X Parameter: CD68 FITC (Log)
Quad Location: 45, 26
Sample ID: -.2
Gate: G7
Total Events: 67195
Y Parameter: IL1 PE-Cy5 (Log)

Quad	Events	% Gated	% Total
UL	0	0.00	0.00
UR	661	28.49	0.98
LL	0	0.00	0.00
LR	1659	71.51	2.47

Quadrant Statistics

File: Data.005
Acquisition Date: 16-Oct-24
Gated Events: 2320
X Parameter: TNFa PE (Log)
Quad Location: 47, 26
Sample ID: -.2
Gate: G7
Total Events: 67195
Y Parameter: IL1 PE-Cy5 (Log)

Quad	Events	% Gated	% Total
UL	98	4.22	0.15
UR	528	22.76	0.79
LL	1520	65.52	2.26
LR	174	7.50	0.26

Histogram Statistics

File: Data.005
Acquisition Date: 16-Oct-24
Gated Events: 5165
X Parameter: CD68 FITC (Log)
Sample ID: -.2
Gate: G2
Total Events: 67195

Marker	Left, Right	Events	% Gated	% Total
All	1, 9910	5165	100.00	7.69
M1	51, 9910	2320	44.92	3.45

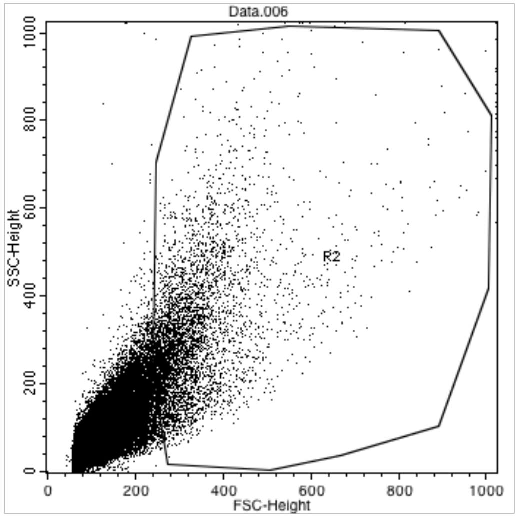

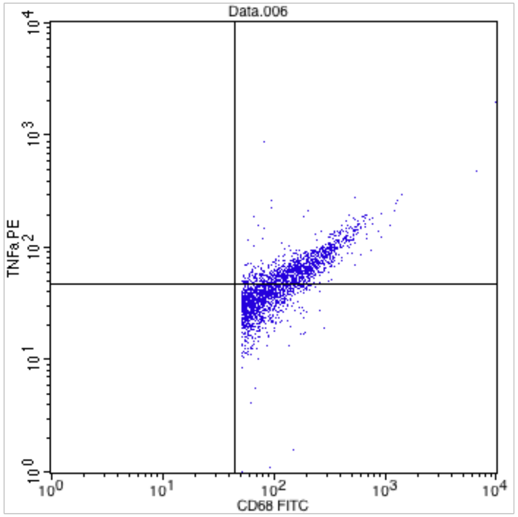

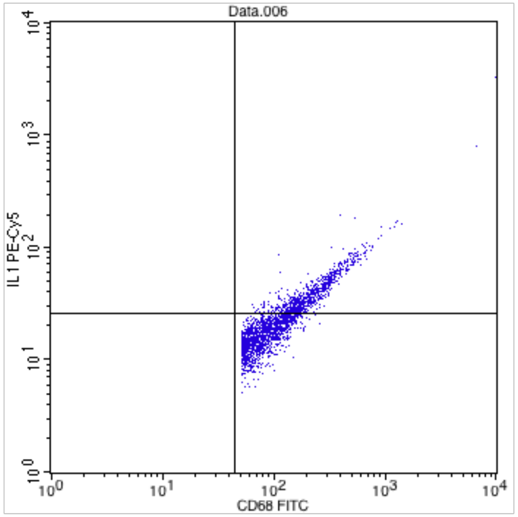

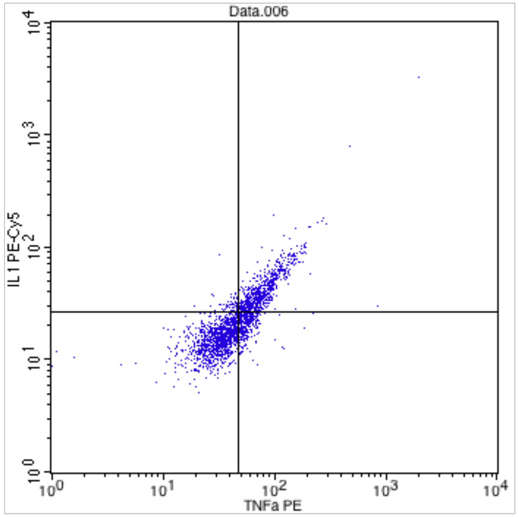

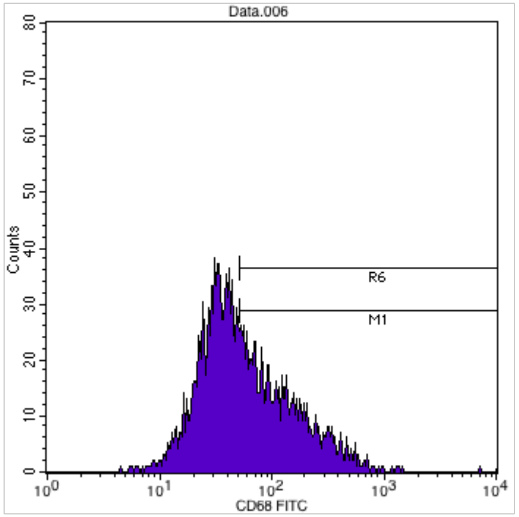

Quadrant Statistics

File: Data.006
Acquisition Date: 16-Oct-24
Gated Events: 2331
X Parameter: CD68 FITC (Log)
Quad Location: 45, 47

Sample ID: -.3
Gate: G7
Total Events: 61523
Y Parameter: TNFa PE (Log)

Quad	Events	% Gated	% Total
UL	0	0.00	0.00
UR	1057	45.35	1.72
LL	0	0.00	0.00
LR	1274	54.65	2.07

Quadrant Statistics

File: Data.006
Acquisition Date: 16-Oct-24
Gated Events: 2331
X Parameter: CD68 FITC (Log)
Quad Location: 45, 26

Sample ID: -.3
Gate: G7
Total Events: 61523
Y Parameter: IL1 PE-Cy5 (Log)

Quad	Events	% Gated	% Total
UL	0	0.00	0.00
UR	823	35.31	1.34
LL	0	0.00	0.00
LR	1508	64.69	2.45

Quadrant Statistics

File: Data.006
Acquisition Date: 16-Oct-24
Gated Events: 2331
X Parameter: TNFa PE (Log)
Quad Location: 47, 26

Sample ID: -.3
Gate: G7
Total Events: 61523
Y Parameter: IL1 PE-Cy5 (Log)

Quad	Events	% Gated	% Total
UL	74	3.17	0.12
UR	708	30.37	1.15
LL	1224	52.51	1.99
LR	325	13.94	0.53

Histogram Statistics

File: Data.006
Acquisition Date: 16-Oct-24
Gated Events: 5254
X Parameter: CD68 FITC (Log)

Sample ID: -.3
Gate: G2
Total Events: 61523

Marker	Left, Right	Events	% Gated	% Total
All	1, 9910	5254	100.00	8.54
M1	51, 9910	2331	44.37	3.79

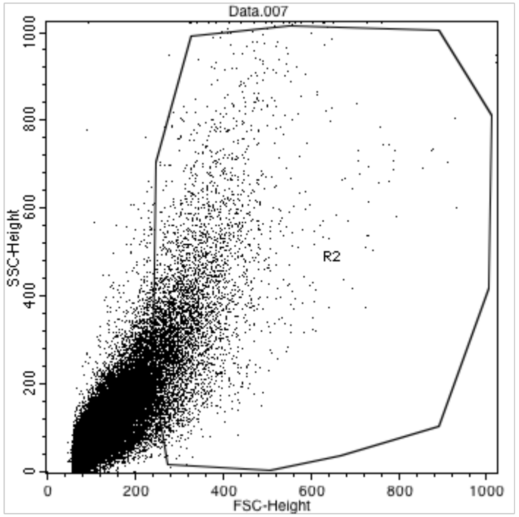

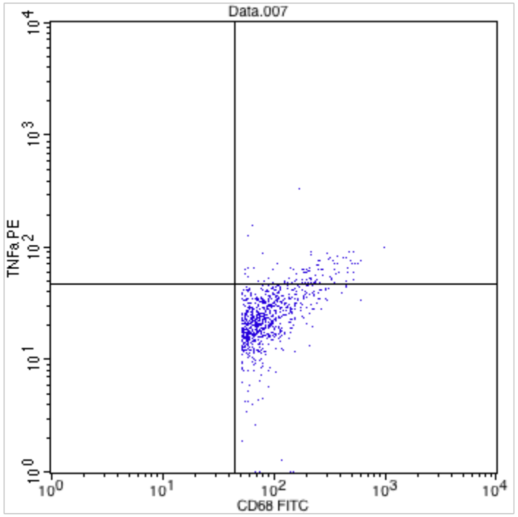

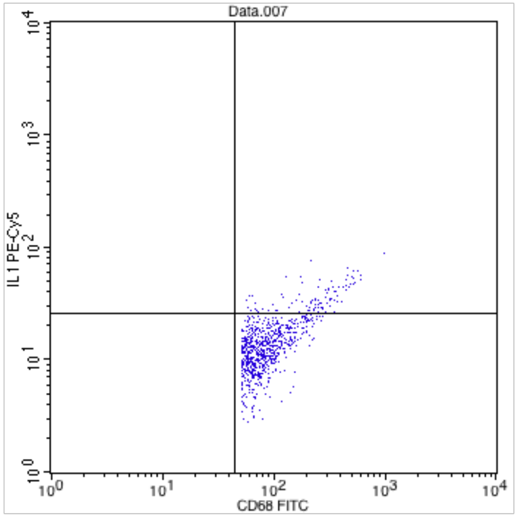

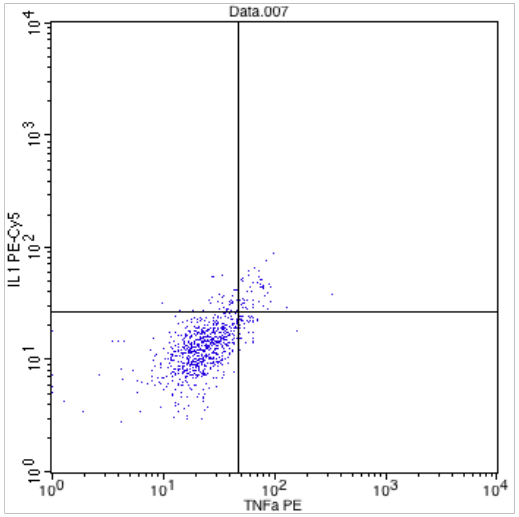

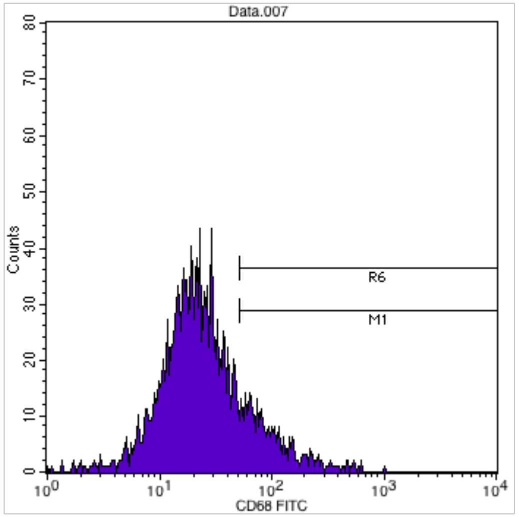

Quadrant Statistics

File: Data.007
Acquisition Date: 16-Oct-24
Gated Events: 835
X Parameter: CD68 FITC (Log)
Quad Location: 45, 47
Sample ID: 1.2
Gate: G7
Total Events: 63513
Y Parameter: TNFa PE (Log)

Quad	Events	% Gated	% Total
UL	0	0.00	0.00
UR	95	11.38	0.15
LL	0	0.00	0.00
LR	740	88.62	1.17

Quadrant Statistics

File: Data.007
Acquisition Date: 16-Oct-24
Gated Events: 835
X Parameter: CD68 FITC (Log)
Quad Location: 45, 26
Sample ID: 1.2
Gate: G7
Total Events: 63513
Y Parameter: IL1 PE-Cy5 (Log)

Quad	Events	% Gated	% Total
UL	0	0.00	0.00
UR	93	11.14	0.15
LL	0	0.00	0.00
LR	742	88.86	1.17

Quadrant Statistics

File: Data.007
Acquisition Date: 16-Oct-24
Gated Events: 835
X Parameter: TNFa PE (Log)
Quad Location: 47, 26
Sample ID: 1.2
Gate: G7
Total Events: 63513
Y Parameter: IL1 PE-Cy5 (Log)

Quad	Events	% Gated	% Total
UL	38	4.55	0.06
UR	50	5.99	0.08
LL	706	84.55	1.11
LR	41	4.91	0.06

Histogram Statistics

File: Data.007
Acquisition Date: 16-Oct-24
Gated Events: 5100
X Parameter: CD68 FITC (Log)
Sample ID: 1.2
Gate: G2
Total Events: 63513

Marker	Left, Right	Events	% Gated	% Total
All	1, 9910	5100	100.00	8.03
M1	51, 9910	835	16.37	1.31

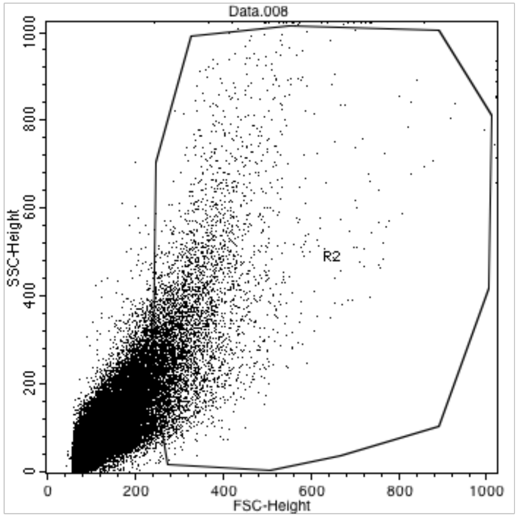

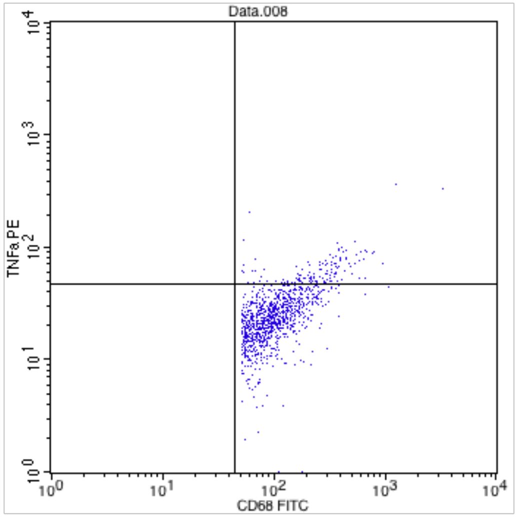

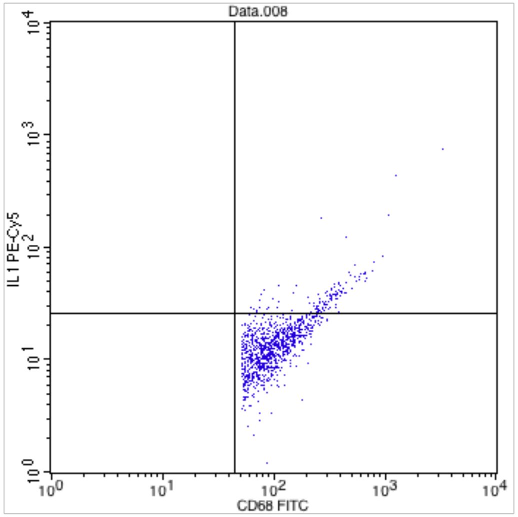

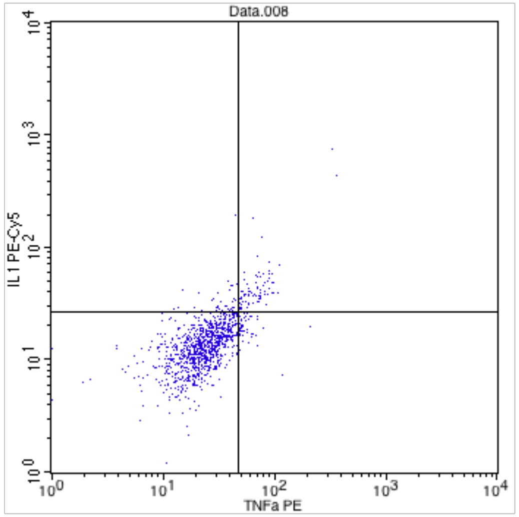

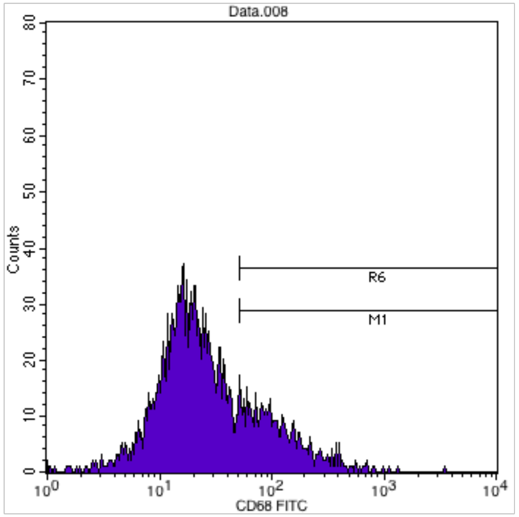

Quadrant Statistics

File: Data.008
Acquisition Date: 16-Oct-24
Gated Events: 1118
X Parameter: CD68 FITC (Log)
Quad Location: 45, 47
Sample ID: 1.3
Gate: G7
Total Events: 71794
Y Parameter: TNFa PE (Log)

Quad	Events	% Gated	% Total
UL	0	0.00	0.00
UR	131	11.72	0.18
LL	0	0.00	0.00
LR	987	88.28	1.37

Quadrant Statistics

File: Data.008
Acquisition Date: 16-Oct-24
Gated Events: 1118
X Parameter: CD68 FITC (Log)
Quad Location: 45, 26
Sample ID: 1.3
Gate: G7
Total Events: 71794
Y Parameter: IL1 PE-Cy5 (Log)

Quad	Events	% Gated	% Total
UL	0	0.00	0.00
UR	119	10.64	0.17
LL	0	0.00	0.00
LR	999	89.36	1.39

Quadrant Statistics

File: Data.008
Acquisition Date: 16-Oct-24
Gated Events: 1118
X Parameter: TNFa PE (Log)
Quad Location: 47, 26
Sample ID: 1.3
Gate: G7
Total Events: 71794
Y Parameter: IL1 PE-Cy5 (Log)

Quad	Events	% Gated	% Total
UL	39	3.49	0.05
UR	76	6.80	0.11
LL	953	85.24	1.33
LR	50	4.47	0.07

Histogram Statistics

File: Data.008
Acquisition Date: 16-Oct-24
Gated Events: 5073
X Parameter: CD68 FITC (Log)
Sample ID: 1.3
Gate: G2
Total Events: 71794

Marker	Left, Right	Events	% Gated	% Total
All	1, 9910	5073	100.00	7.07
M1	51, 9910	1118	22.04	1.56

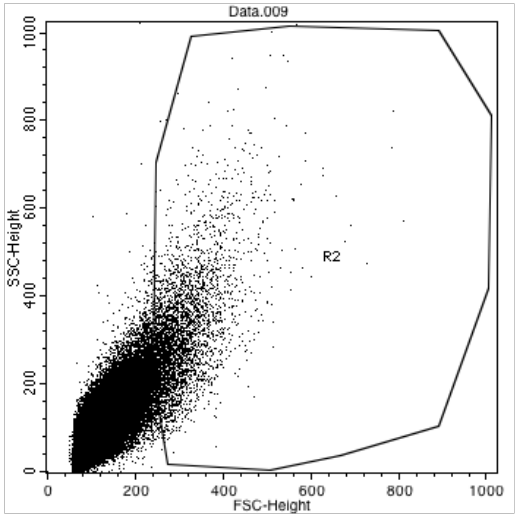

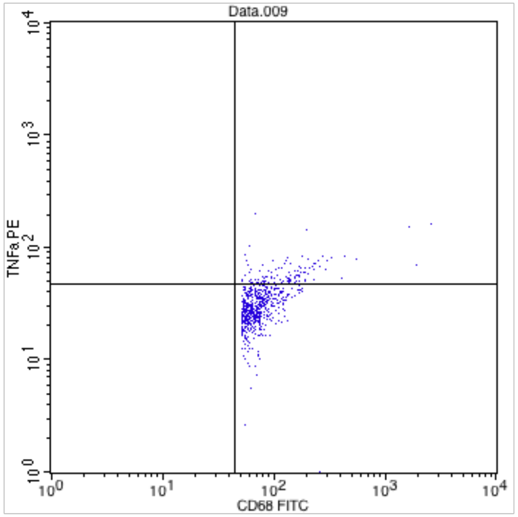

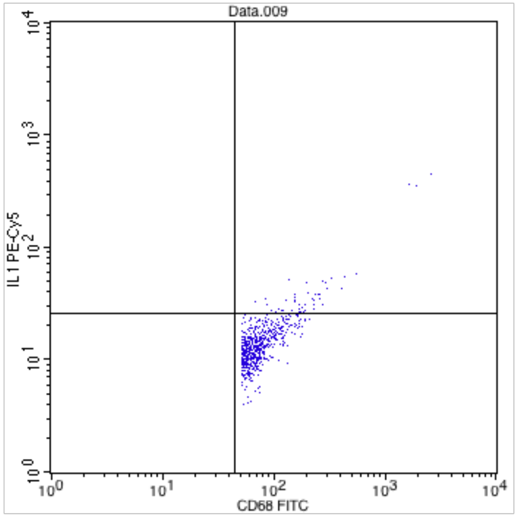

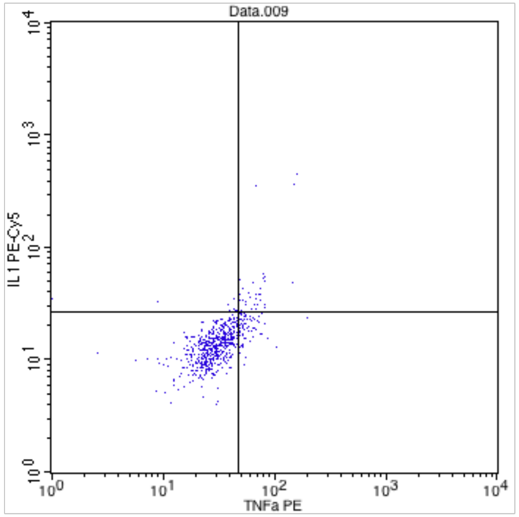

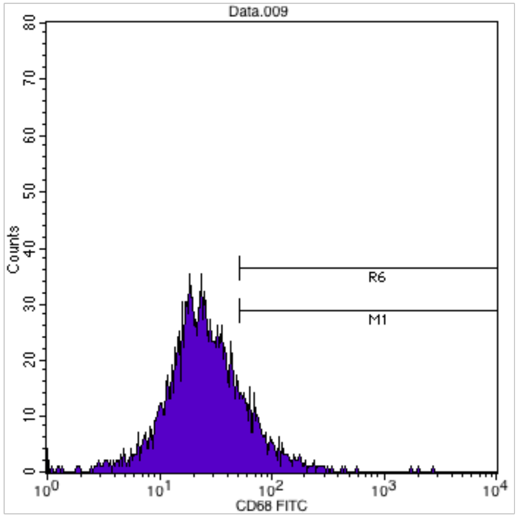

Quadrant Statistics

File: Data.009
Acquisition Date: 16-Oct-24
Gated Events: 643
X Parameter: CD68 FITC (Log)
Quad Location: 45, 47

Sample ID: 2.1
Gate: G7
Total Events: 107803
Y Parameter: TNFa PE (Log)

Quad	Events	% Gated	% Total
UL	0	0.00	0.00
UR	108	16.80	0.10
LL	0	0.00	0.00
LR	535	83.20	0.50

Quadrant Statistics

File: Data.009
Acquisition Date: 16-Oct-24
Gated Events: 643
X Parameter: CD68 FITC (Log)
Quad Location: 45, 26

Sample ID: 2.1
Gate: G7
Total Events: 107803
Y Parameter: IL1 PE-Cy5 (Log)

Quad	Events	% Gated	% Total
UL	0	0.00	0.00
UR	46	7.15	0.04
LL	0	0.00	0.00
LR	597	92.85	0.55

Quadrant Statistics

File: Data.009
Acquisition Date: 16-Oct-24
Gated Events: 643
X Parameter: TNFa PE (Log)
Quad Location: 47, 26

Sample ID: 2.1
Gate: G7
Total Events: 107803
Y Parameter: IL1 PE-Cy5 (Log)

Quad	Events	% Gated	% Total
UL	9	1.40	0.01
UR	34	5.29	0.03
LL	529	82.27	0.49
LR	71	11.04	0.07

Histogram Statistics

File: Data.009
Acquisition Date: 16-Oct-24
Gated Events: 4253
X Parameter: CD68 FITC (Log)

Sample ID: 2.1
Gate: G2
Total Events: 107803

Marker	Left, Right	Events	% Gated	% Total
All	1, 9910	4253	100.00	3.95
M1	51, 9910	643	15.12	0.60

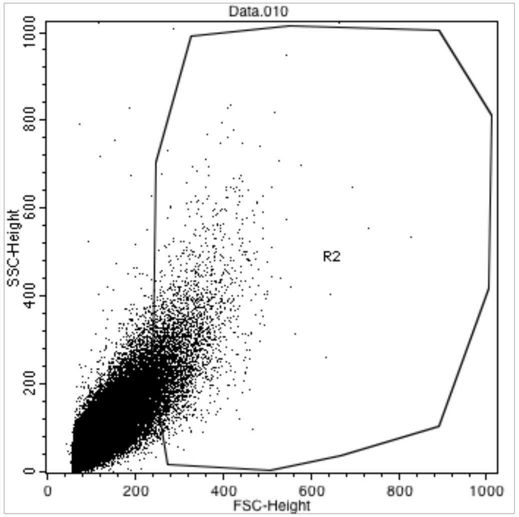

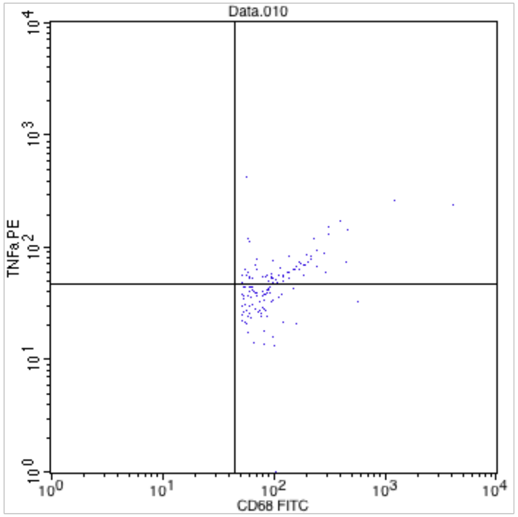

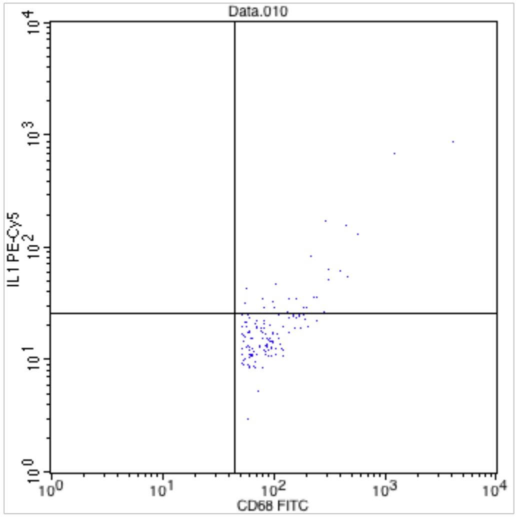

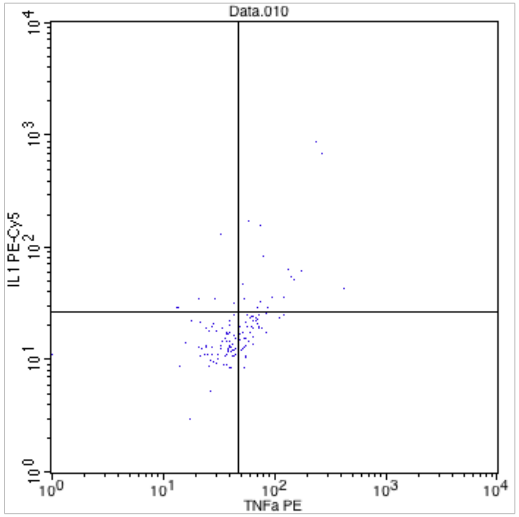

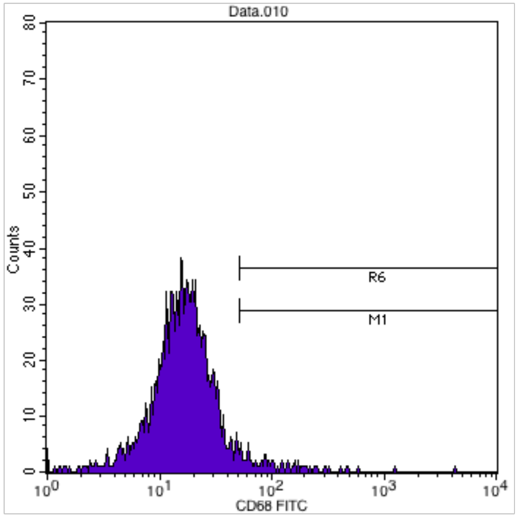

Quadrant Statistics

File: Data.010
Acquisition Date: 16-Oct-24
Gated Events: 132
X Parameter: CD68 FITC (Log)
Quad Location: 45, 47

Sample ID: 2.2
Gate: G7
Total Events: 120343
Y Parameter: TNFa PE (Log)

Quad	Events	% Gated	% Total
UL	0	0.00	0.00
UR	62	46.97	0.05
LL	0	0.00	0.00
LR	70	53.03	0.06

Quadrant Statistics

File: Data.010
Acquisition Date: 16-Oct-24
Gated Events: 132
X Parameter: CD68 FITC (Log)
Quad Location: 45, 26

Sample ID: 2.2
Gate: G7
Total Events: 120343
Y Parameter: IL1 PE-Cy5 (Log)

Quad	Events	% Gated	% Total
UL	0	0.00	0.00
UR	25	18.94	0.02
LL	0	0.00	0.00
LR	107	81.06	0.09

Quadrant Statistics

File: Data.010
Acquisition Date: 16-Oct-24
Gated Events: 132
X Parameter: TNFa PE (Log)
Quad Location: 47, 26

Sample ID: 2.2
Gate: G7
Total Events: 120343
Y Parameter: IL1 PE-Cy5 (Log)

Quad	Events	% Gated	% Total
UL	6	4.55	0.00
UR	18	13.64	0.01
LL	64	48.48	0.05
LR	44	33.33	0.04

Histogram Statistics

File: Data.010
Acquisition Date: 16-Oct-24
Gated Events: 3761
X Parameter: CD68 FITC (Log)

Sample ID: 2.2
Gate: G2
Total Events: 120343

Marker	Left, Right	Events	% Gated	% Total
All	1, 9910	3761	100.00	3.13
M1	51, 9910	132	3.51	0.11

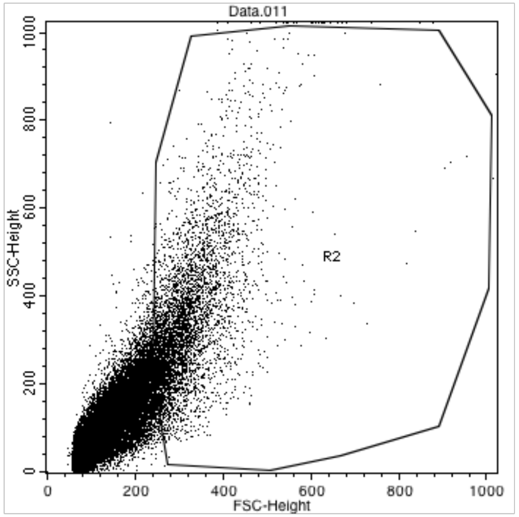

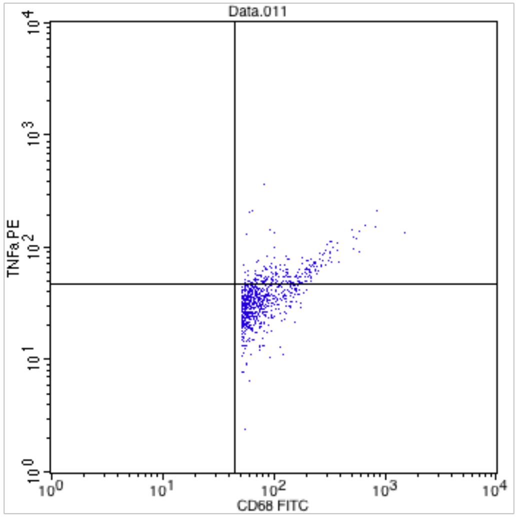

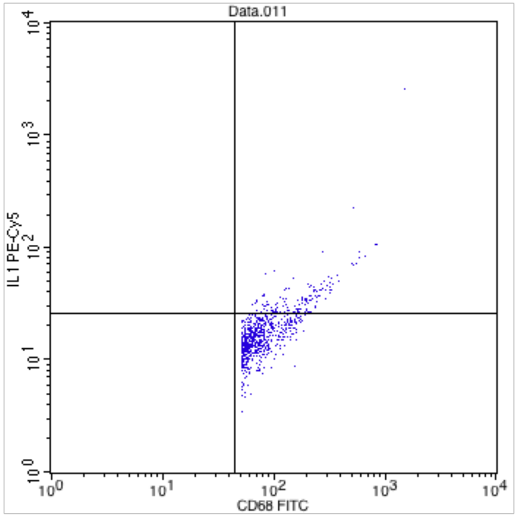

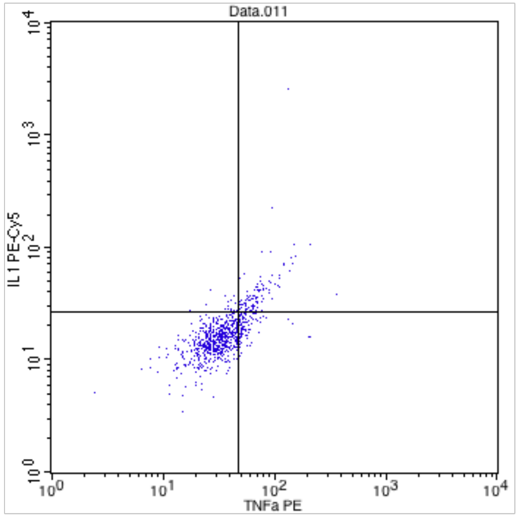

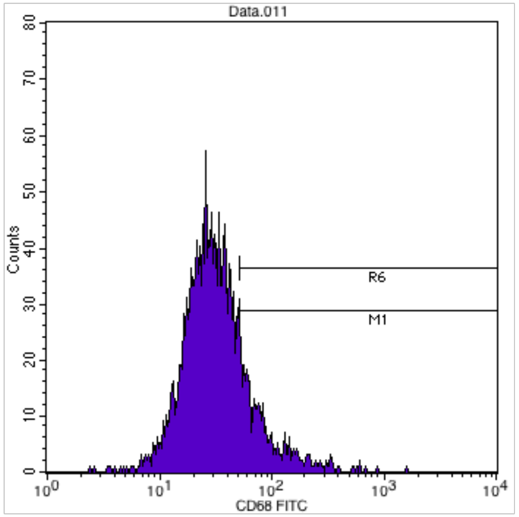

Quadrant Statistics

File: Data.011
Acquisition Date: 16-Oct-24
Gated Events: 848
X Parameter: CD68 FITC (Log)
Quad Location: 45, 47

Sample ID: 3.1
Gate: G7
Total Events: 61985
Y Parameter: TNFa PE (Log)

Quad	Events	% Gated	% Total
UL	0	0.00	0.00
UR	207	24.41	0.33
LL	0	0.00	0.00
LR	641	75.59	1.03

Quadrant Statistics

File: Data.011
Acquisition Date: 16-Oct-24
Gated Events: 848
X Parameter: CD68 FITC (Log)
Quad Location: 45, 26

Sample ID: 3.1
Gate: G7
Total Events: 61985
Y Parameter: IL1 PE-Cy5 (Log)

Quad	Events	% Gated	% Total
UL	0	0.00	0.00
UR	133	15.68	0.21
LL	0	0.00	0.00
LR	715	84.32	1.15

Quadrant Statistics

File: Data.011
Acquisition Date: 16-Oct-24
Gated Events: 848
X Parameter: TNFa PE (Log)
Quad Location: 47, 26

Sample ID: 3.1
Gate: G7
Total Events: 61985
Y Parameter: IL1 PE-Cy5 (Log)

Quad	Events	% Gated	% Total
UL	18	2.12	0.03
UR	106	12.50	0.17
LL	628	74.06	1.01
LR	96	11.32	0.15

Histogram Statistics

File: Data.011
Acquisition Date: 16-Oct-24
Gated Events: 5311
X Parameter: CD68 FITC (Log)

Sample ID: 3.1
Gate: G2
Total Events: 61985

Marker	Left, Right	Events	% Gated	% Total
All	1, 9910	5311	100.00	8.57
M1	51, 9910	848	15.97	1.37

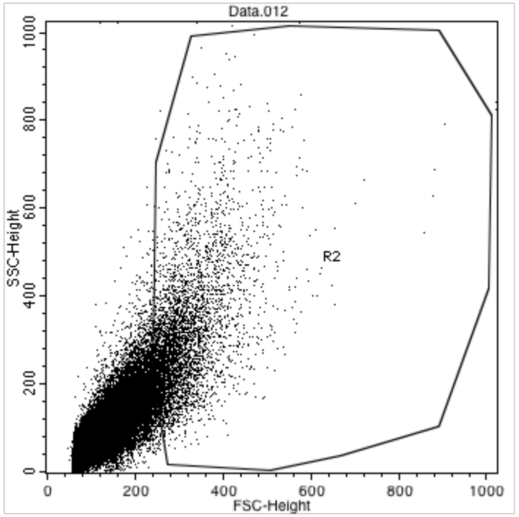

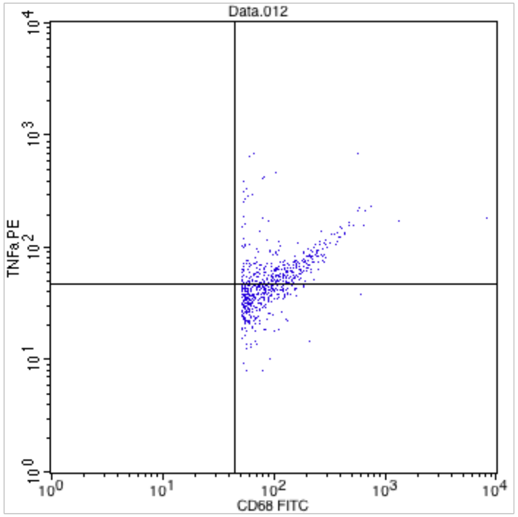

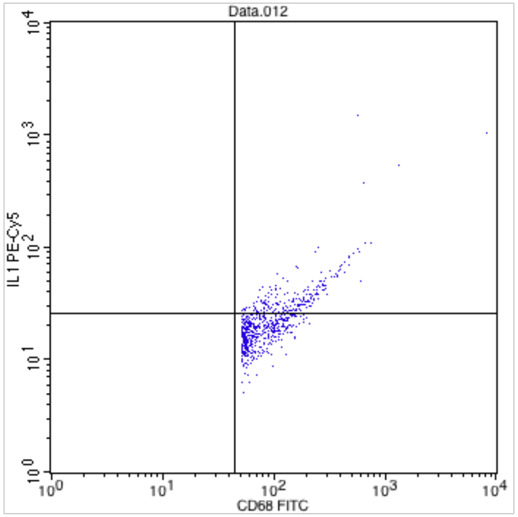

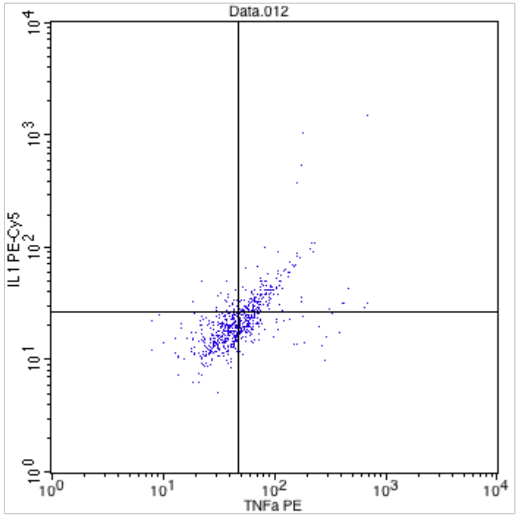

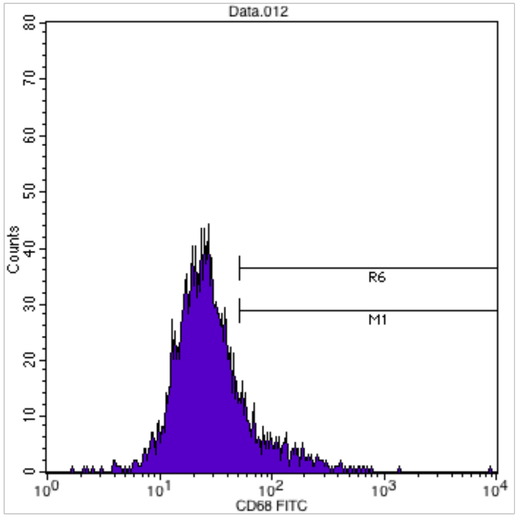

Quadrant Statistics

File: Data.012 Sample ID: 3.2
Acquisition Date: 16-Oct-24 Gate: G7
Gated Events: 659 Total Events: 71067
X Parameter: CD68 FITC (Log) Y Parameter: TNFa PE (Log)
Quad Location: 45, 47

Quad	Events	% Gated	% Total
UL	0	0.00	0.00
UR	318	48.25	0.45
LL	0	0.00	0.00
LR	341	51.75	0.48

Quadrant Statistics

File: Data.012 Sample ID: 3.2
Acquisition Date: 16-Oct-24 Gate: G7
Gated Events: 659 Total Events: 71067
X Parameter: CD68 FITC (Log) Y Parameter: IL1 PE-Cy5 (Log)
Quad Location: 45, 26

Quad	Events	% Gated	% Total
UL	0	0.00	0.00
UR	199	30.20	0.28
LL	0	0.00	0.00
LR	460	69.80	0.65

Quadrant Statistics

File: Data.012 Sample ID: 3.2
Acquisition Date: 16-Oct-24 Gate: G7
Gated Events: 659 Total Events: 71067
X Parameter: TNFa PE (Log) Y Parameter: IL1 PE-Cy5 (Log)
Quad Location: 47, 26

Quad	Events	% Gated	% Total
UL	24	3.64	0.03
UR	164	24.89	0.23
LL	321	48.71	0.45
LR	150	22.76	0.21

Histogram Statistics

File: Data.012 Sample ID: 3.2
Acquisition Date: 16-Oct-24 Gate: G2
Gated Events: 4714 Total Events: 71067
X Parameter: CD68 FITC (Log)

Marker	Left, Right	Events	% Gated	% Total
All	1, 9910	4714	100.00	6.63
M1	51, 9910	659	13.98	0.93

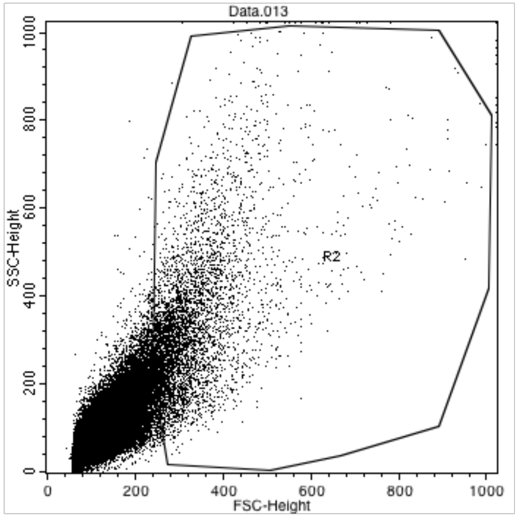

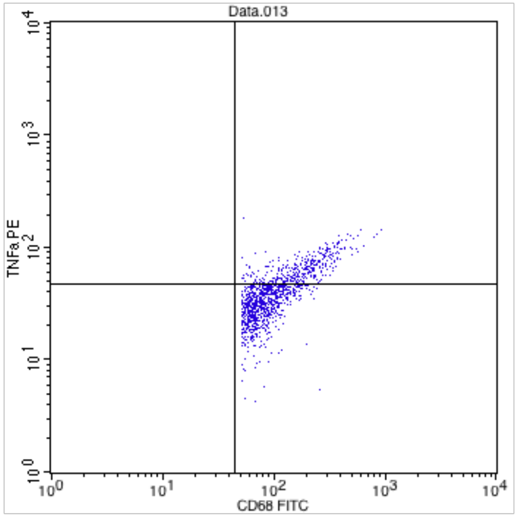

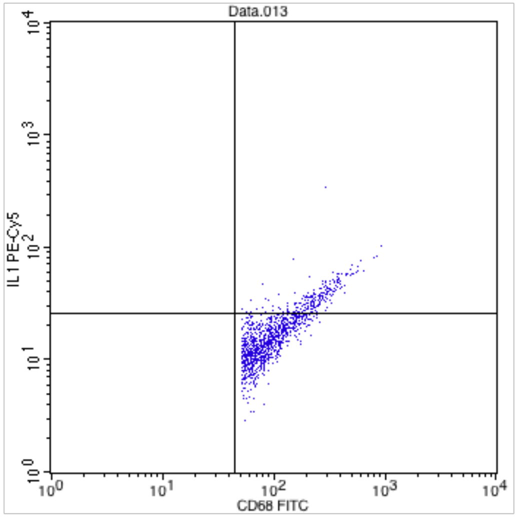

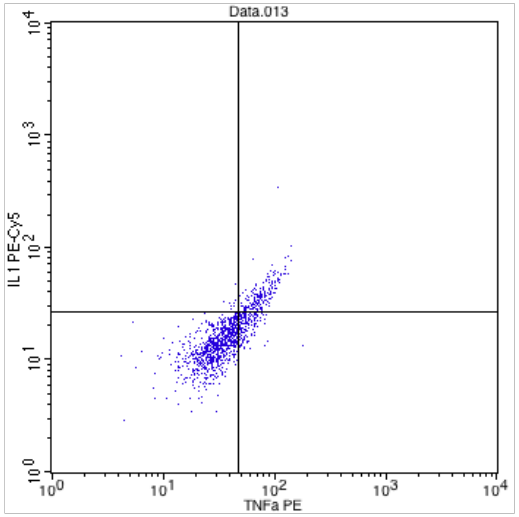

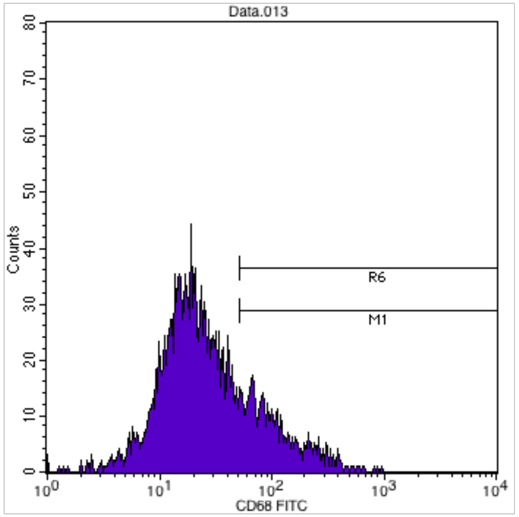

Quadrant Statistics

File: Data.013
Sample ID: 3.3
Acquisition Date: 16-Oct-24
Gate: G7
Gated Events: 1190
Total Events: 61998
X Parameter: CD68 FITC (Log)
Y Parameter: TNFa PE (Log)
Quad Location: 45, 47

Quad	Events	% Gated	% Total
UL	0	0.00	0.00
UR	357	30.00	0.58
LL	0	0.00	0.00
LR	833	70.00	1.34

Quadrant Statistics

File: Data.013
Sample ID: 3.3
Acquisition Date: 16-Oct-24
Gate: G7
Gated Events: 1190
Total Events: 61998
X Parameter: CD68 FITC (Log)
Y Parameter: IL1 PE-Cy5 (Log)
Quad Location: 45, 26

Quad	Events	% Gated	% Total
UL	0	0.00	0.00
UR	210	17.65	0.34
LL	0	0.00	0.00
LR	980	82.35	1.58

Quadrant Statistics

File: Data.013
Sample ID: 3.3
Acquisition Date: 16-Oct-24
Gate: G7
Gated Events: 1190
Total Events: 61998
X Parameter: TNFa PE (Log)
Y Parameter: IL1 PE-Cy5 (Log)
Quad Location: 47, 26

Quad	Events	% Gated	% Total
UL	12	1.01	0.02
UR	188	15.80	0.30
LL	829	69.66	1.34
LR	161	13.53	0.26

Histogram Statistics

File: Data.013
Sample ID: 3.3
Acquisition Date: 16-Oct-24
Gate: G2
Gated Events: 5360
Total Events: 61998
X Parameter: CD68 FITC (Log)

Marker	Left, Right	Events	% Gated	% Total
All	1, 9910	5360	100.00	8.65
M1	51, 9910	1190	22.20	1.92

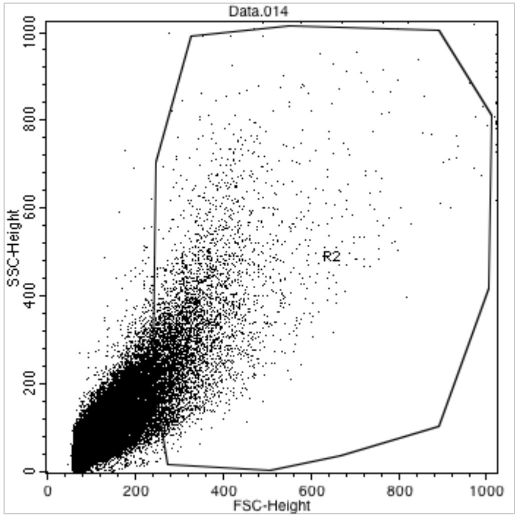

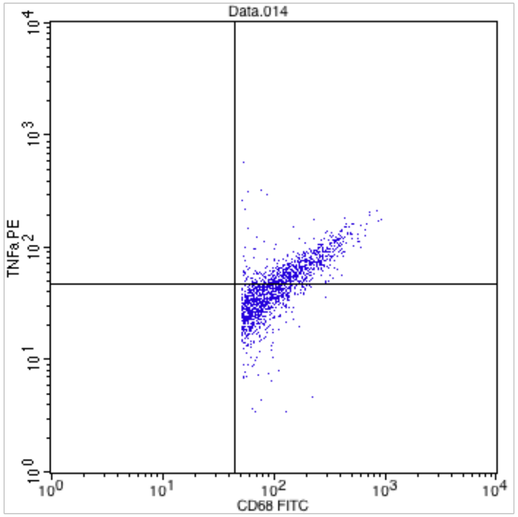

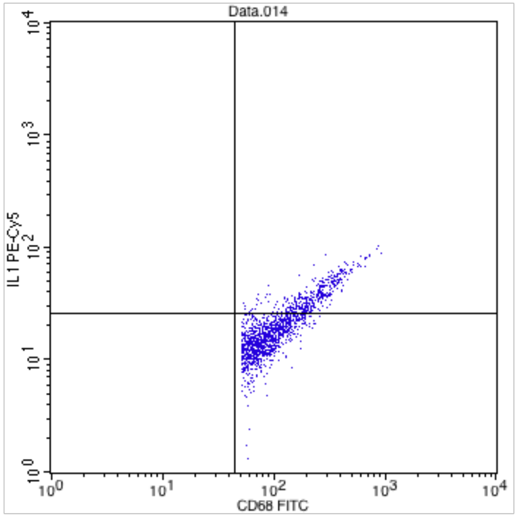

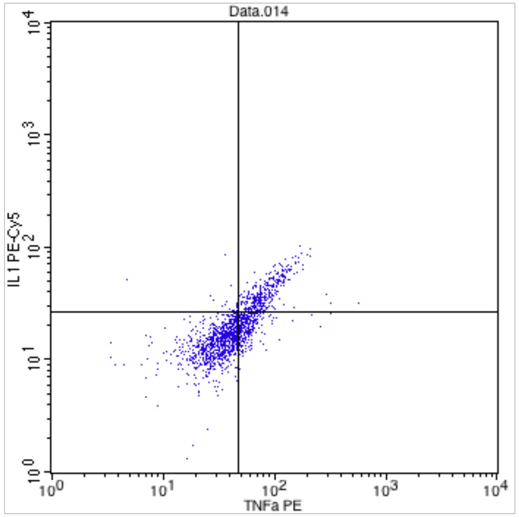

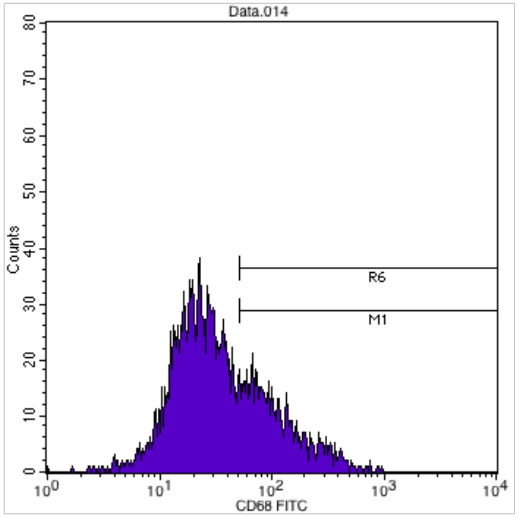

Quadrant Statistics

File: Data.014
Acquisition Date: 16-Oct-24
Gated Events: 1612
X Parameter: CD68 FITC (Log)
Quad Location: 45, 47
Sample ID: 4.1
Gate: G7
Total Events: 66429
Y Parameter: TNFa PE (Log)

Quad	Events	% Gated	% Total
UL	0	0.00	0.00
UR	685	42.49	1.03
LL	0	0.00	0.00
LR	927	57.51	1.40

Quadrant Statistics

File: Data.014
Acquisition Date: 16-Oct-24
Gated Events: 1612
X Parameter: CD68 FITC (Log)
Quad Location: 45, 26
Sample ID: 4.1
Gate: G7
Total Events: 66429
Y Parameter: IL1 PE-Cy5 (Log)

Quad	Events	% Gated	% Total
UL	0	0.00	0.00
UR	414	25.68	0.62
LL	0	0.00	0.00
LR	1198	74.32	1.80

Quadrant Statistics

File: Data.014
Acquisition Date: 16-Oct-24
Gated Events: 1612
X Parameter: TNFa PE (Log)
Quad Location: 47, 26
Sample ID: 4.1
Gate: G7
Total Events: 66429
Y Parameter: IL1 PE-Cy5 (Log)

Quad	Events	% Gated	% Total
UL	19	1.18	0.03
UR	366	22.70	0.55
LL	927	57.51	1.40
LR	300	18.61	0.45

Histogram Statistics

File: Data.014
Acquisition Date: 16-Oct-24
Gated Events: 5308
X Parameter: CD68 FITC (Log)
Sample ID: 4.1
Gate: G2
Total Events: 66429

Marker	Left, Right	Events	% Gated	% Total
All	1, 9910	5308	100.00	7.99
M1	51, 9910	1612	30.37	2.43

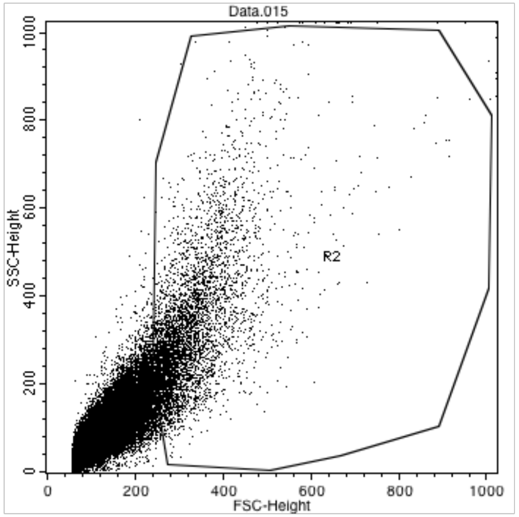

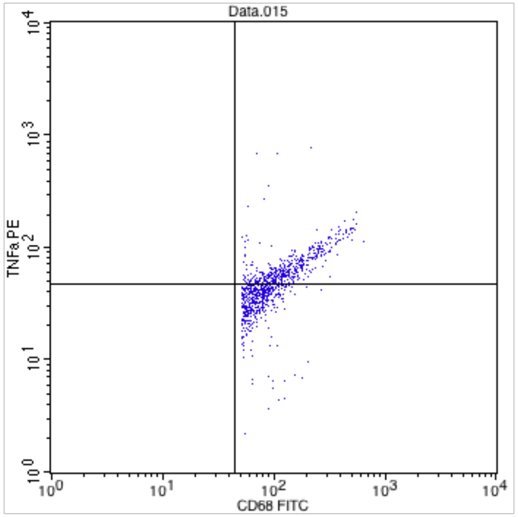

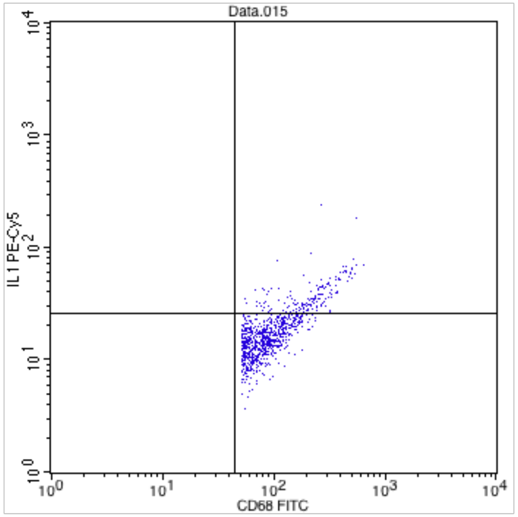

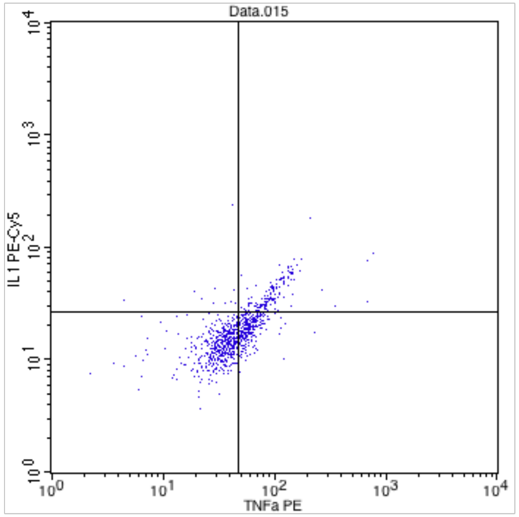

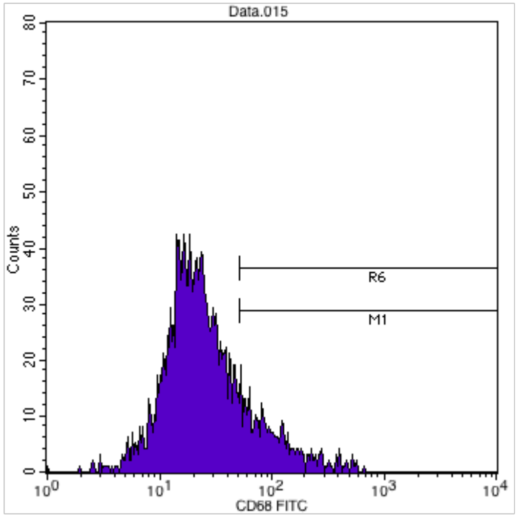

Quadrant Statistics

File: Data.015
Acquisition Date: 16-Oct-24
Gated Events: 902
X Parameter: CD68 FITC (Log)
Quad Location: 45, 47
Sample ID: 4.2
Gate: G7
Total Events: 67218
Y Parameter: TNFa PE (Log)

Quad	Events	% Gated	% Total
UL	0	0.00	0.00
UR	409	45.34	0.61
LL	0	0.00	0.00
LR	493	54.66	0.73

Quadrant Statistics

File: Data.015
Acquisition Date: 16-Oct-24
Gated Events: 902
X Parameter: CD68 FITC (Log)
Quad Location: 45, 26
Sample ID: 4.2
Gate: G7
Total Events: 67218
Y Parameter: IL1 PE-Cy5 (Log)

Quad	Events	% Gated	% Total
UL	0	0.00	0.00
UR	162	17.96	0.24
LL	0	0.00	0.00
LR	740	82.04	1.10

Quadrant Statistics

File: Data.015
Acquisition Date: 16-Oct-24
Gated Events: 902
X Parameter: TNFa PE (Log)
Quad Location: 47, 26
Sample ID: 4.2
Gate: G7
Total Events: 67218
Y Parameter: IL1 PE-Cy5 (Log)

Quad	Events	% Gated	% Total
UL	10	1.11	0.01
UR	142	15.74	0.21
LL	489	54.21	0.73
LR	261	28.94	0.39

Histogram Statistics

File: Data.015
Acquisition Date: 16-Oct-24
Gated Events: 5320
X Parameter: CD68 FITC (Log)
Sample ID: 4.2
Gate: G2
Total Events: 67218

Marker	Left, Right	Events	% Gated	% Total
All	1, 9910	5320	100.00	7.91
M1	51, 9910	902	16.95	1.34

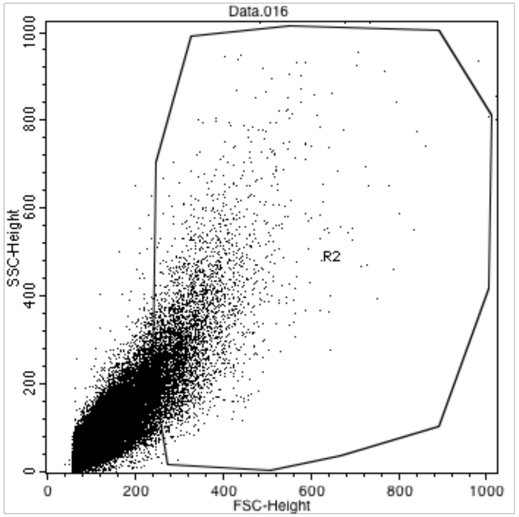

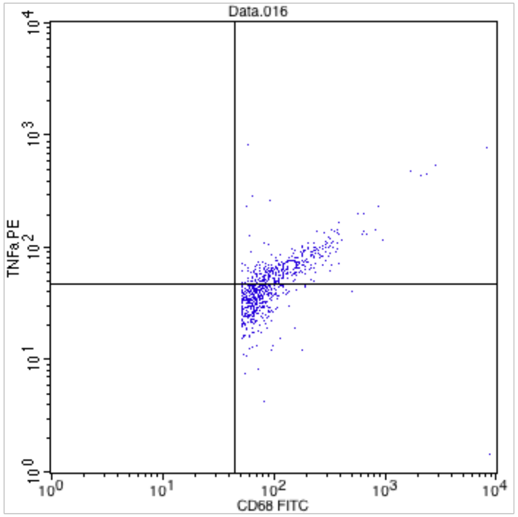

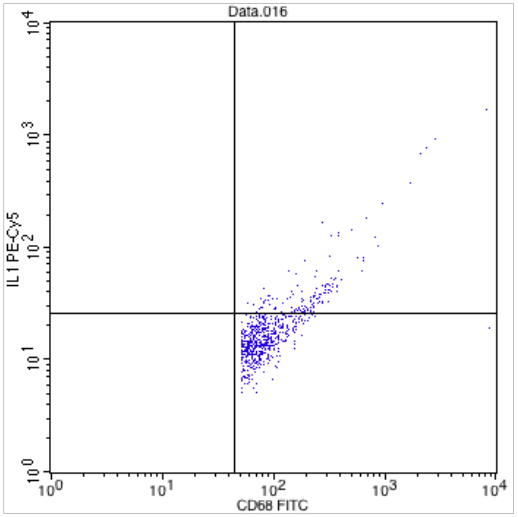

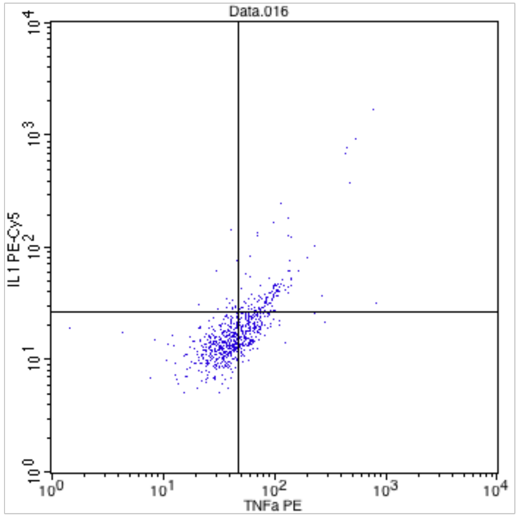

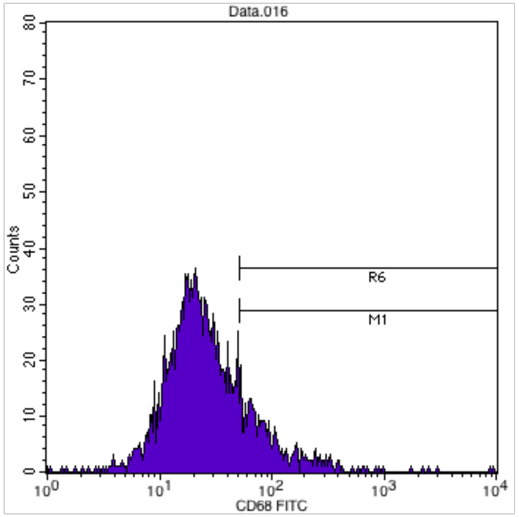

Quadrant Statistics

File: Data.016 Sample ID: 4.3
Acquisition Date: 16-Oct-24 Gate: G7
Gated Events: 731 Total Events: 86872
X Parameter: CD68 FITC (Log) Y Parameter: TNFa PE (Log)
Quad Location: 45, 47

Quad	Events	% Gated	% Total
UL	0	0.00	0.00
UR	324	44.32	0.37
LL	0	0.00	0.00
LR	407	55.68	0.47

Quadrant Statistics

File: Data.016 Sample ID: 4.3
Acquisition Date: 16-Oct-24 Gate: G7
Gated Events: 731 Total Events: 86872
X Parameter: CD68 FITC (Log) Y Parameter: IL1 PE-Cy5 (Log)
Quad Location: 45, 26

Quad	Events	% Gated	% Total
UL	0	0.00	0.00
UR	134	18.33	0.15
LL	0	0.00	0.00
LR	597	81.67	0.69

Quadrant Statistics

File: Data.016 Sample ID: 4.3
Acquisition Date: 16-Oct-24 Gate: G7
Gated Events: 731 Total Events: 86872
X Parameter: TNFa PE (Log) Y Parameter: IL1 PE-Cy5 (Log)
Quad Location: 47, 26

Quad	Events	% Gated	% Total
UL	13	1.78	0.01
UR	112	15.32	0.13
LL	399	54.58	0.46
LR	207	28.32	0.24

Histogram Statistics

File: Data.016 Sample ID: 4.3
Acquisition Date: 16-Oct-24 Gate: G2
Gated Events: 4655 Total Events: 86872
X Parameter: CD68 FITC (Log)

Marker	Left, Right	Events	% Gated	% Total
All	1, 9910	4655	100.00	5.36
M1	51, 9910	731	15.70	0.84

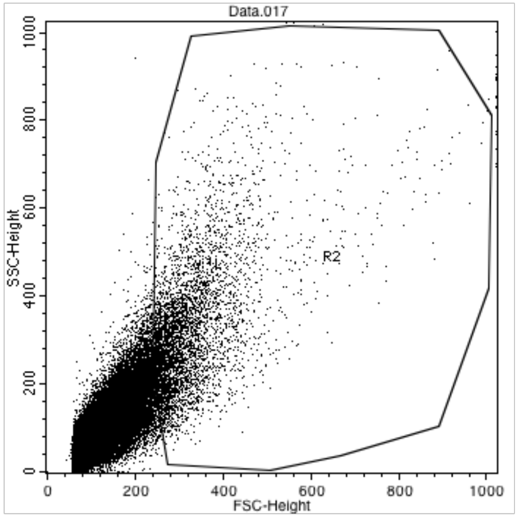

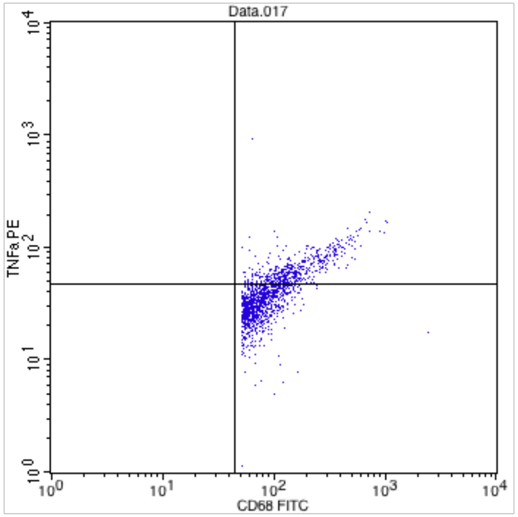

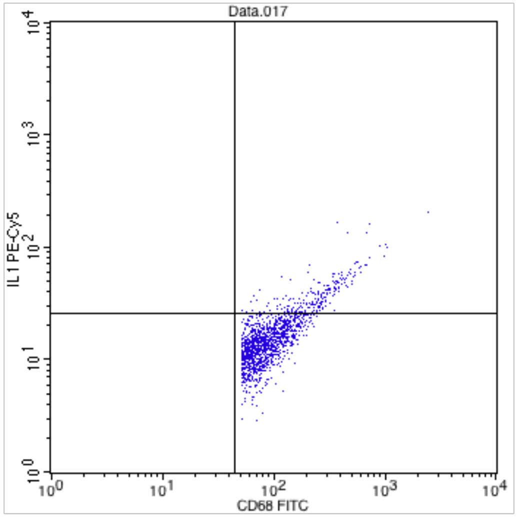

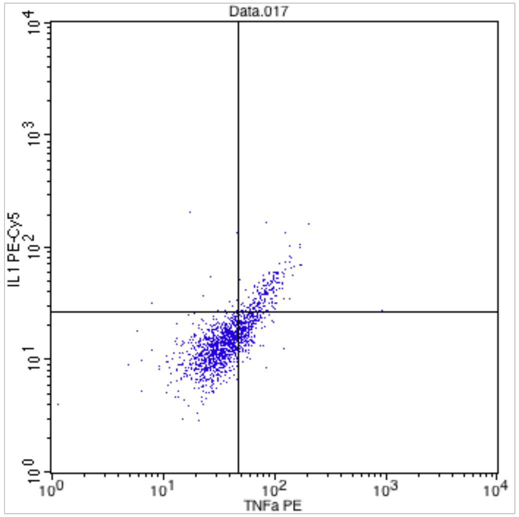

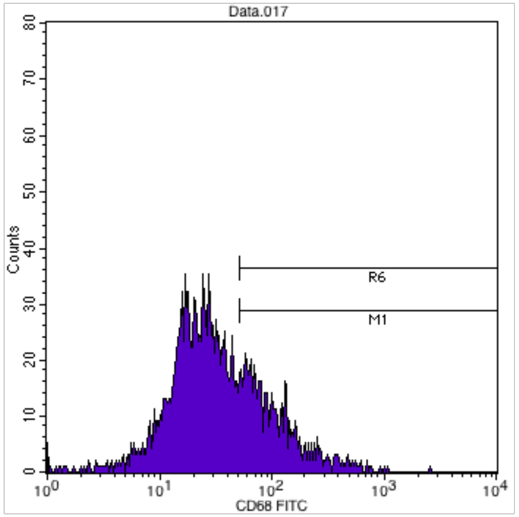

Quadrant Statistics

File: Data.017
Acquisition Date: 16-Oct-24
Gated Events: 1491
X Parameter: CD68 FITC (Log)
Quad Location: 45, 47

Sample ID: 5.1
Gate: G7
Total Events: 66865
Y Parameter: TNFa PE (Log)

Quad	Events	% Gated	% Total
UL	0	0.00	0.00
UR	470	31.52	0.70
LL	0	0.00	0.00
LR	1021	68.48	1.53

Quadrant Statistics

File: Data.017
Acquisition Date: 16-Oct-24
Gated Events: 1491
X Parameter: CD68 FITC (Log)
Quad Location: 45, 26

Sample ID: 5.1
Gate: G7
Total Events: 66865
Y Parameter: IL1 PE-Cy5 (Log)

Quad	Events	% Gated	% Total
UL	0	0.00	0.00
UR	219	14.69	0.33
LL	0	0.00	0.00
LR	1272	85.31	1.90

Quadrant Statistics

File: Data.017
Acquisition Date: 16-Oct-24
Gated Events: 1491
X Parameter: TNFa PE (Log)
Quad Location: 47, 26

Sample ID: 5.1
Gate: G7
Total Events: 66865
Y Parameter: IL1 PE-Cy5 (Log)

Quad	Events	% Gated	% Total
UL	14	0.94	0.02
UR	191	12.81	0.29
LL	1018	68.28	1.52
LR	268	17.97	0.40

Histogram Statistics

File: Data.017
Acquisition Date: 16-Oct-24
Gated Events: 5059
X Parameter: CD68 FITC (Log)

Sample ID: 5.1
Gate: G2
Total Events: 66865

Marker	Left, Right	Events	% Gated	% Total
All	1, 9910	5059	100.00	7.57
M1	51, 9910	1491	29.47	2.23

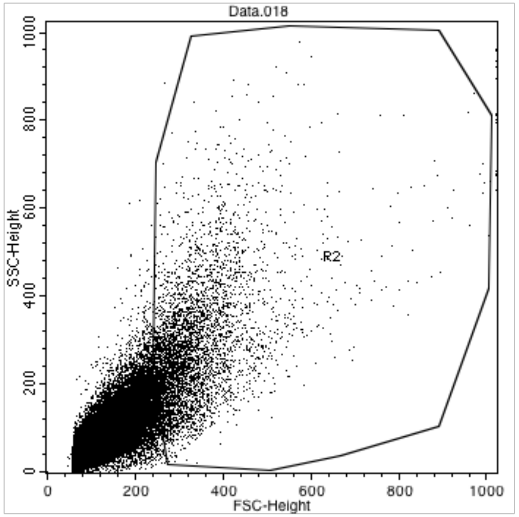

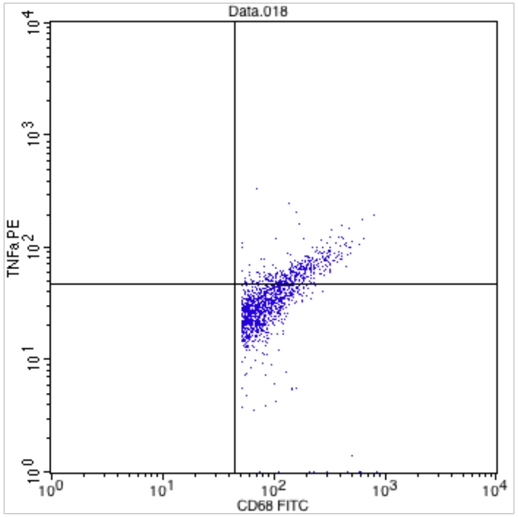

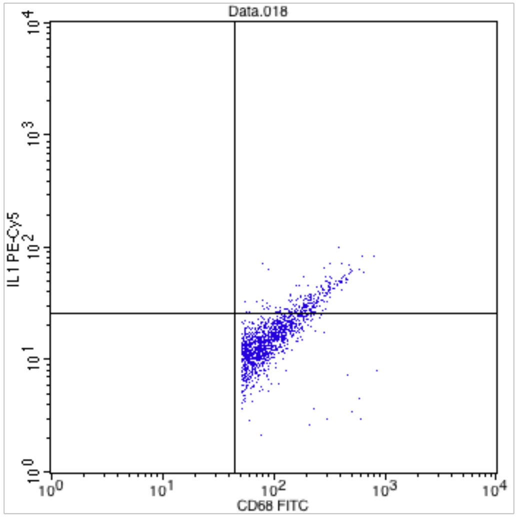

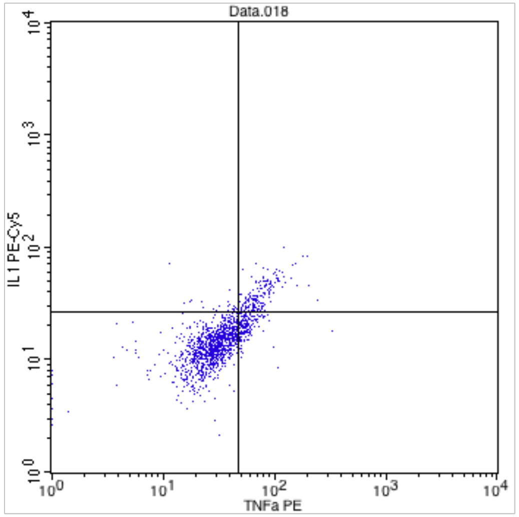

Quadrant Statistics

File: Data.018
Acquisition Date: 16-Oct-24
Gated Events: 1444
X Parameter: CD68 FITC (Log)
Quad Location: 45, 47

Sample ID: 5.2
Gate: G7
Total Events: 61868
Y Parameter: TNFa PE (Log)

Quad	Events	% Gated	% Total
UL	0	0.00	0.00
UR	375	25.97	0.61
LL	0	0.00	0.00
LR	1069	74.03	1.73

Quadrant Statistics

File: Data.018
Acquisition Date: 16-Oct-24
Gated Events: 1444
X Parameter: CD68 FITC (Log)
Quad Location: 45, 26

Sample ID: 5.2
Gate: G7
Total Events: 61868
Y Parameter: IL1 PE-Cy5 (Log)

Quad	Events	% Gated	% Total
UL	0	0.00	0.00
UR	225	15.58	0.36
LL	0	0.00	0.00
LR	1219	84.42	1.97

Quadrant Statistics

File: Data.018
Acquisition Date: 16-Oct-24
Gated Events: 1444
X Parameter: TNFa PE (Log)
Quad Location: 47, 26

Sample ID: 5.2
Gate: G7
Total Events: 61868
Y Parameter: IL1 PE-Cy5 (Log)

Quad	Events	% Gated	% Total
UL	26	1.80	0.04
UR	183	12.67	0.30
LL	1052	72.85	1.70
LR	183	12.67	0.30

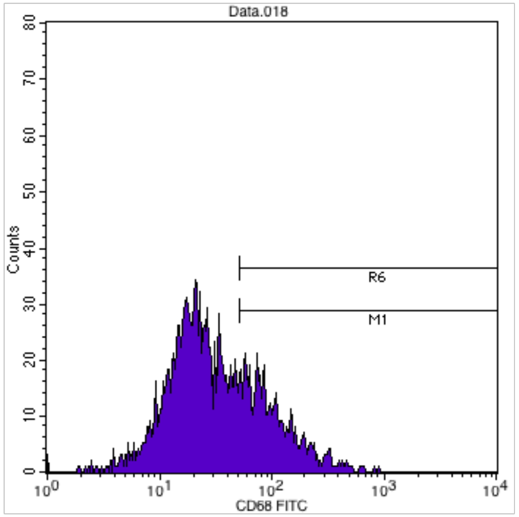

Histogram Statistics

File: Data.018
Acquisition Date: 16-Oct-24
Gated Events: 5073
X Parameter: CD68 FITC (Log)

Sample ID: 5.2
Gate: G2
Total Events: 61868

Marker	Left, Right	Events	% Gated	% Total
All	1, 9910	5073	100.00	8.20
M1	51, 9910	1444	28.46	2.33

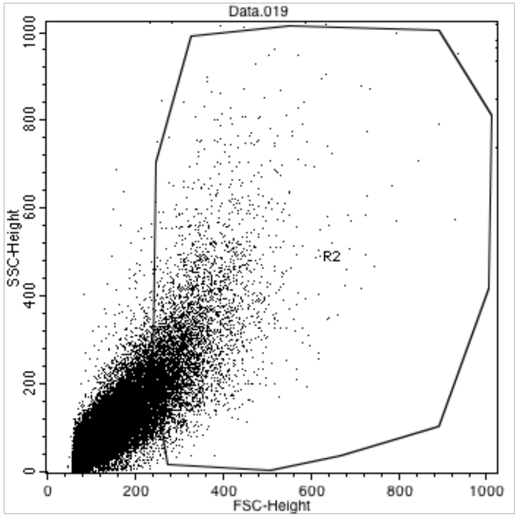

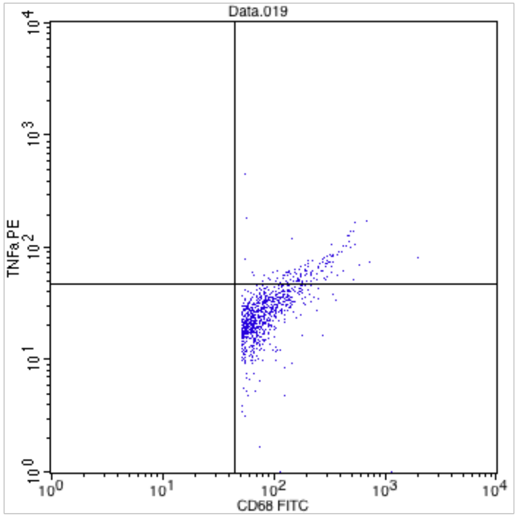

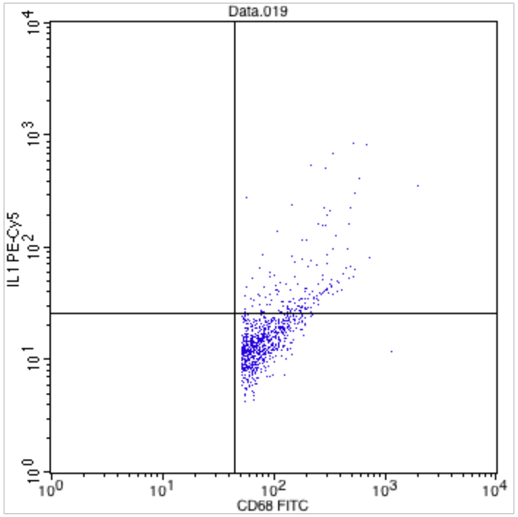

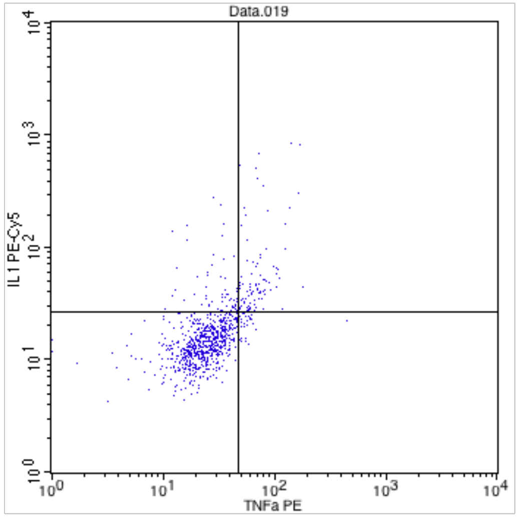

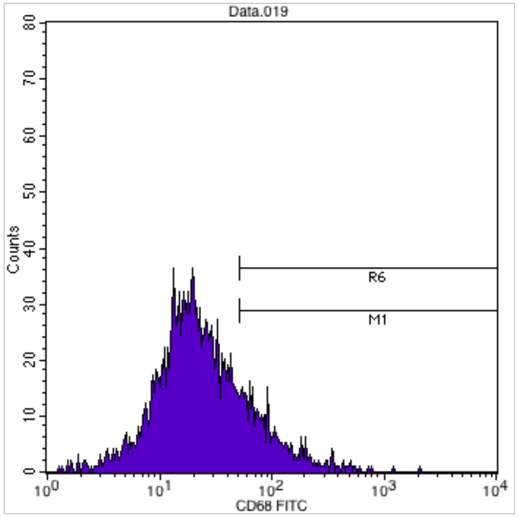

Quadrant Statistics

File: Data.019
Acquisition Date: 16-Oct-24
Gated Events: 883
X Parameter: CD68 FITC (Log)
Quad Location: 45, 47
Sample ID: 5.3
Gate: G7
Total Events: 70660
Y Parameter: TNFa PE (Log)

Quad	Events	% Gated	% Total
UL	0	0.00	0.00
UR	123	13.93	0.17
LL	0	0.00	0.00
LR	760	86.07	1.08

Quadrant Statistics

File: Data.019
Acquisition Date: 16-Oct-24
Gated Events: 883
X Parameter: CD68 FITC (Log)
Quad Location: 45, 26
Sample ID: 5.3
Gate: G7
Total Events: 70660
Y Parameter: IL1 PE-Cy5 (Log)

Quad	Events	% Gated	% Total
UL	0	0.00	0.00
UR	158	17.89	0.22
LL	0	0.00	0.00
LR	725	82.11	1.03

Quadrant Statistics

File: Data.019
Acquisition Date: 16-Oct-24
Gated Events: 883
X Parameter: TNFa PE (Log)
Quad Location: 47, 26
Sample ID: 5.3
Gate: G7
Total Events: 70660
Y Parameter: IL1 PE-Cy5 (Log)

Quad	Events	% Gated	% Total
UL	64	7.25	0.09
UR	89	10.08	0.13
LL	700	79.28	0.99
LR	30	3.40	0.04

Histogram Statistics

File: Data.019
Acquisition Date: 16-Oct-24
Gated Events: 5113
X Parameter: CD68 FITC (Log)
Sample ID: 5.3
Gate: G2
Total Events: 70660

Marker	Left, Right	Events	% Gated	% Total
All	1, 9910	5113	100.00	7.24
M1	51, 9910	883	17.27	1.25

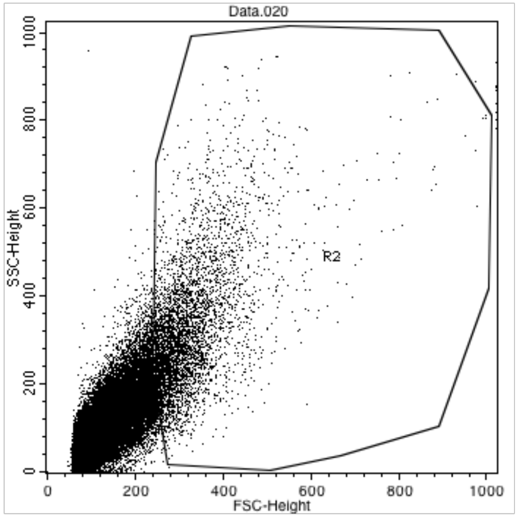

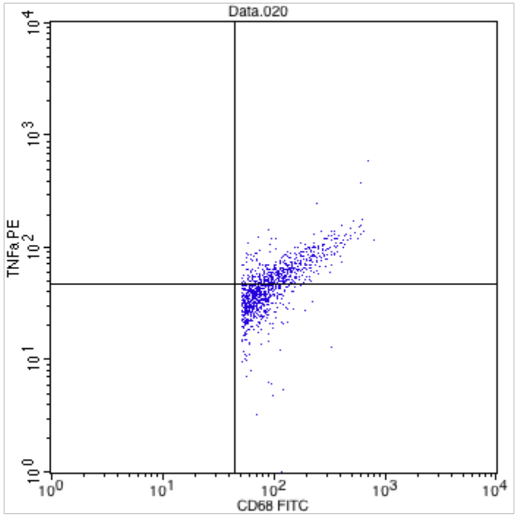

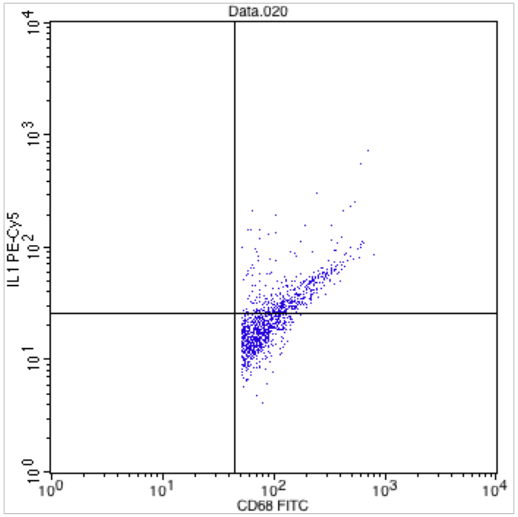

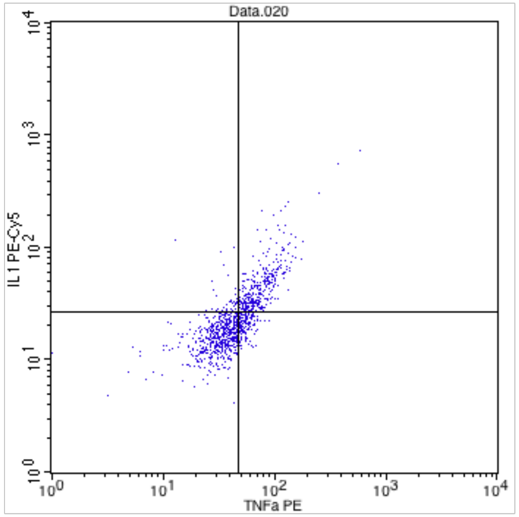

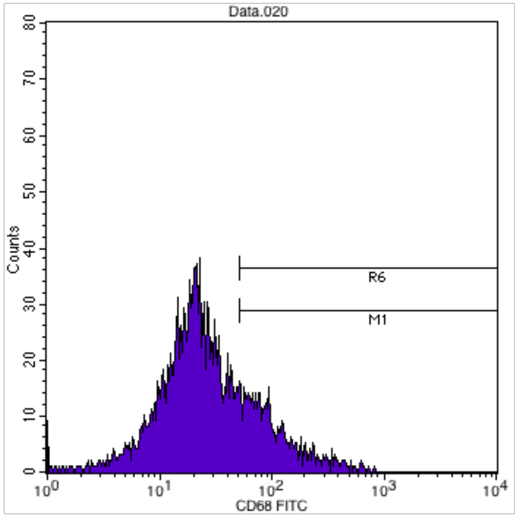

Quadrant Statistics

File: Data.020
Acquisition Date: 16-Oct-24
Gated Events: 1102
X Parameter: CD68 FITC (Log)
Quad Location: 45, 47
Sample ID: 6.2
Gate: G7
Total Events: 68732
Y Parameter: TNFa PE (Log)

Quad	Events	% Gated	% Total
UL	0	0.00	0.00
UR	502	45.55	0.73
LL	0	0.00	0.00
LR	600	54.45	0.87

Quadrant Statistics

File: Data.020
Acquisition Date: 16-Oct-24
Gated Events: 1102
X Parameter: CD68 FITC (Log)
Quad Location: 45, 26
Sample ID: 6.2
Gate: G7
Total Events: 68732
Y Parameter: IL1 PE-Cy5 (Log)

Quad	Events	% Gated	% Total
UL	0	0.00	0.00
UR	411	37.30	0.60
LL	0	0.00	0.00
LR	691	62.70	1.01

Quadrant Statistics

File: Data.020
Acquisition Date: 16-Oct-24
Gated Events: 1102
X Parameter: TNFa PE (Log)
Quad Location: 47, 26
Sample ID: 6.2
Gate: G7
Total Events: 68732
Y Parameter: IL1 PE-Cy5 (Log)

Quad	Events	% Gated	% Total
UL	45	4.08	0.07
UR	346	31.40	0.50
LL	564	51.18	0.82
LR	147	13.34	0.21

Histogram Statistics

File: Data.020
Acquisition Date: 16-Oct-24
Gated Events: 4947
X Parameter: CD68 FITC (Log)
Sample ID: 6.2
Gate: G2
Total Events: 68732

Marker	Left, Right	Events	% Gated	% Total
All	1, 9910	4947	100.00	7.20
M1	51, 9910	1102	22.28	1.60

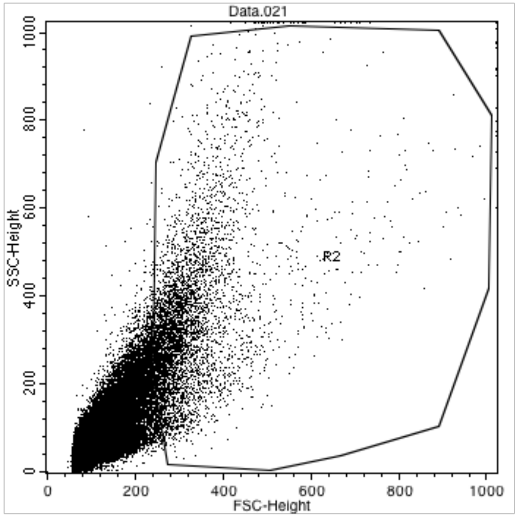

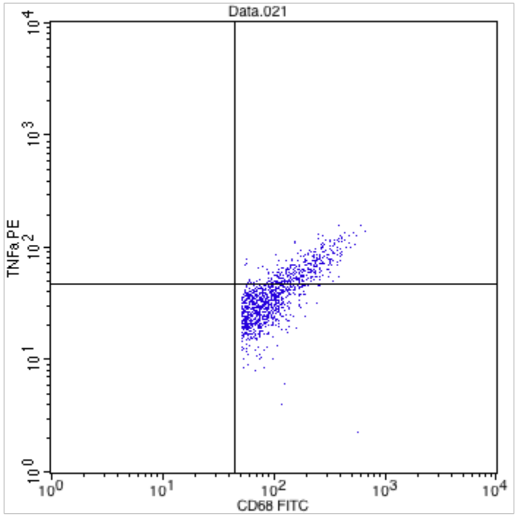

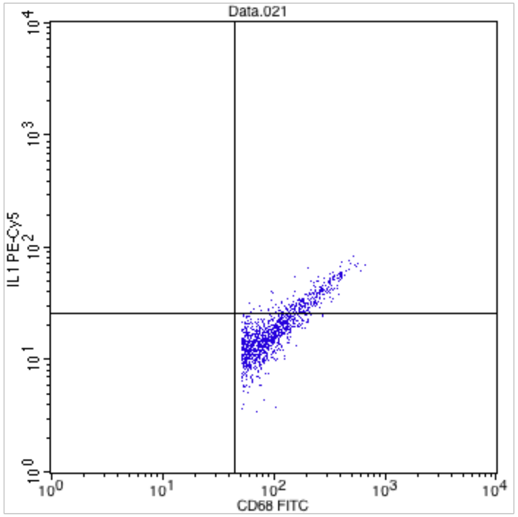

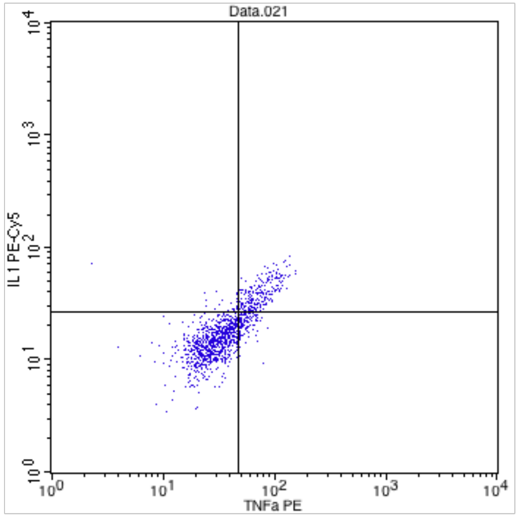

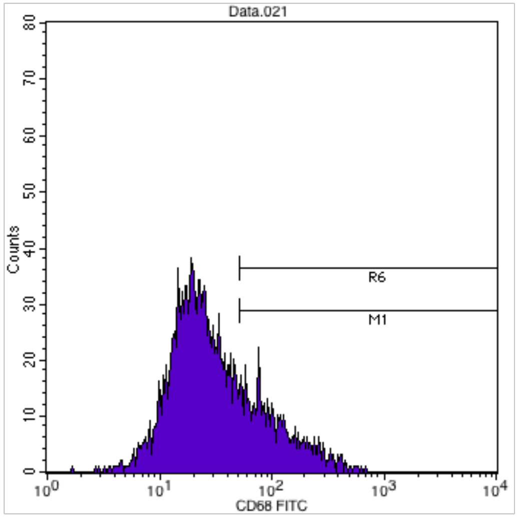

Quadrant Statistics

File: Data.021 Sample ID: 6.2
Acquisition Date: 16-Oct-24 Gate: G7
Gated Events: 1233 Total Events: 61890
X Parameter: CD68 FITC (Log) Y Parameter: TNFa PE (Log)
Quad Location: 45, 47

Quad	Events	% Gated	% Total
UL	0	0.00	0.00
UR	350	28.39	0.57
LL	0	0.00	0.00
LR	883	71.61	1.43

Quadrant Statistics

File: Data.021 Sample ID: 6.2
Acquisition Date: 16-Oct-24 Gate: G7
Gated Events: 1233 Total Events: 61890
X Parameter: CD68 FITC (Log) Y Parameter: IL1 PE-Cy5 (Log)
Quad Location: 45, 26

Quad	Events	% Gated	% Total
UL	0	0.00	0.00
UR	272	22.06	0.44
LL	0	0.00	0.00
LR	961	77.94	1.55

Quadrant Statistics

File: Data.021 Sample ID: 6.2
Acquisition Date: 16-Oct-24 Gate: G7
Gated Events: 1233 Total Events: 61890
X Parameter: TNFa PE (Log) Y Parameter: IL1 PE-Cy5 (Log)
Quad Location: 47, 26

Quad	Events	% Gated	% Total
UL	31	2.51	0.05
UR	228	18.49	0.37
LL	855	69.34	1.38
LR	119	9.65	0.19

Histogram Statistics

File: Data.021 Sample ID: 6.2
Acquisition Date: 16-Oct-24 Gate: G2
Gated Events: 5149 Total Events: 61890
X Parameter: CD68 FITC (Log)

Marker	Left, Right	Events	% Gated	% Total
All	1, 9910	5149	100.00	8.32
M1	51, 9910	1233	23.95	1.99

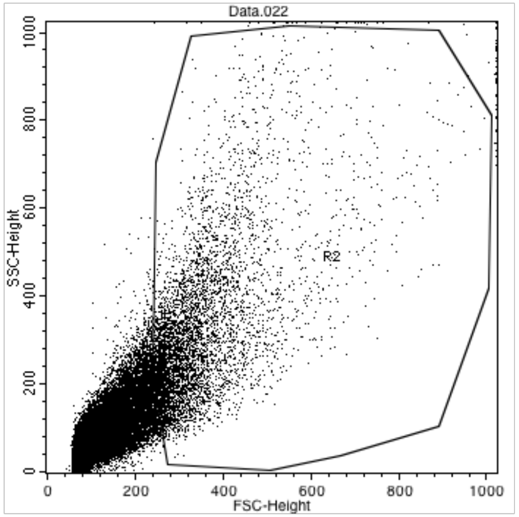

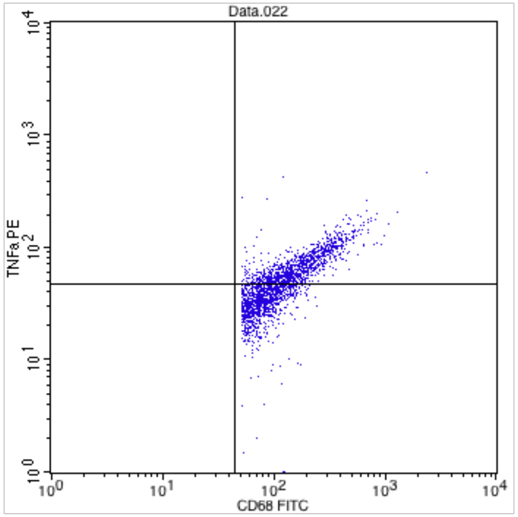

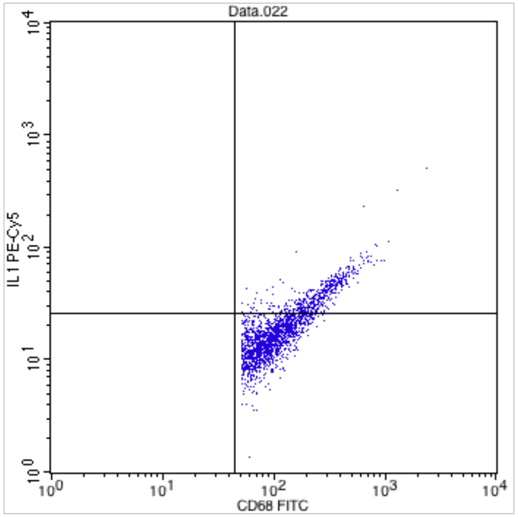

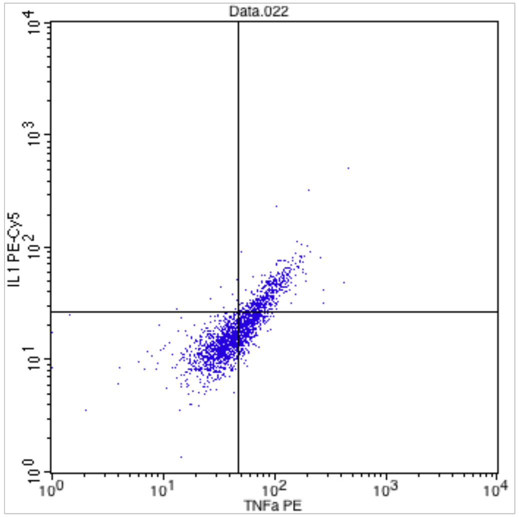

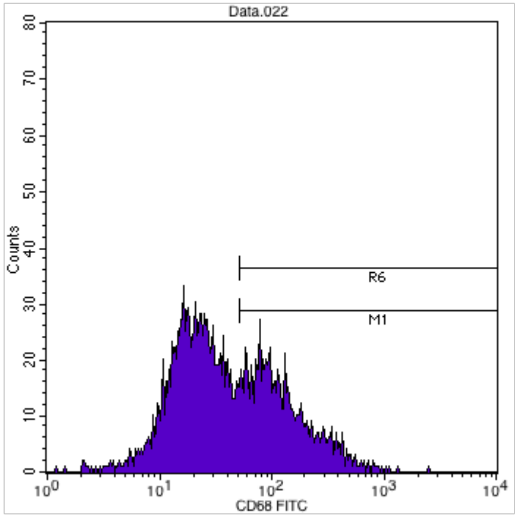

Quadrant Statistics

File: Data.022
Acquisition Date: 16-Oct-24
Gated Events: 2234
X Parameter: CD68 FITC (Log)
Quad Location: 45, 47
Sample ID: 7.1
Gate: G7
Total Events: 54635
Y Parameter: TNFa PE (Log)

Quad	Events	% Gated	% Total
UL	0	0.00	0.00
UR	1072	47.99	1.96
LL	0	0.00	0.00
LR	1162	52.01	2.13

Quadrant Statistics

File: Data.022
Acquisition Date: 16-Oct-24
Gated Events: 2234
X Parameter: CD68 FITC (Log)
Quad Location: 45, 26
Sample ID: 7.1
Gate: G7
Total Events: 54635
Y Parameter: IL1 PE-Cy5 (Log)

Quad	Events	% Gated	% Total
UL	0	0.00	0.00
UR	540	24.17	0.99
LL	0	0.00	0.00
LR	1694	75.83	3.10

Quadrant Statistics

File: Data.022
Acquisition Date: 16-Oct-24
Gated Events: 2234
X Parameter: TNFa PE (Log)
Quad Location: 47, 26
Sample ID: 7.1
Gate: G7
Total Events: 54635
Y Parameter: IL1 PE-Cy5 (Log)

Quad	Events	% Gated	% Total
UL	12	0.54	0.02
UR	499	22.34	0.91
LL	1163	52.06	2.13
LR	560	25.07	1.02

Histogram Statistics

File: Data.022
Acquisition Date: 16-Oct-24
Gated Events: 5723
X Parameter: CD68 FITC (Log)
Sample ID: 7.1
Gate: G2
Total Events: 54635

Marker	Left, Right	Events	% Gated	% Total
All	1, 9910	5723	100.00	10.47
M1	51, 9910	2234	39.04	4.09

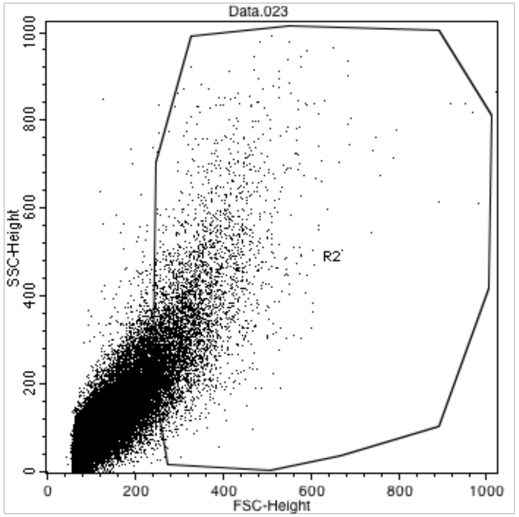

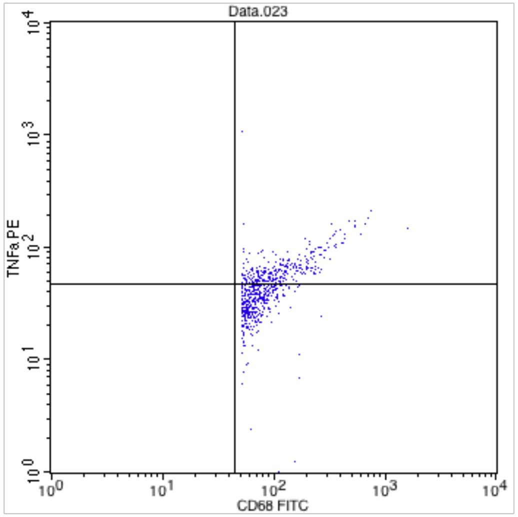

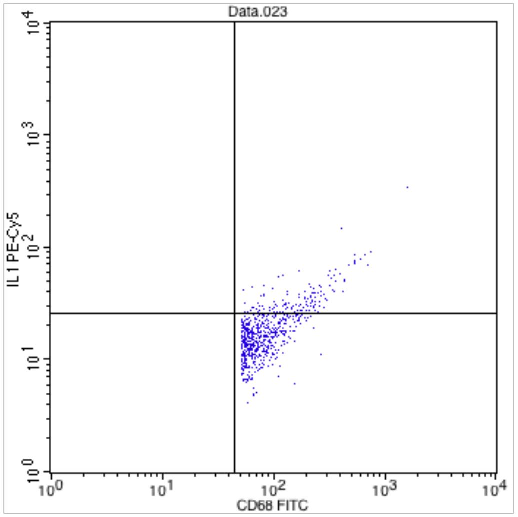

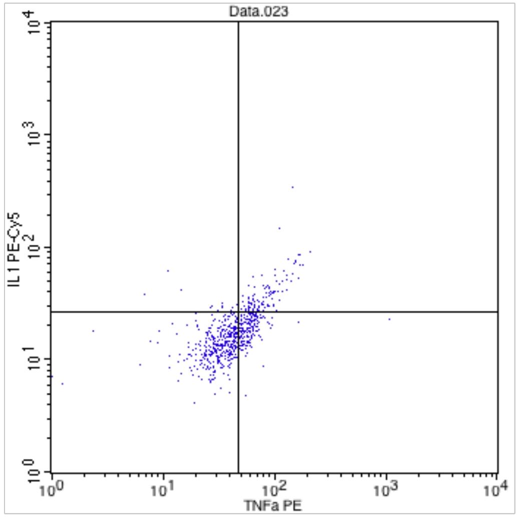

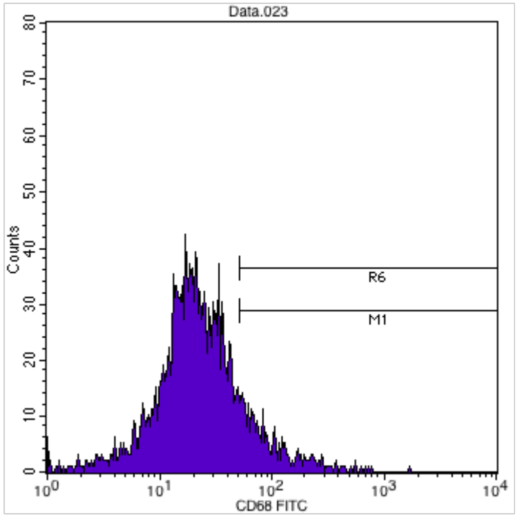

Quadrant Statistics

File: Data.023 Sample ID: 7.3
Acquisition Date: 16-Oct-24 Gate: G7
Gated Events: 667 Total Events: 65735
X Parameter: CD68 FITC (Log) Y Parameter: TNFa PE (Log)
Quad Location: 45, 47

Quad	Events	% Gated	% Total
UL	0	0.00	0.00
UR	281	42.13	0.43
LL	0	0.00	0.00
LR	386	57.87	0.59

Quadrant Statistics

File: Data.023 Sample ID: 7.3
Acquisition Date: 16-Oct-24 Gate: G7
Gated Events: 667 Total Events: 65735
X Parameter: CD68 FITC (Log) Y Parameter: IL1 PE-Cy5 (Log)
Quad Location: 45, 26

Quad	Events	% Gated	% Total
UL	0	0.00	0.00
UR	124	18.59	0.19
LL	0	0.00	0.00
LR	543	81.41	0.83

Quadrant Statistics

File: Data.023 Sample ID: 7.3
Acquisition Date: 16-Oct-24 Gate: G7
Gated Events: 667 Total Events: 65735
X Parameter: TNFa PE (Log) Y Parameter: IL1 PE-Cy5 (Log)
Quad Location: 47, 26

Quad	Events	% Gated	% Total
UL	16	2.40	0.02
UR	104	15.59	0.16
LL	372	55.77	0.57
LR	175	26.24	0.27

Histogram Statistics

File: Data.023 Sample ID: 7.3
Acquisition Date: 16-Oct-24 Gate: G2
Gated Events: 5198 Total Events: 65735
X Parameter: CD68 FITC (Log)

Marker	Left, Right	Events	% Gated	% Total
All	1, 9910	5198	100.00	7.91
M1	51, 9910	667	12.83	1.01

CD68-TNFa-IL1
G2=R2 (Populasi sel hidup)
G7=R2 and R6 (Populasi total sel CD68)
17-10-2024

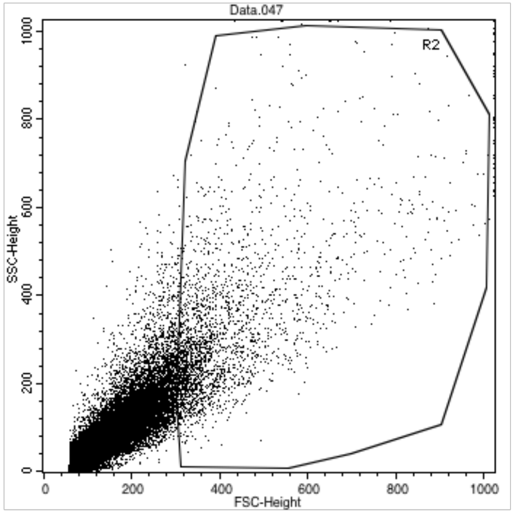

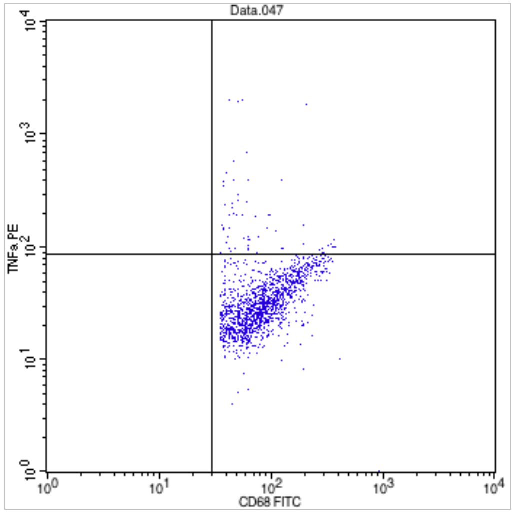

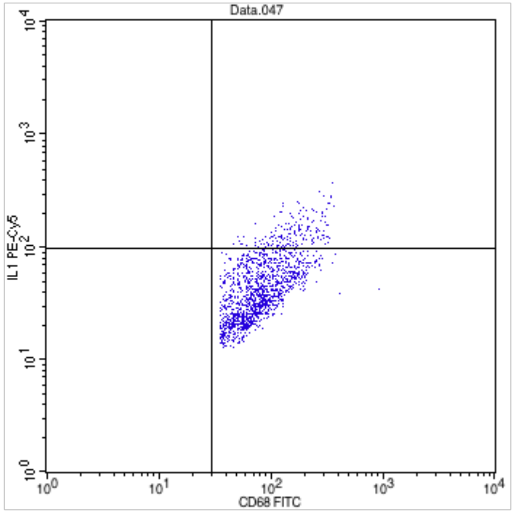

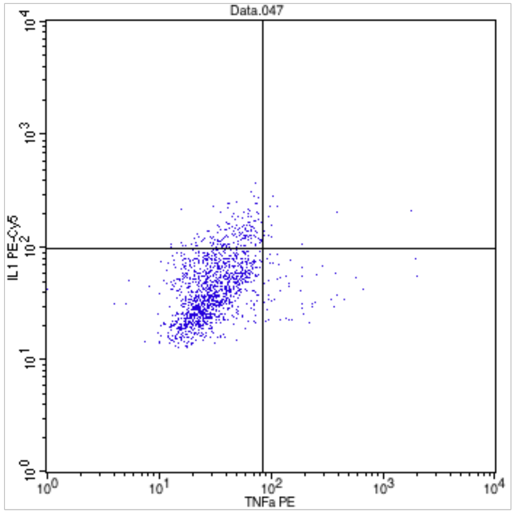

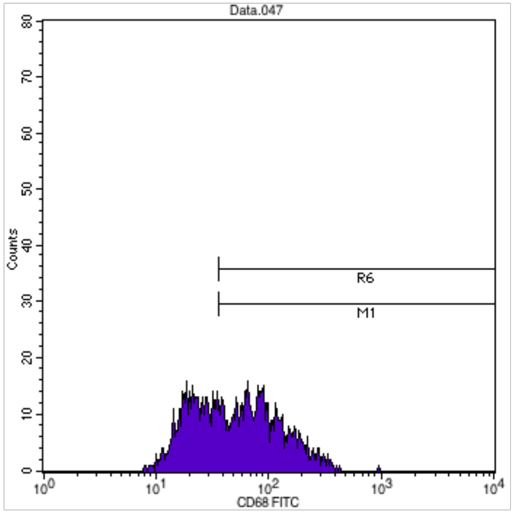

Quadrant Statistics

File: Data.047
Acquisition Date: 18-Oct-24
Gated Events: 1508
X Parameter: CD68 FITC (Log)
Quad Location: 30, 84

Sample ID: Norm.4
Gate: G7
Total Events: 71179
Y Parameter: TNFa PE (Log)

Quad	Events	% Gated	% Total
UL	0	0.00	0.00
UR	62	4.11	0.09
LL	0	0.00	0.00
LR	1446	95.89	2.03

Quadrant Statistics

File: Data.047
Acquisition Date: 18-Oct-24
Gated Events: 1508
X Parameter: CD68 FITC (Log)
Quad Location: 30, 96

Sample ID: Norm.4
Gate: G7
Total Events: 71179
Y Parameter: IL1 PE-Cy5 (Log)

Quad	Events	% Gated	% Total
UL	0	0.00	0.00
UR	173	11.47	0.24
LL	0	0.00	0.00
LR	1335	88.53	1.88

Quadrant Statistics

File: Data.047
Acquisition Date: 18-Oct-24
Gated Events: 1508
X Parameter: TNFa PE (Log)
Quad Location: 84, 96

Sample ID: Norm.4
Gate: G7
Total Events: 71179
Y Parameter: IL1 PE-Cy5 (Log)

Quad	Events	% Gated	% Total
UL	161	10.68	0.23
UR	12	0.80	0.02
LL	1285	85.21	1.81
LR	50	3.32	0.07

Histogram Statistics

File: Data.047
Acquisition Date: 18-Oct-24
Gated Events: 2498
X Parameter: CD68 FITC (Log)

Sample ID: Norm.4
Gate: G2
Total Events: 71179

Marker	Left, Right	Events	% Gated	% Total
All	1, 9910	2498	100.00	3.51
M1	36, 9910	1508	60.37	2.12

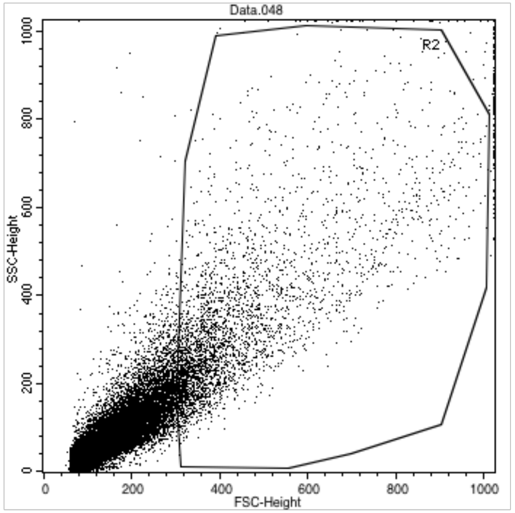

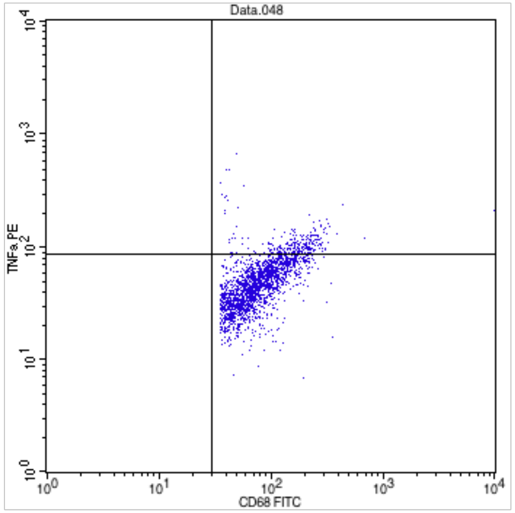

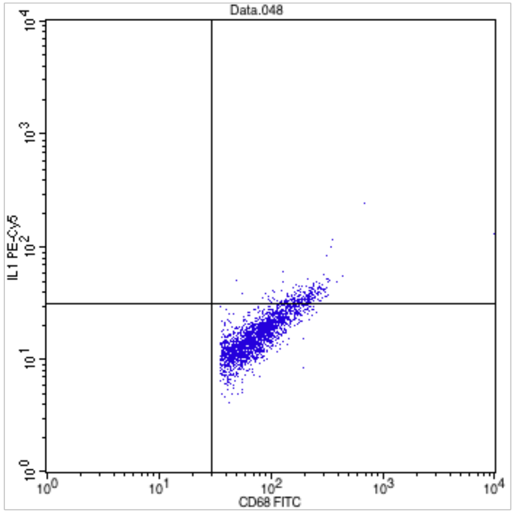

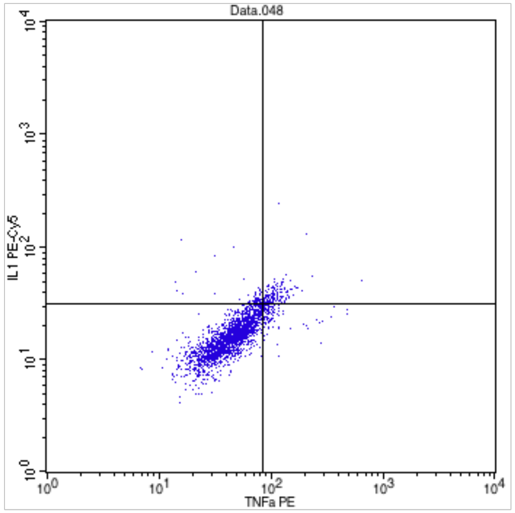

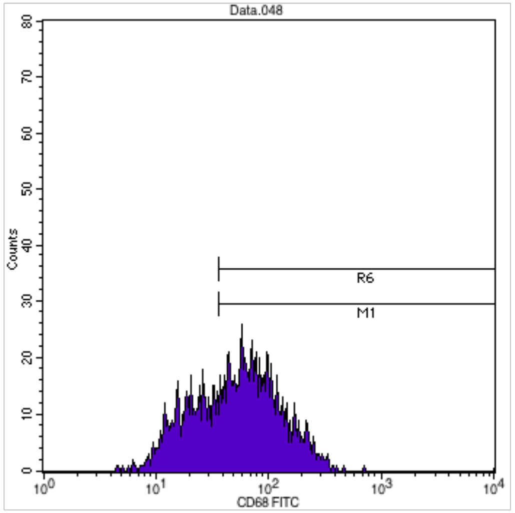

Quadrant Statistics

File: Data.048
Acquisition Date: 18-Oct-24
Gated Events: 2249
X Parameter: CD68 FITC (Log)
Quad Location: 30, 84

Sample ID: Norm.5
Gate: G7
Total Events: 66359
Y Parameter: TNFa PE (Log)

Quad	Events	% Gated	% Total
UL	0	0.00	0.00
UR	262	11.65	0.39
LL	0	0.00	0.00
LR	1987	88.35	2.99

Quadrant Statistics

File: Data.048
Acquisition Date: 18-Oct-24
Gated Events: 2249
X Parameter: CD68 FITC (Log)
Quad Location: 30, 31

Sample ID: Norm.5
Gate: G7
Total Events: 66359
Y Parameter: IL1 PE-Cy5 (Log)

Quad	Events	% Gated	% Total
UL	0	0.00	0.00
UR	210	9.34	0.32
LL	0	0.00	0.00
LR	2039	90.66	3.07

Quadrant Statistics

File: Data.048
Acquisition Date: 18-Oct-24
Gated Events: 2249
X Parameter: TNFa PE (Log)
Quad Location: 84, 31

Sample ID: Norm.5
Gate: G7
Total Events: 66359
Y Parameter: IL1 PE-Cy5 (Log)

Quad	Events	% Gated	% Total
UL	65	2.89	0.10
UR	145	6.45	0.22
LL	1922	85.46	2.90
LR	117	5.20	0.18

Histogram Statistics

File: Data.048
Acquisition Date: 18-Oct-24
Gated Events: 3408
X Parameter: CD68 FITC (Log)

Sample ID: Norm.5
Gate: G2
Total Events: 66359

Marker	Left, Right	Events	% Gated	% Total
All	1, 9910	3408	100.00	5.14
M1	36, 9910	2249	65.99	3.39

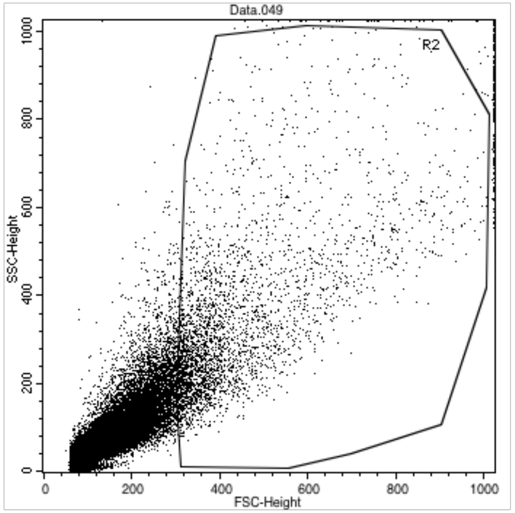

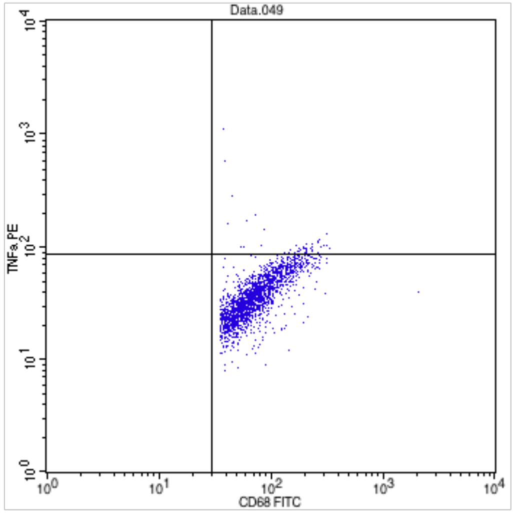

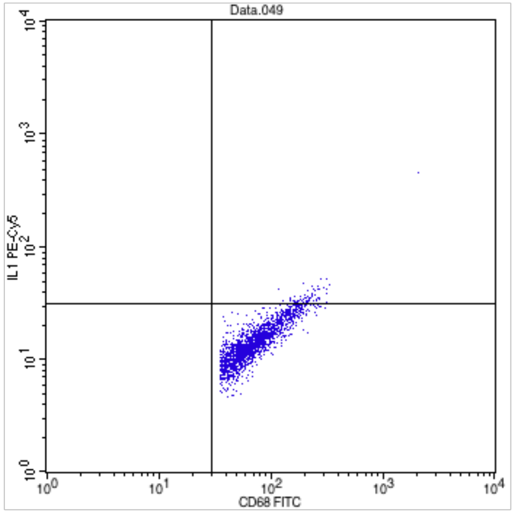

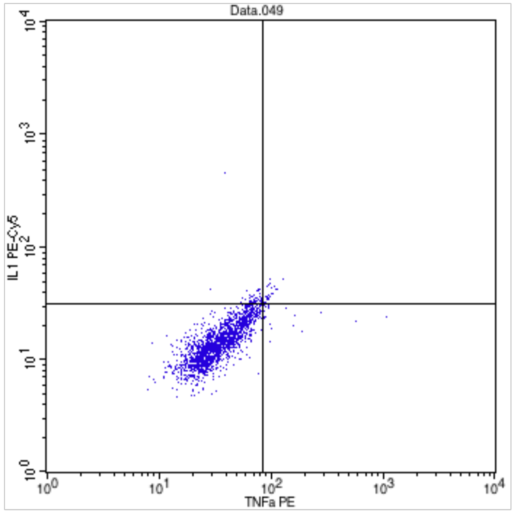

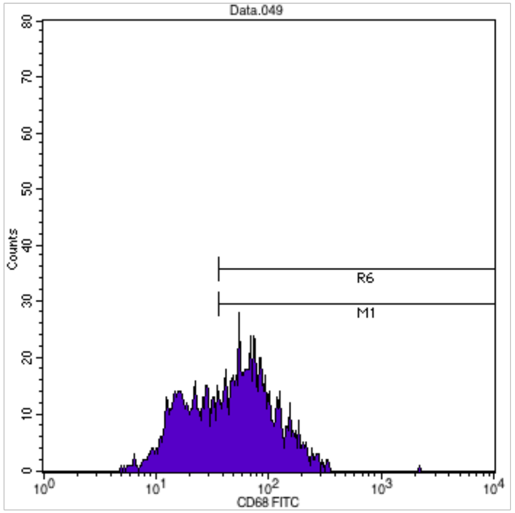

Quadrant Statistics

File: Data.049
Acquisition Date: 18-Oct-24
Gated Events: 2007
X Parameter: CD68 FITC (Log)
Quad Location: 30, 84

Sample ID: Norm.6
Gate: G7
Total Events: 61251
Y Parameter: TNFa PE (Log)

Quad	Events	% Gated	% Total
UL	0	0.00	0.00
UR	47	2.34	0.08
LL	0	0.00	0.00
LR	1960	97.66	3.20

Quadrant Statistics

File: Data.049
Acquisition Date: 18-Oct-24
Gated Events: 2007
X Parameter: CD68 FITC (Log)
Quad Location: 30, 31

Sample ID: Norm.6
Gate: G7
Total Events: 61251
Y Parameter: IL1 PE-Cy5 (Log)

Quad	Events	% Gated	% Total
UL	0	0.00	0.00
UR	72	3.59	0.12
LL	0	0.00	0.00
LR	1935	96.41	3.16

Quadrant Statistics

File: Data.049
Acquisition Date: 18-Oct-24
Gated Events: 2007
X Parameter: TNFa PE (Log)
Quad Location: 84, 31

Sample ID: Norm.6
Gate: G7
Total Events: 61251
Y Parameter: IL1 PE-Cy5 (Log)

Quad	Events	% Gated	% Total
UL	48	2.39	0.08
UR	24	1.20	0.04
LL	1912	95.27	3.12
LR	23	1.15	0.04

Histogram Statistics

File: Data.049
Acquisition Date: 18-Oct-24
Gated Events: 3274
X Parameter: CD68 FITC (Log)

Sample ID: Norm.6
Gate: G2
Total Events: 61251

Marker	Left, Right	Events	% Gated	% Total
All	1, 9910	3274	100.00	5.35
M1	36, 9910	2007	61.30	3.28

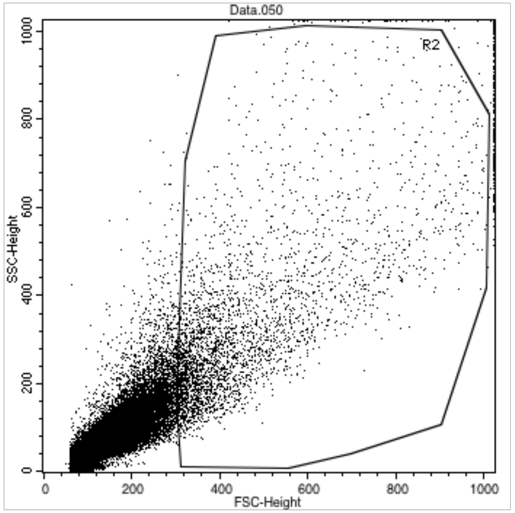

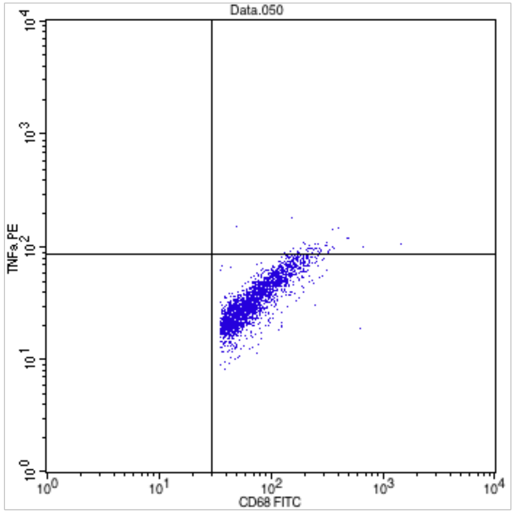

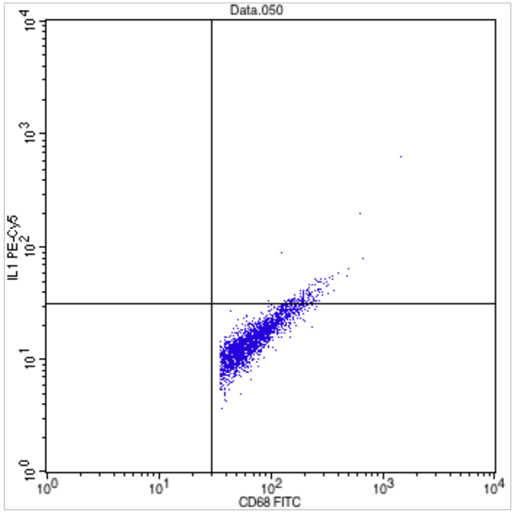

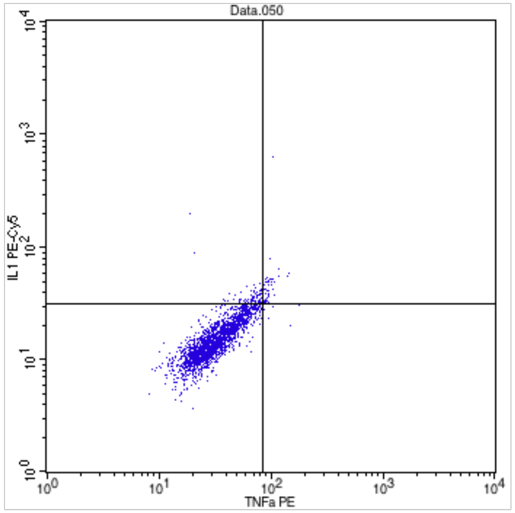

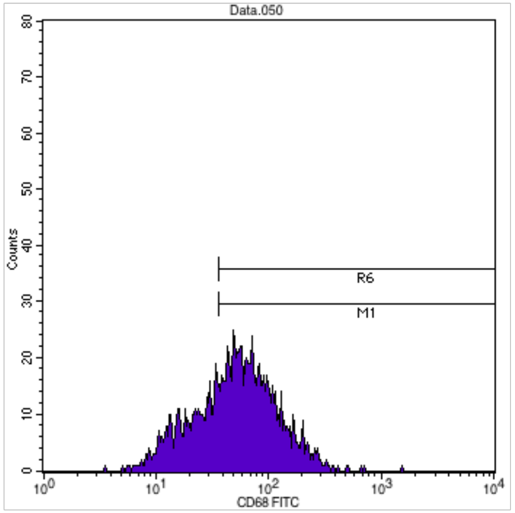

Quadrant Statistics

File: Data.050
Acquisition Date: 18-Oct-24
Gated Events: 2320
X Parameter: CD68 FITC (Log)
Quad Location: 30, 84
Sample ID: +.4
Gate: G7
Total Events: 62371
Y Parameter: TNFa PE (Log)

Quad	Events	% Gated	% Total
UL	0	0.00	0.00
UR	48	2.07	0.08
LL	0	0.00	0.00
LR	2272	97.93	3.64

Quadrant Statistics

File: Data.050
Acquisition Date: 18-Oct-24
Gated Events: 2320
X Parameter: CD68 FITC (Log)
Quad Location: 30, 31
Sample ID: +.4
Gate: G7
Total Events: 62371
Y Parameter: IL1 PE-Cy5 (Log)

Quad	Events	% Gated	% Total
UL	0	0.00	0.00
UR	130	5.60	0.21
LL	0	0.00	0.00
LR	2190	94.40	3.51

Quadrant Statistics

File: Data.050
Acquisition Date: 18-Oct-24
Gated Events: 2320
X Parameter: TNFa PE (Log)
Quad Location: 84, 31
Sample ID: +.4
Gate: G7
Total Events: 62371
Y Parameter: IL1 PE-Cy5 (Log)

Quad	Events	% Gated	% Total
UL	89	3.84	0.14
UR	41	1.77	0.07
LL	2183	94.09	3.50
LR	7	0.30	0.01

Histogram Statistics

File: Data.050
Acquisition Date: 18-Oct-24
Gated Events: 3330
X Parameter: CD68 FITC (Log)
Sample ID: +.4
Gate: G2
Total Events: 62371

Marker	Left, Right	Events	% Gated	% Total
All	1, 9910	3330	100.00	5.34
M1	36, 9910	2320	69.67	3.72

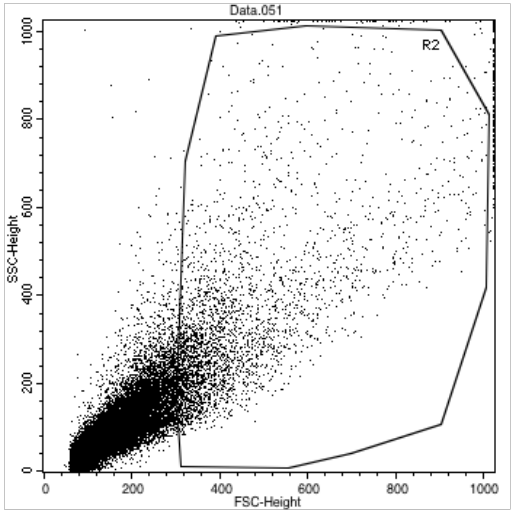

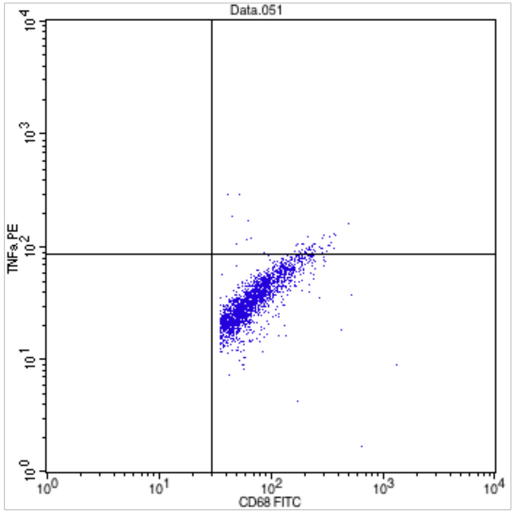

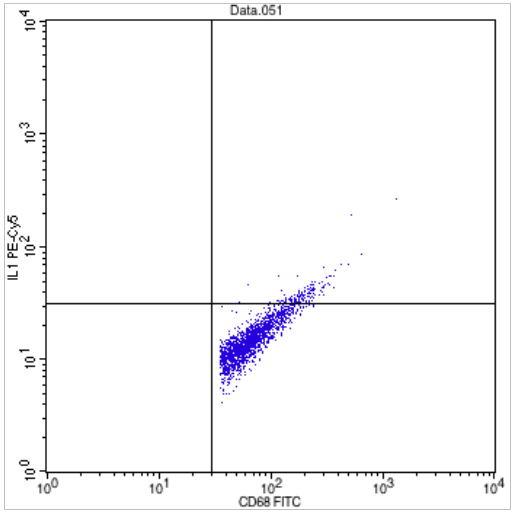

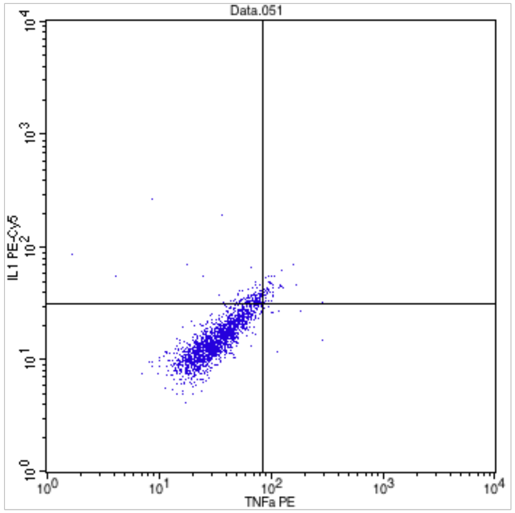

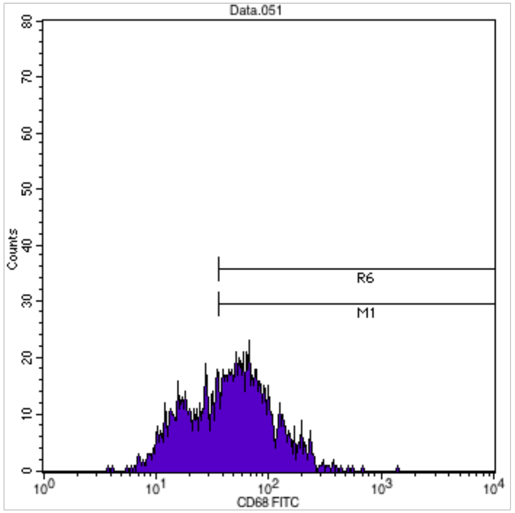

Quadrant Statistics

File: Data.051
Acquisition Date: 18-Oct-24
Gated Events: 2005
X Parameter: CD68 FITC (Log)
Quad Location: 30, 84

Sample ID: +.5
Gate: G7
Total Events: 62125
Y Parameter: TNFa PE (Log)

Quad	Events	% Gated	% Total
UL	0	0.00	0.00
UR	54	2.69	0.09
LL	0	0.00	0.00
LR	1951	97.31	3.14

Quadrant Statistics

File: Data.051
Acquisition Date: 18-Oct-24
Gated Events: 2005
X Parameter: CD68 FITC (Log)
Quad Location: 30, 31

Sample ID: +.5
Gate: G7
Total Events: 62125
Y Parameter: IL1 PE-Cy5 (Log)

Quad	Events	% Gated	% Total
UL	0	0.00	0.00
UR	148	7.38	0.24
LL	0	0.00	0.00
LR	1857	92.62	2.99

Quadrant Statistics

File: Data.051
Acquisition Date: 18-Oct-24
Gated Events: 2005
X Parameter: TNFa PE (Log)
Quad Location: 84, 31

Sample ID: +.5
Gate: G7
Total Events: 62125
Y Parameter: IL1 PE-Cy5 (Log)

Quad	Events	% Gated	% Total
UL	103	5.14	0.17
UR	45	2.24	0.07
LL	1848	92.17	2.97
LR	9	0.45	0.01

Histogram Statistics

File: Data.051
Acquisition Date: 18-Oct-24
Gated Events: 3228
X Parameter: CD68 FITC (Log)

Sample ID: +.5
Gate: G2
Total Events: 62125

Marker	Left, Right	Events	% Gated	% Total
All	1, 9910	3228	100.00	5.20
M1	36, 9910	2005	62.11	3.23

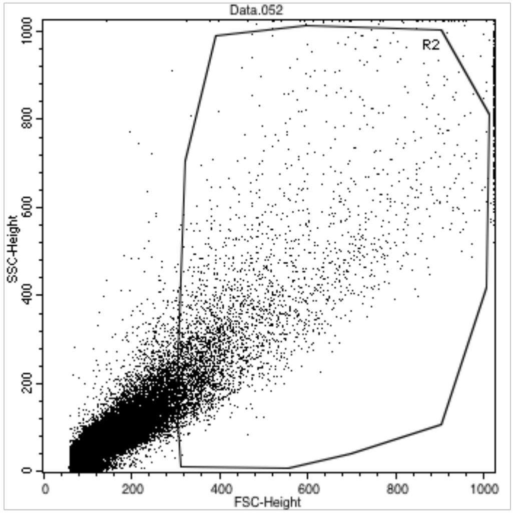

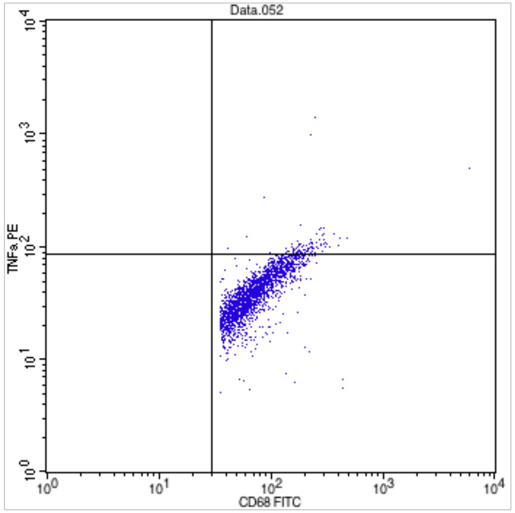

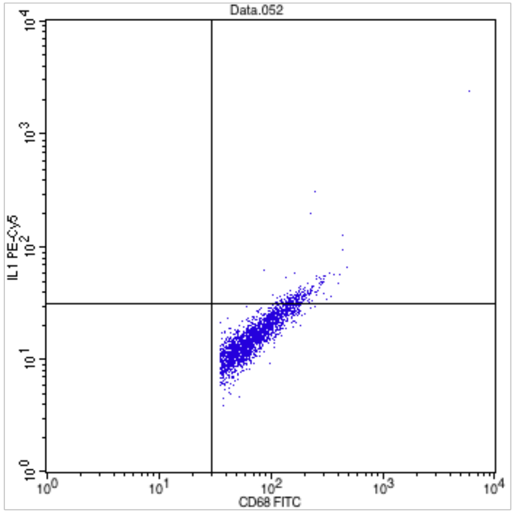

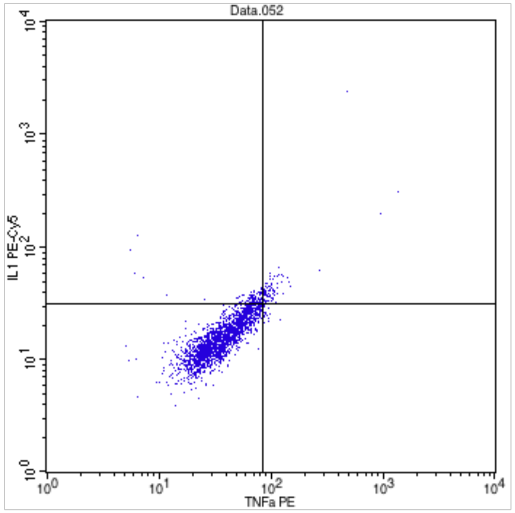

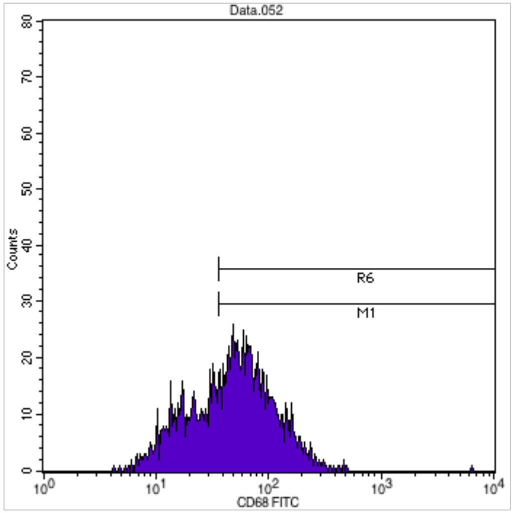

Quadrant Statistics

File: Data.052
Acquisition Date: 18-Oct-24
Gated Events: 2237
X Parameter: CD68 FITC (Log)
Quad Location: 30, 84

Sample ID: +.6
Gate: G7
Total Events: 59300
Y Parameter: TNFa PE (Log)

Quad	Events	% Gated	% Total
UL	0	0.00	0.00
UR	102	4.56	0.17
LL	0	0.00	0.00
LR	2135	95.44	3.60

Quadrant Statistics

File: Data.052
Acquisition Date: 18-Oct-24
Gated Events: 2237
X Parameter: CD68 FITC (Log)
Quad Location: 30, 31

Sample ID: +.6
Gate: G7
Total Events: 59300
Y Parameter: IL1 PE-Cy5 (Log)

Quad	Events	% Gated	% Total
UL	0	0.00	0.00
UR	176	7.87	0.30
LL	0	0.00	0.00
LR	2061	92.13	3.48

Quadrant Statistics

File: Data.052
Acquisition Date: 18-Oct-24
Gated Events: 2237
X Parameter: TNFa PE (Log)
Quad Location: 84, 31

Sample ID: +.6
Gate: G7
Total Events: 59300
Y Parameter: IL1 PE-Cy5 (Log)

Quad	Events	% Gated	% Total
UL	94	4.20	0.16
UR	82	3.67	0.14
LL	2041	91.24	3.44
LR	20	0.89	0.03

Histogram Statistics

File: Data.052
Acquisition Date: 18-Oct-24
Gated Events: 3480
X Parameter: CD68 FITC (Log)

Sample ID: +.6
Gate: G2
Total Events: 59300

Marker	Left, Right	Events	% Gated	% Total
All	1, 9910	3480	100.00	5.87
M1	36, 9910	2237	64.28	3.77

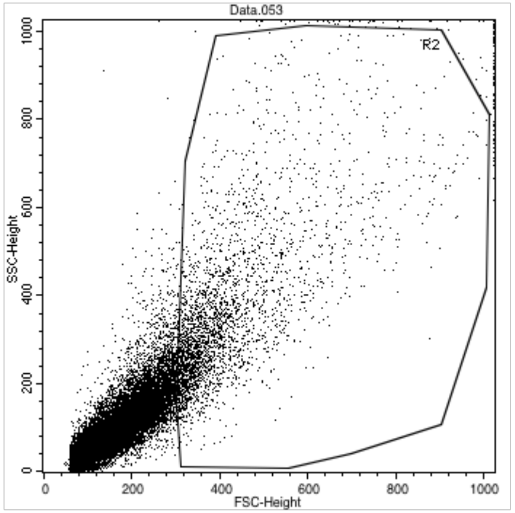

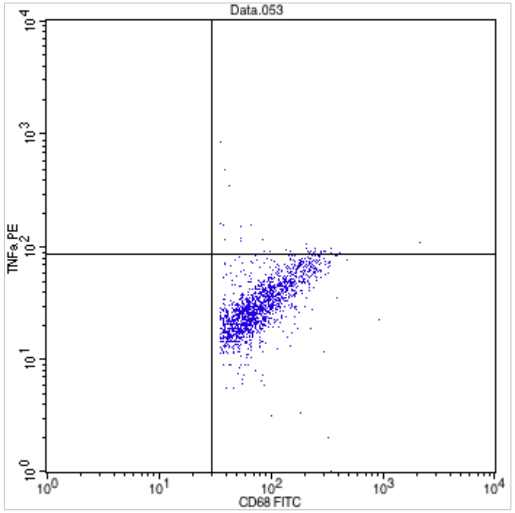

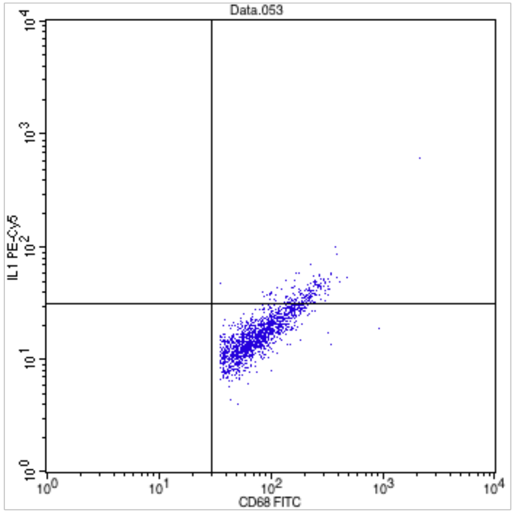

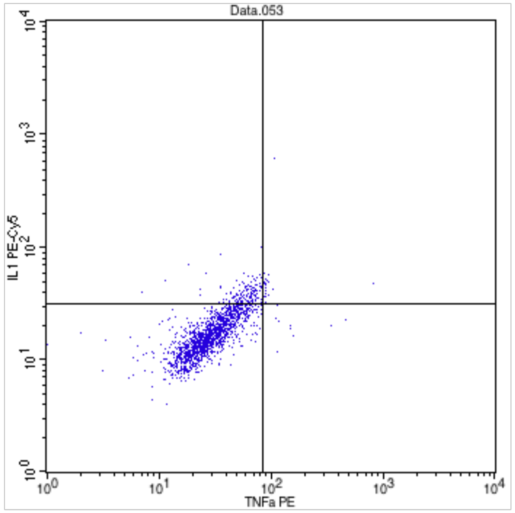

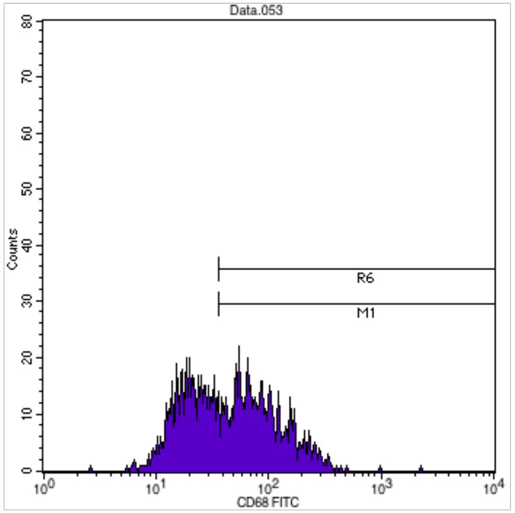

Quadrant Statistics

File: Data.053
Acquisition Date: 18-Oct-24
Gated Events: 1633
X Parameter: CD68 FITC (Log)
Quad Location: 30, 84

Sample ID: -.6
Gate: G7
Total Events: 68433
Y Parameter: TNFa PE (Log)

Quad	Events	% Gated	% Total
UL	0	0.00	0.00
UR	32	1.96	0.05
LL	0	0.00	0.00
LR	1601	98.04	2.34

Quadrant Statistics

File: Data.053
Acquisition Date: 18-Oct-24
Gated Events: 1633
X Parameter: CD68 FITC (Log)
Quad Location: 30, 31

Sample ID: -.6
Gate: G7
Total Events: 68433
Y Parameter: IL1 PE-Cy5 (Log)

Quad	Events	% Gated	% Total
UL	0	0.00	0.00
UR	172	10.53	0.25
LL	0	0.00	0.00
LR	1461	89.47	2.13

Quadrant Statistics

File: Data.053
Acquisition Date: 18-Oct-24
Gated Events: 1633
X Parameter: TNFa PE (Log)
Quad Location: 84, 31

Sample ID: -.6
Gate: G7
Total Events: 68433
Y Parameter: IL1 PE-Cy5 (Log)

Quad	Events	% Gated	% Total
UL	150	9.19	0.22
UR	22	1.35	0.03
LL	1451	88.85	2.12
LR	10	0.61	0.01

Histogram Statistics

File: Data.053
Acquisition Date: 18-Oct-24
Gated Events: 3003
X Parameter: CD68 FITC (Log)

Sample ID: -.6
Gate: G2
Total Events: 68433

Marker	Left, Right	Events	% Gated	% Total
All	1, 9910	3003	100.00	4.39
M1	36, 9910	1633	54.38	2.39

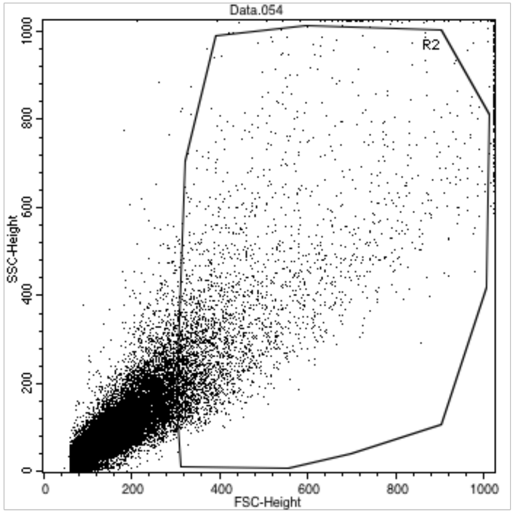

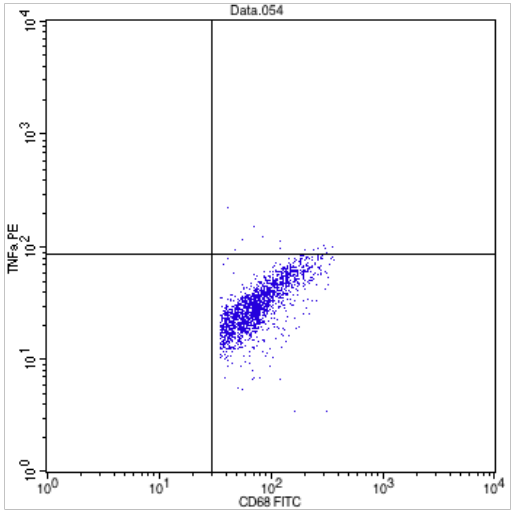

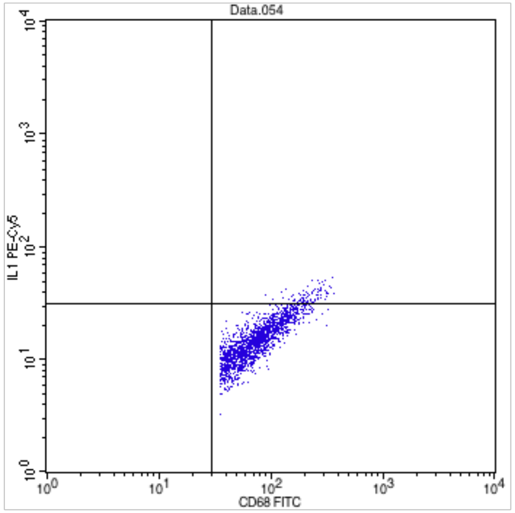

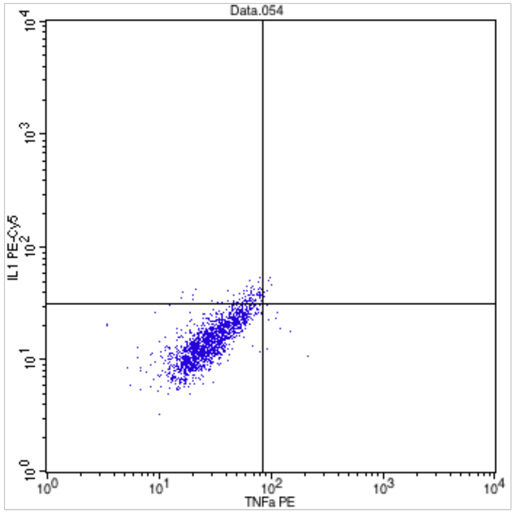

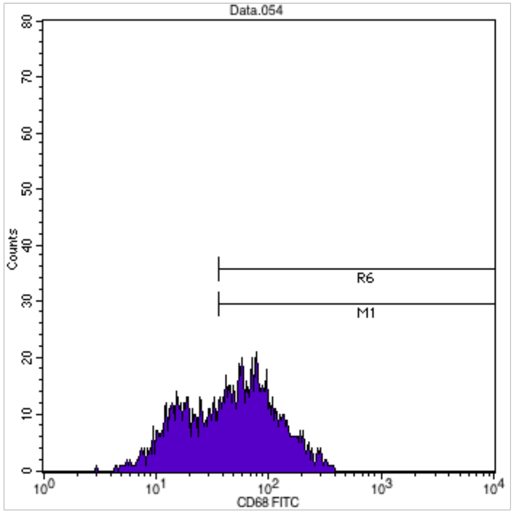

Quadrant Statistics

File: Data.054
Acquisition Date: 18-Oct-24
Gated Events: 1894
X Parameter: CD68 FITC (Log)
Quad Location: 30, 84

Sample ID: 1.4
Gate: G7
Total Events: 63497
Y Parameter: TNFa PE (Log)

Quad	Events	% Gated	% Total
UL	0	0.00	0.00
UR	15	0.79	0.02
LL	0	0.00	0.00
LR	1879	99.21	2.96

Quadrant Statistics

File: Data.054
Acquisition Date: 18-Oct-24
Gated Events: 1894
X Parameter: CD68 FITC (Log)
Quad Location: 30, 31

Sample ID: 1.4
Gate: G7
Total Events: 63497
Y Parameter: IL1 PE-Cy5 (Log)

Quad	Events	% Gated	% Total
UL	0	0.00	0.00
UR	97	5.12	0.15
LL	0	0.00	0.00
LR	1797	94.88	2.83

Quadrant Statistics

File: Data.054
Acquisition Date: 18-Oct-24
Gated Events: 1894
X Parameter: TNFa PE (Log)
Quad Location: 84, 31

Sample ID: 1.4
Gate: G7
Total Events: 63497
Y Parameter: IL1 PE-Cy5 (Log)

Quad	Events	% Gated	% Total
UL	90	4.75	0.14
UR	7	0.37	0.01
LL	1789	94.46	2.82
LR	8	0.42	0.01

Histogram Statistics

File: Data.054
Acquisition Date: 18-Oct-24
Gated Events: 3082
X Parameter: CD68 FITC (Log)

Sample ID: 1.4
Gate: G2
Total Events: 63497

Marker	Left, Right	Events	% Gated	% Total
All	1, 9910	3082	100.00	4.85
M1	36, 9910	1894	61.45	2.98

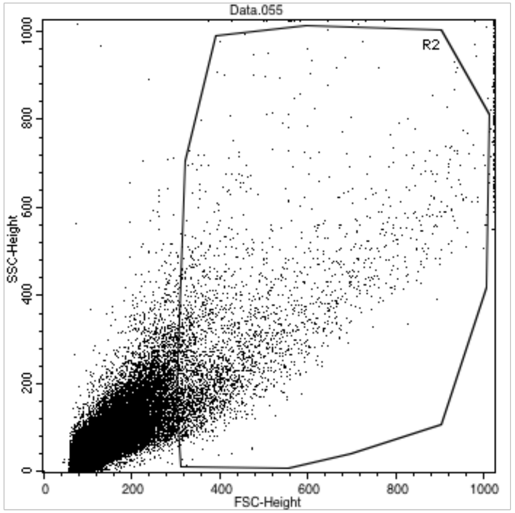

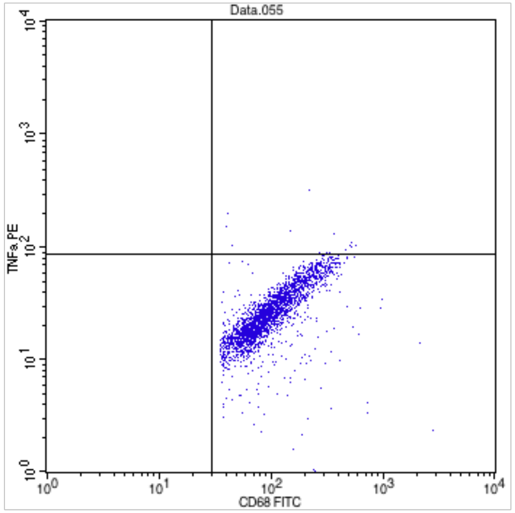

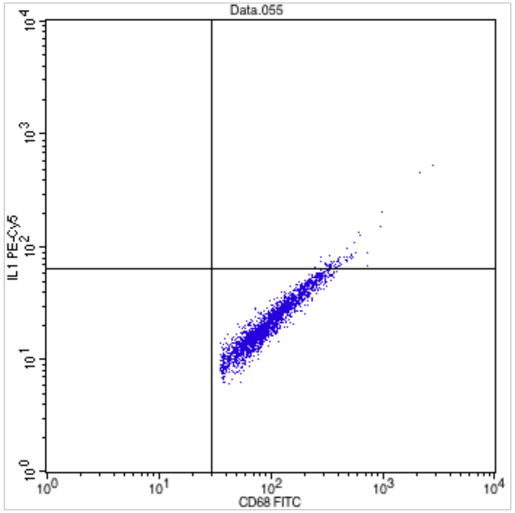

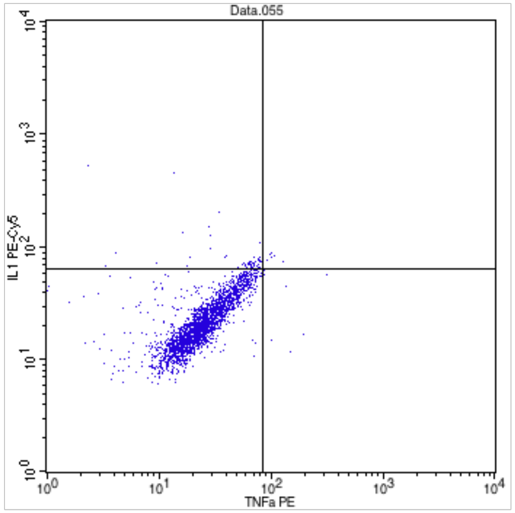

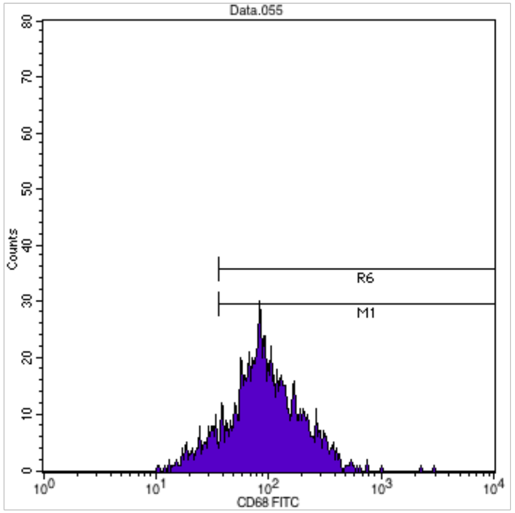

Quadrant Statistics

File: Data.055
Acquisition Date: 18-Oct-24
Gated Events: 2593
X Parameter: CD68 FITC (Log)
Quad Location: 30, 84

Sample ID: 1.5
Gate: G7
Total Events: 73188
Y Parameter: TNFa PE (Log)

Quad	Events	% Gated	% Total
UL	0	0.00	0.00
UR	14	0.54	0.02
LL	0	0.00	0.00
LR	2579	99.46	3.52

Quadrant Statistics

File: Data.055
Acquisition Date: 18-Oct-24
Gated Events: 2593
X Parameter: CD68 FITC (Log)
Quad Location: 30, 63

Sample ID: 1.5
Gate: G7
Total Events: 73188
Y Parameter: IL1 PE-Cy5 (Log)

Quad	Events	% Gated	% Total
UL	0	0.00	0.00
UR	64	2.47	0.09
LL	0	0.00	0.00
LR	2529	97.53	3.46

Quadrant Statistics

File: Data.055
Acquisition Date: 18-Oct-24
Gated Events: 2593
X Parameter: TNFa PE (Log)
Quad Location: 84, 63

Sample ID: 1.5
Gate: G7
Total Events: 73188
Y Parameter: IL1 PE-Cy5 (Log)

Quad	Events	% Gated	% Total
UL	58	2.24	0.08
UR	6	0.23	0.01
LL	2521	97.22	3.44
LR	8	0.31	0.01

Histogram Statistics

File: Data.055
Acquisition Date: 18-Oct-24
Gated Events: 2901
X Parameter: CD68 FITC (Log)

Sample ID: 1.5
Gate: G2
Total Events: 73188

Marker	Left, Right	Events	% Gated	% Total
All	1, 9910	2901	100.00	3.96
M1	36, 9910	2593	89.38	3.54

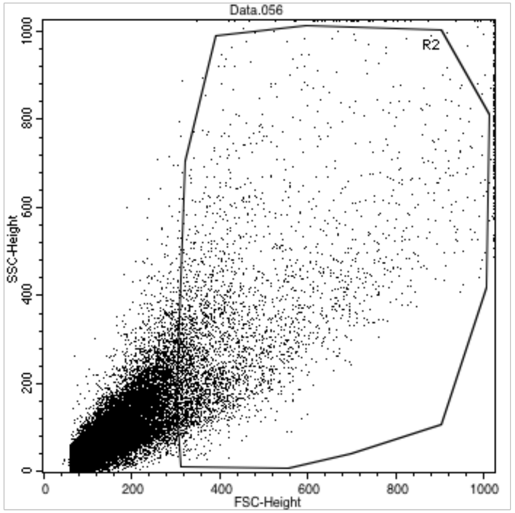

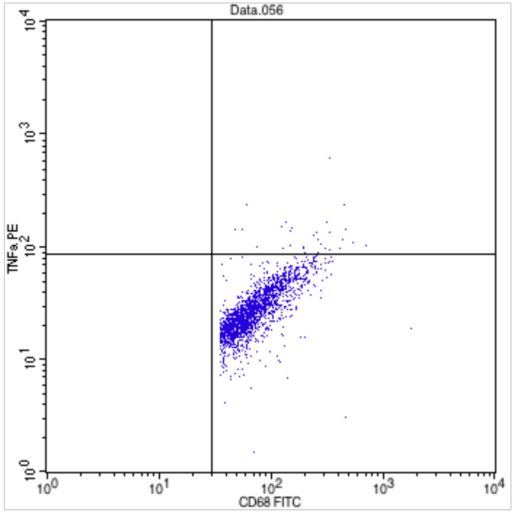

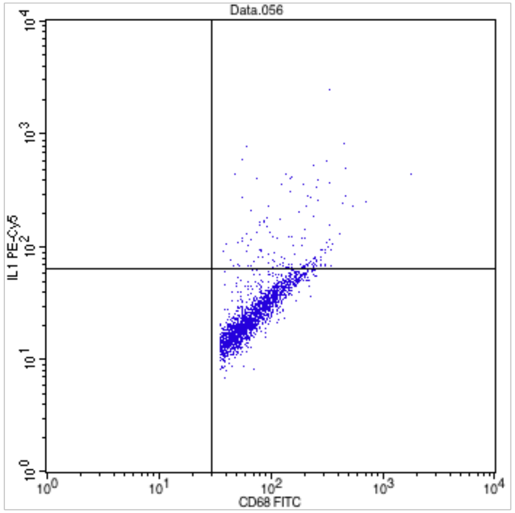

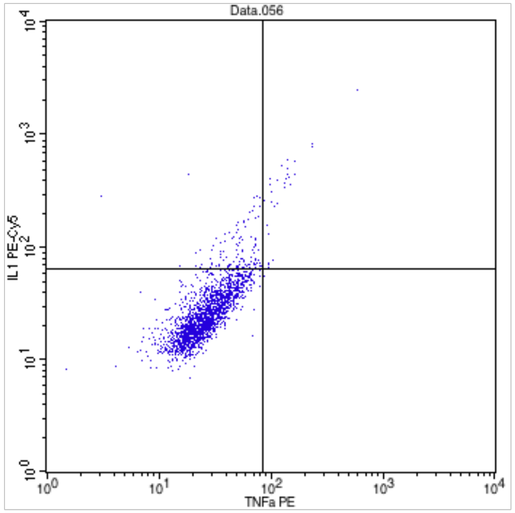

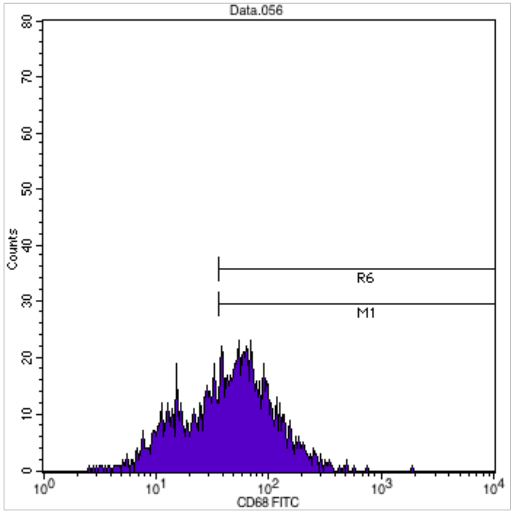

Quadrant Statistics

File: Data.056
Acquisition Date: 18-Oct-24
Gated Events: 2062
X Parameter: CD68 FITC (Log)
Quad Location: 30, 84
Sample ID: 1.7
Gate: G7
Total Events: 57846
Y Parameter: TNFa PE (Log)

Quad	Events	% Gated	% Total
UL	0	0.00	0.00
UR	29	1.41	0.05
LL	0	0.00	0.00
LR	2033	98.59	3.51

Quadrant Statistics

File: Data.056
Acquisition Date: 18-Oct-24
Gated Events: 2062
X Parameter: CD68 FITC (Log)
Quad Location: 30, 63
Sample ID: 1.7
Gate: G7
Total Events: 57846
Y Parameter: IL1 PE-Cy5 (Log)

Quad	Events	% Gated	% Total
UL	0	0.00	0.00
UR	135	6.55	0.23
LL	0	0.00	0.00
LR	1927	93.45	3.33

Quadrant Statistics

File: Data.056
Acquisition Date: 18-Oct-24
Gated Events: 2062
X Parameter: TNFa PE (Log)
Quad Location: 84, 63
Sample ID: 1.7
Gate: G7
Total Events: 57846
Y Parameter: IL1 PE-Cy5 (Log)

Quad	Events	% Gated	% Total
UL	107	5.19	0.18
UR	28	1.36	0.05
LL	1926	93.40	3.33
LR	1	0.05	0.00

Histogram Statistics

File: Data.056
Acquisition Date: 18-Oct-24
Gated Events: 3347
X Parameter: CD68 FITC (Log)
Sample ID: 1.7
Gate: G2
Total Events: 57846

Marker	Left, Right	Events	% Gated	% Total
All	1, 9910	3347	100.00	5.79
M1	36, 9910	2062	61.61	3.56

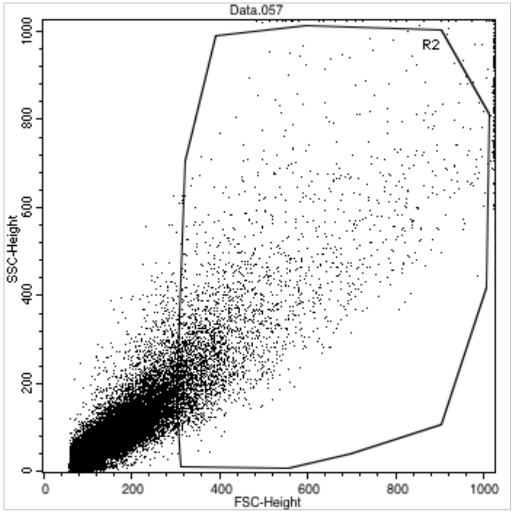

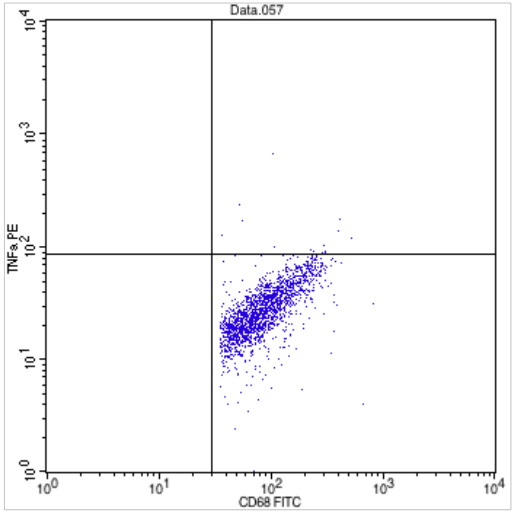

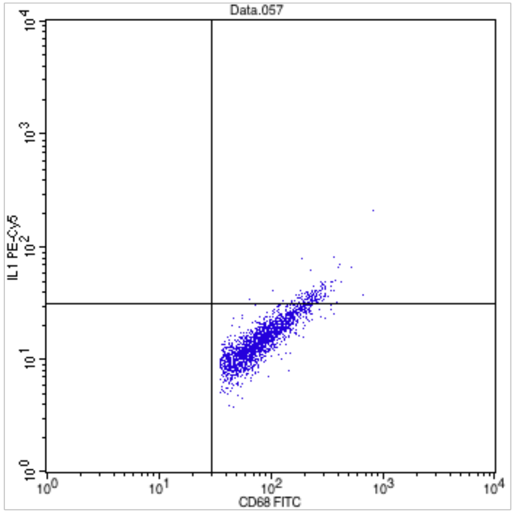

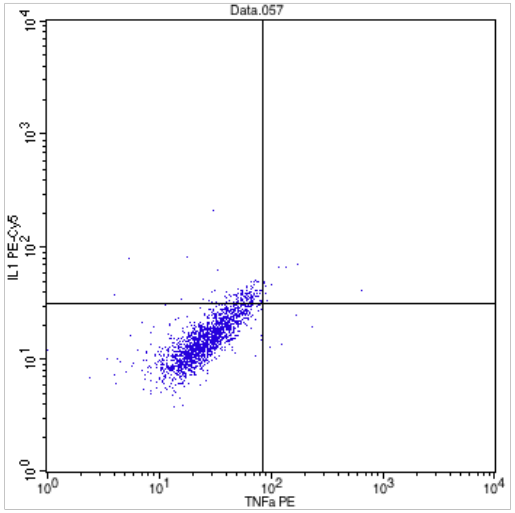

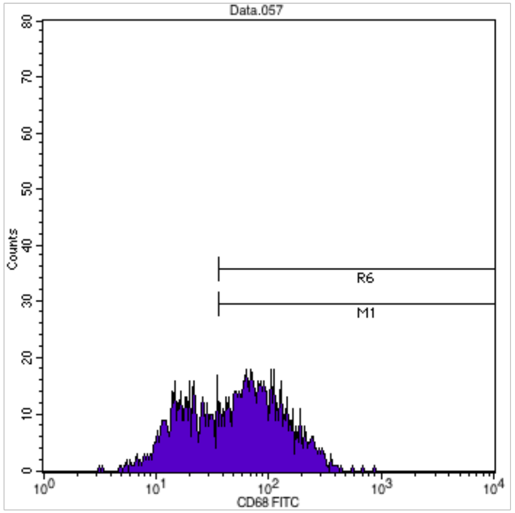

Quadrant Statistics

File: Data.057 Sample ID: 2.4
Acquisition Date: 18-Oct-24 Gate: G7
Gated Events: 1921 Total Events: 67515
X Parameter: CD68 FITC (Log) Y Parameter: TNFa PE (Log)
Quad Location: 30, 84

Quad	Events	% Gated	% Total
UL	0	0.00	0.00
UR	15	0.78	0.02
LL	0	0.00	0.00
LR	1906	99.22	2.82

Quadrant Statistics

File: Data.057 Sample ID: 2.4
Acquisition Date: 18-Oct-24 Gate: G7
Gated Events: 1921 Total Events: 67515
X Parameter: CD68 FITC (Log) Y Parameter: IL1 PE-Cy5 (Log)
Quad Location: 30, 31

Quad	Events	% Gated	% Total
UL	0	0.00	0.00
UR	134	6.98	0.20
LL	0	0.00	0.00
LR	1787	93.02	2.65

Quadrant Statistics

File: Data.057 Sample ID: 2.4
Acquisition Date: 18-Oct-24 Gate: G7
Gated Events: 1921 Total Events: 67515
X Parameter: TNFa PE (Log) Y Parameter: IL1 PE-Cy5 (Log)
Quad Location: 84, 31

Quad	Events	% Gated	% Total
UL	124	6.45	0.18
UR	10	0.52	0.01
LL	1782	92.76	2.64
LR	5	0.26	0.01

Histogram Statistics

File: Data.057 Sample ID: 2.4
Acquisition Date: 18-Oct-24 Gate: G2
Gated Events: 3086 Total Events: 67515
X Parameter: CD68 FITC (Log)

Marker	Left, Right	Events	% Gated	% Total
All	1, 9910	3086	100.00	4.57
M1	36, 9910	1921	62.25	2.85

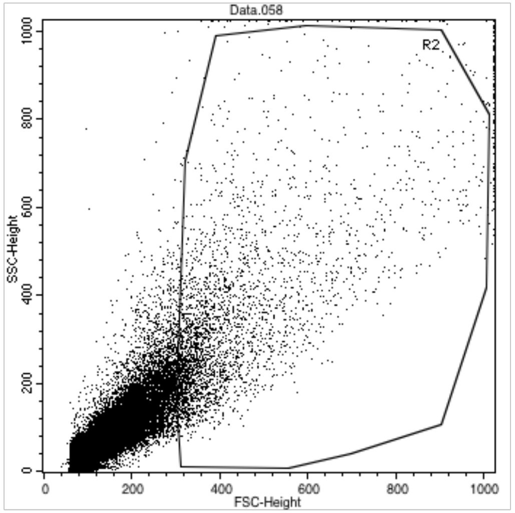

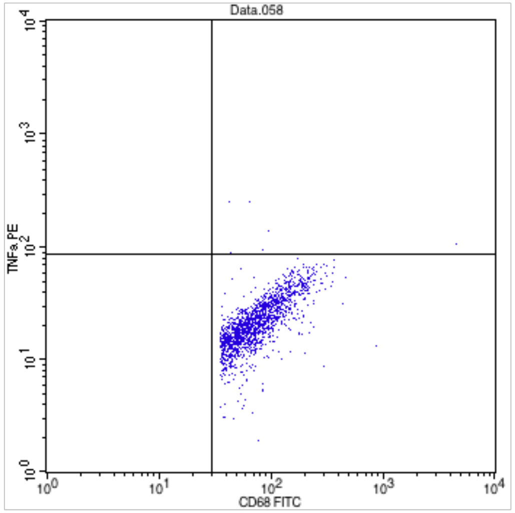

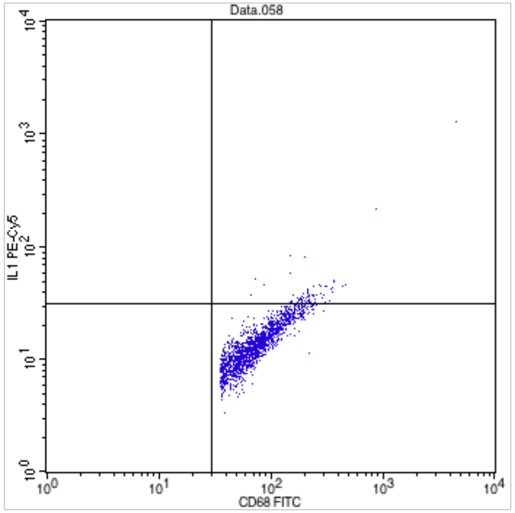

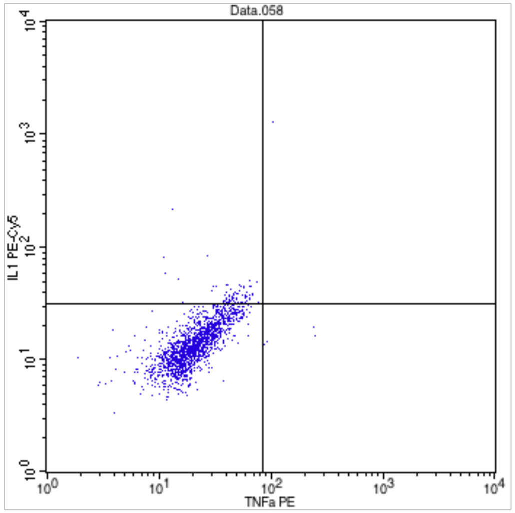

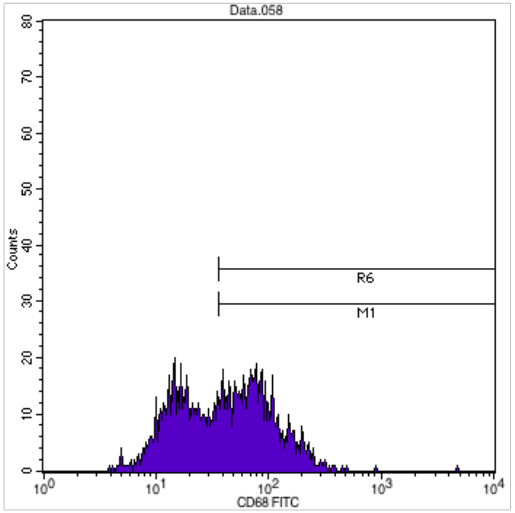

Quadrant Statistics

File: Data.058
Acquisition Date: 18-Oct-24
Gated Events: 1716
X Parameter: CD68 FITC (Log)
Quad Location: 30, 84
Sample ID: 2.5
Gate: G7
Total Events: 63844
Y Parameter: TNFa PE (Log)

Quad	Events	% Gated	% Total
UL	0	0.00	0.00
UR	6	0.35	0.01
LL	0	0.00	0.00
LR	1710	99.65	2.68

Quadrant Statistics

File: Data.058
Acquisition Date: 18-Oct-24
Gated Events: 1716
X Parameter: CD68 FITC (Log)
Quad Location: 30, 31
Sample ID: 2.5
Gate: G7
Total Events: 63844
Y Parameter: IL1 PE-Cy5 (Log)

Quad	Events	% Gated	% Total
UL	0	0.00	0.00
UR	77	4.49	0.12
LL	0	0.00	0.00
LR	1639	95.51	2.57

Quadrant Statistics

File: Data.058
Acquisition Date: 18-Oct-24
Gated Events: 1716
X Parameter: TNFa PE (Log)
Quad Location: 84, 31
Sample ID: 2.5
Gate: G7
Total Events: 63844
Y Parameter: IL1 PE-Cy5 (Log)

Quad	Events	% Gated	% Total
UL	76	4.43	0.12
UR	1	0.06	0.00
LL	1634	95.22	2.56
LR	5	0.29	0.01

Histogram Statistics

File: Data.058
Acquisition Date: 18-Oct-24
Gated Events: 3128
X Parameter: CD68 FITC (Log)
Sample ID: 2.5
Gate: G2
Total Events: 63844

Marker	Left, Right	Events	% Gated	% Total
All	1, 9910	3128	100.00	4.90
M1	36, 9910	1716	54.86	2.69

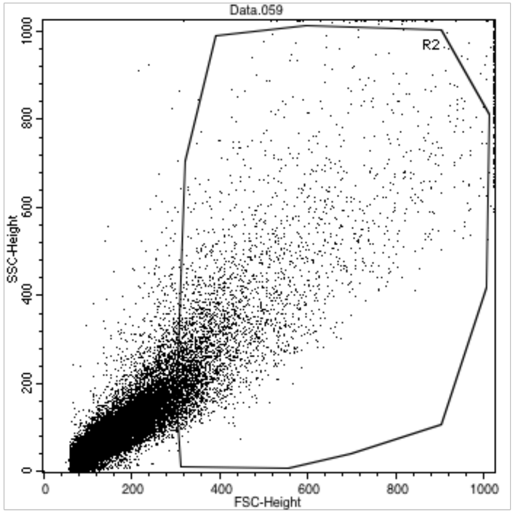

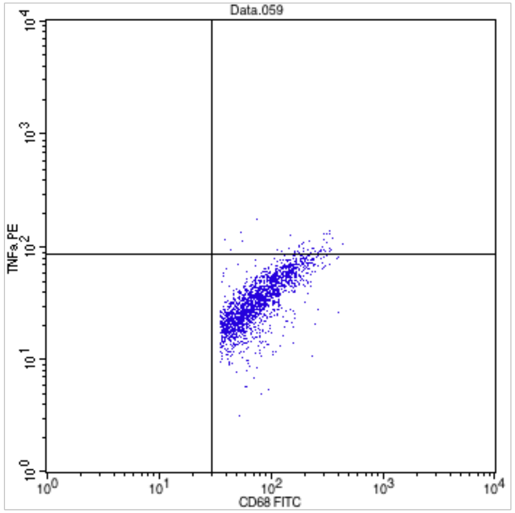

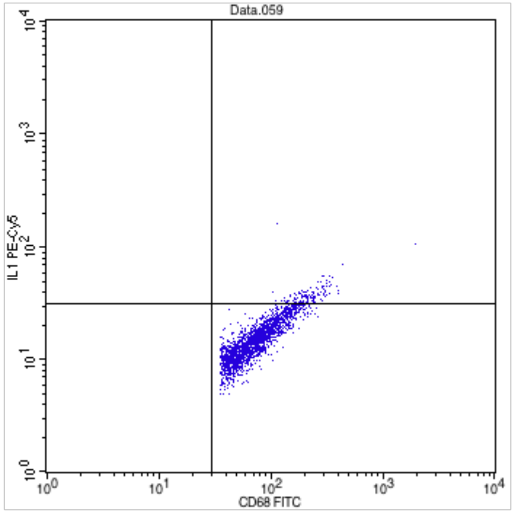

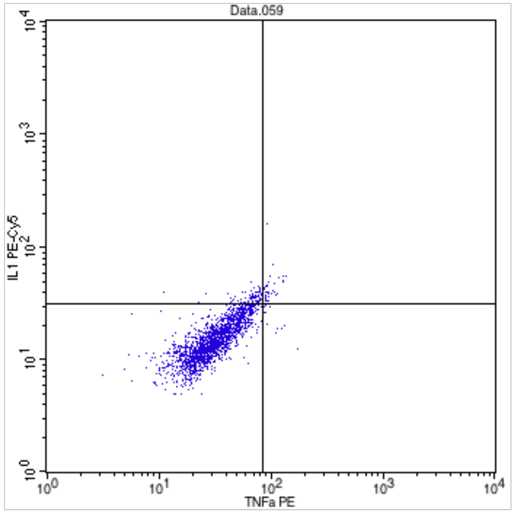

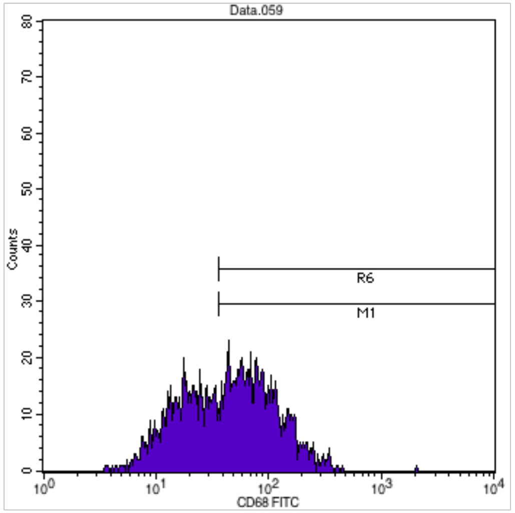

Quadrant Statistics

File: Data.059
Acquisition Date: 18-Oct-24
Gated Events: 2080
X Parameter: CD68 FITC (Log)
Quad Location: 30, 84
Sample ID: 2.6
Gate: G7
Total Events: 57212
Y Parameter: TNFa PE (Log)

Quad	Events	% Gated	% Total
UL	0	0.00	0.00
UR	48	2.31	0.08
LL	0	0.00	0.00
LR	2032	97.69	3.55

Quadrant Statistics

File: Data.059
Acquisition Date: 18-Oct-24
Gated Events: 2080
X Parameter: CD68 FITC (Log)
Quad Location: 30, 31
Sample ID: 2.6
Gate: G7
Total Events: 57212
Y Parameter: IL1 PE-Cy5 (Log)

Quad	Events	% Gated	% Total
UL	0	0.00	0.00
UR	131	6.30	0.23
LL	0	0.00	0.00
LR	1949	93.70	3.41

Quadrant Statistics

File: Data.059
Acquisition Date: 18-Oct-24
Gated Events: 2080
X Parameter: TNFa PE (Log)
Quad Location: 84, 31
Sample ID: 2.6
Gate: G7
Total Events: 57212
Y Parameter: IL1 PE-Cy5 (Log)

Quad	Events	% Gated	% Total
UL	95	4.57	0.17
UR	36	1.73	0.06
LL	1937	93.12	3.39
LR	12	0.58	0.02

Histogram Statistics

File: Data.059
Acquisition Date: 18-Oct-24
Gated Events: 3569
X Parameter: CD68 FITC (Log)
Sample ID: 2.6
Gate: G2
Total Events: 57212

Marker	Left, Right	Events	% Gated	% Total
All	1, 9910	3569	100.00	6.24
M1	36, 9910	2080	58.28	3.64

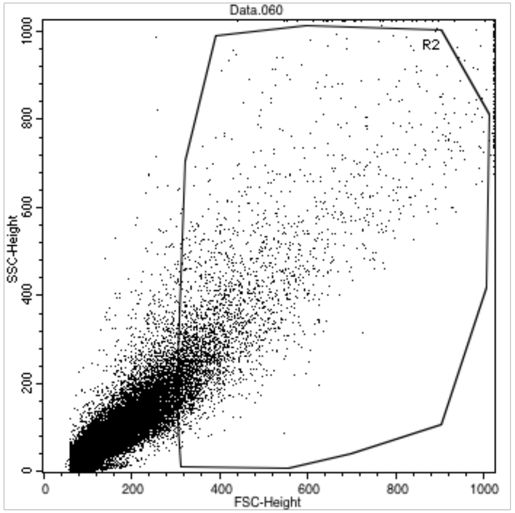

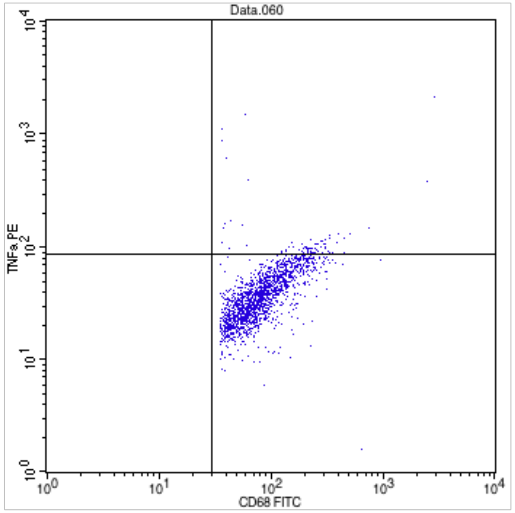

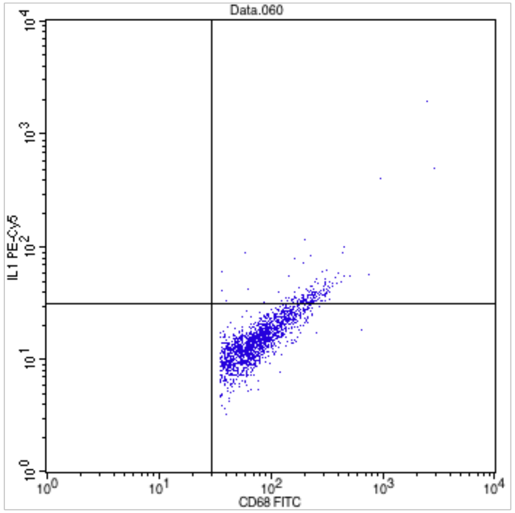

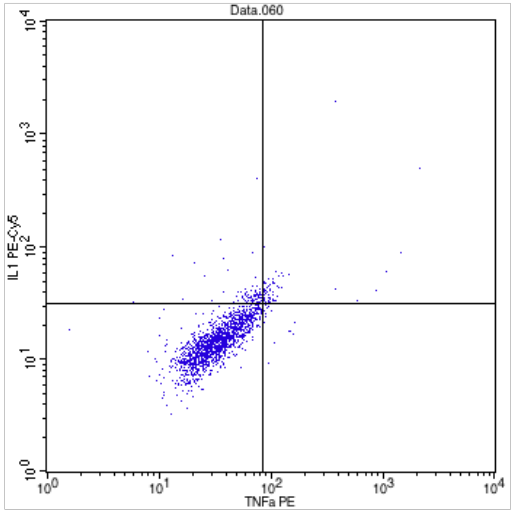

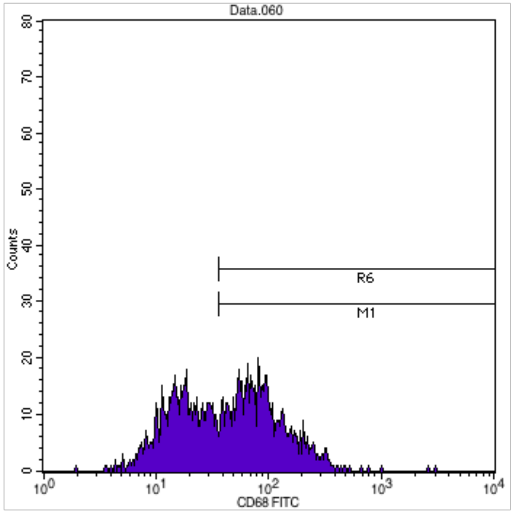

Quadrant Statistics

File: Data.060
Acquisition Date: 18-Oct-24
Gated Events: 1760
X Parameter: CD68 FITC (Log)
Quad Location: 30, 84

Sample ID: 3.4
Gate: G7
Total Events: 65346
Y Parameter: TNFa PE (Log)

Quad	Events	% Gated	% Total
UL	0	0.00	0.00
UR	79	4.49	0.12
LL	0	0.00	0.00
LR	1681	95.51	2.57

Quadrant Statistics

File: Data.060
Acquisition Date: 18-Oct-24
Gated Events: 1760
X Parameter: CD68 FITC (Log)
Quad Location: 30, 31

Sample ID: 3.4
Gate: G7
Total Events: 65346
Y Parameter: IL1 PE-Cy5 (Log)

Quad	Events	% Gated	% Total
UL	0	0.00	0.00
UR	155	8.81	0.24
LL	0	0.00	0.00
LR	1605	91.19	2.46

Quadrant Statistics

File: Data.060
Acquisition Date: 18-Oct-24
Gated Events: 1760
X Parameter: TNFa PE (Log)
Quad Location: 84, 31

Sample ID: 3.4
Gate: G7
Total Events: 65346
Y Parameter: IL1 PE-Cy5 (Log)

Quad	Events	% Gated	% Total
UL	93	5.28	0.14
UR	62	3.52	0.09
LL	1588	90.23	2.43
LR	17	0.97	0.03

Histogram Statistics

File: Data.060
Acquisition Date: 18-Oct-24
Gated Events: 3158
X Parameter: CD68 FITC (Log)

Sample ID: 3.4
Gate: G2
Total Events: 65346

Marker	Left, Right	Events	% Gated	% Total
All	1, 9910	3158	100.00	4.83
M1	36, 9910	1760	55.73	2.69

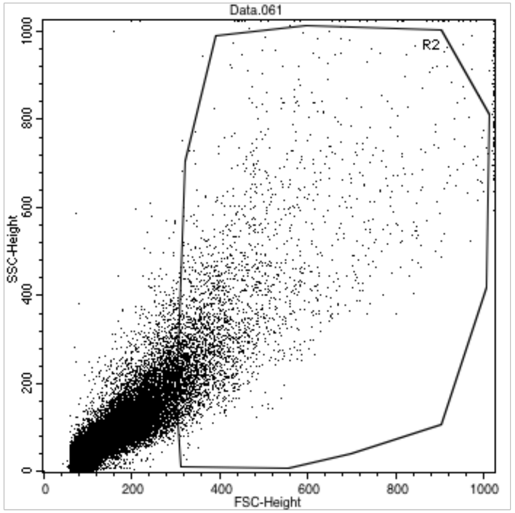

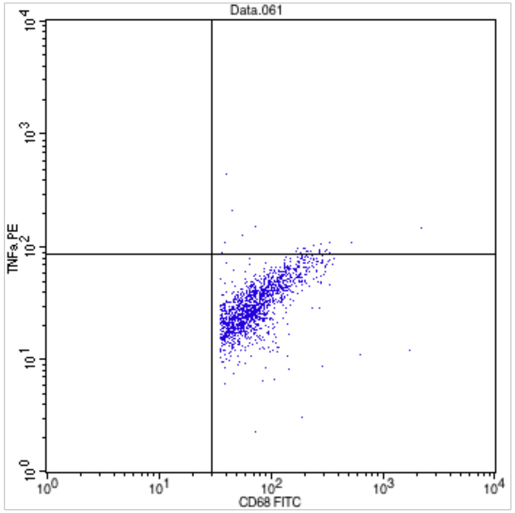

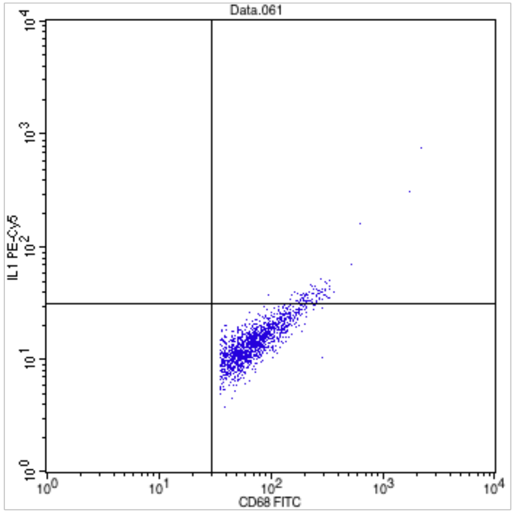

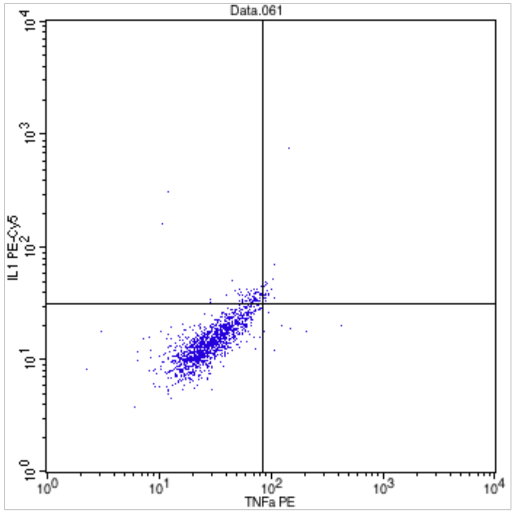

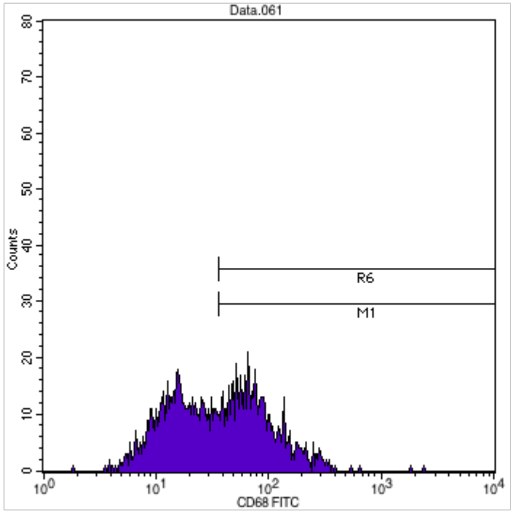

Quadrant Statistics

File: Data.061
Acquisition Date: 18-Oct-24
Gated Events: 1516
X Parameter: CD68 FITC (Log)
Quad Location: 30, 84

Sample ID: 3.5
Gate: G7
Total Events: 61579
Y Parameter: TNFa PE (Log)

Quad	Events	% Gated	% Total
UL	0	0.00	0.00
UR	25	1.65	0.04
LL	0	0.00	0.00
LR	1491	98.35	2.42

Quadrant Statistics

File: Data.061
Acquisition Date: 18-Oct-24
Gated Events: 1516
X Parameter: CD68 FITC (Log)
Quad Location: 30, 31

Sample ID: 3.5
Gate: G7
Total Events: 61579
Y Parameter: IL1 PE-Cy5 (Log)

Quad	Events	% Gated	% Total
UL	0	0.00	0.00
UR	85	5.61	0.14
LL	0	0.00	0.00
LR	1431	94.39	2.32

Quadrant Statistics

File: Data.061
Acquisition Date: 18-Oct-24
Gated Events: 1516
X Parameter: TNFa PE (Log)
Quad Location: 84, 31

Sample ID: 3.5
Gate: G7
Total Events: 61579
Y Parameter: IL1 PE-Cy5 (Log)

Quad	Events	% Gated	% Total
UL	67	4.42	0.11
UR	18	1.19	0.03
LL	1424	93.93	2.31
LR	7	0.46	0.01

Histogram Statistics

File: Data.061
Acquisition Date: 18-Oct-24
Gated Events: 3080
X Parameter: CD68 FITC (Log)

Sample ID: 3.5
Gate: G2
Total Events: 61579

Marker	Left, Right	Events	% Gated	% Total
All	1, 9910	3080	100.00	5.00
M1	36, 9910	1516	49.22	2.46

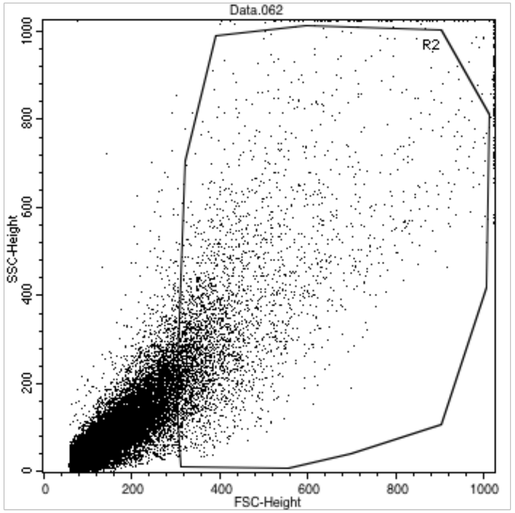

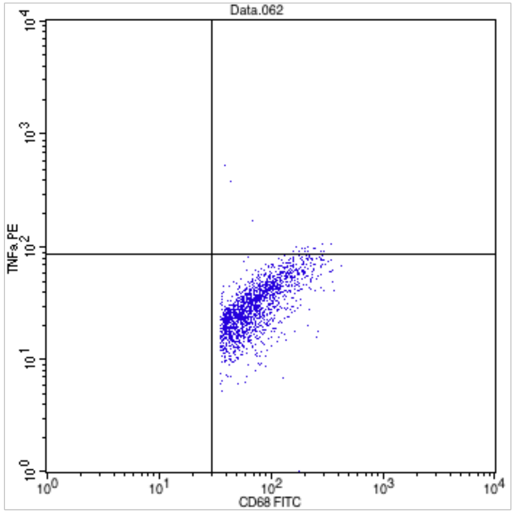

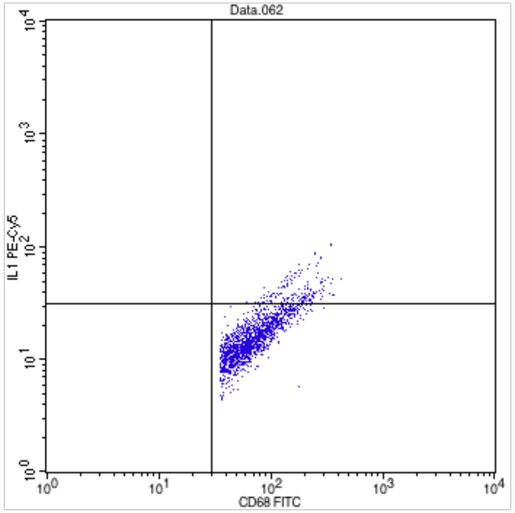

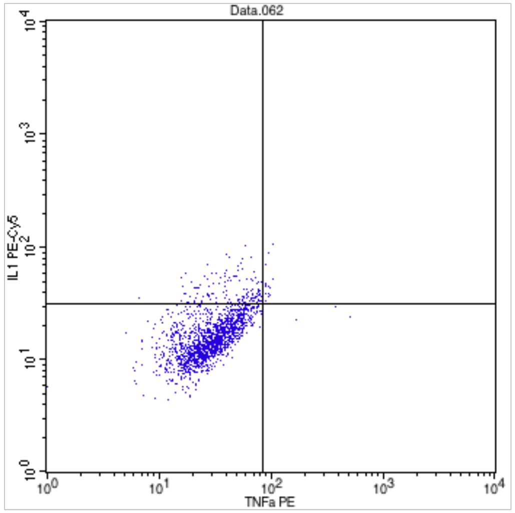

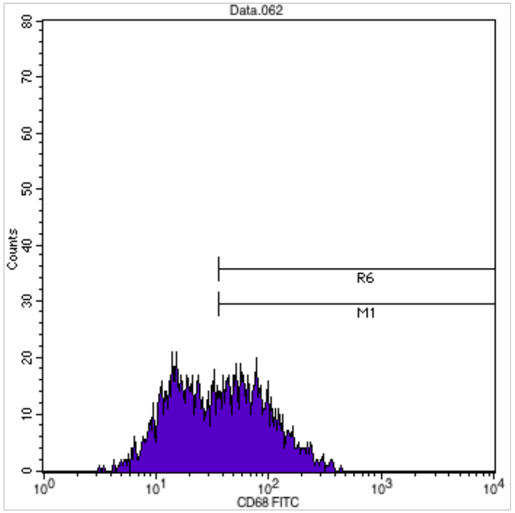

Quadrant Statistics

File: Data.062
Acquisition Date: 18-Oct-24
Gated Events: 1727
X Parameter: CD68 FITC (Log)
Quad Location: 30, 84

Sample ID: 4.4
Gate: G7
Total Events: 52660
Y Parameter: TNFa PE (Log)

Quad	Events	% Gated	% Total
UL	0	0.00	0.00
UR	18	1.04	0.03
LL	0	0.00	0.00
LR	1709	98.96	3.25

Quadrant Statistics

File: Data.062
Acquisition Date: 18-Oct-24
Gated Events: 1727
X Parameter: CD68 FITC (Log)
Quad Location: 30, 31

Sample ID: 4.4
Gate: G7
Total Events: 52660
Y Parameter: IL1 PE-Cy5 (Log)

Quad	Events	% Gated	% Total
UL	0	0.00	0.00
UR	157	9.09	0.30
LL	0	0.00	0.00
LR	1570	90.91	2.98

Quadrant Statistics

File: Data.062
Acquisition Date: 18-Oct-24
Gated Events: 1727
X Parameter: TNFa PE (Log)
Quad Location: 84, 31

Sample ID: 4.4
Gate: G7
Total Events: 52660
Y Parameter: IL1 PE-Cy5 (Log)

Quad	Events	% Gated	% Total
UL	142	8.22	0.27
UR	15	0.87	0.03
LL	1567	90.74	2.98
LR	3	0.17	0.01

Histogram Statistics

File: Data.062
Acquisition Date: 18-Oct-24
Gated Events: 3501
X Parameter: CD68 FITC (Log)

Sample ID: 4.4
Gate: G2
Total Events: 52660

Marker	Left, Right	Events	% Gated	% Total
All	1, 9910	3501	100.00	6.65
M1	36, 9910	1727	49.33	3.28

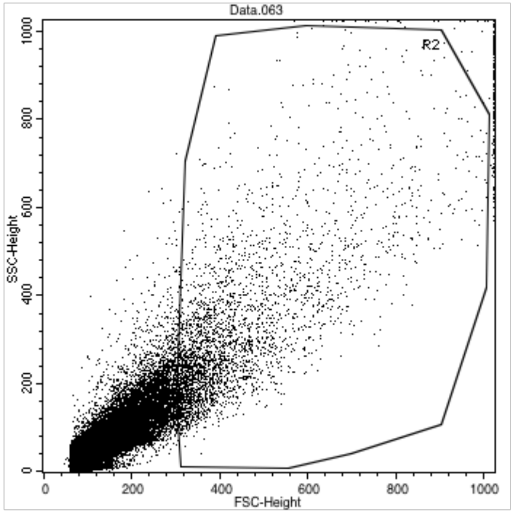

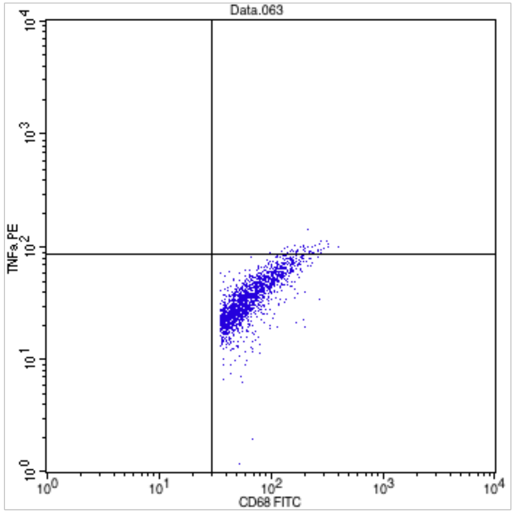

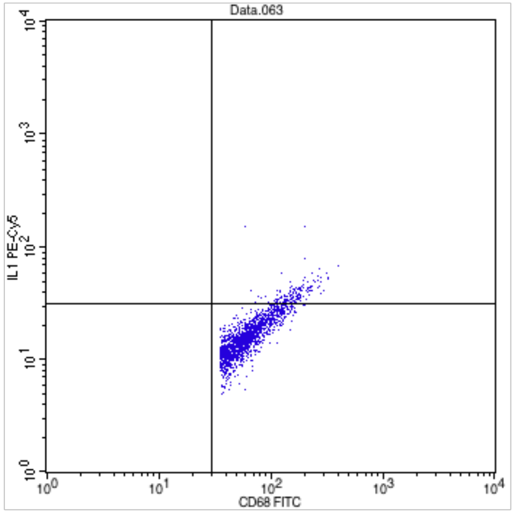

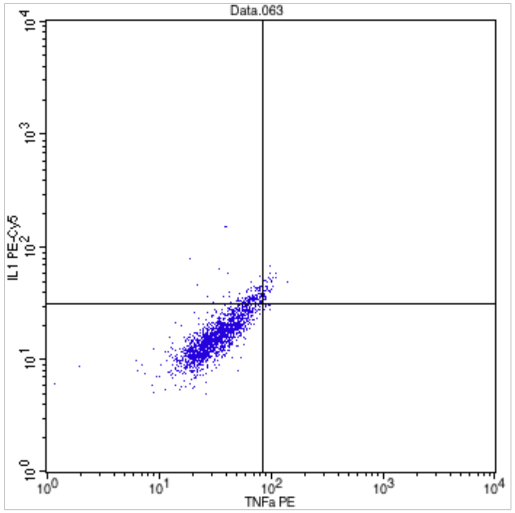

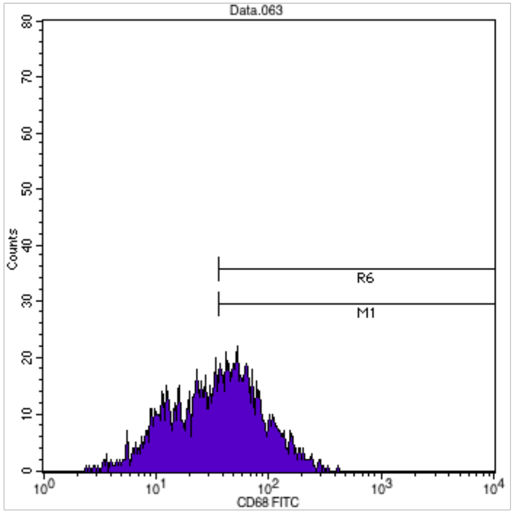

Quadrant Statistics

File: Data.063 Sample ID: 5.4
Acquisition Date: 18-Oct-24 Gate: G7
Gated Events: 1778 Total Events: 60099
X Parameter: CD68 FITC (Log) Y Parameter: TNFa PE (Log)
Quad Location: 30, 84

Quad	Events	% Gated	% Total
UL	0	0.00	0.00
UR	42	2.36	0.07
LL	0	0.00	0.00
LR	1736	97.64	2.89

Quadrant Statistics

File: Data.063 Sample ID: 5.4
Acquisition Date: 18-Oct-24 Gate: G7
Gated Events: 1778 Total Events: 60099
X Parameter: CD68 FITC (Log) Y Parameter: IL1 PE-Cy5 (Log)
Quad Location: 30, 31

Quad	Events	% Gated	% Total
UL	0	0.00	0.00
UR	161	9.06	0.27
LL	0	0.00	0.00
LR	1617	90.94	2.69

Quadrant Statistics

File: Data.063 Sample ID: 5.4
Acquisition Date: 18-Oct-24 Gate: G7
Gated Events: 1778 Total Events: 60099
X Parameter: TNFa PE (Log) Y Parameter: IL1 PE-Cy5 (Log)
Quad Location: 84, 31

Quad	Events	% Gated	% Total
UL	125	7.03	0.21
UR	36	2.02	0.06
LL	1611	90.61	2.68
LR	6	0.34	0.01

Histogram Statistics

File: Data.063 Sample ID: 5.4
Acquisition Date: 18-Oct-24 Gate: G2
Gated Events: 3402 Total Events: 60099
X Parameter: CD68 FITC (Log)

Marker	Left, Right	Events	% Gated	% Total
All	1, 9910	3402	100.00	5.66
M1	36, 9910	1778	52.26	2.96

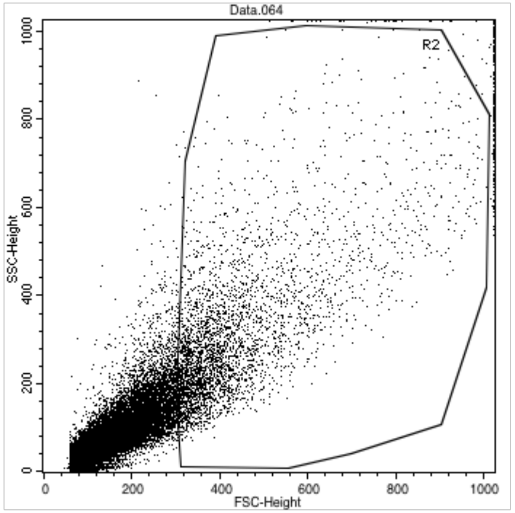

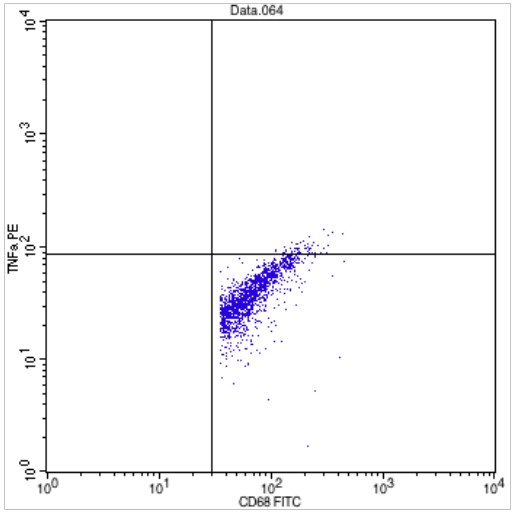

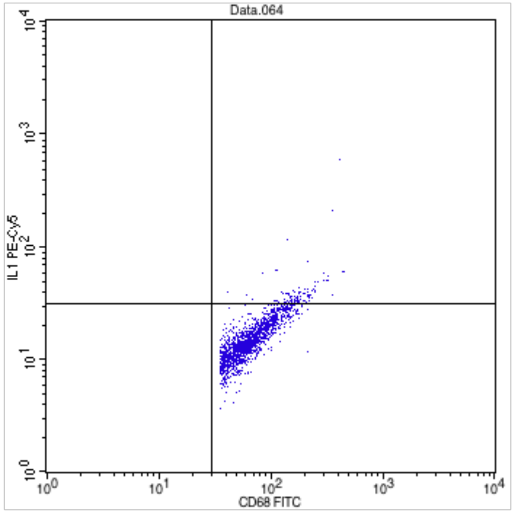

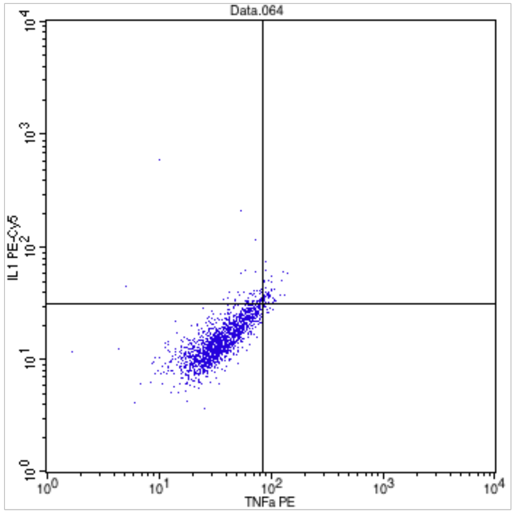

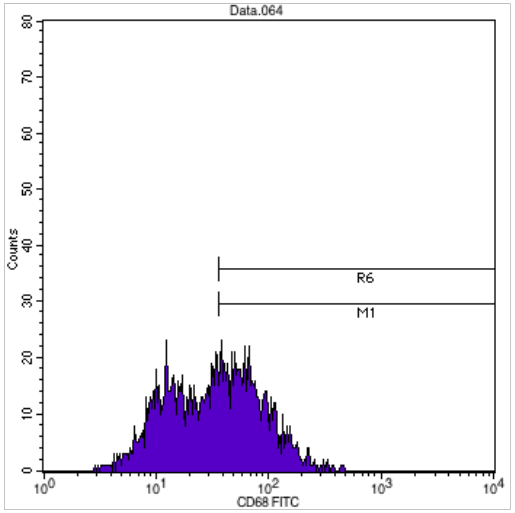

Quadrant Statistics

File: Data.064
Acquisition Date: 18-Oct-24
Gated Events: 1765
X Parameter: CD68 FITC (Log)
Quad Location: 30, 84

Sample ID: 5.5
Gate: G7
Total Events: 52341
Y Parameter: TNFa PE (Log)

Quad	Events	% Gated	% Total
UL	0	0.00	0.00
UR	57	3.23	0.11
LL	0	0.00	0.00
LR	1708	96.77	3.26

Quadrant Statistics

File: Data.064
Acquisition Date: 18-Oct-24
Gated Events: 1765
X Parameter: CD68 FITC (Log)
Quad Location: 30, 31

Sample ID: 5.5
Gate: G7
Total Events: 52341
Y Parameter: IL1 PE-Cy5 (Log)

Quad	Events	% Gated	% Total
UL	0	0.00	0.00
UR	99	5.61	0.19
LL	0	0.00	0.00
LR	1666	94.39	3.18

Quadrant Statistics

File: Data.064
Acquisition Date: 18-Oct-24
Gated Events: 1765
X Parameter: TNFa PE (Log)
Quad Location: 84, 31

Sample ID: 5.5
Gate: G7
Total Events: 52341
Y Parameter: IL1 PE-Cy5 (Log)

Quad	Events	% Gated	% Total
UL	54	3.06	0.10
UR	45	2.55	0.09
LL	1654	93.71	3.16
LR	12	0.68	0.02

Histogram Statistics

File: Data.064
Acquisition Date: 18-Oct-24
Gated Events: 3665
X Parameter: CD68 FITC (Log)

Sample ID: 5.5
Gate: G2
Total Events: 52341

Marker	Left, Right	Events	% Gated	% Total
All	1, 9910	3665	100.00	7.00
M1	36, 9910	1765	48.16	3.37

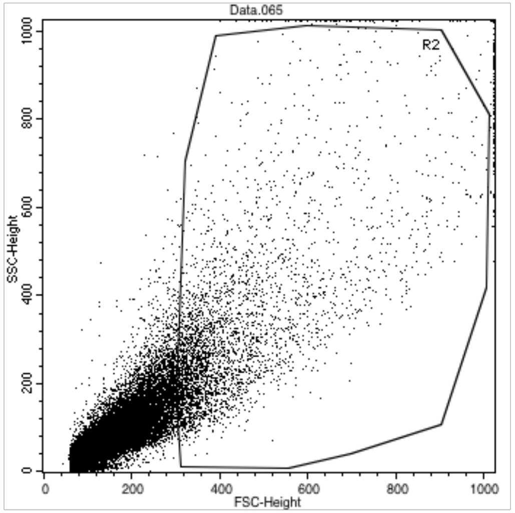

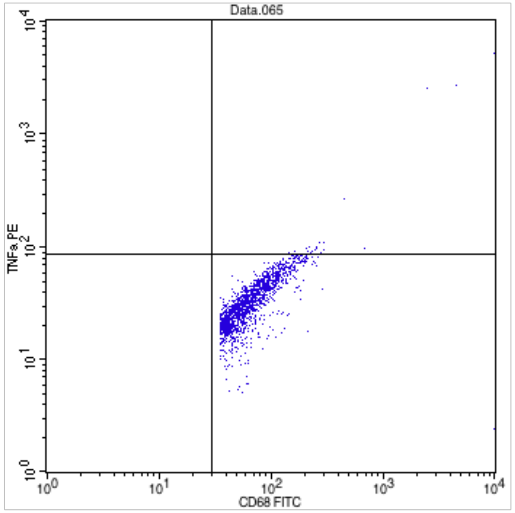

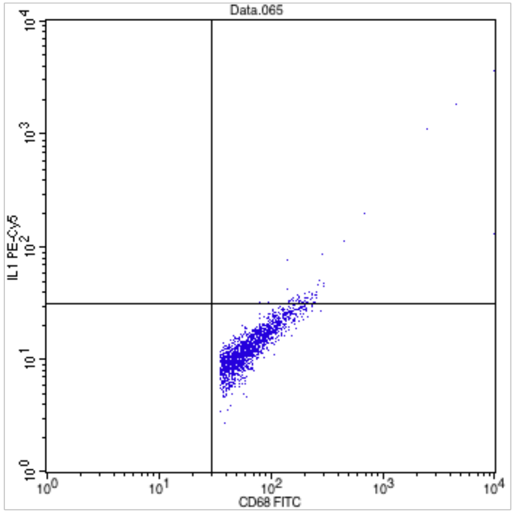

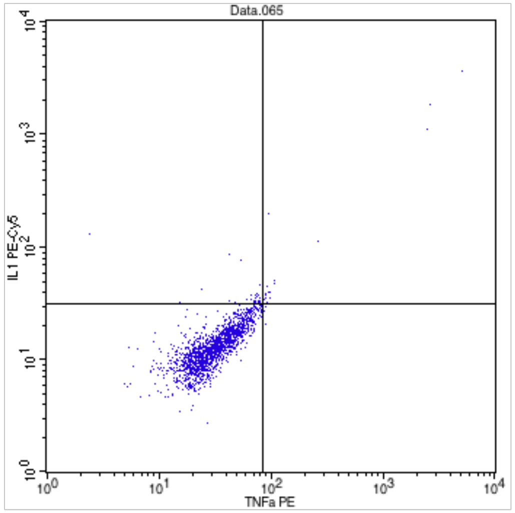

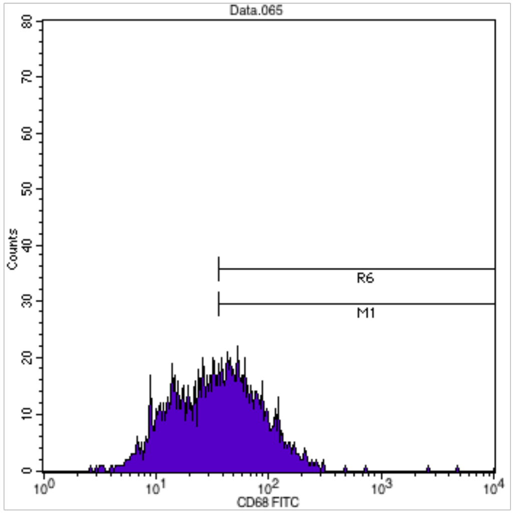

Quadrant Statistics

File: Data.065
Acquisition Date: 18-Oct-24
Gated Events: 1699
X Parameter: CD68 FITC (Log)
Quad Location: 30, 84

Sample ID: 5.6
Gate: G7
Total Events: 54269
Y Parameter: TNFa PE (Log)

Quad	Events	% Gated	% Total
UL	0	0.00	0.00
UR	23	1.35	0.04
LL	0	0.00	0.00
LR	1676	98.65	3.09

Quadrant Statistics

File: Data.065
Acquisition Date: 18-Oct-24
Gated Events: 1699
X Parameter: CD68 FITC (Log)
Quad Location: 30, 31

Sample ID: 5.6
Gate: G7
Total Events: 54269
Y Parameter: IL1 PE-Cy5 (Log)

Quad	Events	% Gated	% Total
UL	0	0.00	0.00
UR	55	3.24	0.10
LL	0	0.00	0.00
LR	1644	96.76	3.03

Quadrant Statistics

File: Data.065
Acquisition Date: 18-Oct-24
Gated Events: 1699
X Parameter: TNFa PE (Log)
Quad Location: 84, 31

Sample ID: 5.6
Gate: G7
Total Events: 54269
Y Parameter: IL1 PE-Cy5 (Log)

Quad	Events	% Gated	% Total
UL	37	2.18	0.07
UR	18	1.06	0.03
LL	1639	96.47	3.02
LR	5	0.29	0.01

Histogram Statistics

File: Data.065
Acquisition Date: 18-Oct-24
Gated Events: 3425
X Parameter: CD68 FITC (Log)

Sample ID: 5.6
Gate: G2
Total Events: 54269

Marker	Left, Right	Events	% Gated	% Total
All	1, 9910	3425	100.00	6.31
M1	36, 9910	1699	49.61	3.13

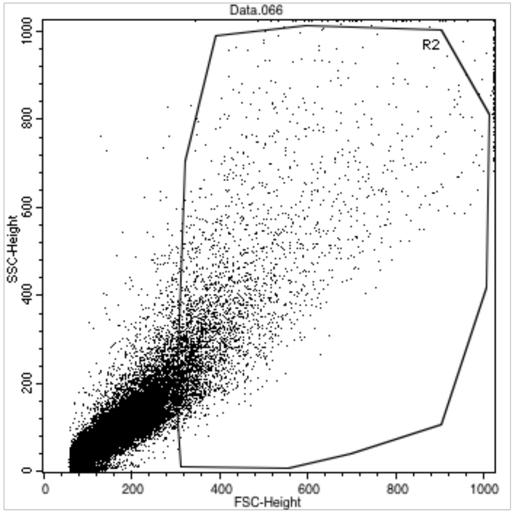

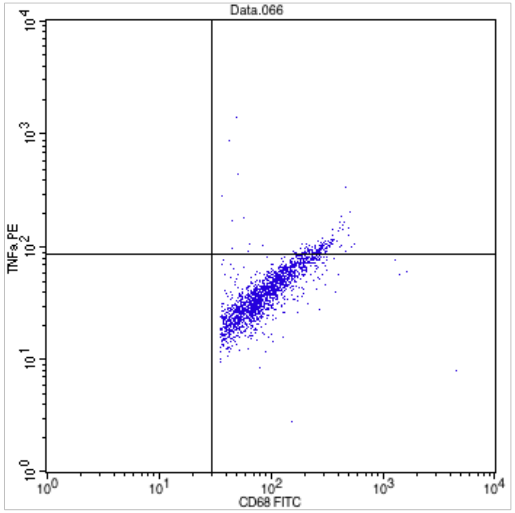

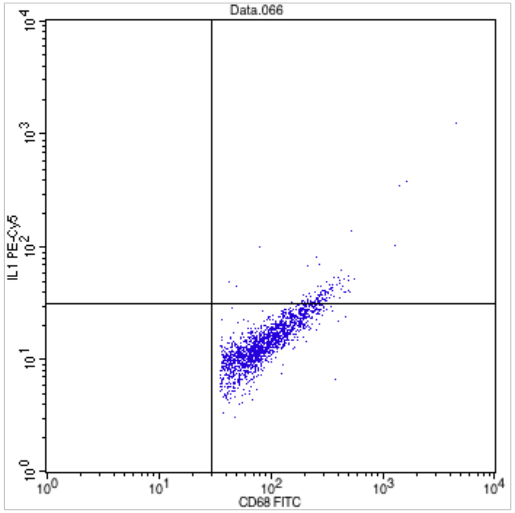

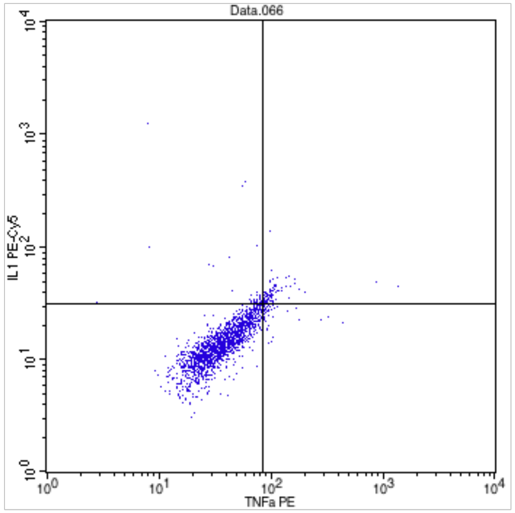

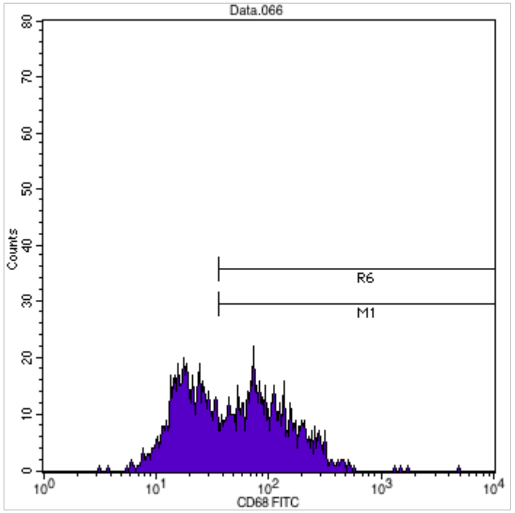

Quadrant Statistics

File: Data.066
Acquisition Date: 18-Oct-24
Gated Events: 1865
X Parameter: CD68 FITC (Log)
Quad Location: 30, 84

Sample ID: 6.4
Gate: G7
Total Events: 60858
Y Parameter: TNFa PE (Log)

Quad	Events	% Gated	% Total
UL	0	0.00	0.00
UR	123	6.60	0.20
LL	0	0.00	0.00
LR	1742	93.40	2.86

Quadrant Statistics

File: Data.066
Acquisition Date: 18-Oct-24
Gated Events: 1865
X Parameter: CD68 FITC (Log)
Quad Location: 30, 31

Sample ID: 6.4
Gate: G7
Total Events: 60858
Y Parameter: IL1 PE-Cy5 (Log)

Quad	Events	% Gated	% Total
UL	0	0.00	0.00
UR	125	6.70	0.21
LL	0	0.00	0.00
LR	1740	93.30	2.86

Quadrant Statistics

File: Data.066
Acquisition Date: 18-Oct-24
Gated Events: 1865
X Parameter: TNFa PE (Log)
Quad Location: 84, 31

Sample ID: 6.4
Gate: G7
Total Events: 60858
Y Parameter: IL1 PE-Cy5 (Log)

Quad	Events	% Gated	% Total
UL	47	2.52	0.08
UR	78	4.18	0.13
LL	1695	90.88	2.79
LR	45	2.41	0.07

Histogram Statistics

File: Data.066
Acquisition Date: 18-Oct-24
Gated Events: 3330
X Parameter: CD68 FITC (Log)

Sample ID: 6.4
Gate: G2
Total Events: 60858

Marker	Left, Right	Events	% Gated	% Total
All	1, 9910	3330	100.00	5.47
M1	36, 9910	1865	56.01	3.06

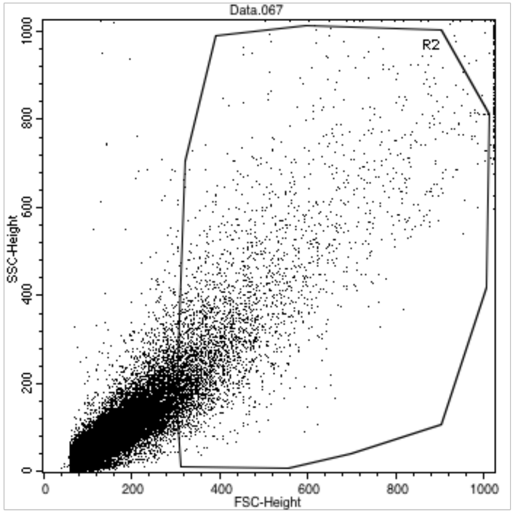

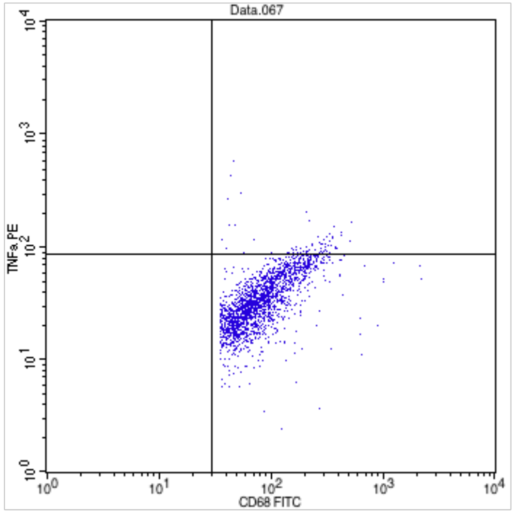

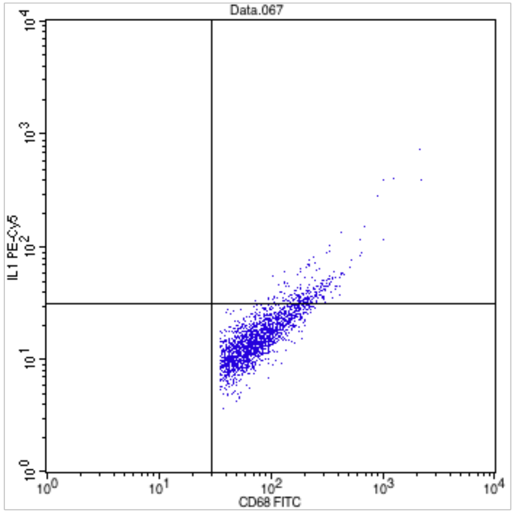

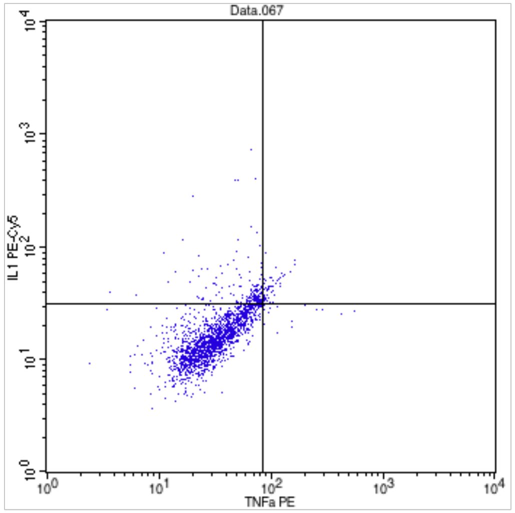

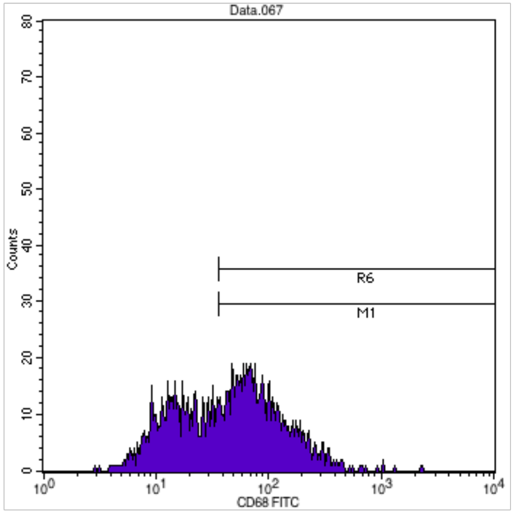

Quadrant Statistics

File: Data.067
Acquisition Date: 18-Oct-24
Gated Events: 2013
X Parameter: CD68 FITC (Log)
Quad Location: 30, 84

Sample ID: 7.4
Gate: G7
Total Events: 60831
Y Parameter: TNFa PE (Log)

Quad	Events	% Gated	% Total
UL	0	0.00	0.00
UR	70	3.48	0.12
LL	0	0.00	0.00
LR	1943	96.52	3.19

Quadrant Statistics

File: Data.067
Acquisition Date: 18-Oct-24
Gated Events: 2013
X Parameter: CD68 FITC (Log)
Quad Location: 30, 31

Sample ID: 7.4
Gate: G7
Total Events: 60831
Y Parameter: IL1 PE-Cy5 (Log)

Quad	Events	% Gated	% Total
UL	0	0.00	0.00
UR	216	10.73	0.36
LL	0	0.00	0.00
LR	1797	89.27	2.95

Quadrant Statistics

File: Data.067
Acquisition Date: 18-Oct-24
Gated Events: 2013
X Parameter: TNFa PE (Log)
Quad Location: 84, 31

Sample ID: 7.4
Gate: G7
Total Events: 60831
Y Parameter: IL1 PE-Cy5 (Log)

Quad	Events	% Gated	% Total
UL	161	8.00	0.26
UR	55	2.73	0.09
LL	1782	88.52	2.93
LR	15	0.75	0.02

Histogram Statistics

File: Data.067
Acquisition Date: 18-Oct-24
Gated Events: 3401
X Parameter: CD68 FITC (Log)

Sample ID: 7.4
Gate: G2
Total Events: 60831

Marker	Left, Right	Events	% Gated	% Total
All	1, 9910	3401	100.00	5.59
M1	36, 9910	2013	59.19	3.31

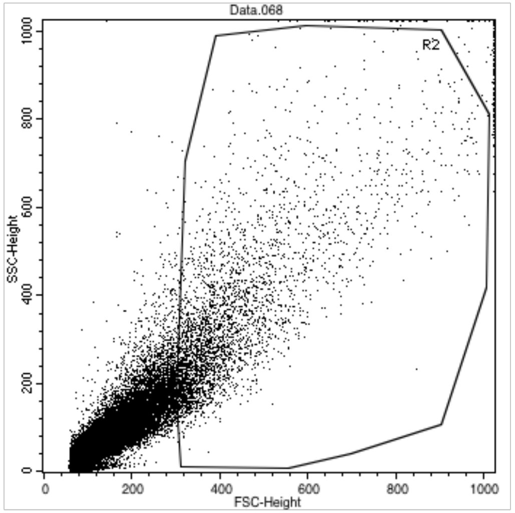

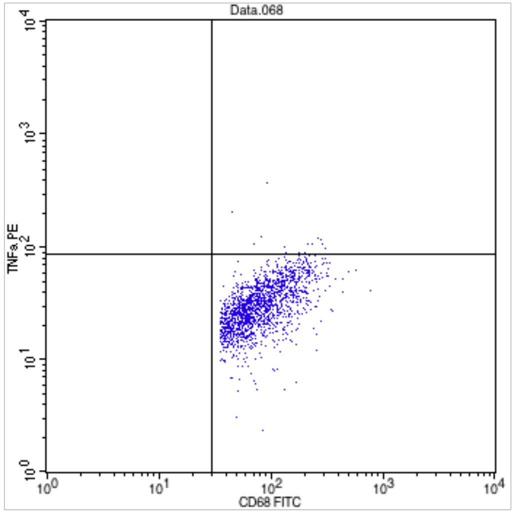

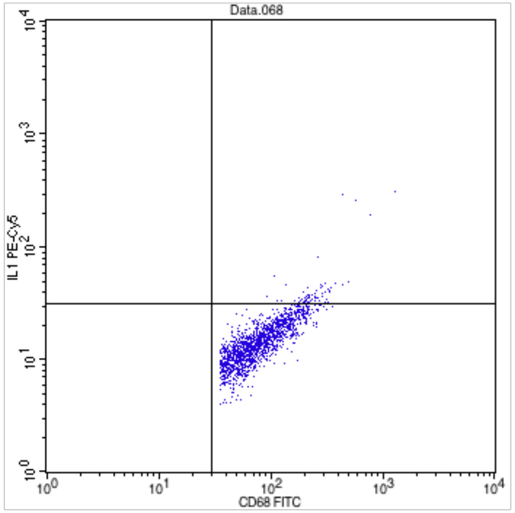

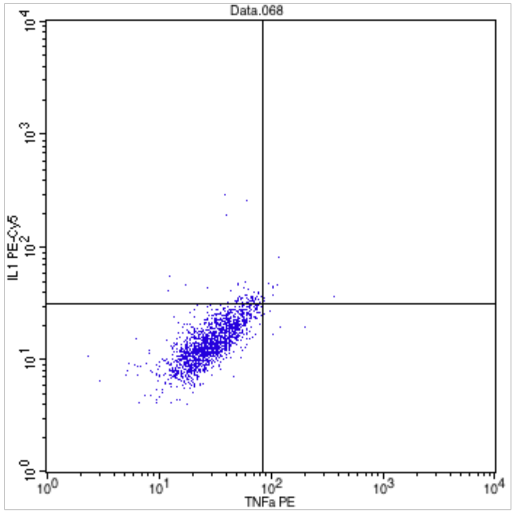

Quadrant Statistics

File: Data.068
Acquisition Date: 18-Oct-24
Gated Events: 1751
X Parameter: CD68 FITC (Log)
Quad Location: 30, 84

Sample ID: 7.6
Gate: G7
Total Events: 58005
Y Parameter: TNFa PE (Log)

Quad	Events	% Gated	% Total
UL	0	0.00	0.00
UR	15	0.86	0.03
LL	0	0.00	0.00
LR	1736	99.14	2.99

Quadrant Statistics

File: Data.068
Acquisition Date: 18-Oct-24
Gated Events: 1751
X Parameter: CD68 FITC (Log)
Quad Location: 30, 31

Sample ID: 7.6
Gate: G7
Total Events: 58005
Y Parameter: IL1 PE-Cy5 (Log)

Quad	Events	% Gated	% Total
UL	0	0.00	0.00
UR	66	3.77	0.11
LL	0	0.00	0.00
LR	1685	96.23	2.90

Quadrant Statistics

File: Data.068
Acquisition Date: 18-Oct-24
Gated Events: 1751
X Parameter: TNFa PE (Log)
Quad Location: 84, 31

Sample ID: 7.6
Gate: G7
Total Events: 58005
Y Parameter: IL1 PE-Cy5 (Log)

Quad	Events	% Gated	% Total
UL	56	3.20	0.10
UR	10	0.57	0.02
LL	1680	95.95	2.90
LR	5	0.29	0.01

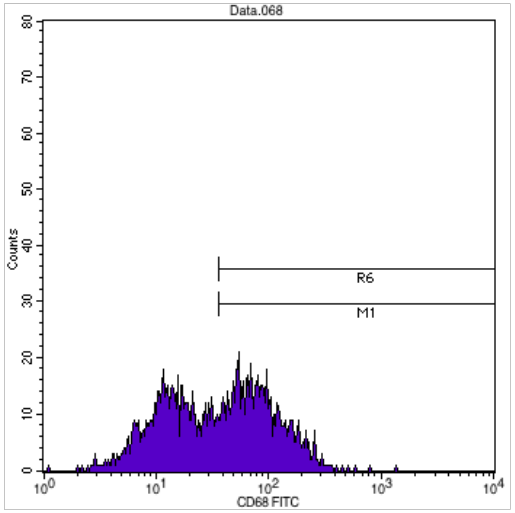

Histogram Statistics

File: Data.068
Acquisition Date: 18-Oct-24
Gated Events: 3491
X Parameter: CD68 FITC (Log)

Sample ID: 7.6
Gate: G2
Total Events: 58005

Marker	Left, Right	Events	% Gated	% Total
All	1, 9910	3491	100.00	6.02
M1	36, 9910	1751	50.16	3.02

CD68-IL6-NFkb
G2=R2 (Populasi sel hidup)
G7=R2 and R6 (Populasi total sel CD68)
16-10-2024

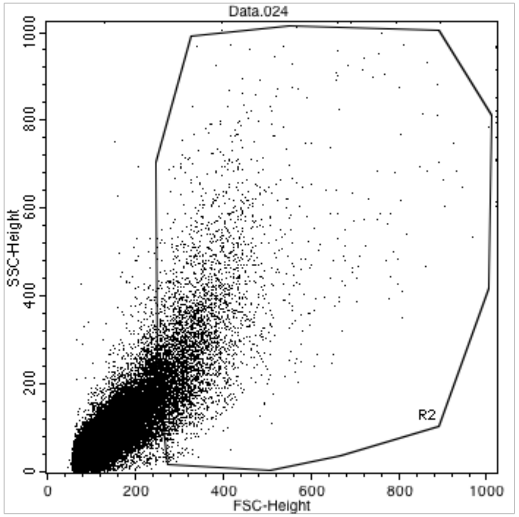

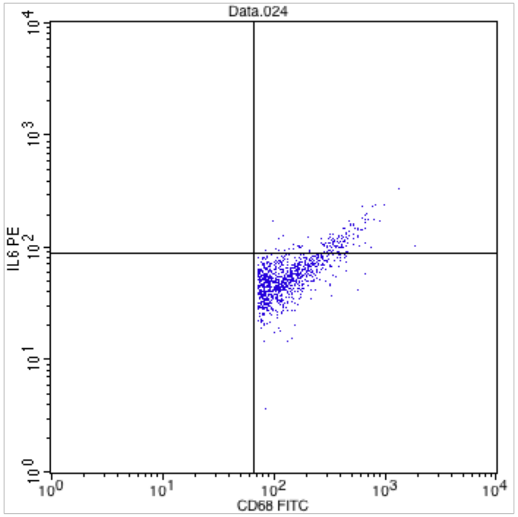

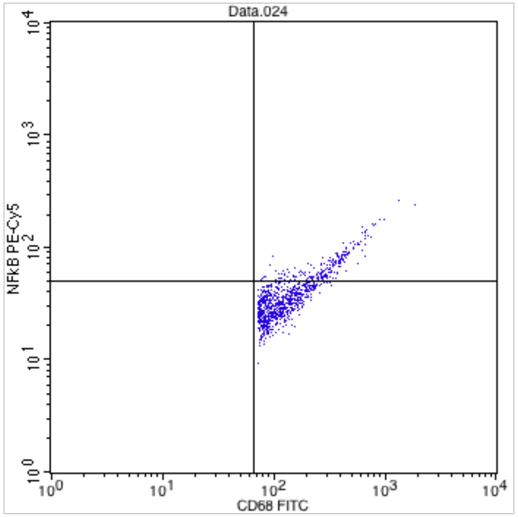

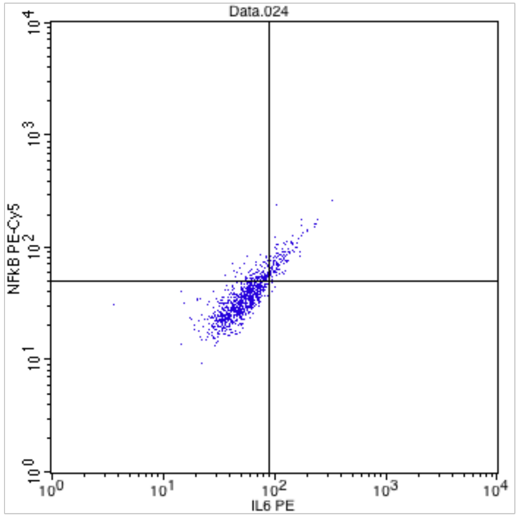

Quadrant Statistics

File: Data.024
Acquisition Date: 16-Oct-24
Gated Events: 991
X Parameter: CD68 FITC (Log)
Quad Location: 66, 90
Sample ID: Norm.1
Gate: G7
Total Events: 70824
Y Parameter: IL6 PE (Log)

Quad	Events	% Gated	% Total
UL	0	0.00	0.00
UR	125	12.61	0.18
LL	0	0.00	0.00
LR	866	87.39	1.22

Quadrant Statistics

File: Data.024
Acquisition Date: 16-Oct-24
Gated Events: 991
X Parameter: CD68 FITC (Log)
Quad Location: 66, 50
Sample ID: Norm.1
Gate: G7
Total Events: 70824
Y Parameter: NFkB PE-Cy5 (Log)

Quad	Events	% Gated	% Total
UL	0	0.00	0.00
UR	222	22.40	0.31
LL	0	0.00	0.00
LR	769	77.60	1.09

Quadrant Statistics

File: Data.024
Acquisition Date: 16-Oct-24
Gated Events: 991
X Parameter: IL6 PE (Log)
Quad Location: 90, 50
Sample ID: Norm.1
Gate: G7
Total Events: 70824
Y Parameter: NFkB PE-Cy5 (Log)

Quad	Events	% Gated	% Total
UL	102	10.29	0.14
UR	120	12.11	0.17
LL	764	77.09	1.08
LR	5	0.50	0.01

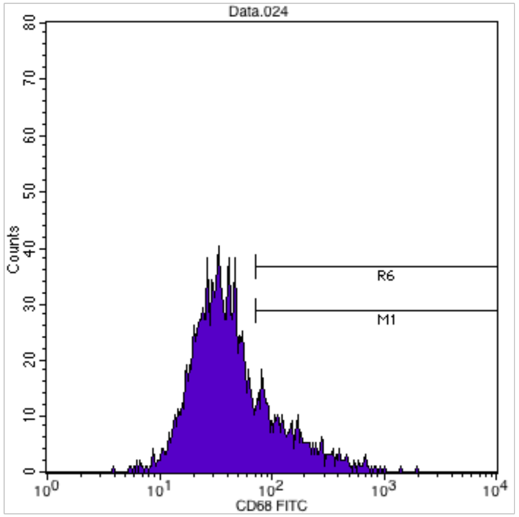

Histogram Statistics

File: Data.024
Acquisition Date: 16-Oct-24
Gated Events: 4795
X Parameter: CD68 FITC (Log)
Sample ID: Norm.1
Gate: G2
Total Events: 70824

Marker	Left, Right	Events	% Gated	% Total
All	1, 9910	4795	100.00	6.77
M1	72, 9910	991	20.67	1.40

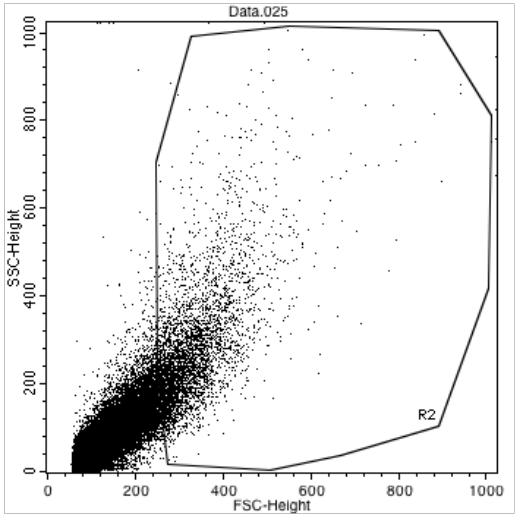

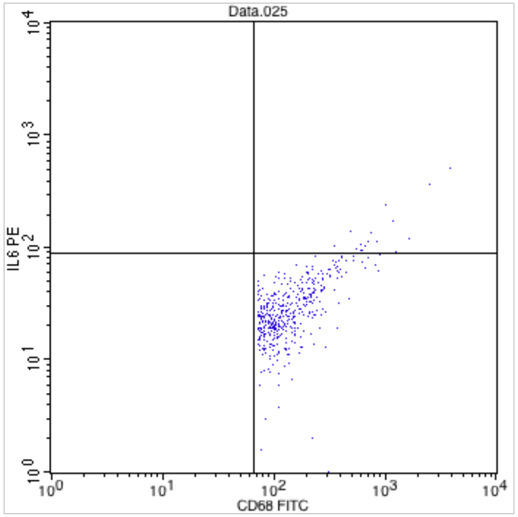

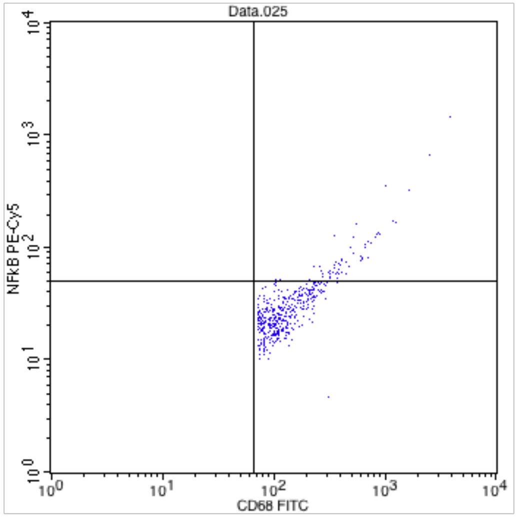

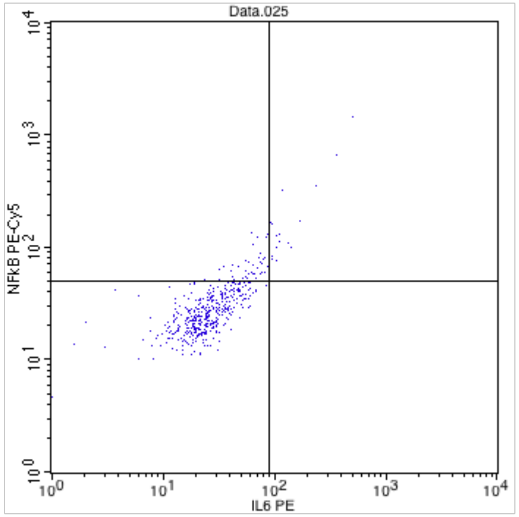

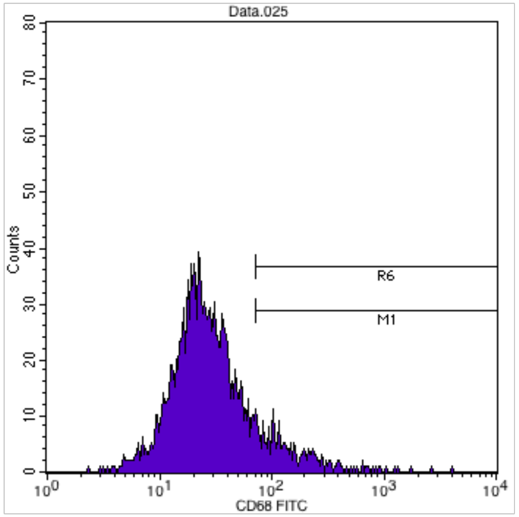

Quadrant Statistics

File: Data.025
Acquisition Date: 16-Oct-24
Gated Events: 468
X Parameter: CD68 FITC (Log)
Quad Location: 66, 90
Sample ID: +.1
Gate: G7
Total Events: 66732
Y Parameter: IL6 PE (Log)

Quad	Events	% Gated	% Total
UL	0	0.00	0.00
UR	16	3.42	0.02
LL	0	0.00	0.00
LR	452	96.58	0.68

Quadrant Statistics

File: Data.025
Acquisition Date: 16-Oct-24
Gated Events: 468
X Parameter: CD68 FITC (Log)
Quad Location: 66, 50
Sample ID: +.1
Gate: G7
Total Events: 66732
Y Parameter: NFkB PE-Cy5 (Log)

Quad	Events	% Gated	% Total
UL	0	0.00	0.00
UR	59	12.61	0.09
LL	0	0.00	0.00
LR	409	87.39	0.61

Quadrant Statistics

File: Data.025
Acquisition Date: 16-Oct-24
Gated Events: 468
X Parameter: IL6 PE (Log)
Quad Location: 90, 50
Sample ID: +.1
Gate: G7
Total Events: 66732
Y Parameter: NFkB PE-Cy5 (Log)

Quad	Events	% Gated	% Total
UL	43	9.19	0.06
UR	16	3.42	0.02
LL	409	87.39	0.61
LR	0	0.00	0.00

Histogram Statistics

File: Data.025
Acquisition Date: 16-Oct-24
Gated Events: 4482
X Parameter: CD68 FITC (Log)
Sample ID: +.1
Gate: G2
Total Events: 66732

Marker	Left, Right	Events	% Gated	% Total
All	1, 9910	4482	100.00	6.72
M1	72, 9910	468	10.44	0.70

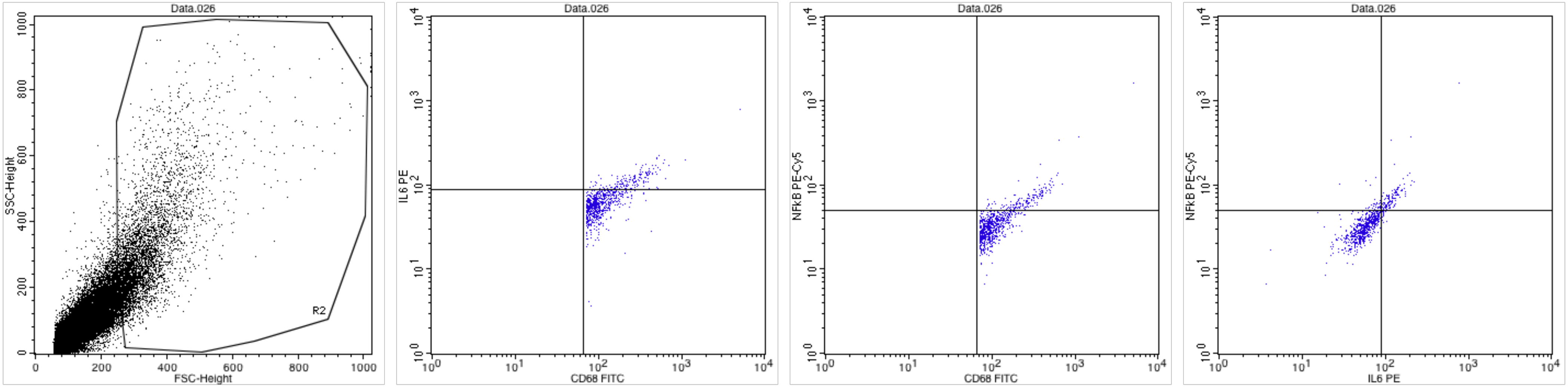

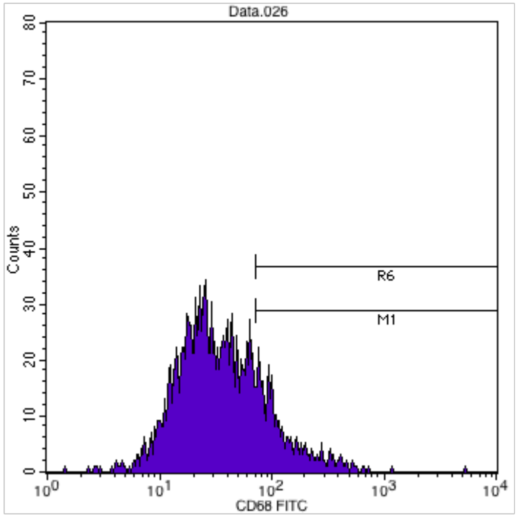

Quadrant Statistics

File: Data.026
Acquisition Date: 16-Oct-24
Gated Events: 857
X Parameter: CD68 FITC (Log)
Quad Location: 66, 90
Sample ID: +.2
Gate: G7
Total Events: 62648
Y Parameter: IL6 PE (Log)

Quad	Events	% Gated	% Total
UL	0	0.00	0.00
UR	147	17.15	0.23
LL	0	0.00	0.00
LR	710	82.85	1.13

Quadrant Statistics

File: Data.026
Acquisition Date: 16-Oct-24
Gated Events: 857
X Parameter: CD68 FITC (Log)
Quad Location: 66, 50
Sample ID: +.2
Gate: G7
Total Events: 62648
Y Parameter: NFkB PE-Cy5 (Log)

Quad	Events	% Gated	% Total
UL	0	0.00	0.00
UR	163	19.02	0.26
LL	0	0.00	0.00
LR	694	80.98	1.11

Quadrant Statistics

File: Data.026
Acquisition Date: 16-Oct-24
Gated Events: 857
X Parameter: IL6 PE (Log)
Quad Location: 90, 50
Sample ID: +.2
Gate: G7
Total Events: 62648
Y Parameter: NFkB PE-Cy5 (Log)

Quad	Events	% Gated	% Total
UL	36	4.20	0.06
UR	127	14.82	0.20
LL	674	78.65	1.08
LR	20	2.33	0.03

Histogram Statistics

File: Data.026
Acquisition Date: 16-Oct-24
Gated Events: 4828
X Parameter: CD68 FITC (Log)
Sample ID: +.2
Gate: G2
Total Events: 62648

Marker	Left, Right	Events	% Gated	% Total
All	1, 9910	4828	100.00	7.71
M1	72, 9910	857	17.75	1.37

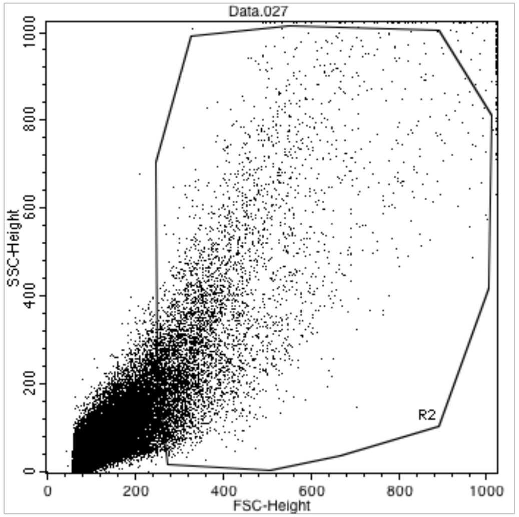

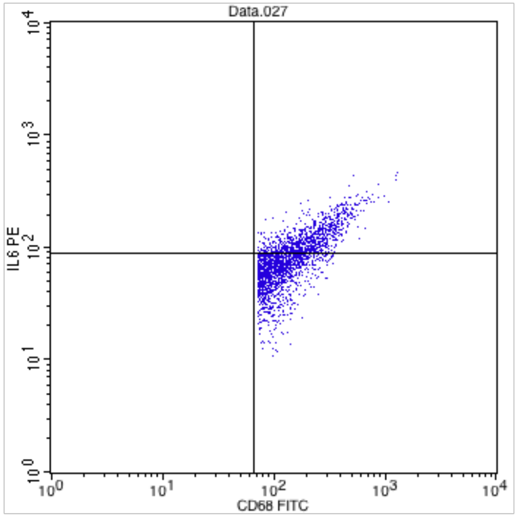

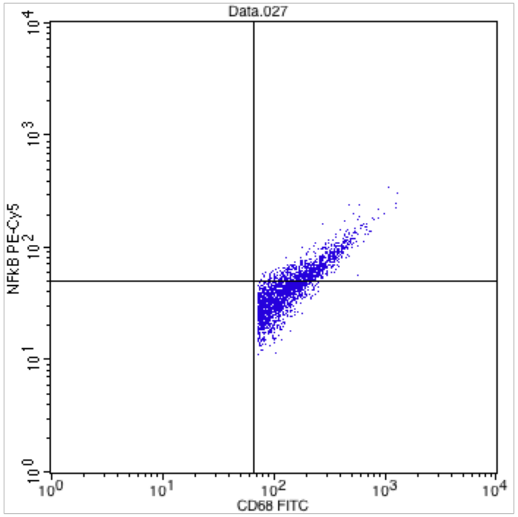

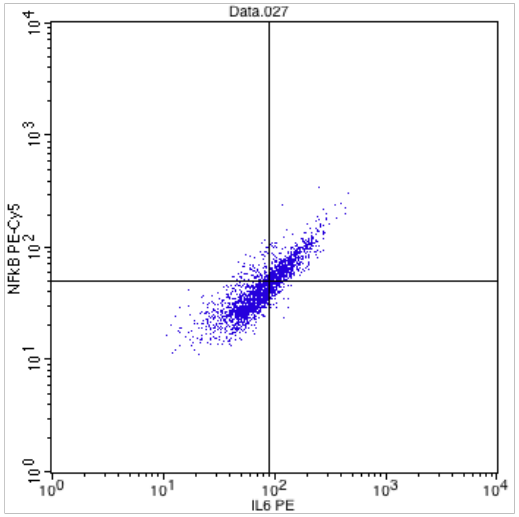

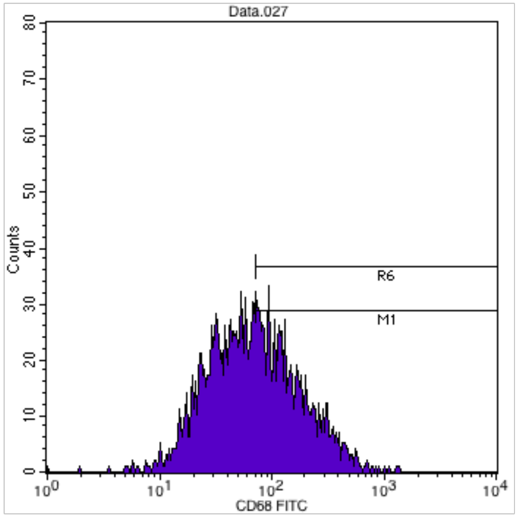

Quadrant Statistics

File: Data.027
Acquisition Date: 16-Oct-24
Gated Events: 2480
X Parameter: CD68 FITC (Log)
Quad Location: 66, 90
Sample ID: -.1
Gate: G7
Total Events: 47924
Y Parameter: IL6 PE (Log)

Quad	Events	% Gated	% Total
UL	0	0.00	0.00
UR	904	36.45	1.89
LL	0	0.00	0.00
LR	1576	63.55	3.29

Quadrant Statistics

File: Data.027
Acquisition Date: 16-Oct-24
Gated Events: 2480
X Parameter: CD68 FITC (Log)
Quad Location: 66, 50
Sample ID: -.1
Gate: G7
Total Events: 47924
Y Parameter: NFkB PE-Cy5 (Log)

Quad	Events	% Gated	% Total
UL	0	0.00	0.00
UR	852	34.35	1.78
LL	0	0.00	0.00
LR	1628	65.65	3.40

Quadrant Statistics

File: Data.027
Acquisition Date: 16-Oct-24
Gated Events: 2480
X Parameter: IL6 PE (Log)
Quad Location: 90, 50
Sample ID: -.1
Gate: G7
Total Events: 47924
Y Parameter: NFkB PE-Cy5 (Log)

Quad	Events	% Gated	% Total
UL	135	5.44	0.28
UR	717	28.91	1.50
LL	1441	58.10	3.01
LR	187	7.54	0.39

Histogram Statistics

File: Data.027
Acquisition Date: 16-Oct-24
Gated Events: 5516
X Parameter: CD68 FITC (Log)
Sample ID: -.1
Gate: G2
Total Events: 47924

Marker	Left, Right	Events	% Gated	% Total
All	1, 9910	5516	100.00	11.51
M1	72, 9910	2480	44.96	5.17

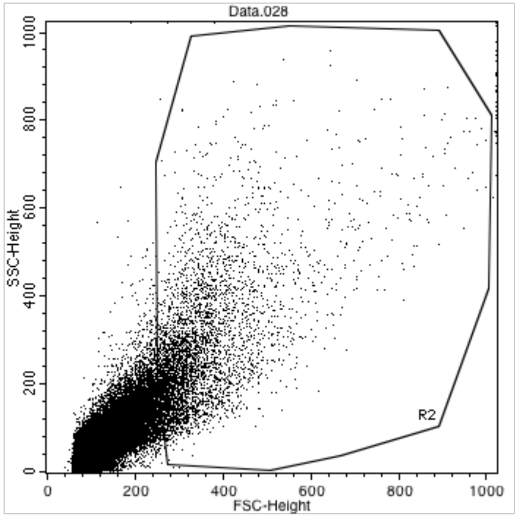

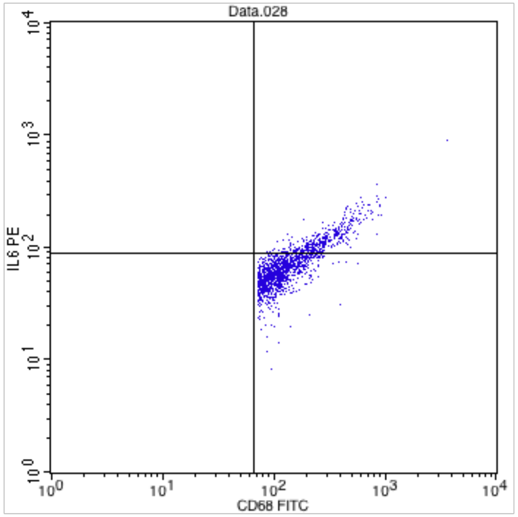

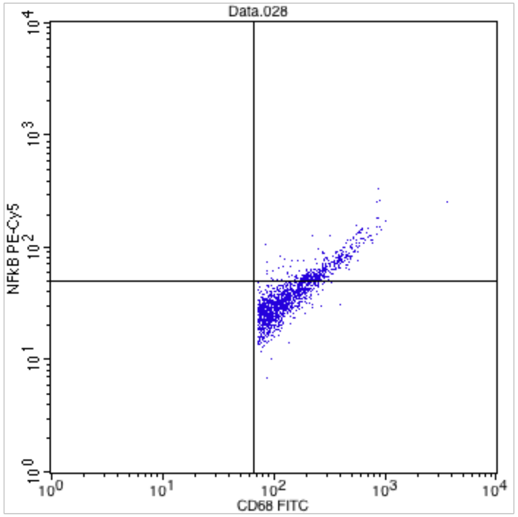

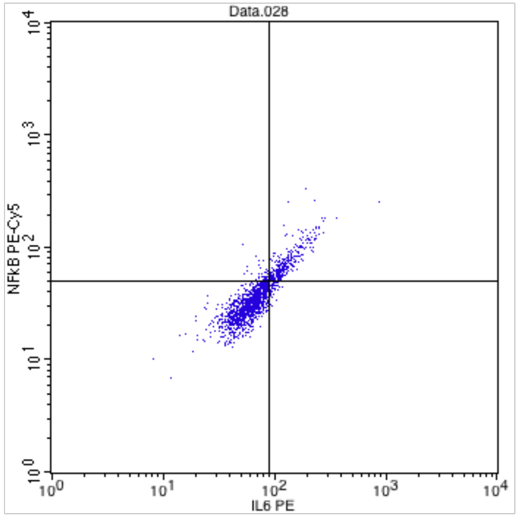

Quadrant Statistics

File: Data.028
Acquisition Date: 16-Oct-24
Gated Events: 1649
X Parameter: CD68 FITC (Log)
Quad Location: 66, 90
Sample ID: -.2
Gate: G7
Total Events: 65604
Y Parameter: IL6 PE (Log)

Quad	Events	% Gated	% Total
UL	0	0.00	0.00
UR	384	23.29	0.59
LL	0	0.00	0.00
LR	1265	76.71	1.93

Quadrant Statistics

File: Data.028
Acquisition Date: 16-Oct-24
Gated Events: 1649
X Parameter: CD68 FITC (Log)
Quad Location: 66, 50
Sample ID: -.2
Gate: G7
Total Events: 65604
Y Parameter: NFkB PE-Cy5 (Log)

Quad	Events	% Gated	% Total
UL	0	0.00	0.00
UR	380	23.04	0.58
LL	0	0.00	0.00
LR	1269	76.96	1.93

Quadrant Statistics

File: Data.028
Acquisition Date: 16-Oct-24
Gated Events: 1649
X Parameter: IL6 PE (Log)
Quad Location: 90, 50
Sample ID: -.2
Gate: G7
Total Events: 65604
Y Parameter: NFkB PE-Cy5 (Log)

Quad	Events	% Gated	% Total
UL	53	3.21	0.08
UR	327	19.83	0.50
LL	1212	73.50	1.85
LR	57	3.46	0.09

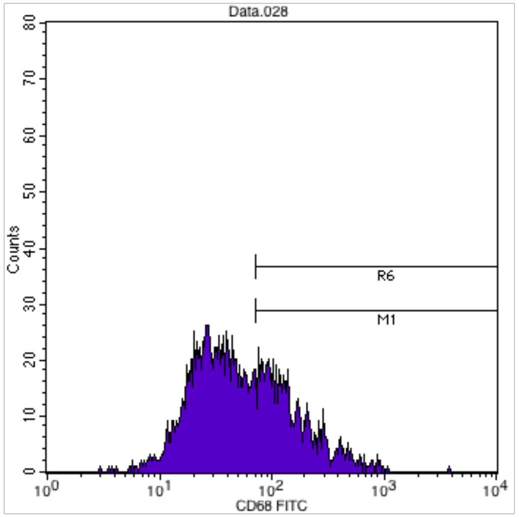

Histogram Statistics

File: Data.028
Acquisition Date: 16-Oct-24
Gated Events: 4674
X Parameter: CD68 FITC (Log)
Sample ID: -.2
Gate: G2
Total Events: 65604

Marker	Left, Right	Events	% Gated	% Total
All	1, 9910	4674	100.00	7.12
M1	72, 9910	1649	35.28	2.51

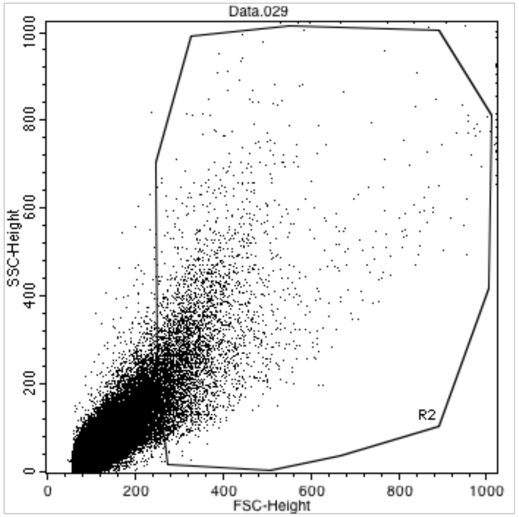

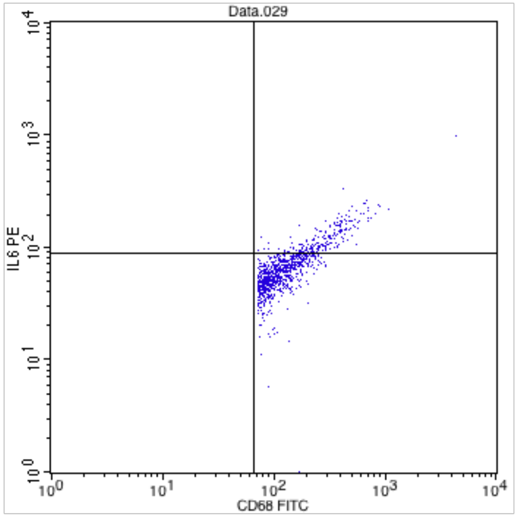

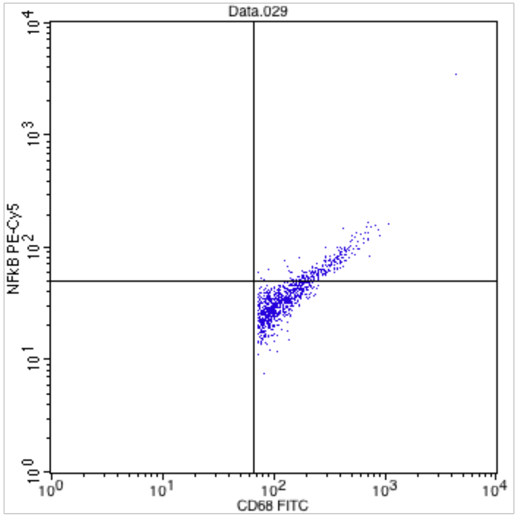

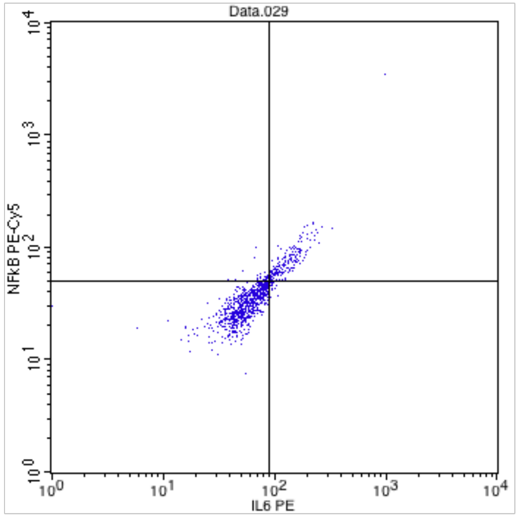

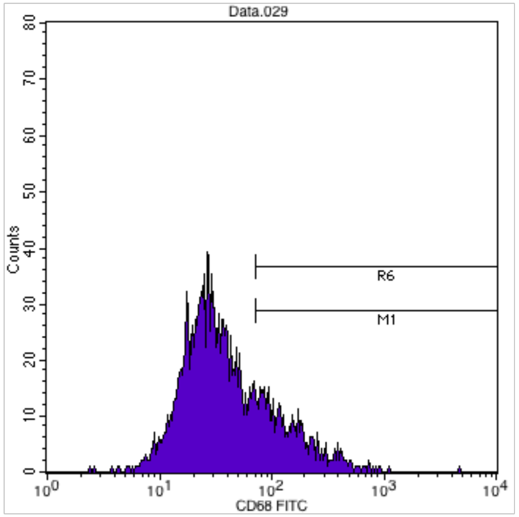

Quadrant Statistics

File: Data.029 Sample ID: -.3
Acquisition Date: 16-Oct-24 Gate: G7
Gated Events: 1027 Total Events: 61391
X Parameter: CD68 FITC (Log) Y Parameter: IL6 PE (Log)
Quad Location: 66, 90

Quad	Events	% Gated	% Total
UL	0	0.00	0.00
UR	207	20.16	0.34
LL	0	0.00	0.00
LR	820	79.84	1.34

Quadrant Statistics

File: Data.029 Sample ID: -.3
Acquisition Date: 16-Oct-24 Gate: G7
Gated Events: 1027 Total Events: 61391
X Parameter: CD68 FITC (Log) Y Parameter: NFkB PE-Cy5 (Log)
Quad Location: 66, 50

Quad	Events	% Gated	% Total
UL	0	0.00	0.00
UR	223	21.71	0.36
LL	0	0.00	0.00
LR	804	78.29	1.31

Quadrant Statistics

File: Data.029 Sample ID: -.3
Acquisition Date: 16-Oct-24 Gate: G7
Gated Events: 1027 Total Events: 61391
X Parameter: IL6 PE (Log) Y Parameter: NFkB PE-Cy5 (Log)
Quad Location: 90, 50

Quad	Events	% Gated	% Total
UL	43	4.19	0.07
UR	180	17.53	0.29
LL	777	75.66	1.27
LR	27	2.63	0.04

Histogram Statistics

File: Data.029 Sample ID: -.3
Acquisition Date: 16-Oct-24 Gate: G2
Gated Events: 4645 Total Events: 61391
X Parameter: CD68 FITC (Log)

Marker	Left, Right	Events	% Gated	% Total
All	1, 9910	4645	100.00	7.57
M1	72, 9910	1027	22.11	1.67

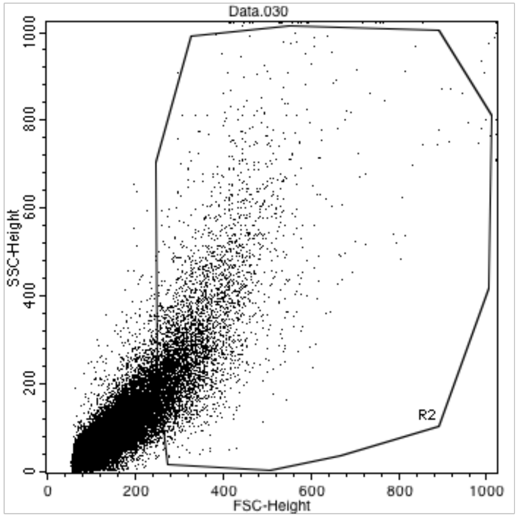

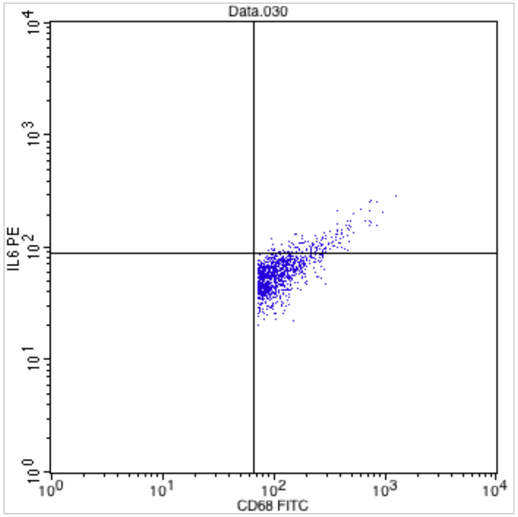

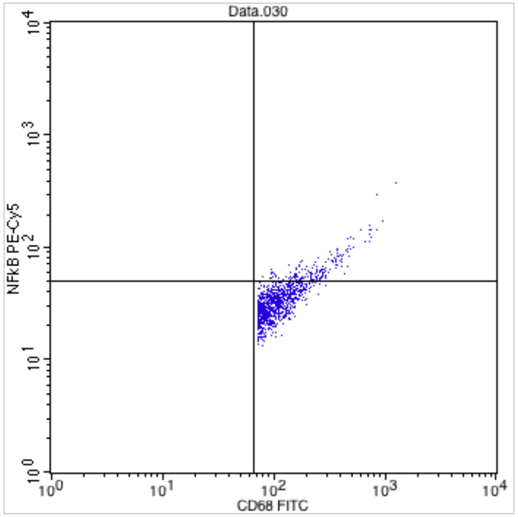

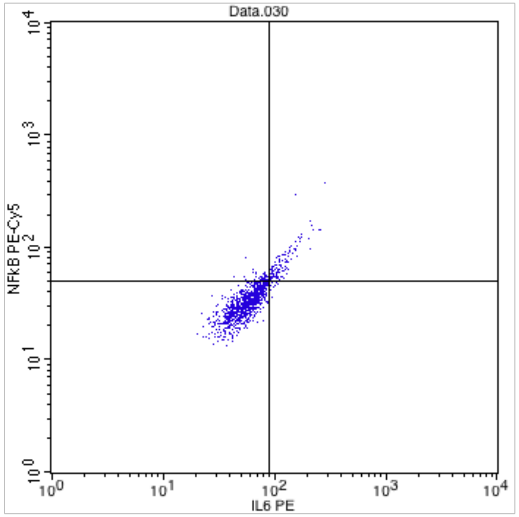

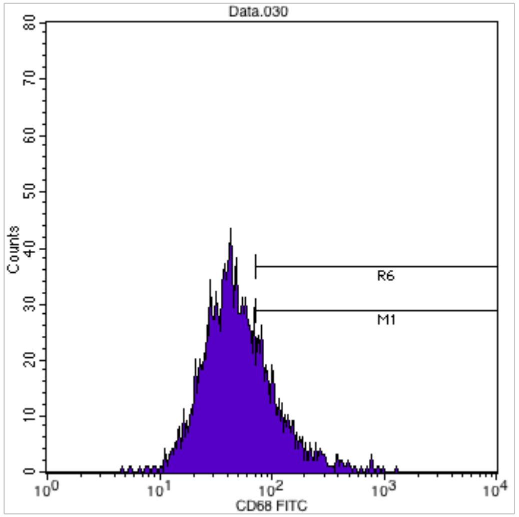

Quadrant Statistics

File: Data.030 Sample ID: 1.2
Acquisition Date: 16-Oct-24 Gate: G7
Gated Events: 1194 Total Events: 56159
X Parameter: CD68 FITC (Log) Y Parameter: IL6 PE (Log)
Quad Location: 66, 90

Quad	Events	% Gated	% Total
UL	0	0.00	0.00
UR	134	11.22	0.24
LL	0	0.00	0.00
LR	1060	88.78	1.89

Quadrant Statistics

File: Data.030 Sample ID: 1.2
Acquisition Date: 16-Oct-24 Gate: G7
Gated Events: 1194 Total Events: 56159
X Parameter: CD68 FITC (Log) Y Parameter: NFkB PE-Cy5 (Log)
Quad Location: 66, 50

Quad	Events	% Gated	% Total
UL	0	0.00	0.00
UR	167	13.99	0.30
LL	0	0.00	0.00
LR	1027	86.01	1.83

Quadrant Statistics

File: Data.030 Sample ID: 1.2
Acquisition Date: 16-Oct-24 Gate: G7
Gated Events: 1194 Total Events: 56159
X Parameter: IL6 PE (Log) Y Parameter: NFkB PE-Cy5 (Log)
Quad Location: 90, 50

Quad	Events	% Gated	% Total
UL	48	4.02	0.09
UR	119	9.97	0.21
LL	1012	84.76	1.80
LR	15	1.26	0.03

Histogram Statistics

File: Data.030 Sample ID: 1.2
Acquisition Date: 16-Oct-24 Gate: G2
Gated Events: 4794 Total Events: 56159
X Parameter: CD68 FITC (Log)

Marker	Left, Right	Events	% Gated	% Total
All	1, 9910	4794	100.00	8.54
M1	72, 9910	1194	24.91	2.13

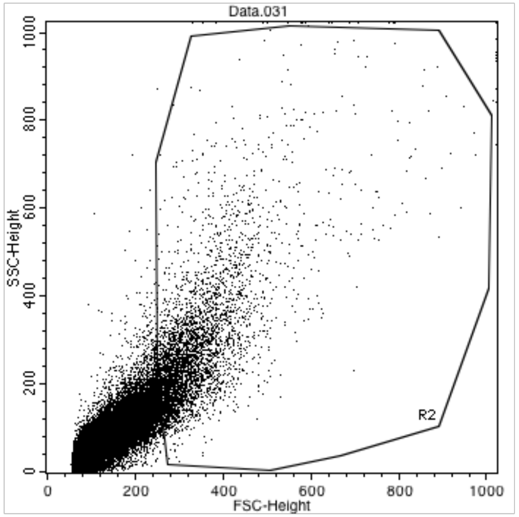

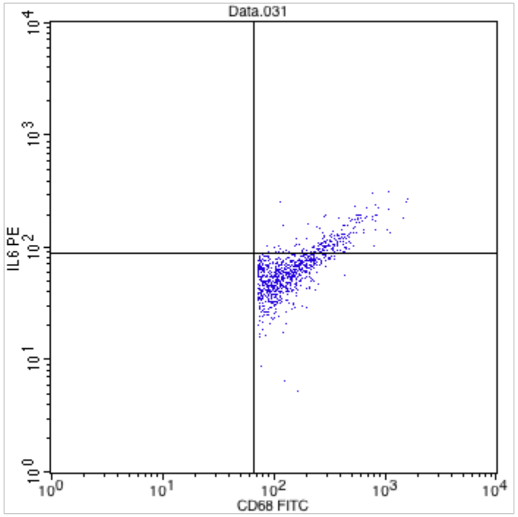

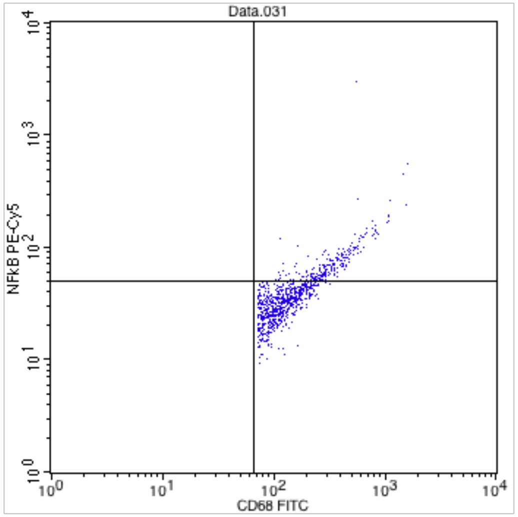

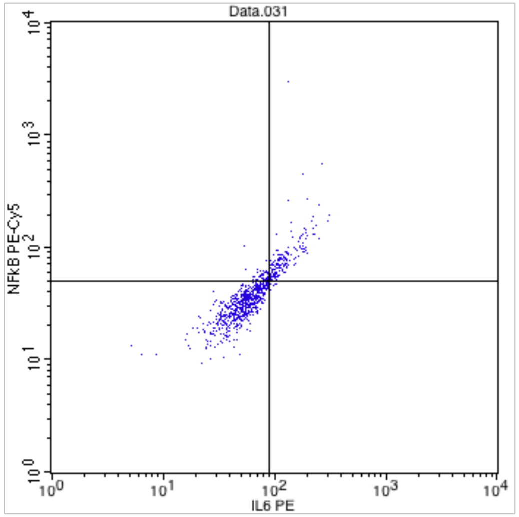

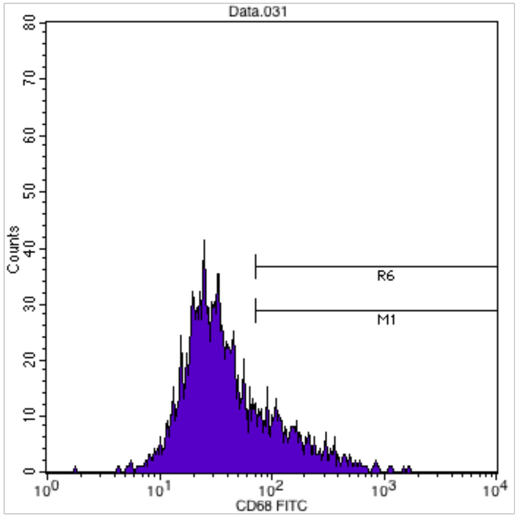

Quadrant Statistics

File: Data.031
Acquisition Date: 16-Oct-24
Gated Events: 925
X Parameter: CD68 FITC (Log)
Quad Location: 66, 90

Sample ID: 1.3
Gate: G7
Total Events: 69756
Y Parameter: IL6 PE (Log)

Quad	Events	% Gated	% Total
UL	0	0.00	0.00
UR	175	18.92	0.25
LL	0	0.00	0.00
LR	750	81.08	1.08

Quadrant Statistics

File: Data.031
Acquisition Date: 16-Oct-24
Gated Events: 925
X Parameter: CD68 FITC (Log)
Quad Location: 66, 50

Sample ID: 1.3
Gate: G7
Total Events: 69756
Y Parameter: NFkB PE-Cy5 (Log)

Quad	Events	% Gated	% Total
UL	0	0.00	0.00
UR	226	24.43	0.32
LL	0	0.00	0.00
LR	699	75.57	1.00

Quadrant Statistics

File: Data.031
Acquisition Date: 16-Oct-24
Gated Events: 925
X Parameter: IL6 PE (Log)
Quad Location: 90, 50

Sample ID: 1.3
Gate: G7
Total Events: 69756
Y Parameter: NFkB PE-Cy5 (Log)

Quad	Events	% Gated	% Total
UL	58	6.27	0.08
UR	168	18.16	0.24
LL	692	74.81	0.99
LR	7	0.76	0.01

Histogram Statistics

File: Data.031
Acquisition Date: 16-Oct-24
Gated Events: 4444
X Parameter: CD68 FITC (Log)

Sample ID: 1.3
Gate: G2
Total Events: 69756

Marker	Left, Right	Events	% Gated	% Total
All	1, 9910	4444	100.00	6.37
M1	72, 9910	925	20.81	1.33

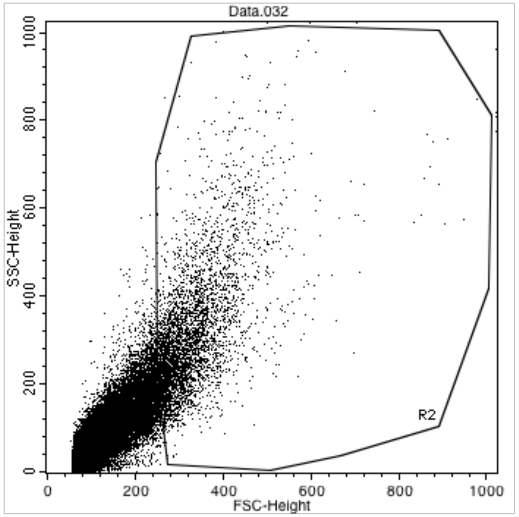

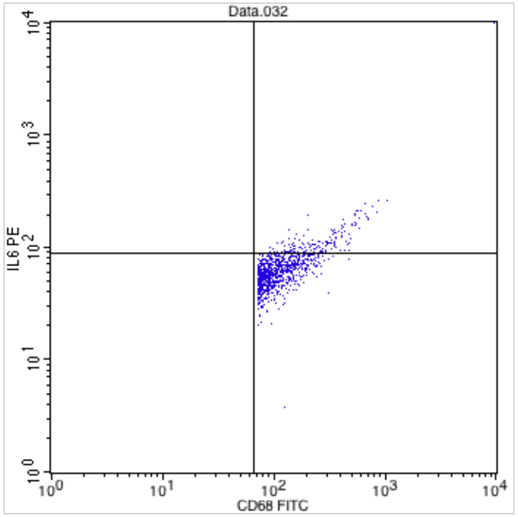

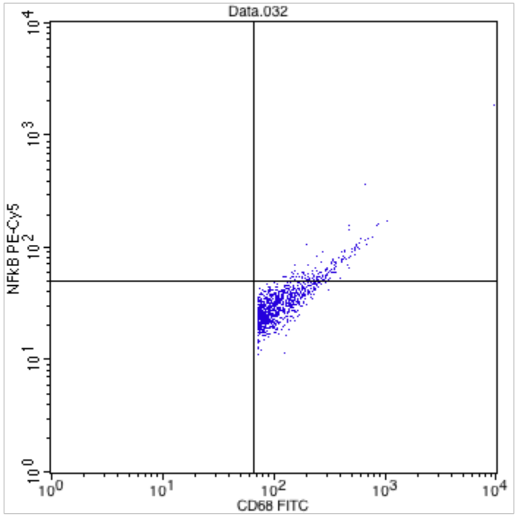

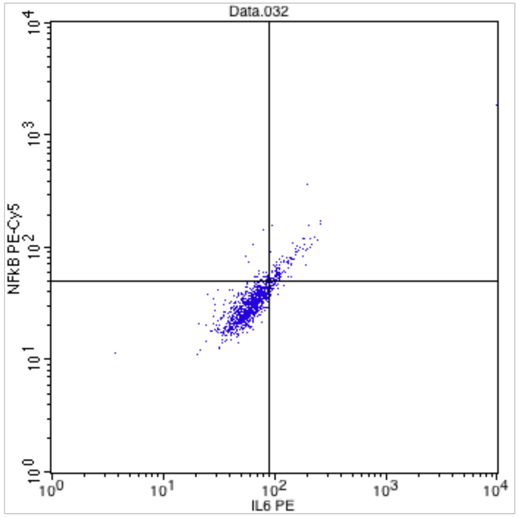

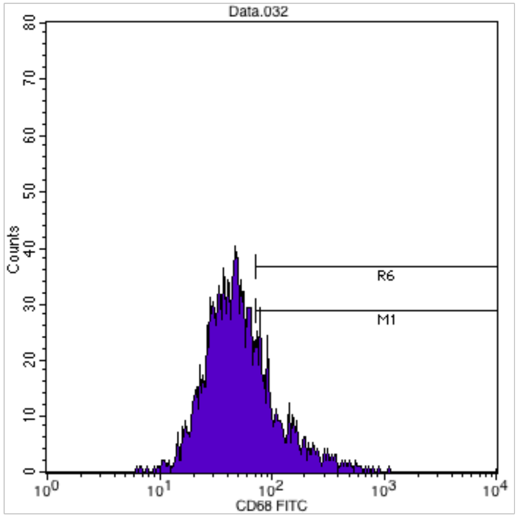

Quadrant Statistics

File: Data.032 Sample ID: 2.1
Acquisition Date: 16-Oct-24 Gate: G7
Gated Events: 1081 Total Events: 77236
X Parameter: CD68 FITC (Log) Y Parameter: IL6 PE (Log)
Quad Location: 66, 90

Quad	Events	% Gated	% Total
UL	0	0.00	0.00
UR	149	13.78	0.19
LL	0	0.00	0.00
LR	932	86.22	1.21

Quadrant Statistics

File: Data.032 Sample ID: 2.1
Acquisition Date: 16-Oct-24 Gate: G7
Gated Events: 1081 Total Events: 77236
X Parameter: CD68 FITC (Log) Y Parameter: NFkB PE-Cy5 (Log)
Quad Location: 66, 50

Quad	Events	% Gated	% Total
UL	0	0.00	0.00
UR	141	13.04	0.18
LL	0	0.00	0.00
LR	940	86.96	1.22

Quadrant Statistics

File: Data.032 Sample ID: 2.1
Acquisition Date: 16-Oct-24 Gate: G7
Gated Events: 1081 Total Events: 77236
X Parameter: IL6 PE (Log) Y Parameter: NFkB PE-Cy5 (Log)
Quad Location: 90, 50

Quad	Events	% Gated	% Total
UL	35	3.24	0.05
UR	106	9.81	0.14
LL	897	82.98	1.16
LR	43	3.98	0.06

Histogram Statistics

File: Data.032 Sample ID: 2.1
Acquisition Date: 16-Oct-24 Gate: G2
Gated Events: 4539 Total Events: 77236
X Parameter: CD68 FITC (Log)

Marker	Left, Right	Events	% Gated	% Total
All	1, 9910	4539	100.00	5.88
M1	72, 9910	1081	23.82	1.40

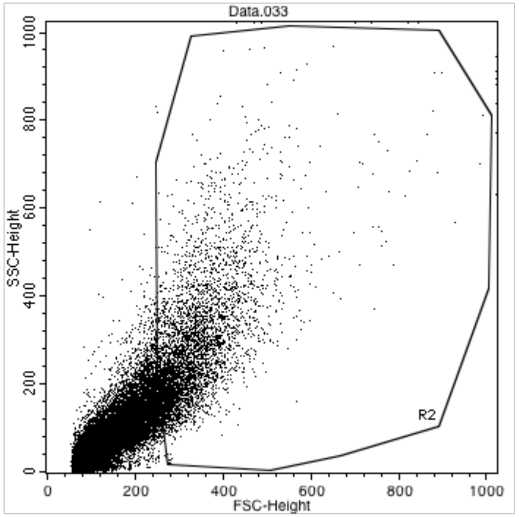

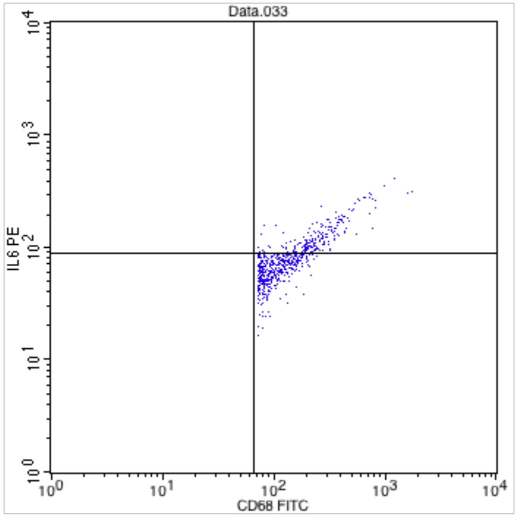

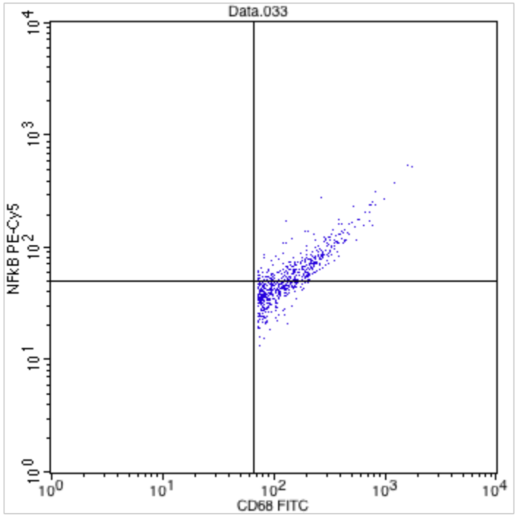

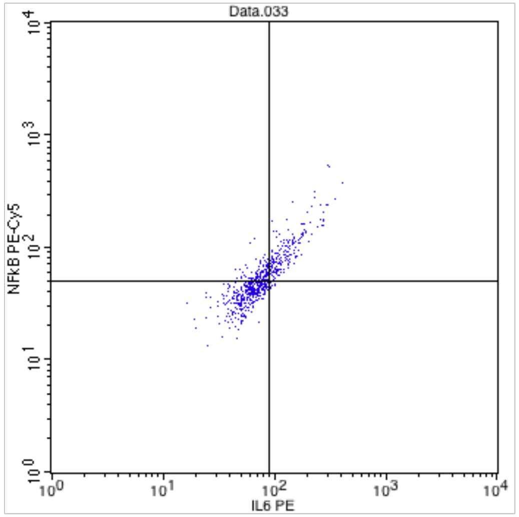

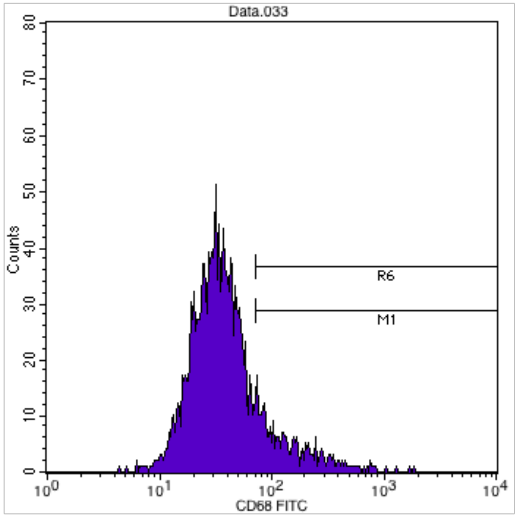

Quadrant Statistics

File: Data.033
Sample ID: 2.2
Acquisition Date: 16-Oct-24
Gate: G7
Gated Events: 653
Total Events: 61684
X Parameter: CD68 FITC (Log)
Y Parameter: IL6 PE (Log)
Quad Location: 66, 90

Quad	Events	% Gated	% Total
UL	0	0.00	0.00
UR	191	29.25	0.31
LL	0	0.00	0.00
LR	462	70.75	0.75

Quadrant Statistics

File: Data.033
Sample ID: 2.2
Acquisition Date: 16-Oct-24
Gate: G7
Gated Events: 653
Total Events: 61684
X Parameter: CD68 FITC (Log)
Y Parameter: NFkB PE-Cy5 (Log)
Quad Location: 66, 50

Quad	Events	% Gated	% Total
UL	0	0.00	0.00
UR	303	46.40	0.49
LL	0	0.00	0.00
LR	350	53.60	0.57

Quadrant Statistics

File: Data.033
Sample ID: 2.2
Acquisition Date: 16-Oct-24
Gate: G7
Gated Events: 653
Total Events: 61684
X Parameter: IL6 PE (Log)
Y Parameter: NFkB PE-Cy5 (Log)
Quad Location: 90, 50

Quad	Events	% Gated	% Total
UL	119	18.22	0.19
UR	184	28.18	0.30
LL	343	52.53	0.56
LR	7	1.07	0.01

Histogram Statistics

File: Data.033
Sample ID: 2.2
Acquisition Date: 16-Oct-24
Gate: G2
Gated Events: 4886
Total Events: 61684
X Parameter: CD68 FITC (Log)

Marker	Left, Right	Events	% Gated	% Total
All	1, 9910	4886	100.00	7.92
M1	72, 9910	653	13.36	1.06

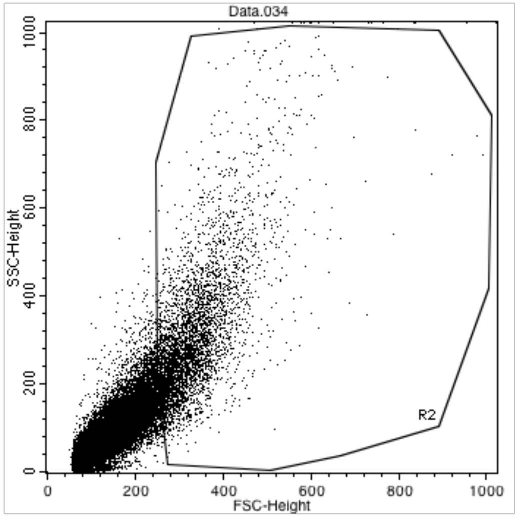

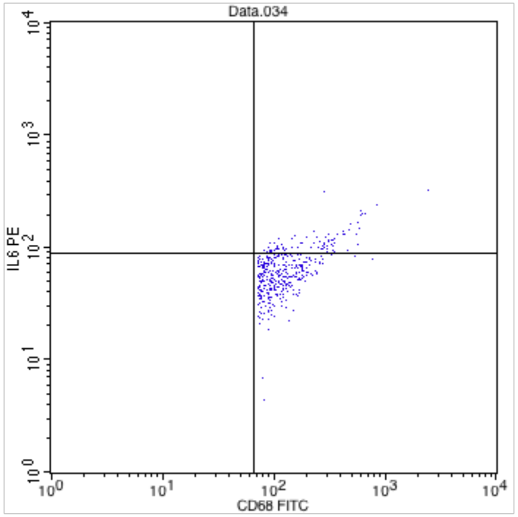

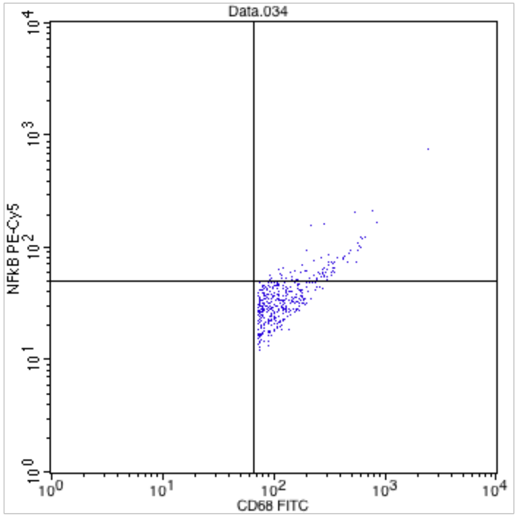

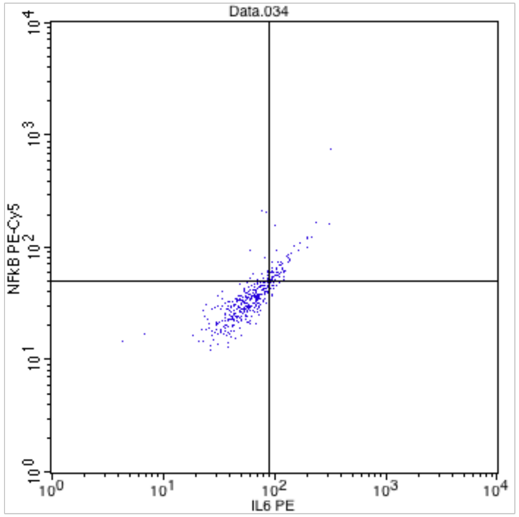

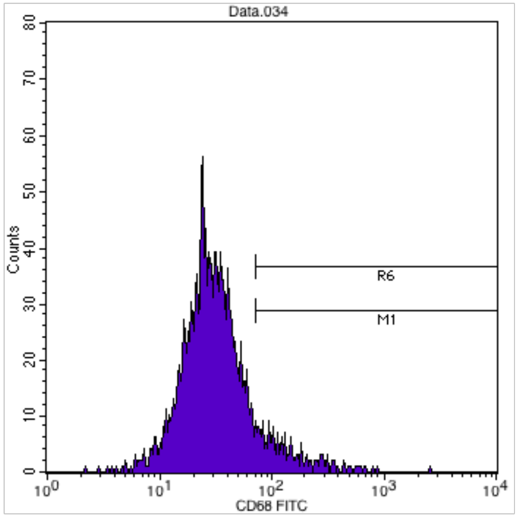

Quadrant Statistics

File: Data.034
Sample ID: 3.1
Acquisition Date: 16-Oct-24
Gate: G7
Gated Events: 413
Total Events: 60397
X Parameter: CD68 FITC (Log)
Y Parameter: IL6 PE (Log)
Quad Location: 66, 90

Quad	Events	% Gated	% Total
UL	0	0.00	0.00
UR	73	17.68	0.12
LL	0	0.00	0.00
LR	340	82.32	0.56

Quadrant Statistics

File: Data.034
Sample ID: 3.1
Acquisition Date: 16-Oct-24
Gate: G7
Gated Events: 413
Total Events: 60397
X Parameter: CD68 FITC (Log)
Y Parameter: NFkB PE-Cy5 (Log)
Quad Location: 66, 50

Quad	Events	% Gated	% Total
UL	0	0.00	0.00
UR	75	18.16	0.12
LL	0	0.00	0.00
LR	338	81.84	0.56

Quadrant Statistics

File: Data.034
Sample ID: 3.1
Acquisition Date: 16-Oct-24
Gate: G7
Gated Events: 413
Total Events: 60397
X Parameter: IL6 PE (Log)
Y Parameter: NFkB PE-Cy5 (Log)
Quad Location: 90, 50

Quad	Events	% Gated	% Total
UL	15	3.63	0.02
UR	60	14.53	0.10
LL	325	78.69	0.54
LR	13	3.15	0.02

Histogram Statistics

File: Data.034
Sample ID: 3.1
Acquisition Date: 16-Oct-24
Gate: G2
Gated Events: 4786
Total Events: 60397
X Parameter: CD68 FITC (Log)

Marker	Left, Right	Events	% Gated	% Total
All	1, 9910	4786	100.00	7.92
M1	72, 9910	413	8.63	0.68

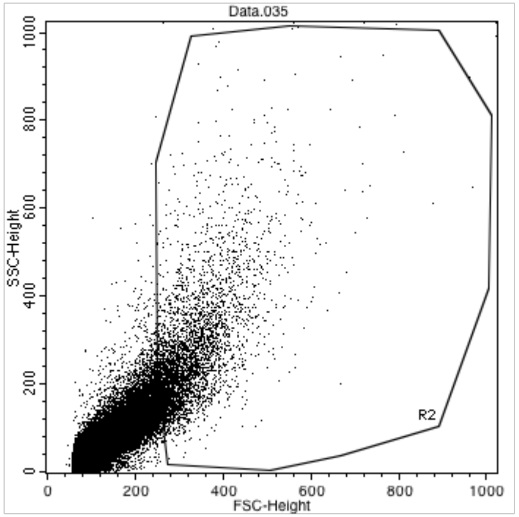

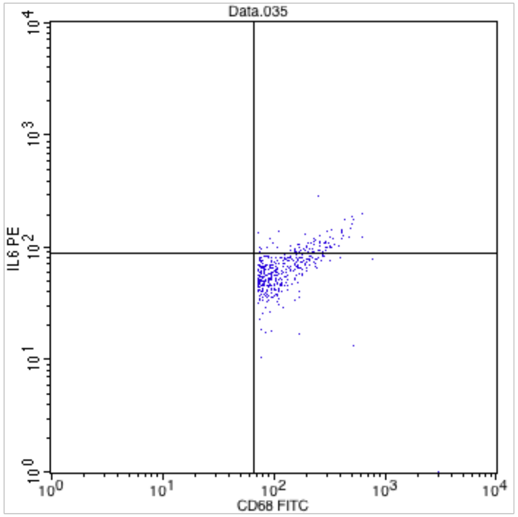

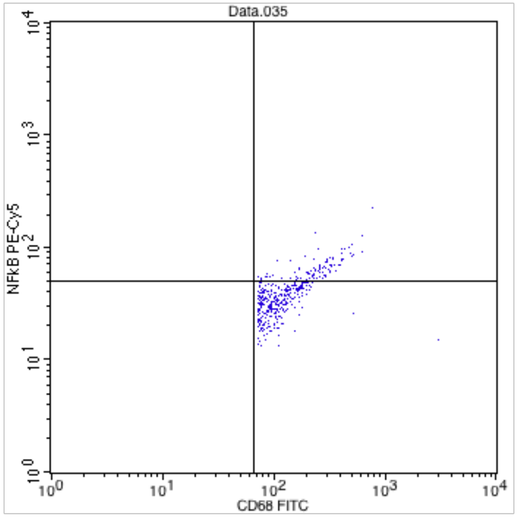

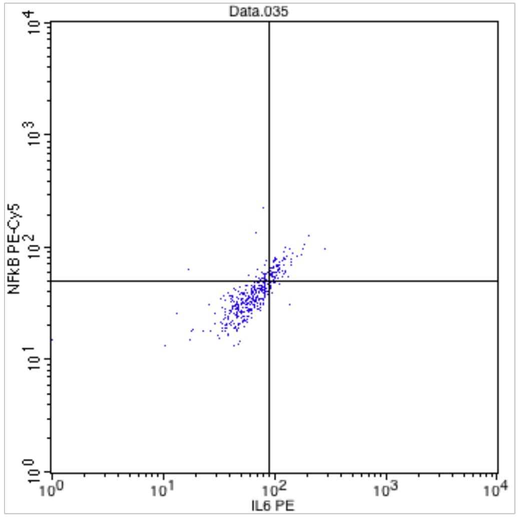

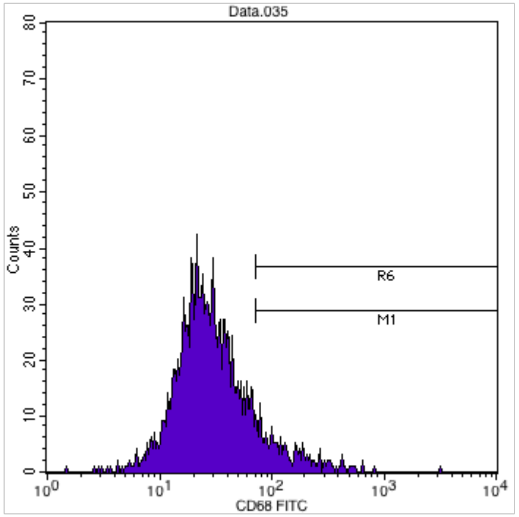

Quadrant Statistics

File: Data.035
Acquisition Date: 16-Oct-24
Gated Events: 433
X Parameter: CD68 FITC (Log)
Quad Location: 66, 90
Sample ID: 3.2
Gate: G7
Total Events: 66943
Y Parameter: IL6 PE (Log)

Quad	Events	% Gated	% Total
UL	0	0.00	0.00
UR	77	17.78	0.12
LL	0	0.00	0.00
LR	356	82.22	0.53

Quadrant Statistics

File: Data.035
Acquisition Date: 16-Oct-24
Gated Events: 433
X Parameter: CD68 FITC (Log)
Quad Location: 66, 50
Sample ID: 3.2
Gate: G7
Total Events: 66943
Y Parameter: NFkB PE-Cy5 (Log)

Quad	Events	% Gated	% Total
UL	0	0.00	0.00
UR	98	22.63	0.15
LL	0	0.00	0.00
LR	335	77.37	0.50

Quadrant Statistics

File: Data.035
Acquisition Date: 16-Oct-24
Gated Events: 433
X Parameter: IL6 PE (Log)
Quad Location: 90, 50
Sample ID: 3.2
Gate: G7
Total Events: 66943
Y Parameter: NFkB PE-Cy5 (Log)

Quad	Events	% Gated	% Total
UL	32	7.39	0.05
UR	66	15.24	0.10
LL	324	74.83	0.48
LR	11	2.54	0.02

Histogram Statistics

File: Data.035
Acquisition Date: 16-Oct-24
Gated Events: 4322
X Parameter: CD68 FITC (Log)
Sample ID: 3.2
Gate: G2
Total Events: 66943

Marker	Left, Right	Events	% Gated	% Total
All	1, 9910	4322	100.00	6.46
M1	72, 9910	433	10.02	0.65

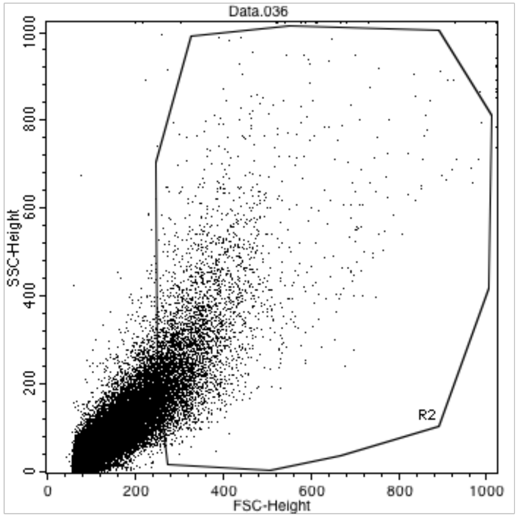

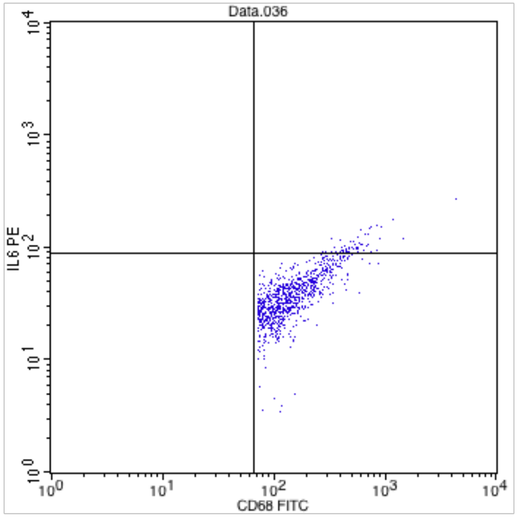

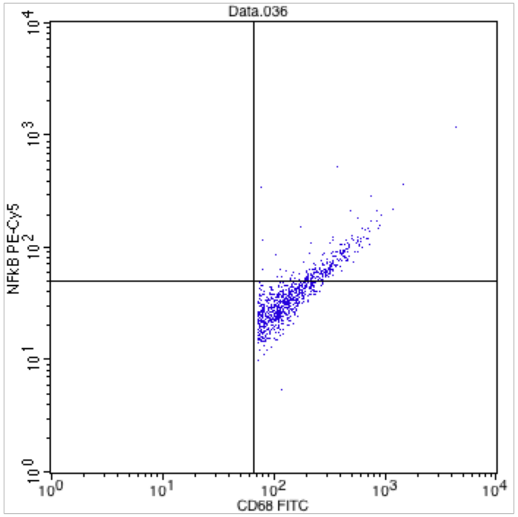

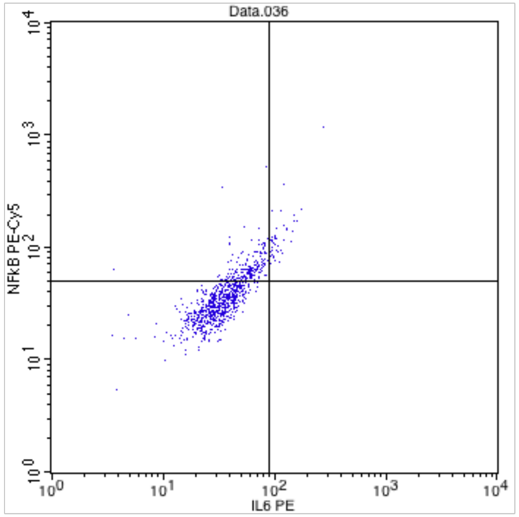

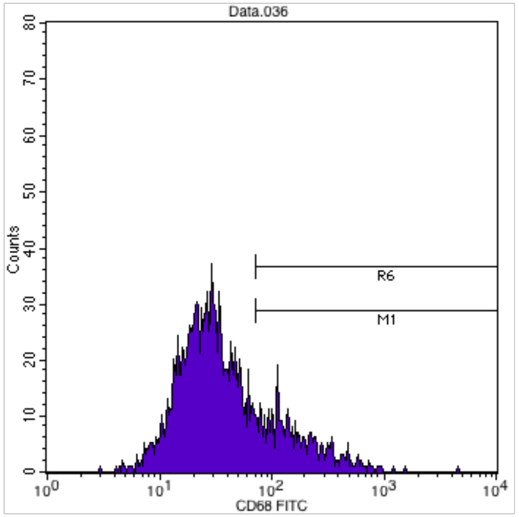

Quadrant Statistics

File: Data.036
Acquisition Date: 16-Oct-24
Gated Events: 945
X Parameter: CD68 FITC (Log)
Quad Location: 66, 90
Sample ID: 3.3
Gate: G7
Total Events: 65410
Y Parameter: IL6 PE (Log)

Quad	Events	% Gated	% Total
UL	0	0.00	0.00
UR	47	4.97	0.07
LL	0	0.00	0.00
LR	898	95.03	1.37

Quadrant Statistics

File: Data.036
Acquisition Date: 16-Oct-24
Gated Events: 945
X Parameter: CD68 FITC (Log)
Quad Location: 66, 50
Sample ID: 3.3
Gate: G7
Total Events: 65410
Y Parameter: NFkB PE-Cy5 (Log)

Quad	Events	% Gated	% Total
UL	0	0.00	0.00
UR	242	25.61	0.37
LL	0	0.00	0.00
LR	703	74.39	1.07

Quadrant Statistics

File: Data.036
Acquisition Date: 16-Oct-24
Gated Events: 945
X Parameter: IL6 PE (Log)
Quad Location: 90, 50
Sample ID: 3.3
Gate: G7
Total Events: 65410
Y Parameter: NFkB PE-Cy5 (Log)

Quad	Events	% Gated	% Total
UL	195	20.63	0.30
UR	47	4.97	0.07
LL	703	74.39	1.07
LR	0	0.00	0.00

Histogram Statistics

File: Data.036
Acquisition Date: 16-Oct-24
Gated Events: 4549
X Parameter: CD68 FITC (Log)
Sample ID: 3.3
Gate: G2
Total Events: 65410

Marker	Left, Right	Events	% Gated	% Total
All	1, 9910	4549	100.00	6.95
M1	72, 9910	945	20.77	1.44

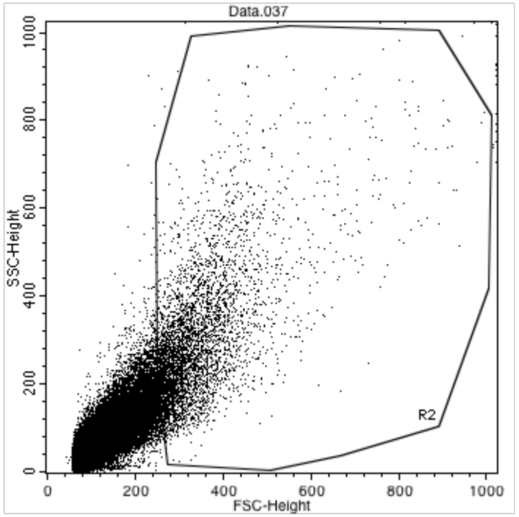

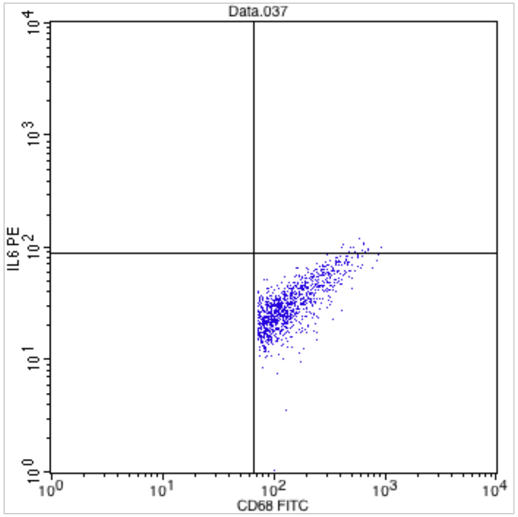

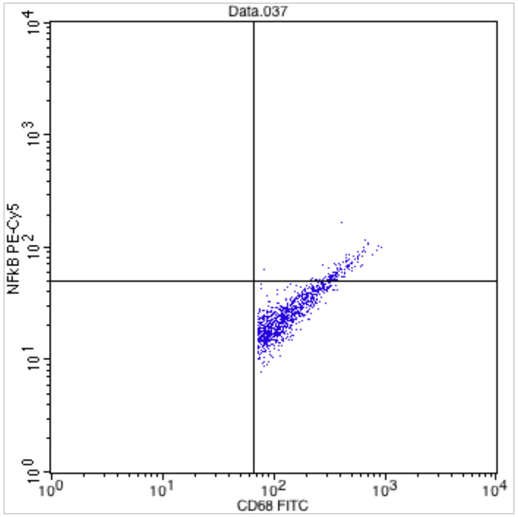

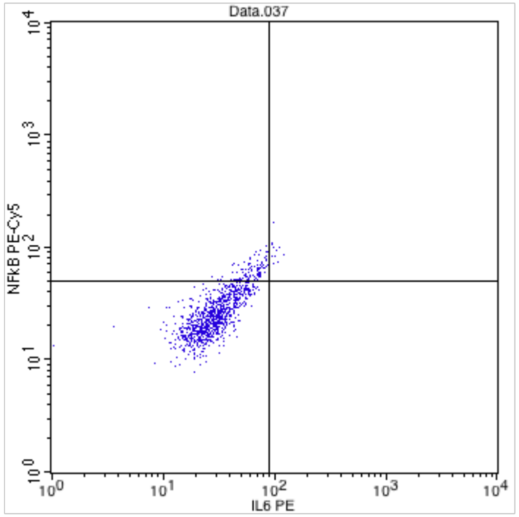

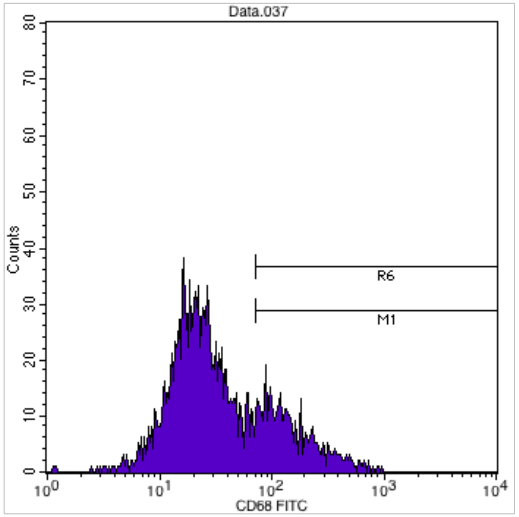

Quadrant Statistics

File: Data.037
Acquisition Date: 16-Oct-24
Gated Events: 1115
X Parameter: CD68 FITC (Log)
Quad Location: 66, 90
Sample ID: 4.1
Gate: G7
Total Events: 69344
Y Parameter: IL6 PE (Log)

Quad	Events	% Gated	% Total
UL	0	0.00	0.00
UR	13	1.17	0.02
LL	0	0.00	0.00
LR	1102	98.83	1.59

Quadrant Statistics

File: Data.037
Acquisition Date: 16-Oct-24
Gated Events: 1115
X Parameter: CD68 FITC (Log)
Quad Location: 66, 50
Sample ID: 4.1
Gate: G7
Total Events: 69344
Y Parameter: NFkB PE-Cy5 (Log)

Quad	Events	% Gated	% Total
UL	0	0.00	0.00
UR	120	10.76	0.17
LL	0	0.00	0.00
LR	995	89.24	1.43

Quadrant Statistics

File: Data.037
Acquisition Date: 16-Oct-24
Gated Events: 1115
X Parameter: IL6 PE (Log)
Quad Location: 90, 50
Sample ID: 4.1
Gate: G7
Total Events: 69344
Y Parameter: NFkB PE-Cy5 (Log)

Quad	Events	% Gated	% Total
UL	107	9.60	0.15
UR	13	1.17	0.02
LL	995	89.24	1.43
LR	0	0.00	0.00

Histogram Statistics

File: Data.037
Acquisition Date: 16-Oct-24
Gated Events: 4804
X Parameter: CD68 FITC (Log)
Sample ID: 4.1
Gate: G2
Total Events: 69344

Marker	Left, Right	Events	% Gated	% Total
All	1, 9910	4804	100.00	6.93
M1	72, 9910	1115	23.21	1.61

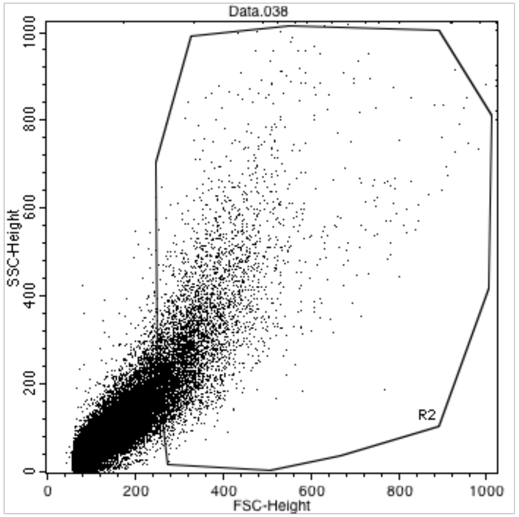

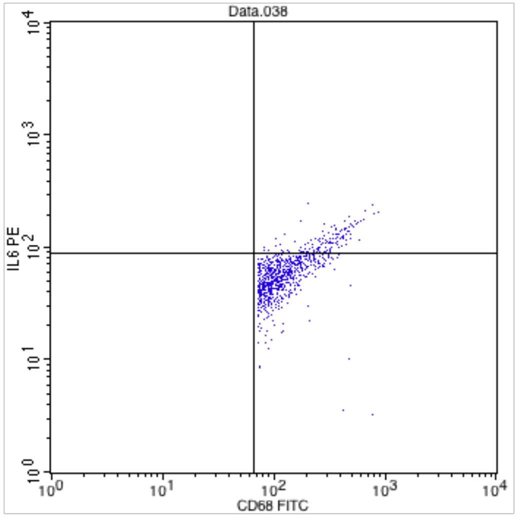

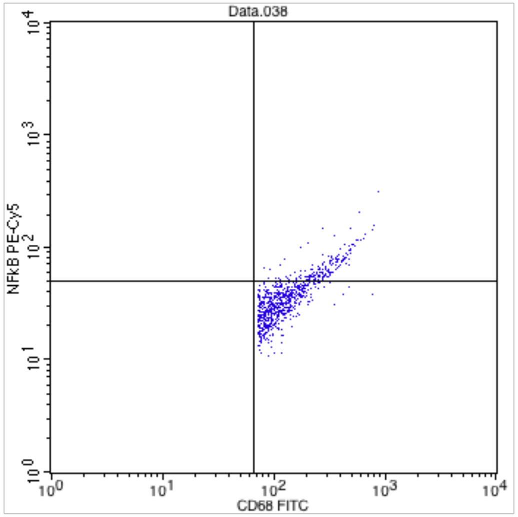

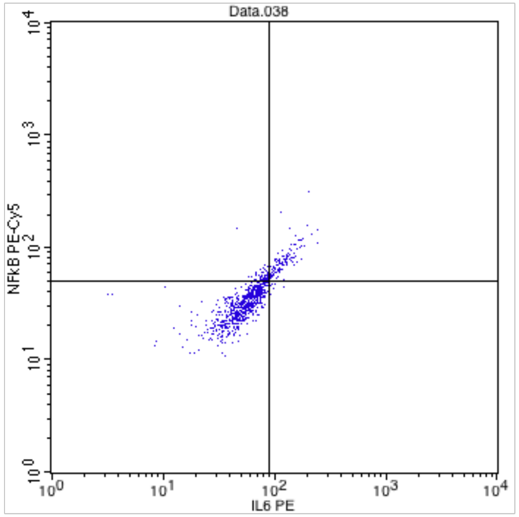

Quadrant Statistics

File: Data.038
Acquisition Date: 16-Oct-24
Gated Events: 844
X Parameter: CD68 FITC (Log)
Quad Location: 66, 90
Sample ID: 4.2
Gate: G7
Total Events: 58938
Y Parameter: IL6 PE (Log)

Quad	Events	% Gated	% Total
UL	0	0.00	0.00
UR	124	14.69	0.21
LL	0	0.00	0.00
LR	720	85.31	1.22

Quadrant Statistics

File: Data.038
Acquisition Date: 16-Oct-24
Gated Events: 844
X Parameter: CD68 FITC (Log)
Quad Location: 66, 50
Sample ID: 4.2
Gate: G7
Total Events: 58938
Y Parameter: NFkB PE-Cy5 (Log)

Quad	Events	% Gated	% Total
UL	0	0.00	0.00
UR	177	20.97	0.30
LL	0	0.00	0.00
LR	667	79.03	1.13

Quadrant Statistics

File: Data.038
Acquisition Date: 16-Oct-24
Gated Events: 844
X Parameter: IL6 PE (Log)
Quad Location: 90, 50
Sample ID: 4.2
Gate: G7
Total Events: 58938
Y Parameter: NFkB PE-Cy5 (Log)

Quad	Events	% Gated	% Total
UL	62	7.35	0.11
UR	115	13.63	0.20
LL	658	77.96	1.12
LR	9	1.07	0.02

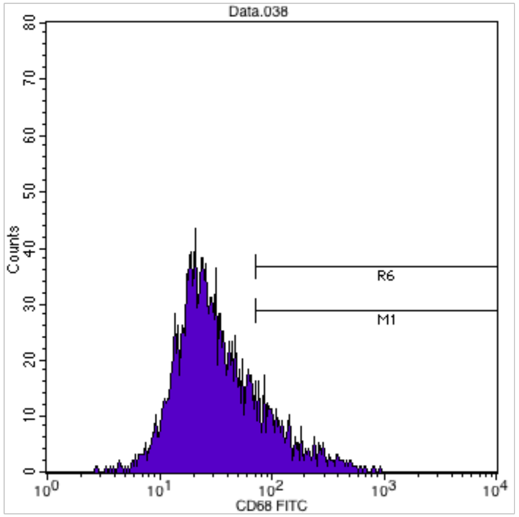

Histogram Statistics

File: Data.038
Acquisition Date: 16-Oct-24
Gated Events: 5237
X Parameter: CD68 FITC (Log)
Sample ID: 4.2
Gate: G2
Total Events: 58938

Marker	Left, Right	Events	% Gated	% Total
All	1, 9910	5237	100.00	8.89
M1	72, 9910	844	16.12	1.43

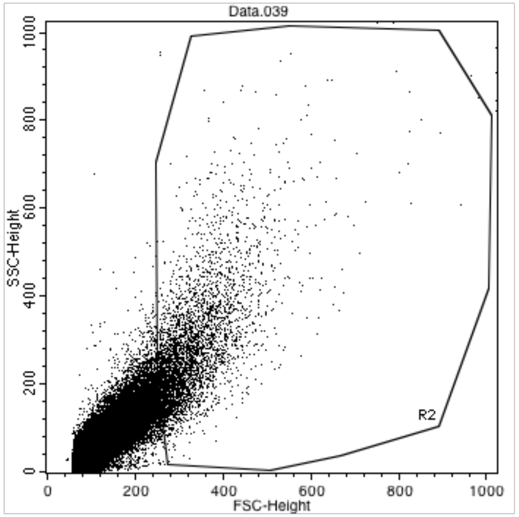

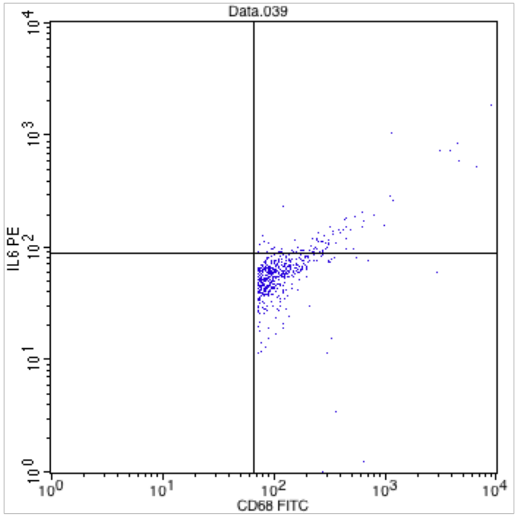

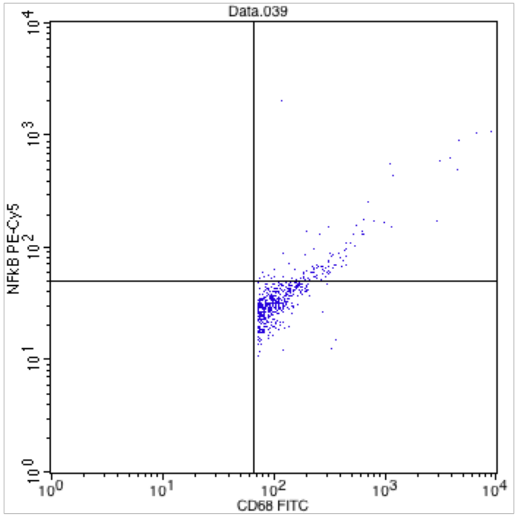

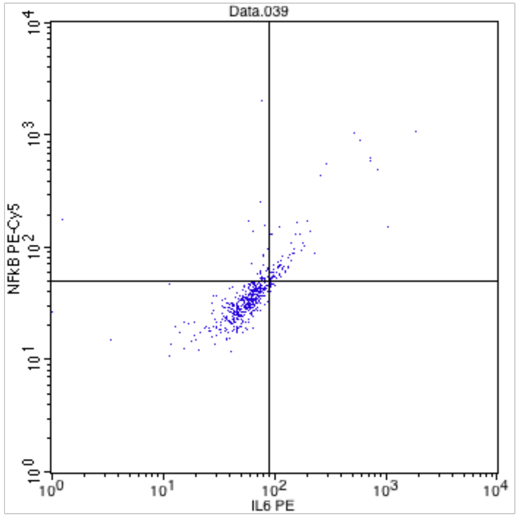

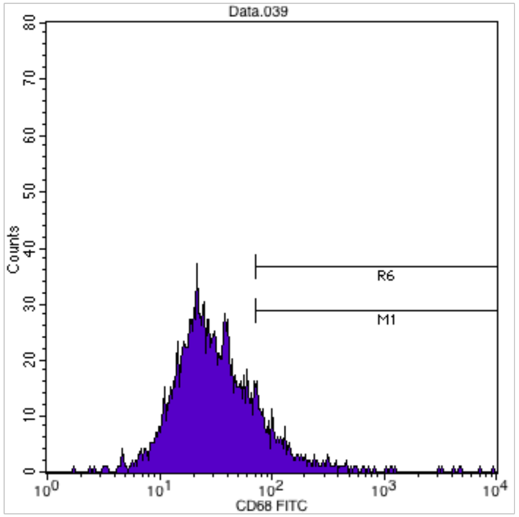

Quadrant Statistics

File: Data.039
Acquisition Date: 16-Oct-24
Gated Events: 537
X Parameter: CD68 FITC (Log)
Quad Location: 66, 90

Sample ID: 4.3
Gate: G7
Total Events: 79025
Y Parameter: IL6 PE (Log)

Quad	Events	% Gated	% Total
UL	0	0.00	0.00
UR	75	13.97	0.09
LL	0	0.00	0.00
LR	462	86.03	0.58

Quadrant Statistics

File: Data.039
Acquisition Date: 16-Oct-24
Gated Events: 537
X Parameter: CD68 FITC (Log)
Quad Location: 66, 50

Sample ID: 4.3
Gate: G7
Total Events: 79025
Y Parameter: NFkB PE-Cy5 (Log)

Quad	Events	% Gated	% Total
UL	0	0.00	0.00
UR	96	17.88	0.12
LL	0	0.00	0.00
LR	441	82.12	0.56

Quadrant Statistics

File: Data.039
Acquisition Date: 16-Oct-24
Gated Events: 537
X Parameter: IL6 PE (Log)
Quad Location: 90, 50

Sample ID: 4.3
Gate: G7
Total Events: 79025
Y Parameter: NFkB PE-Cy5 (Log)

Quad	Events	% Gated	% Total
UL	33	6.15	0.04
UR	63	11.73	0.08
LL	429	79.89	0.54
LR	12	2.23	0.02

Histogram Statistics

File: Data.039
Acquisition Date: 16-Oct-24
Gated Events: 4259
X Parameter: CD68 FITC (Log)

Sample ID: 4.3
Gate: G2
Total Events: 79025

Marker	Left, Right	Events	% Gated	% Total
All	1, 9910	4259	100.00	5.39
M1	72, 9910	537	12.61	0.68

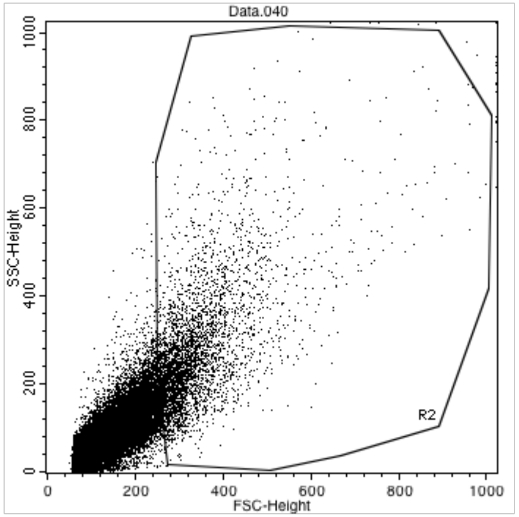

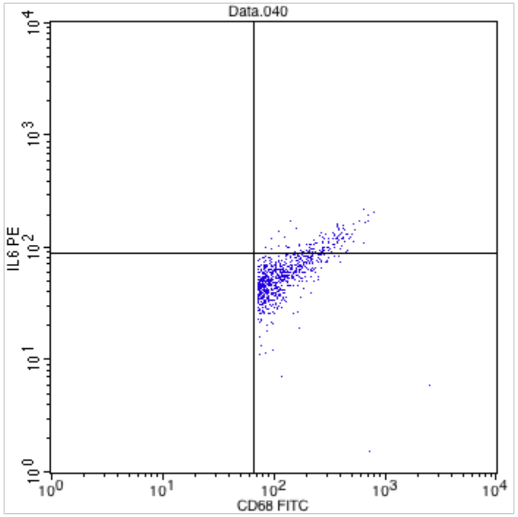

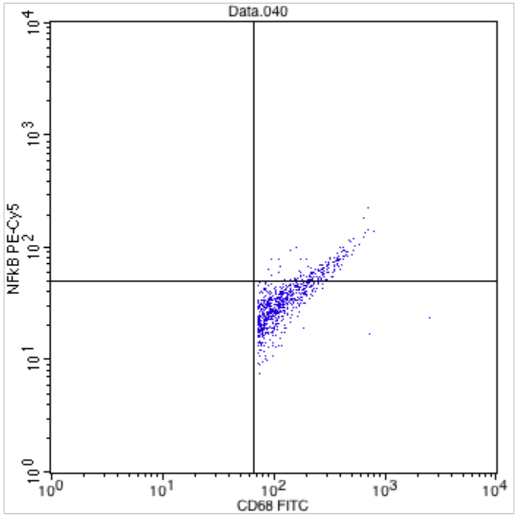

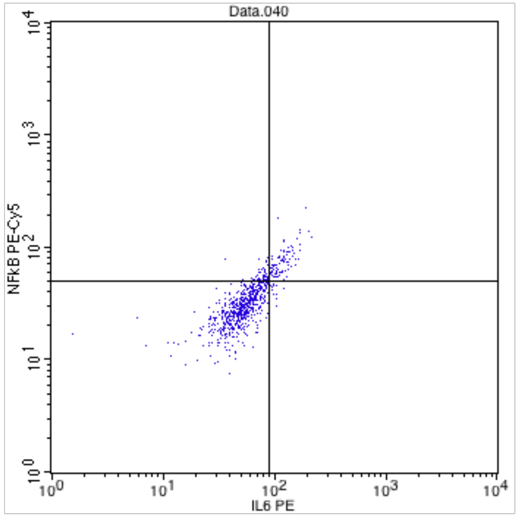

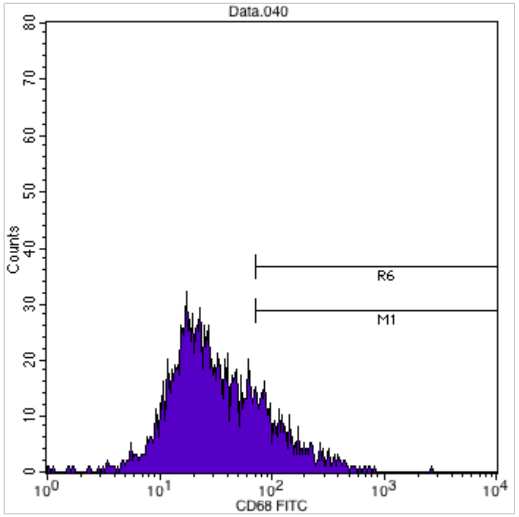

Quadrant Statistics

File: Data.040
Acquisition Date: 16-Oct-24
Gated Events: 808
X Parameter: CD68 FITC (Log)
Quad Location: 66, 90

Sample ID: 5.1
Gate: G7
Total Events: 68318
Y Parameter: IL6 PE (Log)

Quad	Events	% Gated	% Total
UL	0	0.00	0.00
UR	107	13.24	0.16
LL	0	0.00	0.00
LR	701	86.76	1.03

Quadrant Statistics

File: Data.040
Acquisition Date: 16-Oct-24
Gated Events: 808
X Parameter: CD68 FITC (Log)
Quad Location: 66, 50

Sample ID: 5.1
Gate: G7
Total Events: 68318
Y Parameter: NFkB PE-Cy5 (Log)

Quad	Events	% Gated	% Total
UL	0	0.00	0.00
UR	147	18.19	0.22
LL	0	0.00	0.00
LR	661	81.81	0.97

Quadrant Statistics

File: Data.040
Acquisition Date: 16-Oct-24
Gated Events: 808
X Parameter: IL6 PE (Log)
Quad Location: 90, 50

Sample ID: 5.1
Gate: G7
Total Events: 68318
Y Parameter: NFkB PE-Cy5 (Log)

Quad	Events	% Gated	% Total
UL	57	7.05	0.08
UR	90	11.14	0.13
LL	644	79.70	0.94
LR	17	2.10	0.02

Histogram Statistics

File: Data.040
Acquisition Date: 16-Oct-24
Gated Events: 4291
X Parameter: CD68 FITC (Log)

Sample ID: 5.1
Gate: G2
Total Events: 68318

Marker	Left, Right	Events	% Gated	% Total
All	1, 9910	4291	100.00	6.28
M1	72, 9910	808	18.83	1.18

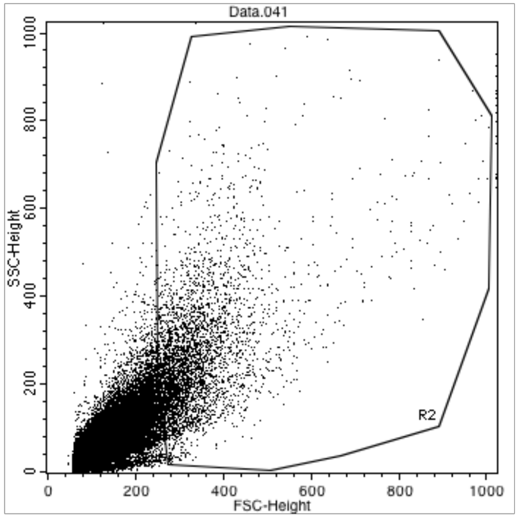

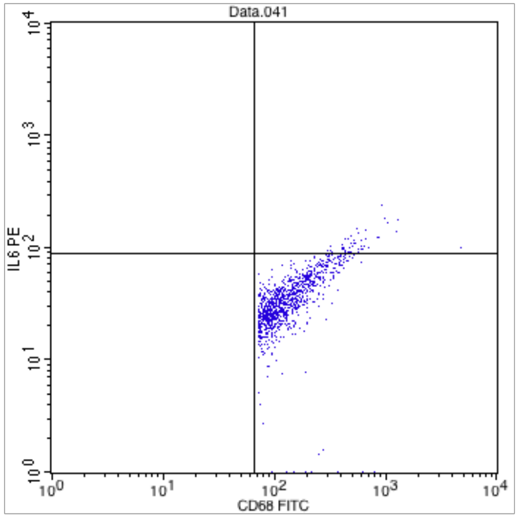

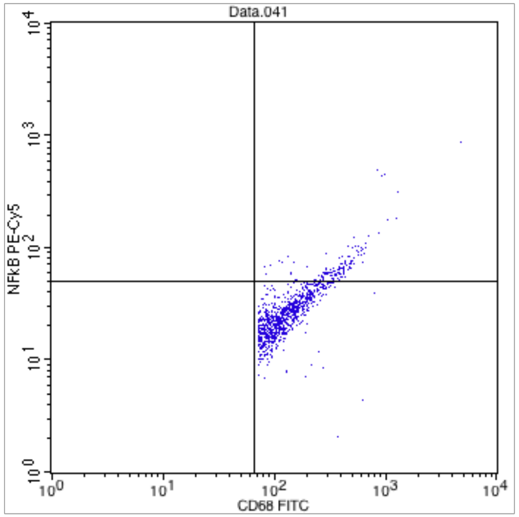

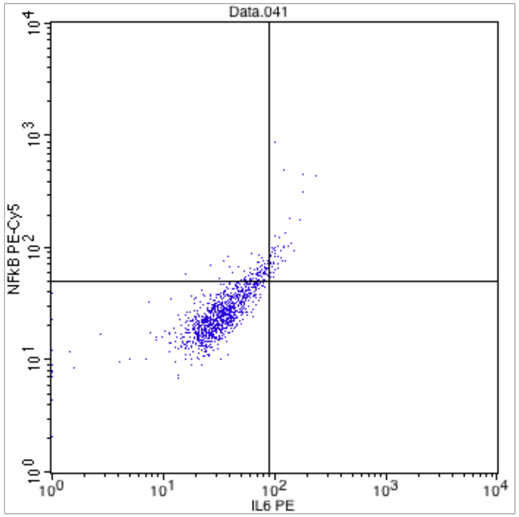

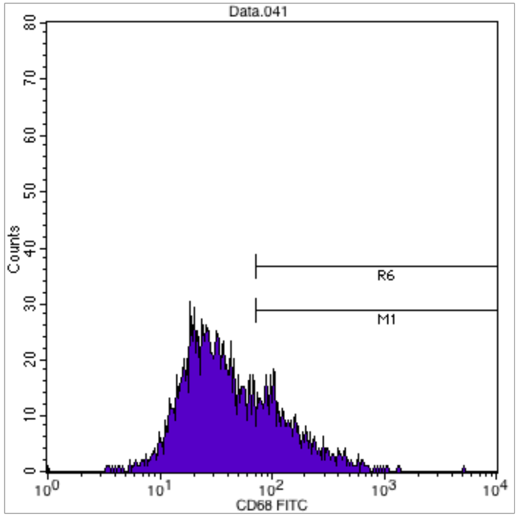

Quadrant Statistics

File: Data.041
Acquisition Date: 16-Oct-24
Gated Events: 1085
X Parameter: CD68 FITC (Log)
Quad Location: 66, 90
Sample ID: 5.2
Gate: G7
Total Events: 64188
Y Parameter: IL6 PE (Log)

Quad	Events	% Gated	% Total
UL	0	0.00	0.00
UR	44	4.06	0.07
LL	0	0.00	0.00
LR	1041	95.94	1.62

Quadrant Statistics

File: Data.041
Acquisition Date: 16-Oct-24
Gated Events: 1085
X Parameter: CD68 FITC (Log)
Quad Location: 66, 50
Sample ID: 5.2
Gate: G7
Total Events: 64188
Y Parameter: NFkB PE-Cy5 (Log)

Quad	Events	% Gated	% Total
UL	0	0.00	0.00
UR	127	11.71	0.20
LL	0	0.00	0.00
LR	958	88.29	1.49

Quadrant Statistics

File: Data.041
Acquisition Date: 16-Oct-24
Gated Events: 1085
X Parameter: IL6 PE (Log)
Quad Location: 90, 50
Sample ID: 5.2
Gate: G7
Total Events: 64188
Y Parameter: NFkB PE-Cy5 (Log)

Quad	Events	% Gated	% Total
UL	83	7.65	0.13
UR	44	4.06	0.07
LL	958	88.29	1.49
LR	0	0.00	0.00

Histogram Statistics

File: Data.041
Acquisition Date: 16-Oct-24
Gated Events: 4231
X Parameter: CD68 FITC (Log)
Sample ID: 5.2
Gate: G2
Total Events: 64188

Marker	Left, Right	Events	% Gated	% Total
All	1, 9910	4231	100.00	6.59
M1	72, 9910	1085	25.64	1.69

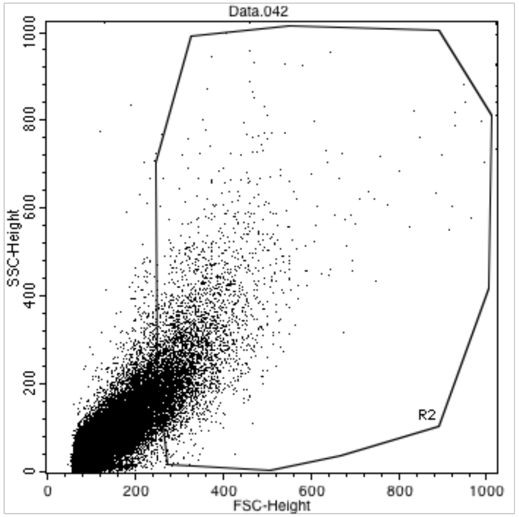

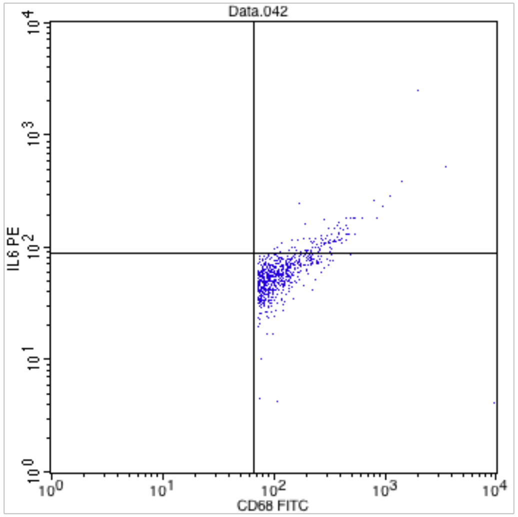

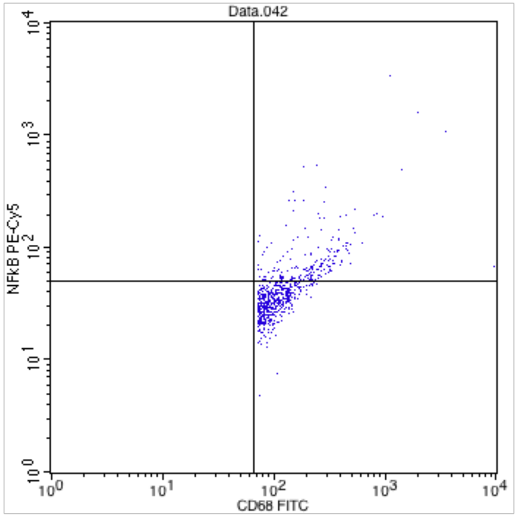

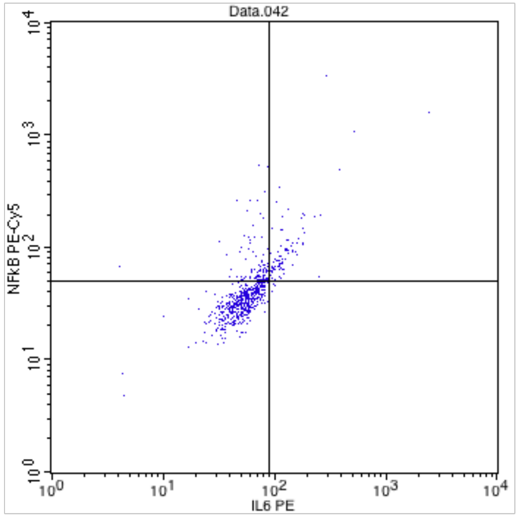

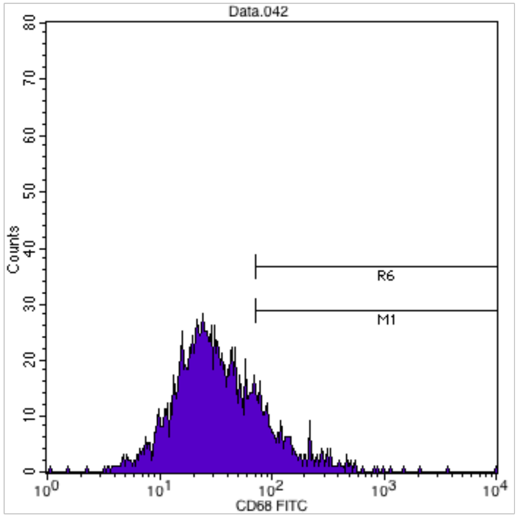

Quadrant Statistics

File: Data.042
Acquisition Date: 16-Oct-24
Gated Events: 693
X Parameter: CD68 FITC (Log)
Quad Location: 66, 90
Sample ID: 5.3
Gate: G7
Total Events: 70652
Y Parameter: IL6 PE (Log)

Quad	Events	% Gated	% Total
UL	0	0.00	0.00
UR	88	12.70	0.12
LL	0	0.00	0.00
LR	605	87.30	0.86

Quadrant Statistics

File: Data.042
Acquisition Date: 16-Oct-24
Gated Events: 693
X Parameter: CD68 FITC (Log)
Quad Location: 66, 50
Sample ID: 5.3
Gate: G7
Total Events: 70652
Y Parameter: NFkB PE-Cy5 (Log)

Quad	Events	% Gated	% Total
UL	0	0.00	0.00
UR	184	26.55	0.26
LL	0	0.00	0.00
LR	509	73.45	0.72

Quadrant Statistics

File: Data.042
Acquisition Date: 16-Oct-24
Gated Events: 693
X Parameter: IL6 PE (Log)
Quad Location: 90, 50
Sample ID: 5.3
Gate: G7
Total Events: 70652
Y Parameter: NFkB PE-Cy5 (Log)

Quad	Events	% Gated	% Total
UL	100	14.43	0.14
UR	84	12.12	0.12
LL	505	72.87	0.71
LR	4	0.58	0.01

Histogram Statistics

File: Data.042
Acquisition Date: 16-Oct-24
Gated Events: 4199
X Parameter: CD68 FITC (Log)
Sample ID: 5.3
Gate: G2
Total Events: 70652

Marker	Left, Right	Events	% Gated	% Total
All	1, 9910	4199	100.00	5.94
M1	72, 9910	693	16.50	0.98

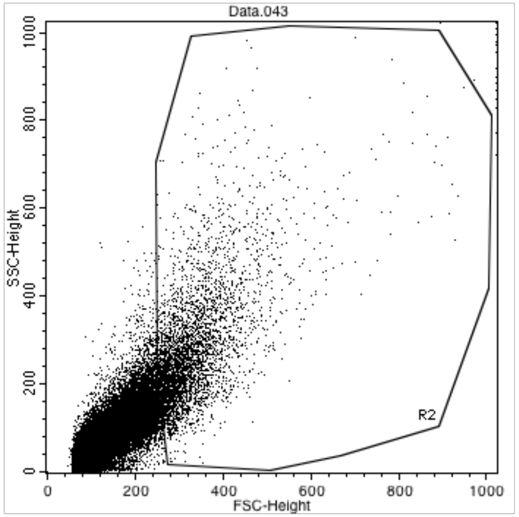

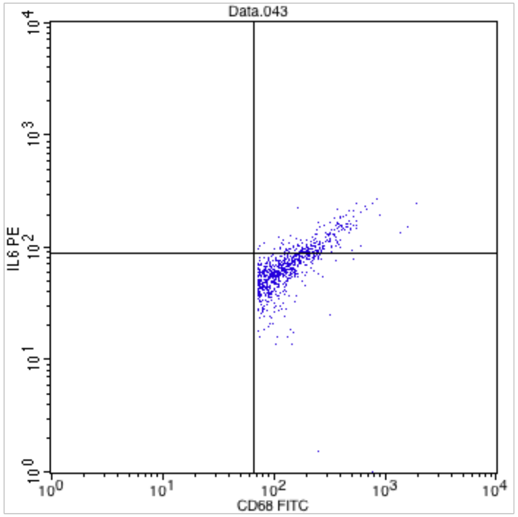

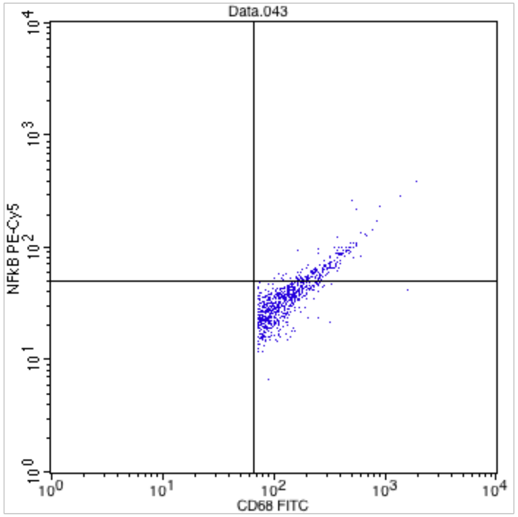

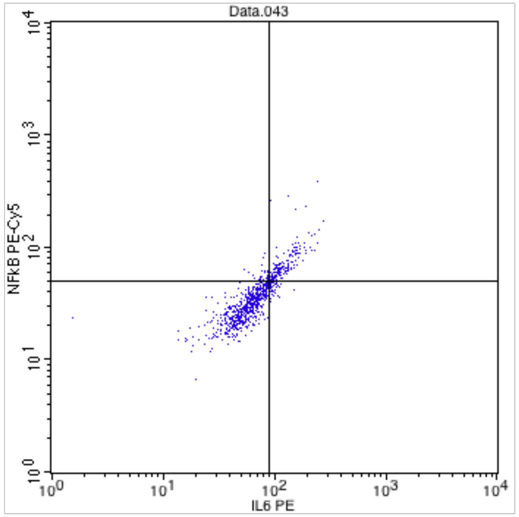

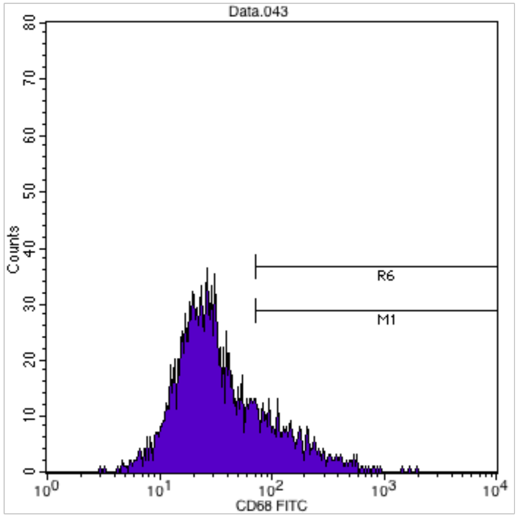

Quadrant Statistics

File: Data.043
Acquisition Date: 16-Oct-24
Gated Events: 848
X Parameter: CD68 FITC (Log)
Quad Location: 66, 90

Sample ID: 6.2
Gate: G7
Total Events: 67880
Y Parameter: IL6 PE (Log)

Quad	Events	% Gated	% Total
UL	0	0.00	0.00
UR	194	22.88	0.29
LL	0	0.00	0.00
LR	654	77.12	0.96

Quadrant Statistics

File: Data.043
Acquisition Date: 16-Oct-24
Gated Events: 848
X Parameter: CD68 FITC (Log)
Quad Location: 66, 50

Sample ID: 6.2
Gate: G7
Total Events: 67880
Y Parameter: NFkB PE-Cy5 (Log)

Quad	Events	% Gated	% Total
UL	0	0.00	0.00
UR	176	20.75	0.26
LL	0	0.00	0.00
LR	672	79.25	0.99

Quadrant Statistics

File: Data.043
Acquisition Date: 16-Oct-24
Gated Events: 848
X Parameter: IL6 PE (Log)
Quad Location: 90, 50

Sample ID: 6.2
Gate: G7
Total Events: 67880
Y Parameter: NFkB PE-Cy5 (Log)

Quad	Events	% Gated	% Total
UL	30	3.54	0.04
UR	146	17.22	0.22
LL	624	73.58	0.92
LR	48	5.66	0.07

Histogram Statistics

File: Data.043
Acquisition Date: 16-Oct-24
Gated Events: 4433
X Parameter: CD68 FITC (Log)

Sample ID: 6.2
Gate: G2
Total Events: 67880

Marker	Left, Right	Events	% Gated	% Total
All	1, 9910	4433	100.00	6.53
M1	72, 9910	848	19.13	1.25

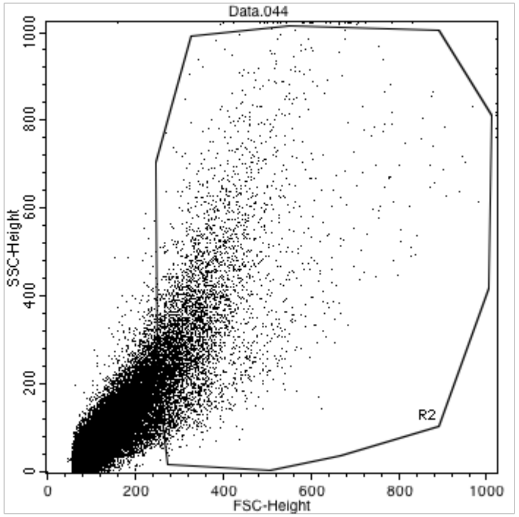

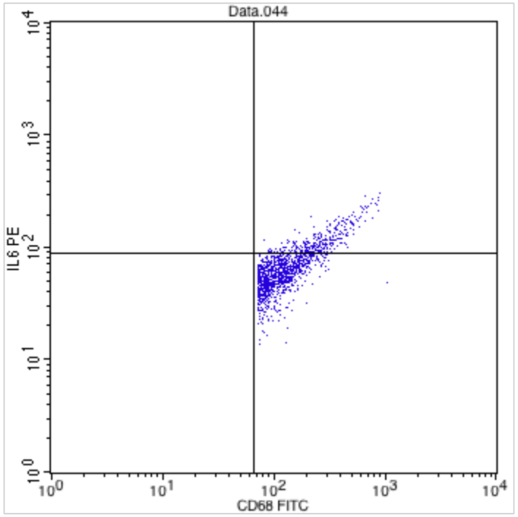

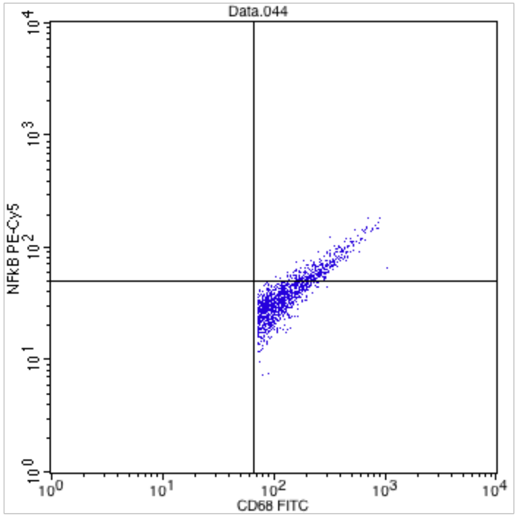

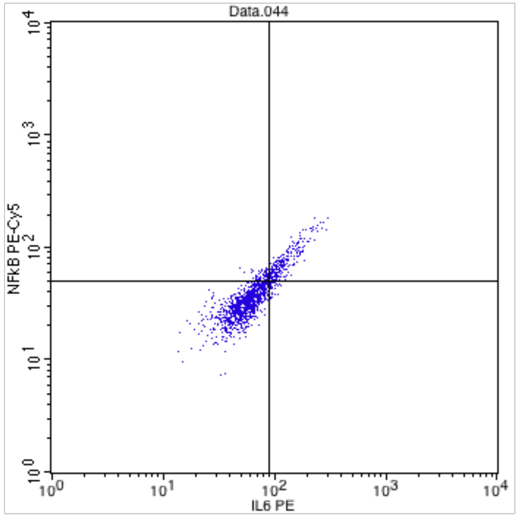

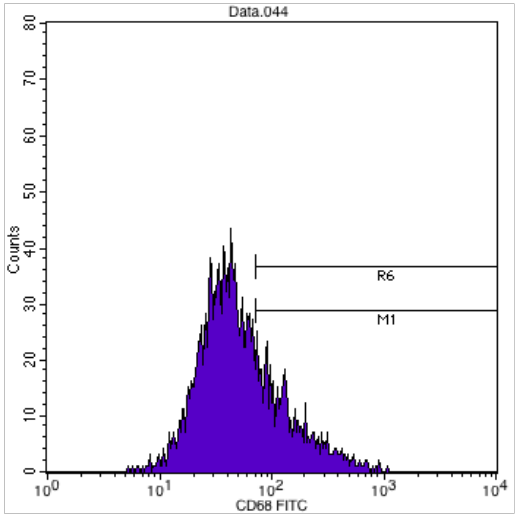

Quadrant Statistics

File: Data.044
Acquisition Date: 16-Oct-24
Gated Events: 1412
X Parameter: CD68 FITC (Log)
Quad Location: 66, 90
Sample ID: 6.3
Gate: G7
Total Events: 53318
Y Parameter: IL6 PE (Log)

Quad	Events	% Gated	% Total
UL	0	0.00	0.00
UR	291	20.61	0.55
LL	0	0.00	0.00
LR	1121	79.39	2.10

Quadrant Statistics

File: Data.044
Acquisition Date: 16-Oct-24
Gated Events: 1412
X Parameter: CD68 FITC (Log)
Quad Location: 66, 50
Sample ID: 6.3
Gate: G7
Total Events: 53318
Y Parameter: NFkB PE-Cy5 (Log)

Quad	Events	% Gated	% Total
UL	0	0.00	0.00
UR	323	22.88	0.61
LL	0	0.00	0.00
LR	1089	77.12	2.04

Quadrant Statistics

File: Data.044
Acquisition Date: 16-Oct-24
Gated Events: 1412
X Parameter: IL6 PE (Log)
Quad Location: 90, 50
Sample ID: 6.3
Gate: G7
Total Events: 53318
Y Parameter: NFkB PE-Cy5 (Log)

Quad	Events	% Gated	% Total
UL	72	5.10	0.14
UR	251	17.78	0.47
LL	1049	74.29	1.97
LR	40	2.83	0.08

Histogram Statistics

File: Data.044
Acquisition Date: 16-Oct-24
Gated Events: 5217
X Parameter: CD68 FITC (Log)
Sample ID: 6.3
Gate: G2
Total Events: 53318

Marker	Left, Right	Events	% Gated	% Total
All	1, 9910	5217	100.00	9.78
M1	72, 9910	1412	27.07	2.65

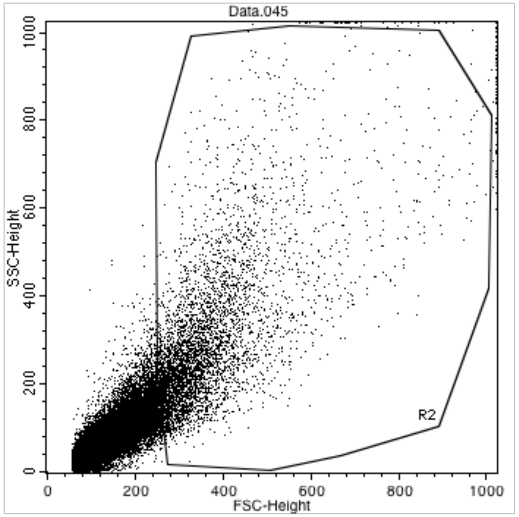

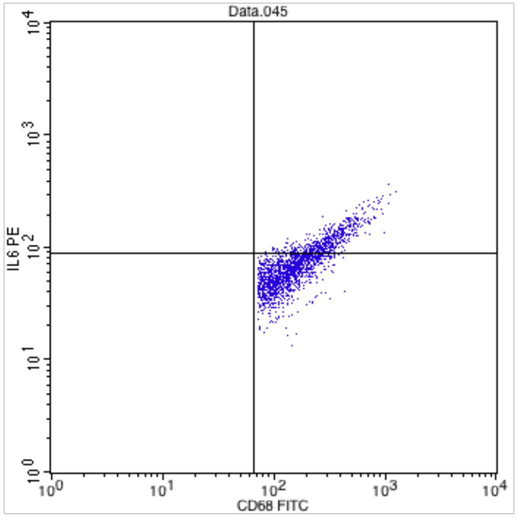

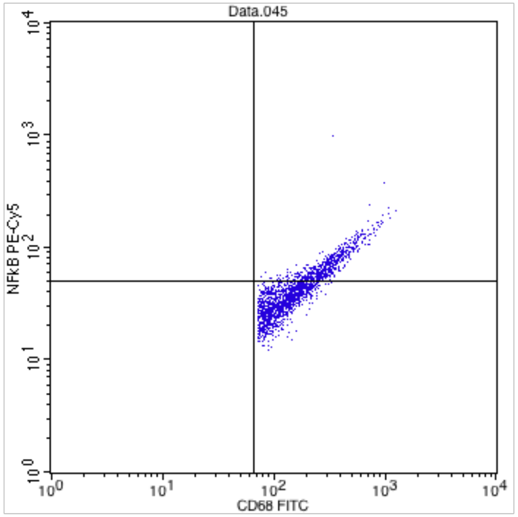

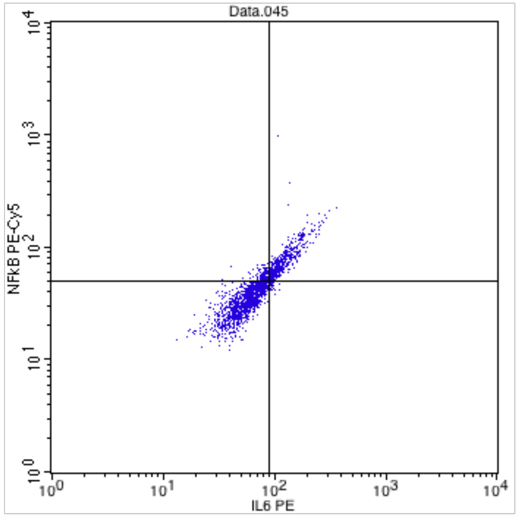

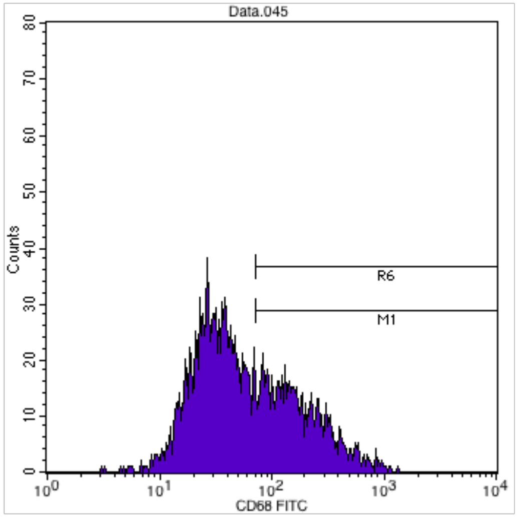

Quadrant Statistics

File: Data.045
Sample ID: 7.1
Acquisition Date: 16-Oct-24
Gate: G7
Gated Events: 1927
Total Events: 53824
X Parameter: CD68 FITC (Log)
Y Parameter: IL6 PE (Log)
Quad Location: 66, 90

Quad	Events	% Gated	% Total
UL	0	0.00	0.00
UR	492	25.53	0.91
LL	0	0.00	0.00
LR	1435	74.47	2.67

Quadrant Statistics

File: Data.045
Sample ID: 7.1
Acquisition Date: 16-Oct-24
Gate: G7
Gated Events: 1927
Total Events: 53824
X Parameter: CD68 FITC (Log)
Y Parameter: NFkB PE-Cy5 (Log)
Quad Location: 66, 50

Quad	Events	% Gated	% Total
UL	0	0.00	0.00
UR	620	32.17	1.15
LL	0	0.00	0.00
LR	1307	67.83	2.43

Quadrant Statistics

File: Data.045
Sample ID: 7.1
Acquisition Date: 16-Oct-24
Gate: G7
Gated Events: 1927
Total Events: 53824
X Parameter: IL6 PE (Log)
Y Parameter: NFkB PE-Cy5 (Log)
Quad Location: 90, 50

Quad	Events	% Gated	% Total
UL	161	8.35	0.30
UR	459	23.82	0.85
LL	1274	66.11	2.37
LR	33	1.71	0.06

Histogram Statistics

File: Data.045
Sample ID: 7.1
Acquisition Date: 16-Oct-24
Gate: G2
Gated Events: 5235
Total Events: 53824
X Parameter: CD68 FITC (Log)

Marker	Left, Right	Events	% Gated	% Total
All	1, 9910	5235	100.00	9.73
M1	72, 9910	1927	36.81	3.58

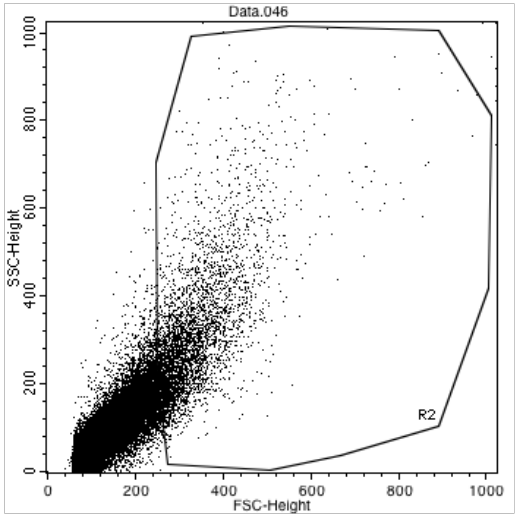

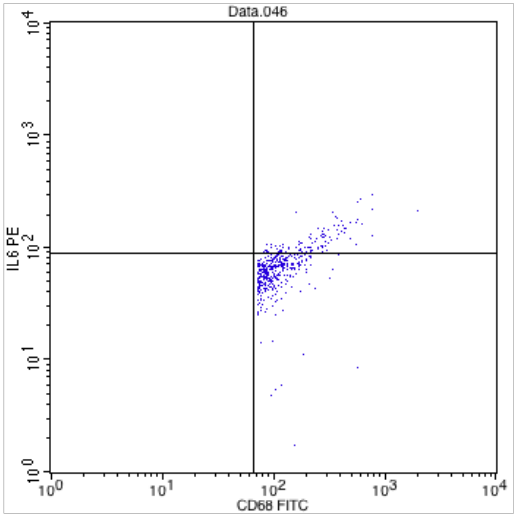

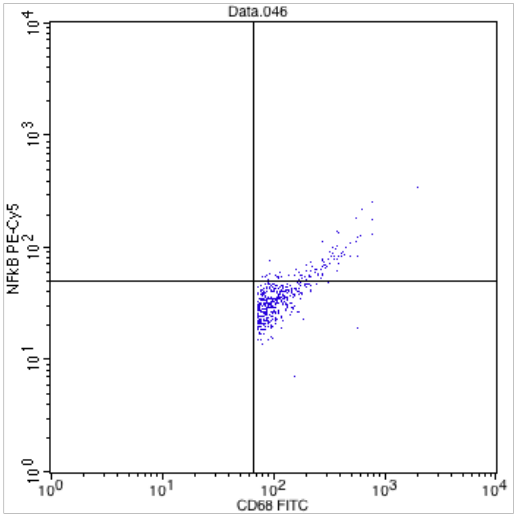

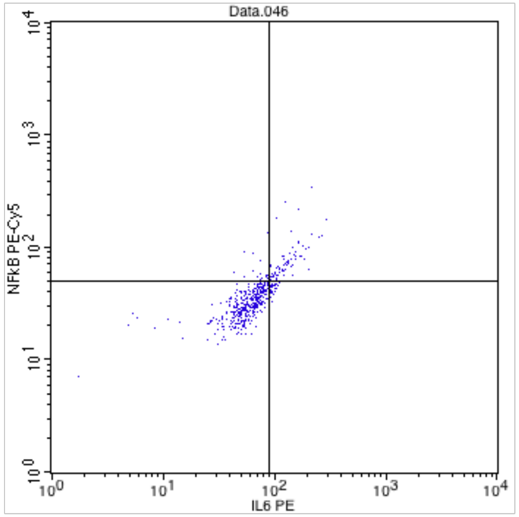

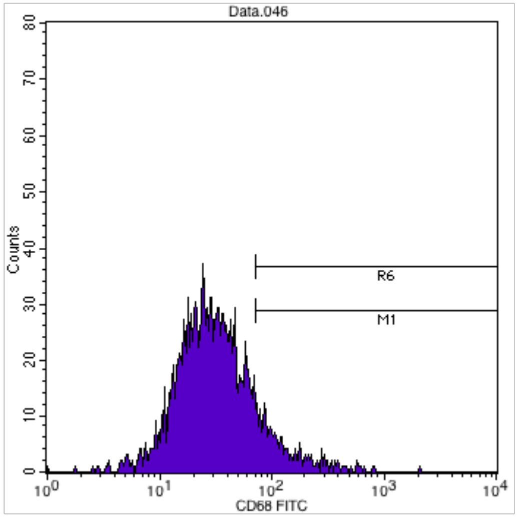

Quadrant Statistics

File: Data.046 Sample ID: 7.3
Acquisition Date: 16-Oct-24 Gate: G7
Gated Events: 494 Total Events: 66785
X Parameter: CD68 FITC (Log) Y Parameter: IL6 PE (Log)
Quad Location: 66, 90

Quad	Events	% Gated	% Total
UL	0	0.00	0.00
UR	100	20.24	0.15
LL	0	0.00	0.00
LR	394	79.76	0.59

Quadrant Statistics

File: Data.046 Sample ID: 7.3
Acquisition Date: 16-Oct-24 Gate: G7
Gated Events: 494 Total Events: 66785
X Parameter: CD68 FITC (Log) Y Parameter: NFkB PE-Cy5 (Log)
Quad Location: 66, 50

Quad	Events	% Gated	% Total
UL	0	0.00	0.00
UR	86	17.41	0.13
LL	0	0.00	0.00
LR	408	82.59	0.61

Quadrant Statistics

File: Data.046 Sample ID: 7.3
Acquisition Date: 16-Oct-24 Gate: G7
Gated Events: 494 Total Events: 66785
X Parameter: IL6 PE (Log) Y Parameter: NFkB PE-Cy5 (Log)
Quad Location: 90, 50

Quad	Events	% Gated	% Total
UL	15	3.04	0.02
UR	71	14.37	0.11
LL	379	76.72	0.57
LR	29	5.87	0.04

Histogram Statistics

File: Data.046 Sample ID: 7.3
Acquisition Date: 16-Oct-24 Gate: G2
Gated Events: 4595 Total Events: 66785
X Parameter: CD68 FITC (Log)

Marker	Left, Right	Events	% Gated	% Total
All	1, 9910	4595	100.00	6.88
M1	72, 9910	494	10.75	0.74

CD68-IL6-NFkb
G2=R2 (Populasi sel hidup)
G7=R2 and R6 (Populasi total sel CD68)
17-10-2024

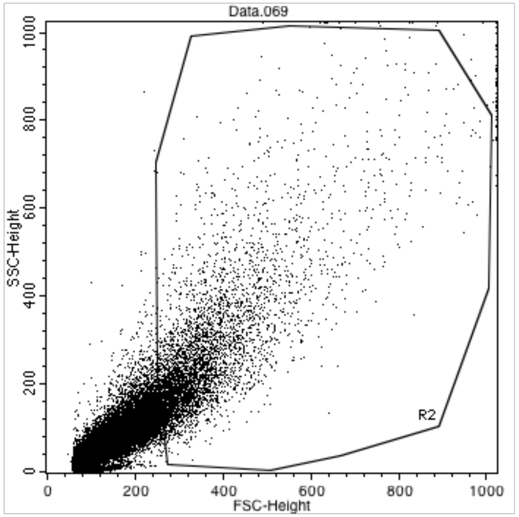

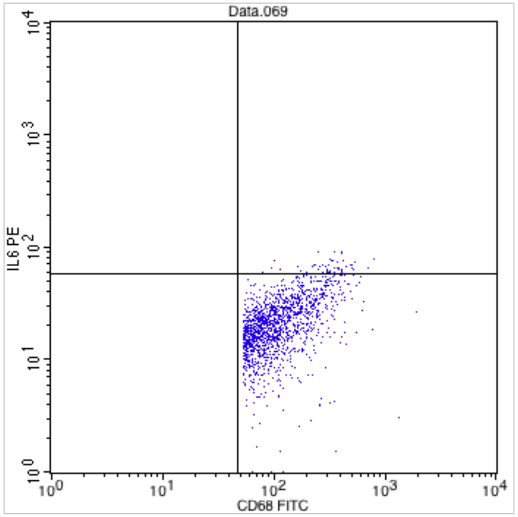

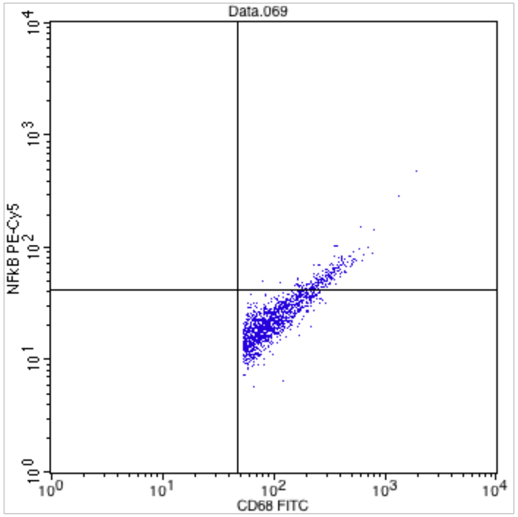

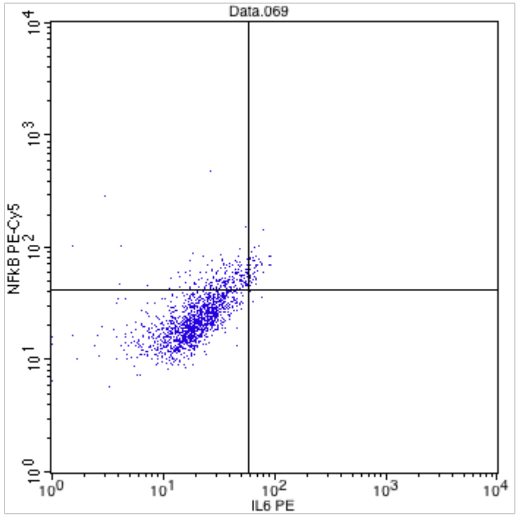

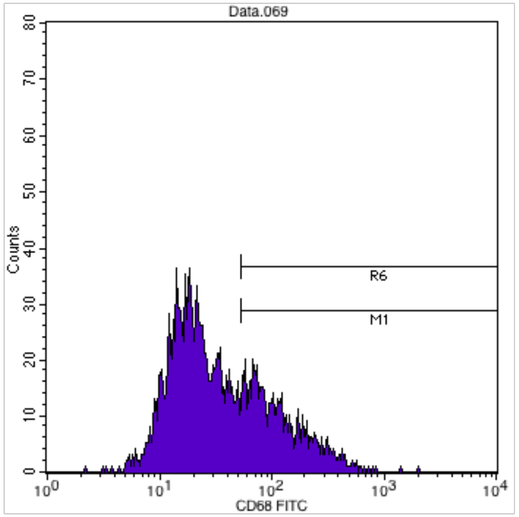

Quadrant Statistics

File: Data.069
Acquisition Date: 18-Oct-24
Gated Events: 1572
X Parameter: CD68 FITC (Log)
Quad Location: 47, 58
Sample ID: Norm.4
Gate: G7
Total Events: 55113
Y Parameter: IL6 PE (Log)

Quad	Events	% Gated	% Total
UL	0	0.00	0.00
UR	47	2.99	0.09
LL	0	0.00	0.00
LR	1525	97.01	2.77

Quadrant Statistics

File: Data.069
Acquisition Date: 18-Oct-24
Gated Events: 1572
X Parameter: CD68 FITC (Log)
Quad Location: 47, 42
Sample ID: Norm.4
Gate: G7
Total Events: 55113
Y Parameter: NFkB PE-Cy5 (Log)

Quad	Events	% Gated	% Total
UL	0	0.00	0.00
UR	225	14.31	0.41
LL	0	0.00	0.00
LR	1347	85.69	2.44

Quadrant Statistics

File: Data.069
Acquisition Date: 18-Oct-24
Gated Events: 1572
X Parameter: IL6 PE (Log)
Quad Location: 58, 42
Sample ID: Norm.4
Gate: G7
Total Events: 55113
Y Parameter: NFkB PE-Cy5 (Log)

Quad	Events	% Gated	% Total
UL	181	11.51	0.33
UR	44	2.80	0.08
LL	1344	85.50	2.44
LR	3	0.19	0.01

Histogram Statistics

File: Data.069
Acquisition Date: 18-Oct-24
Gated Events: 4999
X Parameter: CD68 FITC (Log)
Sample ID: Norm.4
Gate: G2
Total Events: 55113

Marker	Left, Right	Events	% Gated	% Total
All	1, 9910	4999	100.00	9.07
M1	53, 9910	1572	31.45	2.85

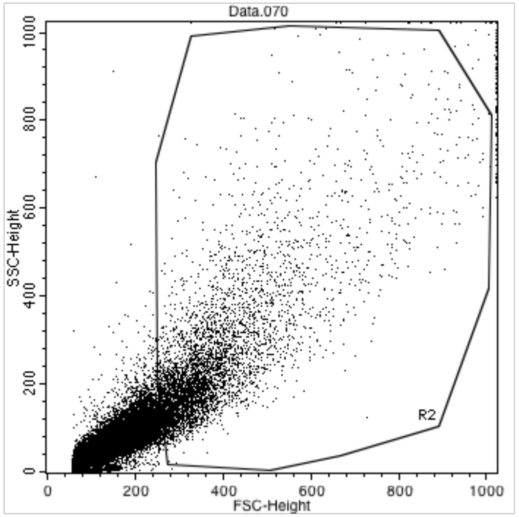

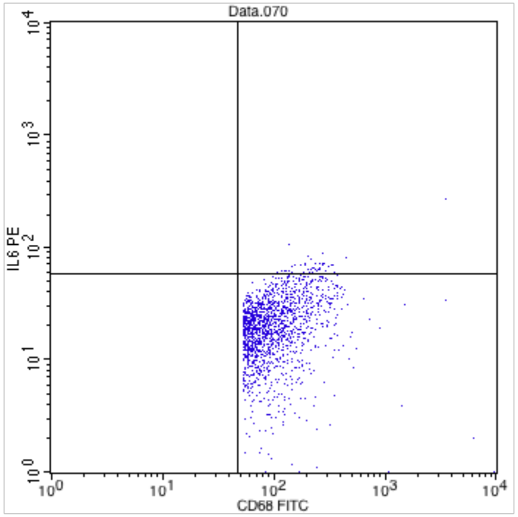

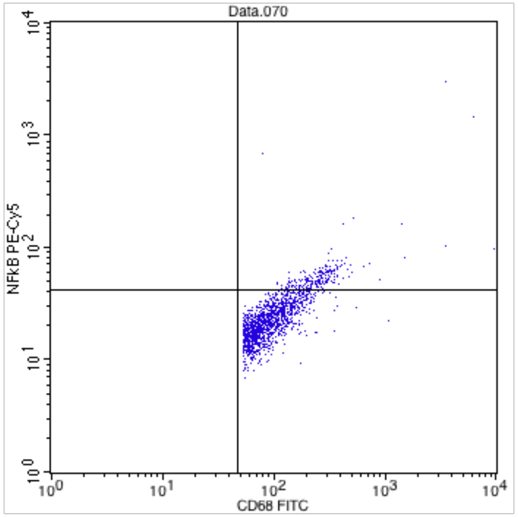

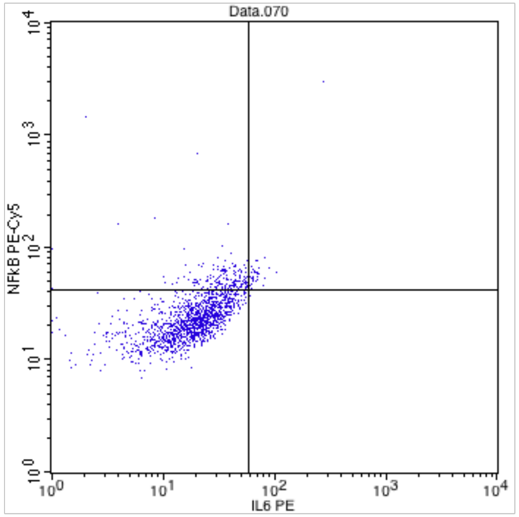

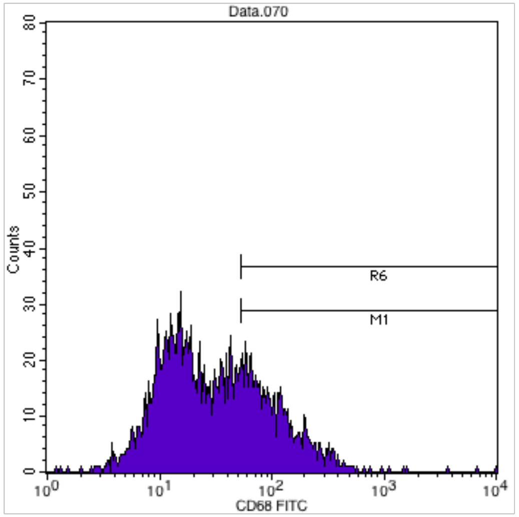

Quadrant Statistics

File: Data.070
Acquisition Date: 18-Oct-24
Gated Events: 1566
X Parameter: CD68 FITC (Log)
Quad Location: 47, 58
Sample ID: Norm.5
Gate: G7
Total Events: 51543
Y Parameter: IL6 PE (Log)

Quad	Events	% Gated	% Total
UL	0	0.00	0.00
UR	42	2.68	0.08
LL	0	0.00	0.00
LR	1524	97.32	2.96

Quadrant Statistics

File: Data.070
Acquisition Date: 18-Oct-24
Gated Events: 1566
X Parameter: CD68 FITC (Log)
Quad Location: 47, 42
Sample ID: Norm.5
Gate: G7
Total Events: 51543
Y Parameter: NFkB PE-Cy5 (Log)

Quad	Events	% Gated	% Total
UL	0	0.00	0.00
UR	214	13.67	0.42
LL	0	0.00	0.00
LR	1352	86.33	2.62

Quadrant Statistics

File: Data.070
Acquisition Date: 18-Oct-24
Gated Events: 1566
X Parameter: IL6 PE (Log)
Quad Location: 58, 42
Sample ID: Norm.5
Gate: G7
Total Events: 51543
Y Parameter: NFkB PE-Cy5 (Log)

Quad	Events	% Gated	% Total
UL	177	11.30	0.34
UR	37	2.36	0.07
LL	1350	86.21	2.62
LR	2	0.13	0.00

Histogram Statistics

File: Data.070
Acquisition Date: 18-Oct-24
Gated Events: 5143
X Parameter: CD68 FITC (Log)
Sample ID: Norm.5
Gate: G2
Total Events: 51543

Marker	Left, Right	Events	% Gated	% Total
All	1, 9910	5143	100.00	9.98
M1	53, 9910	1566	30.45	3.04

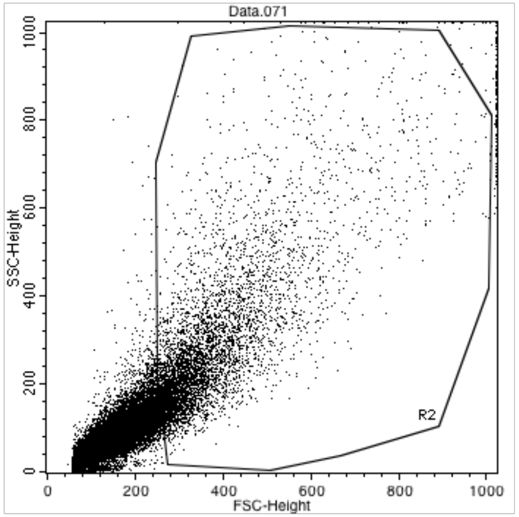

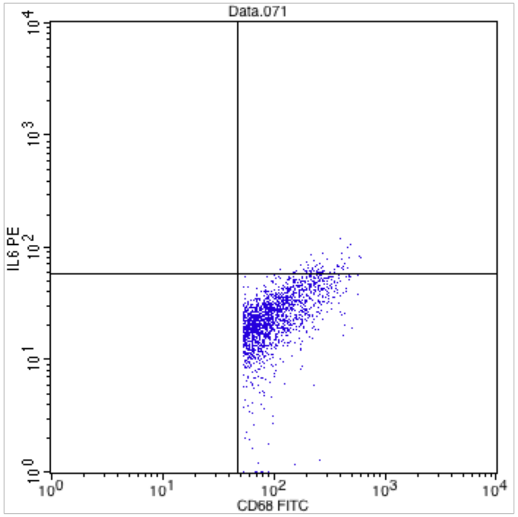

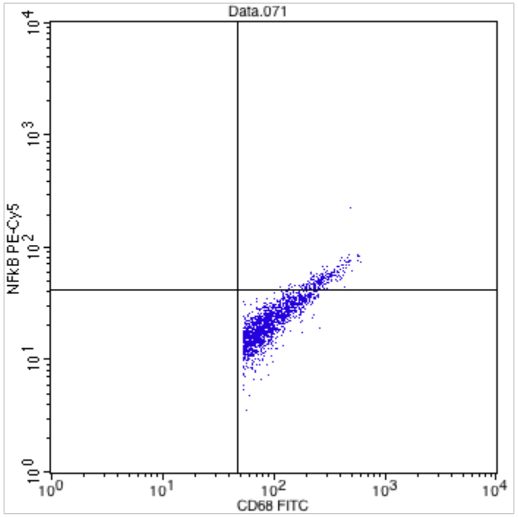

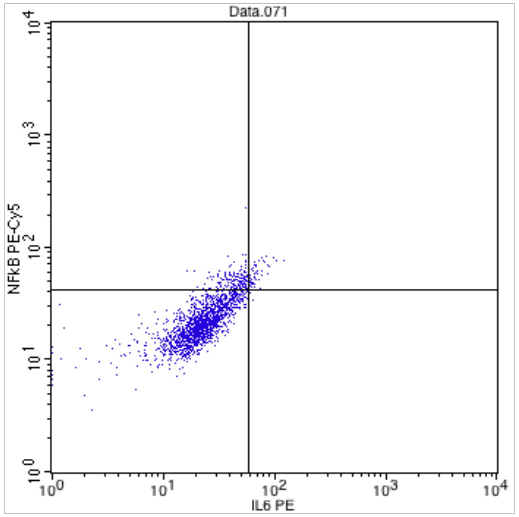

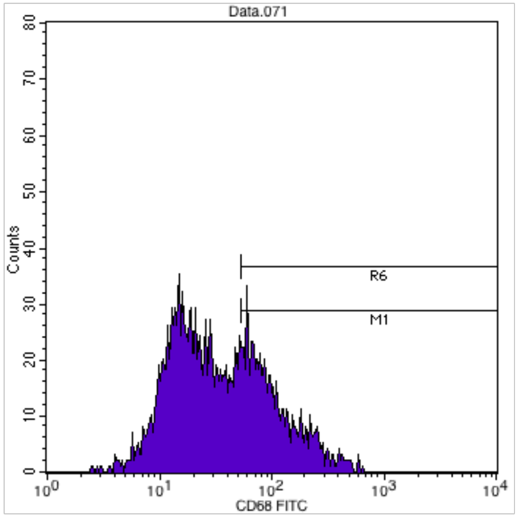

Quadrant Statistics

File: Data.071
Acquisition Date: 18-Oct-24
Gated Events: 1901
X Parameter: CD68 FITC (Log)
Quad Location: 47, 58
Sample ID: Norm.6
Gate: G7
Total Events: 48730
Y Parameter: IL6 PE (Log)

Quad	Events	% Gated	% Total
UL	0	0.00	0.00
UR	62	3.26	0.13
LL	0	0.00	0.00
LR	1839	96.74	3.77

Quadrant Statistics

File: Data.071
Acquisition Date: 18-Oct-24
Gated Events: 1901
X Parameter: CD68 FITC (Log)
Quad Location: 47, 42
Sample ID: Norm.6
Gate: G7
Total Events: 48730
Y Parameter: NFkB PE-Cy5 (Log)

Quad	Events	% Gated	% Total
UL	0	0.00	0.00
UR	222	11.68	0.46
LL	0	0.00	0.00
LR	1679	88.32	3.45

Quadrant Statistics

File: Data.071
Acquisition Date: 18-Oct-24
Gated Events: 1901
X Parameter: IL6 PE (Log)
Quad Location: 58, 42
Sample ID: Norm.6
Gate: G7
Total Events: 48730
Y Parameter: NFkB PE-Cy5 (Log)

Quad	Events	% Gated	% Total
UL	171	9.00	0.35
UR	51	2.68	0.10
LL	1673	88.01	3.43
LR	6	0.32	0.01

Histogram Statistics

File: Data.071
Acquisition Date: 18-Oct-24
Gated Events: 5682
X Parameter: CD68 FITC (Log)
Sample ID: Norm.6
Gate: G2
Total Events: 48730

Marker	Left, Right	Events	% Gated	% Total
All	1, 9910	5682	100.00	11.66
M1	53, 9910	1901	33.46	3.90

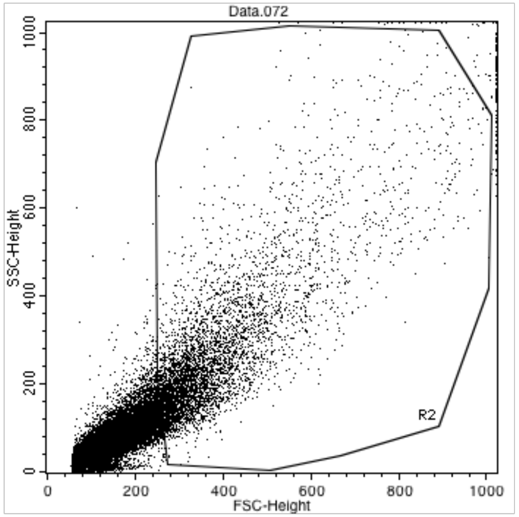

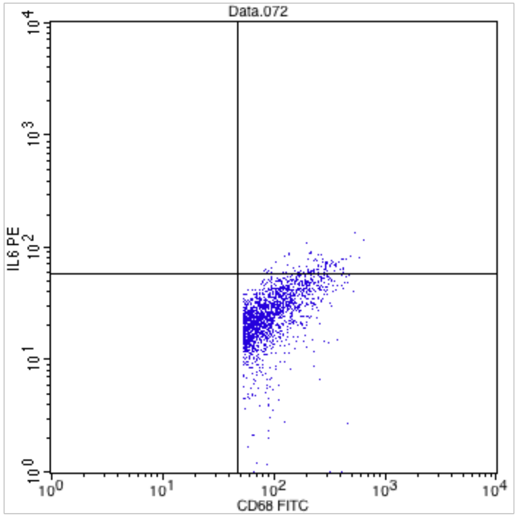

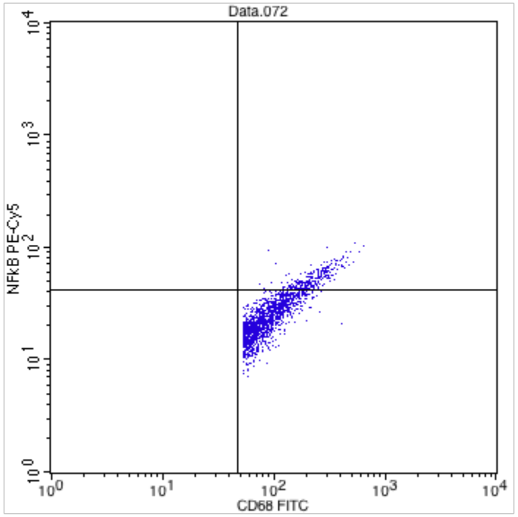

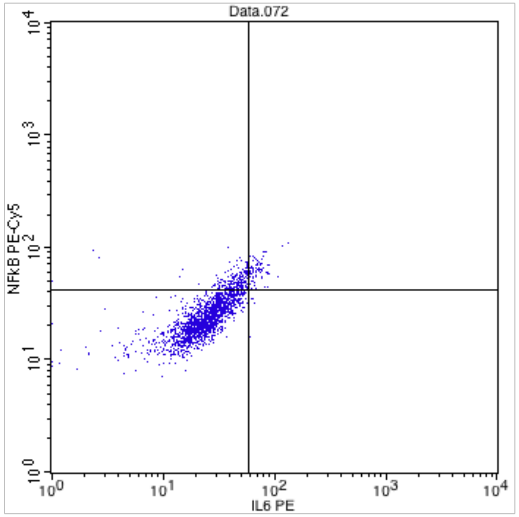

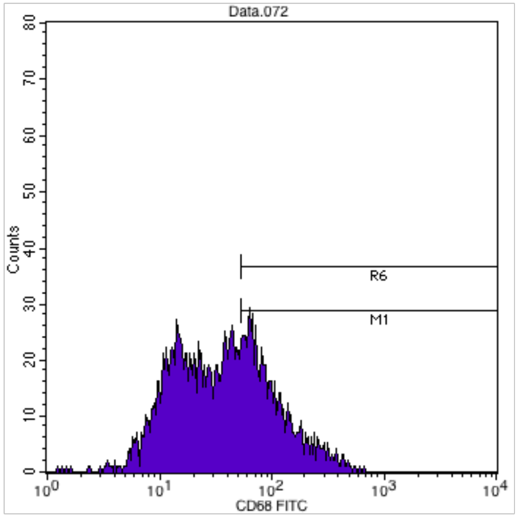

Quadrant Statistics

File: Data.072
Acquisition Date: 18-Oct-24
Gated Events: 1934
X Parameter: CD68 FITC (Log)
Quad Location: 47, 58
Sample ID: +.4
Gate: G7
Total Events: 51630
Y Parameter: IL6 PE (Log)

Quad	Events	% Gated	% Total
UL	0	0.00	0.00
UR	91	4.71	0.18
LL	0	0.00	0.00
LR	1843	95.29	3.57

Quadrant Statistics

File: Data.072
Acquisition Date: 18-Oct-24
Gated Events: 1934
X Parameter: CD68 FITC (Log)
Quad Location: 47, 42
Sample ID: +.4
Gate: G7
Total Events: 51630
Y Parameter: NFkB PE-Cy5 (Log)

Quad	Events	% Gated	% Total
UL	0	0.00	0.00
UR	281	14.53	0.54
LL	0	0.00	0.00
LR	1653	85.47	3.20

Quadrant Statistics

File: Data.072
Acquisition Date: 18-Oct-24
Gated Events: 1934
X Parameter: IL6 PE (Log)
Quad Location: 58, 42
Sample ID: +.4
Gate: G7
Total Events: 51630
Y Parameter: NFkB PE-Cy5 (Log)

Quad	Events	% Gated	% Total
UL	199	10.29	0.39
UR	82	4.24	0.16
LL	1648	85.21	3.19
LR	5	0.26	0.01

Histogram Statistics

File: Data.072
Acquisition Date: 18-Oct-24
Gated Events: 5341
X Parameter: CD68 FITC (Log)
Sample ID: +.4
Gate: G2
Total Events: 51630

Marker	Left, Right	Events	% Gated	% Total
All	1, 9910	5341	100.00	10.34
M1	53, 9910	1934	36.21	3.75

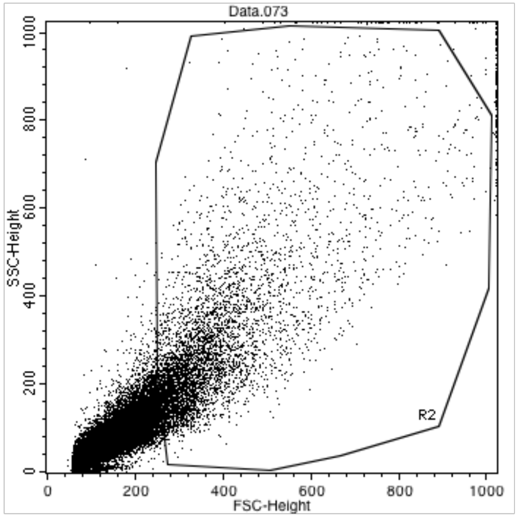

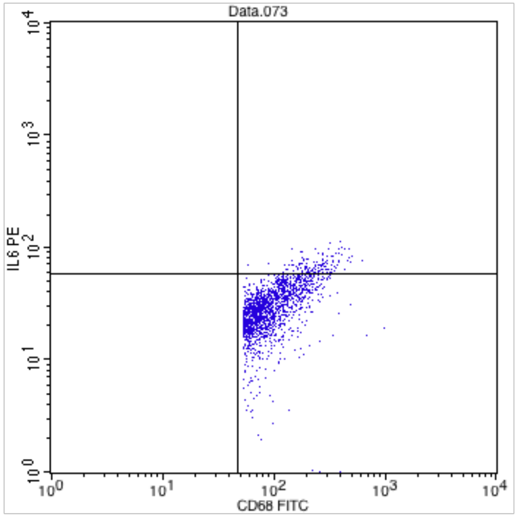

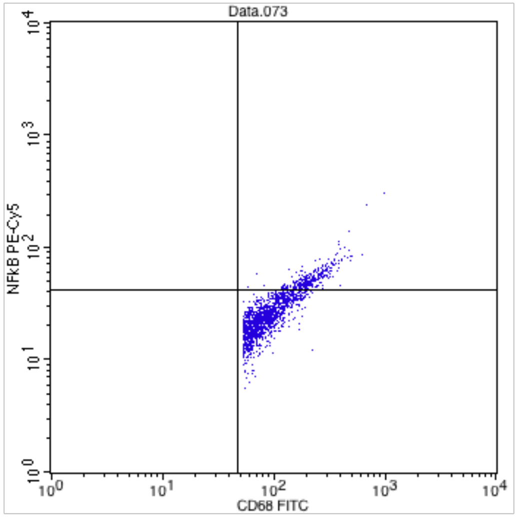

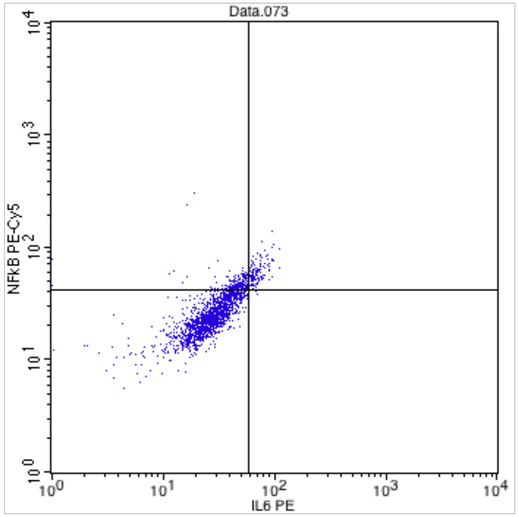

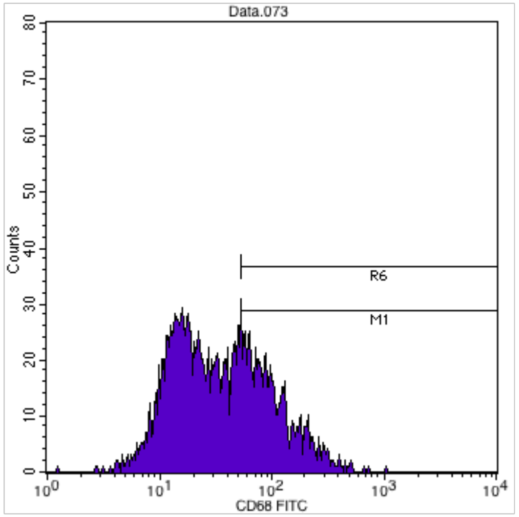

Quadrant Statistics

File: Data.073 Sample ID: +.5
Acquisition Date: 18-Oct-24 Gate: G7
Gated Events: 1813 Total Events: 54625
X Parameter: CD68 FITC (Log) Y Parameter: IL6 PE (Log)
Quad Location: 47, 58

Quad	Events	% Gated	% Total
UL	0	0.00	0.00
UR	133	7.34	0.24
LL	0	0.00	0.00
LR	1680	92.66	3.08

Quadrant Statistics

File: Data.073 Sample ID: +.5
Acquisition Date: 18-Oct-24 Gate: G7
Gated Events: 1813 Total Events: 54625
X Parameter: CD68 FITC (Log) Y Parameter: NFkB PE-Cy5 (Log)
Quad Location: 47, 42

Quad	Events	% Gated	% Total
UL	0	0.00	0.00
UR	300	16.55	0.55
LL	0	0.00	0.00
LR	1513	83.45	2.77

Quadrant Statistics

File: Data.073 Sample ID: +.5
Acquisition Date: 18-Oct-24 Gate: G7
Gated Events: 1813 Total Events: 54625
X Parameter: IL6 PE (Log) Y Parameter: NFkB PE-Cy5 (Log)
Quad Location: 58, 42

Quad	Events	% Gated	% Total
UL	176	9.71	0.32
UR	124	6.84	0.23
LL	1509	83.23	2.76
LR	4	0.22	0.01

Histogram Statistics

File: Data.073 Sample ID: +.5
Acquisition Date: 18-Oct-24 Gate: G2
Gated Events: 5411 Total Events: 54625
X Parameter: CD68 FITC (Log)

Marker	Left, Right	Events	% Gated	% Total
All	1, 9910	5411	100.00	9.91
M1	53, 9910	1813	33.51	3.32

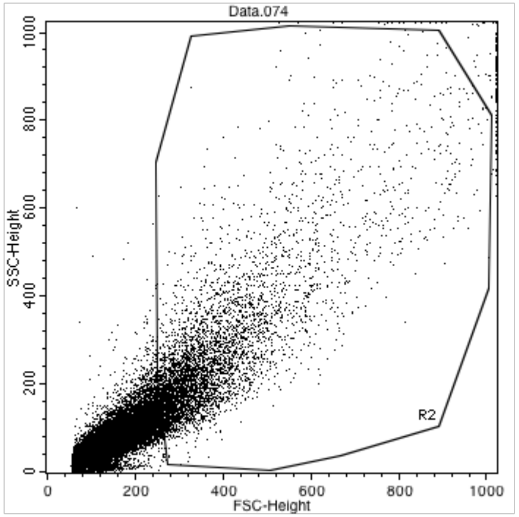

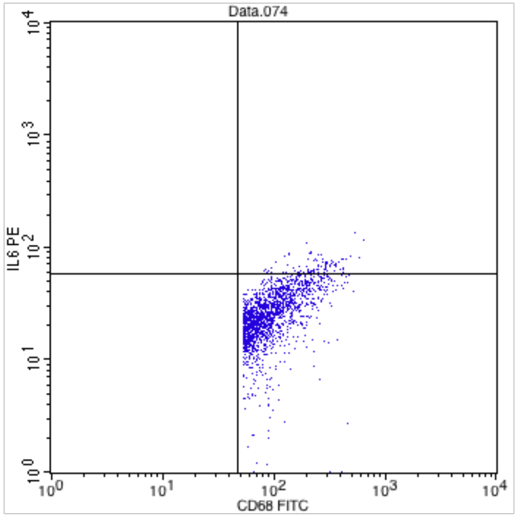

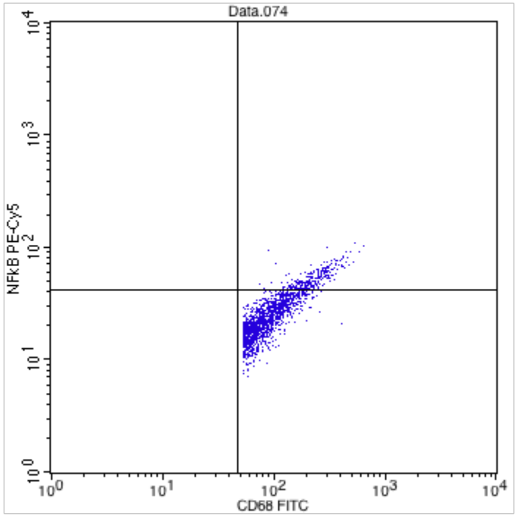

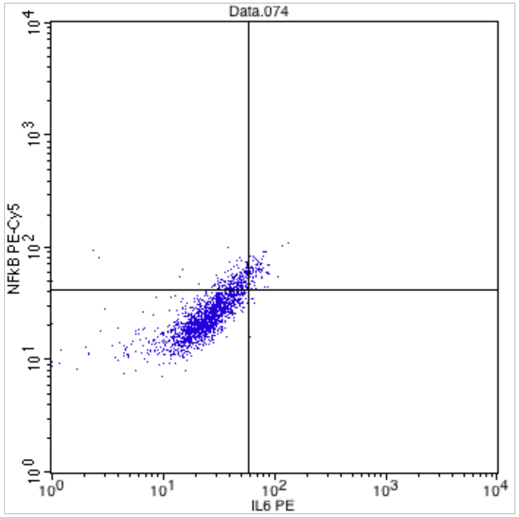

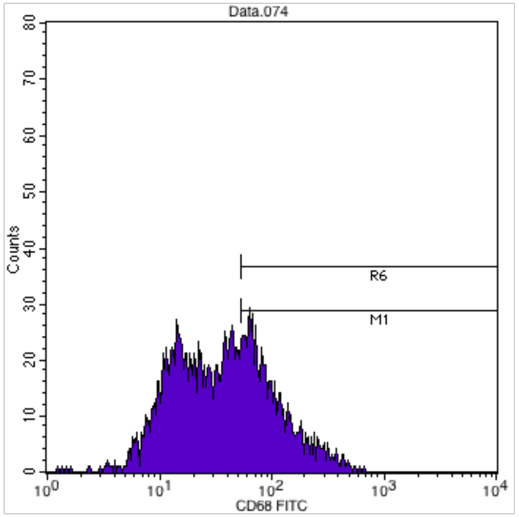

Quadrant Statistics

File: Data.074
Acquisition Date: 18-Oct-24
Gated Events: 1934
X Parameter: CD68 FITC (Log)
Quad Location: 47, 58
Sample ID: +.6
Gate: G7
Total Events: 51630
Y Parameter: IL6 PE (Log)

Quad	Events	% Gated	% Total
UL	0	0.00	0.00
UR	91	4.71	0.18
LL	0	0.00	0.00
LR	1843	95.29	3.57

Quadrant Statistics

File: Data.074
Acquisition Date: 18-Oct-24
Gated Events: 1934
X Parameter: CD68 FITC (Log)
Quad Location: 47, 42
Sample ID: +.6
Gate: G7
Total Events: 51630
Y Parameter: NFkB PE-Cy5 (Log)

Quad	Events	% Gated	% Total
UL	0	0.00	0.00
UR	281	14.53	0.54
LL	0	0.00	0.00
LR	1653	85.47	3.20

Quadrant Statistics

File: Data.074
Acquisition Date: 18-Oct-24
Gated Events: 1934
X Parameter: IL6 PE (Log)
Quad Location: 58, 42
Sample ID: +.6
Gate: G7
Total Events: 51630
Y Parameter: NFkB PE-Cy5 (Log)

Quad	Events	% Gated	% Total
UL	199	10.29	0.39
UR	82	4.24	0.16
LL	1648	85.21	3.19
LR	5	0.26	0.01

Histogram Statistics

File: Data.074
Acquisition Date: 18-Oct-24
Gated Events: 5341
X Parameter: CD68 FITC (Log)
Sample ID: +.6
Gate: G2
Total Events: 51630

Marker	Left, Right	Events	% Gated	% Total
All	1, 9910	5341	100.00	10.34
M1	53, 9910	1934	36.21	3.75

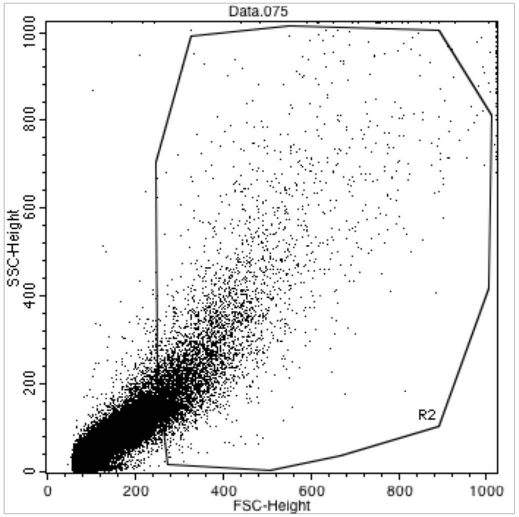

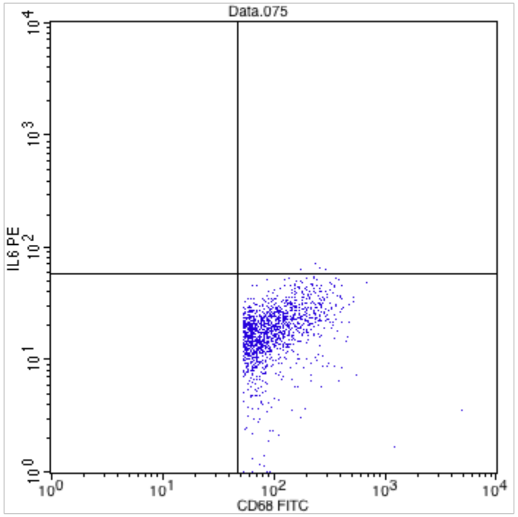

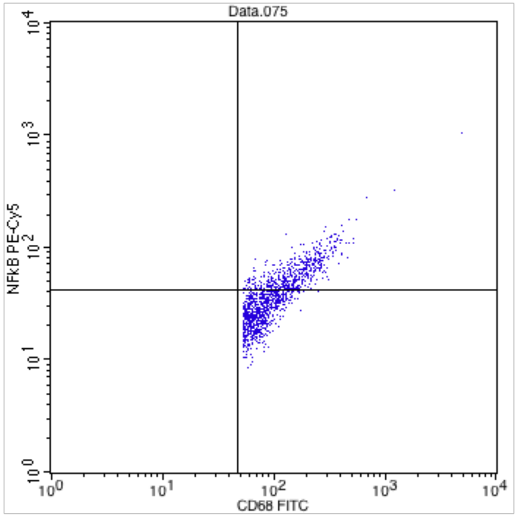

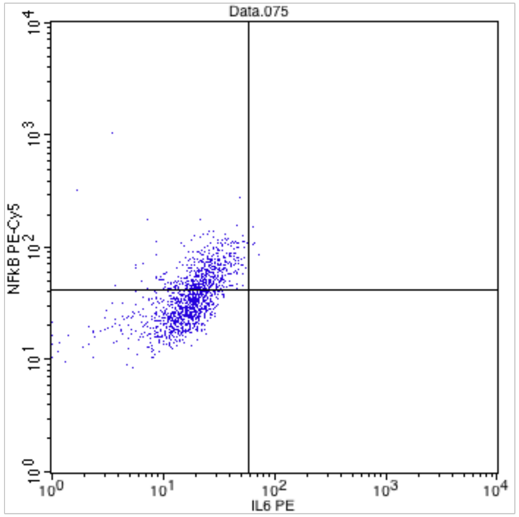

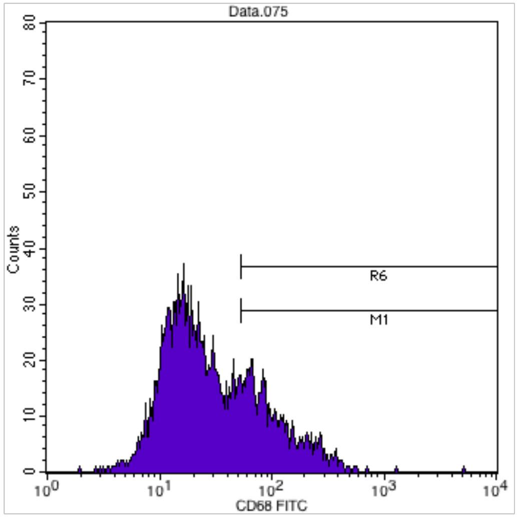

Quadrant Statistics

File: Data.075 Sample ID: -.6
Acquisition Date: 18-Oct-24 Gate: G7
Gated Events: 1363 Total Events: 58769
X Parameter: CD68 FITC (Log) Y Parameter: IL6 PE (Log)
Quad Location: 47, 58

Quad	Events	% Gated	% Total
UL	0	0.00	0.00
UR	6	0.44	0.01
LL	0	0.00	0.00
LR	1357	99.56	2.31

Quadrant Statistics

File: Data.075 Sample ID: -.6
Acquisition Date: 18-Oct-24 Gate: G7
Gated Events: 1363 Total Events: 58769
X Parameter: CD68 FITC (Log) Y Parameter: NFkB PE-Cy5 (Log)
Quad Location: 47, 42

Quad	Events	% Gated	% Total
UL	0	0.00	0.00
UR	514	37.71	0.87
LL	0	0.00	0.00
LR	849	62.29	1.44

Quadrant Statistics

File: Data.075 Sample ID: -.6
Acquisition Date: 18-Oct-24 Gate: G7
Gated Events: 1363 Total Events: 58769
X Parameter: IL6 PE (Log) Y Parameter: NFkB PE-Cy5 (Log)
Quad Location: 58, 42

Quad	Events	% Gated	% Total
UL	510	37.42	0.87
UR	4	0.29	0.01
LL	849	62.29	1.44
LR	0	0.00	0.00

Histogram Statistics

File: Data.075 Sample ID: -.6
Acquisition Date: 18-Oct-24 Gate: G2
Gated Events: 5182 Total Events: 58769
X Parameter: CD68 FITC (Log)

Marker	Left, Right	Events	% Gated	% Total
All	1, 9910	5182	100.00	8.82
M1	53, 9910	1363	26.30	2.32

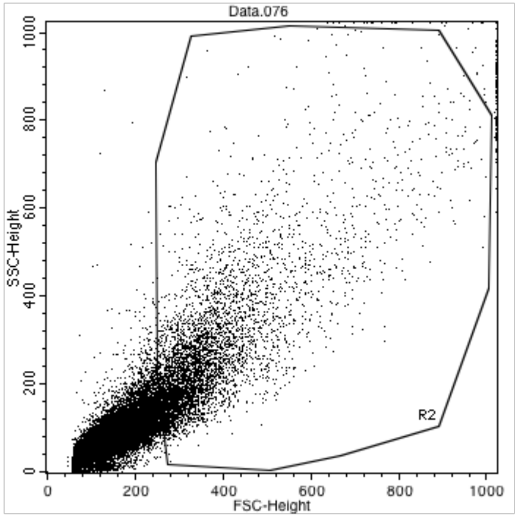

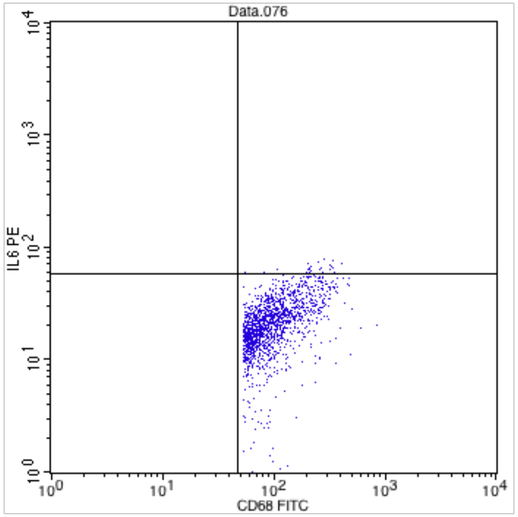

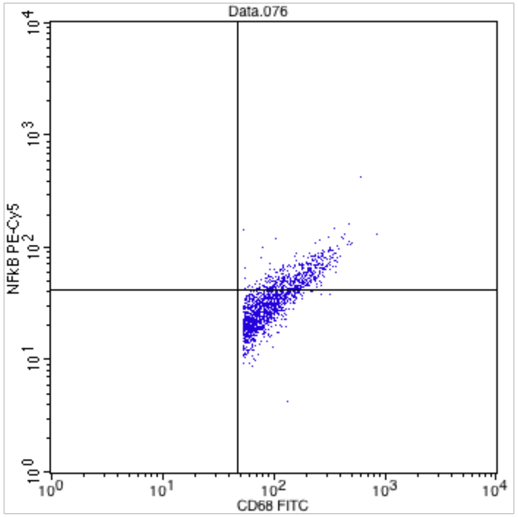

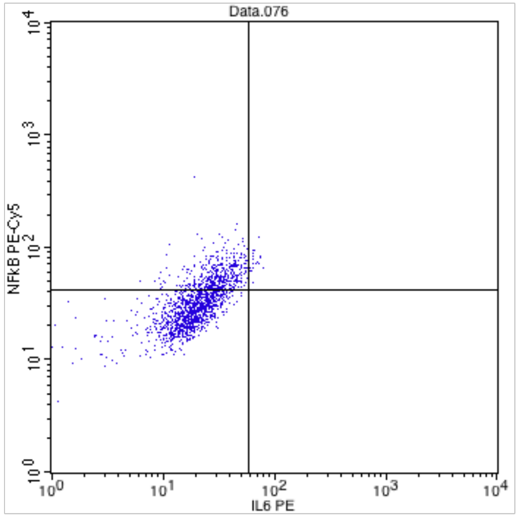

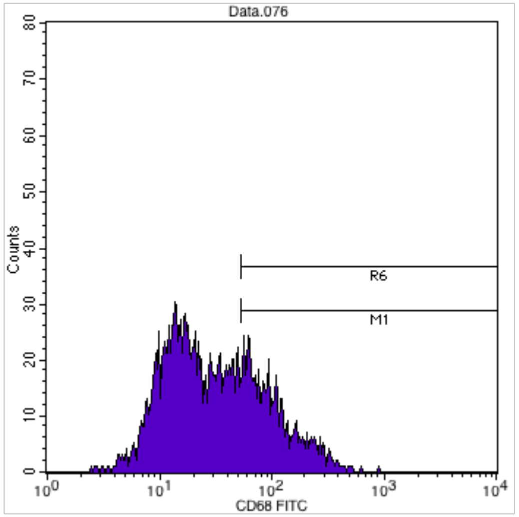

Quadrant Statistics

File: Data.076
Acquisition Date: 18-Oct-24
Gated Events: 1628
X Parameter: CD68 FITC (Log)
Quad Location: 47, 58
Sample ID: 1.4
Gate: G7
Total Events: 52667
Y Parameter: IL6 PE (Log)

Quad	Events	% Gated	% Total
UL	0	0.00	0.00
UR	23	1.41	0.04
LL	0	0.00	0.00
LR	1605	98.59	3.05

Quadrant Statistics

File: Data.076
Acquisition Date: 18-Oct-24
Gated Events: 1628
X Parameter: CD68 FITC (Log)
Quad Location: 47, 42
Sample ID: 1.4
Gate: G7
Total Events: 52667
Y Parameter: NFkB PE-Cy5 (Log)

Quad	Events	% Gated	% Total
UL	0	0.00	0.00
UR	479	29.42	0.91
LL	0	0.00	0.00
LR	1149	70.58	2.18

Quadrant Statistics

File: Data.076
Acquisition Date: 18-Oct-24
Gated Events: 1628
X Parameter: IL6 PE (Log)
Quad Location: 58, 42
Sample ID: 1.4
Gate: G7
Total Events: 52667
Y Parameter: NFkB PE-Cy5 (Log)

Quad	Events	% Gated	% Total
UL	456	28.01	0.87
UR	23	1.41	0.04
LL	1149	70.58	2.18
LR	0	0.00	0.00

Histogram Statistics

File: Data.076
Acquisition Date: 18-Oct-24
Gated Events: 5263
X Parameter: CD68 FITC (Log)
Sample ID: 1.4
Gate: G2
Total Events: 52667

Marker	Left, Right	Events	% Gated	% Total
All	1, 9910	5263	100.00	9.99
M1	53, 9910	1628	30.93	3.09

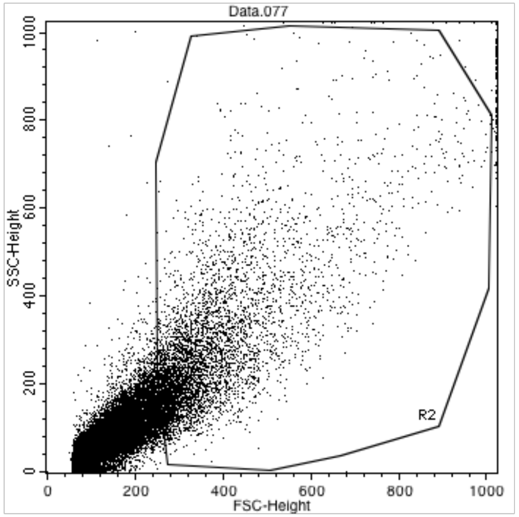

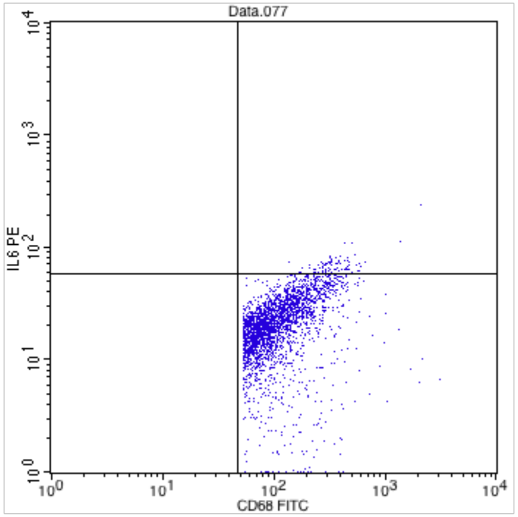

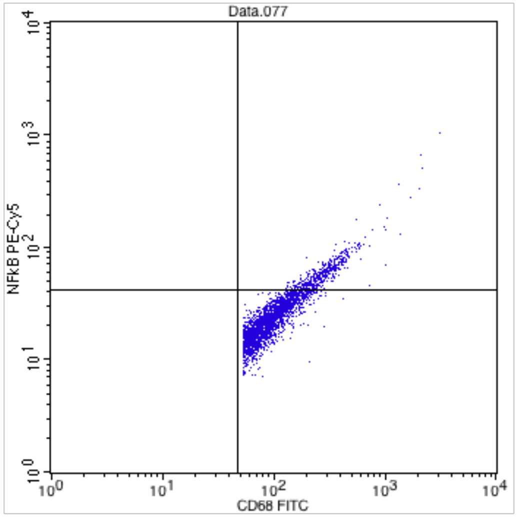

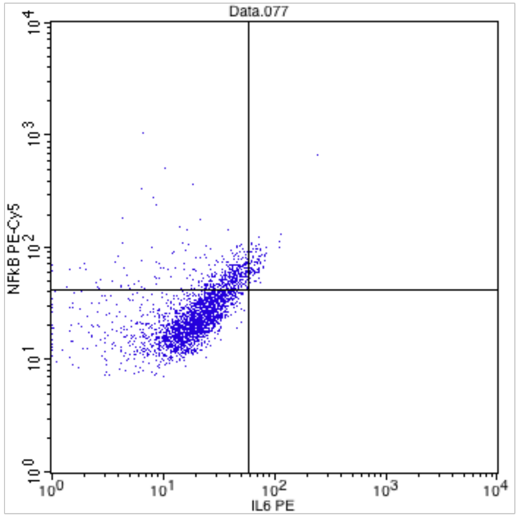

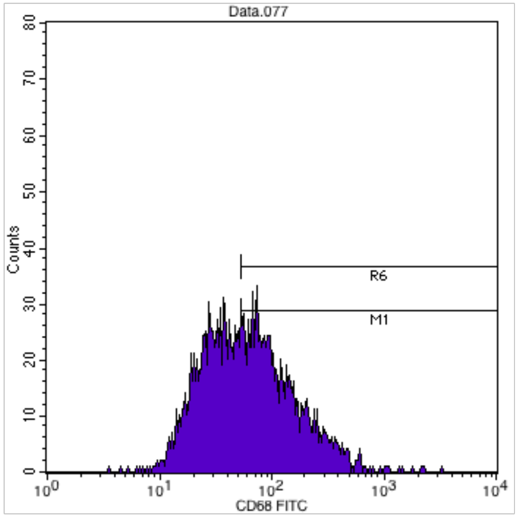

Quadrant Statistics

File: Data.077
Acquisition Date: 18-Oct-24
Gated Events: 2805
X Parameter: CD68 FITC (Log)
Quad Location: 47, 58
Sample ID: 1.5
Gate: G7
Total Events: 52408
Y Parameter: IL6 PE (Log)

Quad	Events	% Gated	% Total
UL	0	0.00	0.00
UR	83	2.96	0.16
LL	0	0.00	0.00
LR	2722	97.04	5.19

Quadrant Statistics

File: Data.077
Acquisition Date: 18-Oct-24
Gated Events: 2805
X Parameter: CD68 FITC (Log)
Quad Location: 47, 42
Sample ID: 1.5
Gate: G7
Total Events: 52408
Y Parameter: NFkB PE-Cy5 (Log)

Quad	Events	% Gated	% Total
UL	0	0.00	0.00
UR	513	18.29	0.98
LL	0	0.00	0.00
LR	2292	81.71	4.37

Quadrant Statistics

File: Data.077
Acquisition Date: 18-Oct-24
Gated Events: 2805
X Parameter: IL6 PE (Log)
Quad Location: 58, 42
Sample ID: 1.5
Gate: G7
Total Events: 52408
Y Parameter: NFkB PE-Cy5 (Log)

Quad	Events	% Gated	% Total
UL	432	15.40	0.82
UR	81	2.89	0.15
LL	2292	81.71	4.37
LR	0	0.00	0.00

Histogram Statistics

File: Data.077
Acquisition Date: 18-Oct-24
Gated Events: 5370
X Parameter: CD68 FITC (Log)
Sample ID: 1.5
Gate: G2
Total Events: 52408

Marker	Left, Right	Events	% Gated	% Total
All	1, 9910	5370	100.00	10.25
M1	53, 9910	2805	52.23	5.35

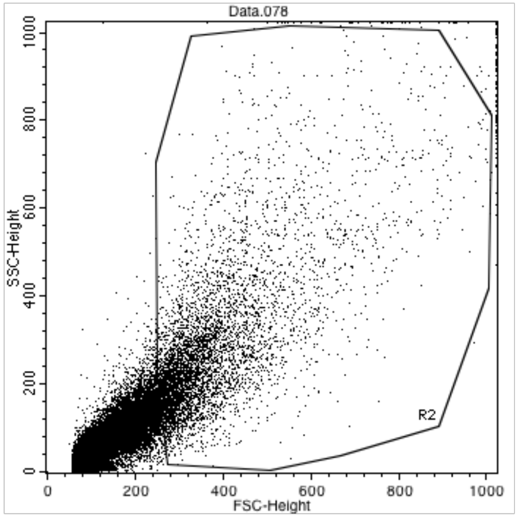

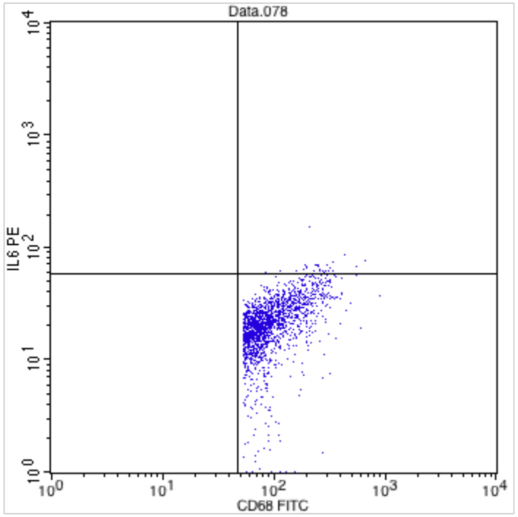

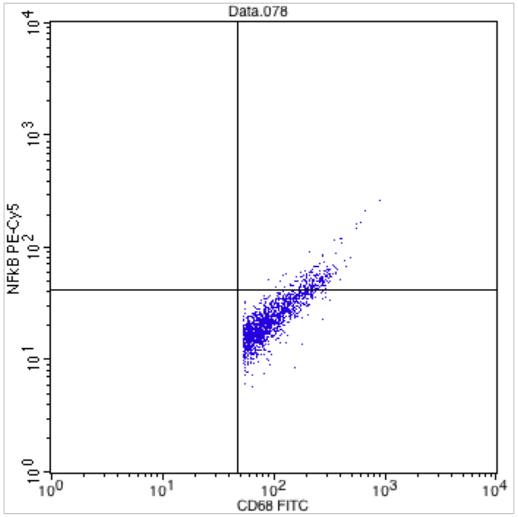

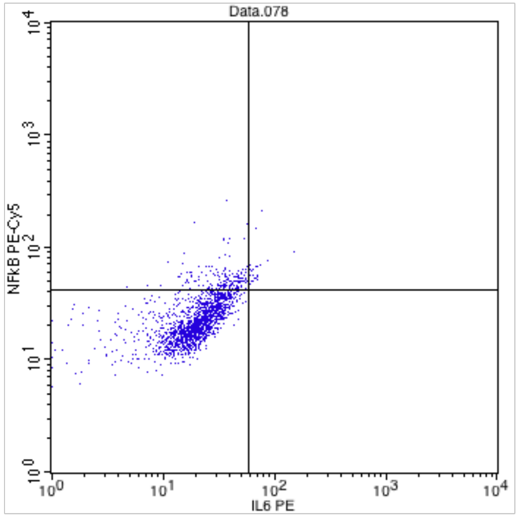

Quadrant Statistics

File: Data.078
Acquisition Date: 18-Oct-24
Gated Events: 1797
X Parameter: CD68 FITC (Log)
Quad Location: 47, 58
Sample ID: 1.7
Gate: G7
Total Events: 48987
Y Parameter: IL6 PE (Log)

Quad	Events	% Gated	% Total
UL	0	0.00	0.00
UR	29	1.61	0.06
LL	0	0.00	0.00
LR	1768	98.39	3.61

Quadrant Statistics

File: Data.078
Acquisition Date: 18-Oct-24
Gated Events: 1797
X Parameter: CD68 FITC (Log)
Quad Location: 47, 42
Sample ID: 1.7
Gate: G7
Total Events: 48987
Y Parameter: NFkB PE-Cy5 (Log)

Quad	Events	% Gated	% Total
UL	0	0.00	0.00
UR	196	10.91	0.40
LL	0	0.00	0.00
LR	1601	89.09	3.27

Quadrant Statistics

File: Data.078
Acquisition Date: 18-Oct-24
Gated Events: 1797
X Parameter: IL6 PE (Log)
Quad Location: 58, 42
Sample ID: 1.7
Gate: G7
Total Events: 48987
Y Parameter: NFkB PE-Cy5 (Log)

Quad	Events	% Gated	% Total
UL	171	9.52	0.35
UR	25	1.39	0.05
LL	1600	89.04	3.27
LR	1	0.06	0.00

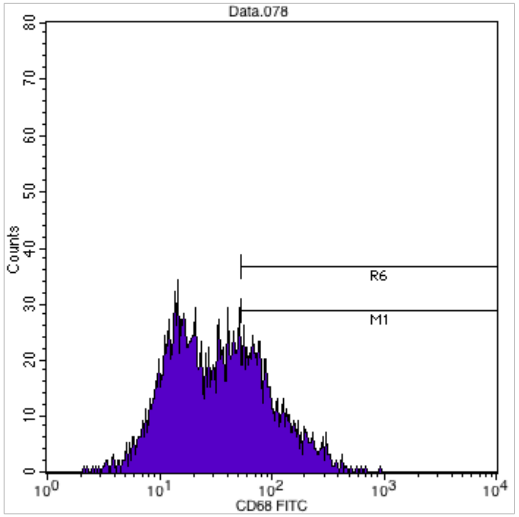

Histogram Statistics

File: Data.078
Acquisition Date: 18-Oct-24
Gated Events: 5613
X Parameter: CD68 FITC (Log)
Sample ID: 1.7
Gate: G2
Total Events: 48987

Marker	Left, Right	Events	% Gated	% Total
All	1, 9910	5613	100.00	11.46
M1	53, 9910	1797	32.01	3.67

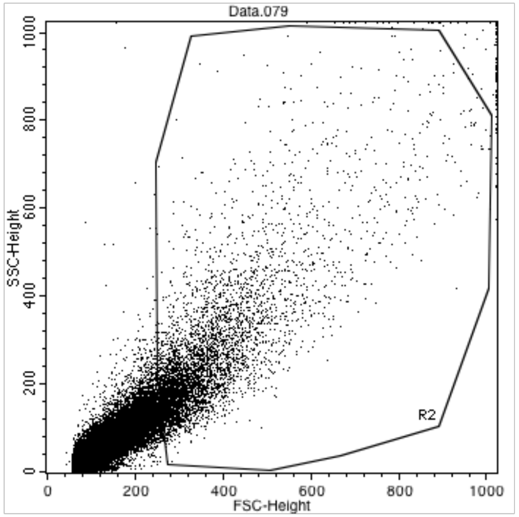

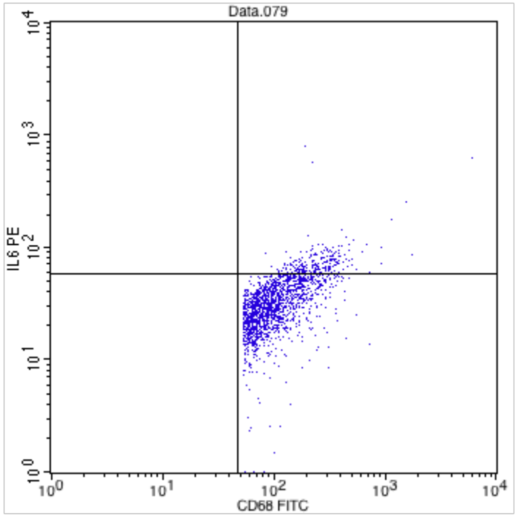

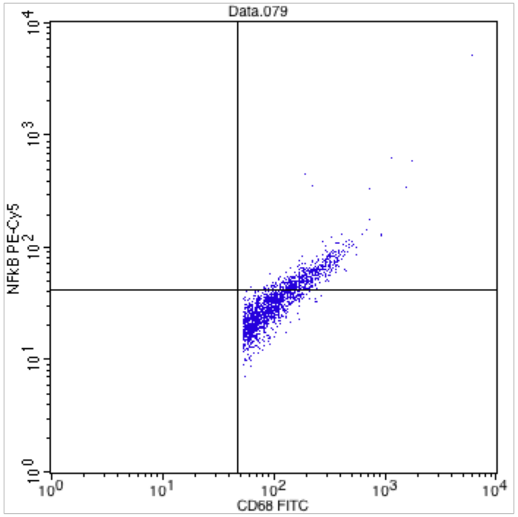

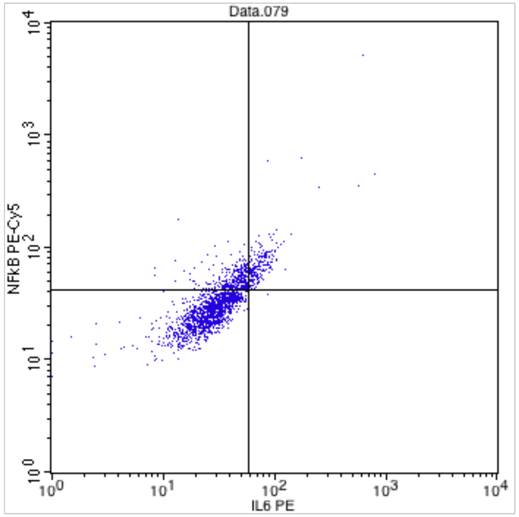

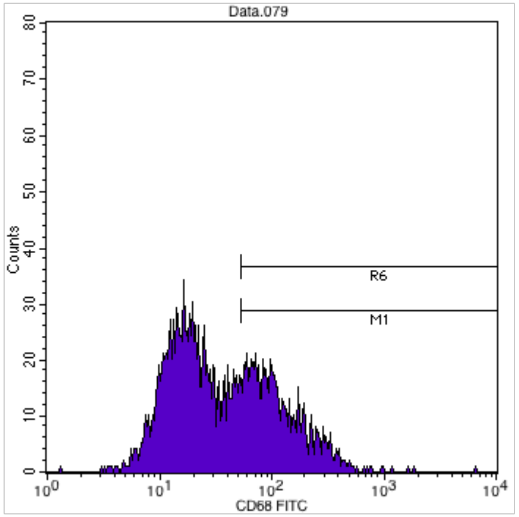

Quadrant Statistics

File: Data.079 | Sample ID: 2.4
Acquisition Date: 18-Oct-24 | Gate: G7
Gated Events: 1865 | Total Events: 58212
X Parameter: CD68 FITC (Log) | Y Parameter: IL6 PE (Log)
Quad Location: 47, 58

Quad	Events	% Gated	% Total
UL	0	0.00	0.00
UR	220	11.80	0.38
LL	0	0.00	0.00
LR	1645	88.20	2.83

Quadrant Statistics

File: Data.079 | Sample ID: 2.4
Acquisition Date: 18-Oct-24 | Gate: G7
Gated Events: 1865 | Total Events: 58212
X Parameter: CD68 FITC (Log) | Y Parameter: NFkB PE-Cy5 (Log)
Quad Location: 47, 42

Quad	Events	% Gated	% Total
UL	0	0.00	0.00
UR	544	29.17	0.93
LL	0	0.00	0.00
LR	1321	70.83	2.27

Quadrant Statistics

File: Data.079 | Sample ID: 2.4
Acquisition Date: 18-Oct-24 | Gate: G7
Gated Events: 1865 | Total Events: 58212
X Parameter: IL6 PE (Log) | Y Parameter: NFkB PE-Cy5 (Log)
Quad Location: 58, 42

Quad	Events	% Gated	% Total
UL	334	17.91	0.57
UR	210	11.26	0.36
LL	1320	70.78	2.27
LR	1	0.05	0.00

Histogram Statistics

File: Data.079 | Sample ID: 2.4
Acquisition Date: 18-Oct-24 | Gate: G2
Gated Events: 5123 | Total Events: 58212
X Parameter: CD68 FITC (Log)

Marker	Left, Right	Events	% Gated	% Total
All	1, 9910	5123	100.00	8.80
M1	53, 9910	1865	36.40	3.20

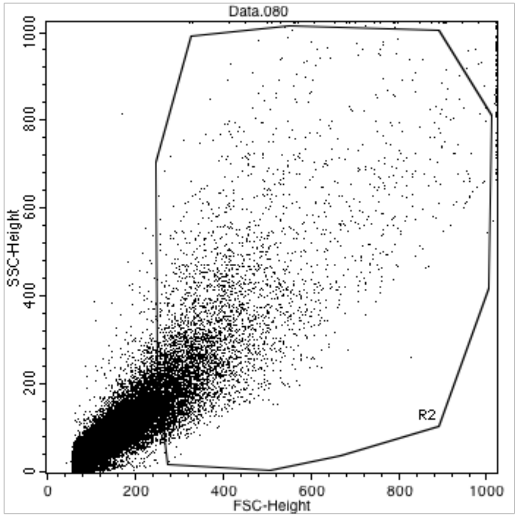

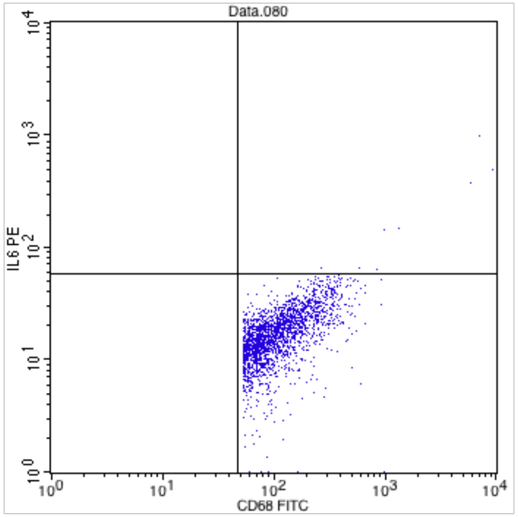

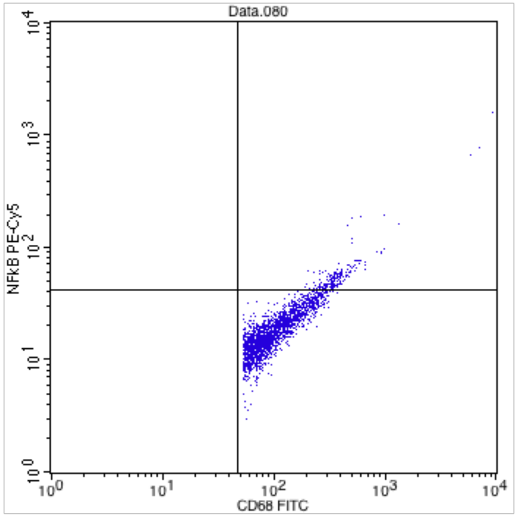

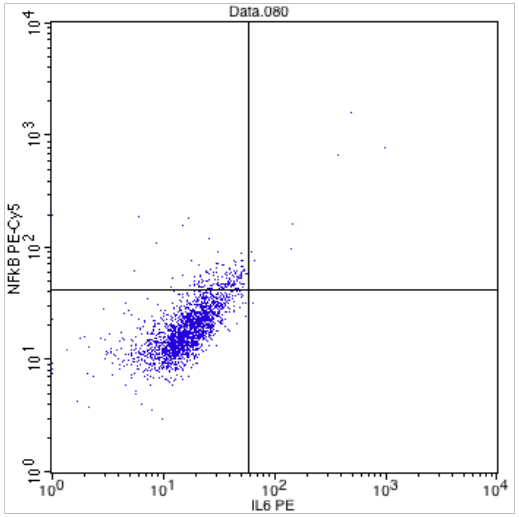

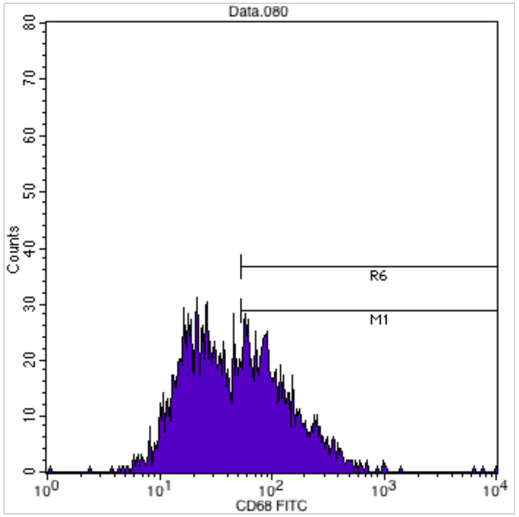

Quadrant Statistics

File: Data.080
Sample ID: 2.5
Acquisition Date: 18-Oct-24
Gate: G7
Gated Events: 2314
Total Events: 55393
X Parameter: CD68 FITC (Log)
Y Parameter: IL6 PE (Log)
Quad Location: 47, 58

Quad	Events	% Gated	% Total
UL	0	0.00	0.00
UR	9	0.39	0.02
LL	0	0.00	0.00
LR	2305	99.61	4.16

Quadrant Statistics

File: Data.080
Sample ID: 2.5
Acquisition Date: 18-Oct-24
Gate: G7
Gated Events: 2314
Total Events: 55393
X Parameter: CD68 FITC (Log)
Y Parameter: NFkB PE-Cy5 (Log)
Quad Location: 47, 42

Quad	Events	% Gated	% Total
UL	0	0.00	0.00
UR	181	7.82	0.33
LL	0	0.00	0.00
LR	2133	92.18	3.85

Quadrant Statistics

File: Data.080
Sample ID: 2.5
Acquisition Date: 18-Oct-24
Gate: G7
Gated Events: 2314
Total Events: 55393
X Parameter: IL6 PE (Log)
Y Parameter: NFkB PE-Cy5 (Log)
Quad Location: 58, 42

Quad	Events	% Gated	% Total
UL	174	7.52	0.31
UR	7	0.30	0.01
LL	2132	92.13	3.85
LR	1	0.04	0.00

Histogram Statistics

File: Data.080
Sample ID: 2.5
Acquisition Date: 18-Oct-24
Gate: G2
Gated Events: 5367
Total Events: 55393
X Parameter: CD68 FITC (Log)

Marker	Left, Right	Events	% Gated	% Total
All	1, 9910	5367	100.00	9.69
M1	53, 9910	2314	43.12	4.18

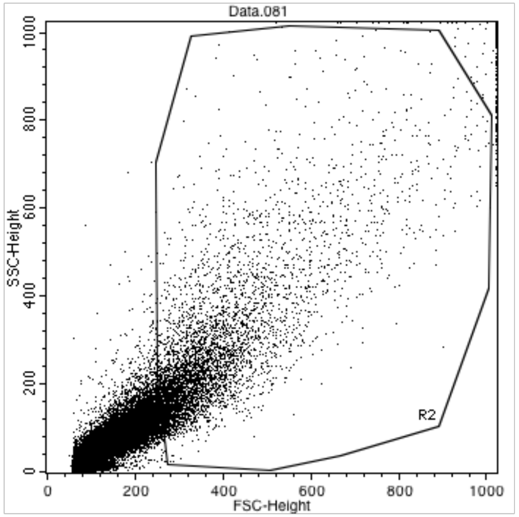

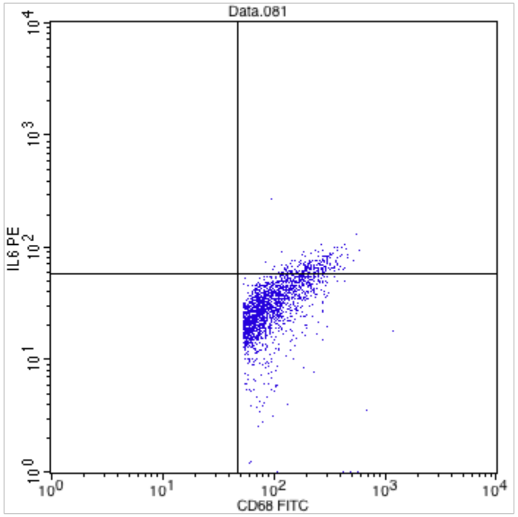

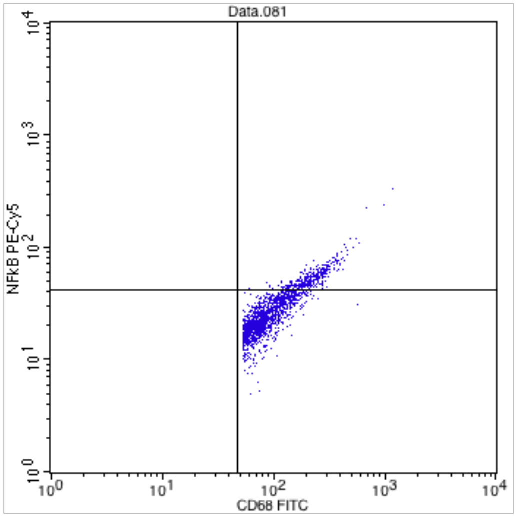

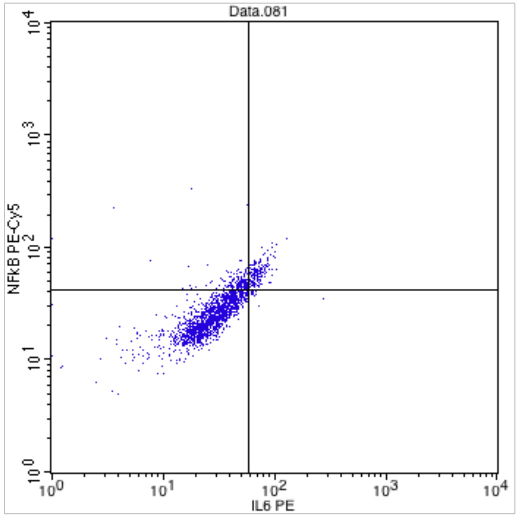

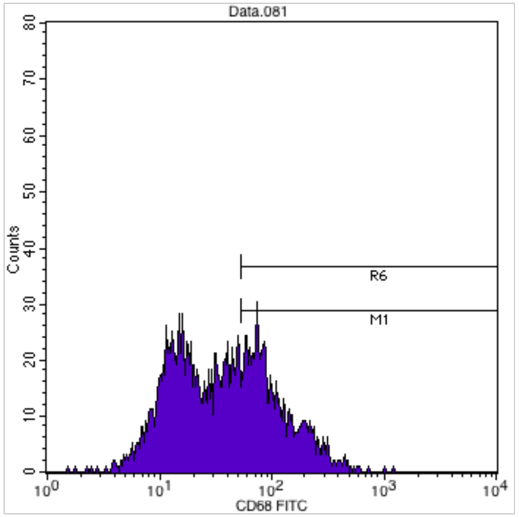

Quadrant Statistics

File: Data.081
Acquisition Date: 18-Oct-24
Gated Events: 1939
X Parameter: CD68 FITC (Log)
Quad Location: 47, 58
Sample ID: 2.6
Gate: G7
Total Events: 54001
Y Parameter: IL6 PE (Log)

Quad	Events	% Gated	% Total
UL	0	0.00	0.00
UR	195	10.06	0.36
LL	0	0.00	0.00
LR	1744	89.94	3.23

Quadrant Statistics

File: Data.081
Acquisition Date: 18-Oct-24
Gated Events: 1939
X Parameter: CD68 FITC (Log)
Quad Location: 47, 42
Sample ID: 2.6
Gate: G7
Total Events: 54001
Y Parameter: NFkB PE-Cy5 (Log)

Quad	Events	% Gated	% Total
UL	0	0.00	0.00
UR	361	18.62	0.67
LL	0	0.00	0.00
LR	1578	81.38	2.92

Quadrant Statistics

File: Data.081
Acquisition Date: 18-Oct-24
Gated Events: 1939
X Parameter: IL6 PE (Log)
Quad Location: 58, 42
Sample ID: 2.6
Gate: G7
Total Events: 54001
Y Parameter: NFkB PE-Cy5 (Log)

Quad	Events	% Gated	% Total
UL	189	9.75	0.35
UR	172	8.87	0.32
LL	1559	80.40	2.89
LR	19	0.98	0.04

Histogram Statistics

File: Data.081
Acquisition Date: 18-Oct-24
Gated Events: 5227
X Parameter: CD68 FITC (Log)
Sample ID: 2.6
Gate: G2
Total Events: 54001

Marker	Left, Right	Events	% Gated	% Total
All	1, 9910	5227	100.00	9.68
M1	53, 9910	1939	37.10	3.59

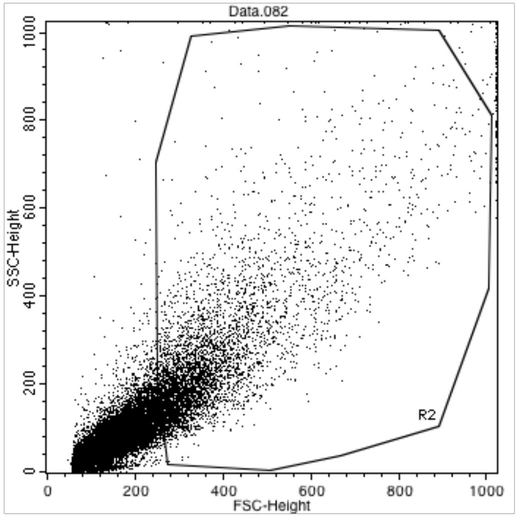

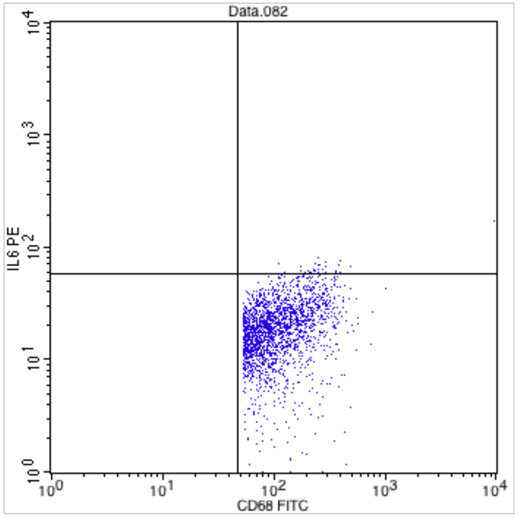

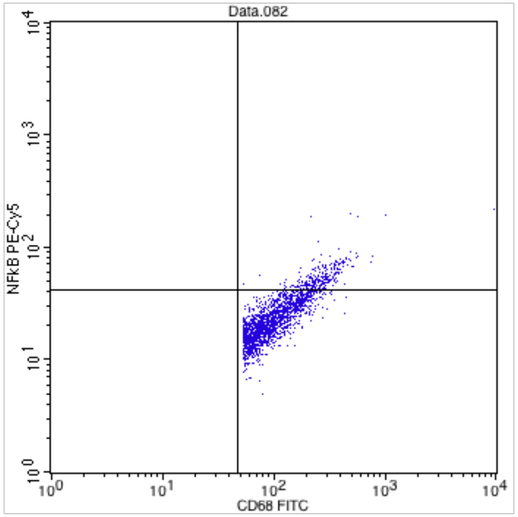

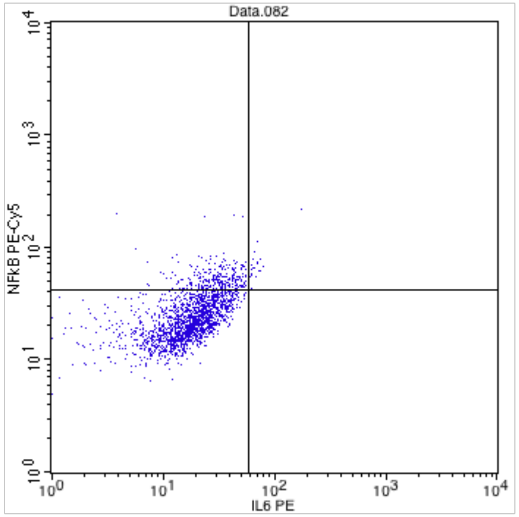

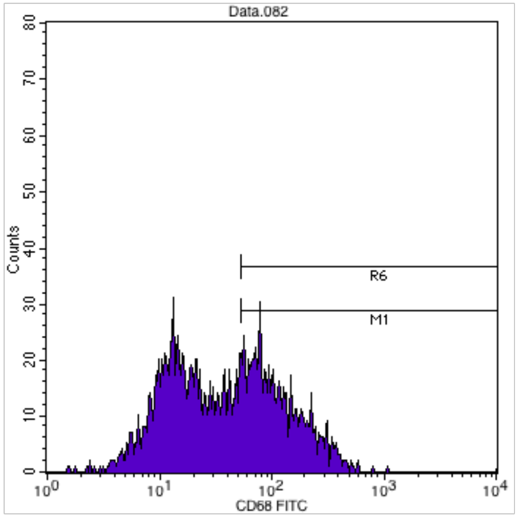

Quadrant Statistics

File: Data.082
Acquisition Date: 18-Oct-24
Gated Events: 2146
X Parameter: CD68 FITC (Log)
Quad Location: 47, 58
Sample ID: 3.4
Gate: G7
Total Events: 56097
Y Parameter: IL6 PE (Log)

Quad	Events	% Gated	% Total
UL	0	0.00	0.00
UR	30	1.40	0.05
LL	0	0.00	0.00
LR	2116	98.60	3.77

Quadrant Statistics

File: Data.082
Acquisition Date: 18-Oct-24
Gated Events: 2146
X Parameter: CD68 FITC (Log)
Quad Location: 47, 42
Sample ID: 3.4
Gate: G7
Total Events: 56097
Y Parameter: NFkB PE-Cy5 (Log)

Quad	Events	% Gated	% Total
UL	0	0.00	0.00
UR	299	13.93	0.53
LL	0	0.00	0.00
LR	1847	86.07	3.29

Quadrant Statistics

File: Data.082
Acquisition Date: 18-Oct-24
Gated Events: 2146
X Parameter: IL6 PE (Log)
Quad Location: 58, 42
Sample ID: 3.4
Gate: G7
Total Events: 56097
Y Parameter: NFkB PE-Cy5 (Log)

Quad	Events	% Gated	% Total
UL	272	12.67	0.48
UR	27	1.26	0.05
LL	1846	86.02	3.29
LR	1	0.05	0.00

Histogram Statistics

File: Data.082
Acquisition Date: 18-Oct-24
Gated Events: 5060
X Parameter: CD68 FITC (Log)
Sample ID: 3.4
Gate: G2
Total Events: 56097

Marker	Left, Right	Events	% Gated	% Total
All	1, 9910	5060	100.00	9.02
M1	53, 9910	2146	42.41	3.83

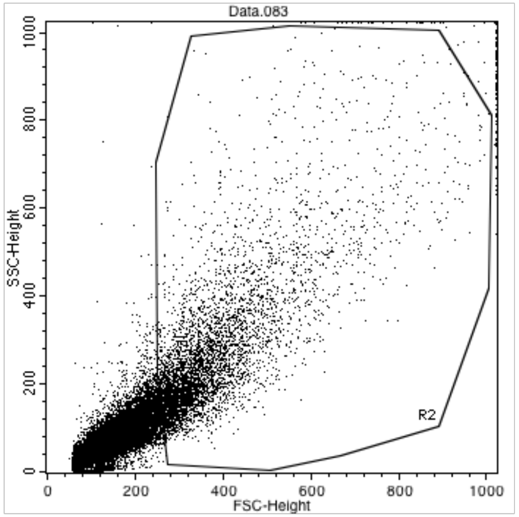

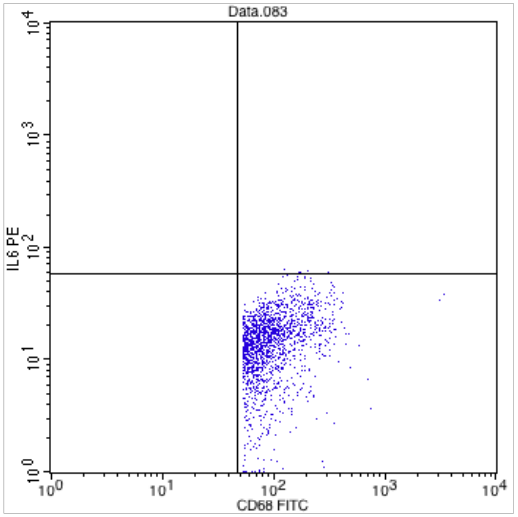

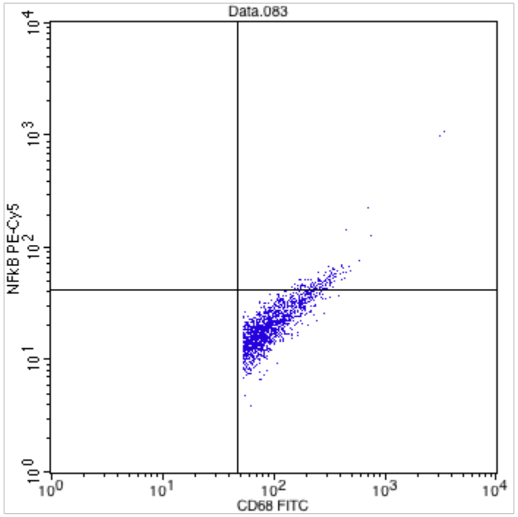

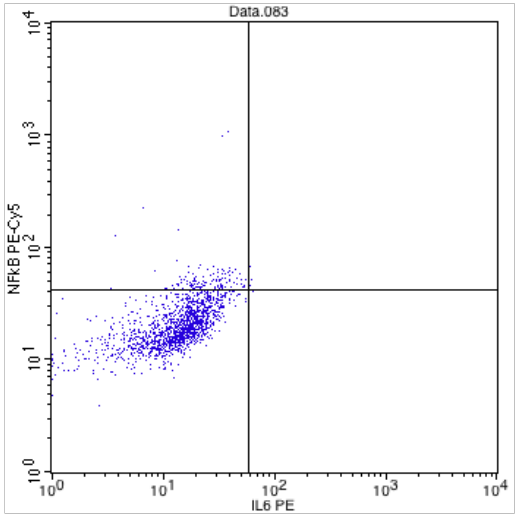

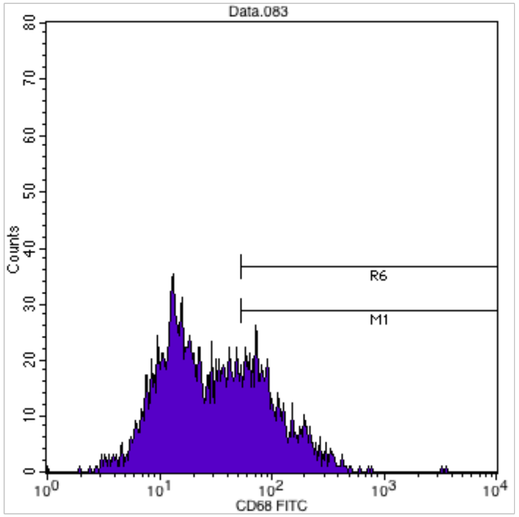

Quadrant Statistics

File: Data.083
Sample ID: 3.5
Acquisition Date: 18-Oct-24
Gate: G7
Gated Events: 1650
Total Events: 49298
X Parameter: CD68 FITC (Log)
Y Parameter: IL6 PE (Log)
Quad Location: 47, 58

Quad	Events	% Gated	% Total
UL	0	0.00	0.00
UR	6	0.36	0.01
LL	0	0.00	0.00
LR	1644	99.64	3.33

Quadrant Statistics

File: Data.083
Sample ID: 3.5
Acquisition Date: 18-Oct-24
Gate: G7
Gated Events: 1650
Total Events: 49298
X Parameter: CD68 FITC (Log)
Y Parameter: NFkB PE-Cy5 (Log)
Quad Location: 47, 42

Quad	Events	% Gated	% Total
UL	0	0.00	0.00
UR	133	8.06	0.27
LL	0	0.00	0.00
LR	1517	91.94	3.08

Quadrant Statistics

File: Data.083
Sample ID: 3.5
Acquisition Date: 18-Oct-24
Gate: G7
Gated Events: 1650
Total Events: 49298
X Parameter: IL6 PE (Log)
Y Parameter: NFkB PE-Cy5 (Log)
Quad Location: 58, 42

Quad	Events	% Gated	% Total
UL	129	7.82	0.26
UR	4	0.24	0.01
LL	1516	91.88	3.08
LR	1	0.06	0.00

Histogram Statistics

File: Data.083
Sample ID: 3.5
Acquisition Date: 18-Oct-24
Gate: G2
Gated Events: 5385
Total Events: 49298
X Parameter: CD68 FITC (Log)

Marker	Left, Right	Events	% Gated	% Total
All	1, 9910	5385	100.00	10.92
M1	53, 9910	1650	30.64	3.35

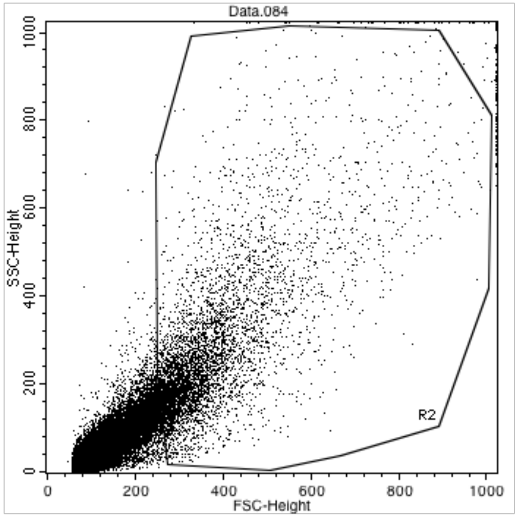

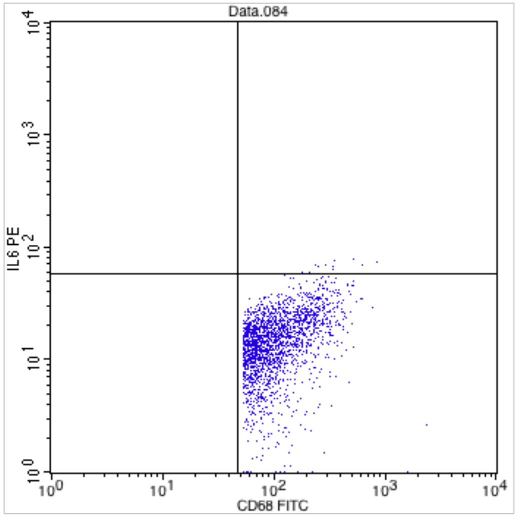

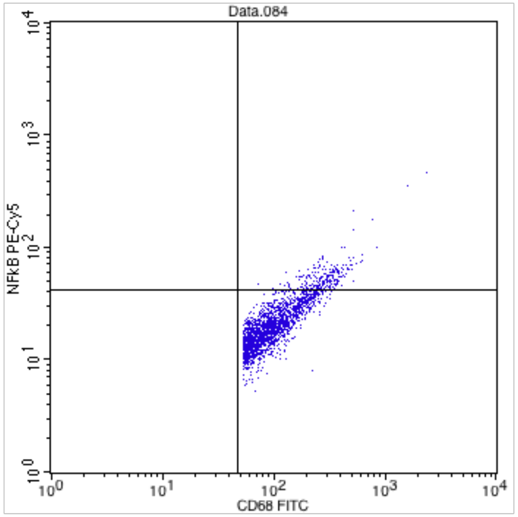

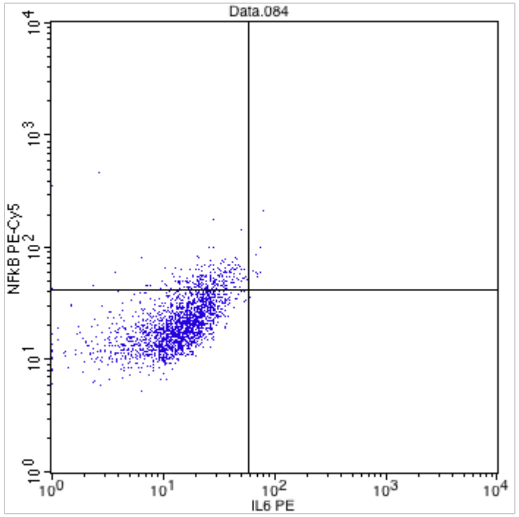

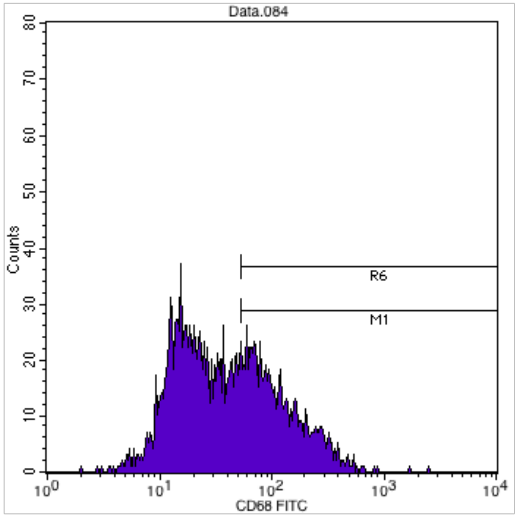

Quadrant Statistics

File: Data.084
Acquisition Date: 18-Oct-24
Gated Events: 2128
X Parameter: CD68 FITC (Log)
Quad Location: 47, 58
Sample ID: 4.4
Gate: G7
Total Events: 51076
Y Parameter: IL6 PE (Log)

Quad	Events	% Gated	% Total
UL	0	0.00	0.00
UR	11	0.52	0.02
LL	0	0.00	0.00
LR	2117	99.48	4.14

Quadrant Statistics

File: Data.084
Acquisition Date: 18-Oct-24
Gated Events: 2128
X Parameter: CD68 FITC (Log)
Quad Location: 47, 42
Sample ID: 4.4
Gate: G7
Total Events: 51076
Y Parameter: NFkB PE-Cy5 (Log)

Quad	Events	% Gated	% Total
UL	0	0.00	0.00
UR	239	11.23	0.47
LL	0	0.00	0.00
LR	1889	88.77	3.70

Quadrant Statistics

File: Data.084
Acquisition Date: 18-Oct-24
Gated Events: 2128
X Parameter: IL6 PE (Log)
Quad Location: 58, 42
Sample ID: 4.4
Gate: G7
Total Events: 51076
Y Parameter: NFkB PE-Cy5 (Log)

Quad	Events	% Gated	% Total
UL	229	10.76	0.45
UR	10	0.47	0.02
LL	1888	88.72	3.70
LR	1	0.05	0.00

Histogram Statistics

File: Data.084
Acquisition Date: 18-Oct-24
Gated Events: 5511
X Parameter: CD68 FITC (Log)
Sample ID: 4.4
Gate: G2
Total Events: 51076

Marker	Left, Right	Events	% Gated	% Total
All	1, 9910	5511	100.00	10.79
M1	53, 9910	2128	38.61	4.17

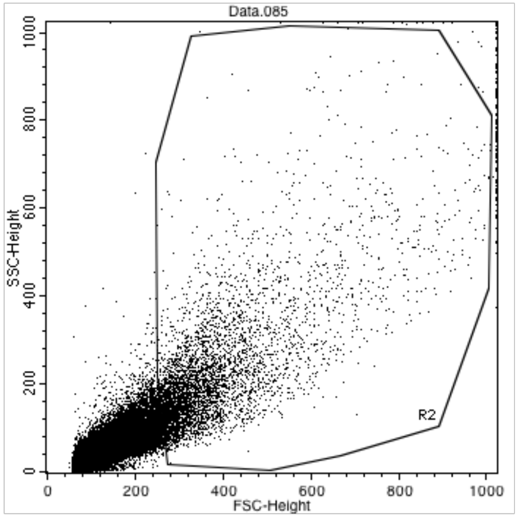

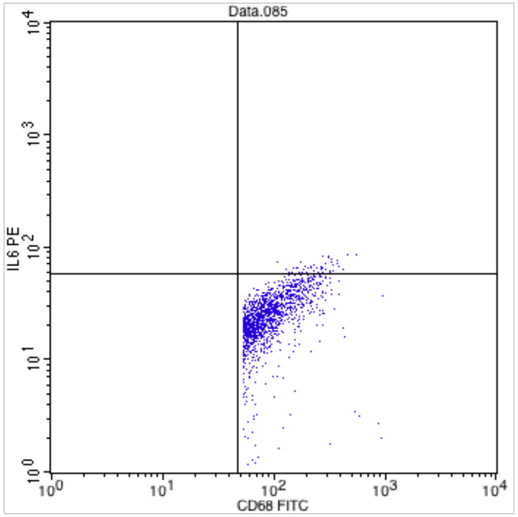

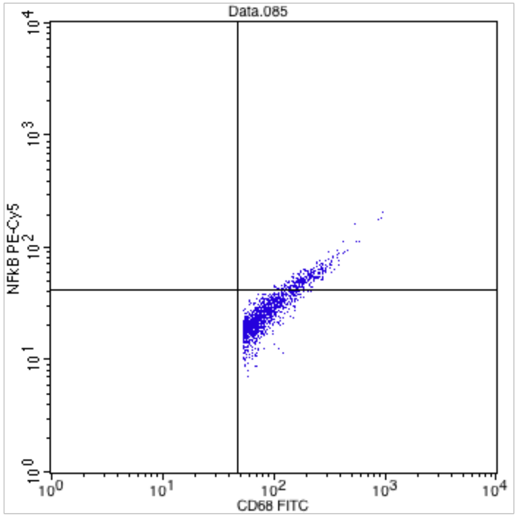

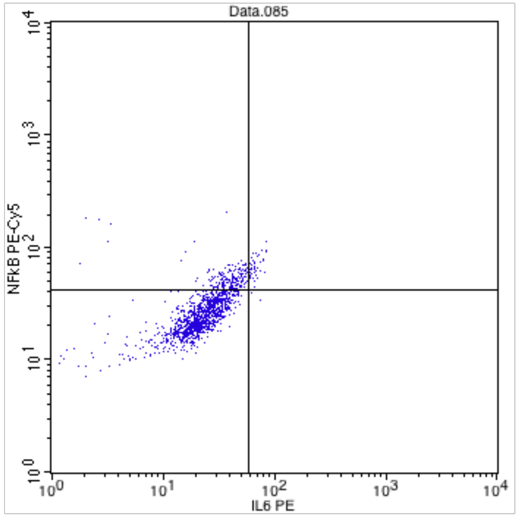

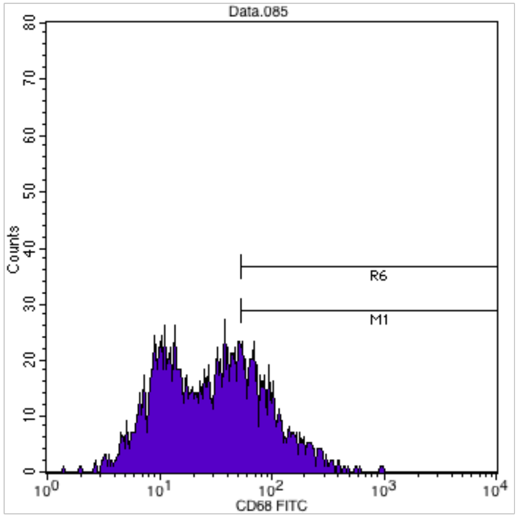

Quadrant Statistics

File: Data.085
Acquisition Date: 18-Oct-24
Gated Events: 1448
X Parameter: CD68 FITC (Log)
Quad Location: 47, 58
Sample ID: 5.4
Gate: G7
Total Events: 54445
Y Parameter: IL6 PE (Log)

Quad	Events	% Gated	% Total
UL	0	0.00	0.00
UR	45	3.11	0.08
LL	0	0.00	0.00
LR	1403	96.89	2.58

Quadrant Statistics

File: Data.085
Acquisition Date: 18-Oct-24
Gated Events: 1448
X Parameter: CD68 FITC (Log)
Quad Location: 47, 42
Sample ID: 5.4
Gate: G7
Total Events: 54445
Y Parameter: NFkB PE-Cy5 (Log)

Quad	Events	% Gated	% Total
UL	0	0.00	0.00
UR	261	18.02	0.48
LL	0	0.00	0.00
LR	1187	81.98	2.18

Quadrant Statistics

File: Data.085
Acquisition Date: 18-Oct-24
Gated Events: 1448
X Parameter: IL6 PE (Log)
Quad Location: 58, 42
Sample ID: 5.4
Gate: G7
Total Events: 54445
Y Parameter: NFkB PE-Cy5 (Log)

Quad	Events	% Gated	% Total
UL	220	15.19	0.40
UR	41	2.83	0.08
LL	1185	81.84	2.18
LR	2	0.14	0.00

Histogram Statistics

File: Data.085
Acquisition Date: 18-Oct-24
Gated Events: 4892
X Parameter: CD68 FITC (Log)
Sample ID: 5.4
Gate: G2
Total Events: 54445

Marker	Left, Right	Events	% Gated	% Total
All	1, 9910	4892	100.00	8.99
M1	53, 9910	1448	29.60	2.66

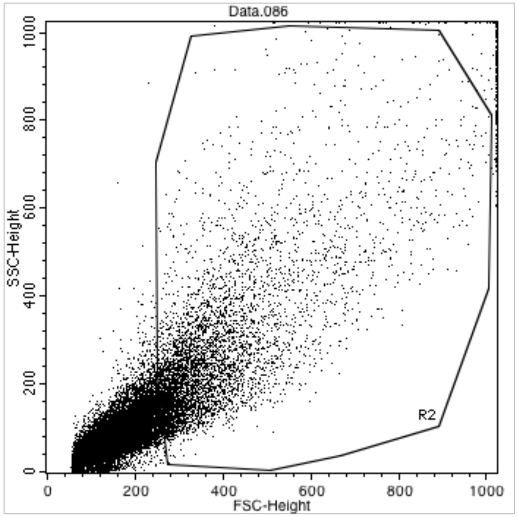

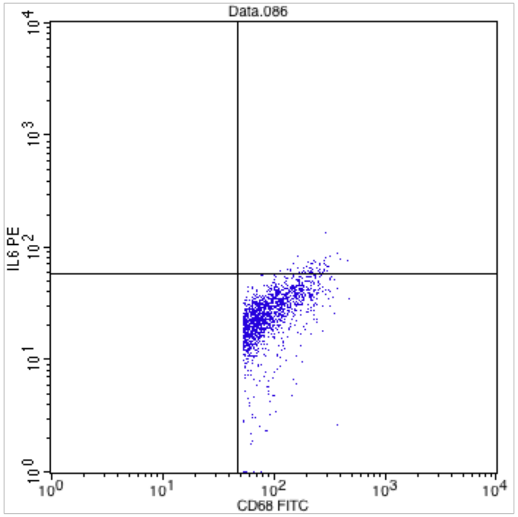

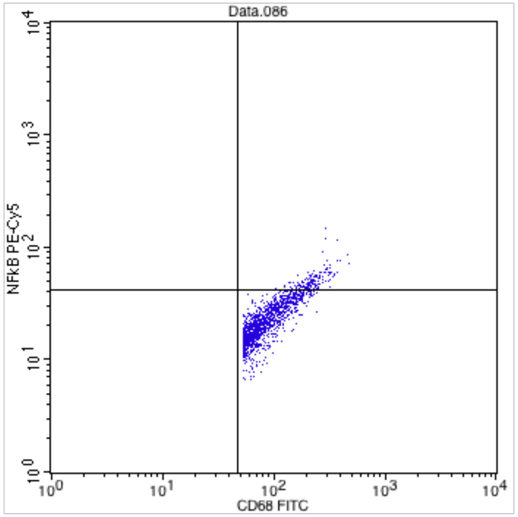

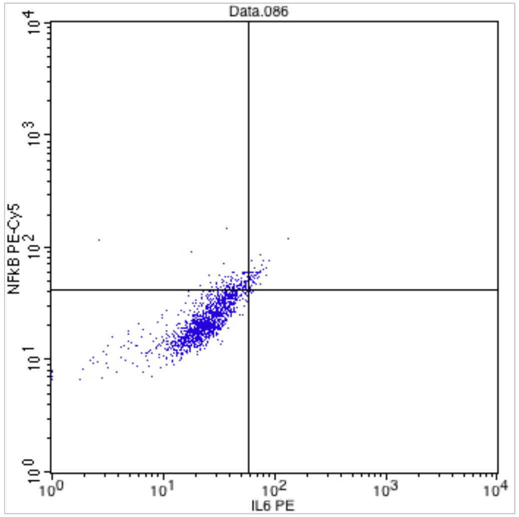

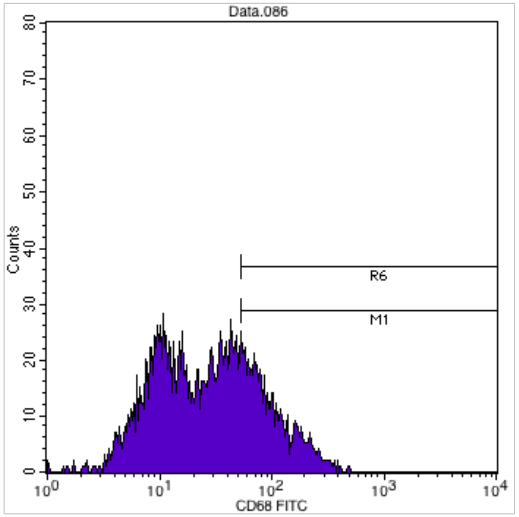

Quadrant Statistics

File: Data.086
Acquisition Date: 18-Oct-24
Gated Events: 1560
X Parameter: CD68 FITC (Log)
Quad Location: 47, 58

Sample ID: 5.5
Gate: G7
Total Events: 51322
Y Parameter: IL6 PE (Log)

Quad	Events	% Gated	% Total
UL	0	0.00	0.00
UR	52	3.33	0.10
LL	0	0.00	0.00
LR	1508	96.67	2.94

Quadrant Statistics

File: Data.086
Acquisition Date: 18-Oct-24
Gated Events: 1560
X Parameter: CD68 FITC (Log)
Quad Location: 47, 42

Sample ID: 5.5
Gate: G7
Total Events: 51322
Y Parameter: NFkB PE-Cy5 (Log)

Quad	Events	% Gated	% Total
UL	0	0.00	0.00
UR	154	9.87	0.30
LL	0	0.00	0.00
LR	1406	90.13	2.74

Quadrant Statistics

File: Data.086
Acquisition Date: 18-Oct-24
Gated Events: 1560
X Parameter: IL6 PE (Log)
Quad Location: 58, 42

Sample ID: 5.5
Gate: G7
Total Events: 51322
Y Parameter: NFkB PE-Cy5 (Log)

Quad	Events	% Gated	% Total
UL	111	7.12	0.22
UR	43	2.76	0.08
LL	1402	89.87	2.73
LR	4	0.26	0.01

Histogram Statistics

File: Data.086
Acquisition Date: 18-Oct-24
Gated Events: 5567
X Parameter: CD68 FITC (Log)

Sample ID: 5.5
Gate: G2
Total Events: 51322

Marker	Left, Right	Events	% Gated	% Total
All	1, 9910	5567	100.00	10.85
M1	53, 9910	1560	28.02	3.04

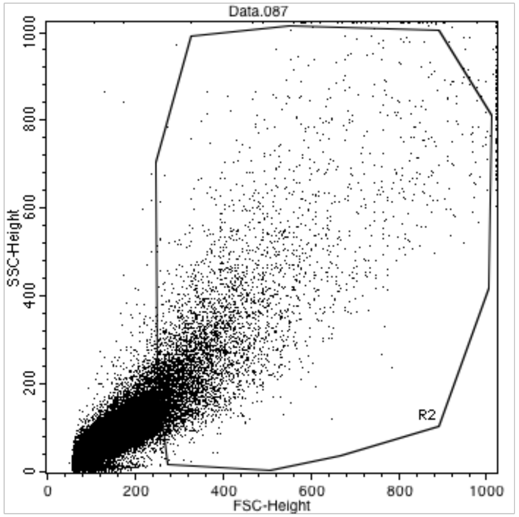

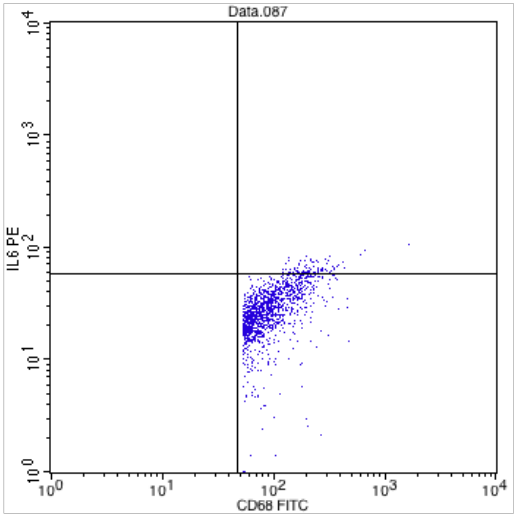

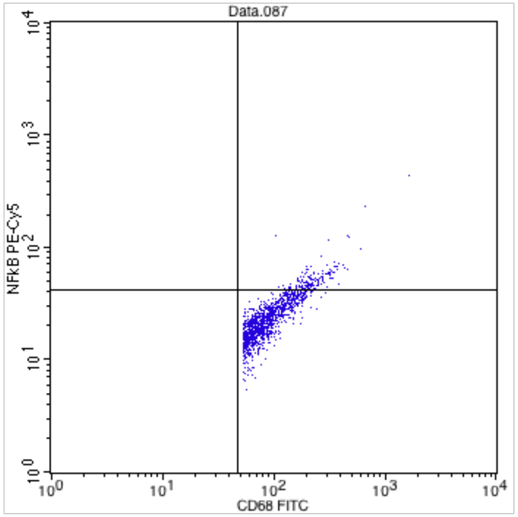

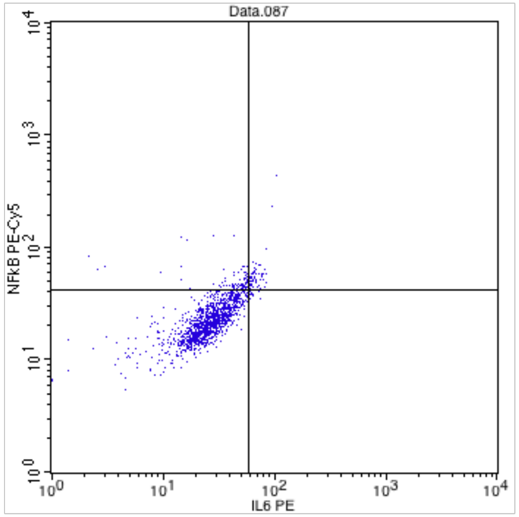

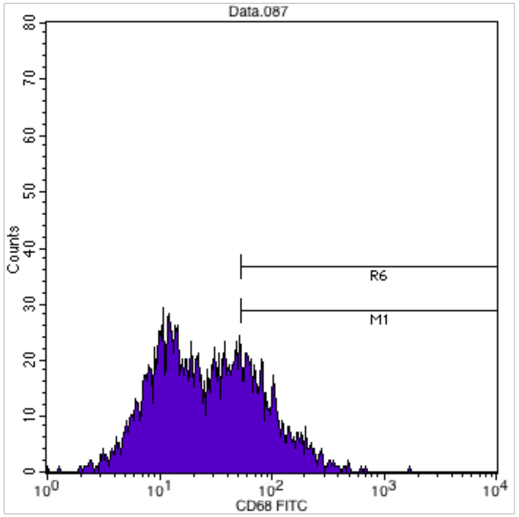

Quadrant Statistics

File: Data.087
Acquisition Date: 18-Oct-24
Gated Events: 1421
X Parameter: CD68 FITC (Log)
Quad Location: 47, 58
Sample ID: 5.6
Gate: G7
Total Events: 52061
Y Parameter: IL6 PE (Log)

Quad	Events	% Gated	% Total
UL	0	0.00	0.00
UR	81	5.70	0.16
LL	0	0.00	0.00
LR	1340	94.30	2.57

Quadrant Statistics

File: Data.087
Acquisition Date: 18-Oct-24
Gated Events: 1421
X Parameter: CD68 FITC (Log)
Quad Location: 47, 42
Sample ID: 5.6
Gate: G7
Total Events: 52061
Y Parameter: NFkB PE-Cy5 (Log)

Quad	Events	% Gated	% Total
UL	0	0.00	0.00
UR	153	10.77	0.29
LL	0	0.00	0.00
LR	1268	89.23	2.44

Quadrant Statistics

File: Data.087
Acquisition Date: 18-Oct-24
Gated Events: 1421
X Parameter: IL6 PE (Log)
Quad Location: 58, 42
Sample ID: 5.6
Gate: G7
Total Events: 52061
Y Parameter: NFkB PE-Cy5 (Log)

Quad	Events	% Gated	% Total
UL	88	6.19	0.17
UR	65	4.57	0.12
LL	1255	88.32	2.41
LR	13	0.91	0.02

Histogram Statistics

File: Data.087
Acquisition Date: 18-Oct-24
Gated Events: 5463
X Parameter: CD68 FITC (Log)
Sample ID: 5.6
Gate: G2
Total Events: 52061

Marker	Left, Right	Events	% Gated	% Total
All	1, 9910	5463	100.00	10.49
M1	53, 9910	1421	26.01	2.73

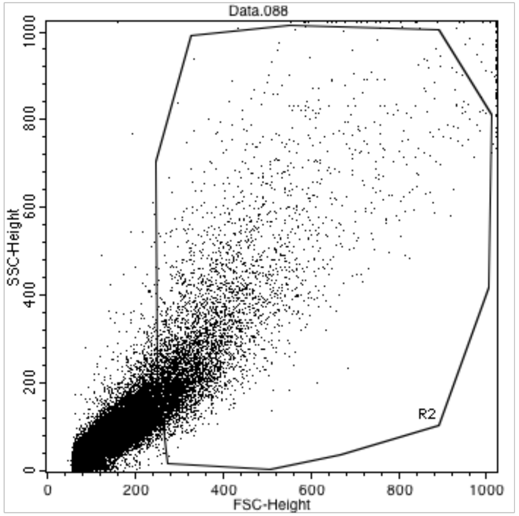

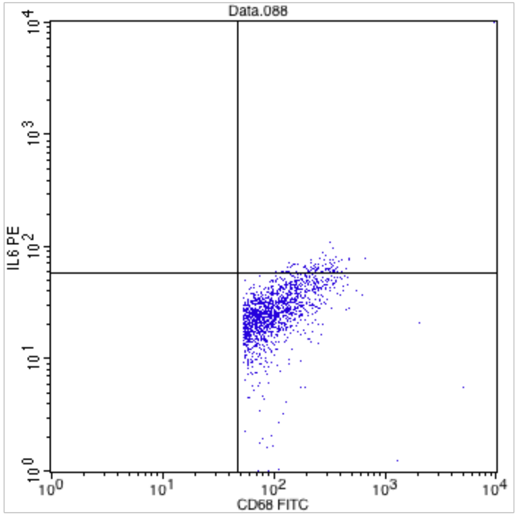

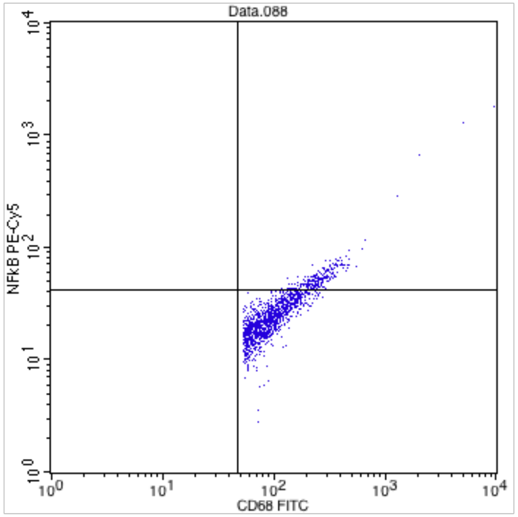

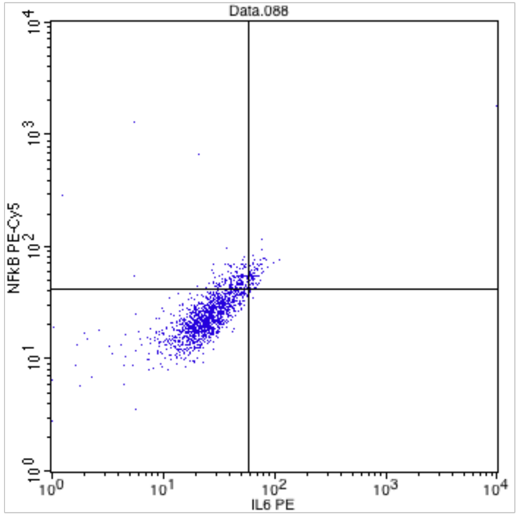

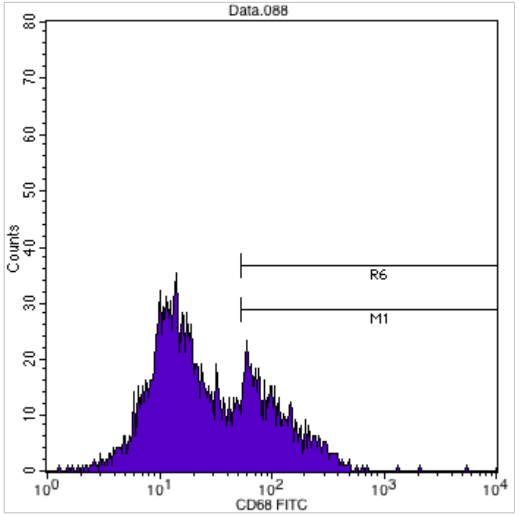

Quadrant Statistics

File: Data.088
Acquisition Date: 18-Oct-24
Gated Events: 1530
X Parameter: CD68 FITC (Log)
Quad Location: 47, 58
Sample ID: 6.4
Gate: G7
Total Events: 59042
Y Parameter: IL6 PE (Log)

Quad	Events	% Gated	% Total
UL	0	0.00	0.00
UR	84	5.49	0.14
LL	0	0.00	0.00
LR	1446	94.51	2.45

Quadrant Statistics

File: Data.088
Acquisition Date: 18-Oct-24
Gated Events: 1530
X Parameter: CD68 FITC (Log)
Quad Location: 47, 42
Sample ID: 6.4
Gate: G7
Total Events: 59042
Y Parameter: NFkB PE-Cy5 (Log)

Quad	Events	% Gated	% Total
UL	0	0.00	0.00
UR	228	14.90	0.39
LL	0	0.00	0.00
LR	1302	85.10	2.21

Quadrant Statistics

File: Data.088
Acquisition Date: 18-Oct-24
Gated Events: 1530
X Parameter: IL6 PE (Log)
Quad Location: 58, 42
Sample ID: 6.4
Gate: G7
Total Events: 59042
Y Parameter: NFkB PE-Cy5 (Log)

Quad	Events	% Gated	% Total
UL	152	9.93	0.26
UR	76	4.97	0.13
LL	1297	84.77	2.20
LR	5	0.33	0.01

Histogram Statistics

File: Data.088
Acquisition Date: 18-Oct-24
Gated Events: 5279
X Parameter: CD68 FITC (Log)
Sample ID: 6.4
Gate: G2
Total Events: 59042

Marker	Left, Right	Events	% Gated	% Total
All	1, 9910	5279	100.00	8.94
M1	53, 9910	1530	28.98	2.59

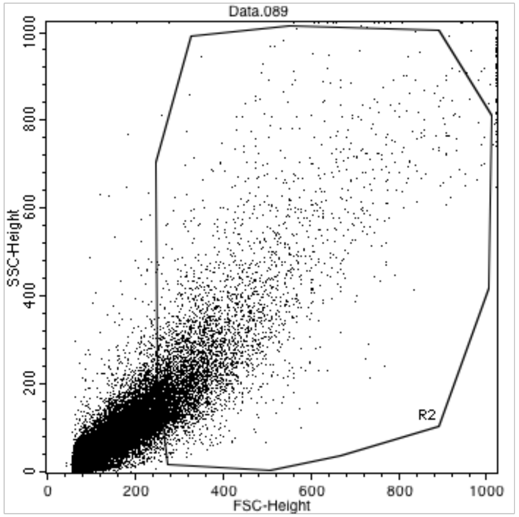

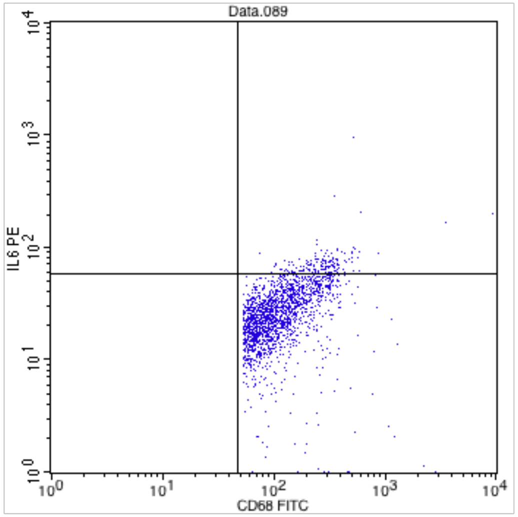

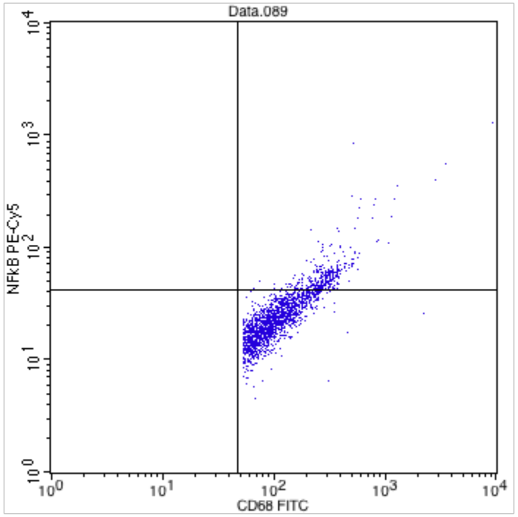

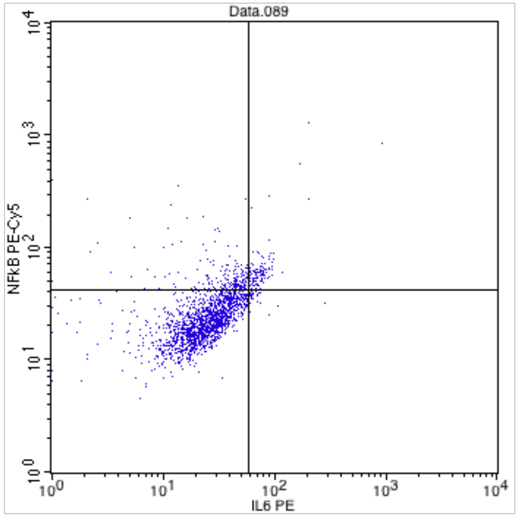

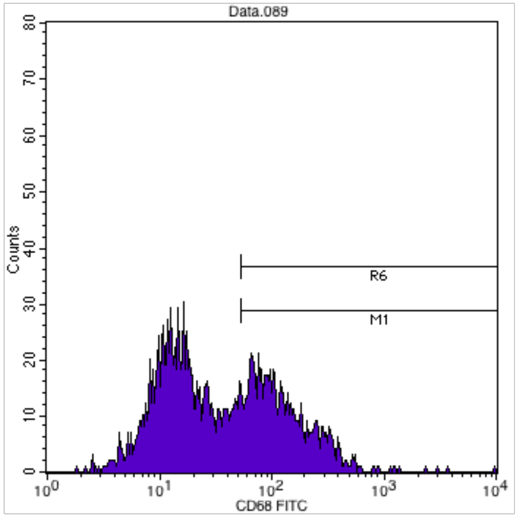

Quadrant Statistics

File: Data.089
Acquisition Date: 18-Oct-24
Gated Events: 1969
X Parameter: CD68 FITC (Log)
Quad Location: 47, 58

Sample ID: 7.4
Gate: G7
Total Events: 62873
Y Parameter: IL6 PE (Log)

Quad	Events	% Gated	% Total
UL	0	0.00	0.00
UR	160	8.13	0.25
LL	0	0.00	0.00
LR	1809	91.87	2.88

Quadrant Statistics

File: Data.089
Acquisition Date: 18-Oct-24
Gated Events: 1969
X Parameter: CD68 FITC (Log)
Quad Location: 47, 42

Sample ID: 7.4
Gate: G7
Total Events: 62873
Y Parameter: NFkB PE-Cy5 (Log)

Quad	Events	% Gated	% Total
UL	0	0.00	0.00
UR	328	16.66	0.52
LL	0	0.00	0.00
LR	1641	83.34	2.61

Quadrant Statistics

File: Data.089
Acquisition Date: 18-Oct-24
Gated Events: 1969
X Parameter: IL6 PE (Log)
Quad Location: 58, 42

Sample ID: 7.4
Gate: G7
Total Events: 62873
Y Parameter: NFkB PE-Cy5 (Log)

Quad	Events	% Gated	% Total
UL	204	10.36	0.32
UR	124	6.30	0.20
LL	1610	81.77	2.56
LR	31	1.57	0.05

Histogram Statistics

File: Data.089
Acquisition Date: 18-Oct-24
Gated Events: 4932
X Parameter: CD68 FITC (Log)

Sample ID: 7.4
Gate: G2
Total Events: 62873

Marker	Left, Right	Events	% Gated	% Total
All	1, 9910	4932	100.00	7.84
M1	53, 9910	1969	39.92	3.13

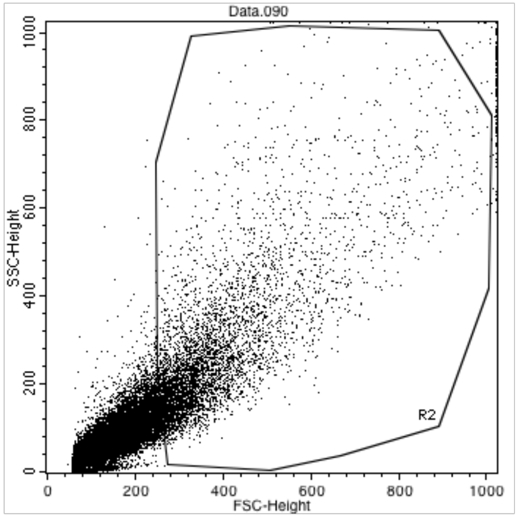

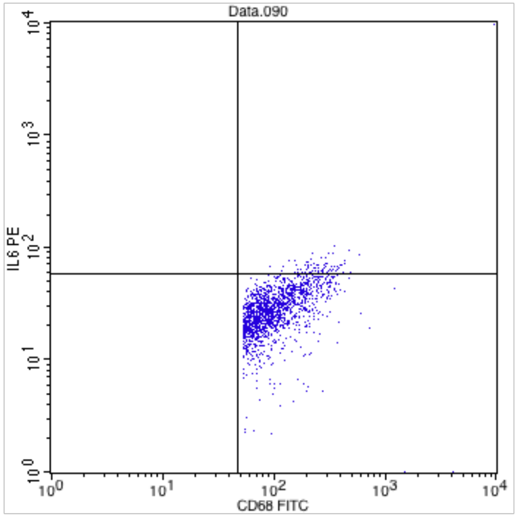

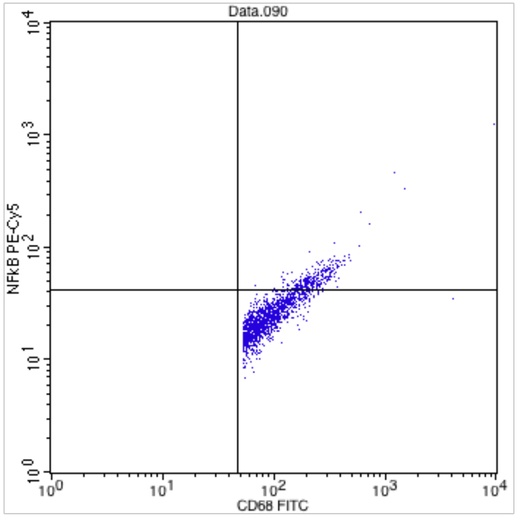

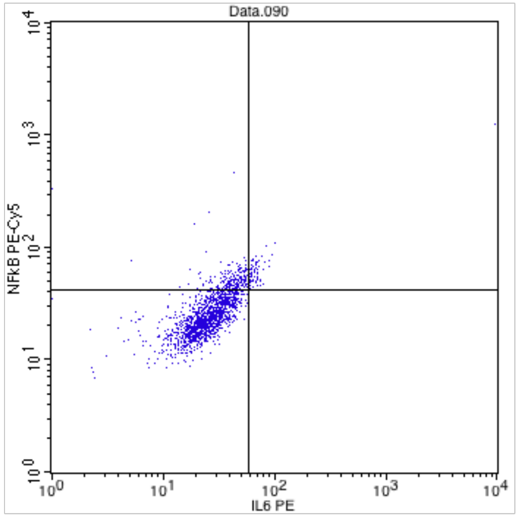

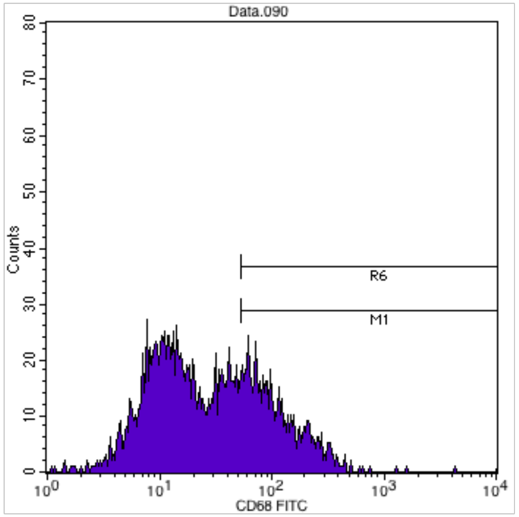

Quadrant Statistics

File: Data.090
Acquisition Date: 18-Oct-24
Gated Events: 1724
X Parameter: CD68 FITC (Log)
Quad Location: 47, 58

Sample ID: 7.6
Gate: G7
Total Events: 53924
Y Parameter: IL6 PE (Log)

Quad	Events	% Gated	% Total
UL	0	0.00	0.00
UR	76	4.41	0.14
LL	0	0.00	0.00
LR	1648	95.59	3.06

Quadrant Statistics

File: Data.090
Acquisition Date: 18-Oct-24
Gated Events: 1724
X Parameter: CD68 FITC (Log)
Quad Location: 47, 42

Sample ID: 7.6
Gate: G7
Total Events: 53924
Y Parameter: NFkB PE-Cy5 (Log)

Quad	Events	% Gated	% Total
UL	0	0.00	0.00
UR	279	16.18	0.52
LL	0	0.00	0.00
LR	1445	83.82	2.68

Quadrant Statistics

File: Data.090
Acquisition Date: 18-Oct-24
Gated Events: 1724
X Parameter: IL6 PE (Log)
Quad Location: 58, 42

Sample ID: 7.6
Gate: G7
Total Events: 53924
Y Parameter: NFkB PE-Cy5 (Log)

Quad	Events	% Gated	% Total
UL	207	12.01	0.38
UR	72	4.18	0.13
LL	1442	83.64	2.67
LR	3	0.17	0.01

Histogram Statistics

File: Data.090
Acquisition Date: 18-Oct-24
Gated Events: 5395
X Parameter: CD68 FITC (Log)

Sample ID: 7.6
Gate: G2
Total Events: 53924

Marker	Left, Right	Events	% Gated	% Total
All	1, 9910	5395	100.00	10.00
M1	53, 9910	1724	31.96	3.20
