# Supplementary material for: Kratom (Mitragyna speciosa) as a Phytochemical-Based Natural Product Exhibiting Opioid-like Analgesic Effects with Reduced Tolerance and Dependence Liability via TLR4-Associated Neuroimmune Modulation
Source: Molecules. 2026 Apr 26;31(9):1428. doi: 10.3390/molecules31091428 (PMC13164666; doi:10.3390/molecules31091428)
Supplement: Supplementary file 1 [file molecules-31-01428-s001.zip › Kratom_HRMS Results_EtOH96.pdf]

Compounds

Unit Laboratorium Riset Unggulan IPB

Structure	Name	RT [min]	Formula	Molecular Weight	Group Areas
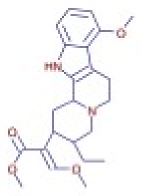	Mitragynine	11.84	C23 H30 N2 O4	398.2206	8.82e10

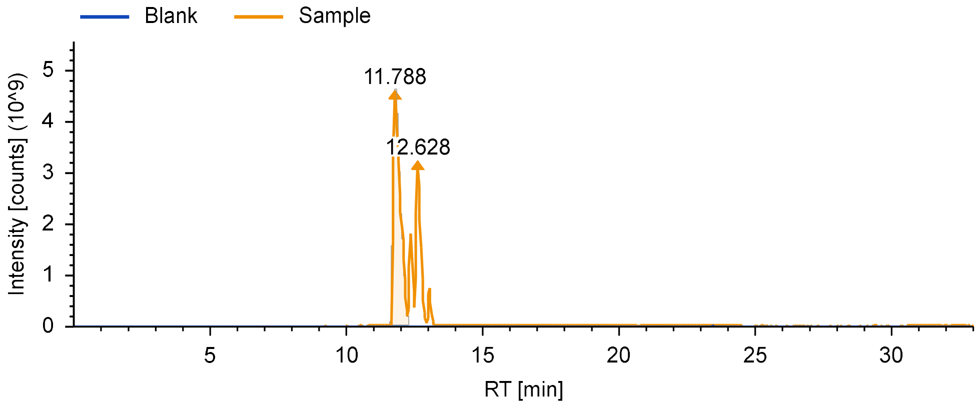

KratomEtOH96 (F3) #4076, RT=11.858 min, MS1, FTMS (+)
C23 H30 N2 O4 as [M+H]+1

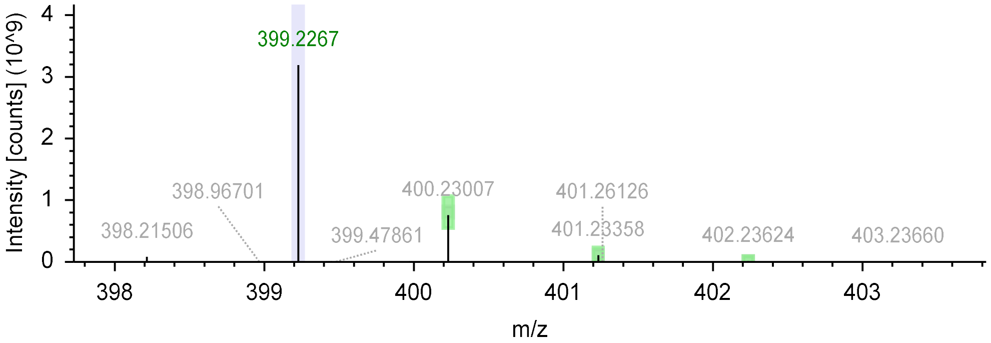

KratomEtOH96 (F3) #4077, RT=11.863 min, MS2, FTMS (+), (HCD, DDA, 399.2271@(18;35;53), +1)

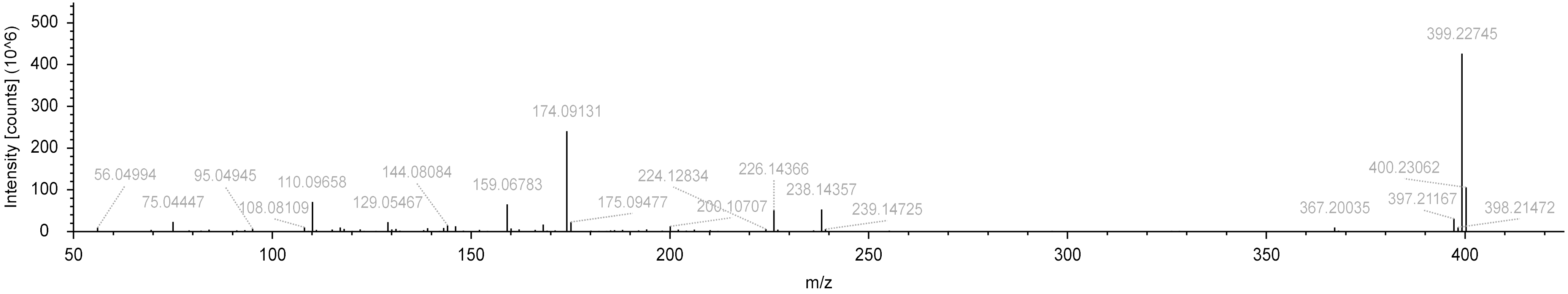

© Reported with Compound Discoverer 3.2

Structure	Name	RT [min]	Formula	Molecular Weight	Group Areas

© Reported with Compound Discoverer 3.2

Structure	Name	RT [min]	Formula	Molecular Weight	Group Areas
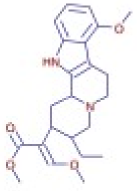	Mitragynine	12.65	C23 H30 N2 O4	398.2206	3.69e10

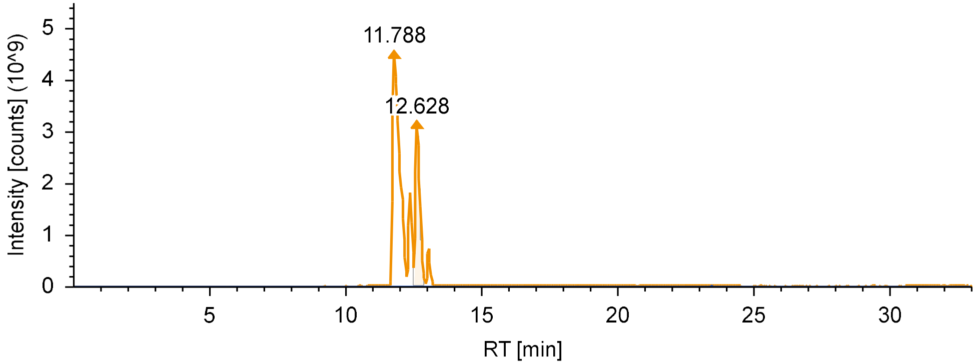

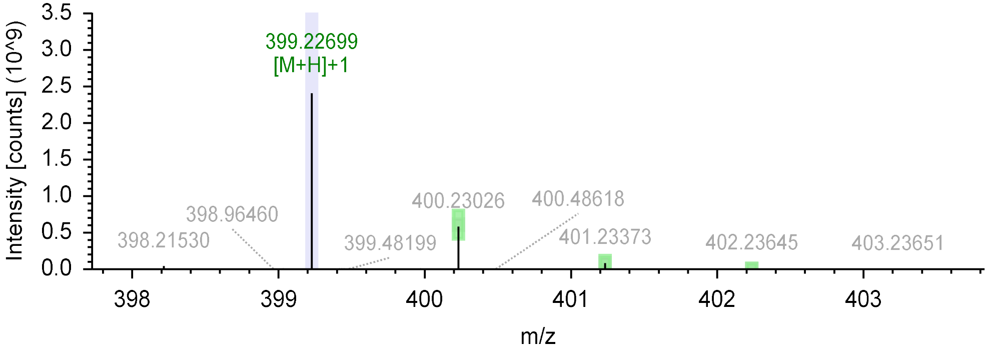

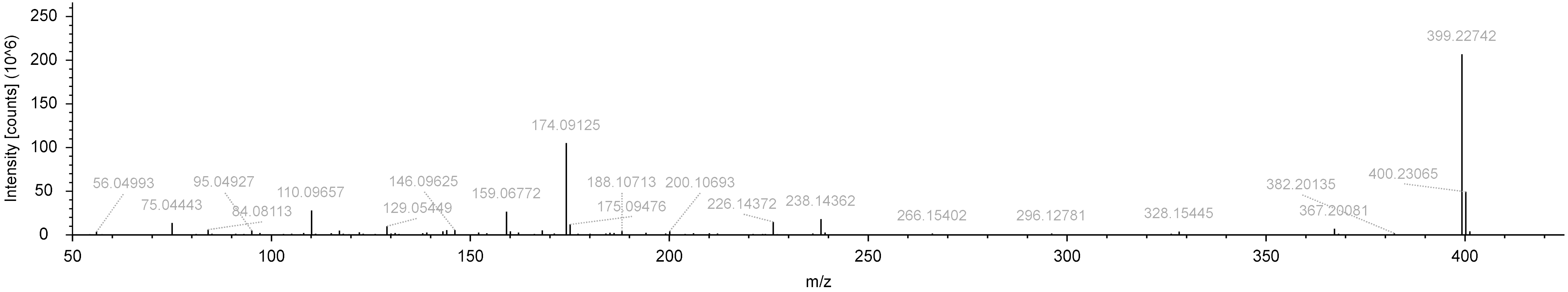

© Reported with Compound Discoverer 3.2

Compounds

Unit Laboratorium Riset Unggulan IPB

Structure	Name	RT [min]	Formula	Molecular Weight	Group Areas
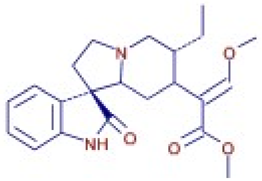	Rhynchophylline	10.14	C22 H28 N2 O4	384.2049	2.60e10

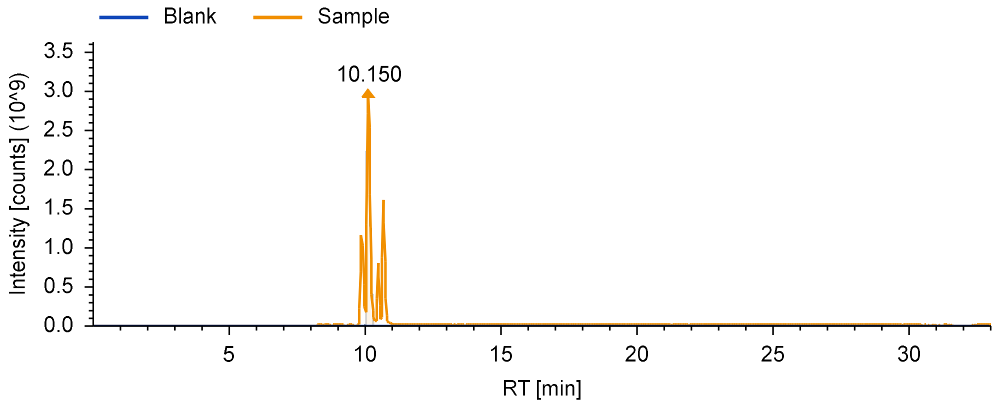

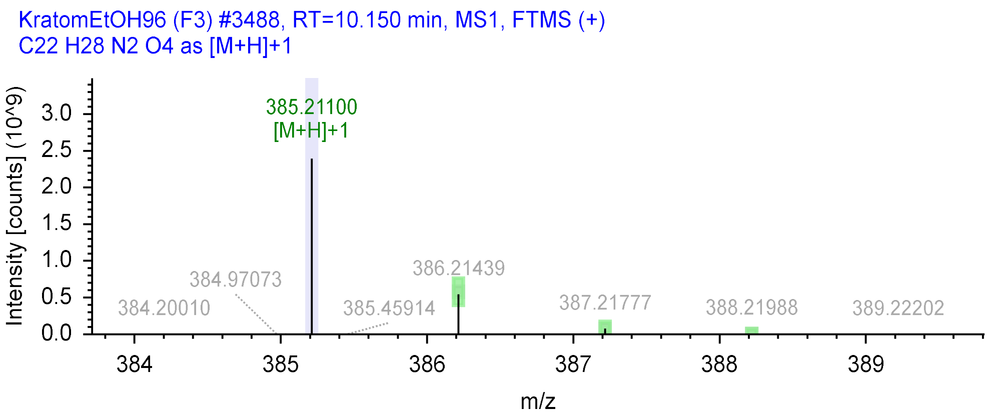

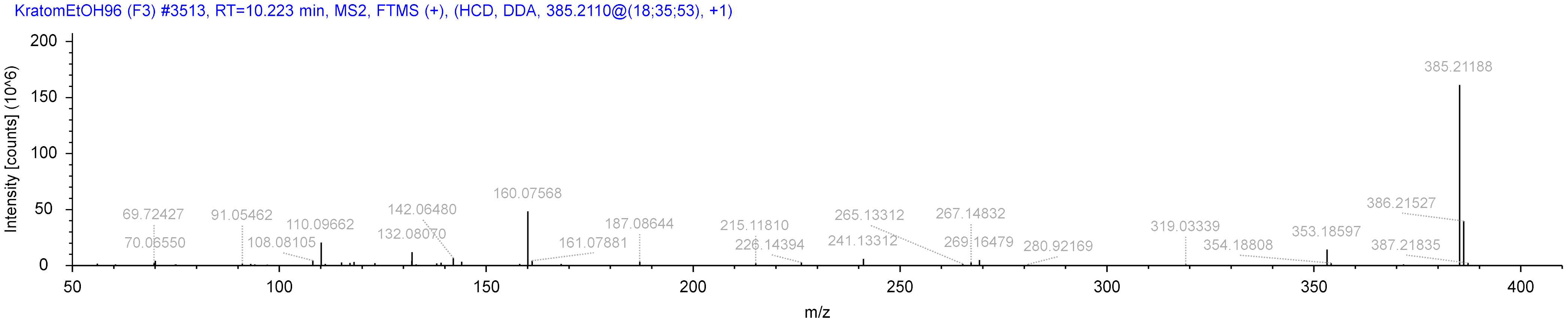

© Reported with Compound Discoverer 3.2

Structure	Name	RT [min]	Formula	Molecular Weight	Group Areas
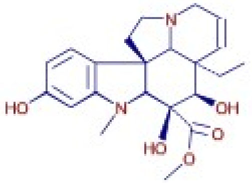	Methyl (2beta,3beta,4beta,5alpha,12beta,19alpha)-3,4,16-trihydroxy-1-methyl-6,7-didehydroaspidospermidine-3-carboxylate	9.57	C22 H28 N2 O5	400.1998	2.57e10

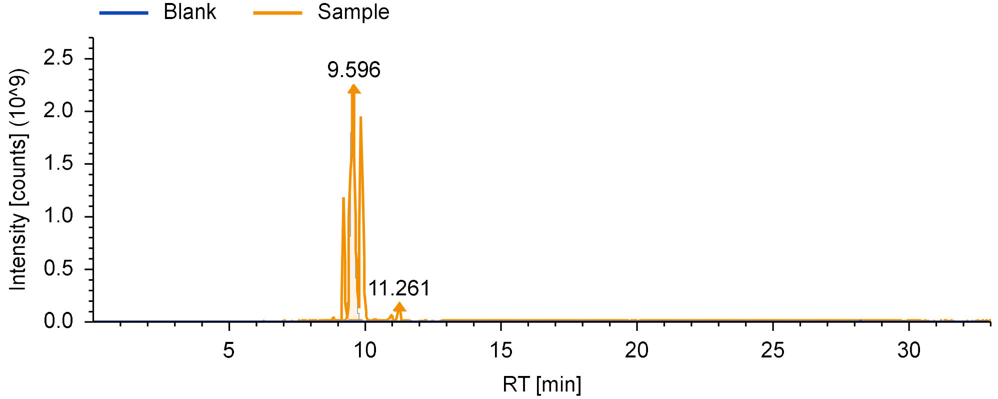

KratomEtOH96 (F3) #3284, RT=9.560 min, MS1, FTMS (+)
C22 H28 N2 O5 as [M+H]+1

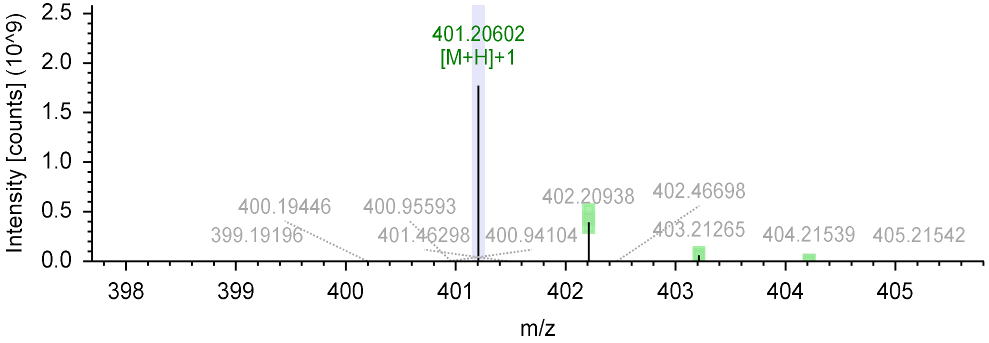

KratomEtOH96 (F3) #3273, RT=9.531 min, MS2, FTMS (+), (HCD, DDA, 401.2061@(18;35;53), +1)

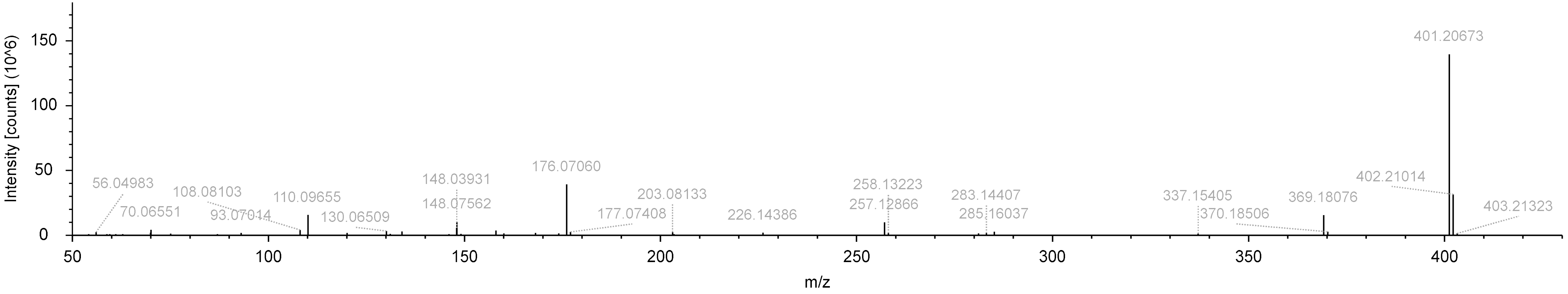

© Reported with Compound Discoverer 3.2

Structure	Name	RT [min]	Formula	Molecular Weight	Group Areas
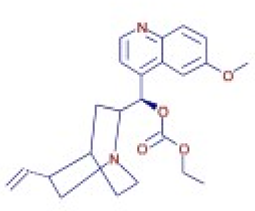	Ethyl (4beta,8alpha,9R)-6'-methoxycinchonan-9-yl carbonate	13.29	C23 H28 N2 O4	396.2049	1.76e10

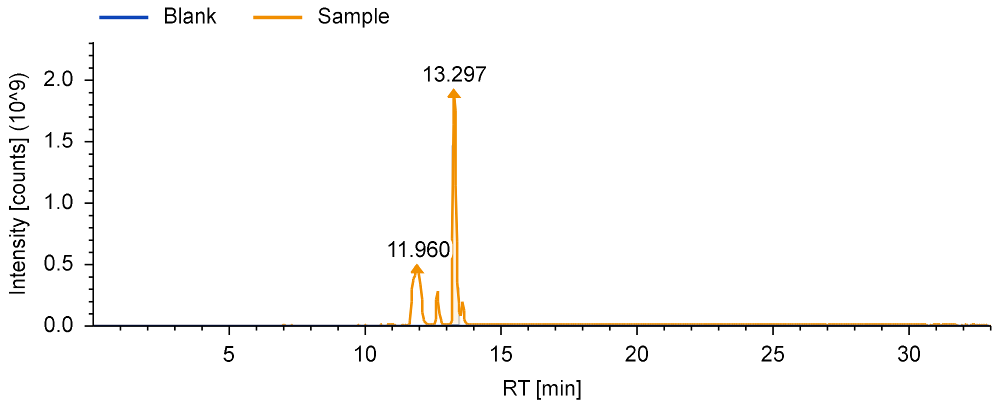

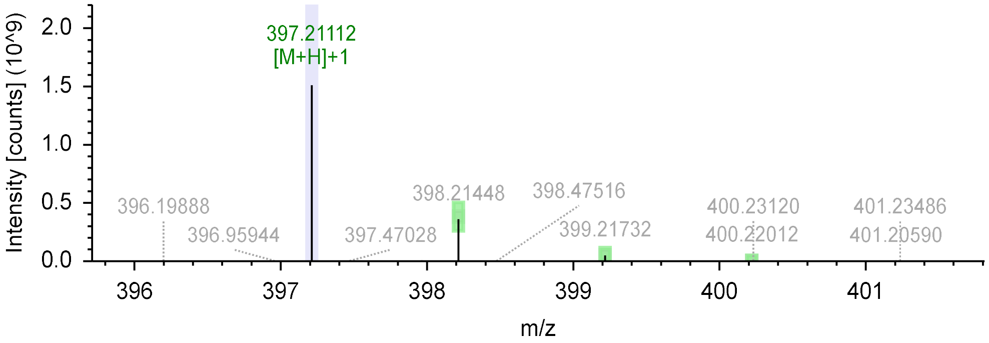

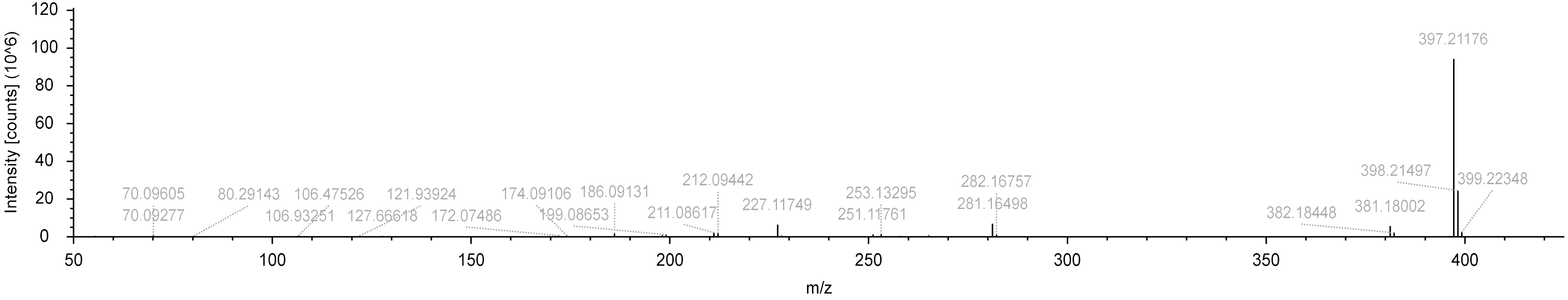

© Reported with Compound Discoverer 3.2

Compounds

Unit Laboratorium Riset Unggulan IPB

Structure	Name	RT [min]	Formula	Molecular Weight	Group Areas
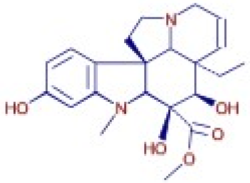	Methyl (2beta,3beta,4beta,5alpha,12beta, 19alpha)-3,4,16-trihydroxy-1-methyl-6,7-didehydroaspidospermidine-3-carboxylate	9.90	C22 H28 N2 O5	400.1998	1.50e10

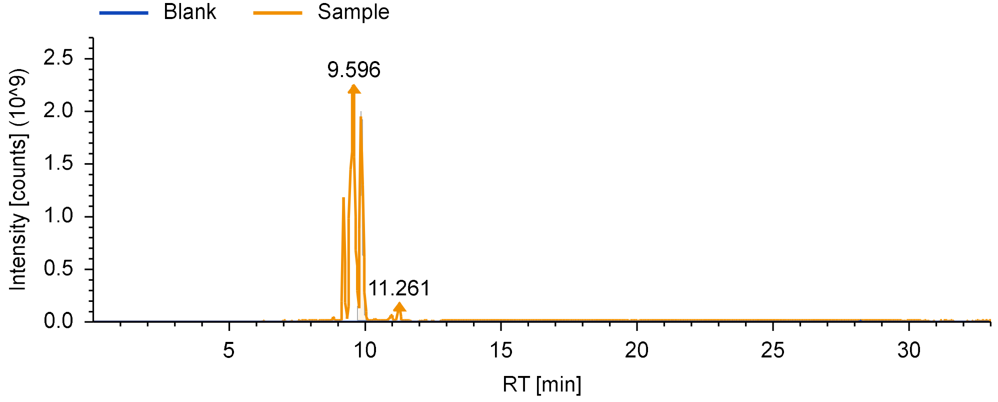

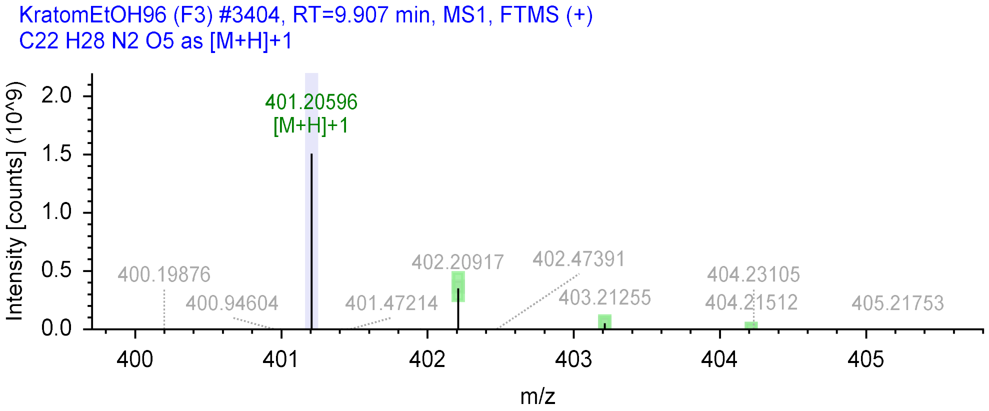

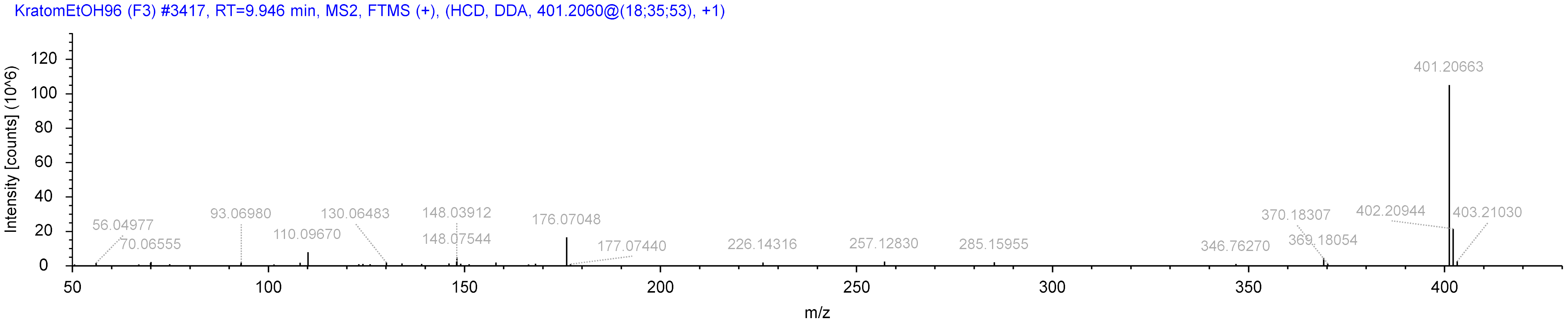

© Reported with Compound Discoverer 3.2

Compounds

Unit Laboratorium Riset Unggulan IPB

Structure	Name	RT [min]	Formula	Molecular Weight	Group Areas
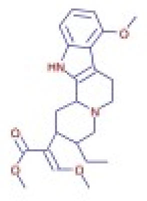	Mitragynine	12.39	C23 H30 N2 O4	398.2206	1.37e10

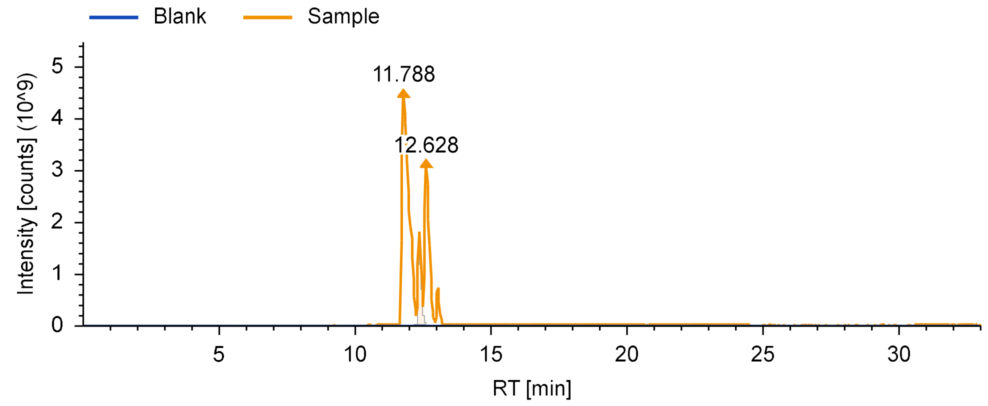

KratomEtOH96 (F3) #4256, RT=12.383 min, MS1, FTMS (+)
C23 H30 N2 O4 as [M+H]+1

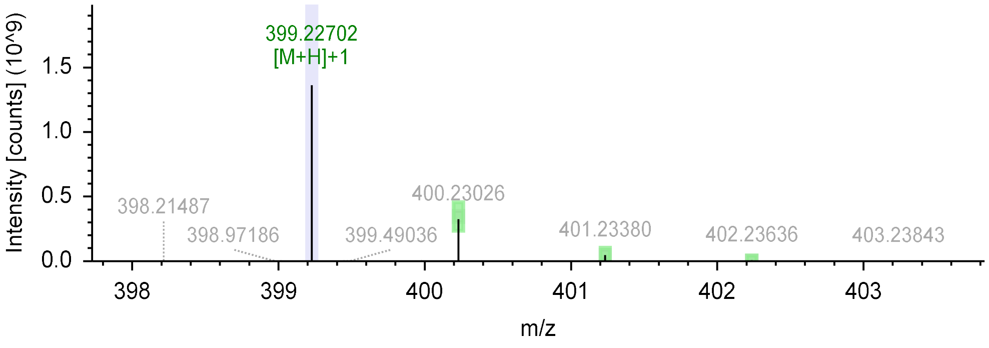

KratomEtOH96 (F3) #4269, RT=12.422 min, MS2, FTMS (+), (HCD, DDA, 399.2270@(18;35;53), +1)

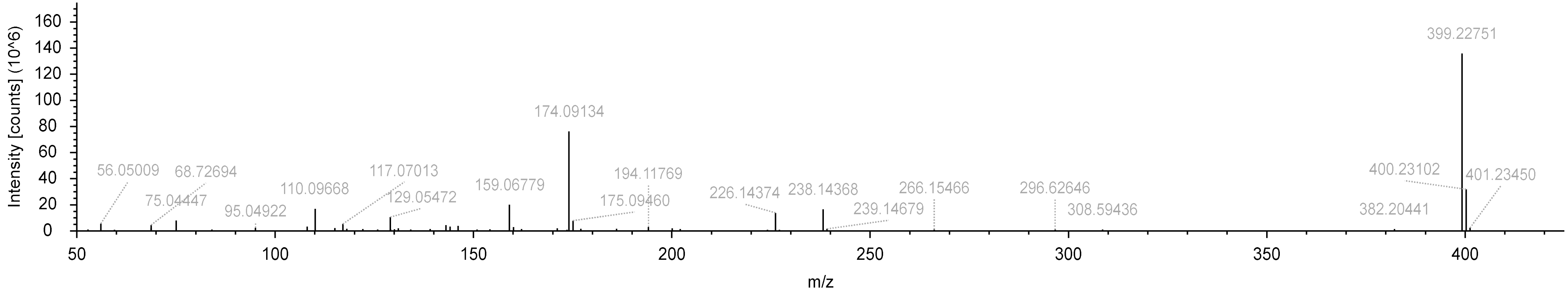

© Reported with Compound Discoverer 3.2

Structure	Name	RT [min]	Formula	Molecular Weight	Group Areas
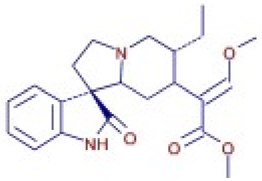	Rhynchophylline	10.71	C22 H28 N2 O4	384.2049	1.01e10

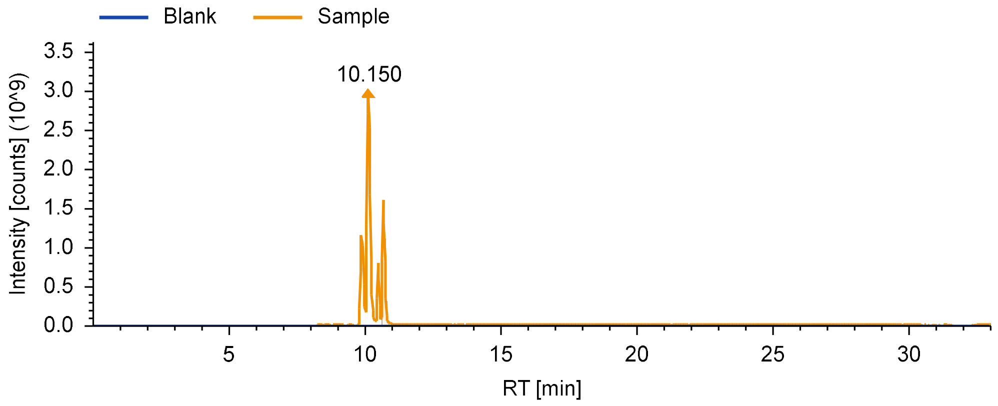

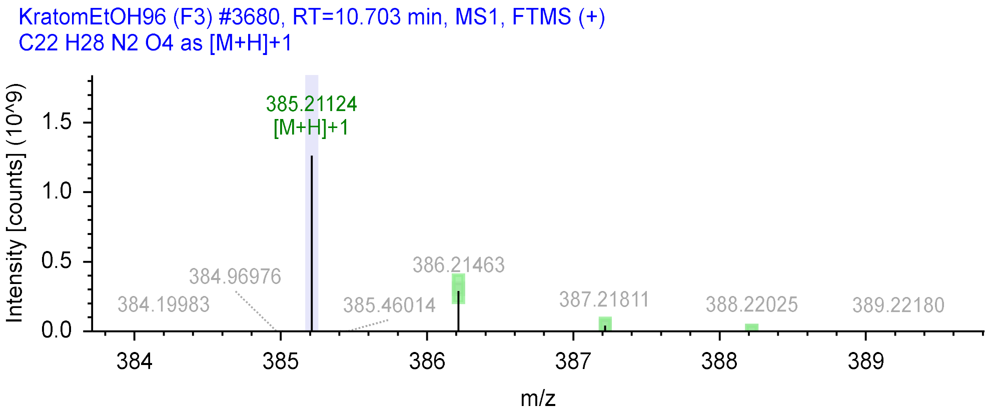

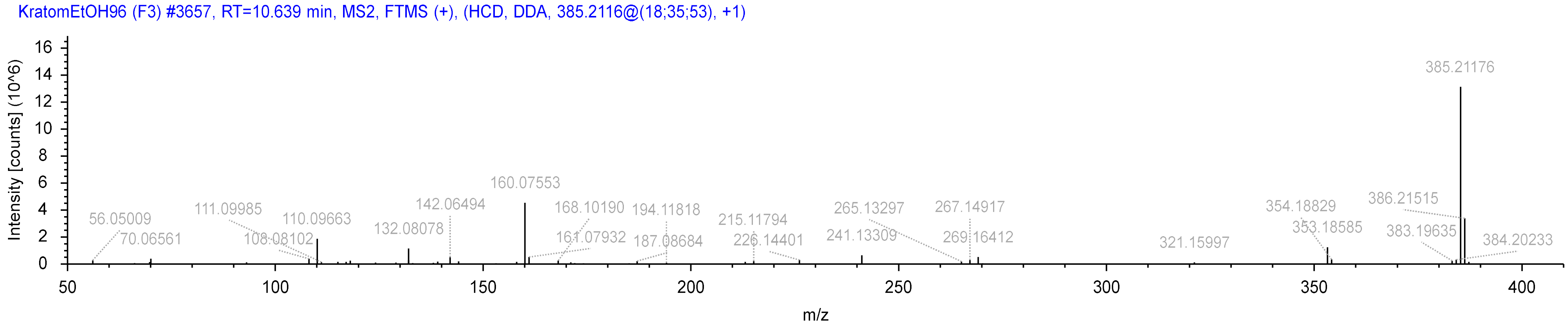

© Reported with Compound Discoverer 3.2

Structure	Name	RT [min]	Formula	Molecular Weight	Group Areas
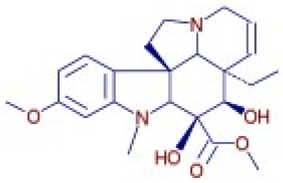	deacetylvindoline	11.19	C23 H30 N2 O5	414.2155	7.95e9

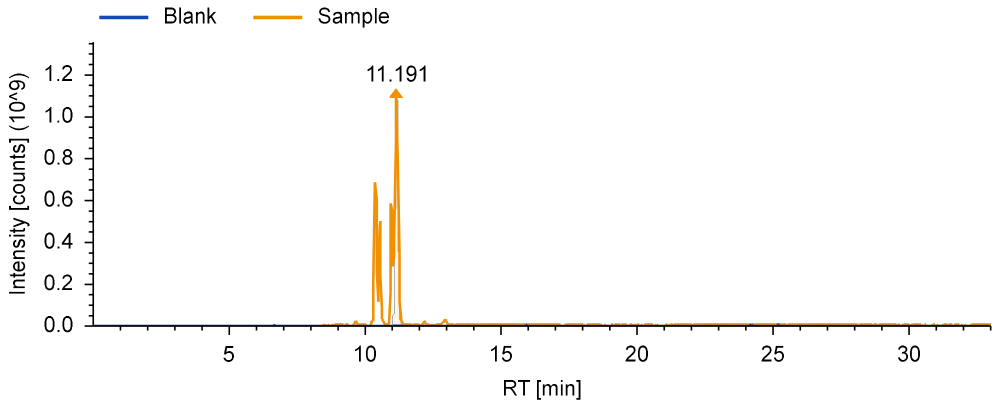

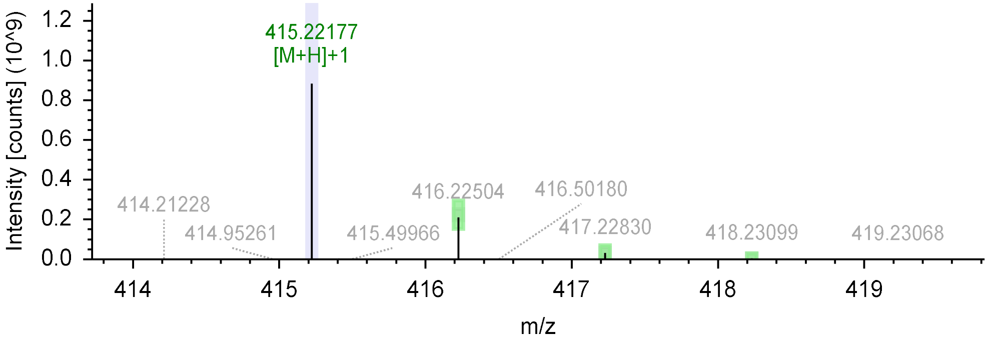

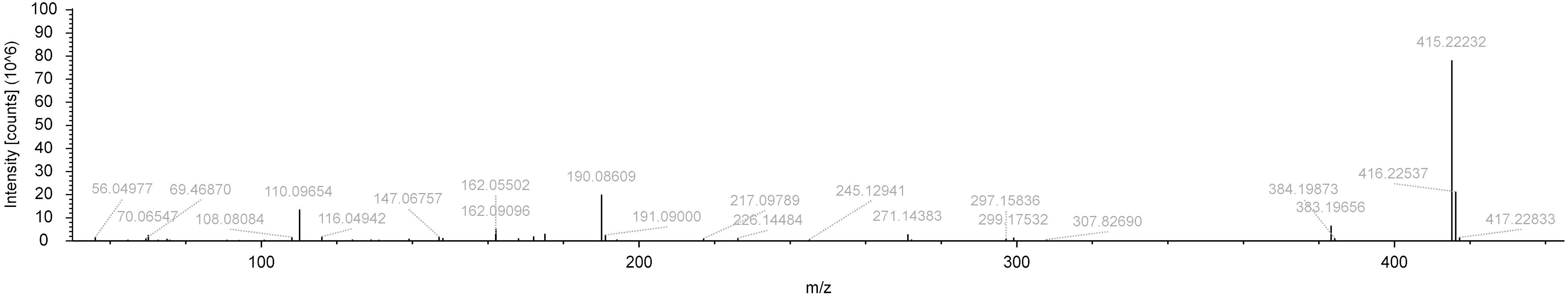

© Reported with Compound Discoverer 3.2

Structure	Name	RT [min]	Formula	Molecular Weight	Group Areas
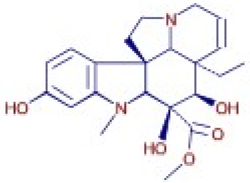	Methyl (2beta,3beta,4beta,5alpha,12beta,19alpha)-3,4,16-trihydroxy-1-methyl-6,7-didehydroaspidospermidine-3-carboxylate	9.24	C22 H28 N2 O5	400.1998	6.55e9

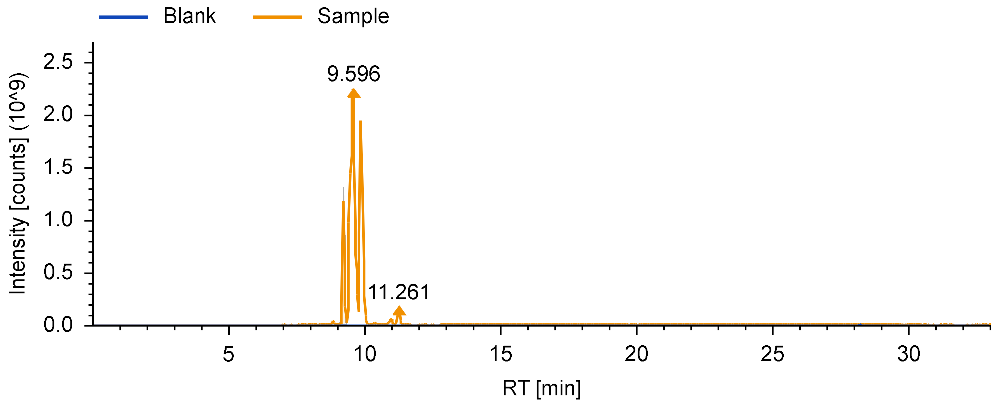

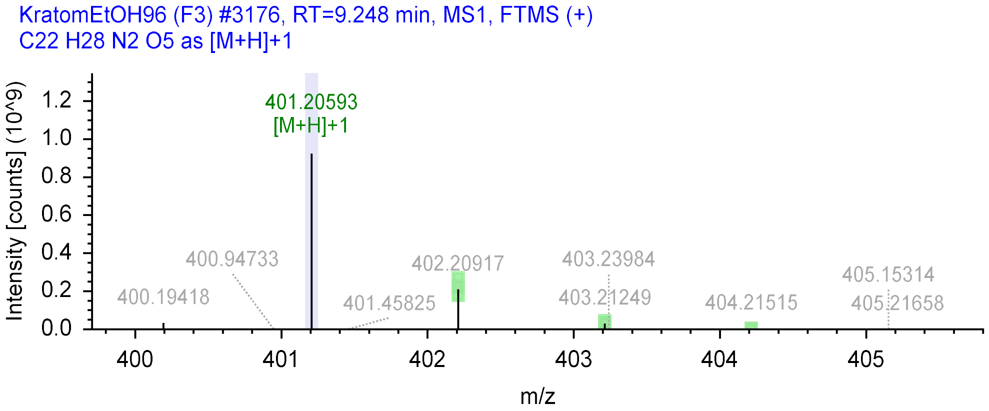

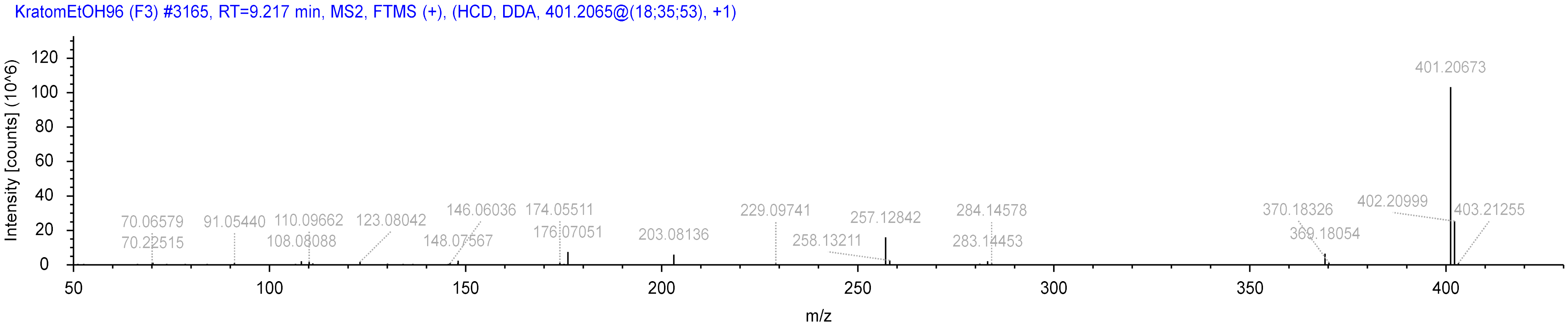

© Reported with Compound Discoverer 3.2

Structure	Name	RT [min]	Formula	Molecular Weight	Group Areas
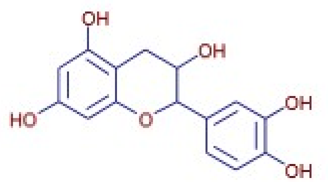	Cianidanol	7.24	C15 H14 O6	290.0790	5.51e9

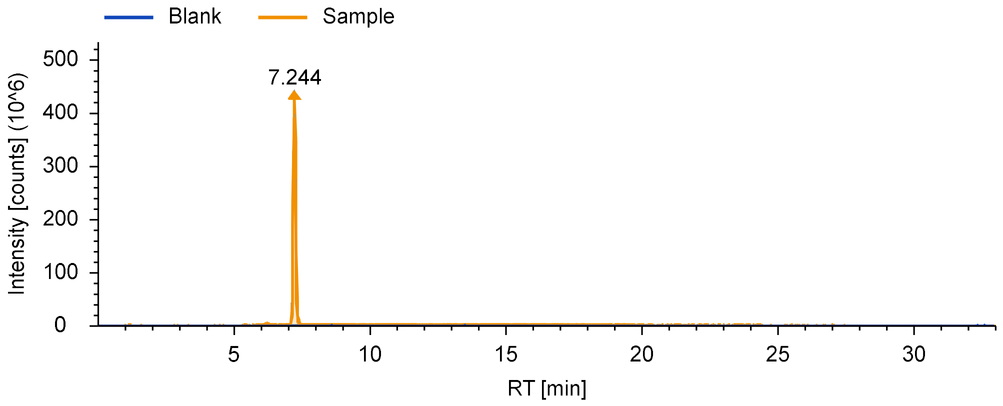

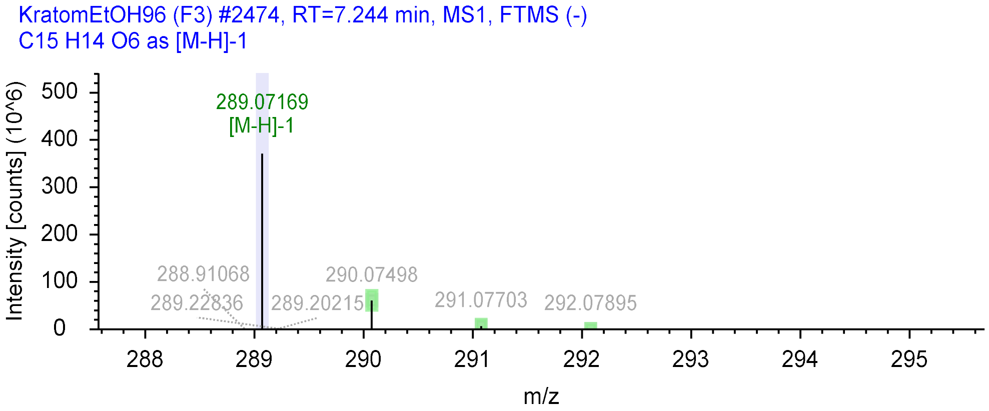

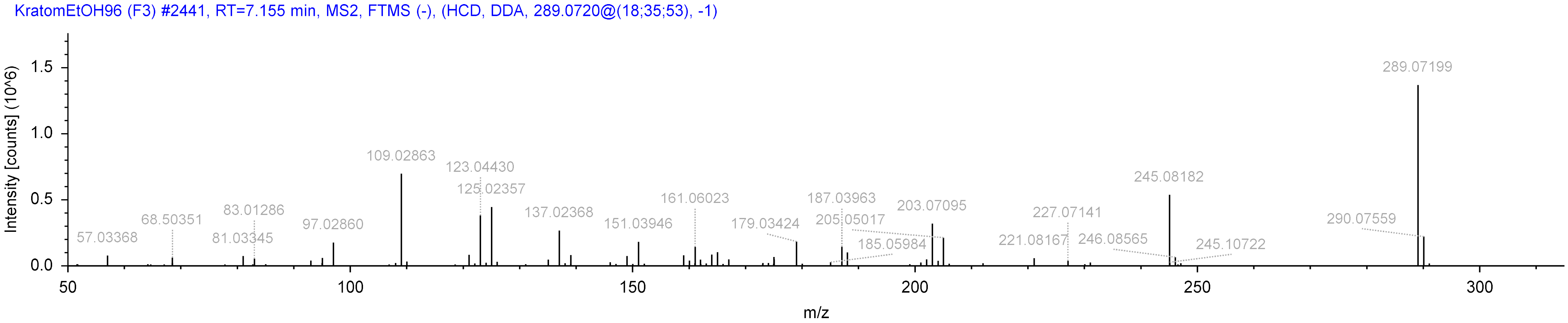

© Reported with Compound Discoverer 3.2

Compounds

Unit Laboratorium Riset Unggulan IPB

Structure	Name	RT [min]	Formula	Molecular Weight	Group Areas
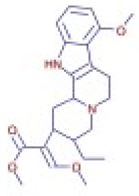	Mitragynine	13.07	C23 H30 N2 O4	398.2206	4.52e9

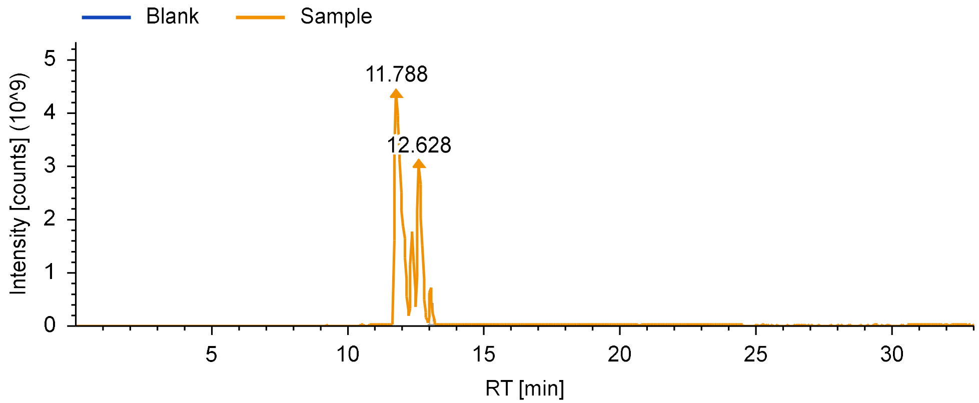

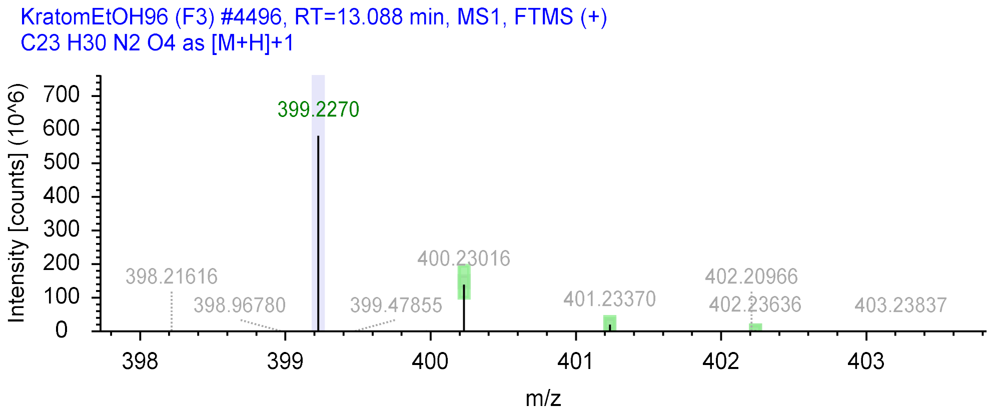

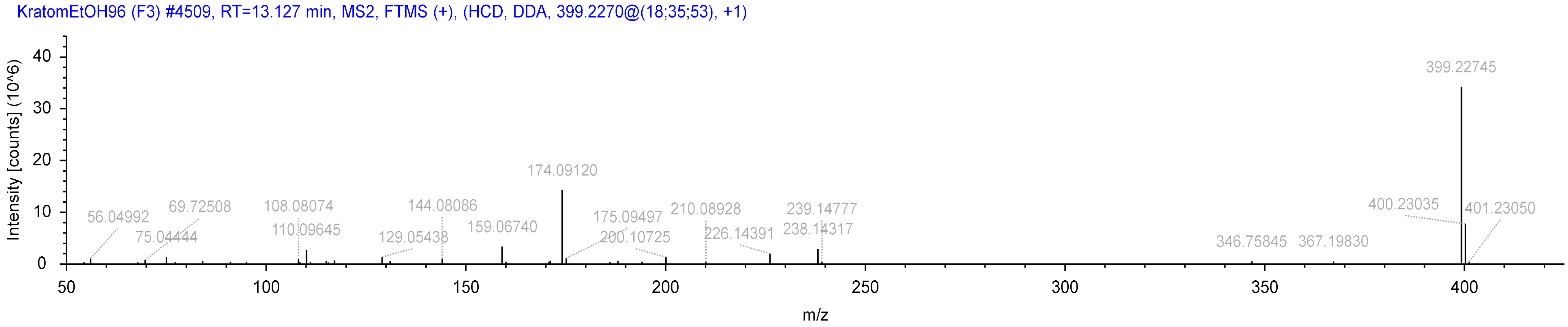

© Reported with Compound Discoverer 3.2

Compounds

Unit Laboratorium Riset Unggulan IPB

Structure	Name	RT [min]	Formula	Molecular Weight	Group Areas
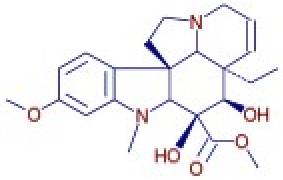	deacetylvindoline	11.00	C23 H30 N2 O5	414.2155	3.63e9

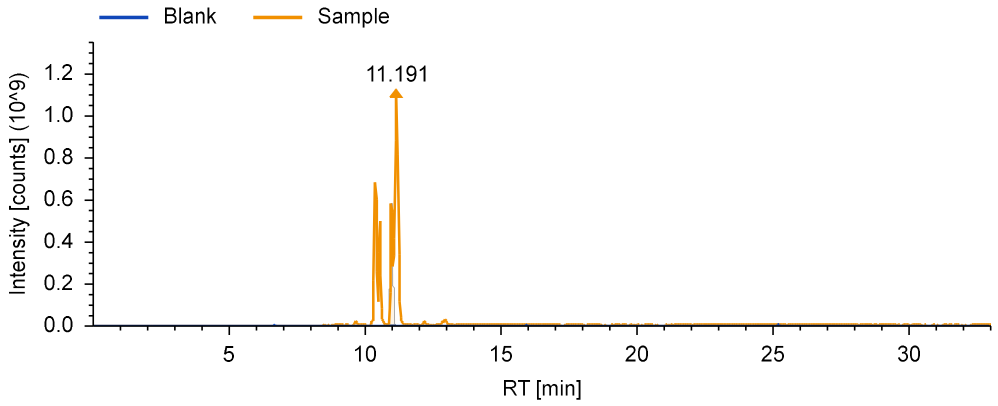

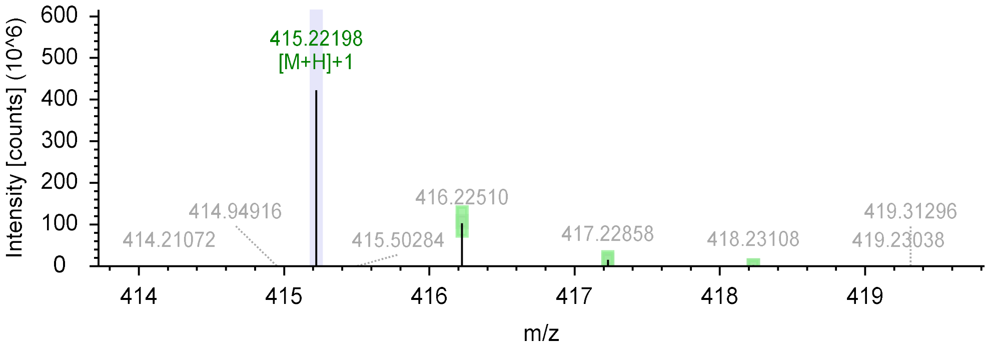

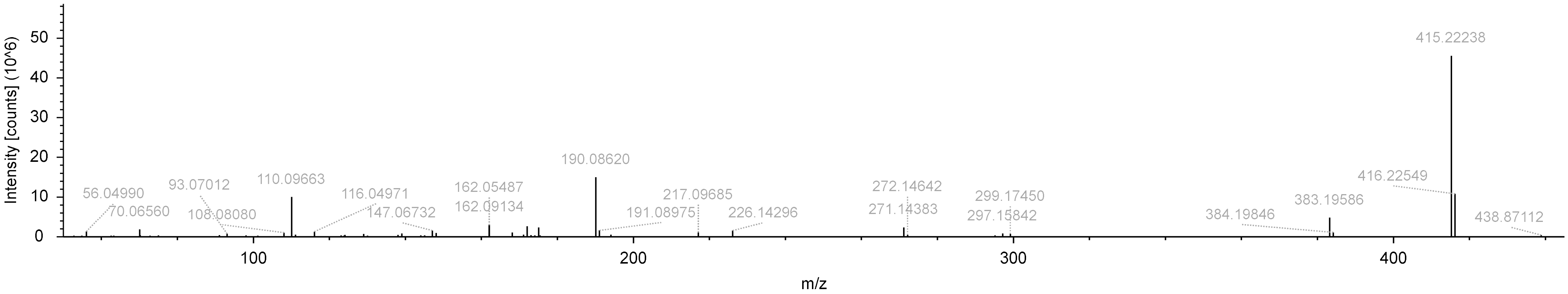

© Reported with Compound Discoverer 3.2

Compounds

Unit Laboratorium Riset Unggulan IPB

Structure	Name	RT [min]	Formula	Molecular Weight	Group Areas
		9.31	C17 H23 N10 P	398.1845	3.35e9

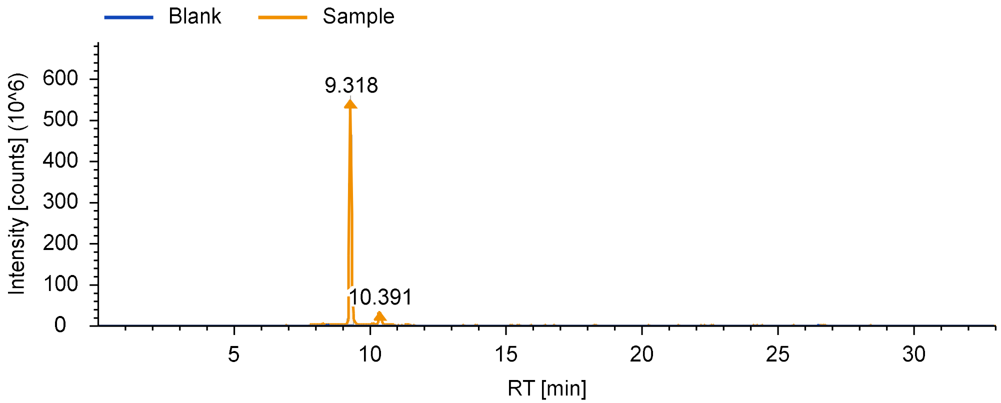

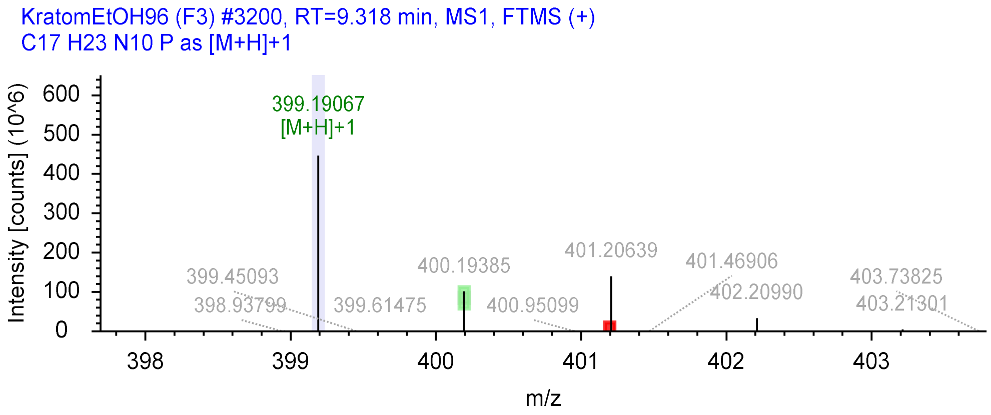

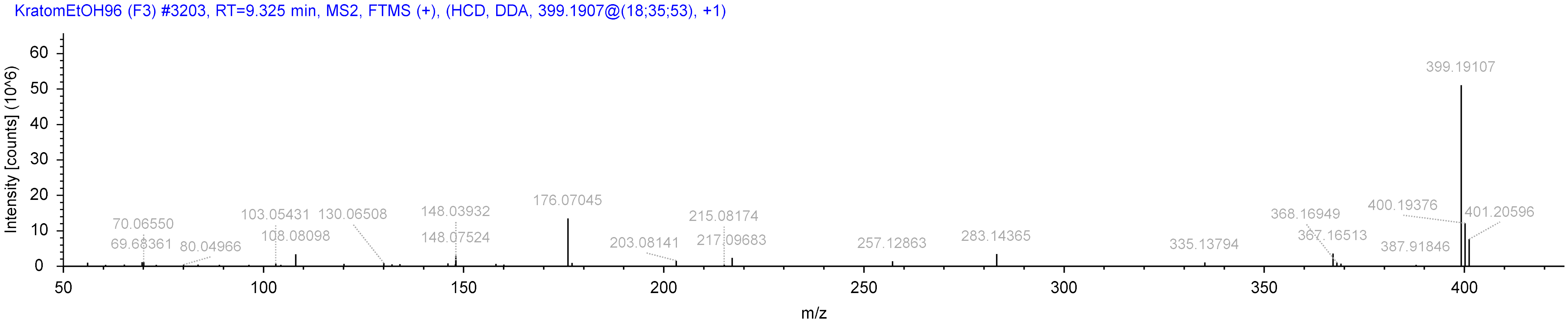

© Reported with Compound Discoverer 3.2

Structure	Name	RT [min]	Formula	Molecular Weight	Group Areas
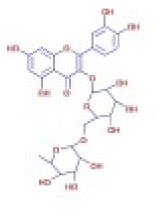	Rutin	8.48	C27 H30 O16	610.1534	2.99e9

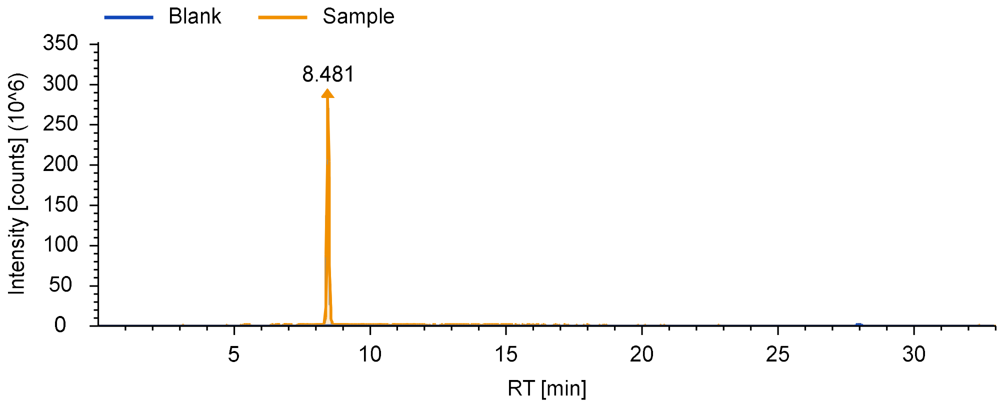

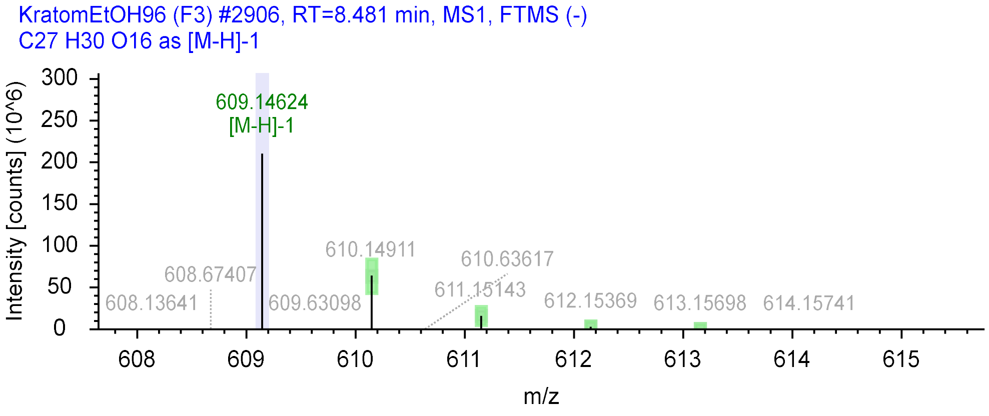

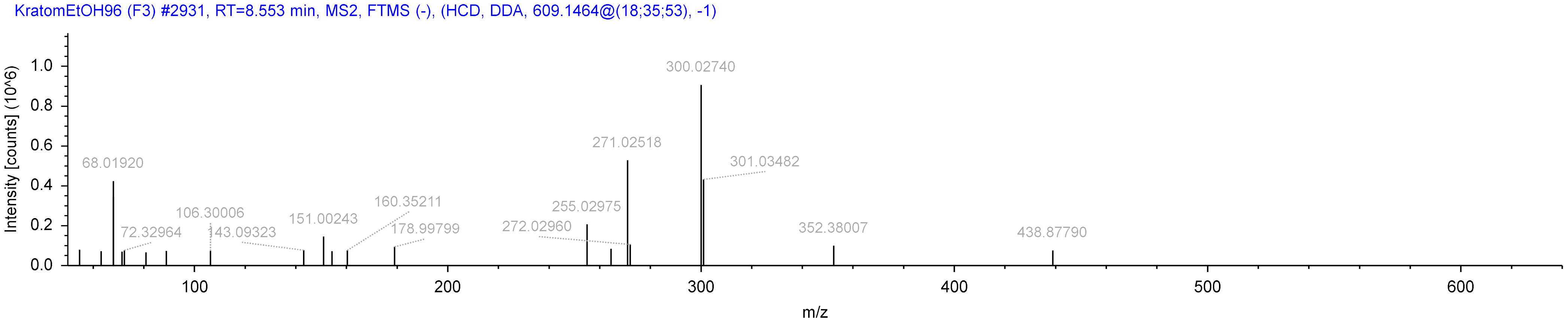

© Reported with Compound Discoverer 3.2

Structure	Name	RT [min]	Formula	Molecular Weight	Group Areas
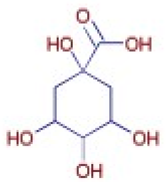	1,3,4,5-Tetrahydroxycyclohexanecarboxylic acid	1.13	C7 H12 O6	192.0634	2.72e9

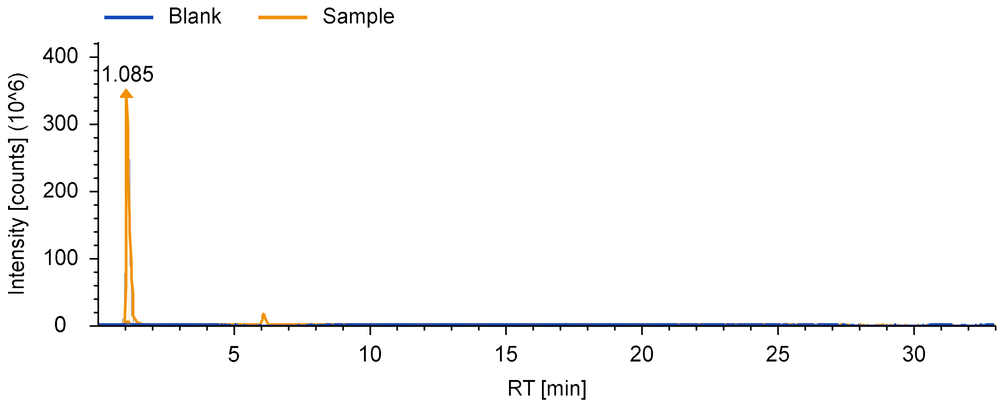

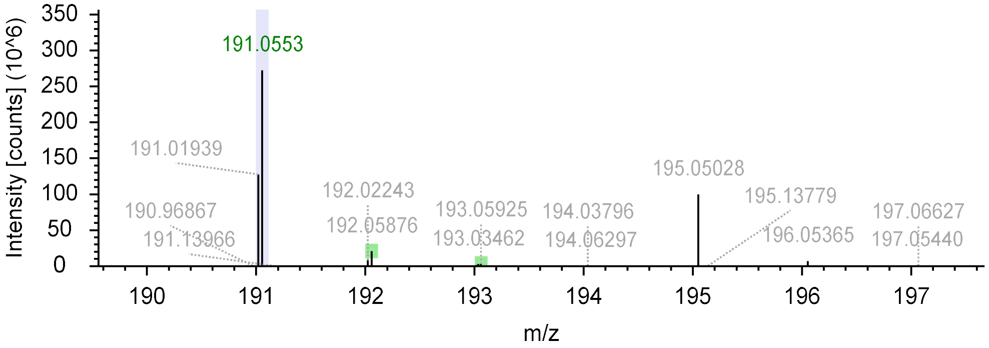

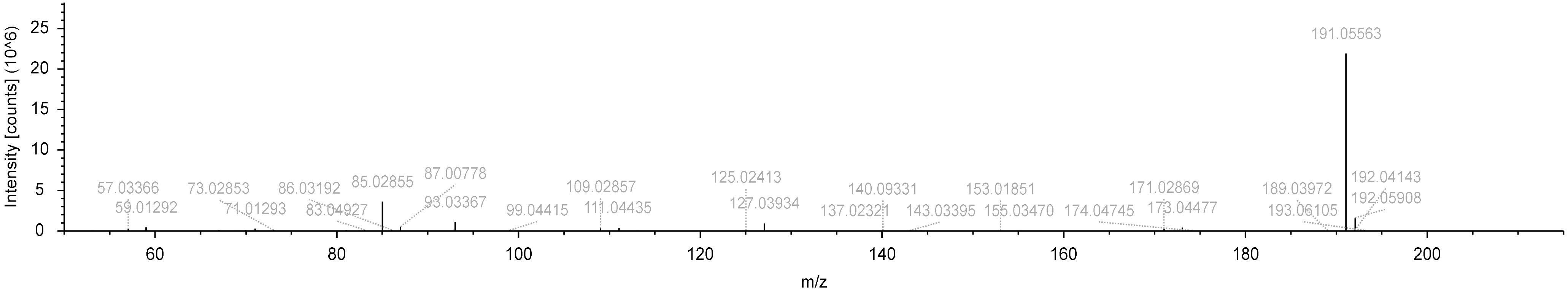

© Reported with Compound Discoverer 3.2

Compounds

Unit Laboratorium Riset Unggulan IPB

Structure	Name	RT [min]	Formula	Molecular Weight	Group Areas
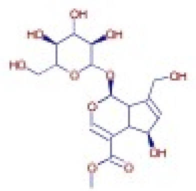	6beta-hydroxygeniposide	7.67	C17 H24 O11	404.1319	2.49e9

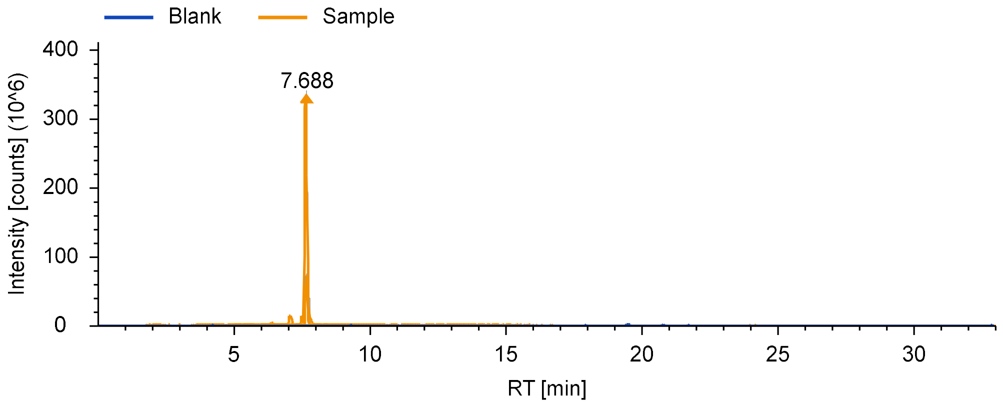

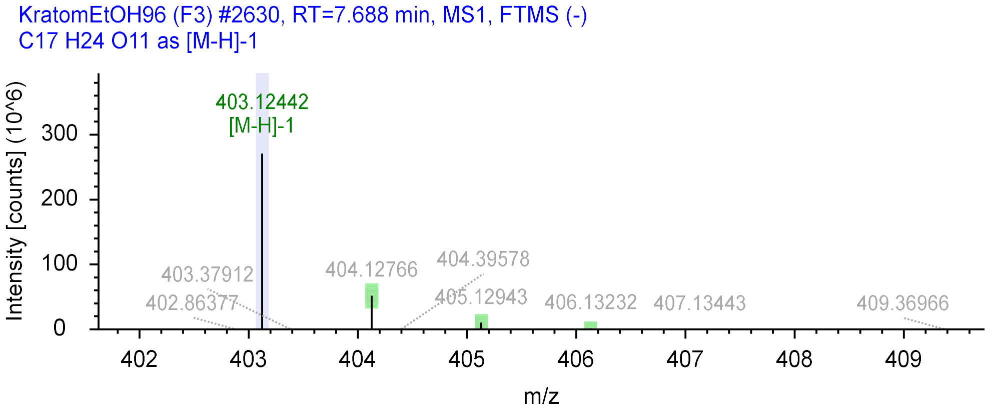

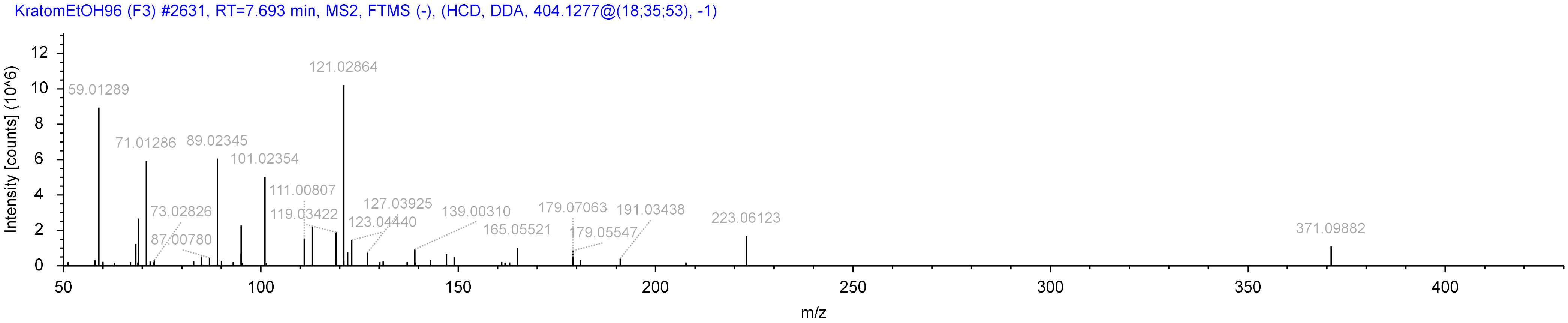

© Reported with Compound Discoverer 3.2

Structure	Name	RT [min]	Formula	Molecular Weight	Group Areas
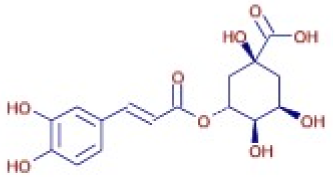	Chlorogenic acid	6.14	C16 H18 O9	354.0951	2.30e9

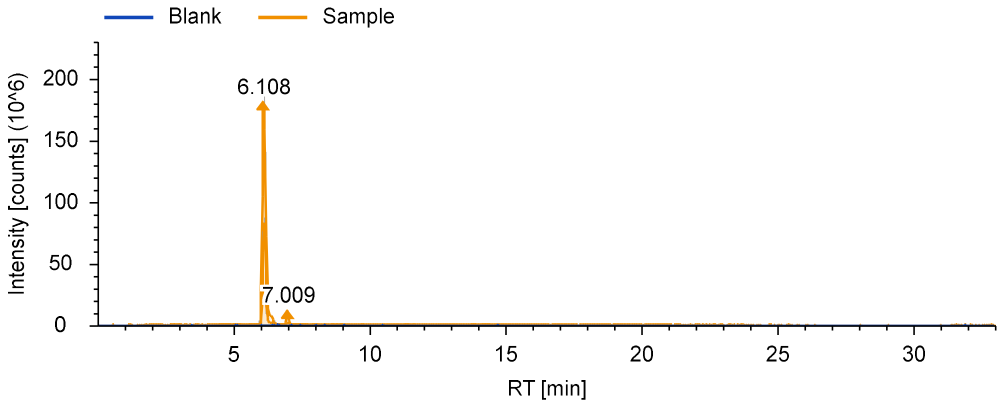

KratomEtOH96 (F3) #2090, RT=6.141 min, MS1, FTMS (-)
C16 H18 O9 as [M-H]-1

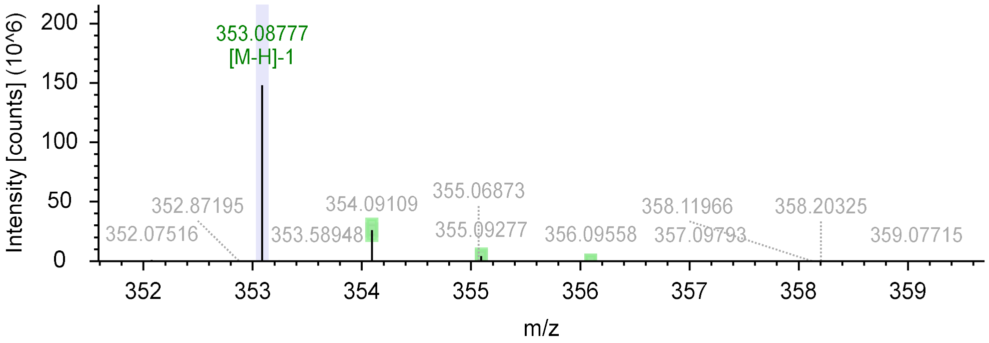

KratomEtOH96 (F3) #2115, RT=6.213 min, MS2, FTMS (-), (HCD, DDA, 353.0877@(18;35;53), -1)

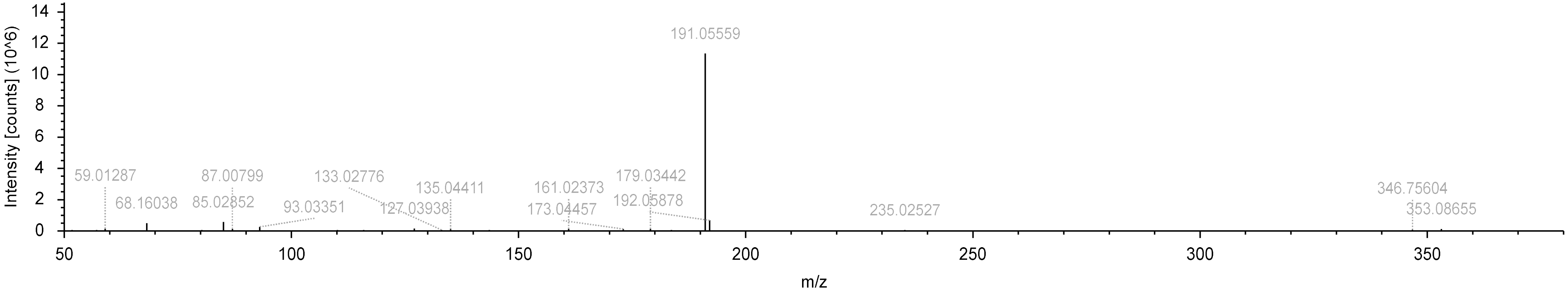

© Reported with Compound Discoverer 3.2

Structure	Name	RT [min]	Formula	Molecular Weight	Group Areas
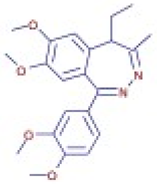	Tofisopam	10.03	C22 H26 N2 O4	382.1893	2.27e9

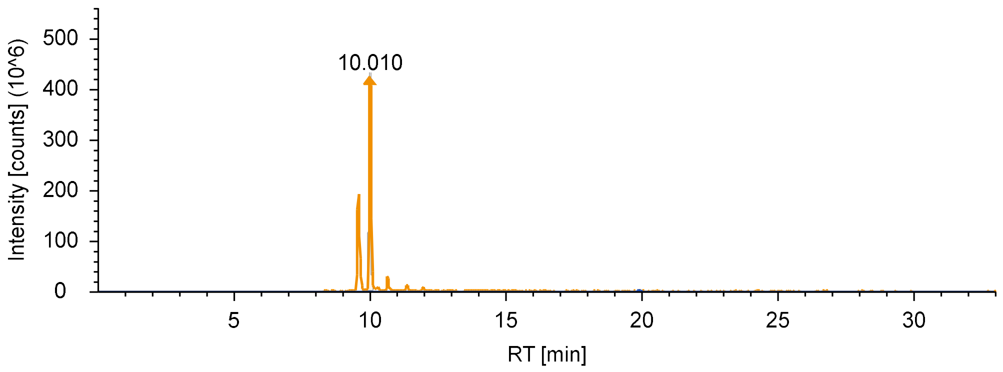

KratomEtOH96 (F3) #3452, RT=10.044 min, MS1, FTMS (+)
C22 H26 N2 O4 as [M+H]+1

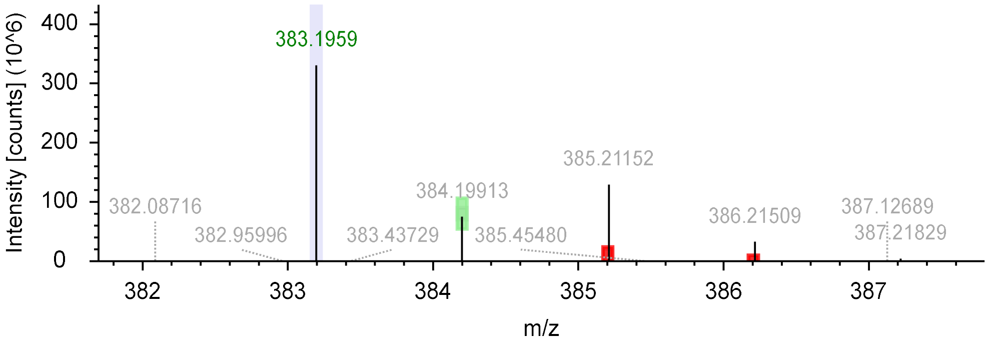

KratomEtOH96 (F3) #3443, RT=10.017 min, MS2, FTMS (+), (HCD, DDA, 383.1961@(18;35;53), +1)

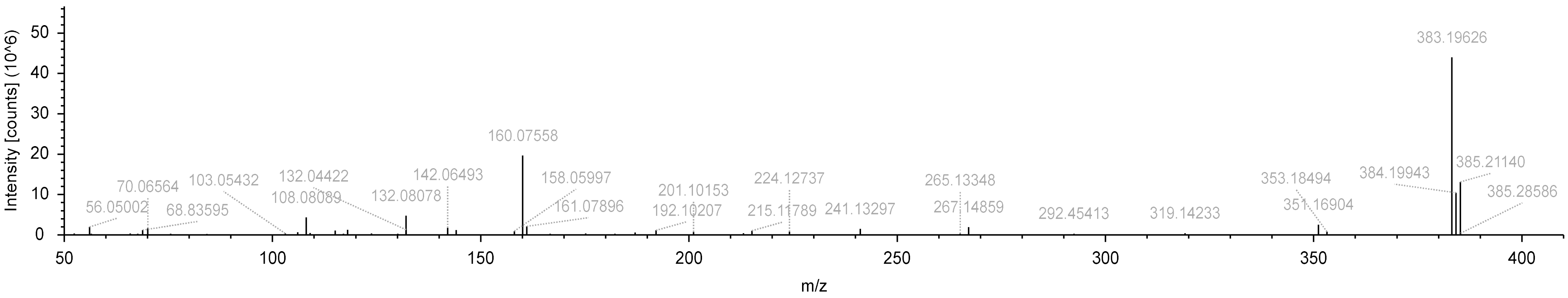

© Reported with Compound Discoverer 3.2

Structure	Name	RT [min]	Formula	Molecular Weight	Group Areas
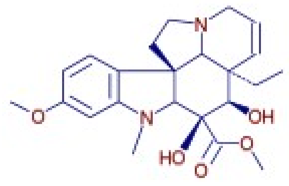	deacetylvindoline	10.56	C23 H30 N2 O5	414.2155	2.10e9

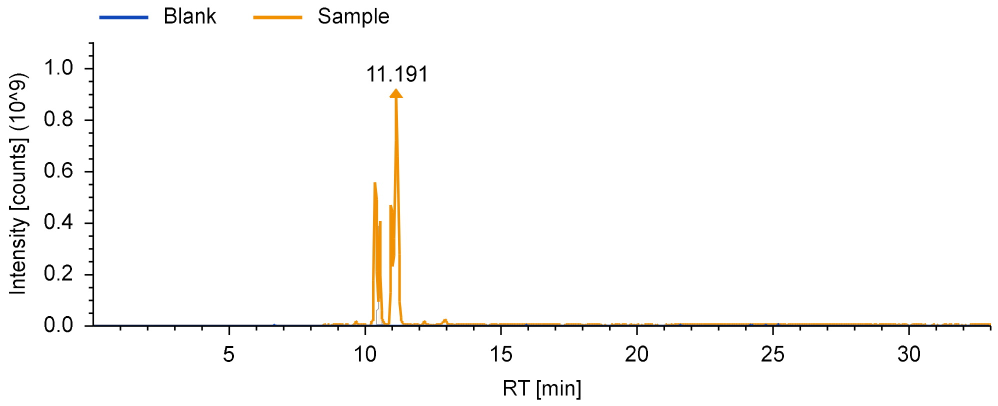

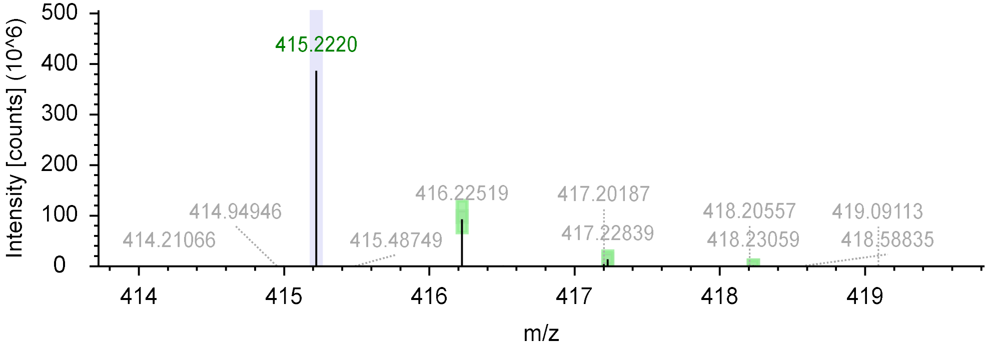

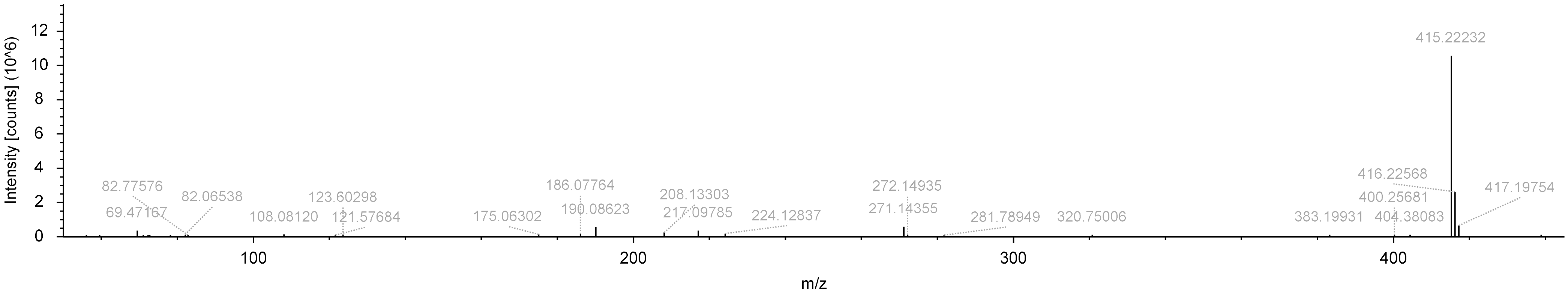

© Reported with Compound Discoverer 3.2

Structure	Name	RT [min]	Formula	Molecular Weight	Group Areas
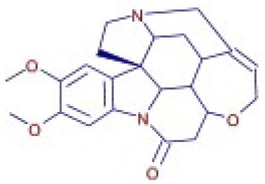	(-)-Brucine	13.43	C23 H26 N2 O4	394.1893	2.08e9

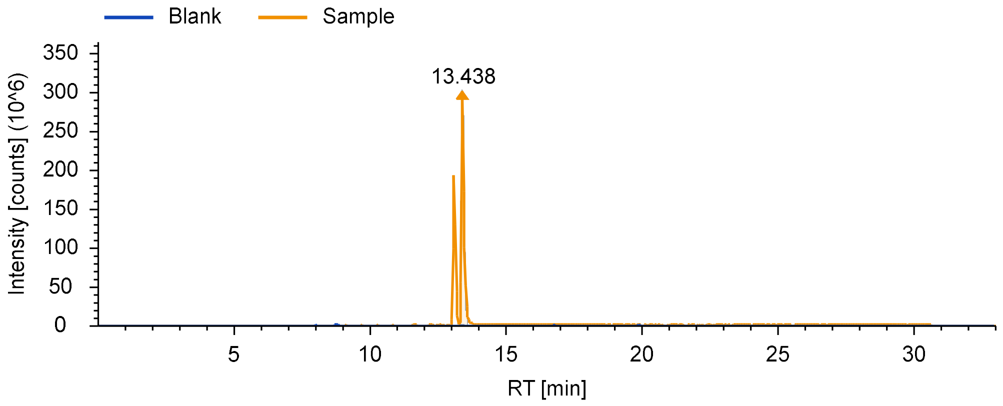

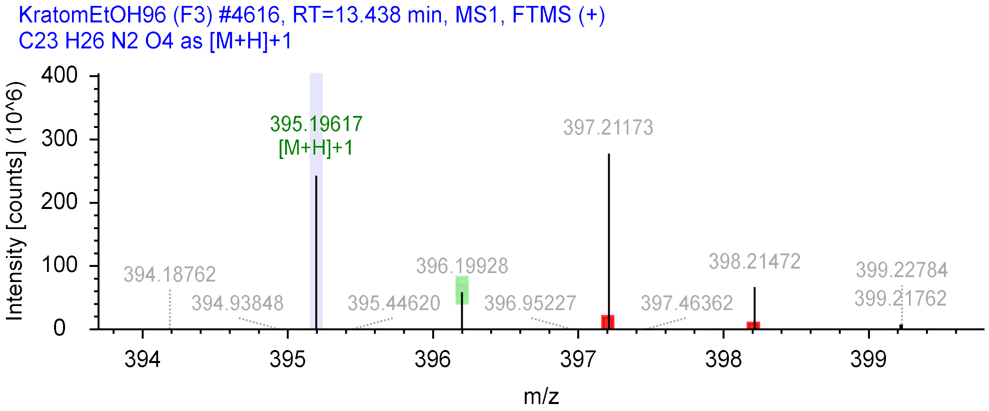

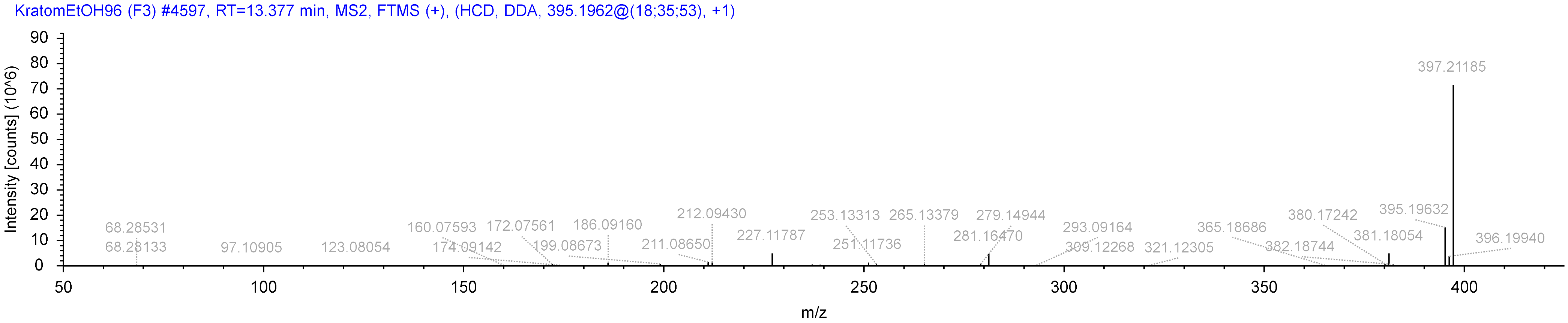

© Reported with Compound Discoverer 3.2

Compounds

Unit Laboratorium Riset Unggulan IPB

Structure	Name	RT [min]	Formula	Molecular Weight	Group Areas
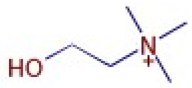	Choline	1.05	C5 H13 N O	103.0997	2.07e9

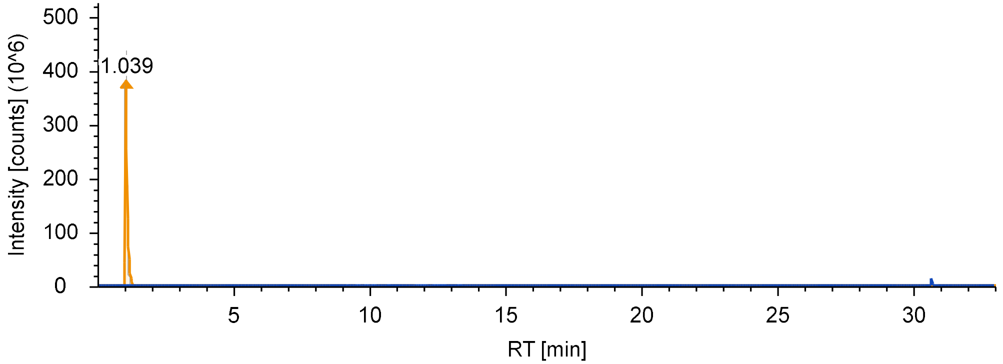

KratomEtOH96 (F3) #356, RT=1.039 min, MS1, FTMS (+)
C5 H13 N O as [M+H]+1

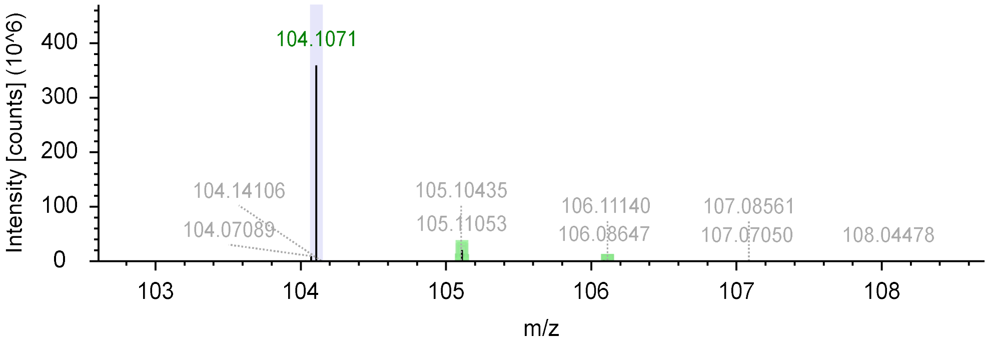

KratomEtOH96 (F3) #347, RT=1.014 min, MS2, FTMS (+), (HCD, DDA, 104.1072@(18;35;53), +1)

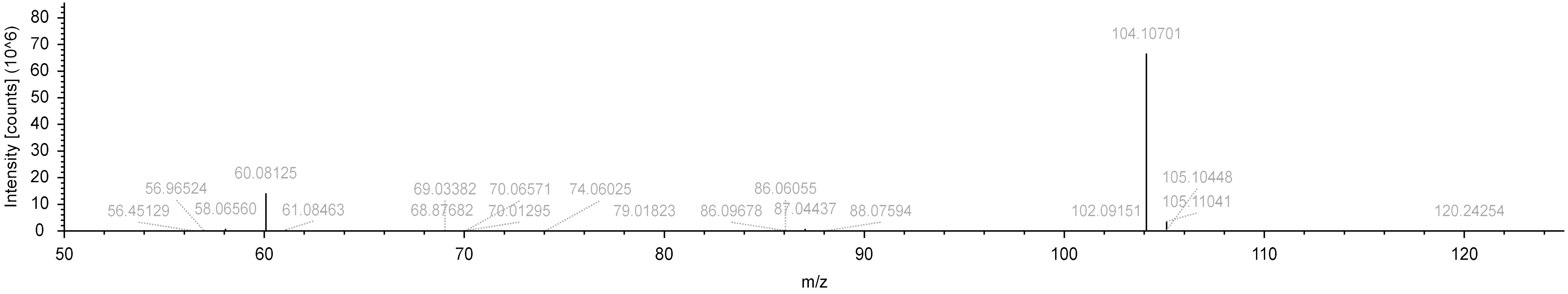

© Reported with Compound Discoverer 3.2

Structure	Name	RT [min]	Formula	Molecular Weight	Group Areas
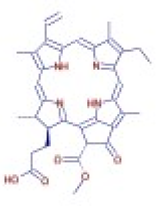	Pheophorbide A	26.63	C35 H36 N4 O5	592.2686	2.01e9

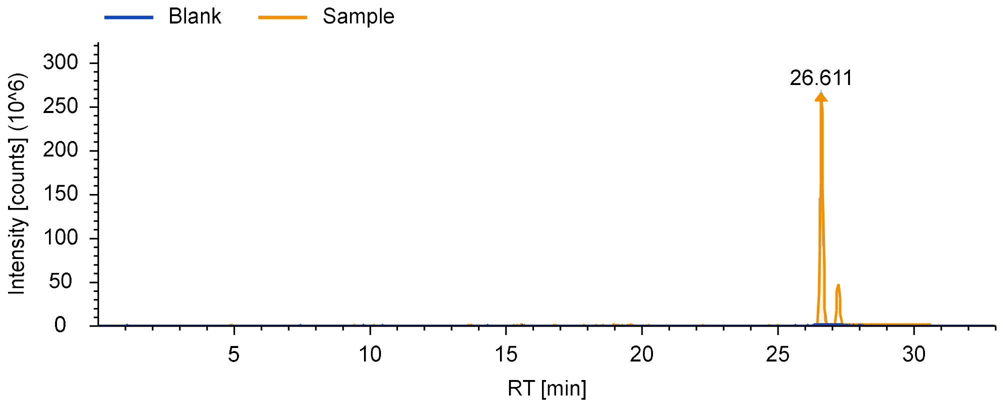

KratomEtOH96 (F3) #9092, RT=26.611 min, MS1, FTMS (+)
C35 H36 N4 O5 as [M+H]+1

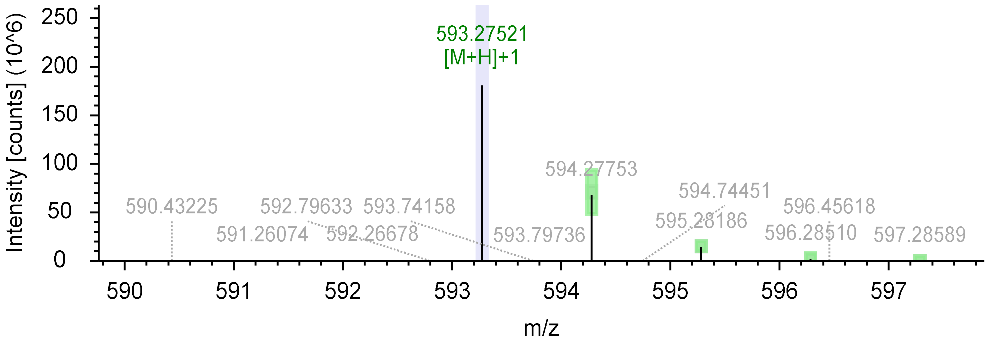

KratomEtOH96 (F3) #9129, RT=26.720 min, MS2, FTMS (+), (HCD, DDA, 593.2751@(18;35;53), +1)

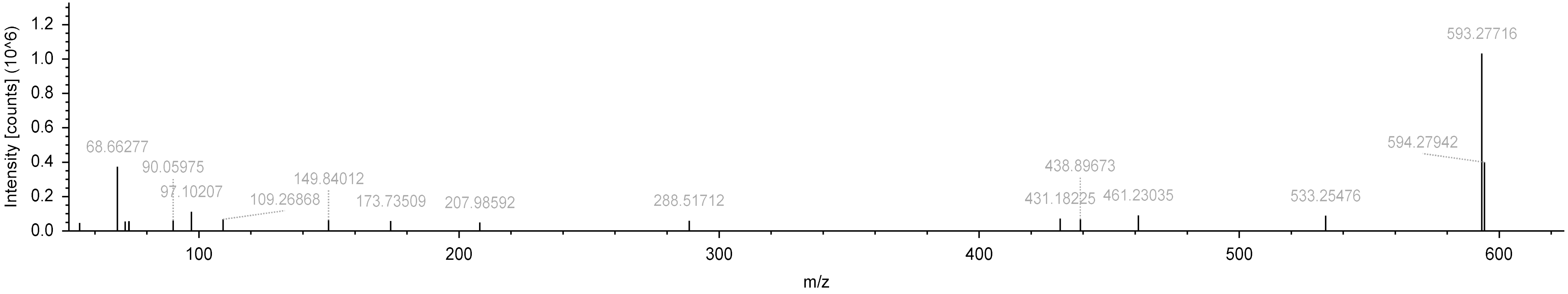

© Reported with Compound Discoverer 3.2

Compounds

Unit Laboratorium Riset Unggulan IPB

Structure	Name	RT [min]	Formula	Molecular Weight	Group Areas
		10.14	C23 H28 N2 O5	412.1998	1.91e9

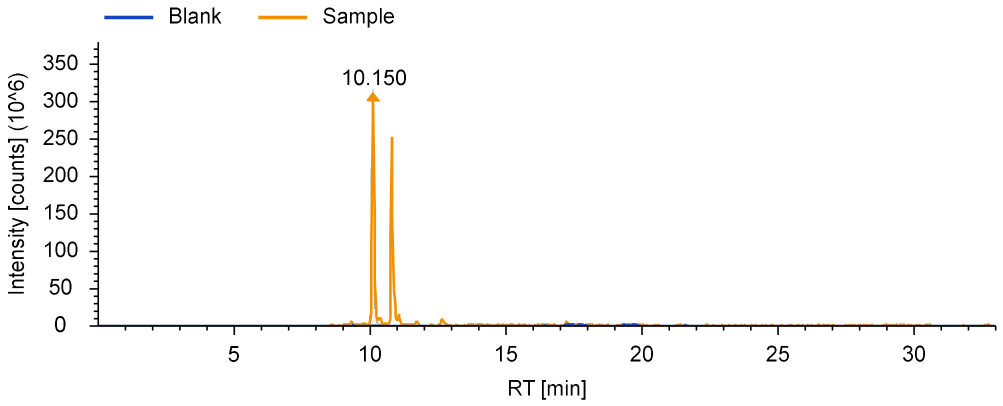

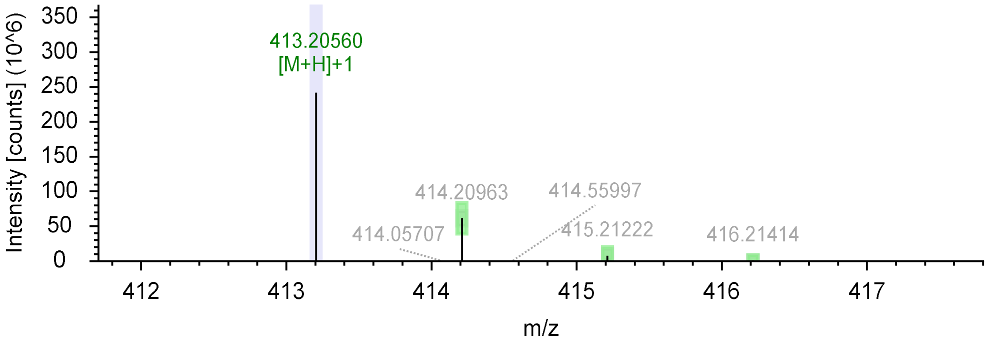

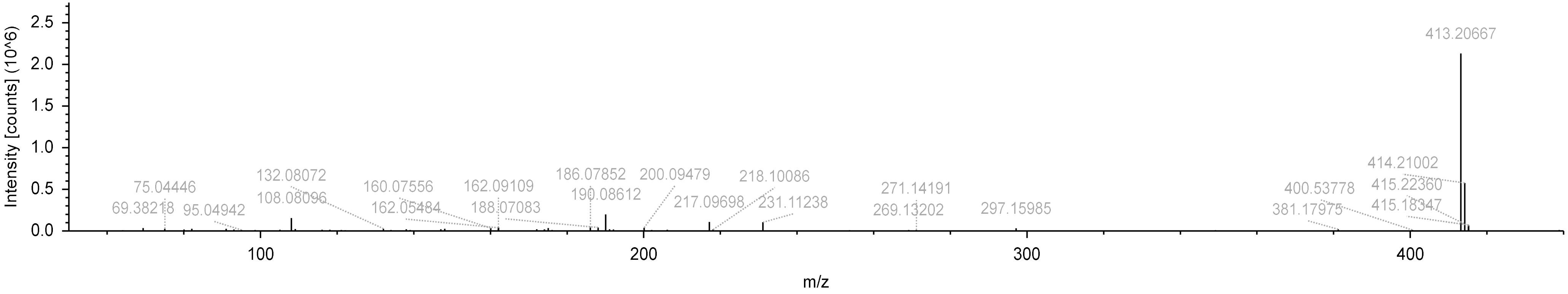

© Reported with Compound Discoverer 3.2

Structure	Name	RT [min]	Formula	Molecular Weight	Group Areas
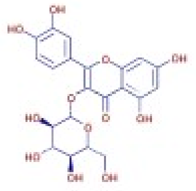	isoquercetin	8.79	C21 H20 O12	464.0955	1.89e9

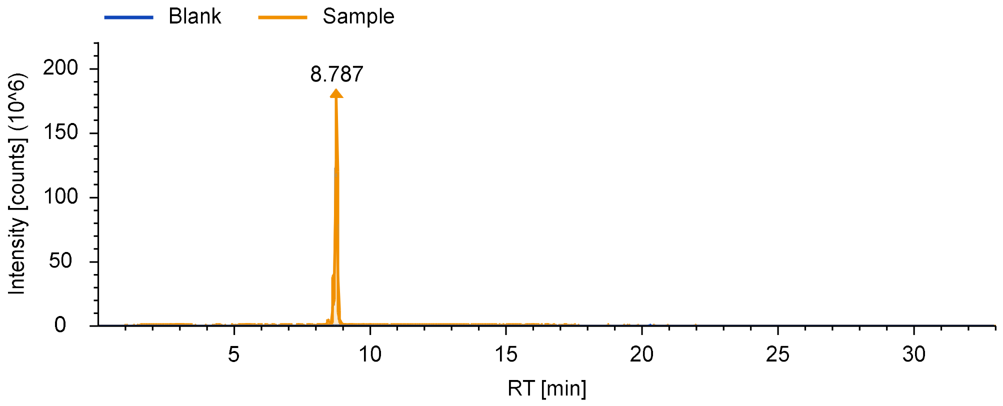

KratomEtOH96 (F3) #3014, RT=8.787 min, MS1, FTMS (-)
C21 H20 O12 as [M-H]-1

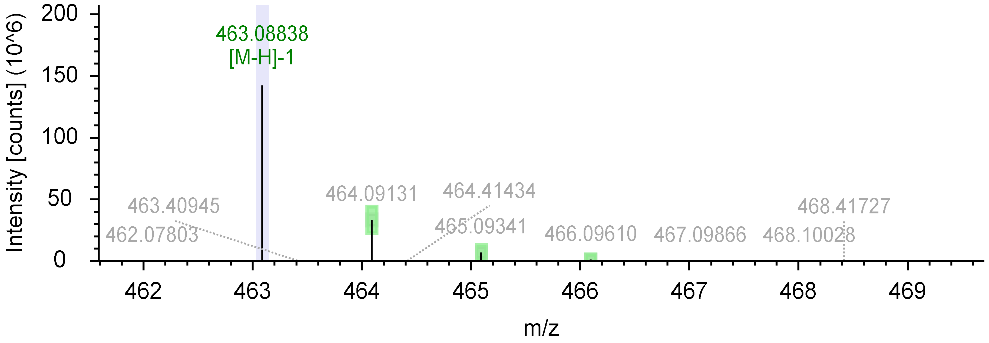

KratomEtOH96 (F3) #3027, RT=8.825 min, MS2, FTMS (-), (HCD, DDA, 463.0884@(18;35;53), -1)

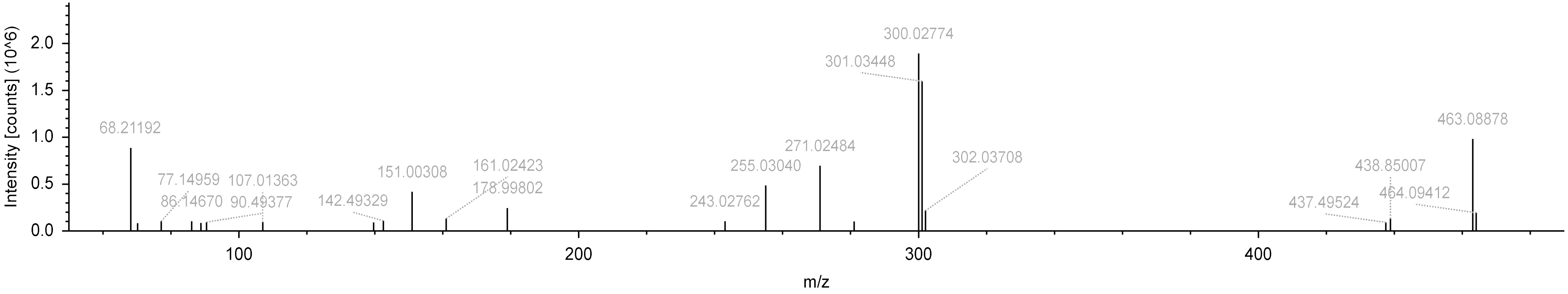

© Reported with Compound Discoverer 3.2

Compounds

Unit Laboratorium Riset Unggulan IPB

Structure	Name	RT [min]	Formula	Molecular Weight	Group Areas
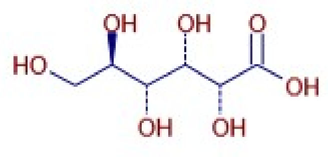	D-Gluconic acid	1.07	$C_6H_{12}O_7$	196.0583	1.88e9

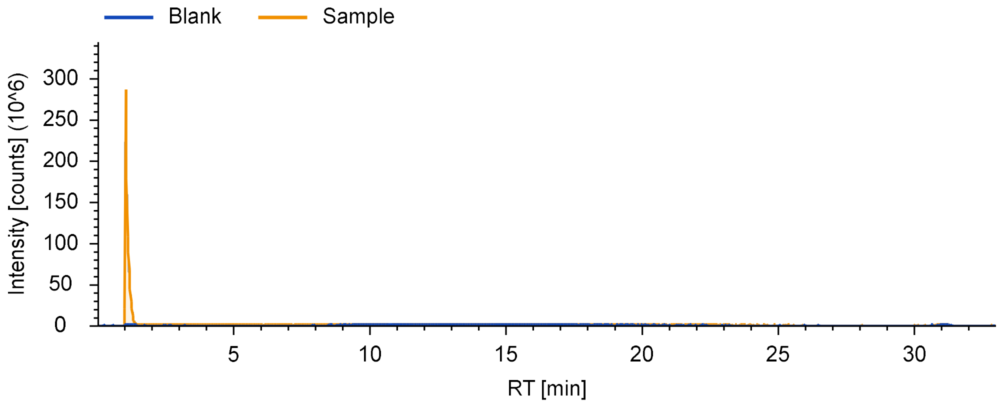

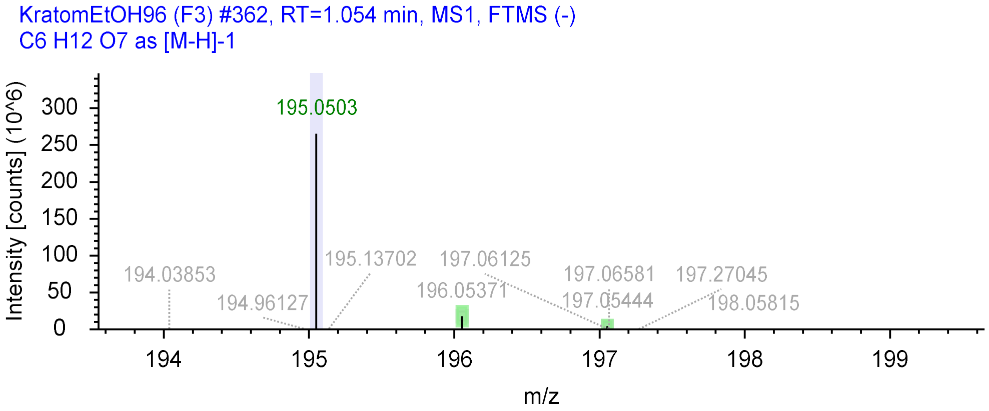

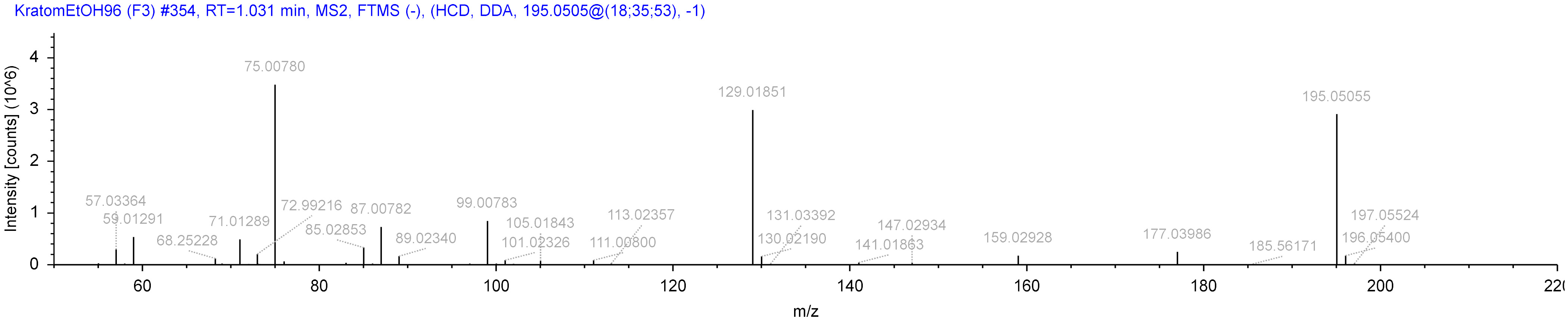

© Reported with Compound Discoverer 3.2

Structure	Name	RT [min]	Formula	Molecular Weight	Group Areas
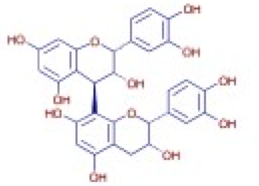	(+)-Procyanidin B2	6.83	C30 H26 O12	578.1424	1.81e9

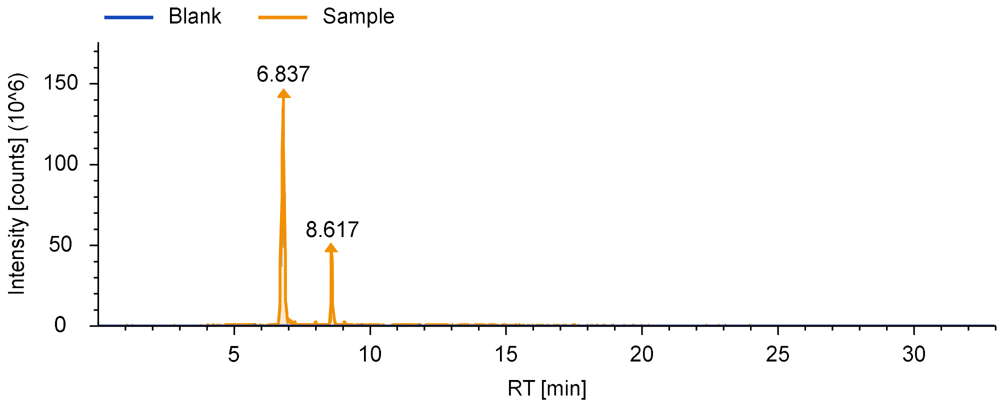

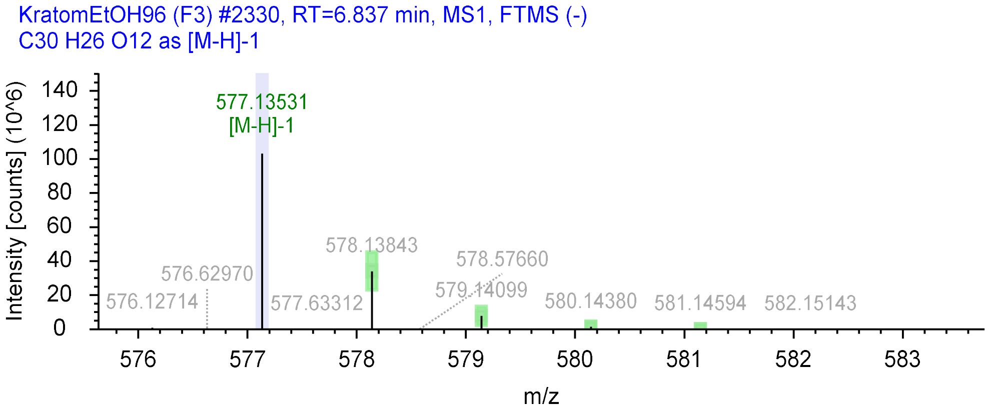

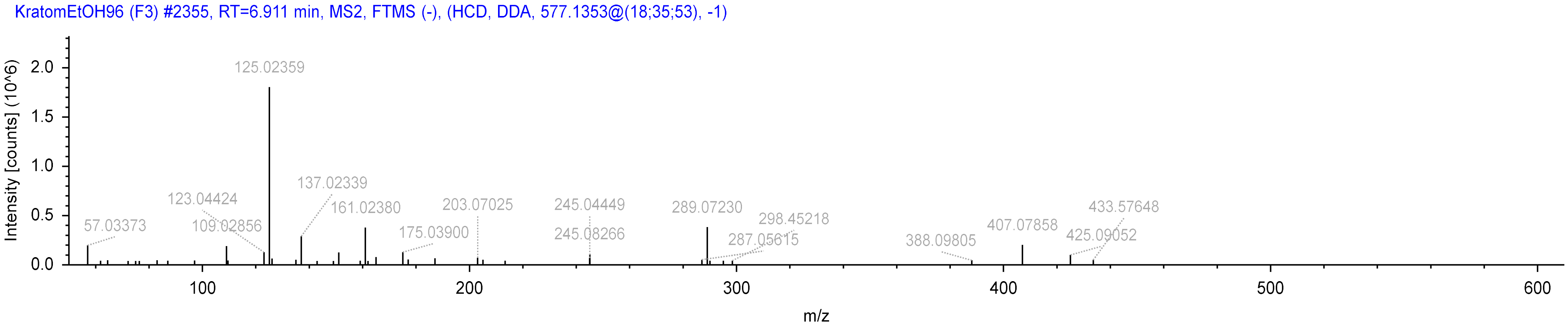

© Reported with Compound Discoverer 3.2

Compounds

Unit Laboratorium Riset Unggulan IPB

Structure	Name	RT [min]	Formula	Molecular Weight	Group Areas
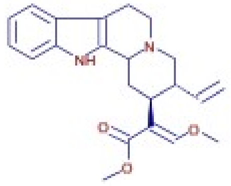	hirsuteine	12.45	C22 H26 N2 O3	366.1943	1.32e9

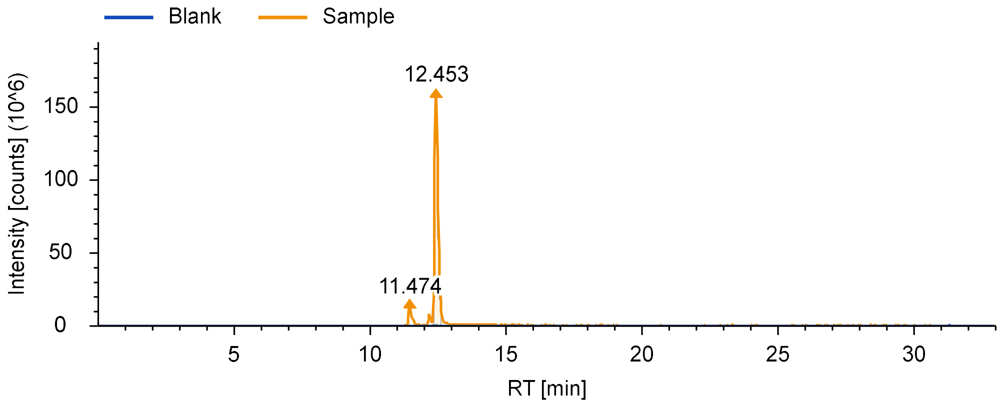

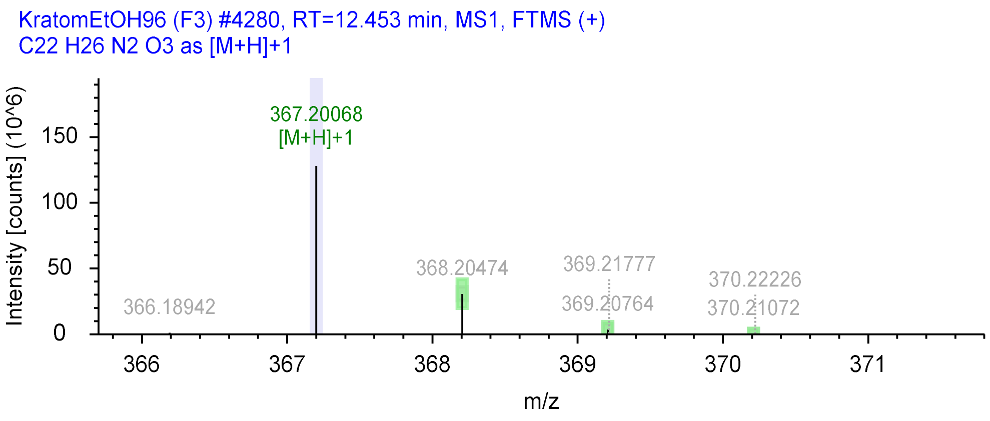

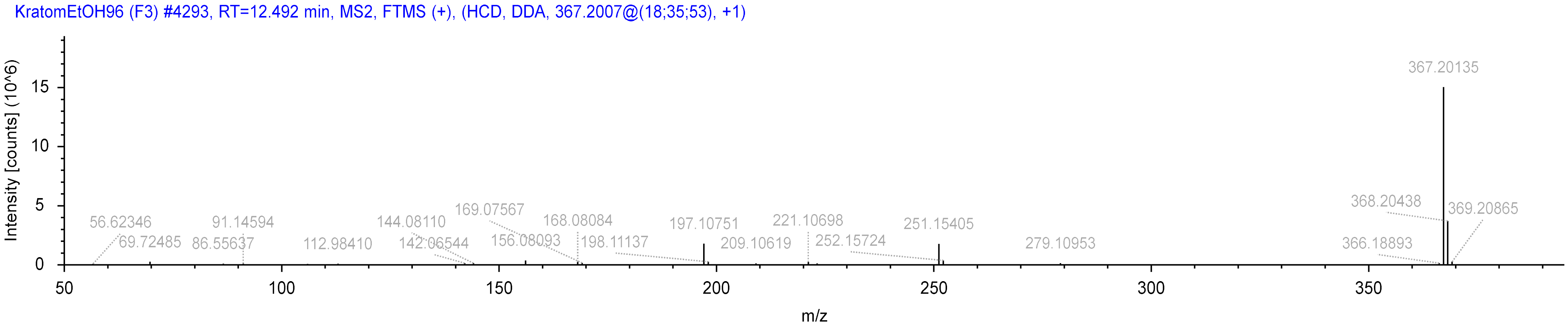

© Reported with Compound Discoverer 3.2

Structure	Name	RT [min]	Formula	Molecular Weight	Group Areas
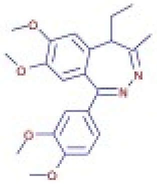	Tofisopam	9.59	C22 H26 N2 O4	382.1893	1.23e9

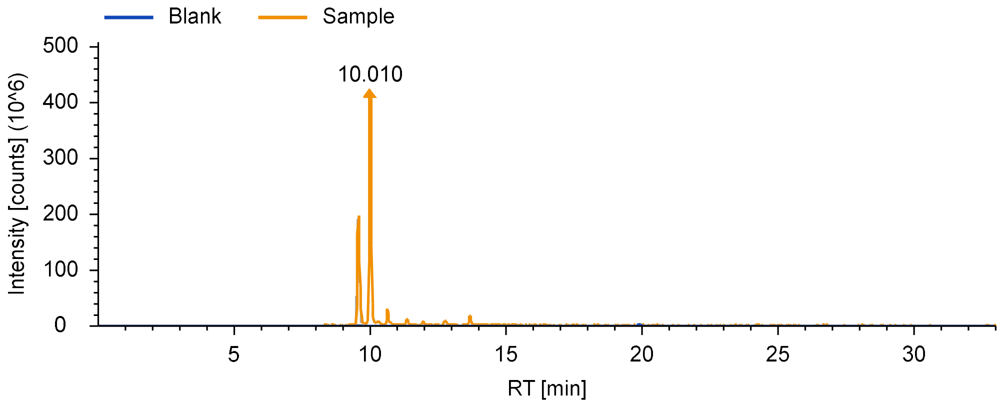

KratomEtOH96 (F3) #3296, RT=9.596 min, MS1, FTMS (+)
C22 H26 N2 O4 as [M+H]+1

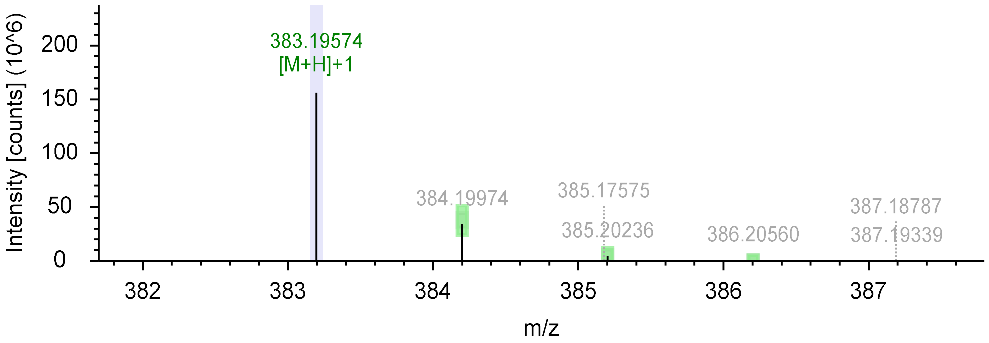

KratomEtOH96 (F3) #3275, RT=9.533 min, MS2, FTMS (+), (HCD, DDA, 383.1962@(18;35;53), +1)

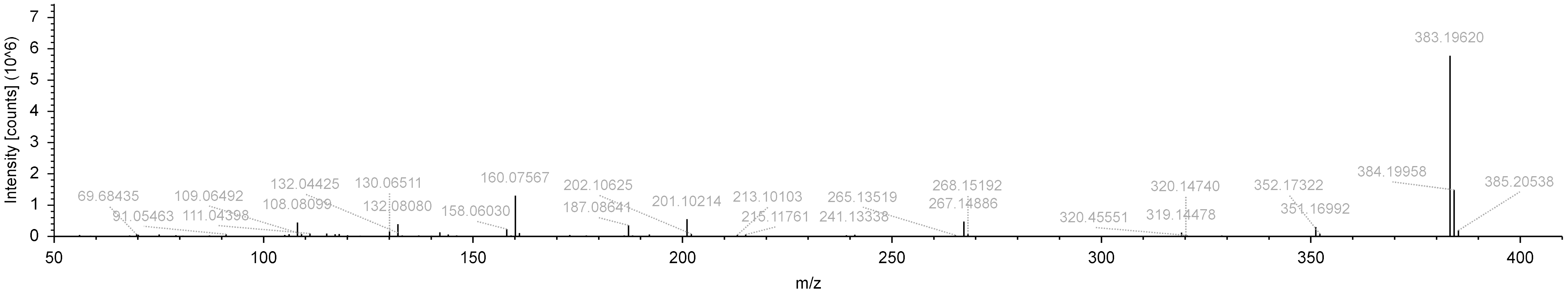

© Reported with Compound Discoverer 3.2

Structure	Name	RT [min]	Formula	Molecular Weight	Group Areas
		10.84	C23 H28 N2 O5	412.1998	1.22e9

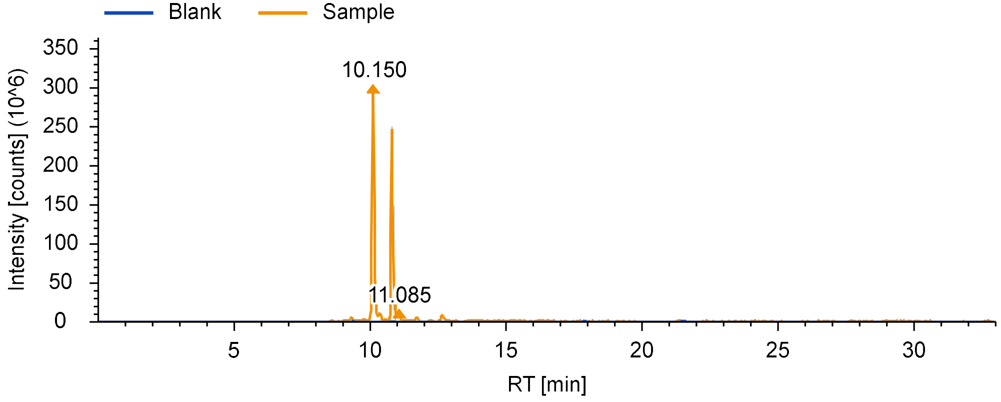

KratomEtOH96 (F3) #3728, RT=10.840 min, MS1, FTMS (+)
C23 H28 N2 O5 as [M+H]+1

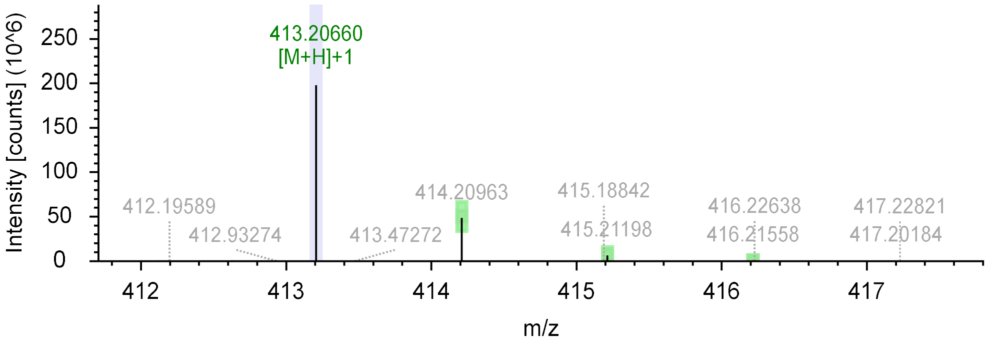

KratomEtOH96 (F3) #3707, RT=10.778 min, MS2, FTMS (+), (HCD, DDA, 413.2065@(18;35;53), +1)

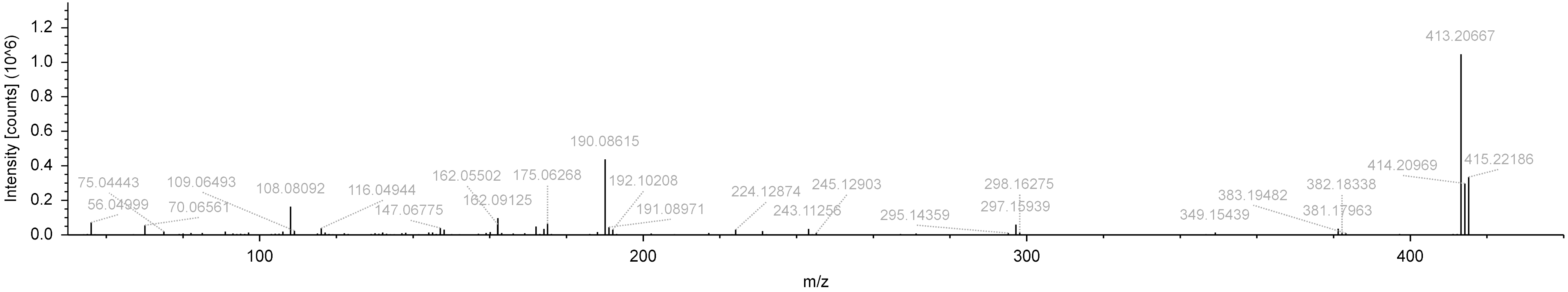

© Reported with Compound Discoverer 3.2

Structure	Name	RT [min]	Formula	Molecular Weight	Group Areas
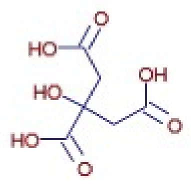	Citric acid	1.12	C6 H8 O7	192.0270	9.80e8

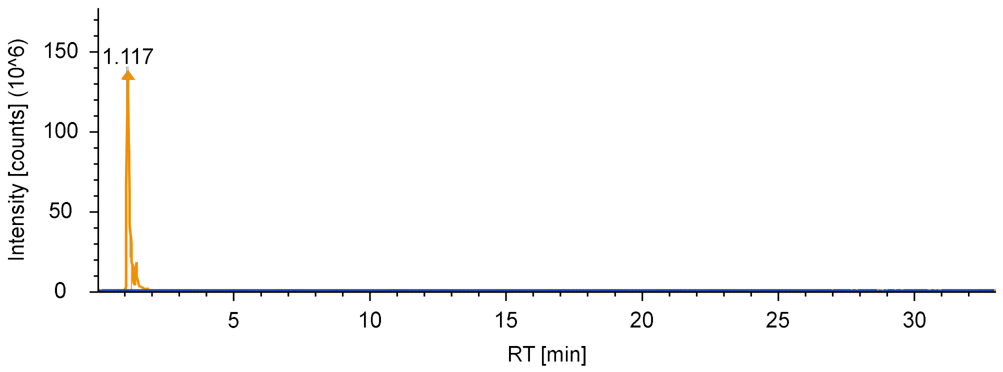

KratomEtOH96 (F3) #386, RT=1.117 min, MS1, FTMS (-)
C6 H8 O7 as [M-H]-1

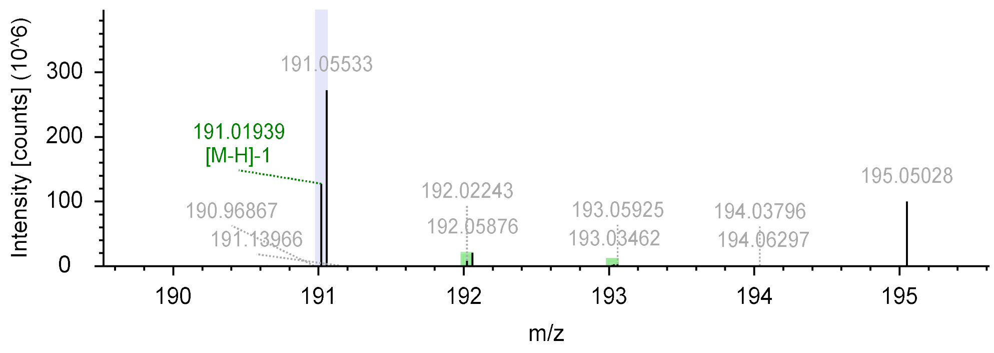

KratomEtOH96 (F3) #365, RT=1.061 min, MS2, FTMS (-), (HCD, DDA, 191.0556@(18;35;53), -1)

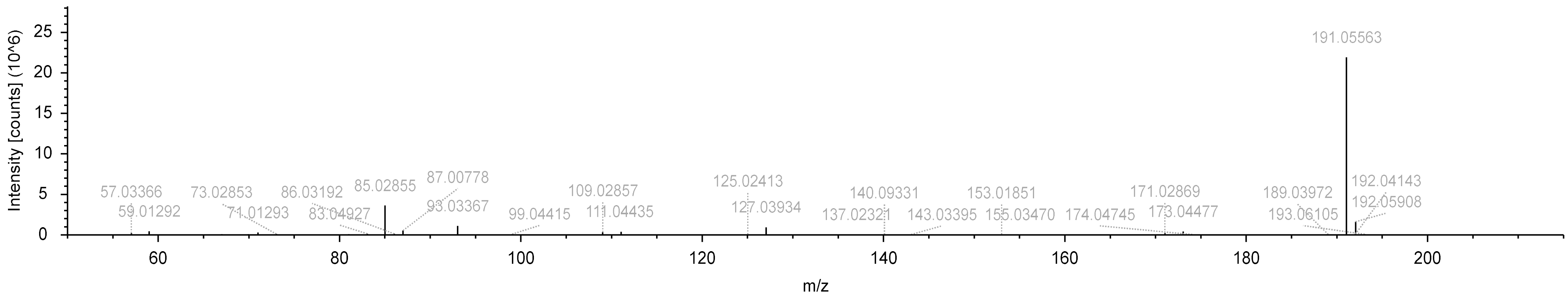

© Reported with Compound Discoverer 3.2

Compounds

Unit Laboratorium Riset Unggulan IPB

Structure	Name	RT [min]	Formula	Molecular Weight	Group Areas
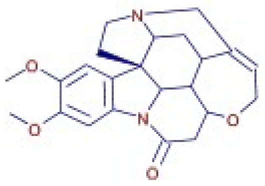	(-)-Brucine	13.13	C23 H26 N2 O4	394.1893	9.69e8

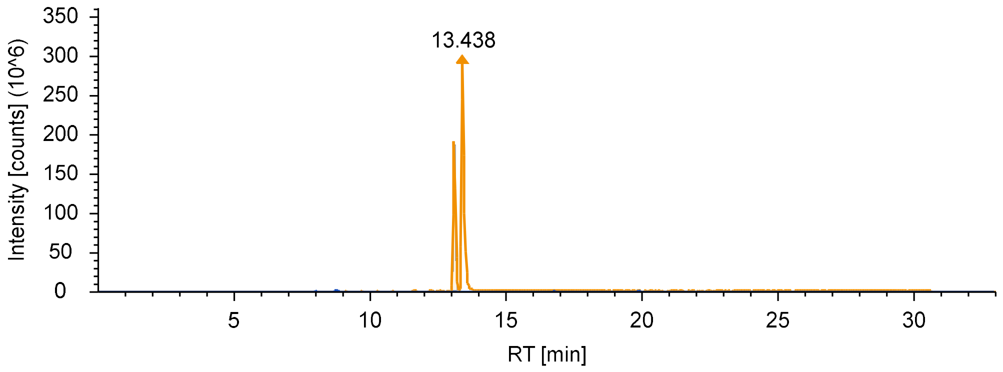

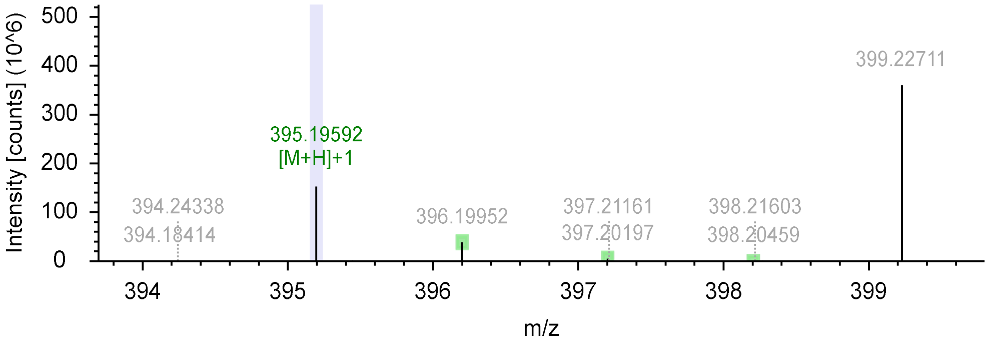

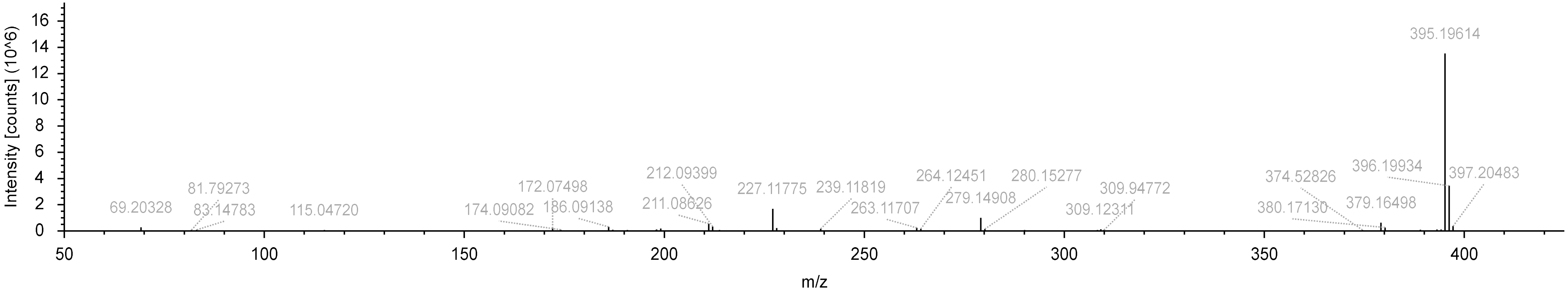

© Reported with Compound Discoverer 3.2

Compounds

Unit Laboratorium Riset Unggulan IPB

Structure	Name	RT [min]	Formula	Molecular Weight	Group Areas
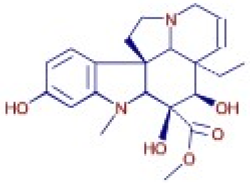	Methyl (2beta,3beta,4beta,5alpha,12beta,19alpha)-3,4,16-trihydroxy-1-methyl-6,7-didehydroaspidospermidine-3-carboxylate	11.27	C22 H28 N2 O5	400.1998	9.19e8

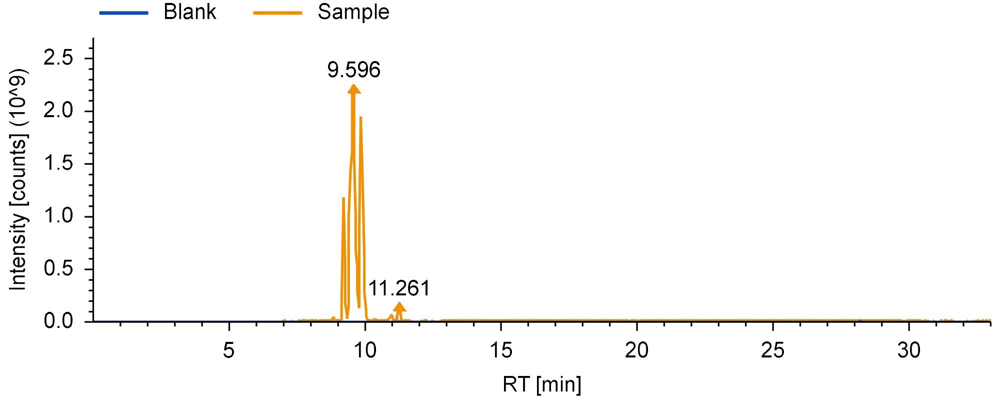

KratomEtOH96 (F3) #3872, RT=11.261 min, MS1, FTMS (+)
C22 H28 N2 O5 as [M+H]+1

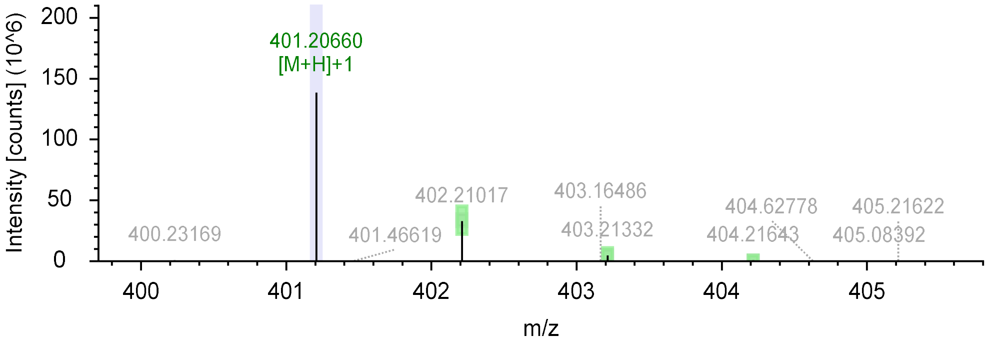

KratomEtOH96 (F3) #3873, RT=11.266 min, MS2, FTMS (+), (HCD, DDA, 401.2067@(18;35;53), +1)

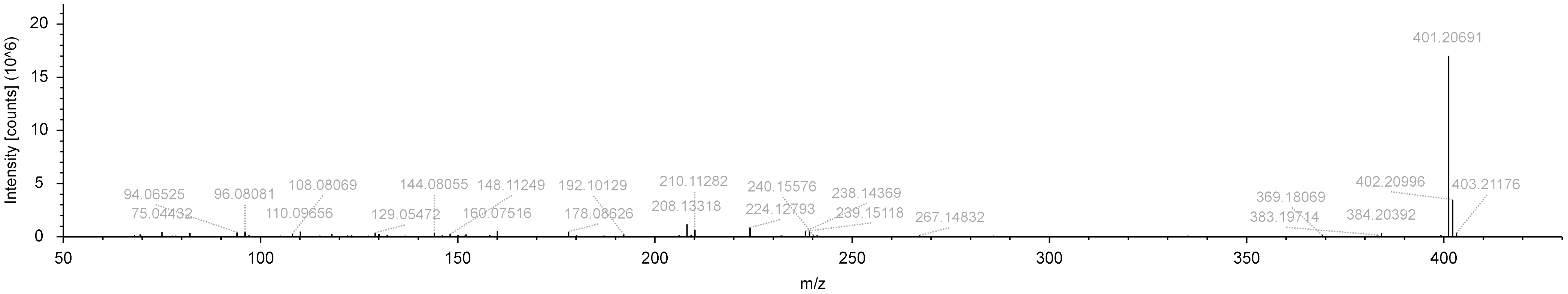

© Reported with Compound Discoverer 3.2

Structure	Name	RT [min]	Formula	Molecular Weight	Group Areas
		28.91	C36 H38 N4 O5	606.2842	8.94e8

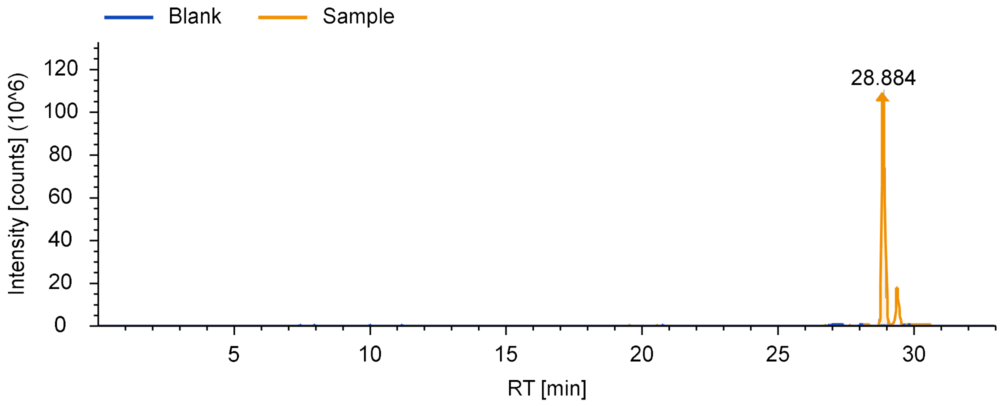

KratomEtOH96 (F3) #9872, RT=28.918 min, MS1, FTMS (+)
C36 H38 N4 O5 as [M+H]+1

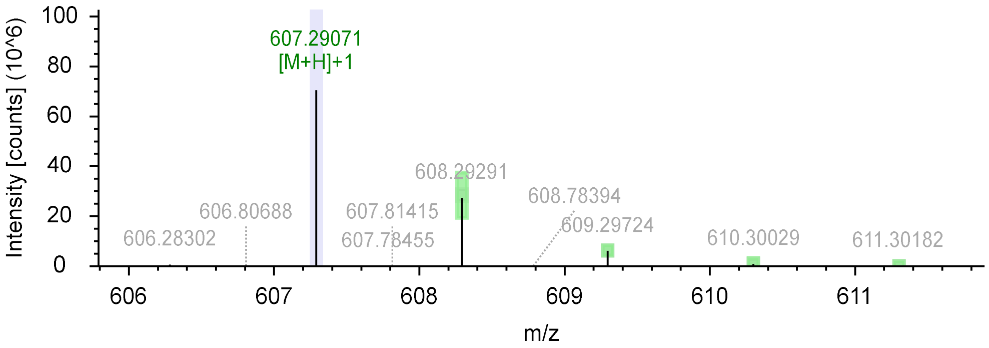

KratomEtOH96 (F3) #9897, RT=28.994 min, MS2, FTMS (+), (HCD, DDA, 607.2905@(18;35;53), +1)

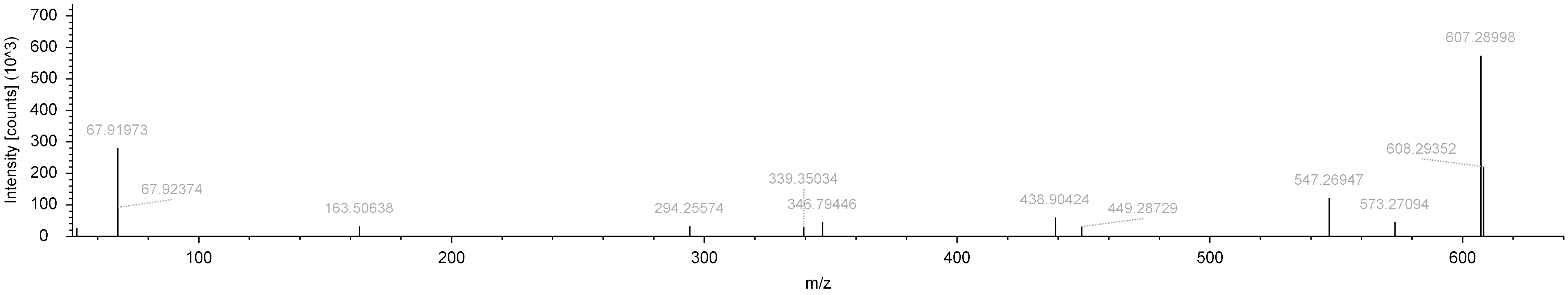

© Reported with Compound Discoverer 3.2

Structure	Name	RT [min]	Formula	Molecular Weight	Group Areas
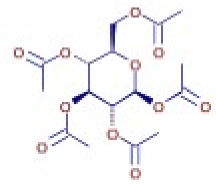	beta-d-glucose pentaacetate	6.16	C16 H22 O11	390.1162	8.23e8

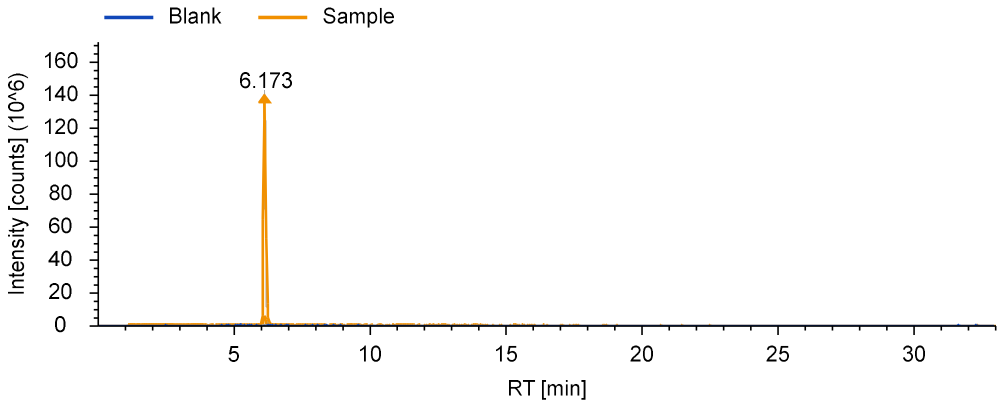

KratomEtOH96 (F3) #2102, RT=6.173 min, MS1, FTMS (-)
C16 H22 O11 as [M-H]-1

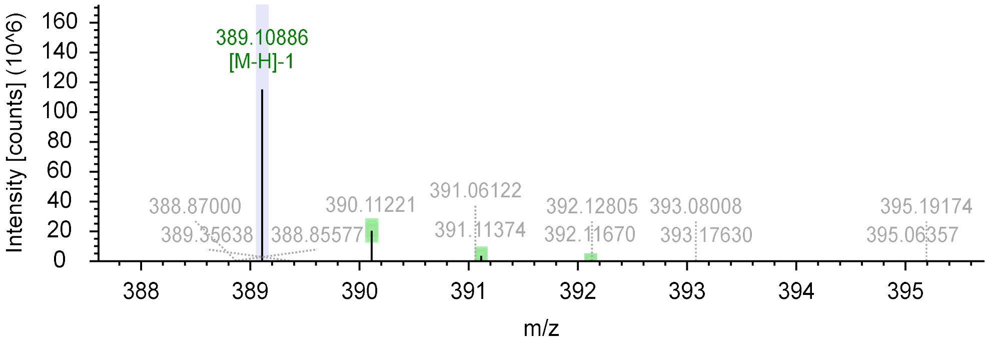

KratomEtOH96 (F3) #2069, RT=6.083 min, MS2, FTMS (-), (HCD, DDA, 389.1091@(18;35;53), -1)

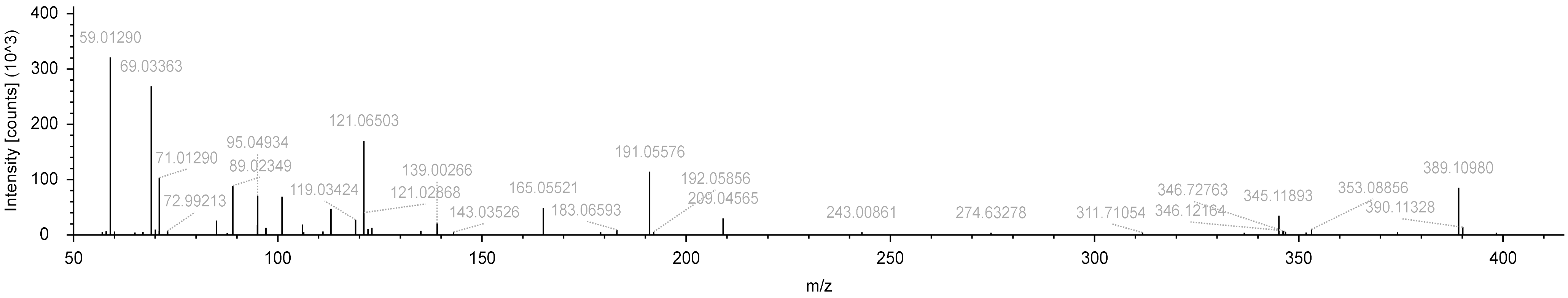

© Reported with Compound Discoverer 3.2

Structure	Name	RT [min]	Formula	Molecular Weight	Group Areas
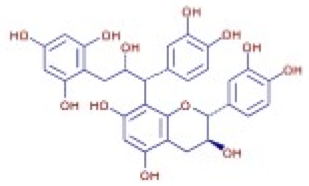	Gambiriin A1	7.24	C30 H28 O12	580.1581	8.17e8

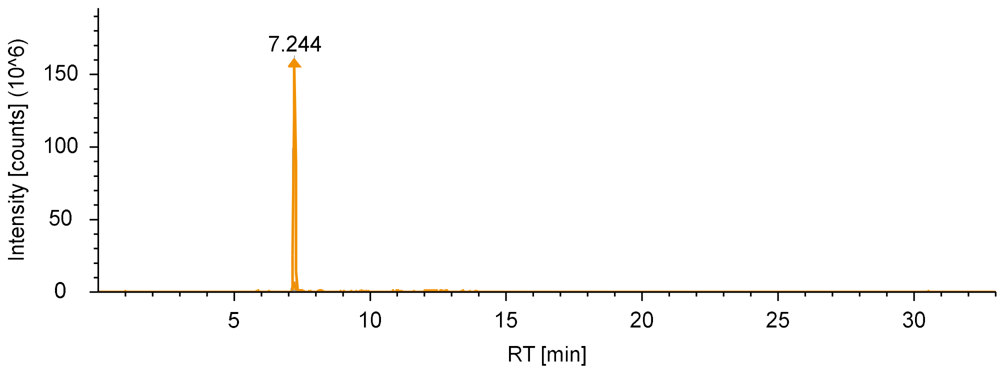

KratomEtOH96 (F3) #2474, RT=7.244 min, MS1, FTMS (-)
C30 H28 O12 as [M-H]-1

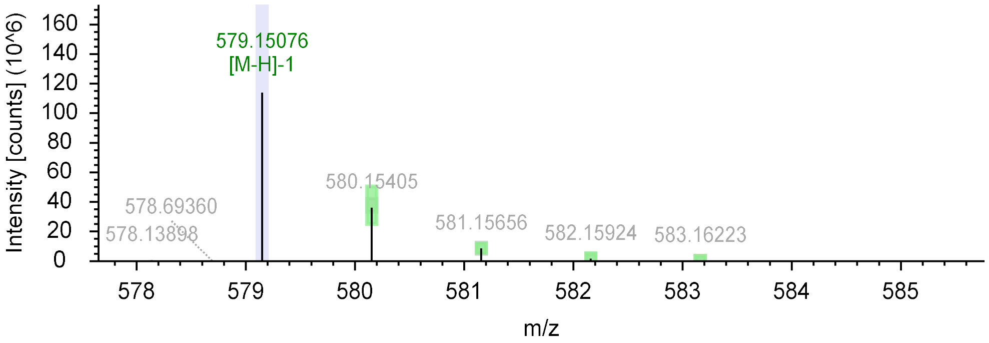

KratomEtOH96 (F3) #2454, RT=7.189 min, MS2, FTMS (-), (HCD, DDA, 579.1508@(18;35;53), -1)

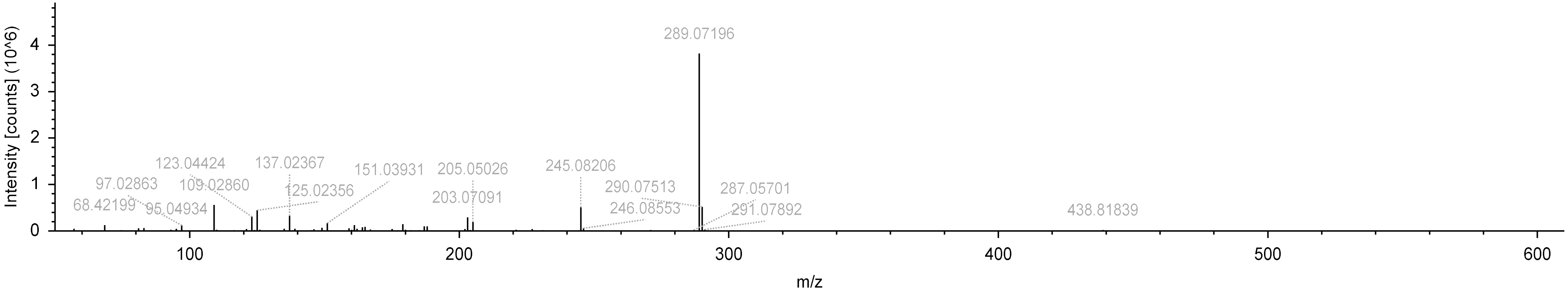

© Reported with Compound Discoverer 3.2

Structure	Name	RT [min]	Formula	Molecular Weight	Group Areas
		10.63	C22 H28 N2 O6	416.1947	8.15e8

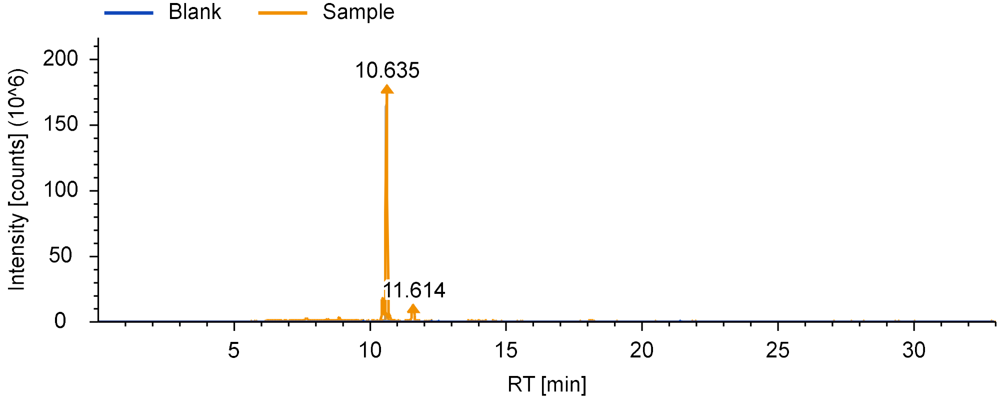

KratomEtOH96 (F3) #3656, RT=10.635 min, MS1, FTMS (+)
C22 H28 N2 O6 as [M+H]+1

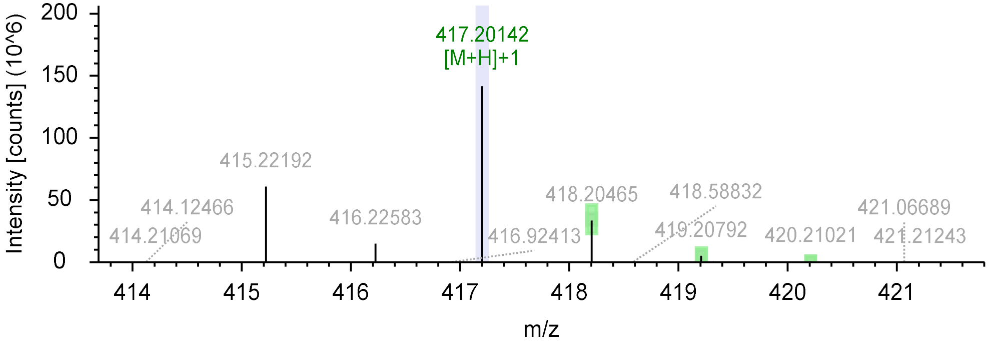

KratomEtOH96 (F3) #3659, RT=10.641 min, MS2, FTMS (+), (HCD, DDA, 417.2014@(18;35;53), +1)

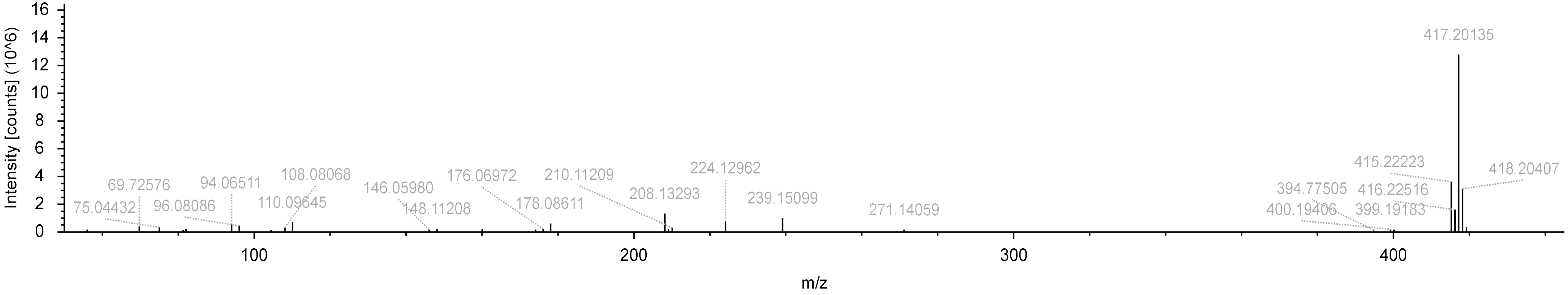

© Reported with Compound Discoverer 3.2

Structure	Name	RT [min]	Formula	Molecular Weight	Group Areas
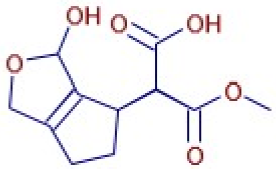	2-(3-Hydroxy-3,4,5,6-tetrahydro-1H-cyclopenta[c]furan-4-yl)-3-methoxy-3-oxopropanoic acid	7.67	C11 H14 O6	242.0790	7.46e8

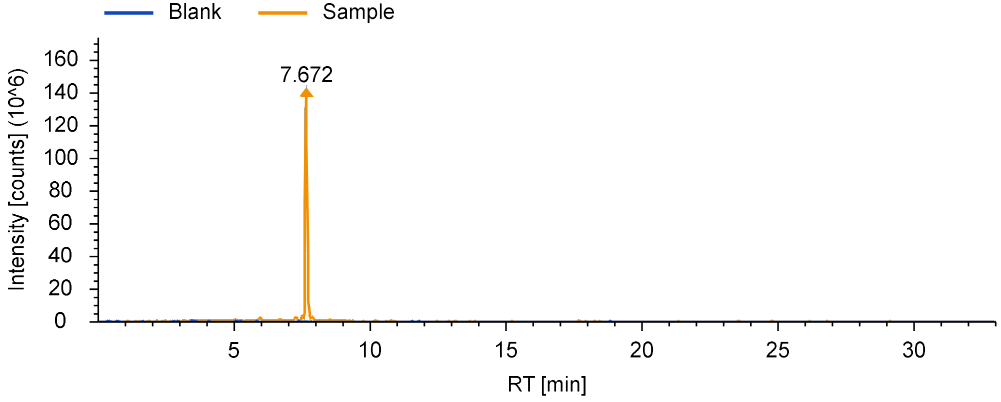

KratomEtOH96 (F3) #2624, RT=7.672 min, MS1, FTMS (+)
C11 H14 O6 as [M+H]+1

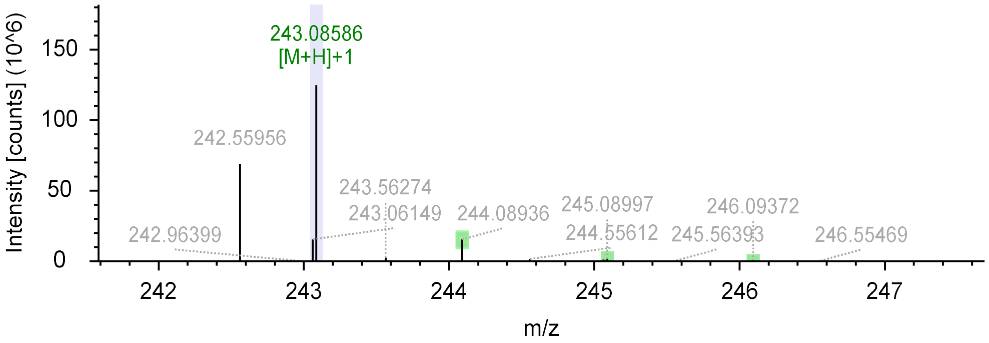

KratomEtOH96 (F3) #2663, RT=7.777 min, MS2, FTMS (+), (HCD, DDA, 242.5598@(18;35;53), +2)

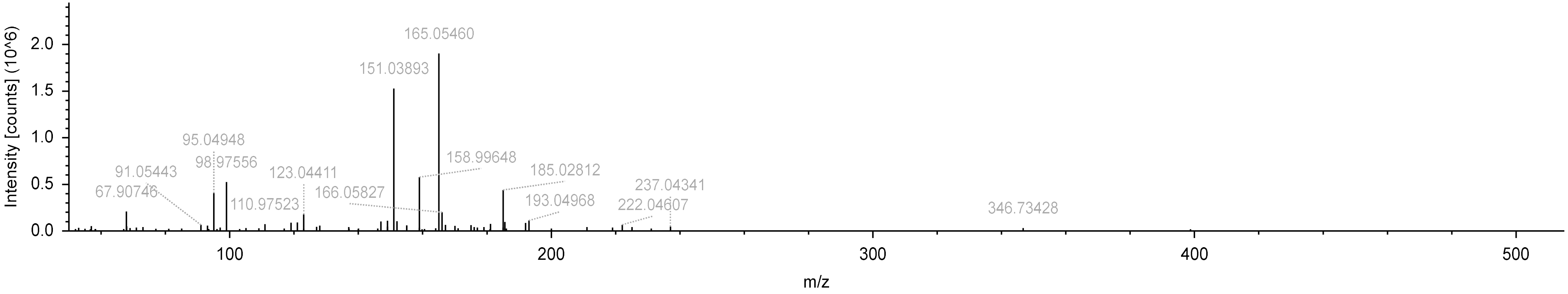

© Reported with Compound Discoverer 3.2

Compounds

Unit Laboratorium Riset Unggulan IPB

Structure	Name	RT [min]	Formula	Molecular Weight	Group Areas
		11.83	C46 H60 N4 O8	796.4411	6.64e8

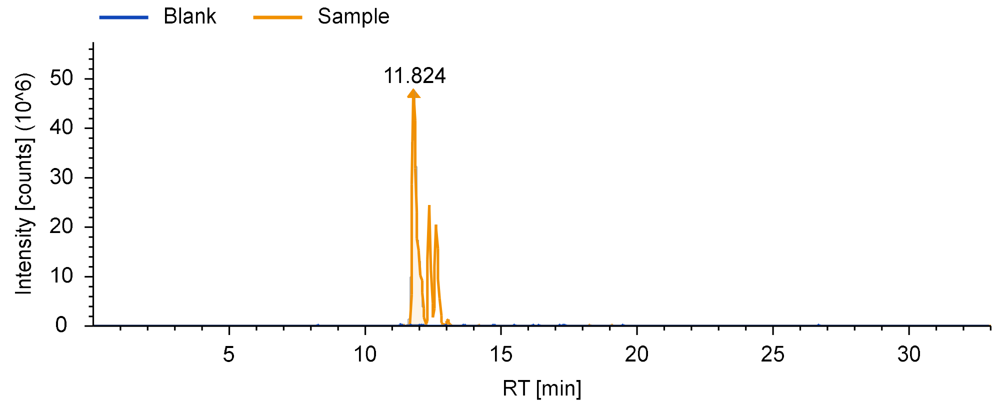

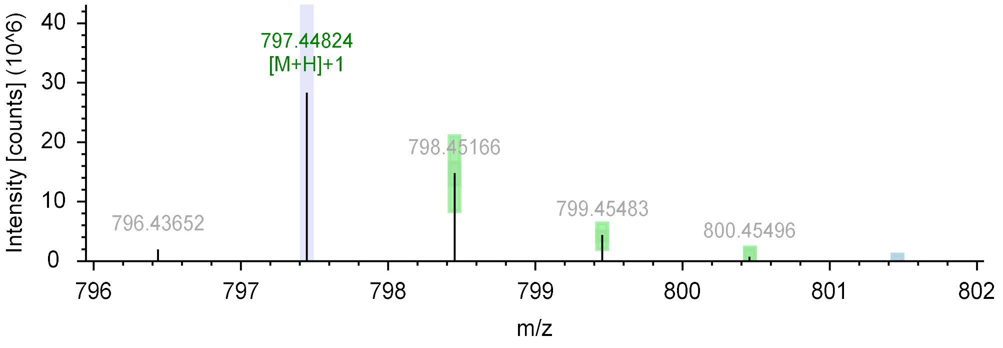

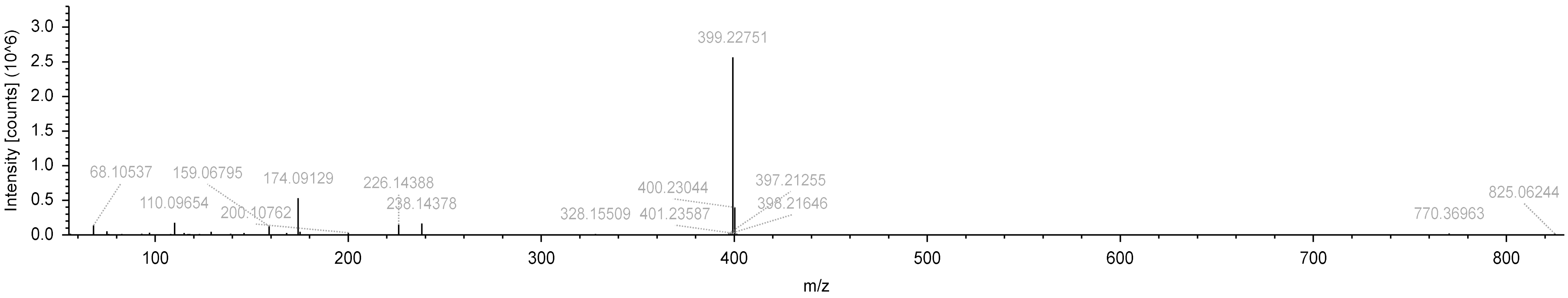

© Reported with Compound Discoverer 3.2

Compounds

Unit Laboratorium Riset Unggulan IPB

Structure	Name	RT [min]	Formula	Molecular Weight	Group Areas
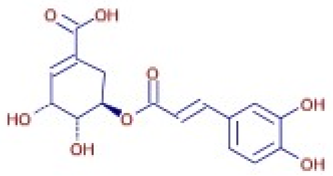	5-CAFFEOYLSHIKIMIC ACID	7.24	C16 H16 O8	336.0845	6.10e8

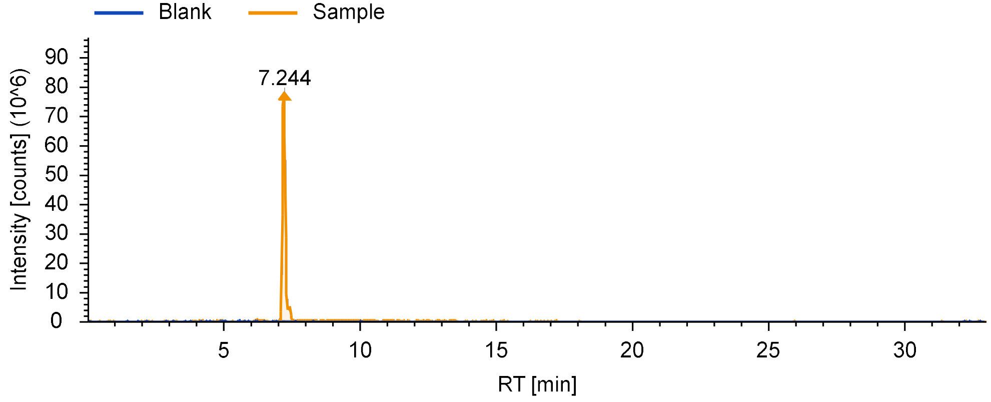

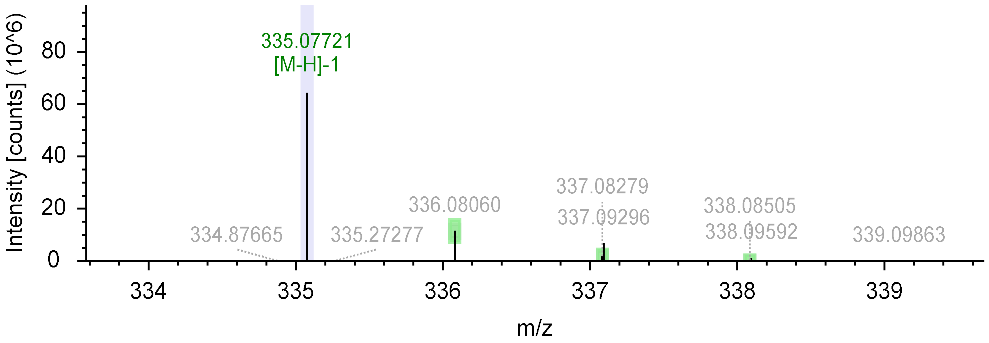

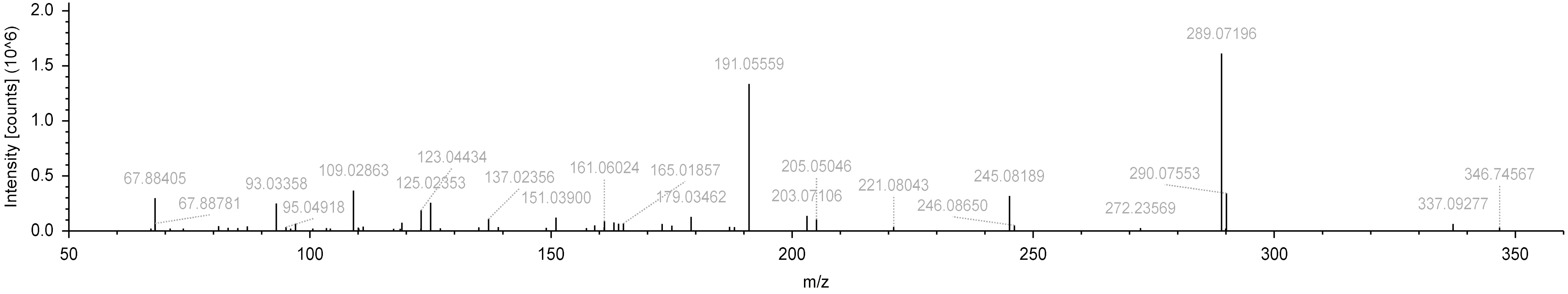

© Reported with Compound Discoverer 3.2

Structure	Name	RT [min]	Formula	Molecular Weight	Group Areas
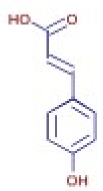	(E)-p-coumaric acid	7.68	C9 H8 O3	164.0473	5.71e8

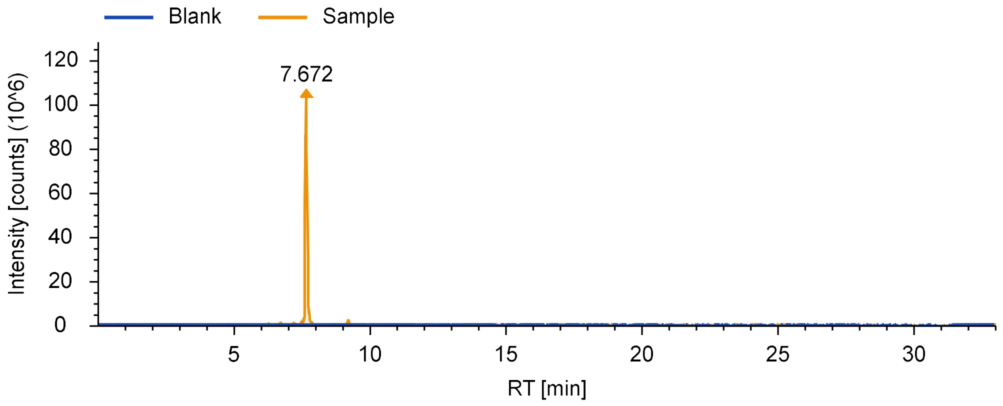

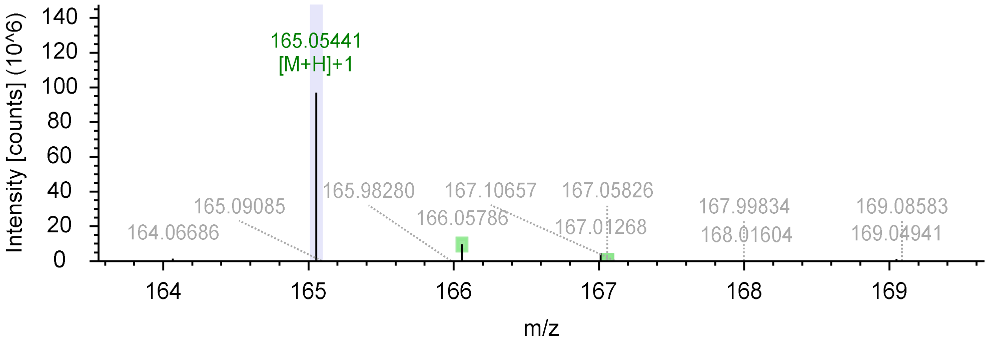

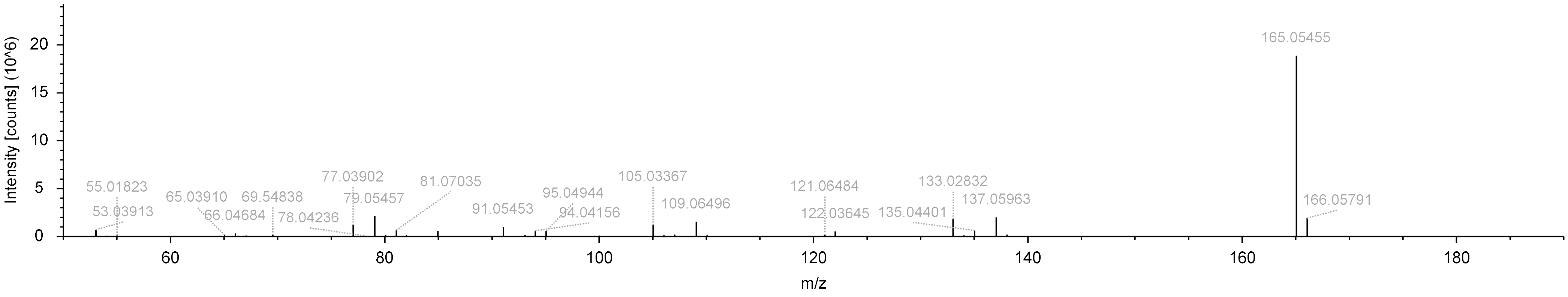

© Reported with Compound Discoverer 3.2

Compounds

Unit Laboratorium Riset Unggulan IPB

Structure	Name	RT [min]	Formula	Molecular Weight	Group Areas
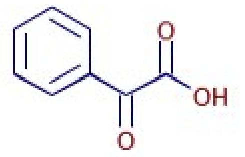	Phenylglyoxylic acid	7.67	C8 H6 O3	150.0317	5.14e8

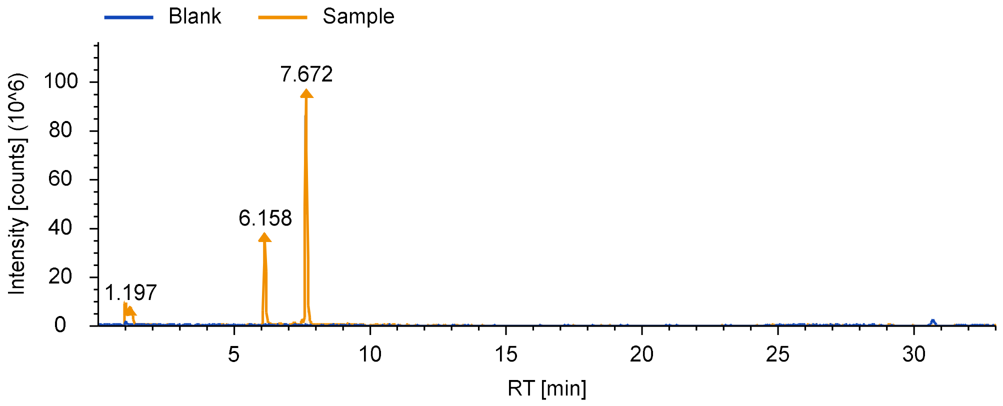

KratomEtOH96 (F3) #2624, RT=7.672 min, MS1, FTMS (+)
C8 H6 O3 as [M+H]+1

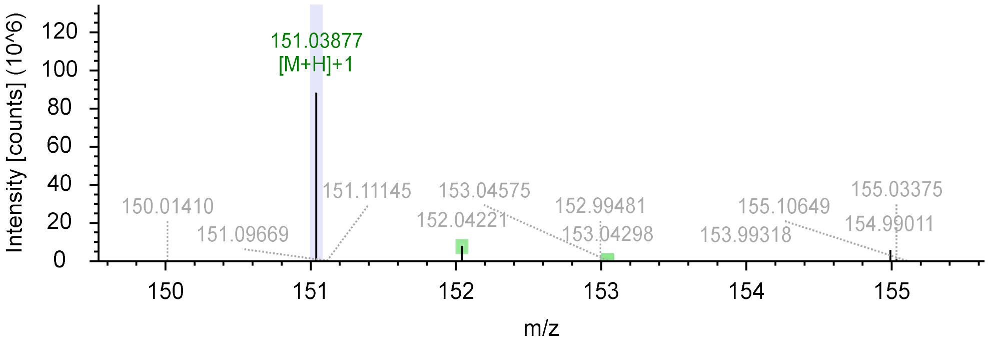

KratomEtOH96 (F3) #2603, RT=7.614 min, MS2, FTMS (+), (HCD, DDA, 151.0390@(18;35;53), +1)

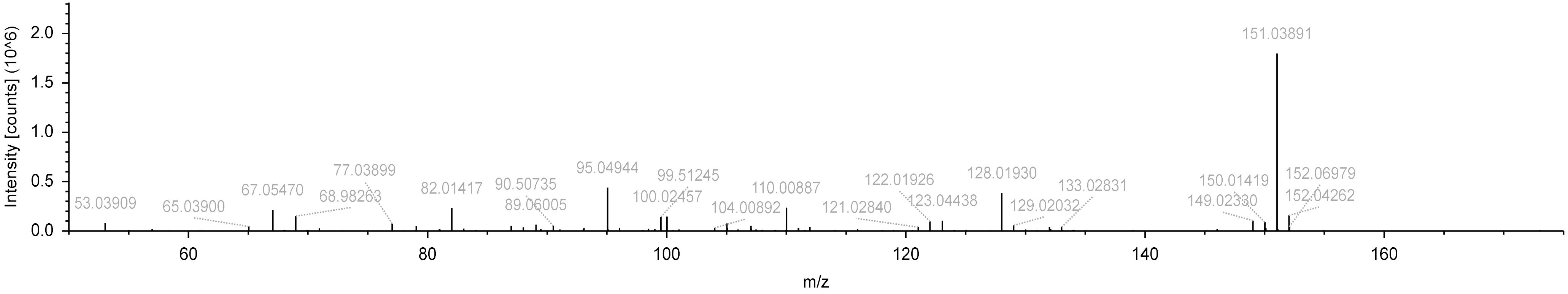

© Reported with Compound Discoverer 3.2

Compounds

Unit Laboratorium Riset Unggulan IPB

Structure	Name	RT [min]	Formula	Molecular Weight	Group Areas
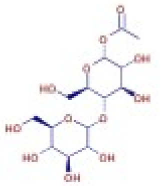	1-O-acetyl-alpha-maltose	1.07	C14 H24 O12	384.1268	5.13e8

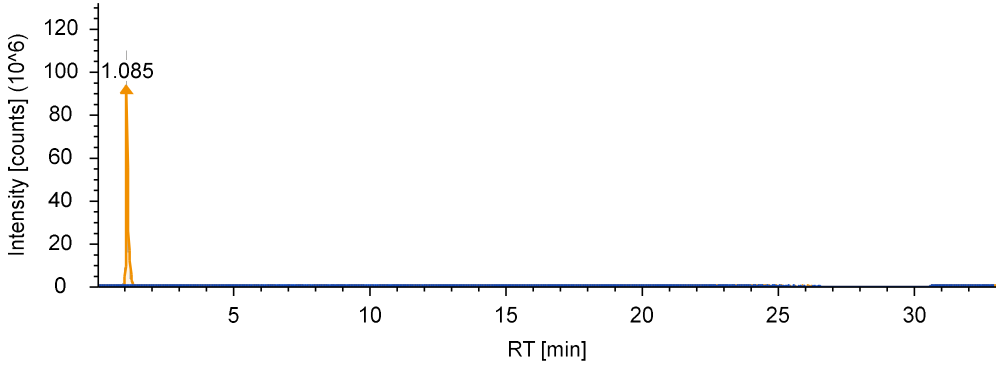

KratomEtOH96 (F3) #362, RT=1.054 min, MS1, FTMS (-)
C14 H24 O12 as [M-H]-1

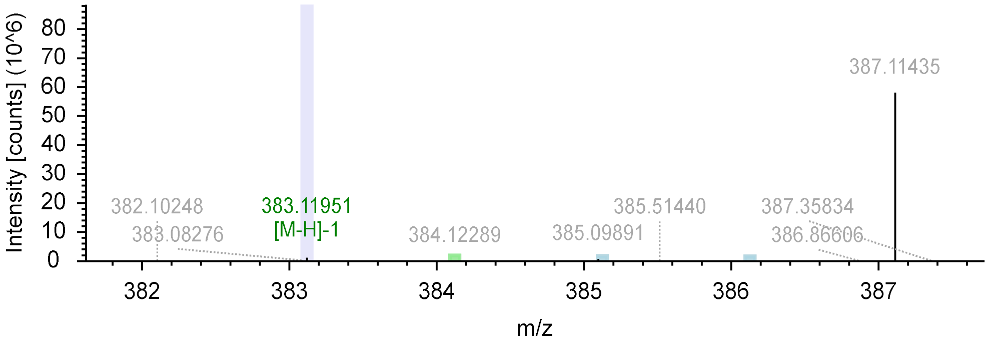

KratomEtOH96 (F3) #377, RT=1.092 min, MS2, FTMS (-), (HCD, DDA, 383.1194@(18;35;53), -1)

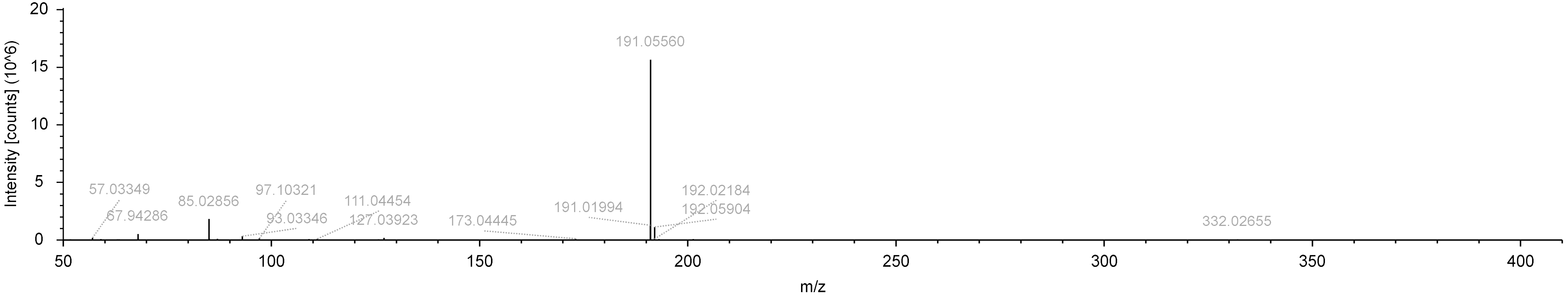

© Reported with Compound Discoverer 3.2

Structure	Name	RT [min]	Formula	Molecular Weight	Group Areas
		1.07	C14 H20 N4 O9	388.1230	5.11e8

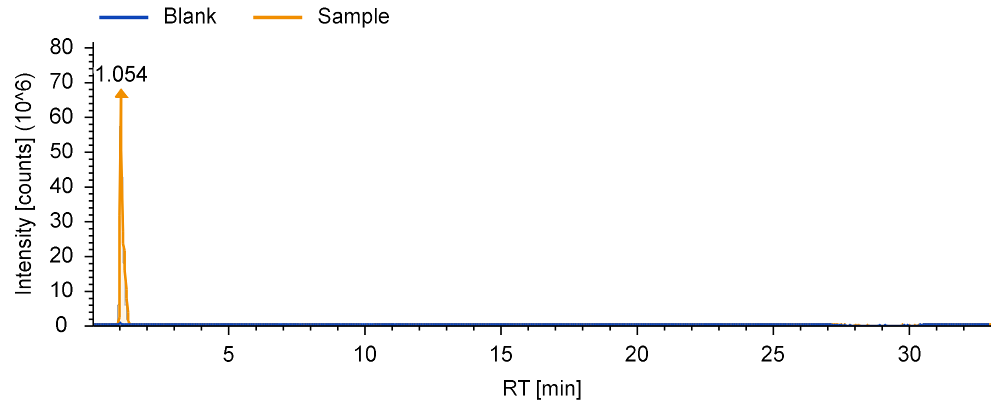

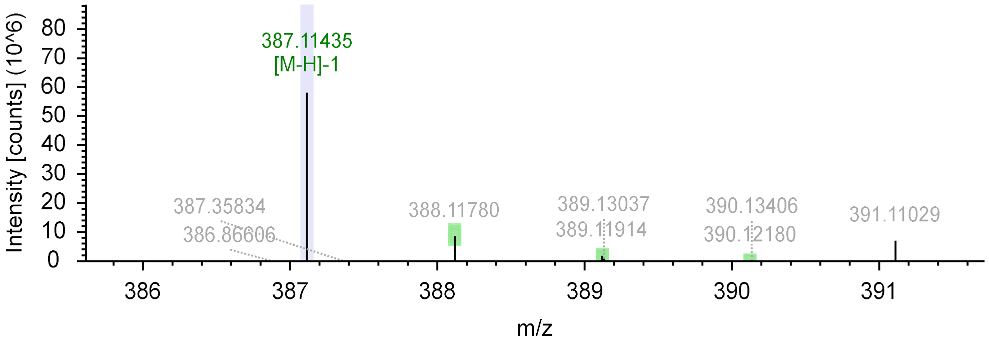

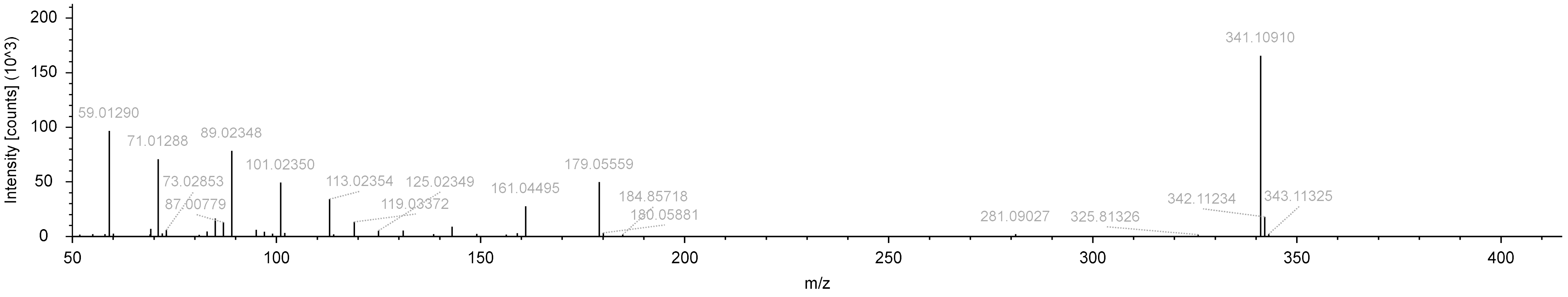

© Reported with Compound Discoverer 3.2

Structure	Name	RT [min]	Formula	Molecular Weight	Group Areas
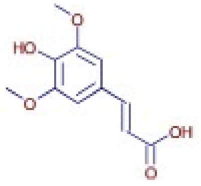	Sinapinic acid	7.67	C11 H12 O5	224.0685	4.90e8

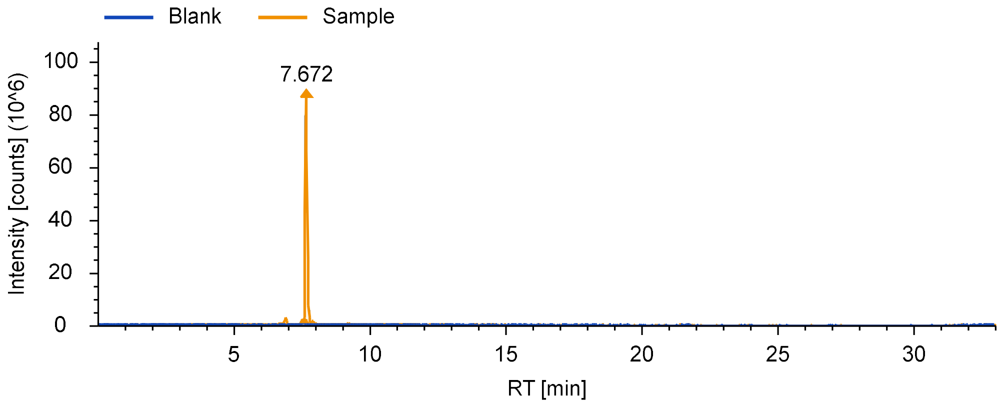

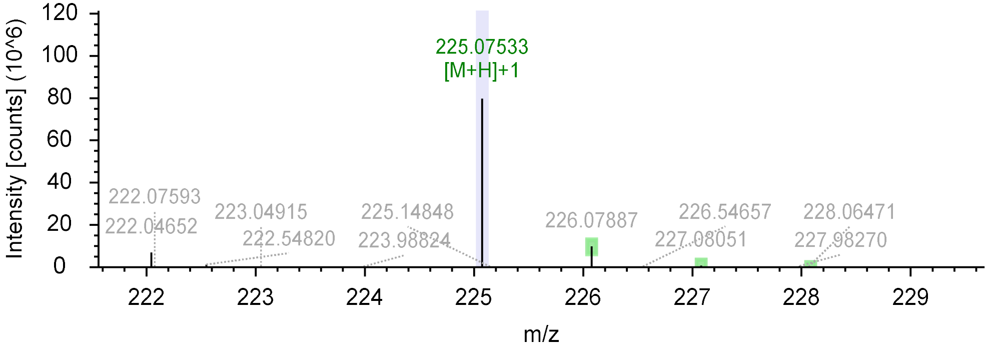

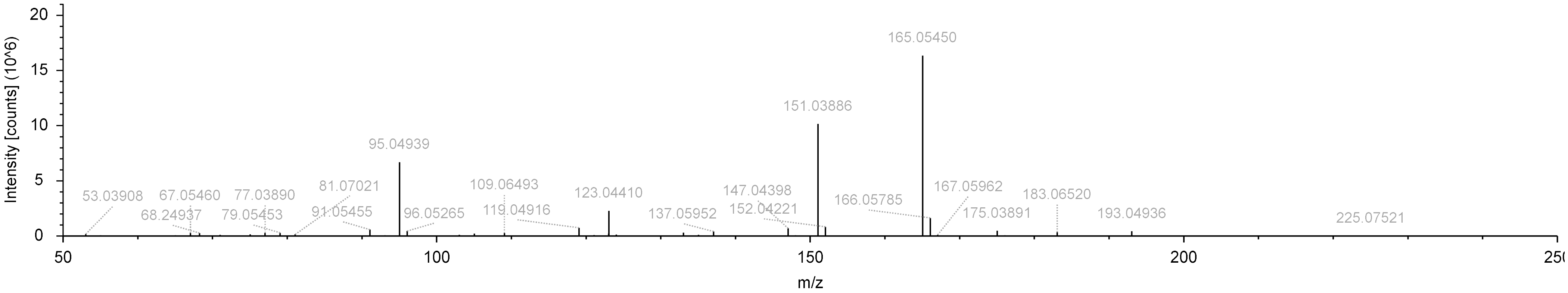

© Reported with Compound Discoverer 3.2

Compounds

Unit Laboratorium Riset Unggulan IPB

Structure	Name	RT [min]	Formula	Molecular Weight	Group Areas
		10.76	C44 H64 N4 O13	856.4470	4.80e8

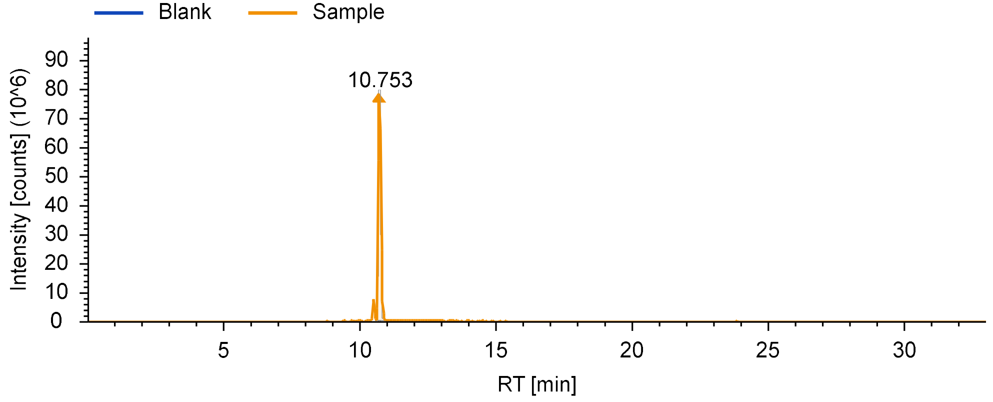

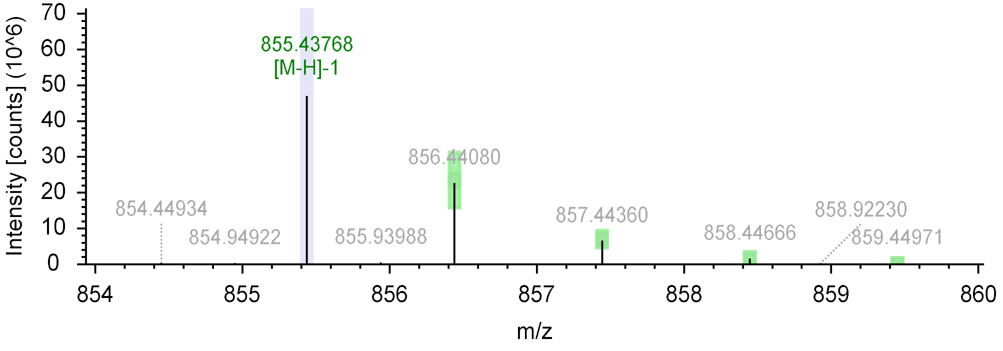

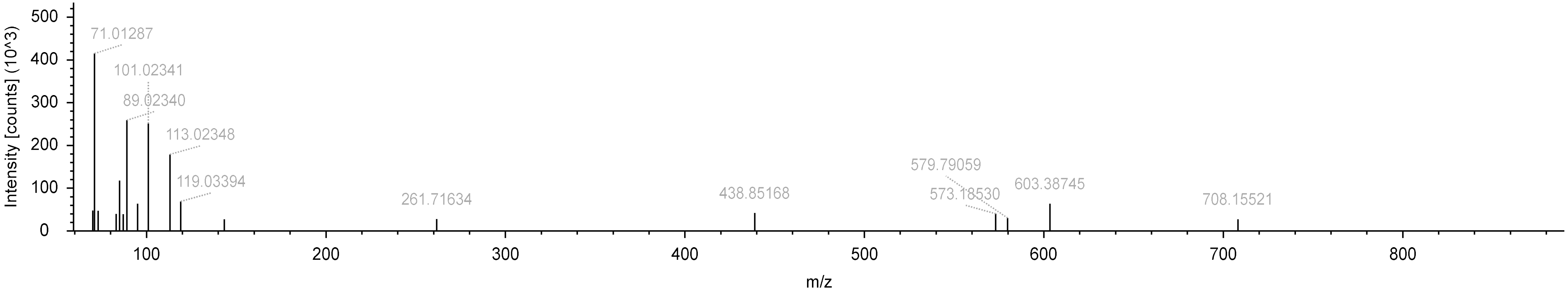

© Reported with Compound Discoverer 3.2

Compounds

Unit Laboratorium Riset Unggulan IPB

Structure	Name	RT [min]	Formula	Molecular Weight	Group Areas
		1.14	C7 H12 O8	224.0532	4.76e8

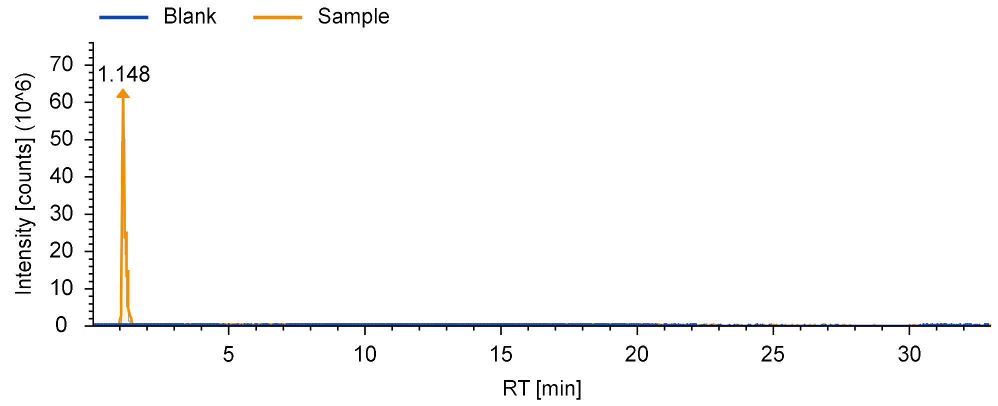

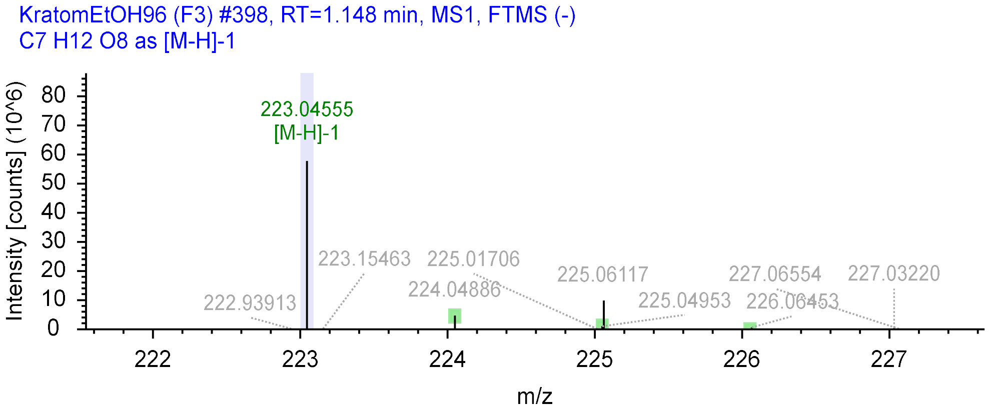

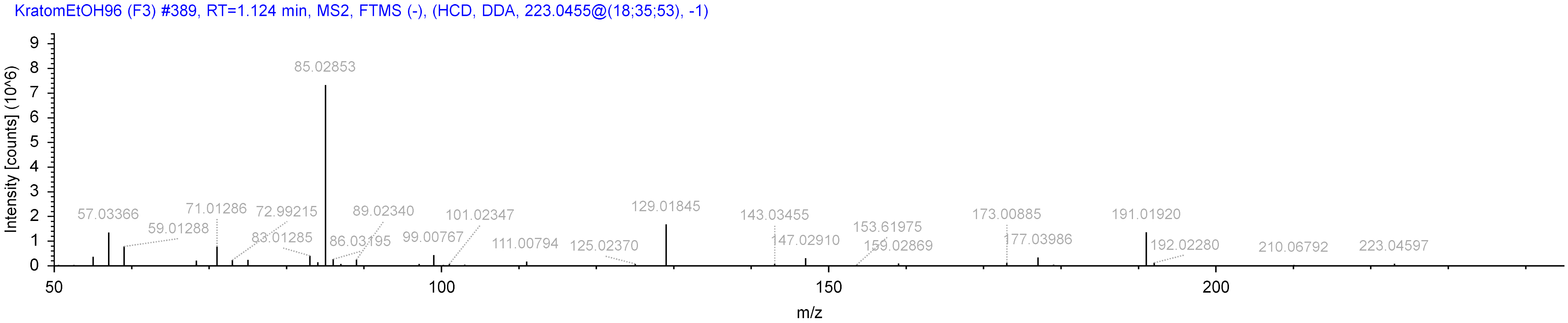

© Reported with Compound Discoverer 3.2

Structure	Name	RT [min]	Formula	Molecular Weight	Group Areas
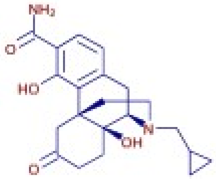	Samidorphan	8.42	C21 H26 N2 O4	370.1893	4.74e8

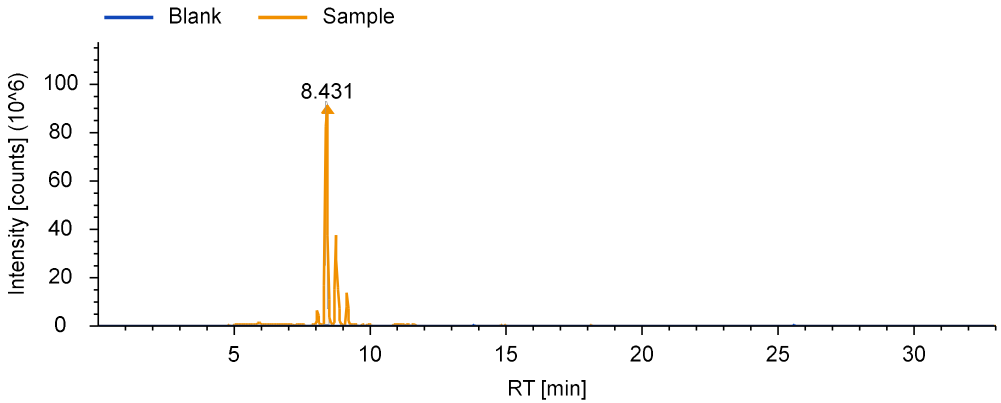

KratomEtOH96 (F3) #2888, RT=8.431 min, MS1, FTMS (+)
C21 H26 N2 O4 as [M+H]+1

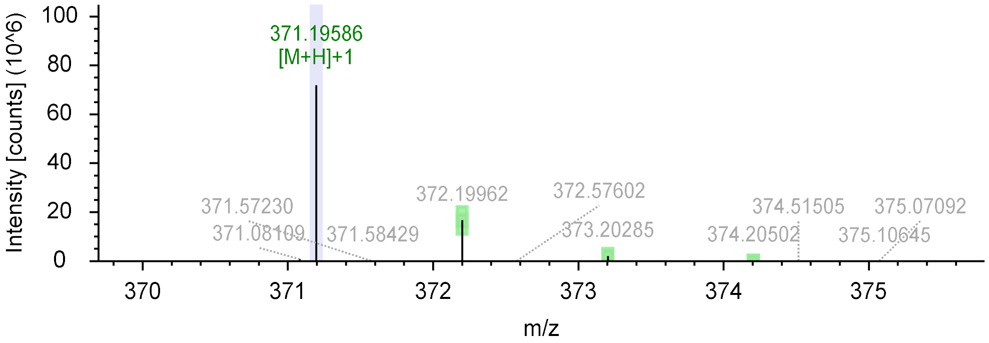

KratomEtOH96 (F3) #2867, RT=8.369 min, MS2, FTMS (+), (HCD, DDA, 371.1962@(18;35;53), +1)

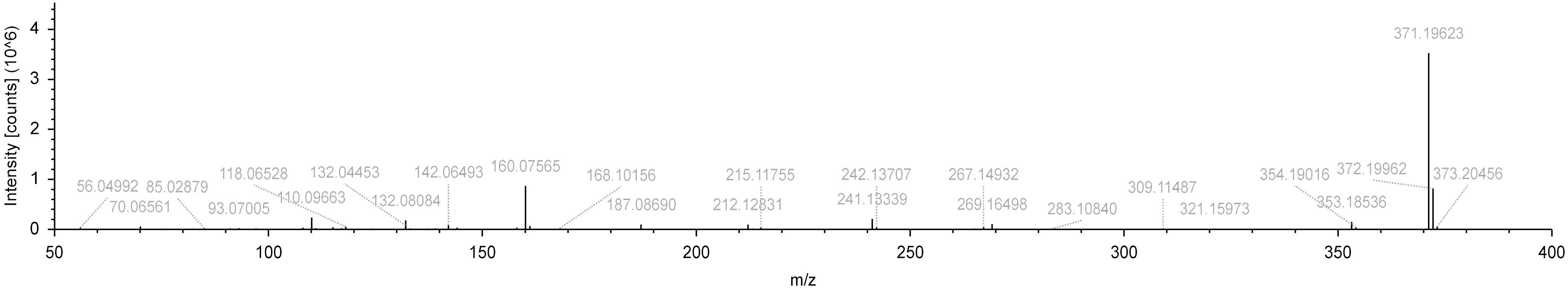

© Reported with Compound Discoverer 3.2

Compounds

Unit Laboratorium Riset Unggulan IPB

Structure	Name	RT [min]	Formula	Molecular Weight	Group Areas
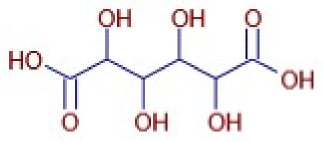	Hexaric acid	1.11	C6 H10 O8	210.0376	4.61e8

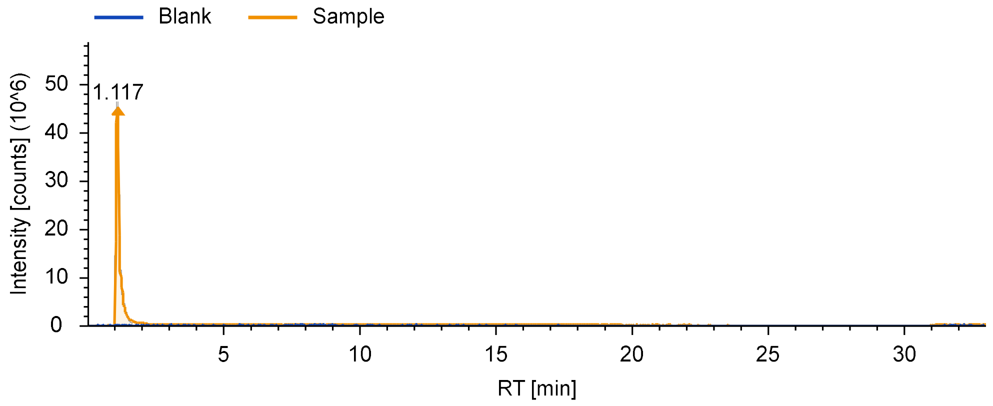

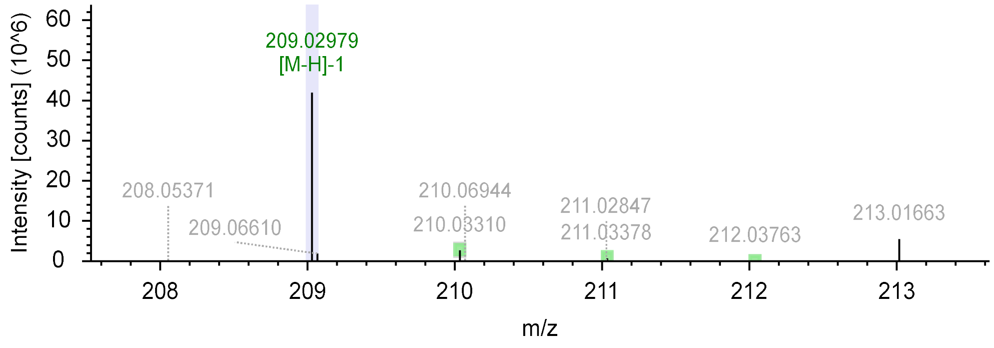

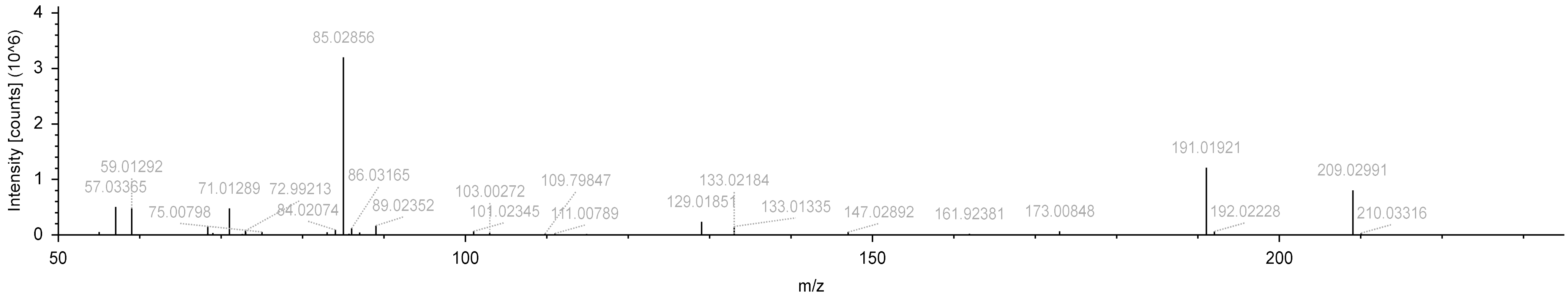

© Reported with Compound Discoverer 3.2

Compounds

Unit Laboratorium Riset Unggulan IPB

Structure	Name	RT [min]	Formula	Molecular Weight	Group Areas
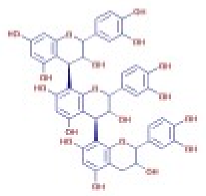	Procyanidin C1	7.55	C45 H38 O18	866.2058	4.09e8

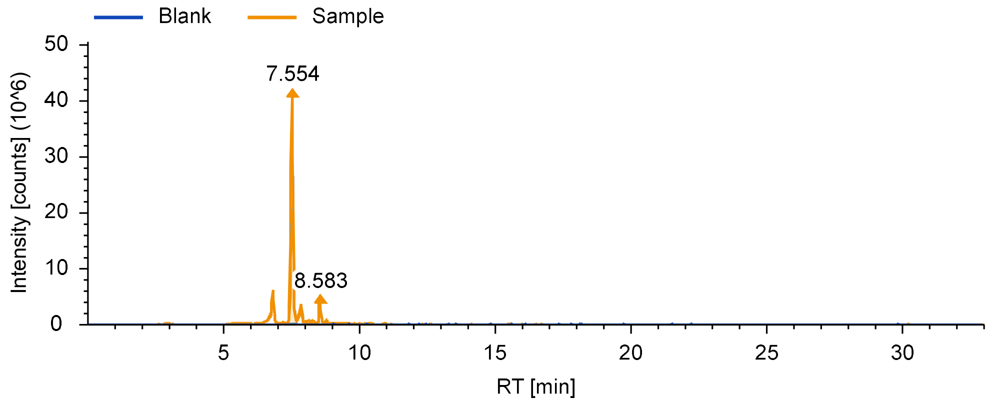

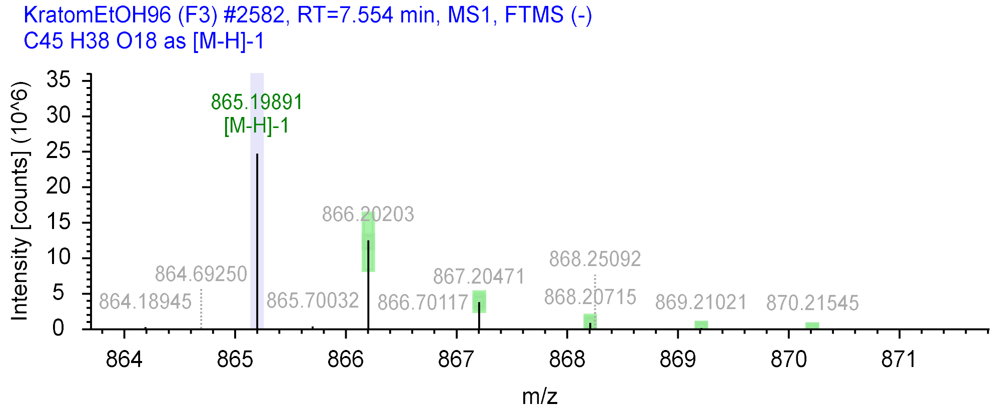

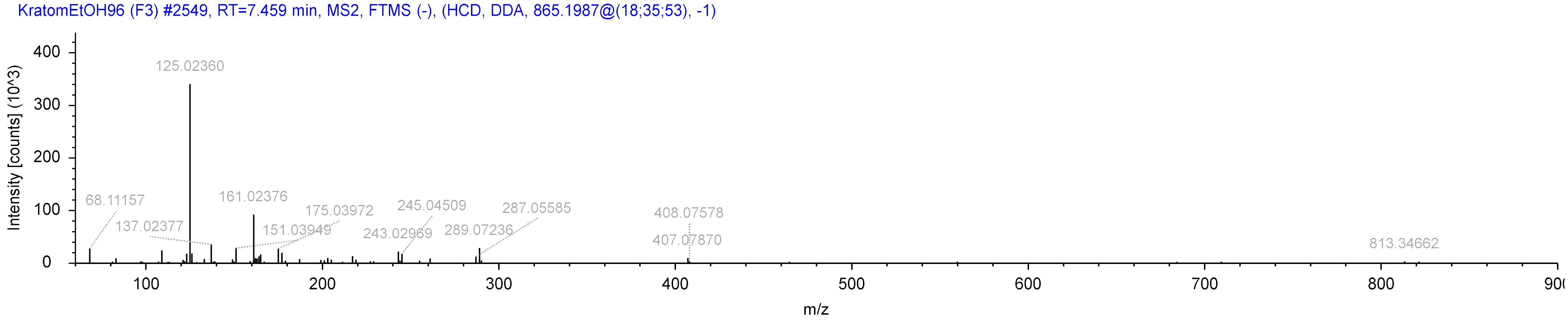

© Reported with Compound Discoverer 3.2

Structure	Name	RT [min]	Formula	Molecular Weight	Group Areas
		8.76	C23 H40 N5 O6 P3	575.2191	4.07e8

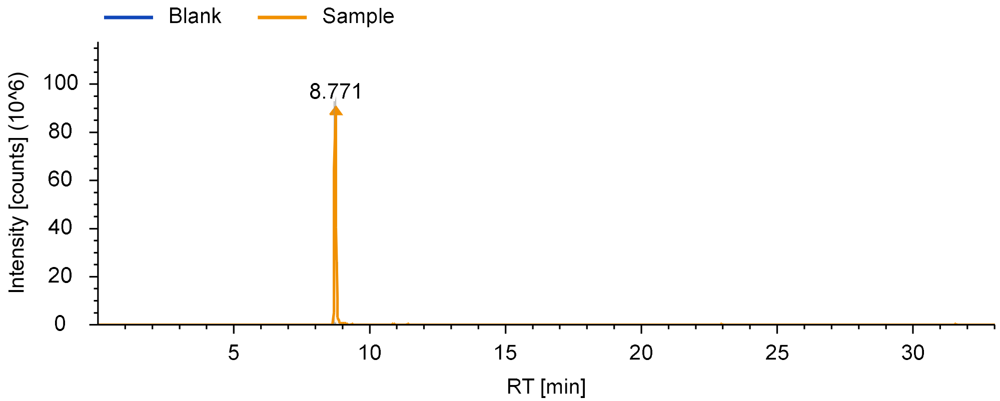

KratomEtOH96 (F3) #3008, RT=8.771 min, MS1, FTMS (+)
C23 H40 N5 O6 P3 as [M+H]+1

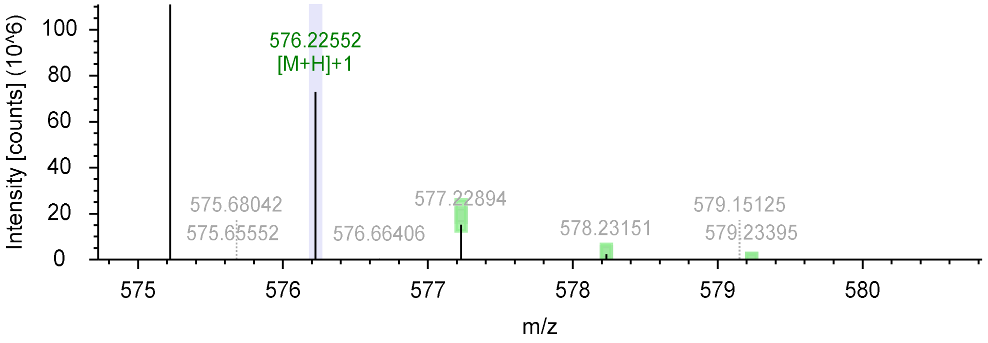

KratomEtOH96 (F3) #2987, RT=8.712 min, MS2, FTMS (+), (HCD, DDA, 575.2234@(18;35;53), +1)

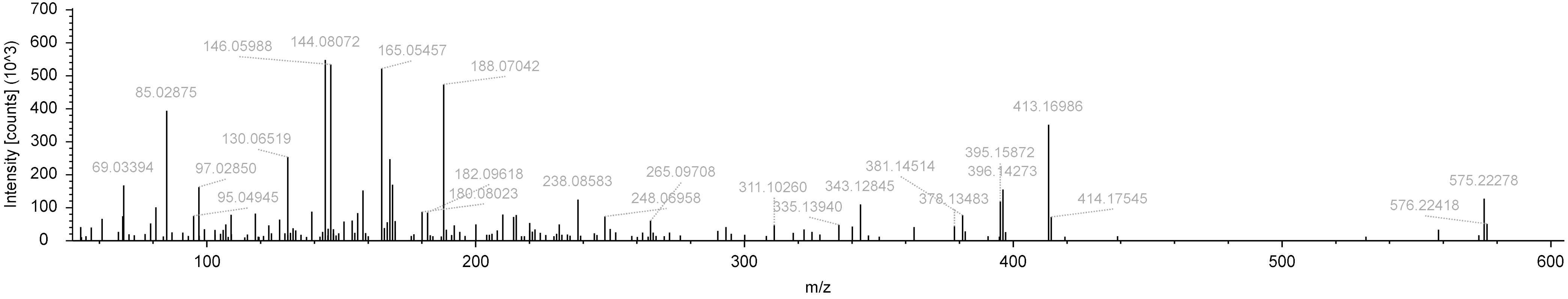

© Reported with Compound Discoverer 3.2

Structure	Name	RT [min]	Formula	Molecular Weight	Group Areas
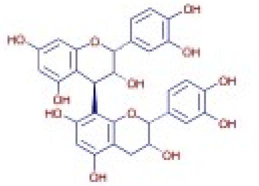	(+)-Procyanidin B2	8.63	C30 H26 O12	578.1424	3.86e8

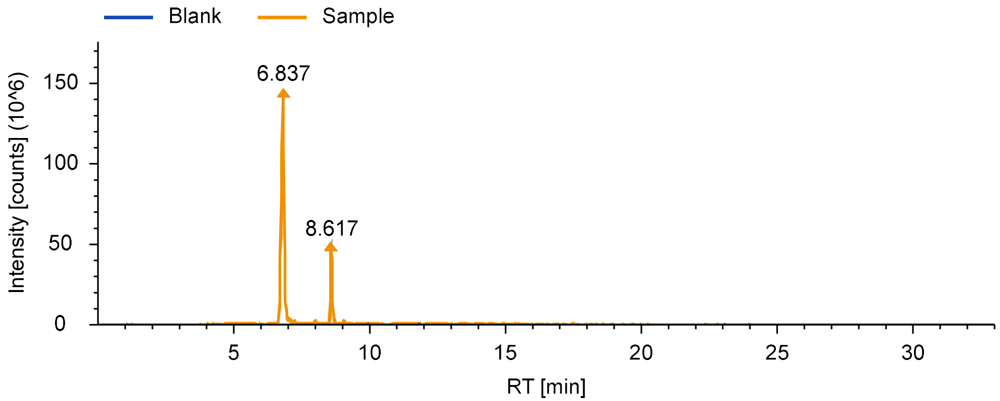

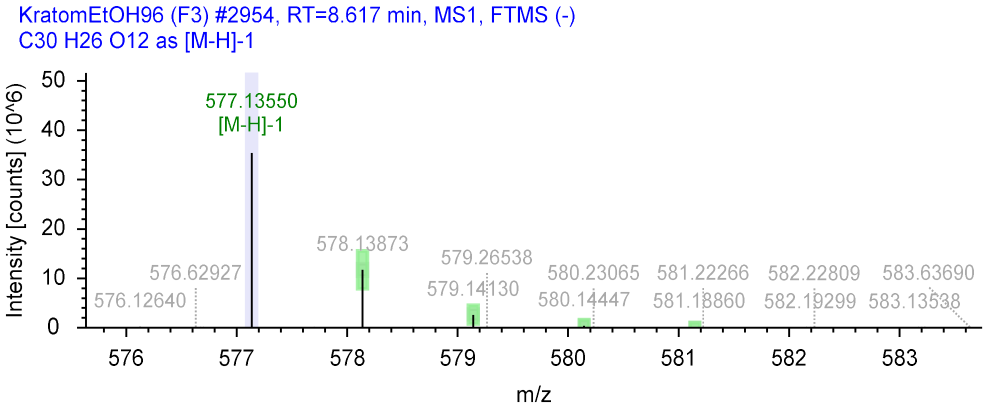

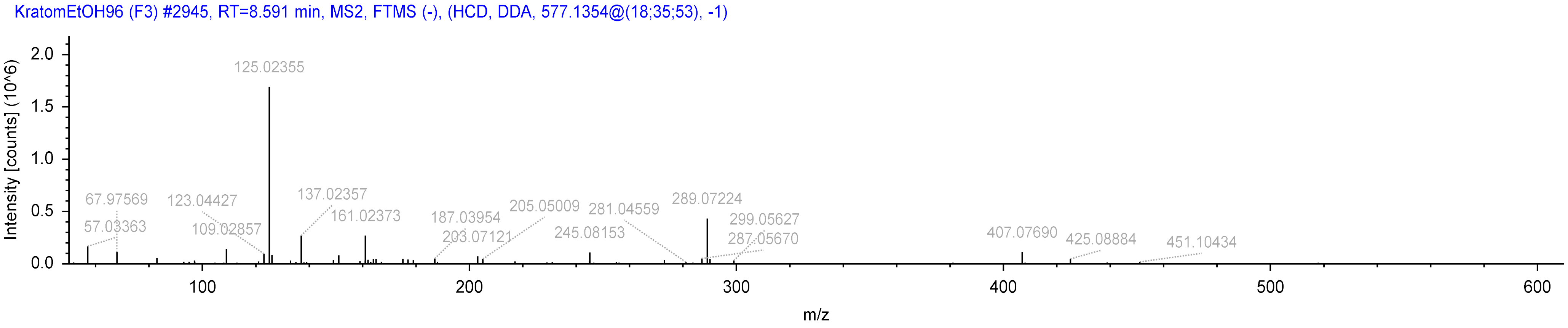

© Reported with Compound Discoverer 3.2

Structure	Name	RT [min]	Formula	Molecular Weight	Group Areas
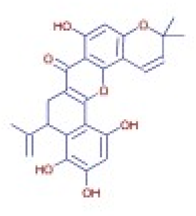	6,10,11,13-Tetrahydroxy-9-isopropenyl-3,3-dimethyl-8,9-dihydro-3H,7H-benzo[c]pyrano[3,2-h]xanthen-7-one	18.31	C25 H22 O7	434.1366	3.62e8

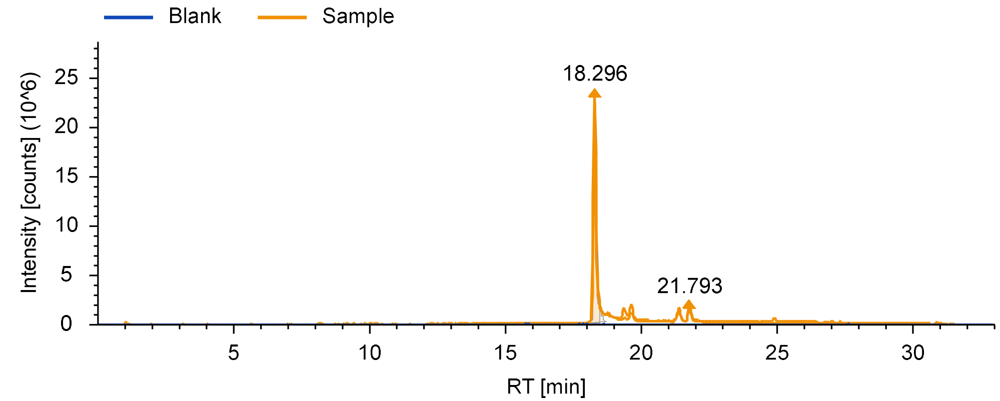

KratomEtOH96 (F3) #6260, RT=18.296 min, MS1, FTMS (+)
C25 H22 O7 as [M+H]+1

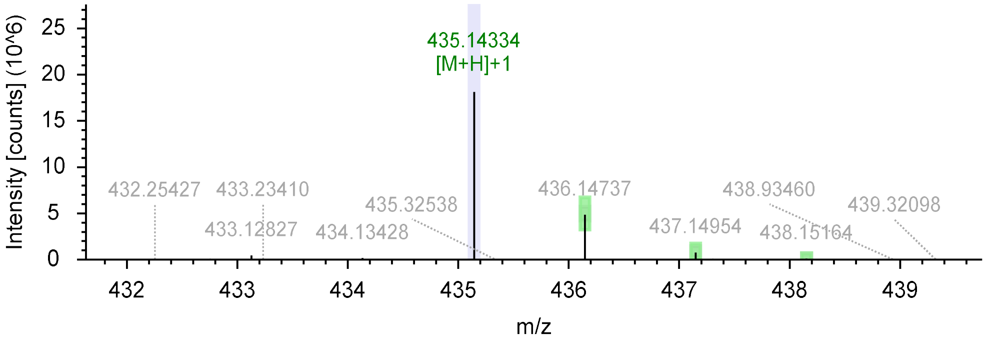

KratomEtOH96 (F3) #6239, RT=18.233 min, MS2, FTMS (+), (HCD, DDA, 435.1440@(18;35;53), +1)

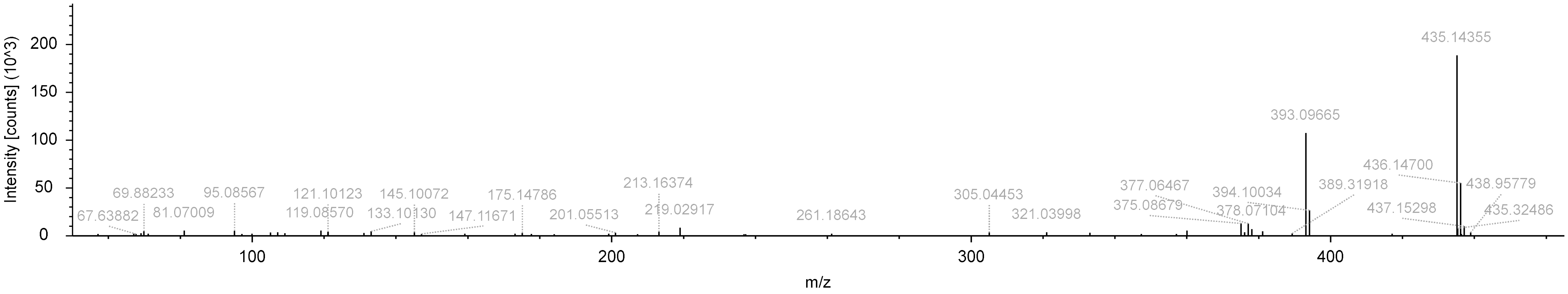

© Reported with Compound Discoverer 3.2

Compounds

Unit Laboratorium Riset Unggulan IPB

Structure	Name	RT [min]	Formula	Molecular Weight	Group Areas
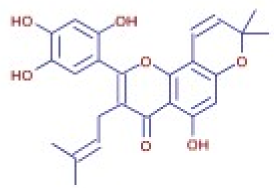	artonin E	18.79	C25 H24 O7	436.1522	3.48e8

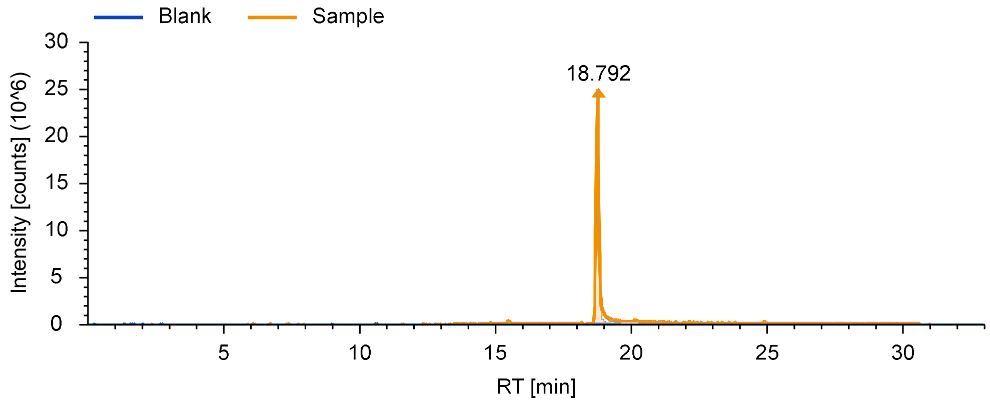

KratomEtOH96 (F3) #6428, RT=18.792 min, MS1, FTMS (+)
C25 H24 O7 as [M+H]+1

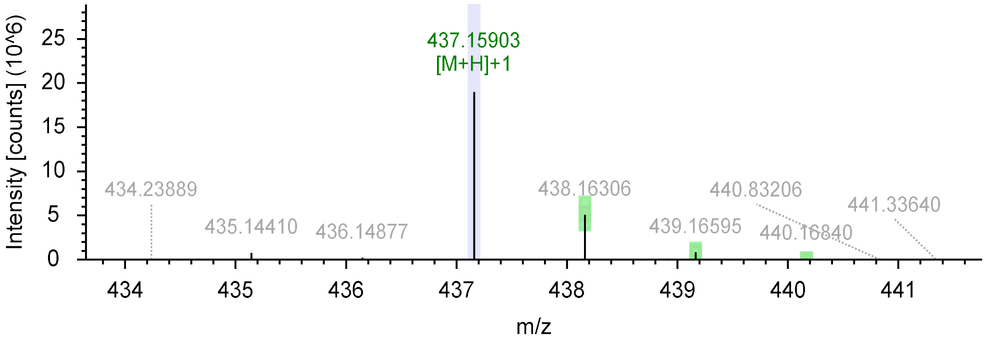

KratomEtOH96 (F3) #6453, RT=18.867 min, MS2, FTMS (+), (HCD, DDA, 437.1593@(18;35;53), +1)

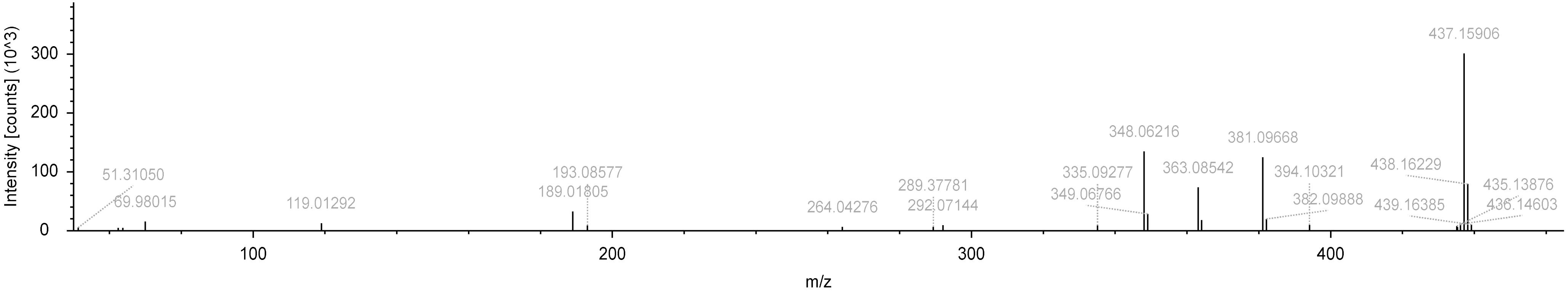

© Reported with Compound Discoverer 3.2

Compounds

Unit Laboratorium Riset Unggulan IPB

Structure	Name	RT [min]	Formula	Molecular Weight	Group Areas
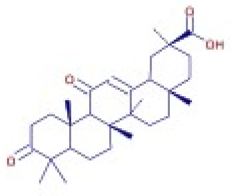	3-Oxoglycyrrhetic acid	11.96	C30 H44 O4	468.3240	3.40e8

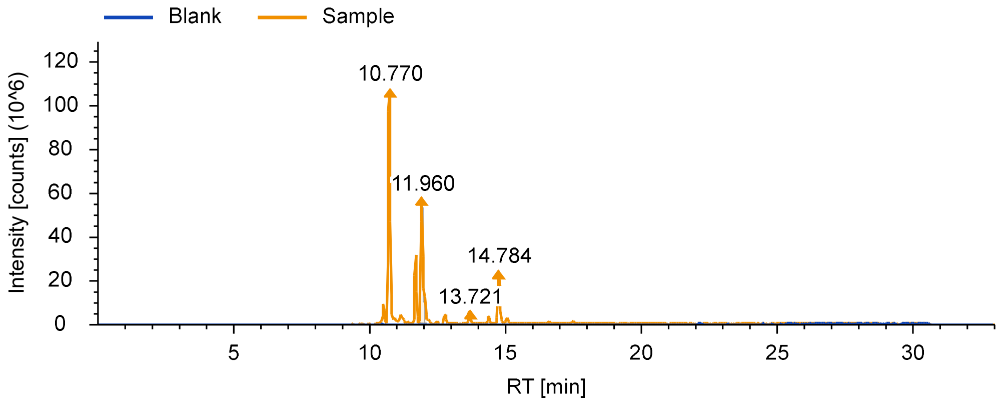

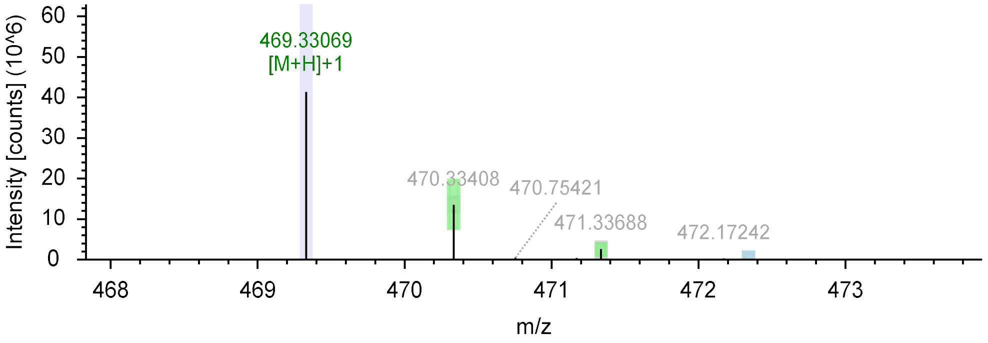

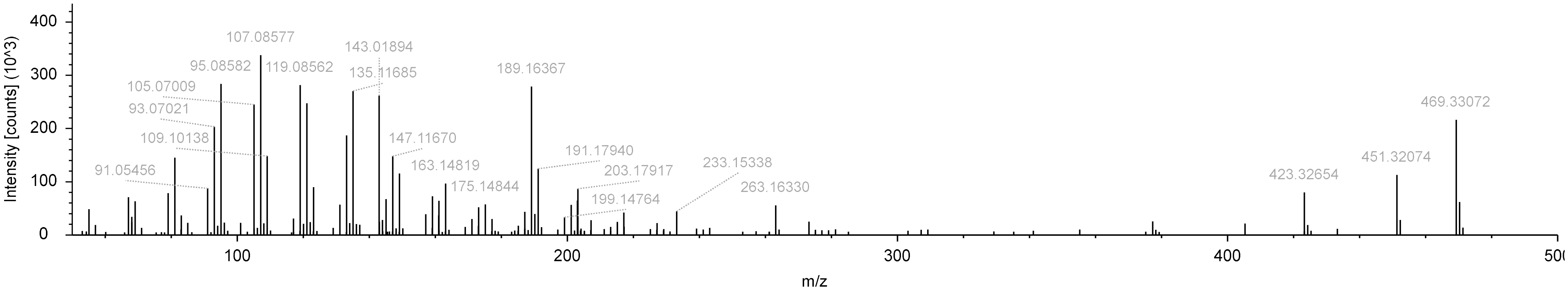

© Reported with Compound Discoverer 3.2

Structure	Name	RT [min]	Formula	Molecular Weight	Group Areas
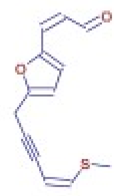	(2Z)-3-{5-[(4Z)-5-(Methylsulfanyl)-4-penten-2-yn-1-yl]-2-furyl}acrylaldehyde	1.16	C13 H12 O2 S	232.0558	3.35e8

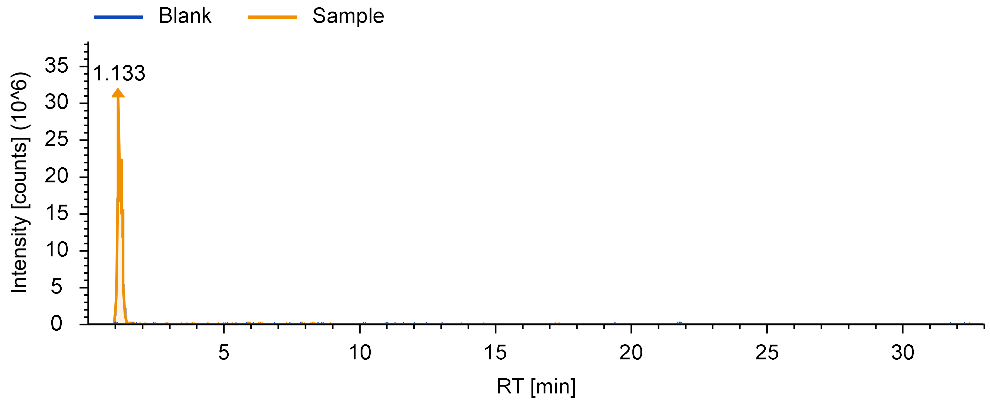

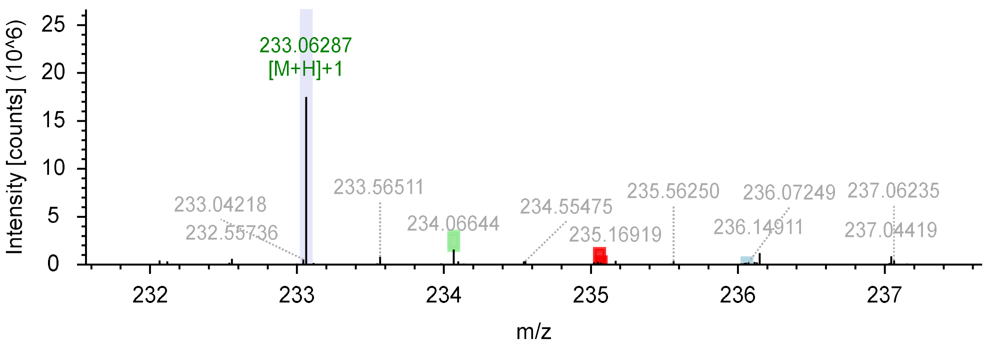

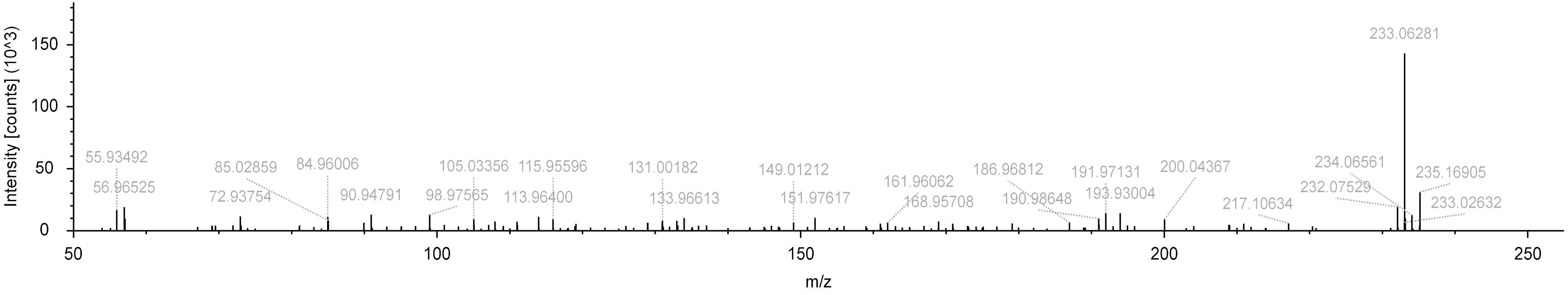

© Reported with Compound Discoverer 3.2

Compounds

Unit Laboratorium Riset Unggulan IPB

Structure	Name	RT [min]	Formula	Molecular Weight	Group Areas
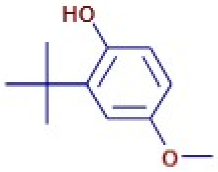	3-BHA	13.97	C11 H16 O2	180.1150	3.29e8

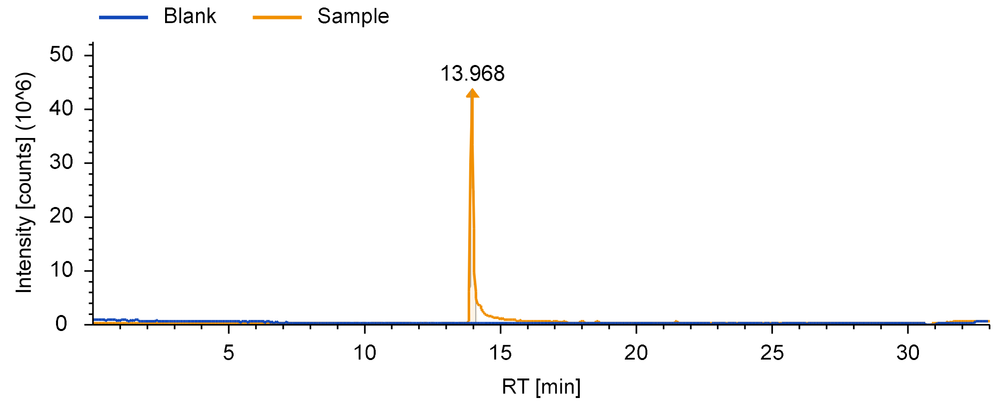

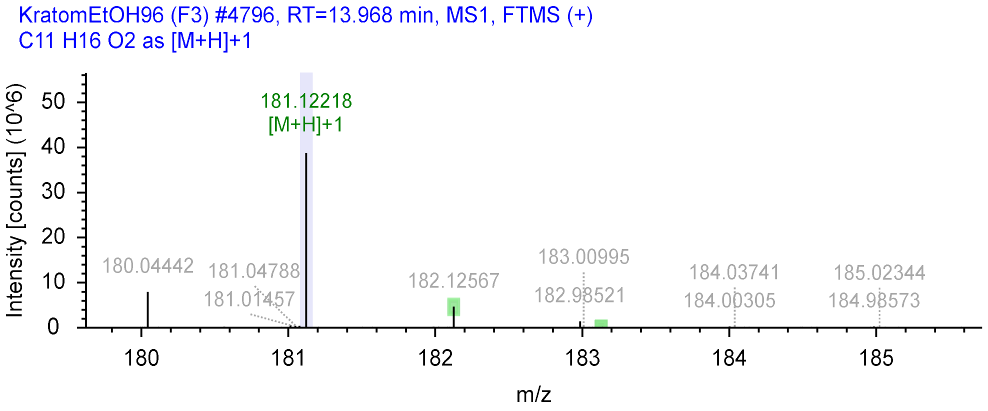

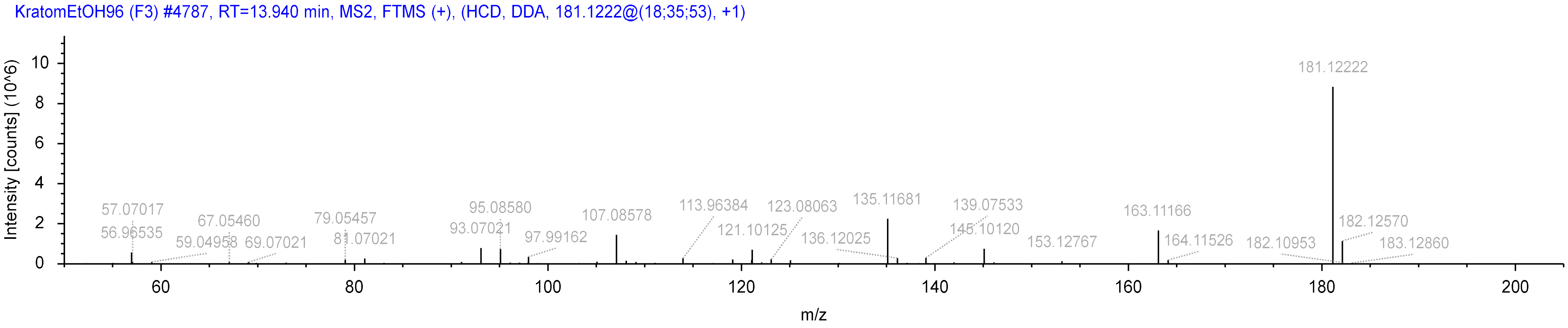

© Reported with Compound Discoverer 3.2

Compounds

Unit Laboratorium Riset Unggulan IPB

Structure	Name	RT [min]	Formula	Molecular Weight	Group Areas
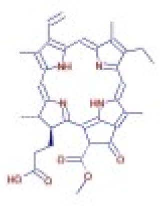	Pheophorbide A	27.24	C35 H36 N4 O5	592.2686	3.29e8

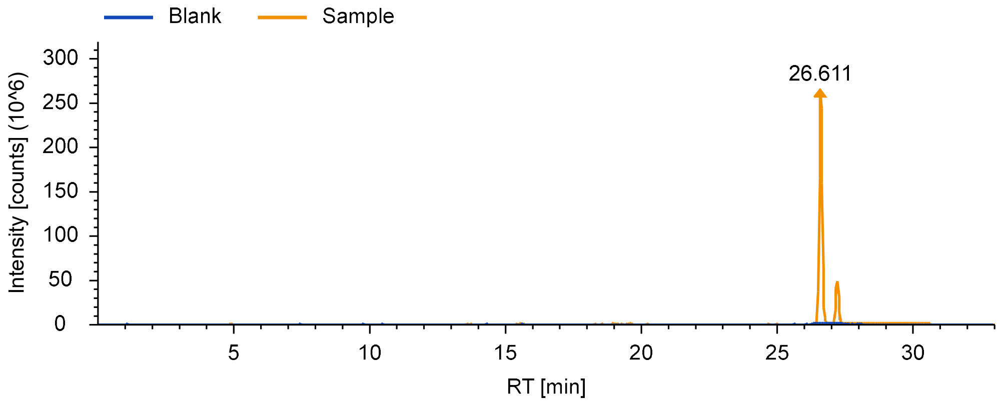

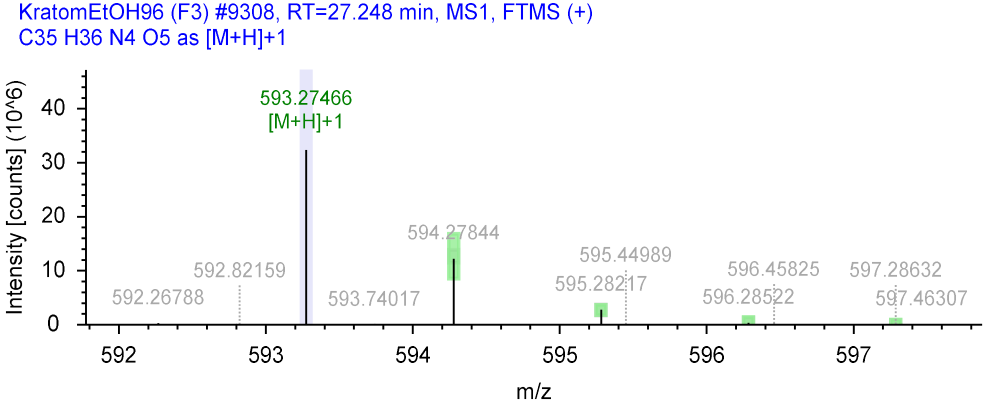

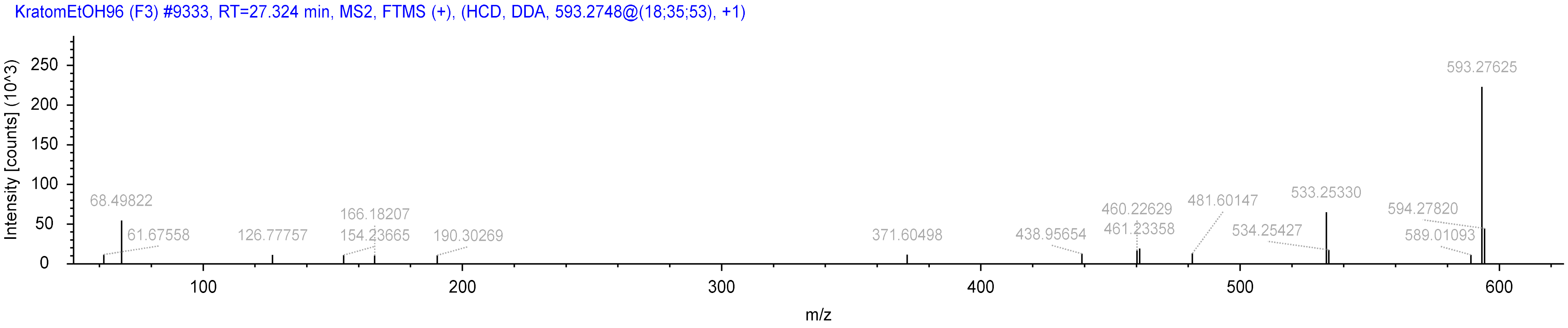

© Reported with Compound Discoverer 3.2

Structure	Name	RT [min]	Formula	Molecular Weight	Group Areas
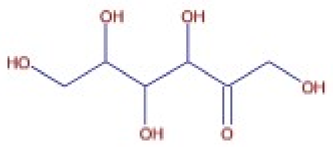	Hex-2-ulose	1.07	C6 H12 O6	180.0634	3.28e8

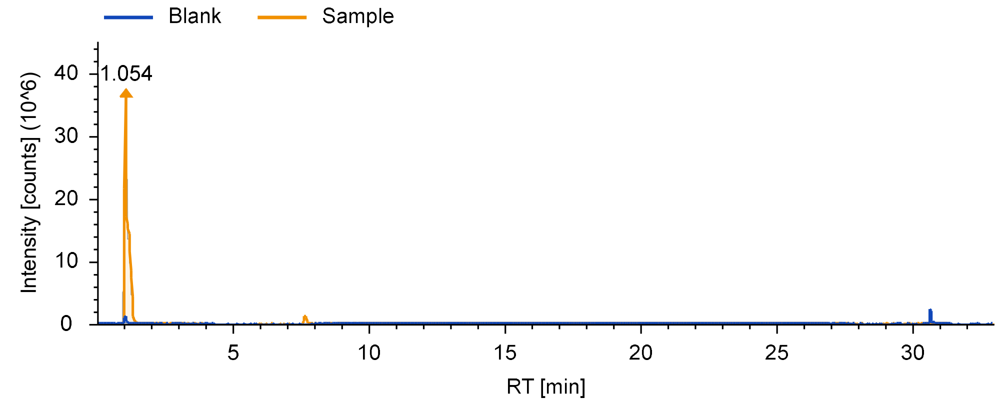

KratomEtOH96 (F3) #362, RT=1.054 min, MS1, FTMS (-)
C6 H12 O6 as [M-H]-1

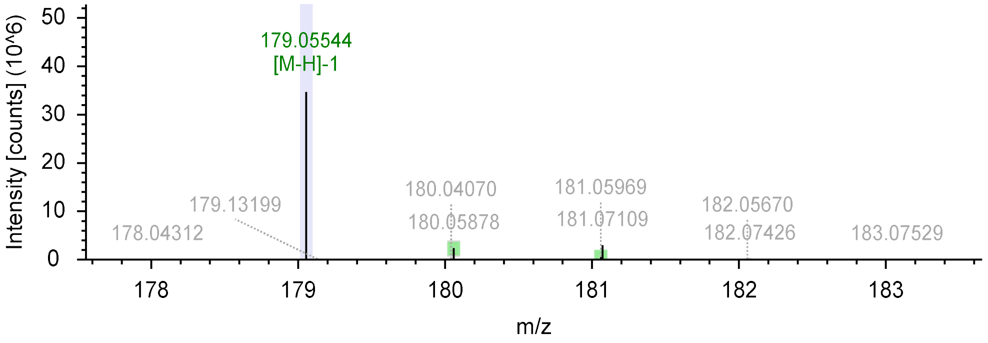

KratomEtOH96 (F3) #353, RT=1.030 min, MS2, FTMS (-), (HCD, DDA, 179.0555@(18;35;53), -1)

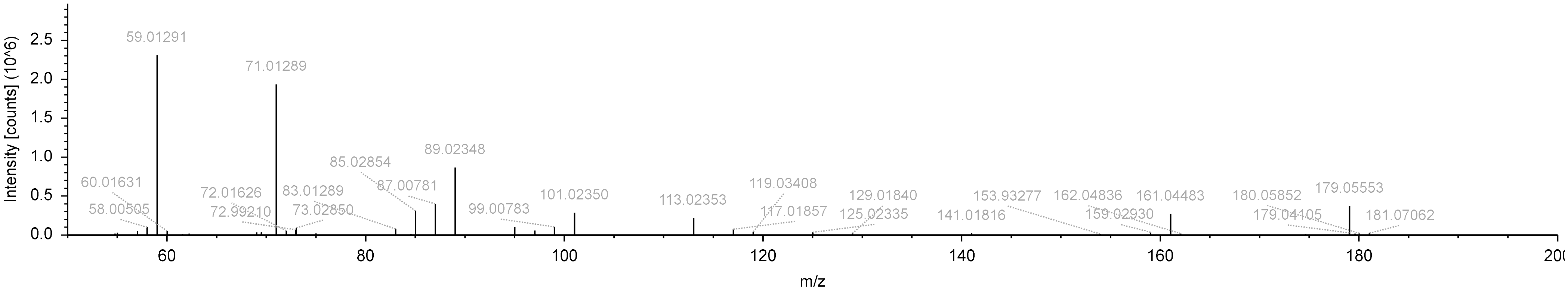

© Reported with Compound Discoverer 3.2

Structure	Name	RT [min]	Formula	Molecular Weight	Group Areas
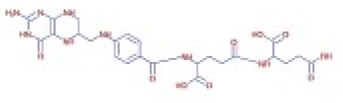	N-(4-{[(2-Amino-4-oxo-1,4,5,6,7,8-hexahydro-6-pteridinyl)methyl]amino}benzoyl)-gamma-glutamylglutamic acid	8.76	C24 H30 N8 O9	574.2136	3.20e8

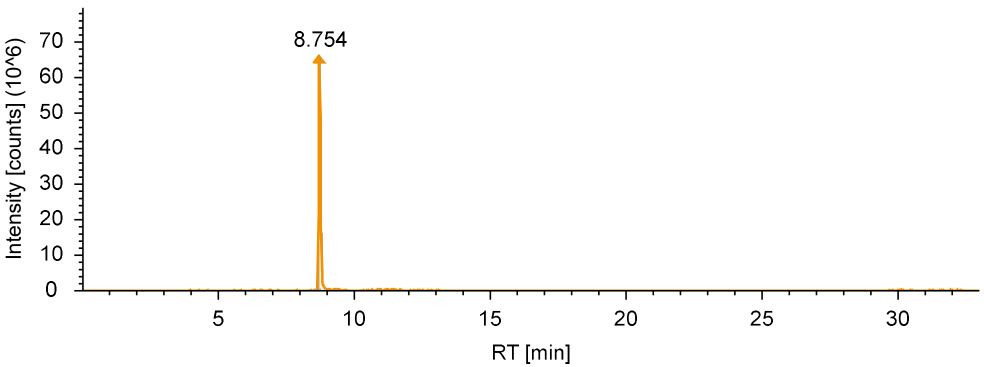

KratomEtOH96 (F3) #3002, RT=8.754 min, MS1, FTMS (-)
C24 H30 N8 O9 as [M-H]-1

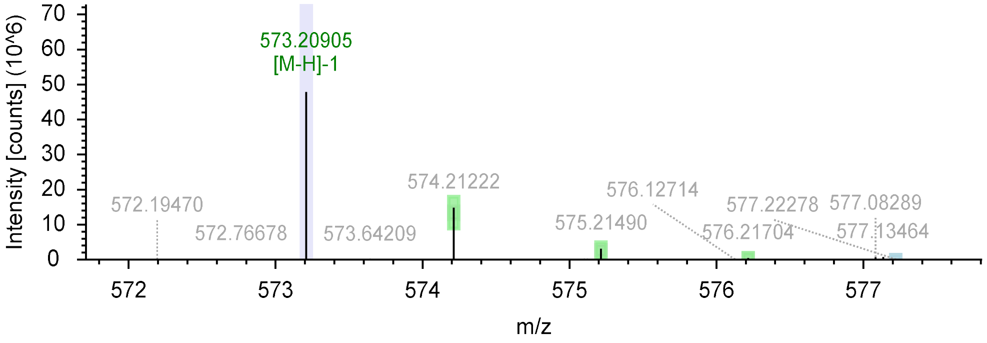

KratomEtOH96 (F3) #2993, RT=8.729 min, MS2, FTMS (-), (HCD, DDA, 573.2092@(18;35;53), -1)

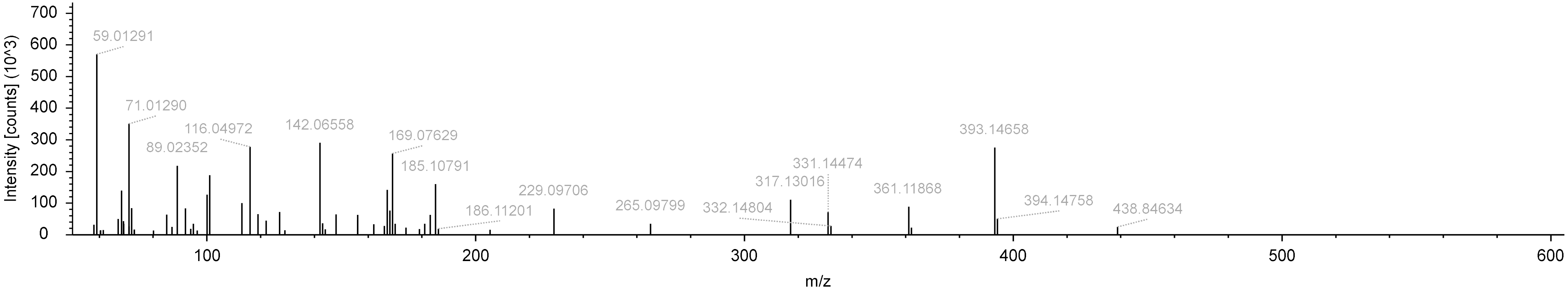

© Reported with Compound Discoverer 3.2

Compounds

Unit Laboratorium Riset Unggulan IPB

Structure	Name	RT [min]	Formula	Molecular Weight	Group Areas
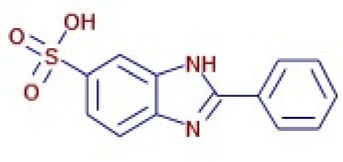	Ensulizole	6.00	C13 H10 N2 O3 S	274.0412	3.13e8

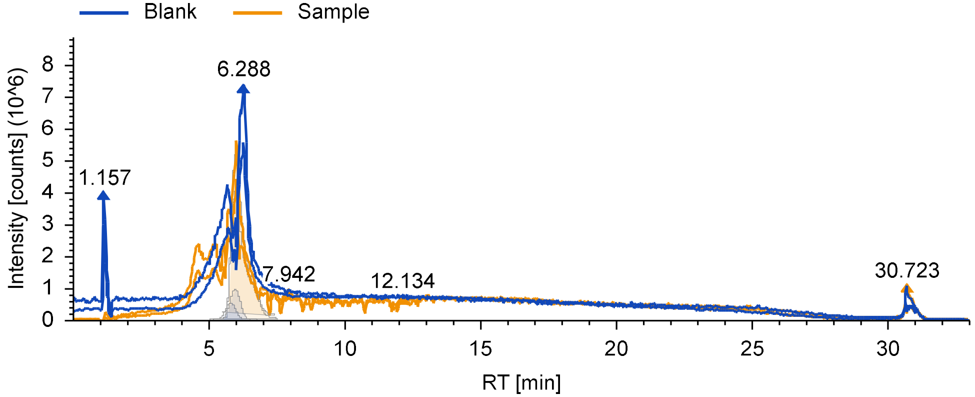

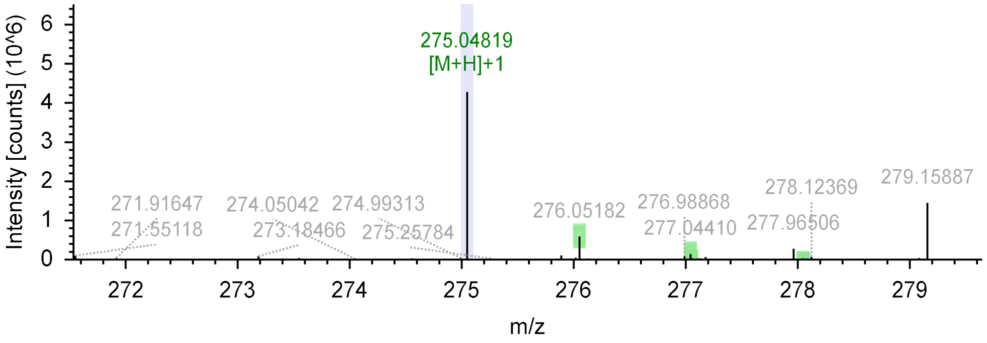

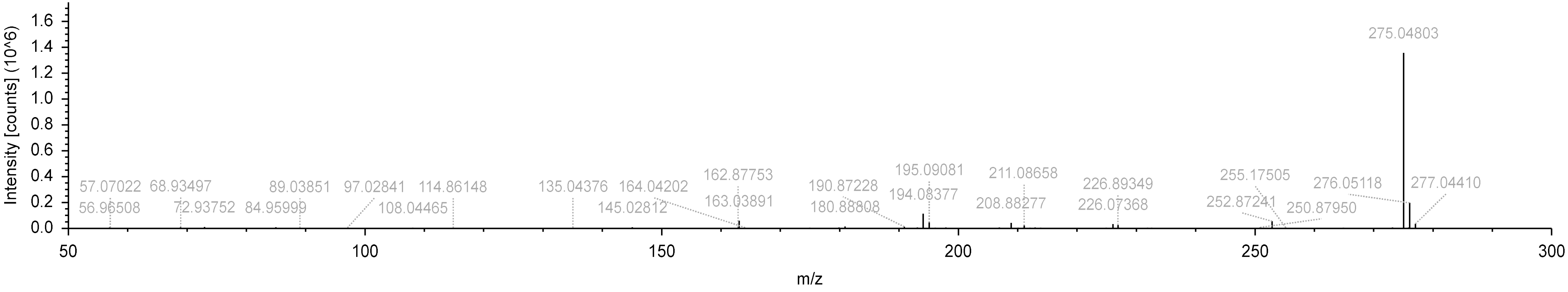

© Reported with Compound Discoverer 3.2

Structure	Name	RT [min]	Formula	Molecular Weight	Group Areas
	13-KODE	20.71	C18 H30 O3	294.2195	3.13e8

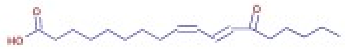

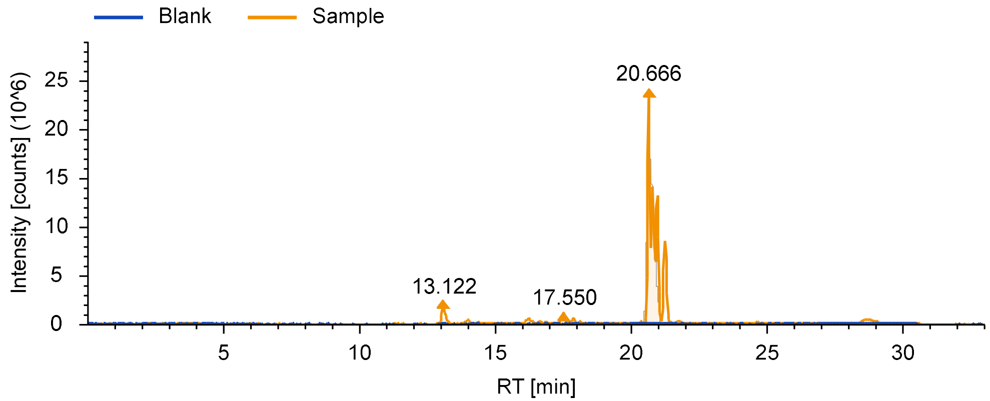

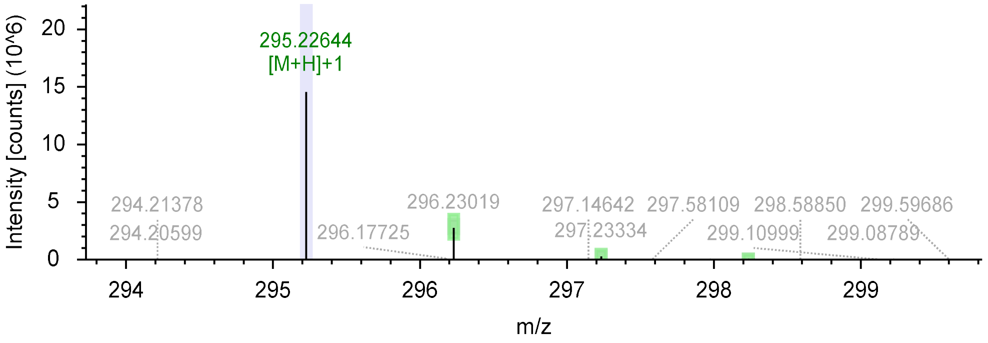

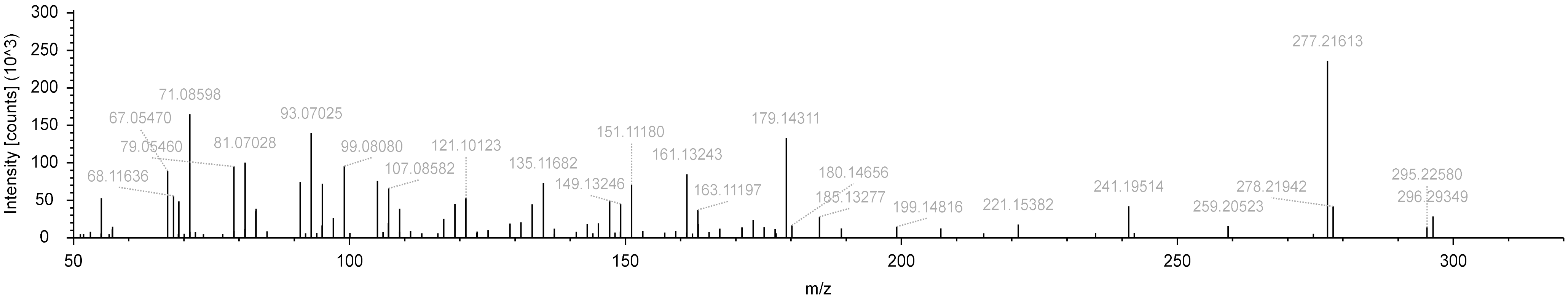

© Reported with Compound Discoverer 3.2

Compounds

Unit Laboratorium Riset Unggulan IPB

Structure	Name	RT [min]	Formula	Molecular Weight	Group Areas
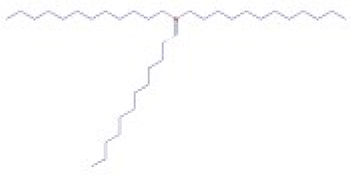	TRILAURYLAMINE	30.43	C36 H75 N	521.5900	2.90e8

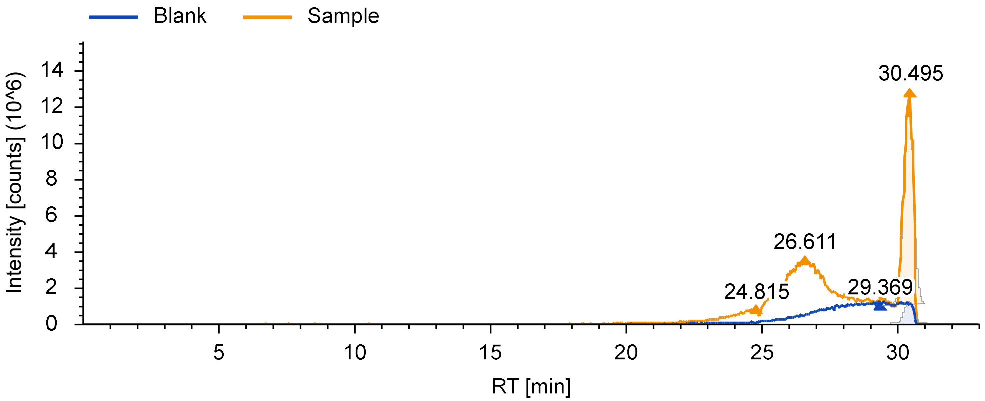

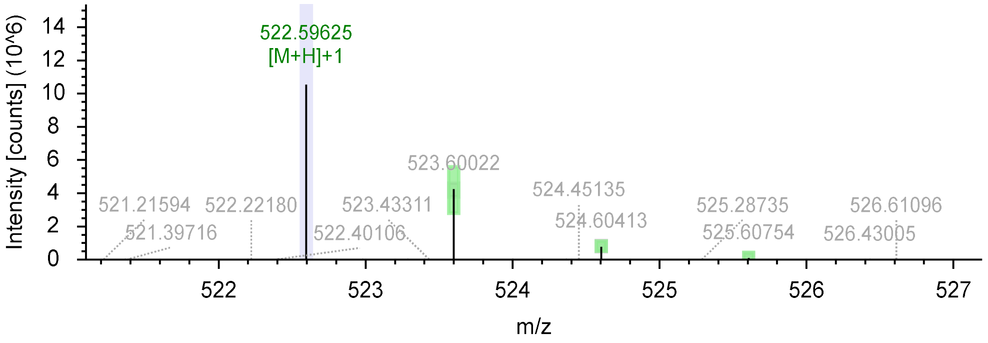

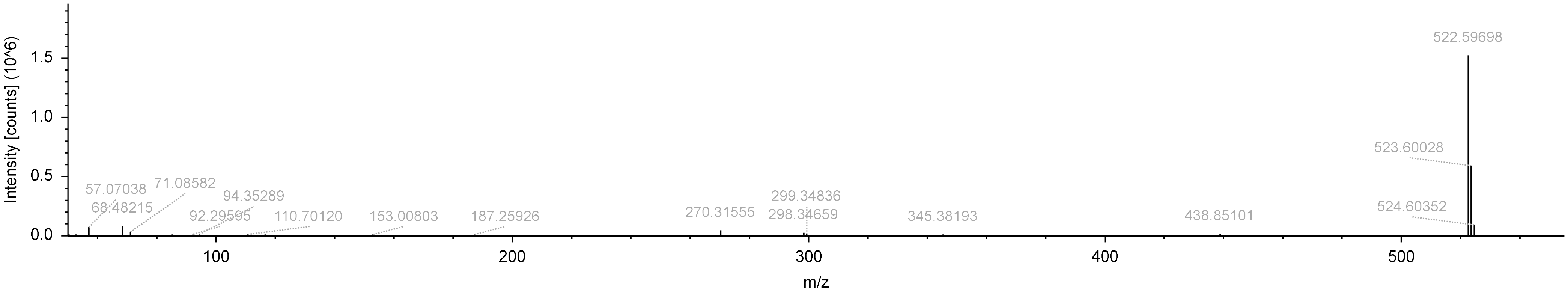

© Reported with Compound Discoverer 3.2

Compounds

Unit Laboratorium Riset Unggulan IPB

Structure	Name	RT [min]	Formula	Molecular Weight	Group Areas
		29.44	C38 H53 N3 O	567.4189	2.89e8

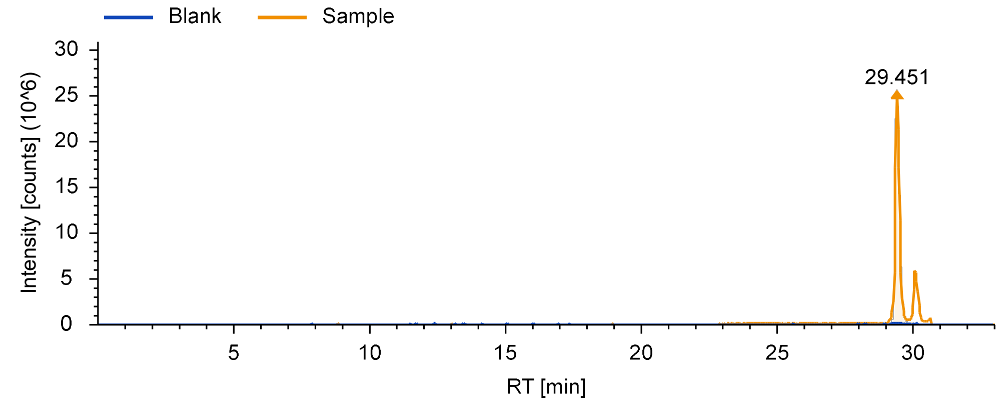

KratomEtOH96 (F3) #10052, RT=29.451 min, MS1, FTMS (+)
C38 H53 N3 O as [M+H]+1

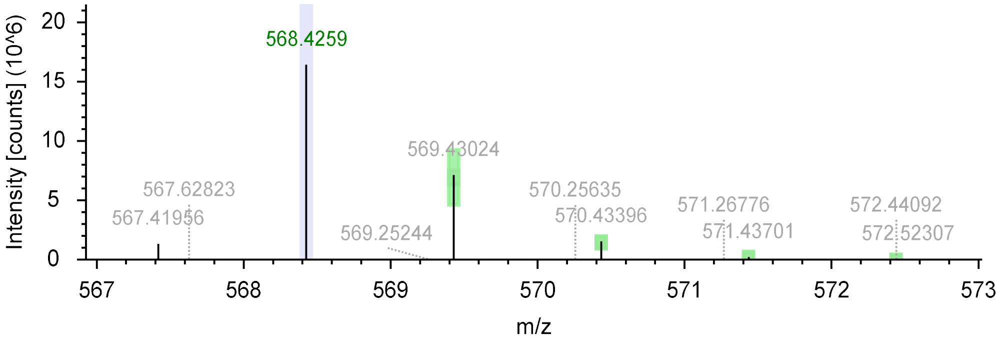

KratomEtOH96 (F3) #10053, RT=29.456 min, MS2, FTMS (+), (HCD, DDA, 568.4260@(18;35;53), +1)

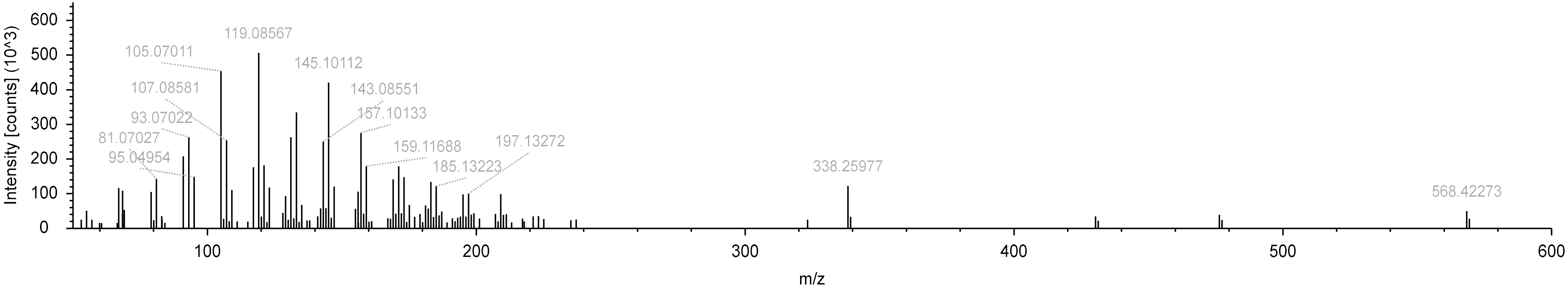

© Reported with Compound Discoverer 3.2

Structure	Name	RT [min]	Formula	Molecular Weight	Group Areas
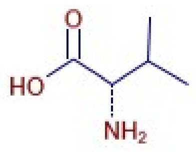	L-(+)-Valine	1.09	C5 H11 N O2	117.0790	2.80e8

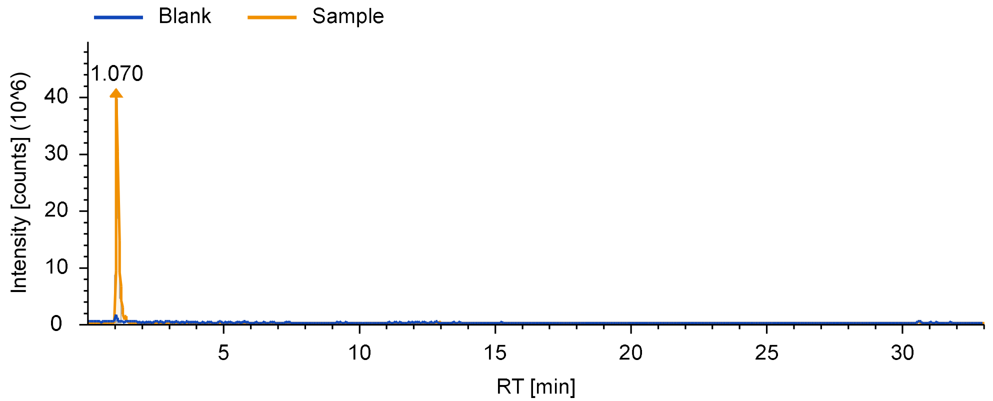

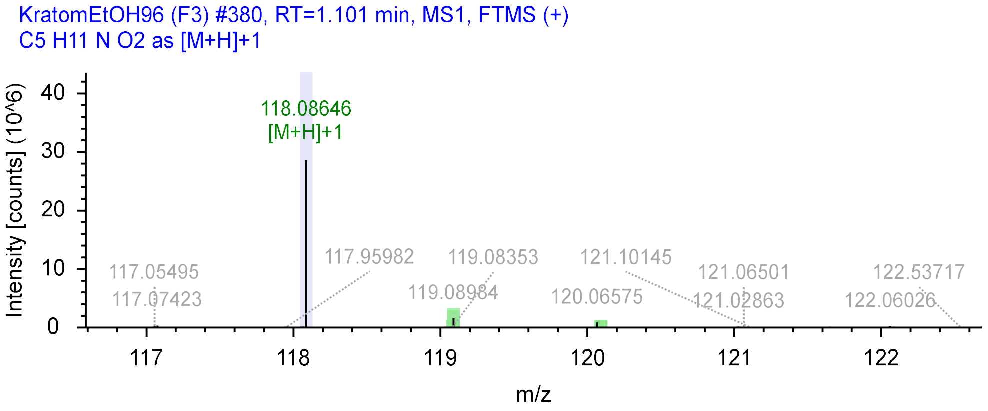

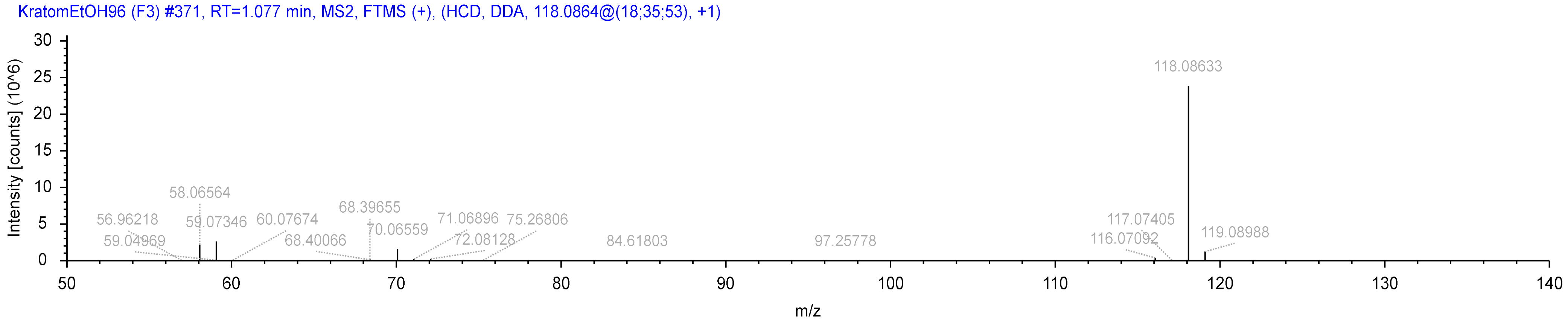

© Reported with Compound Discoverer 3.2

Compounds

Unit Laboratorium Riset Unggulan IPB

Structure	Name	RT [min]	Formula	Molecular Weight	Group Areas
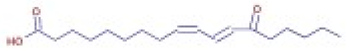	13-KODE	18.98	C18 H30 O3	294.2195	2.77e8

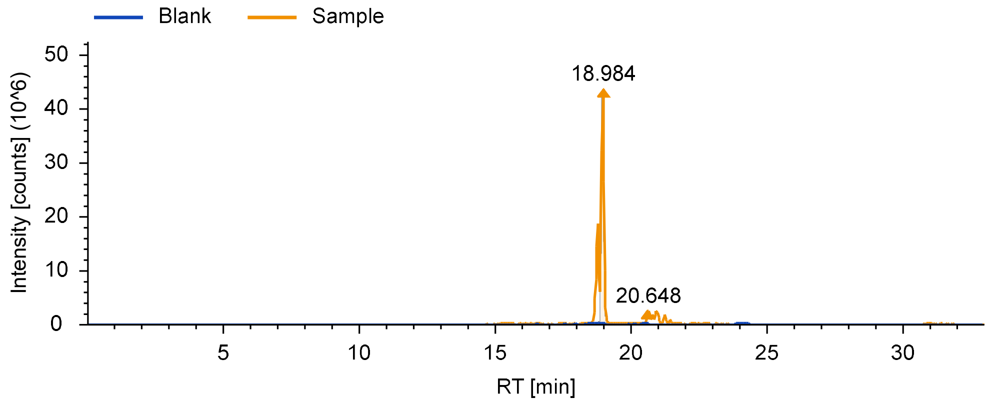

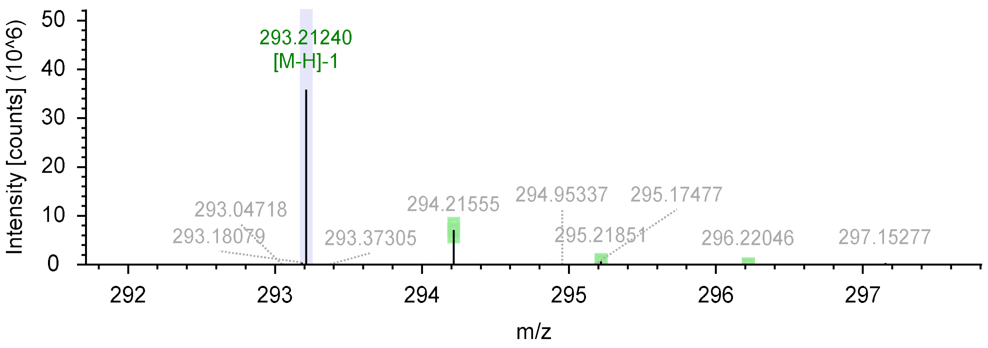

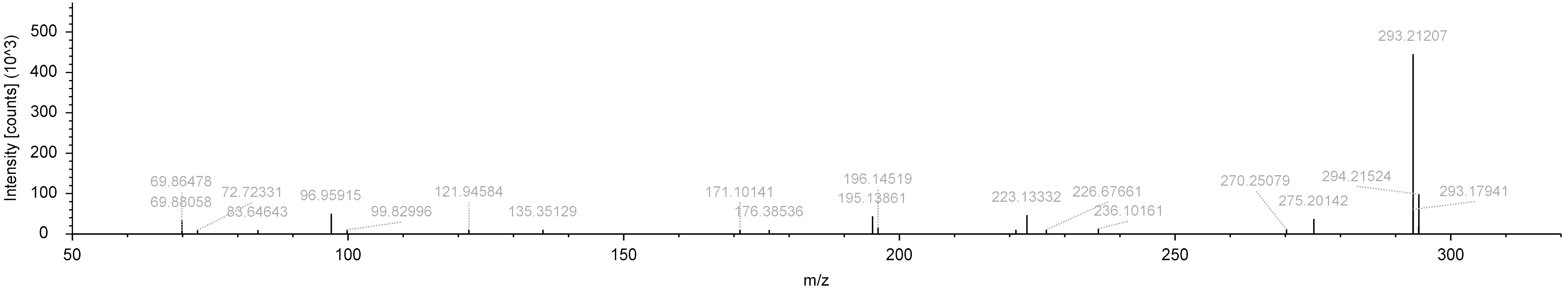

© Reported with Compound Discoverer 3.2

Structure	Name	RT [min]	Formula	Molecular Weight	Group Areas
		11.95	C41 H69 N4 O10 P S	840.4472	2.67e8

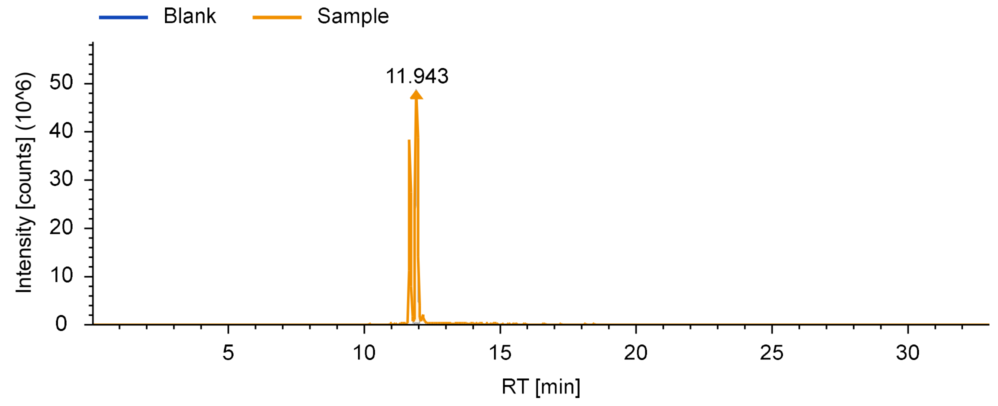

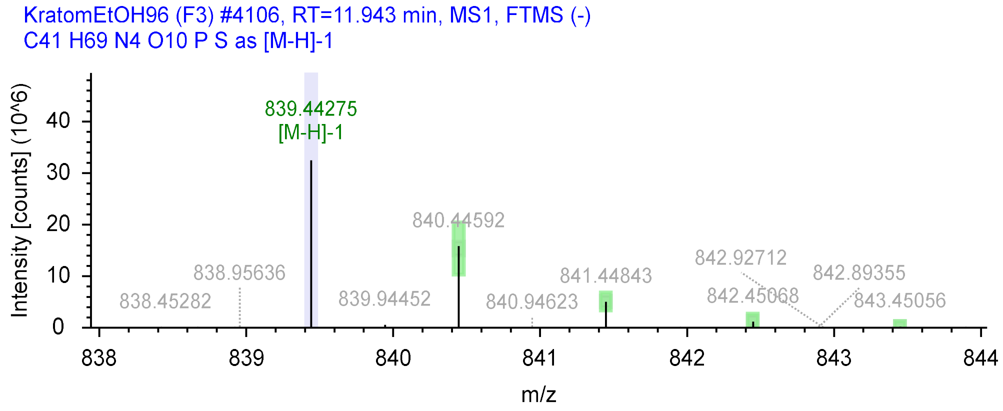

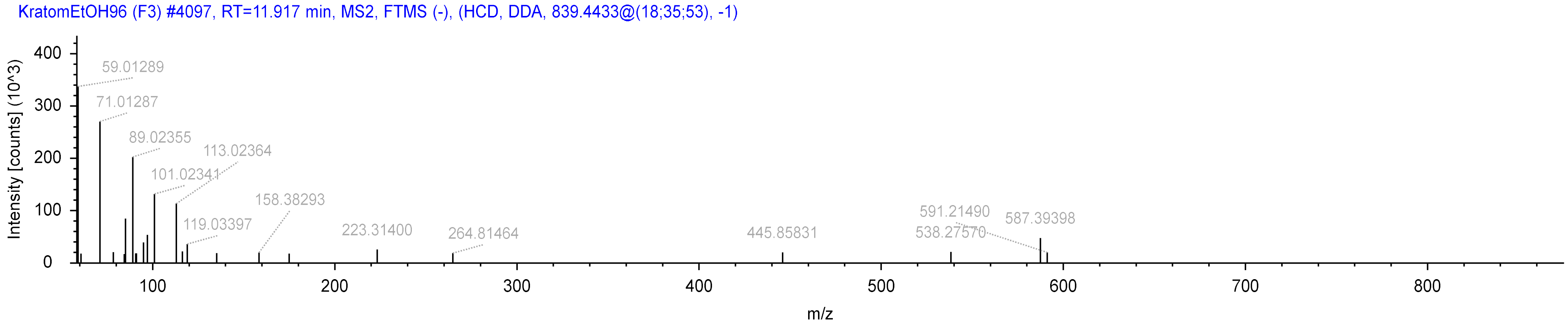

© Reported with Compound Discoverer 3.2

Structure	Name	RT [min]	Formula	Molecular Weight	Group Areas
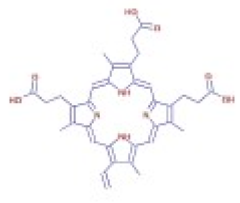	3,3',3''-(3,8,13,17-Tetramethyl-12-vinyl-2,7,18-porphyrintriyl)tripropanoic acid	26.08	C35 H36 N4 O6	608.2635	2.63e8

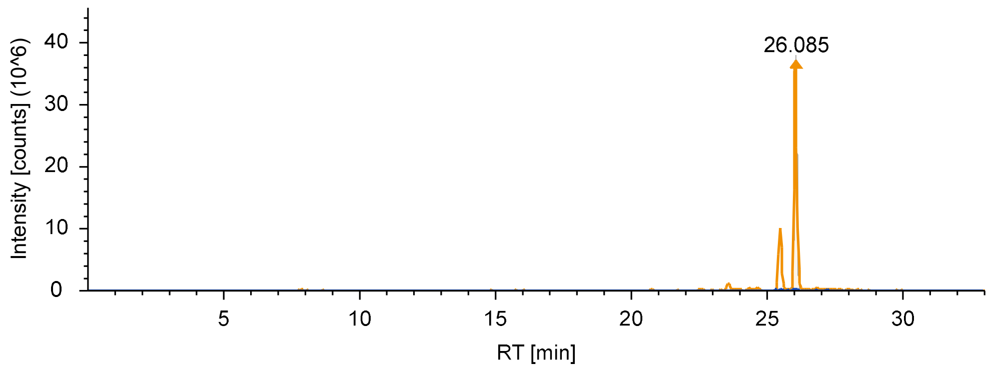

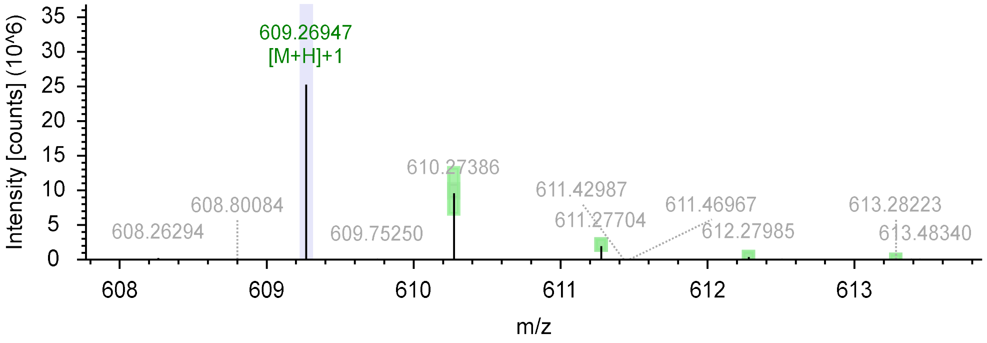

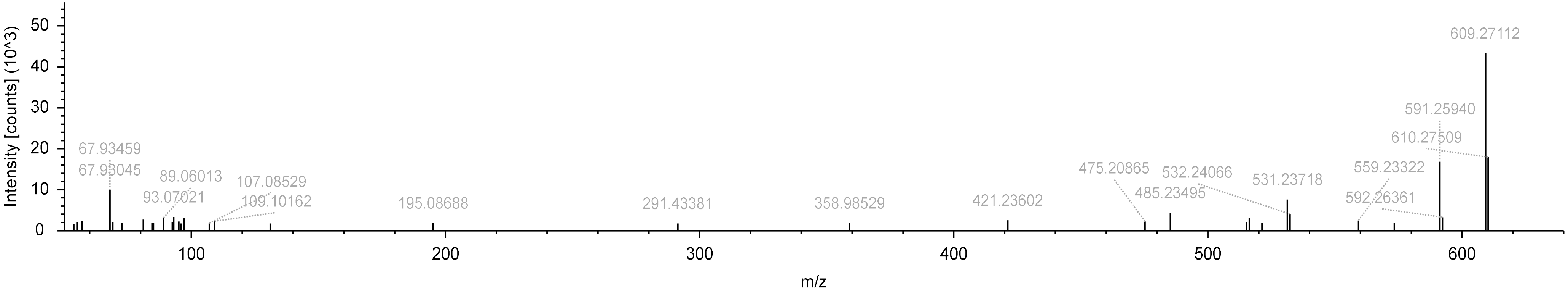

© Reported with Compound Discoverer 3.2

Structure	Name	RT [min]	Formula	Molecular Weight	Group Areas
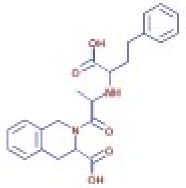	Quinaprilat	13.35	C23 H26 N2 O5	410.1842	2.63e8

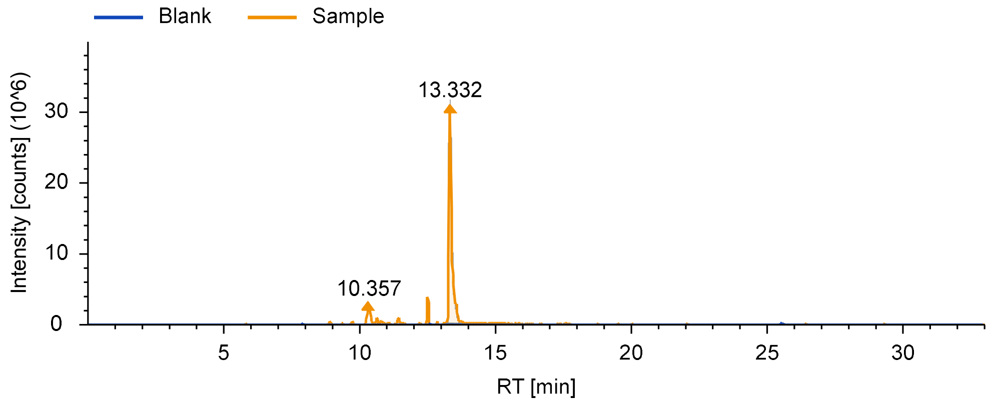

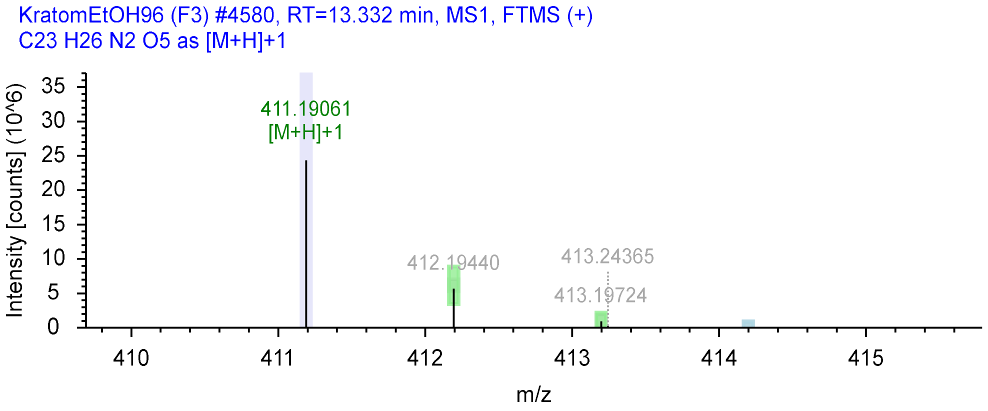

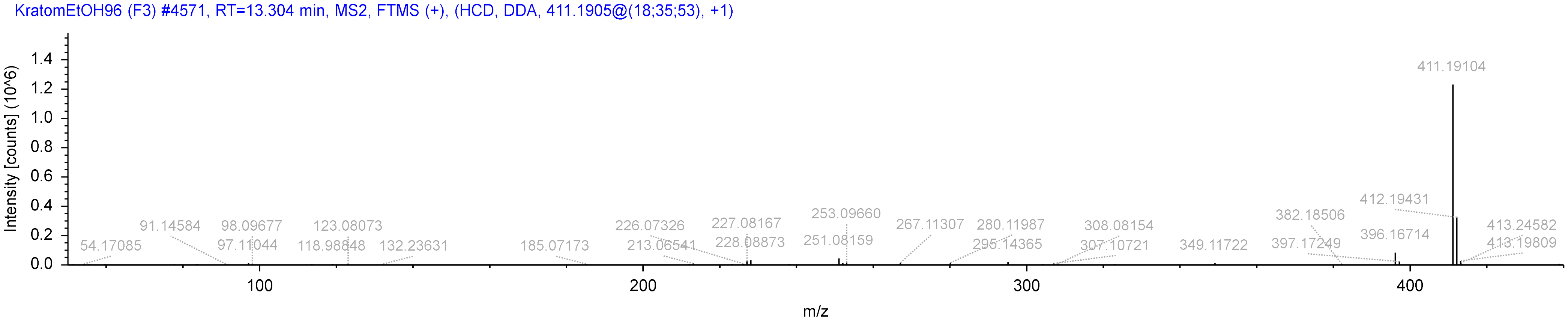

© Reported with Compound Discoverer 3.2

Structure	Name	RT [min]	Formula	Molecular Weight	Group Areas
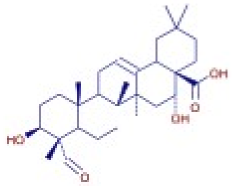	Quillaic Acid	10.76	C30 H46 O5	486.3345	2.54e8

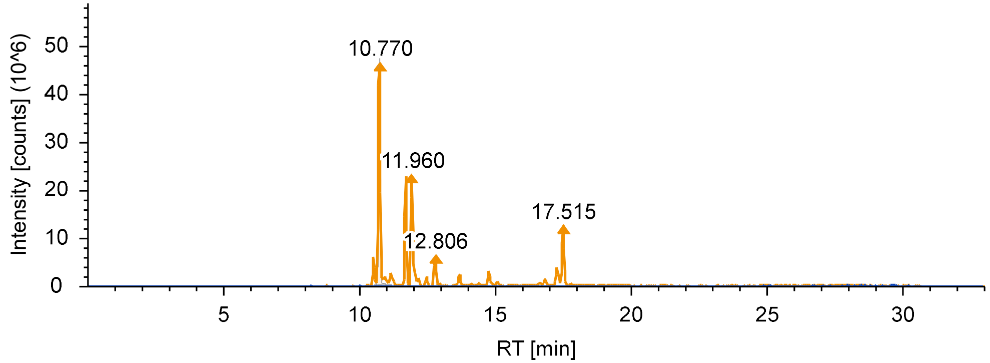

KratomEtOH96 (F3) #3704, RT=10.770 min, MS1, FTMS (+)
C30 H46 O5 as [M+H]+1

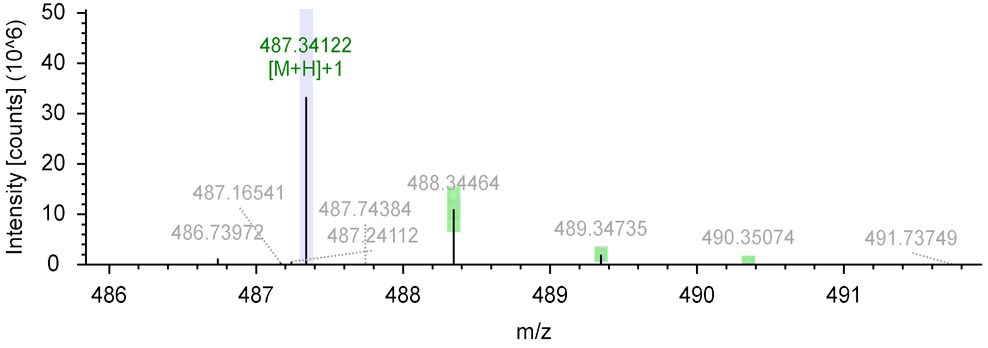

KratomEtOH96 (F3) #3694, RT=10.742 min, MS2, FTMS (+), (HCD, DDA, 487.3414@(18;35;53), +1)

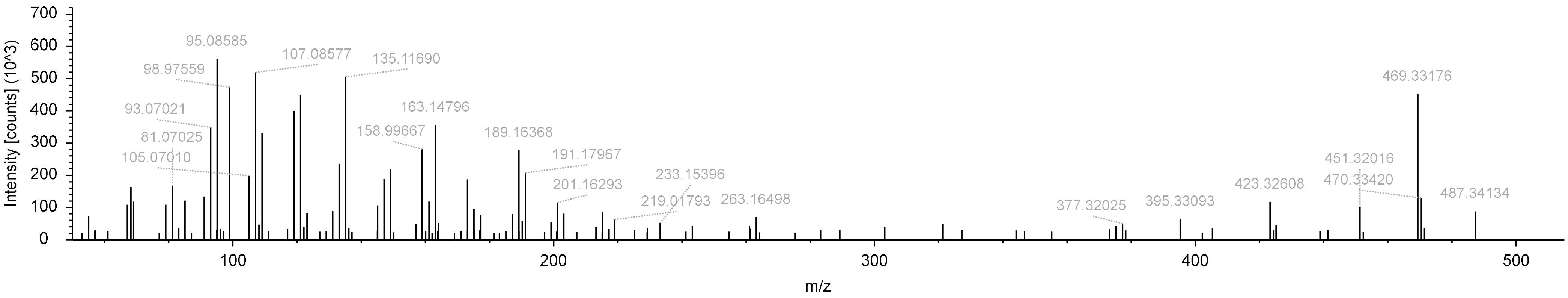

© Reported with Compound Discoverer 3.2

Compounds

Unit Laboratorium Riset Unggulan IPB

Structure	Name	RT [min]	Formula	Molecular Weight	Group Areas
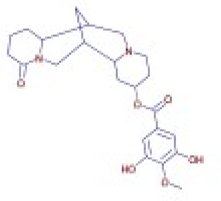	(7alpha,13beta)-2-Oxospartein-13-yl 3,5-dihydroxy-4-methoxybenzoate	11.71	C23 H30 N2 O6	430.2104	2.51e8

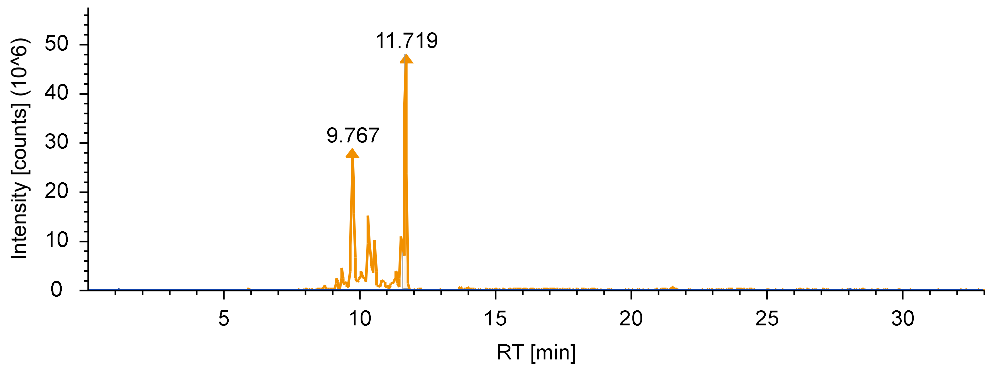

KratomEtOH96 (F3) #4028, RT=11.719 min, MS1, FTMS (+)
C23 H30 N2 O6 as [M+H]+1

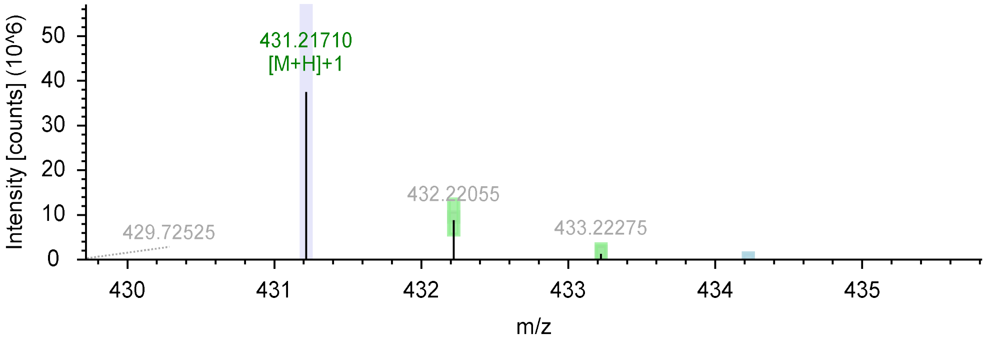

KratomEtOH96 (F3) #4041, RT=11.757 min, MS2, FTMS (+), (HCD, DDA, 431.2171@(18;35;53), +1)

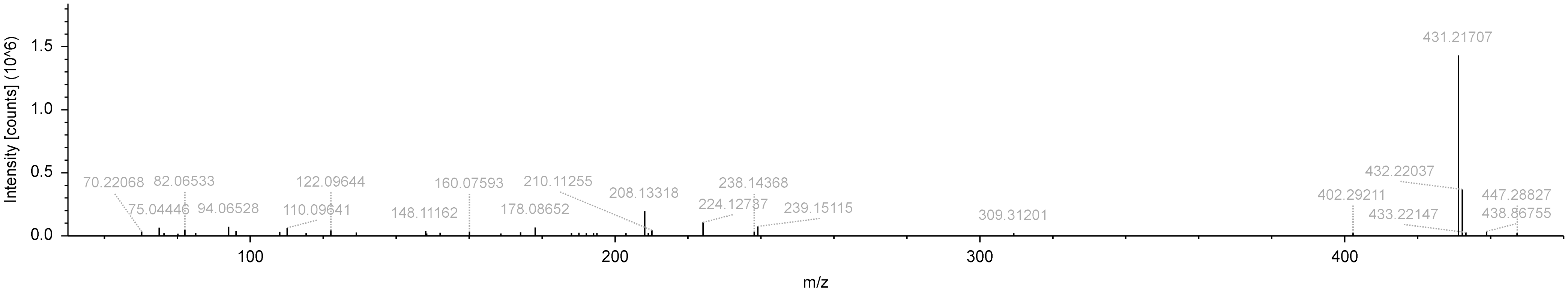

© Reported with Compound Discoverer 3.2

Compounds

Unit Laboratorium Riset Unggulan IPB

Structure	Name	RT [min]	Formula	Molecular Weight	Group Areas
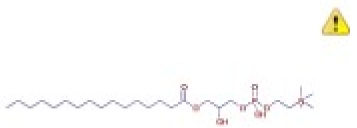	MFCD00036904	19.83	C24 H50 N O7 P	495.3325	2.42e8

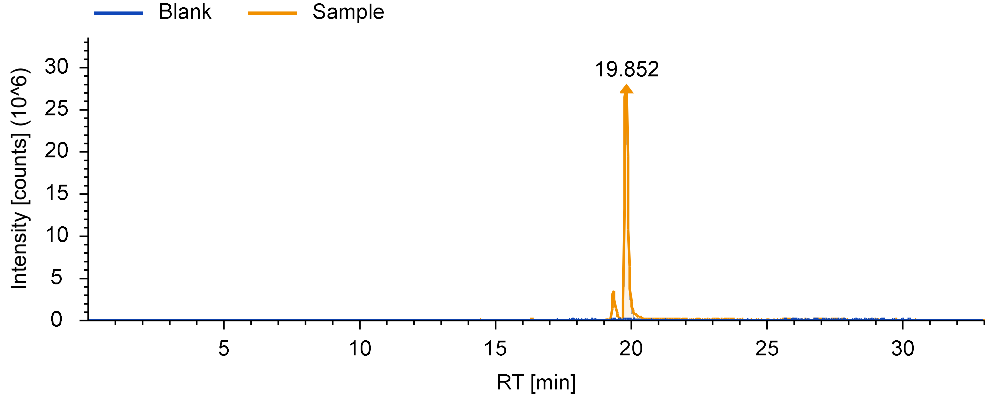

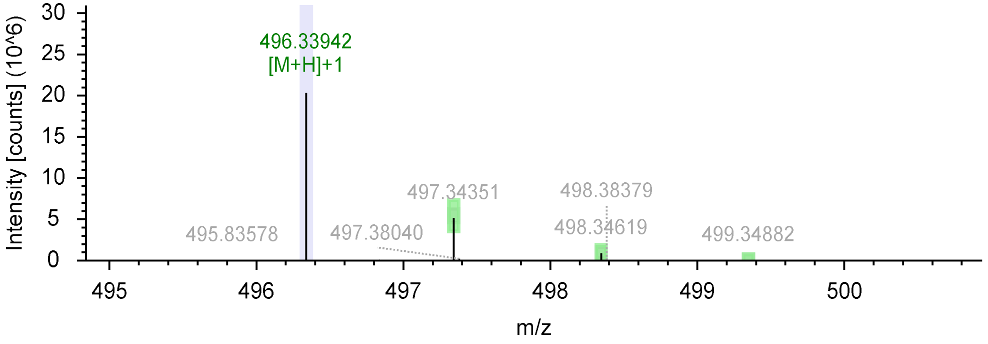

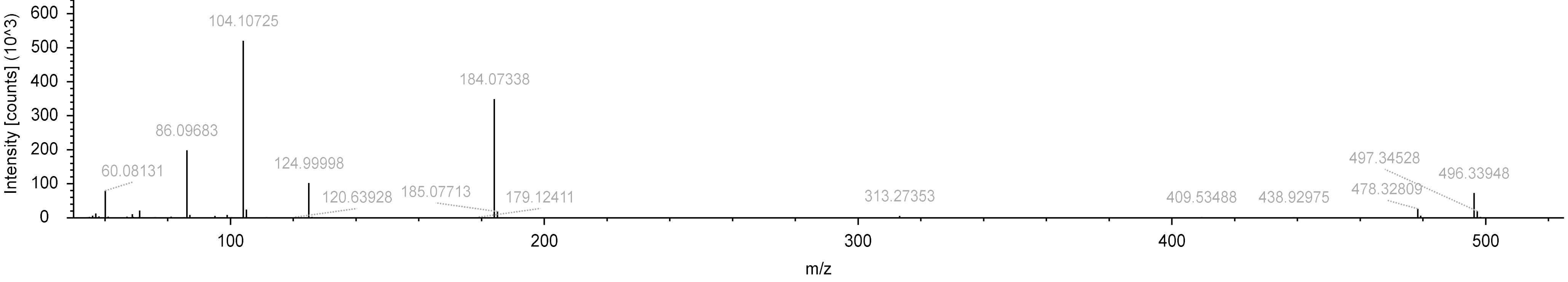

© Reported with Compound Discoverer 3.2

Compounds

Unit Laboratorium Riset Unggulan IPB

Structure	Name	RT [min]	Formula	Molecular Weight	Group Areas
		7.67	C15 H18 N6 O9	426.1135	2.40e8

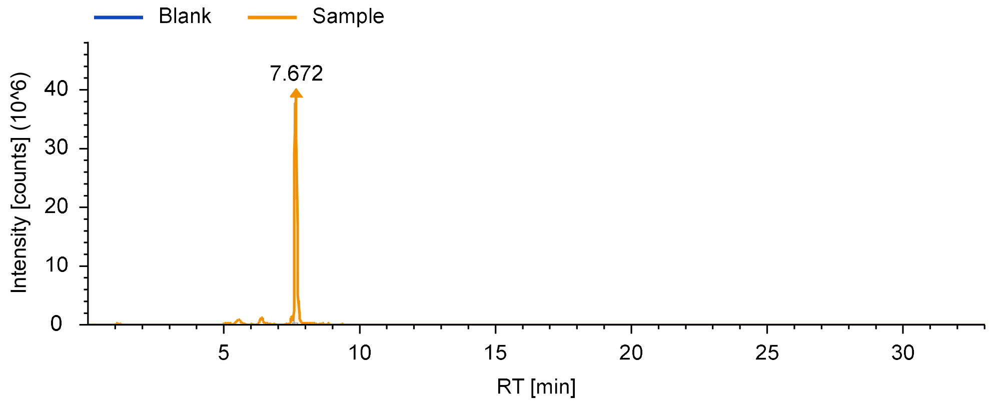

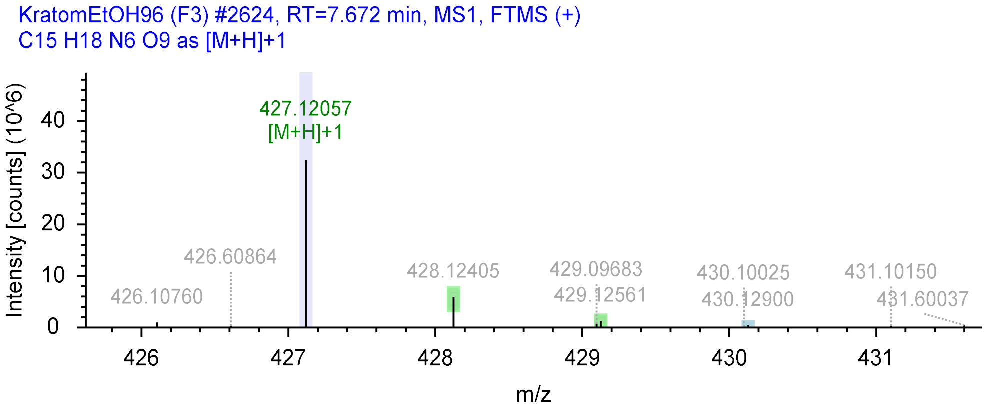

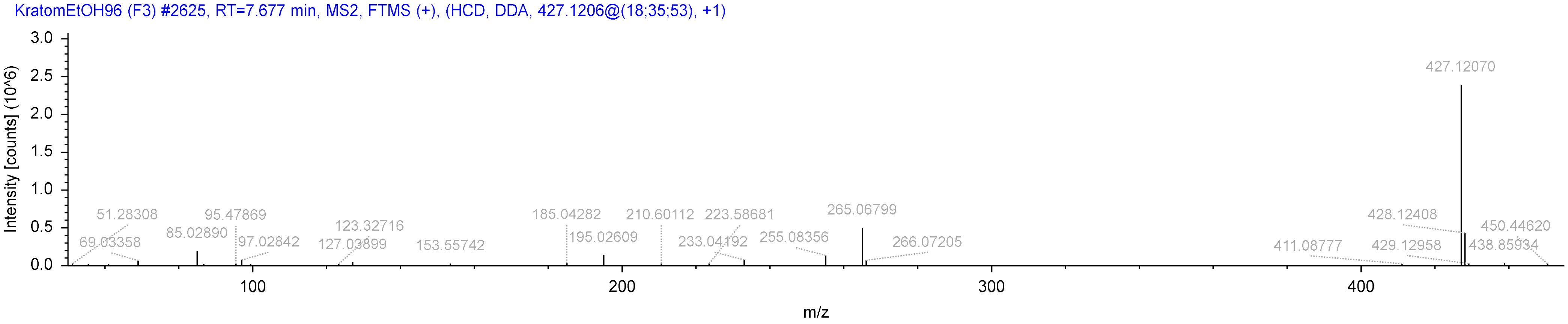

© Reported with Compound Discoverer 3.2

Compounds

Unit Laboratorium Riset Unggulan IPB

Structure	Name	RT [min]	Formula	Molecular Weight	Group Areas
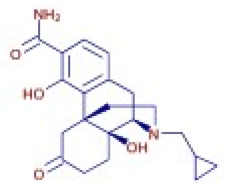	Samidorphan	8.79	C21 H26 N2 O4	370.1893	2.38e8

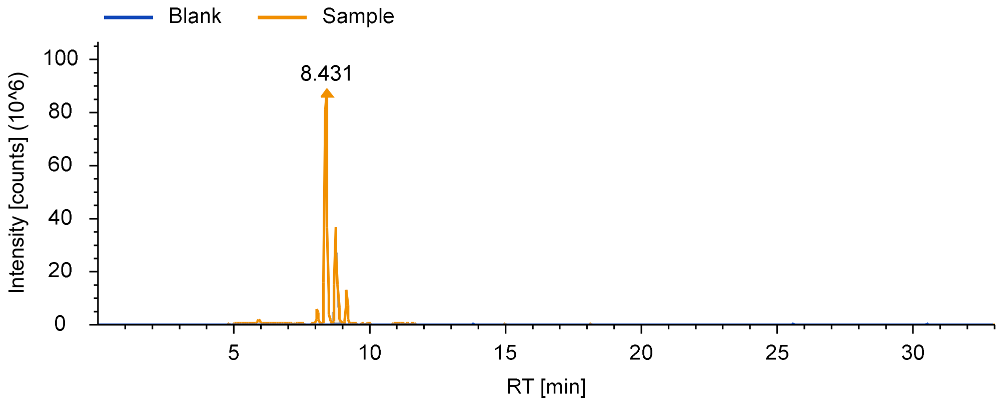

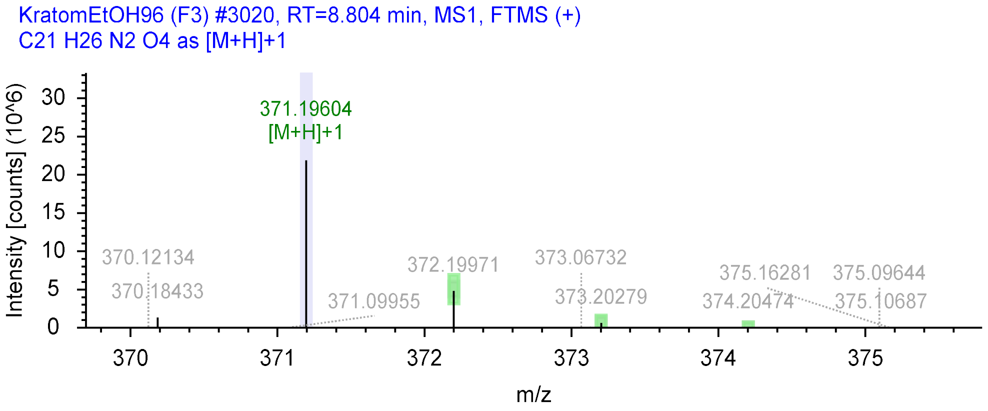

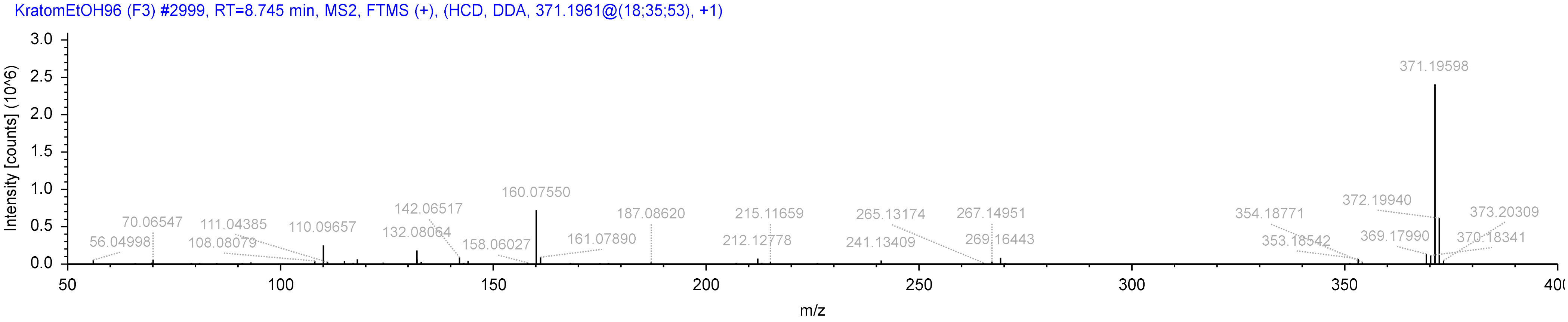

© Reported with Compound Discoverer 3.2

Compounds

Unit Laboratorium Riset Unggulan IPB

Structure	Name	RT [min]	Formula	Molecular Weight	Group Areas
		10.14	C37 H32 N4 O15	772.1864	2.37e8

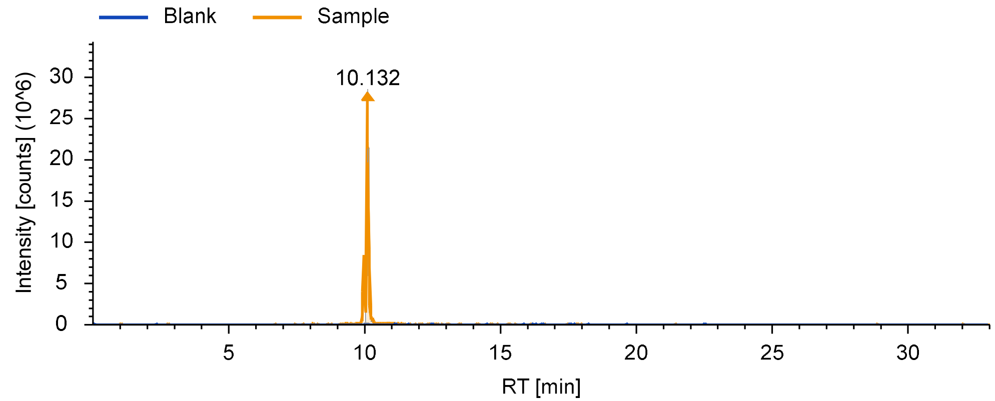

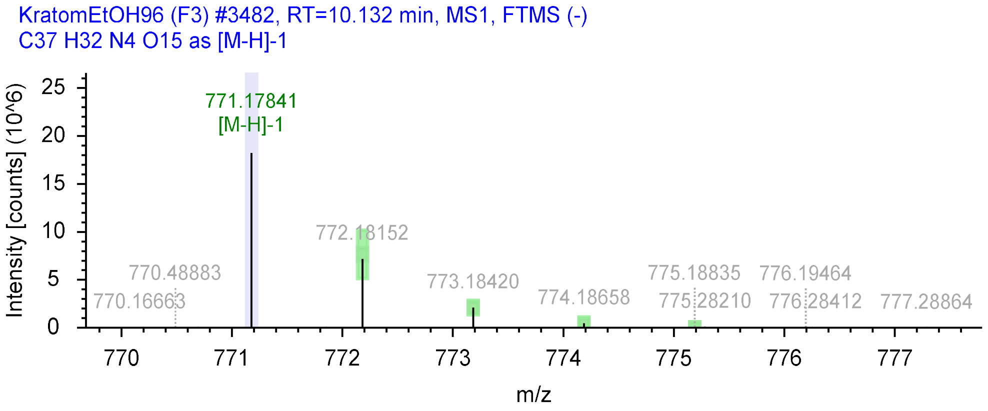

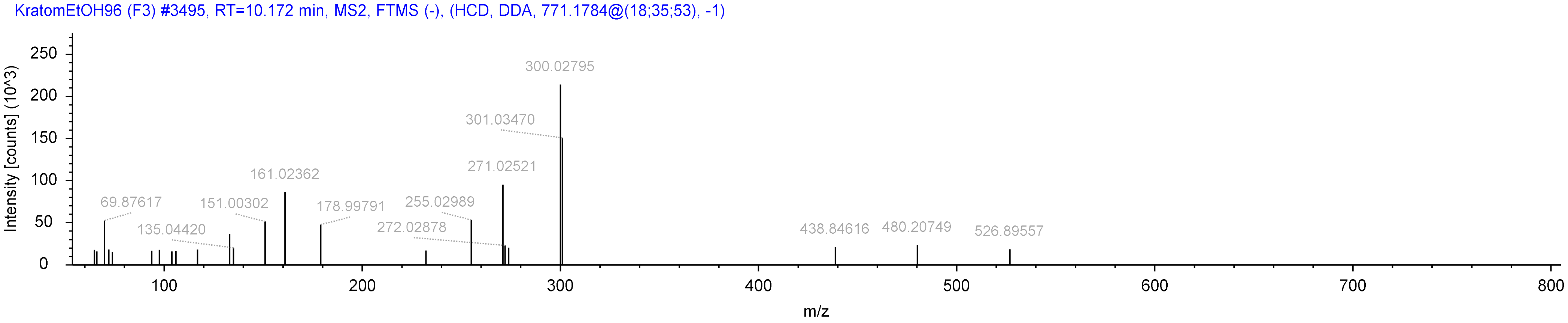

© Reported with Compound Discoverer 3.2

Compounds

Unit Laboratorium Riset Unggulan IPB

Structure	Name	RT [min]	Formula	Molecular Weight	Group Areas
		1.09	C13 H24 O12	372.1268	2.29e8

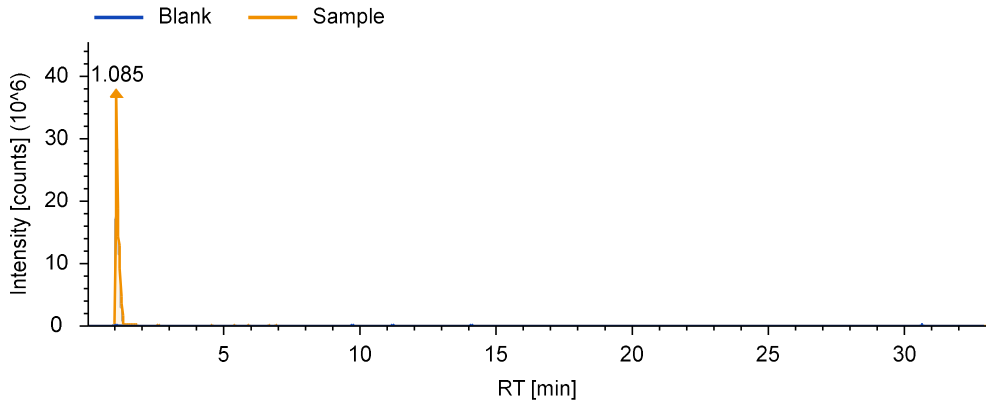

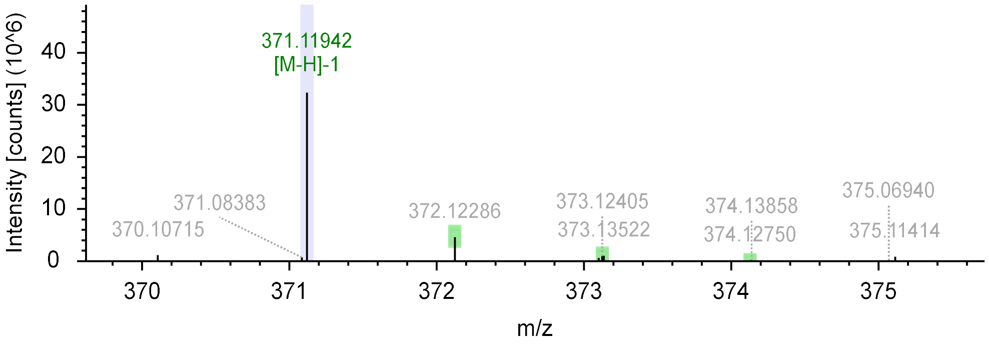

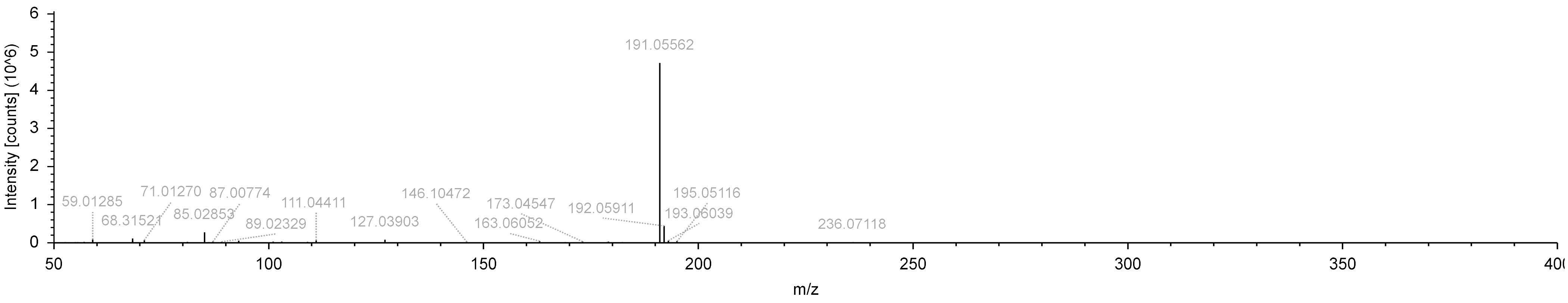

© Reported with Compound Discoverer 3.2

Compounds

Unit Laboratorium Riset Unggulan IPB

Structure	Name	RT [min]	Formula	Molecular Weight	Group Areas
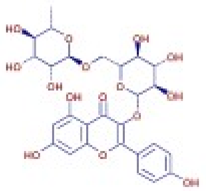	NICTOFLORIN	8.86	C27 H30 O15	594.1585	2.29e8

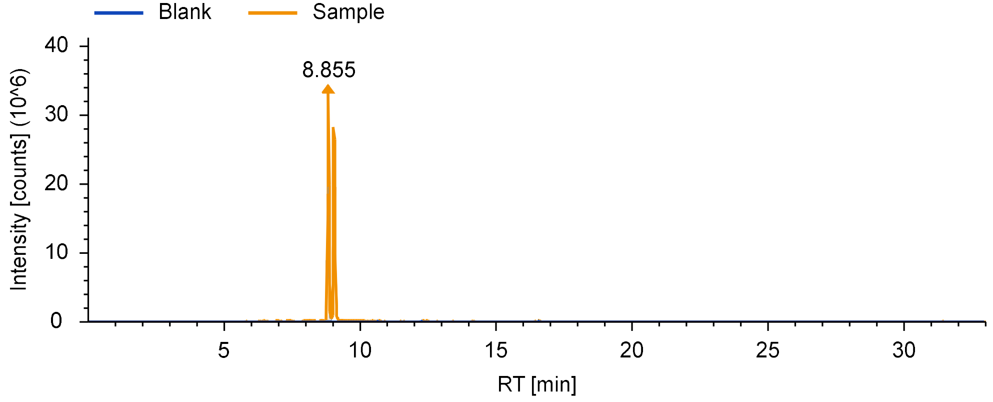

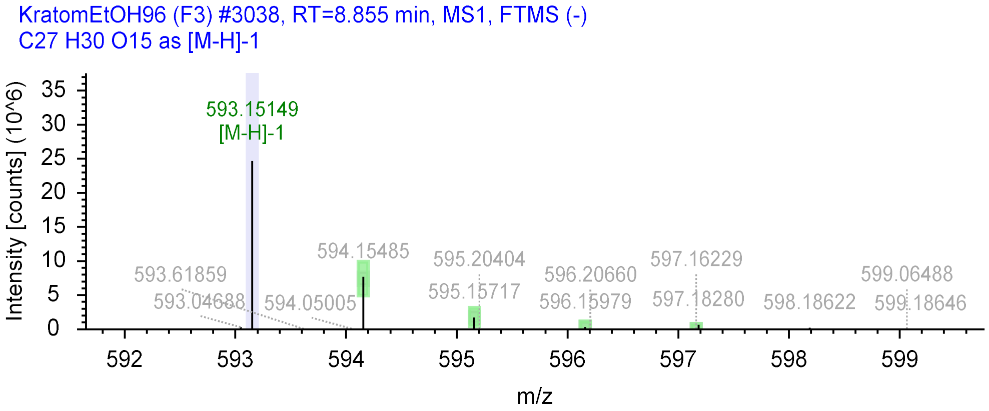

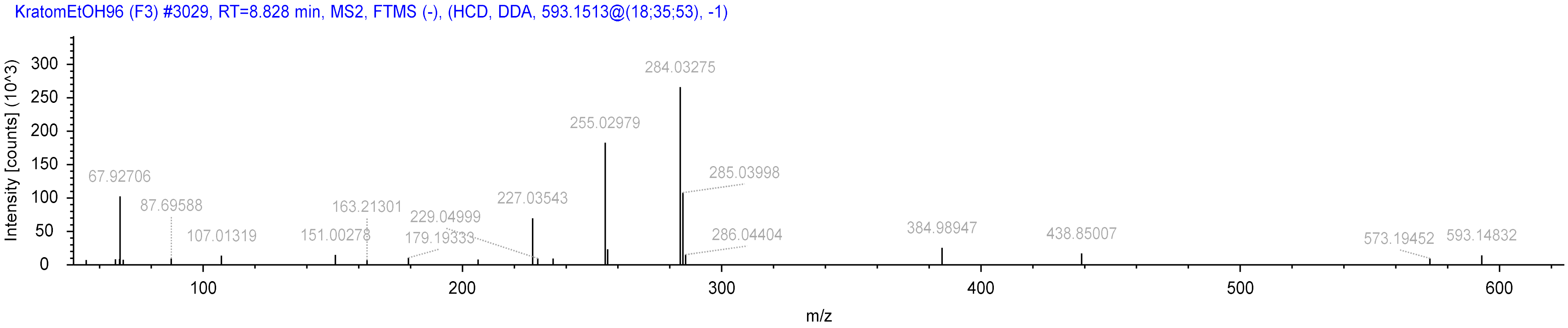

© Reported with Compound Discoverer 3.2

Compounds

Unit Laboratorium Riset Unggulan IPB

Structure	Name	RT [min]	Formula	Molecular Weight	Group Areas
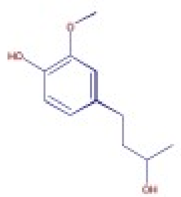	ZINGEROL	9.12	C11 H16 O3	196.1099	2.29e8

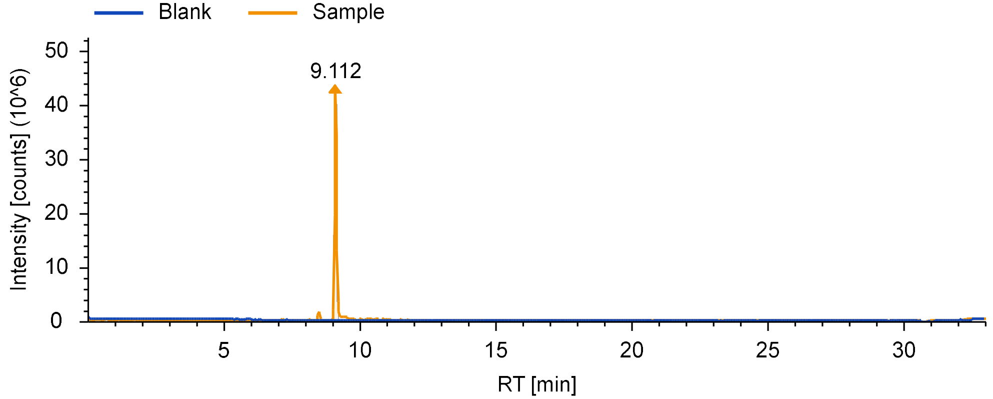

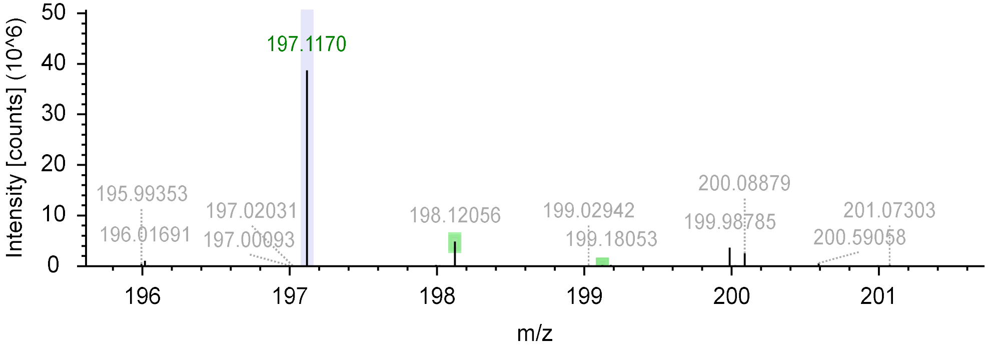

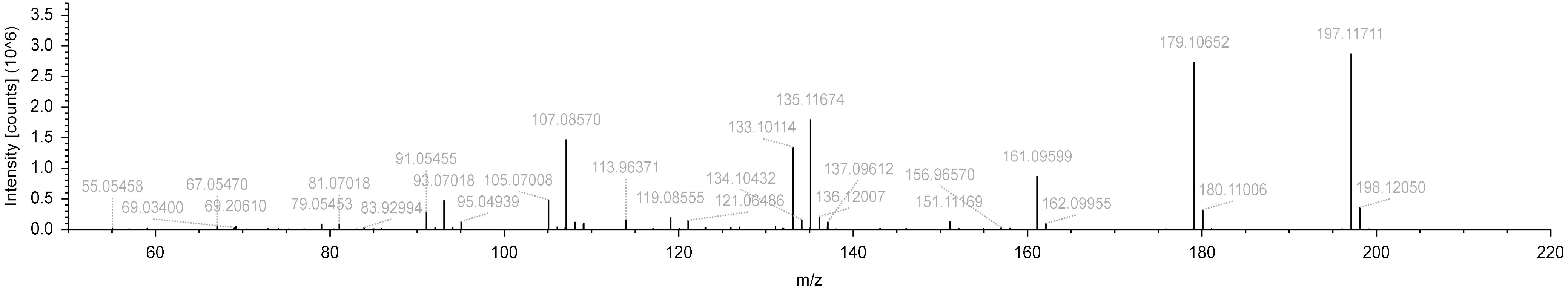

© Reported with Compound Discoverer 3.2

Compounds

Unit Laboratorium Riset Unggulan IPB

Structure	Name	RT [min]	Formula	Molecular Weight	Group Areas
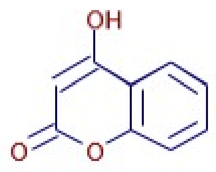	IN00458	6.13	C9 H6 O3	162.0317	2.27e8

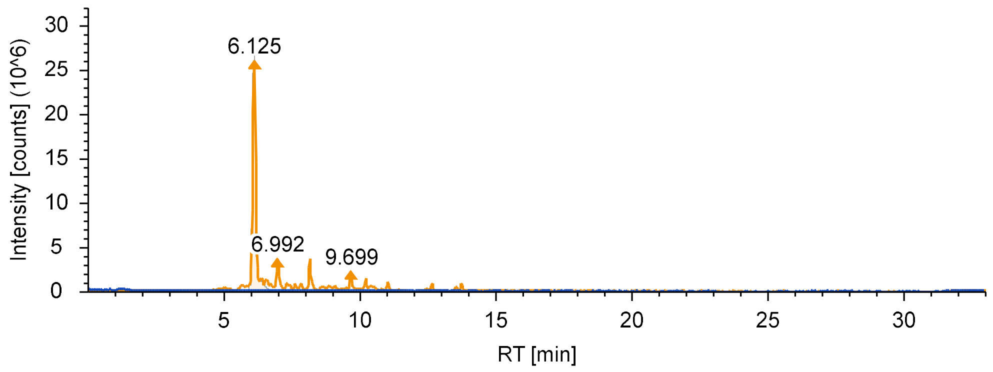

KratomEtOH96 (F3) #2084, RT=6.125 min, MS1, FTMS (+)
C9 H6 O3 as [M+H]+1

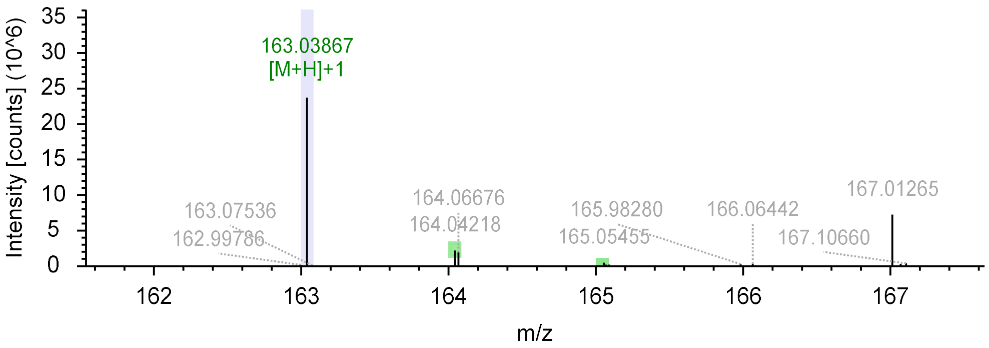

KratomEtOH96 (F3) #2063, RT=6.066 min, MS2, FTMS (+), (HCD, DDA, 163.0387@(18;35;53), +1)

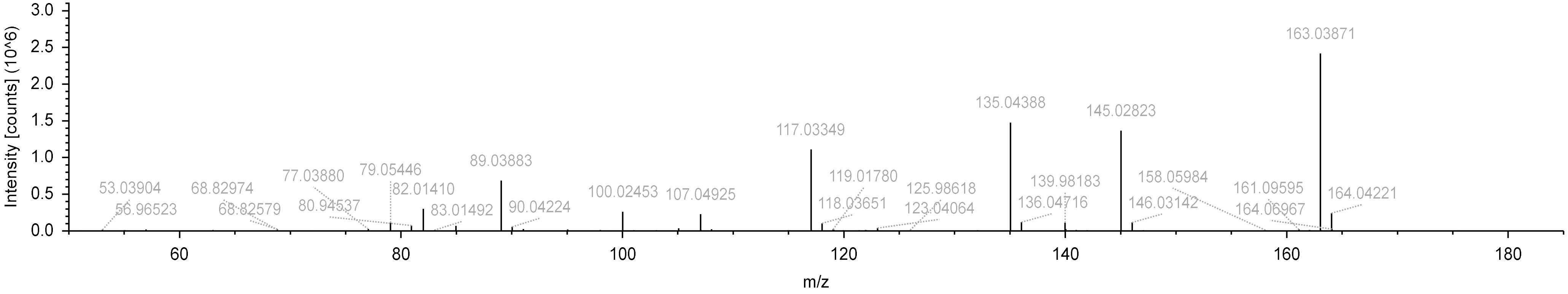

© Reported with Compound Discoverer 3.2

Structure	Name	RT [min]	Formula	Molecular Weight	Group Areas
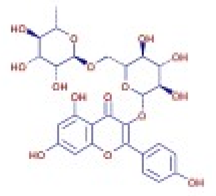	NICTOFLORIN	9.08	C27 H30 O15	594.1585	2.26e8

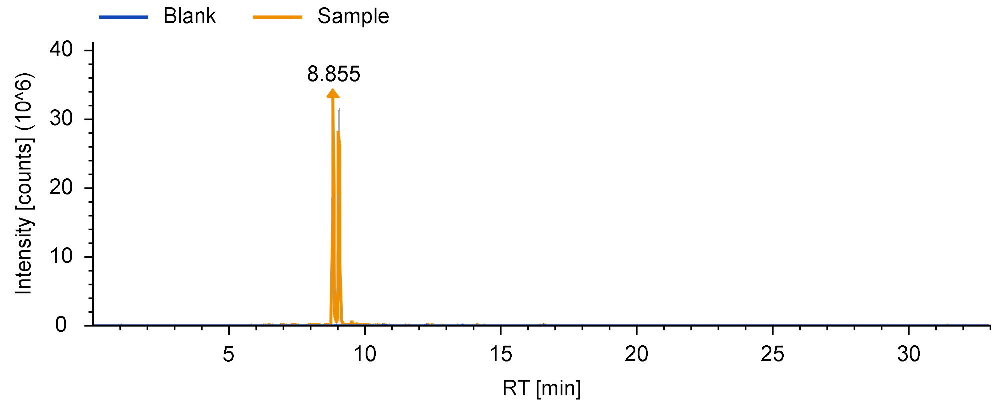

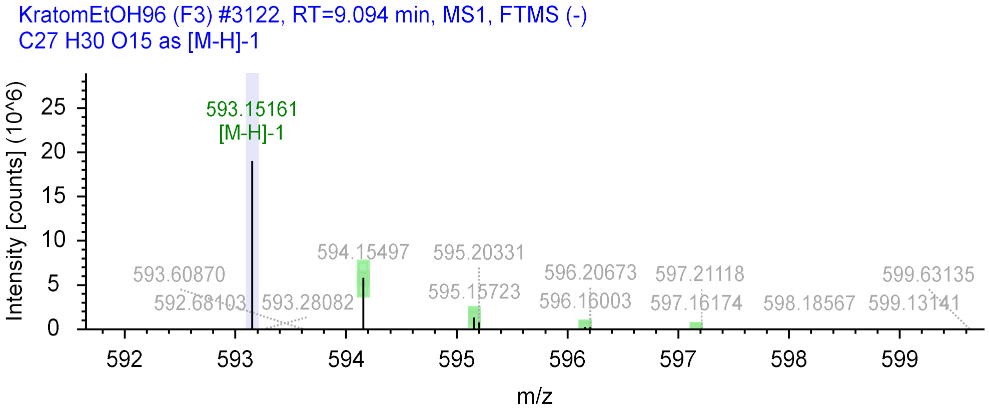

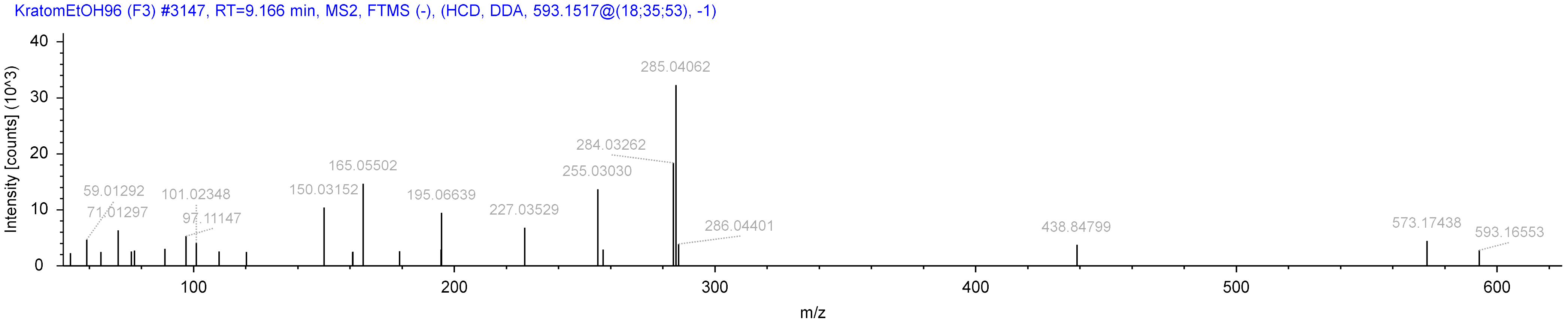

© Reported with Compound Discoverer 3.2

Structure	Name	RT [min]	Formula	Molecular Weight	Group Areas
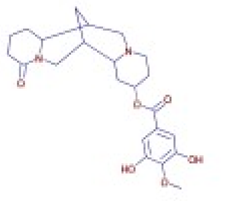	(7alpha,13beta)-2-Oxospartein-13-yl 3,5-dihydroxy-4-methoxybenzoate	9.77	C23 H30 N2 O6	430.2104	2.22e8

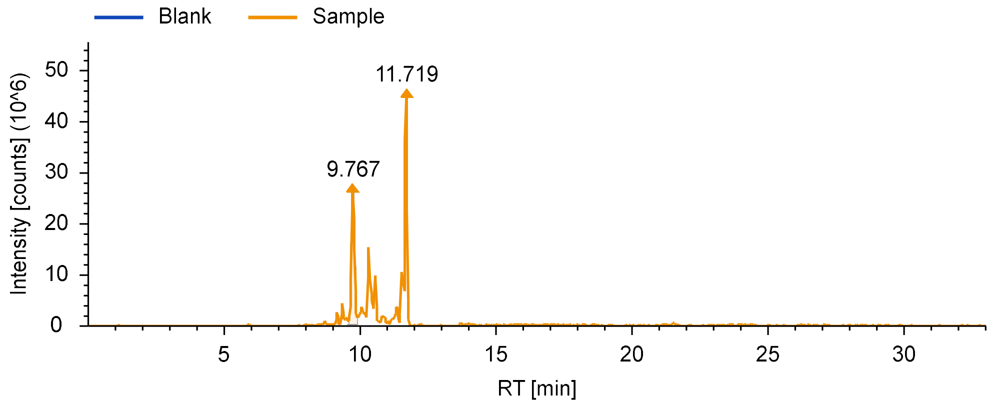

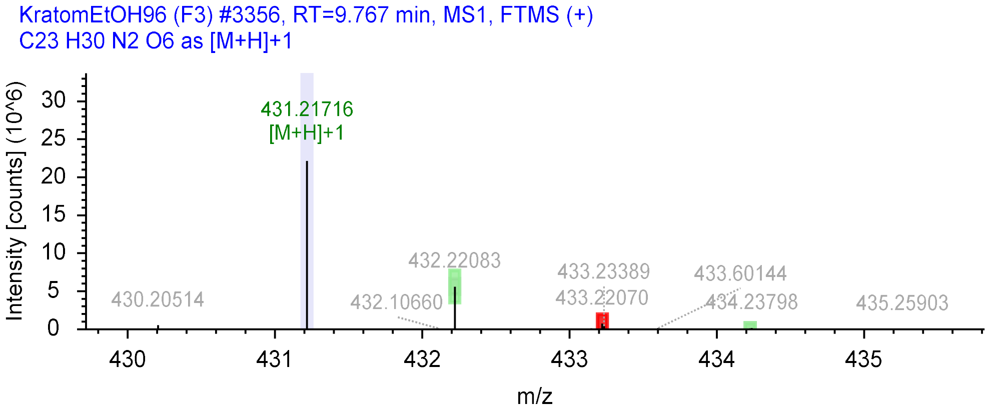

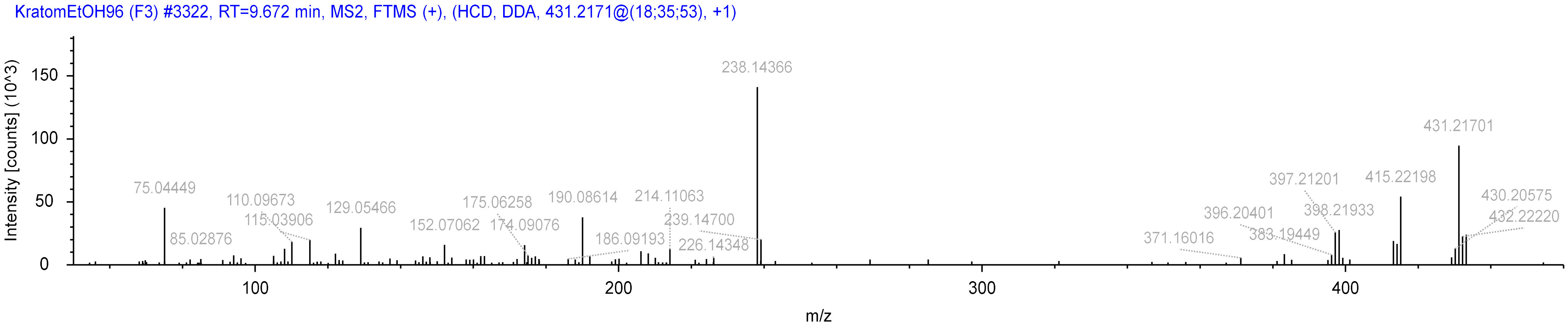

© Reported with Compound Discoverer 3.2

Structure	Name	RT [min]	Formula	Molecular Weight	Group Areas
		1.12	C14 H18 N4 O8	370.1125	2.19e8

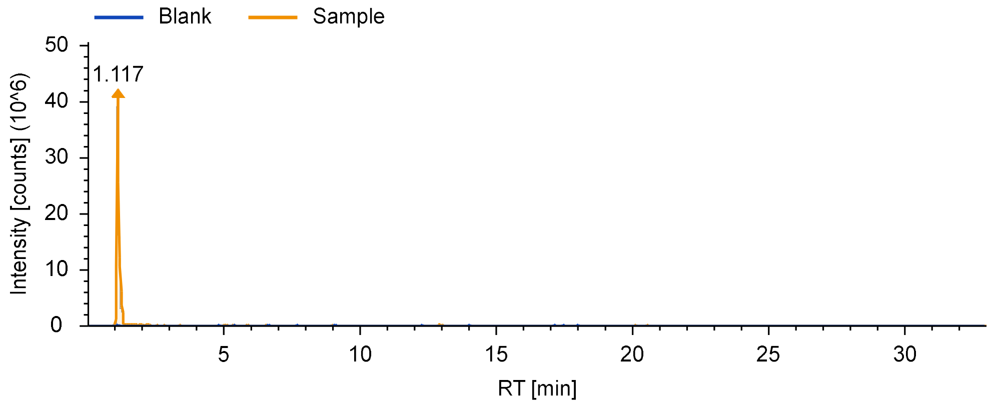

KratomEtOH96 (F3) #386, RT=1.117 min, MS1, FTMS (-)
C14 H18 N4 O8 as [M-H]-1

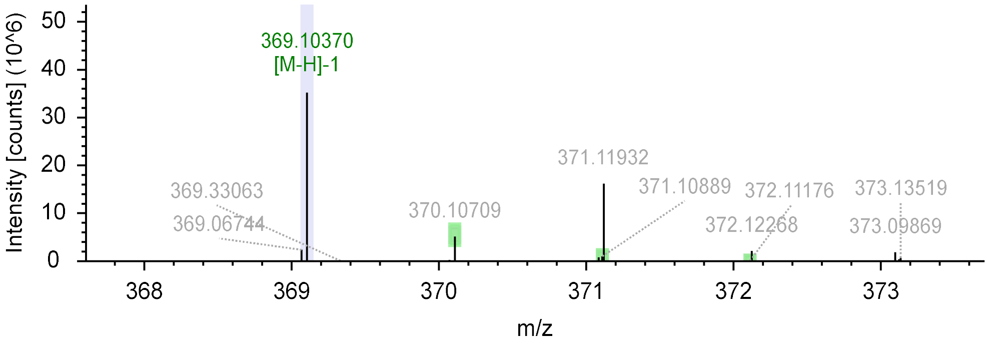

KratomEtOH96 (F3) #390, RT=1.125 min, MS2, FTMS (-), (HCD, DDA, 369.1037@(18;35;53), -1)

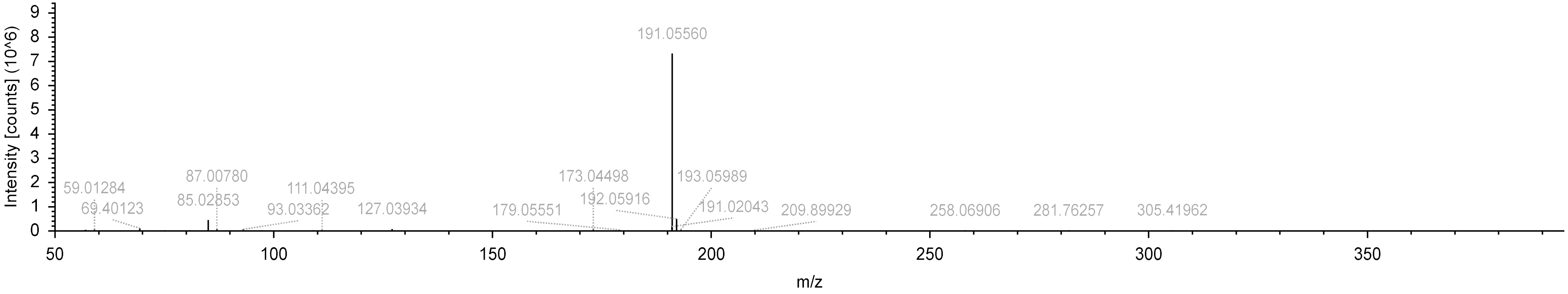

© Reported with Compound Discoverer 3.2

Compounds

Unit Laboratorium Riset Unggulan IPB

Structure	Name	RT [min]	Formula	Molecular Weight	Group Areas
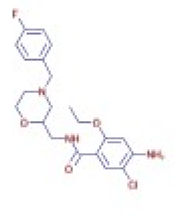	Mosapride	7.67	C21 H25 Cl F N3 O3	421.1568	2.12e8

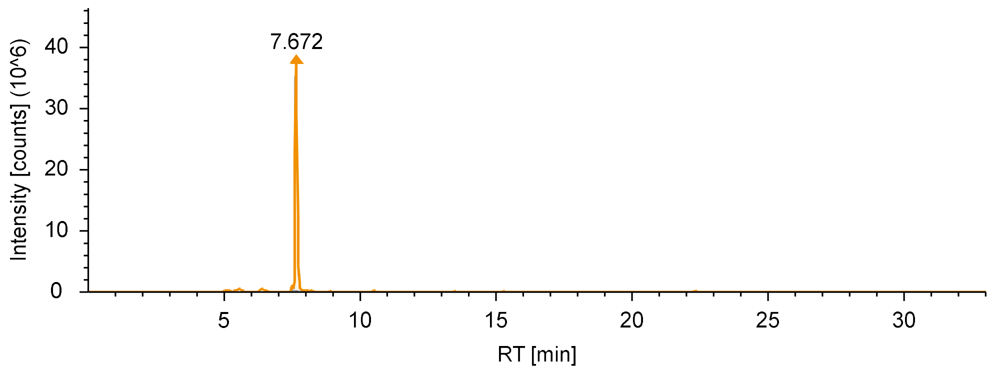

KratomEtOH96 (F3) #2624, RT=7.672 min, MS1, FTMS (+)
C21 H25 Cl F N3 O3 as [M+H]+1

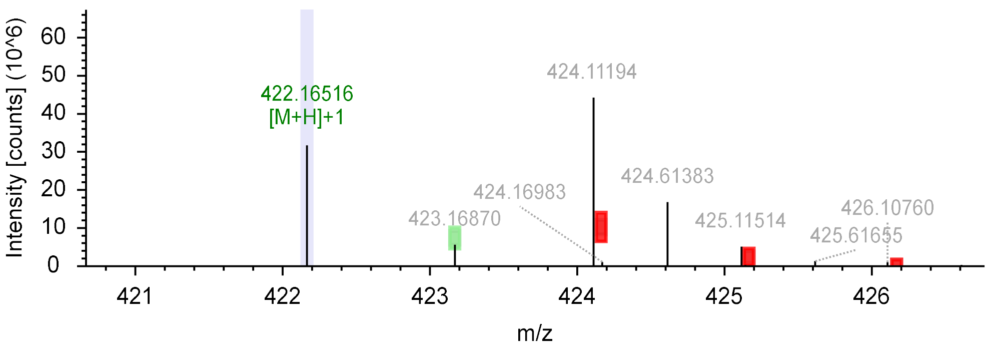

KratomEtOH96 (F3) #2617, RT=7.649 min, MS2, FTMS (+), (HCD, DDA, 424.1121@(18;35;53), +2)

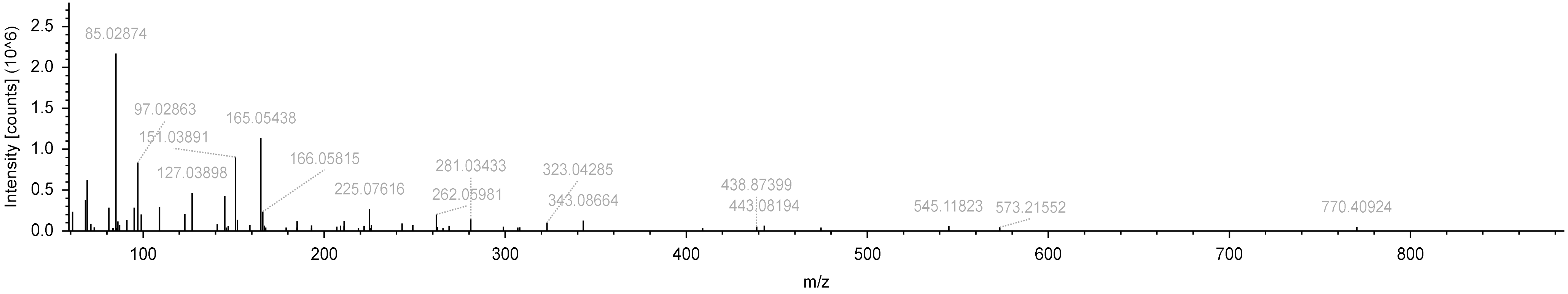

© Reported with Compound Discoverer 3.2

Compounds

Unit Laboratorium Riset Unggulan IPB

Structure	Name	RT [min]	Formula	Molecular Weight	Group Areas
		10.39	C22 H26 N2 O5	398.1842	2.09e8

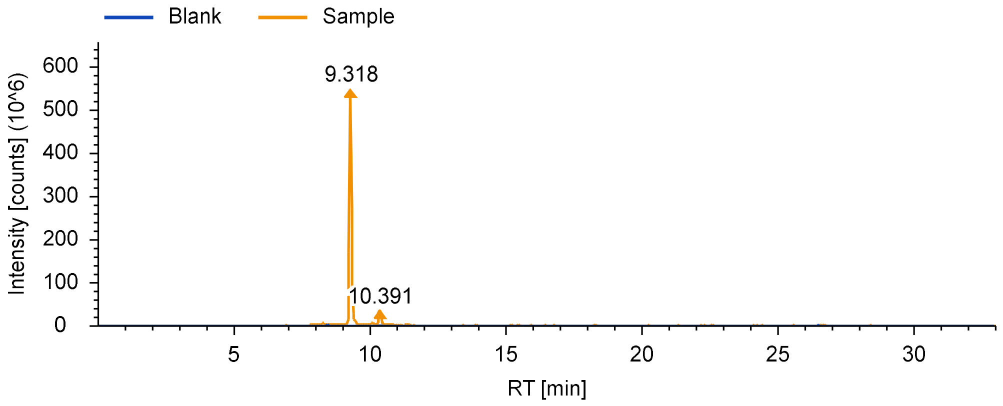

KratomEtOH96 (F3) #3572, RT=10.391 min, MS1, FTMS (+)
C22 H26 N2 O5 as [M+H]+1

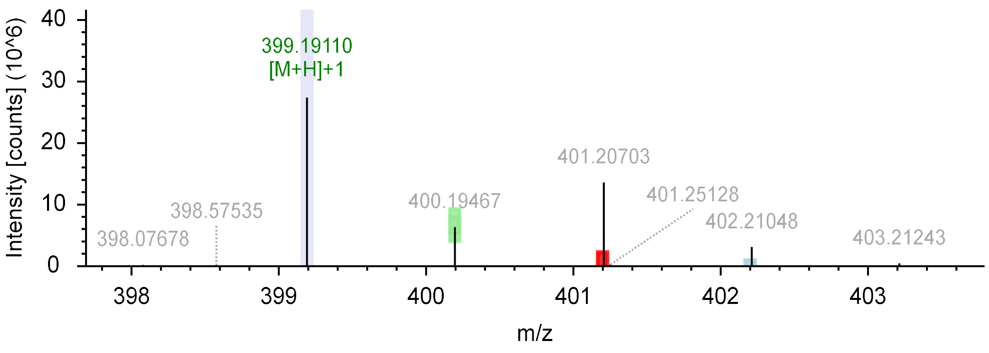

KratomEtOH96 (F3) #3563, RT=10.364 min, MS2, FTMS (+), (HCD, DDA, 399.1910@(18;35;53), +1)

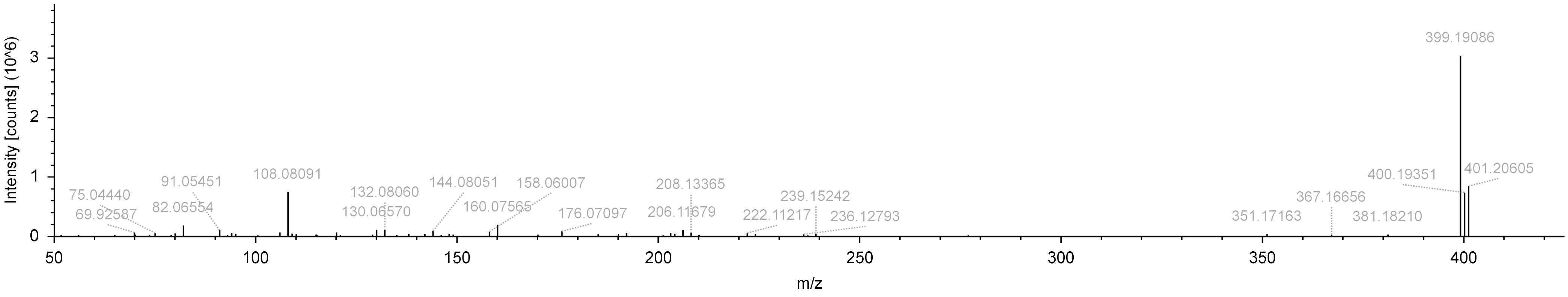

© Reported with Compound Discoverer 3.2

Structure	Name	RT [min]	Formula	Molecular Weight	Group Areas
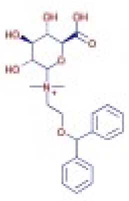	(3R,4S,5S,6S)-6-Carboxy-N-[2-(diphenylmethoxy)ethyl]-3,4,5-trihydroxy-N,N-dimethyltetrahydro-2H-pyran-2-aminium (non-preferred name)	7.00	C23 H30 N O7	432.2022	2.07e8

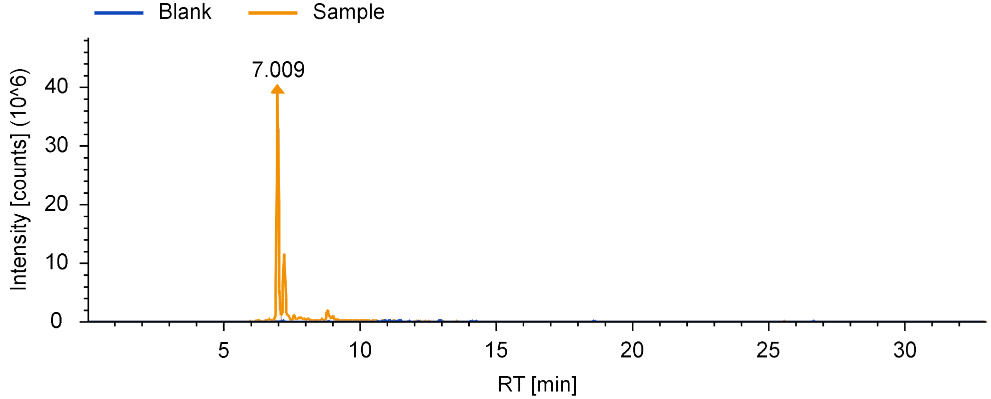

KratomEtOH96 (F3) #2390, RT=7.009 min, MS1, FTMS (-)
C23 H30 N O7 as [M-H]-1

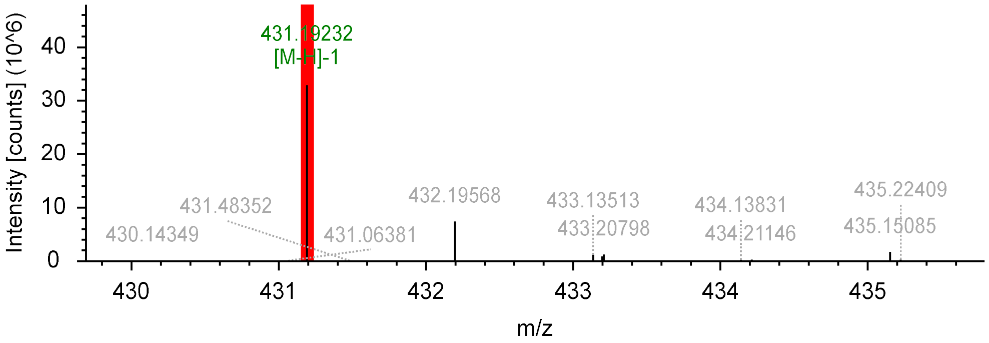

KratomEtOH96 (F3) #2369, RT=6.948 min, MS2, FTMS (-), (HCD, DDA, 431.1924@(18;35;53), -1)

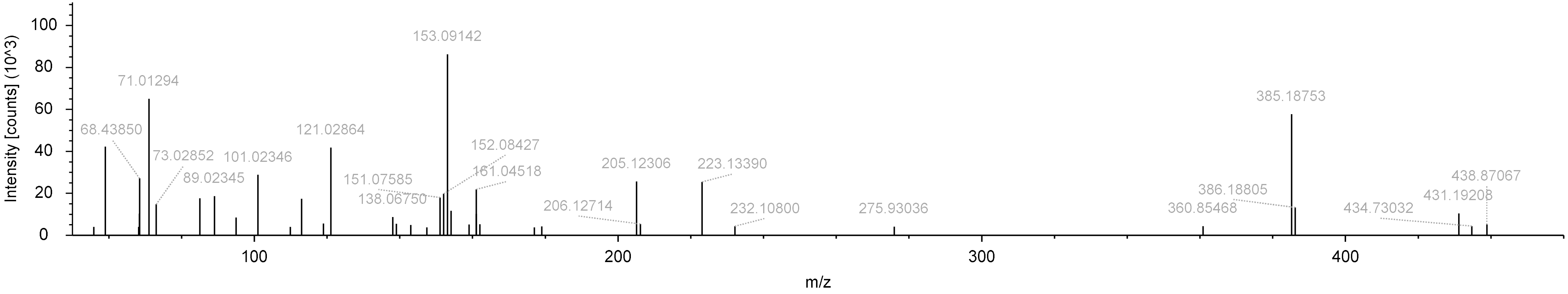

© Reported with Compound Discoverer 3.2

Compounds

Unit Laboratorium Riset Unggulan IPB

Structure	Name	RT [min]	Formula	Molecular Weight	Group Areas
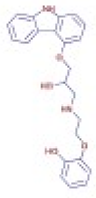	O-DESMETHYLCARVEDILOL	13.00	C23 H24 N2 O4	392.1736	2.07e8

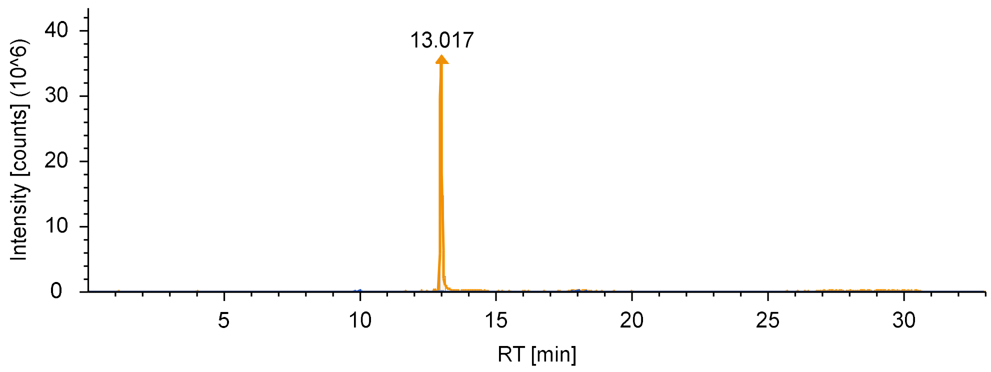

KratomEtOH96 (F3) #4472, RT=13.017 min, MS1, FTMS (+)
C23 H24 N2 O4 as [M+H]+1

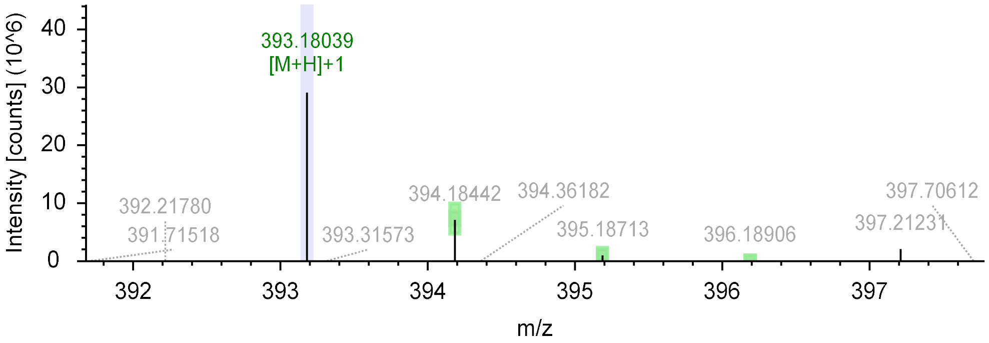

KratomEtOH96 (F3) #4451, RT=12.954 min, MS2, FTMS (+), (HCD, DDA, 393.1809@(18;35;53), +1)

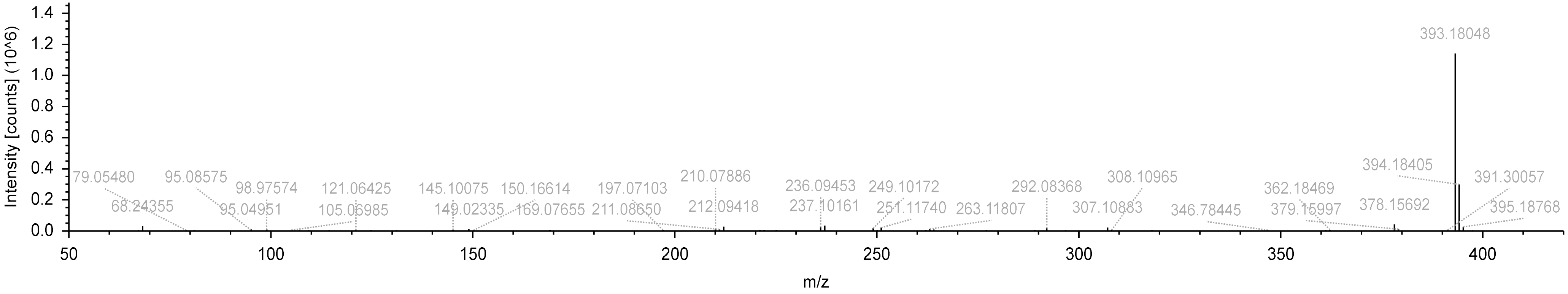

© Reported with Compound Discoverer 3.2

Structure	Name	RT [min]	Formula	Molecular Weight	Group Areas
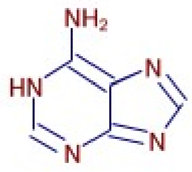	Adenine	1.13	C5 H5 N5	135.0545	2.05e8

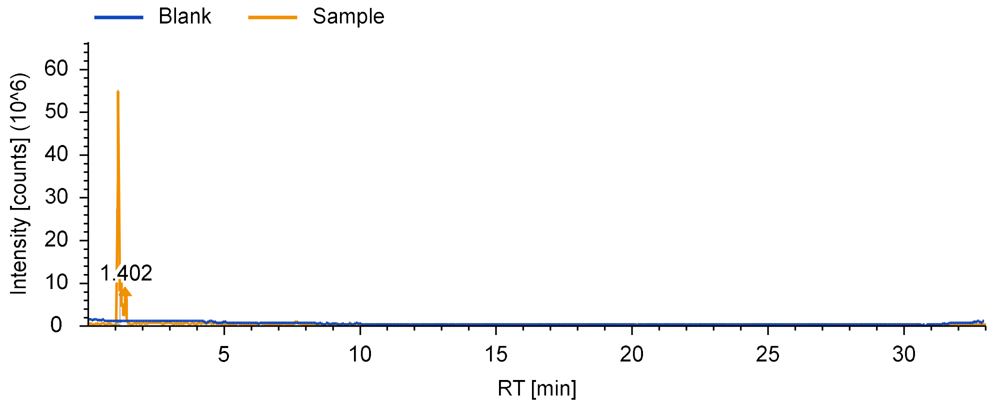

KratomEtOH96 (F3) #392, RT=1.133 min, MS1, FTMS (+)
C5 H5 N5 as [M+H]+1

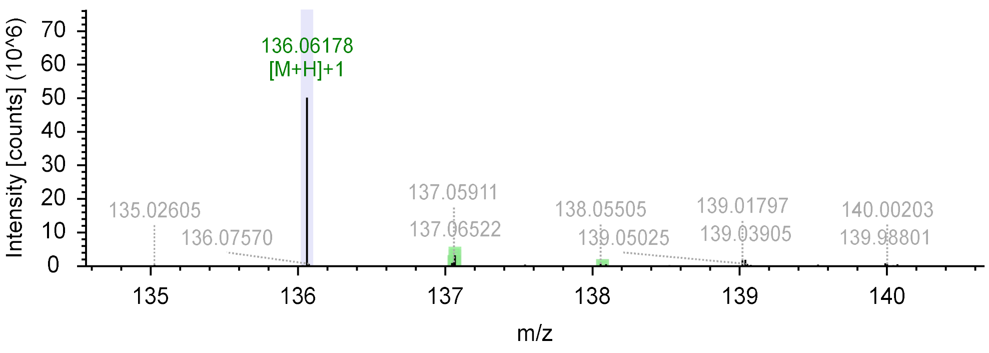

© Reported with Compound Discoverer 3.2

Compounds

Unit Laboratorium Riset Unggulan IPB

Structure	Name	RT [min]	Formula	Molecular Weight	Group Areas
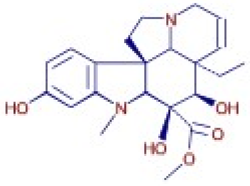	Methyl (2beta,3beta,4beta,5alpha,12beta,19alpha)-3,4,16-trihydroxy-1-methyl-6,7-didehydroaspidospermidine-3-carboxylate	8.87	C22 H28 N2 O5	400.1998	2.03e8

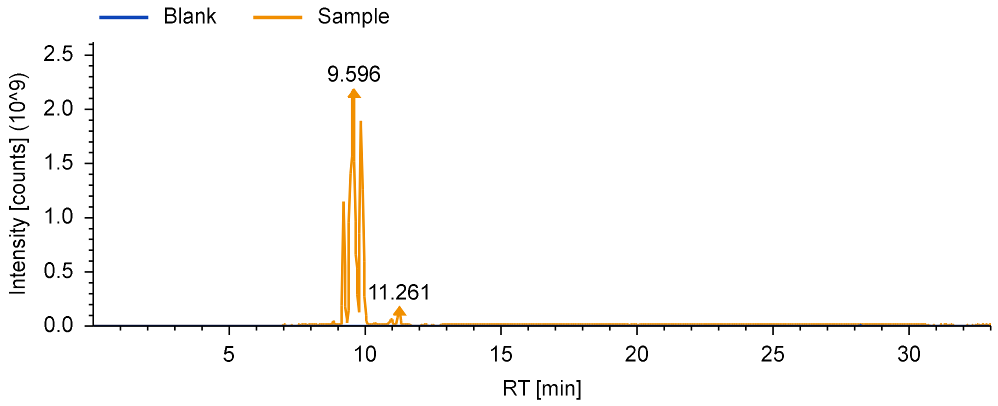

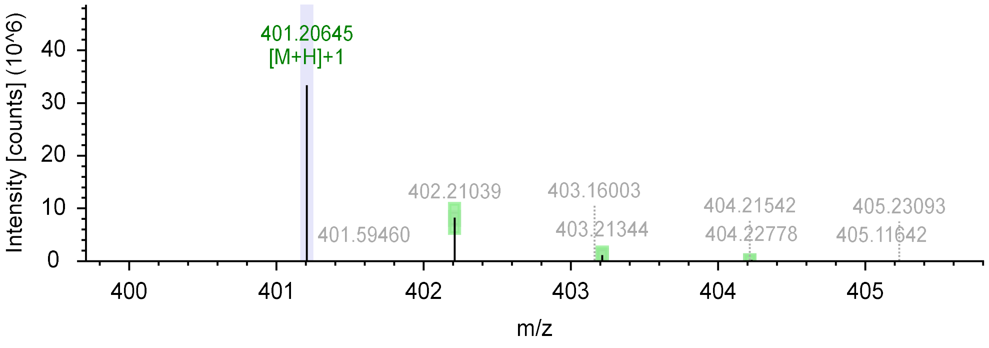

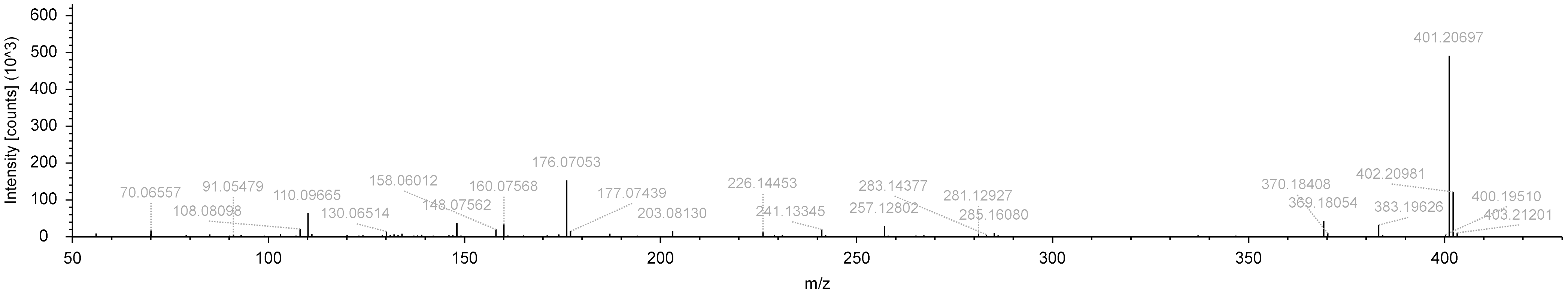

© Reported with Compound Discoverer 3.2

Structure	Name	RT [min]	Formula	Molecular Weight	Group Areas
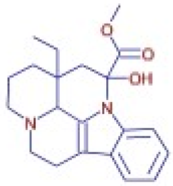	Methyl 14-hydroxy-14,15-dihydroeburnamenine-14-carboxylate	8.55	C21 H26 N2 O3	354.1943	1.98e8

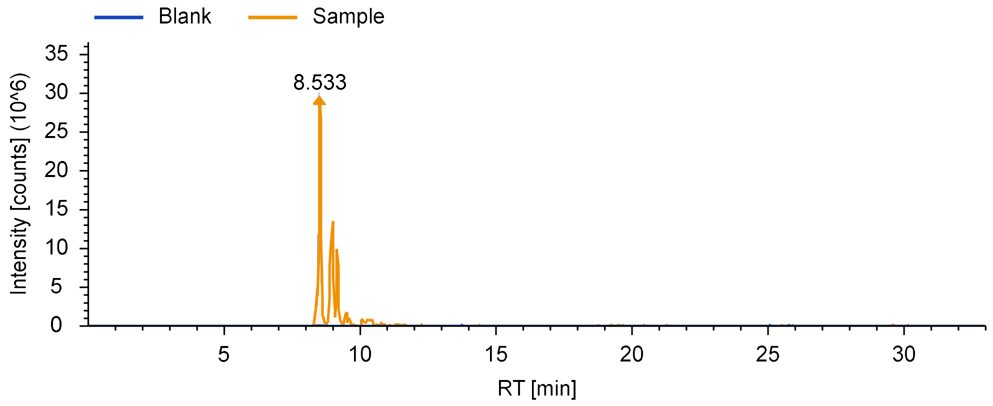

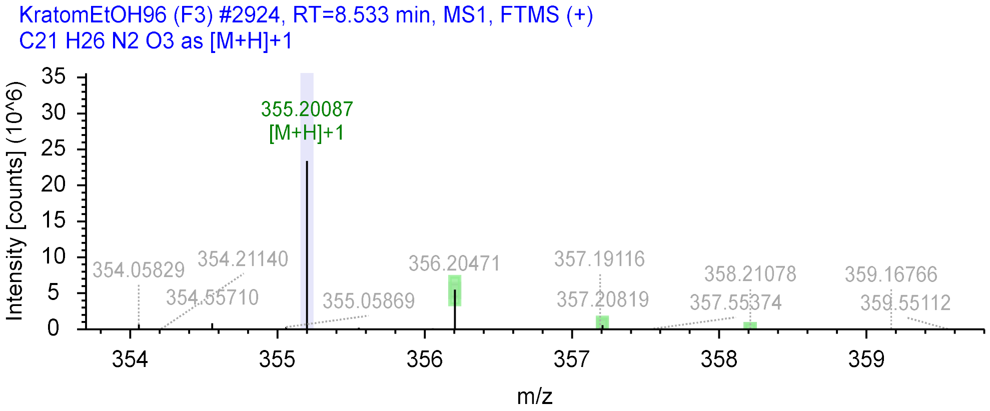

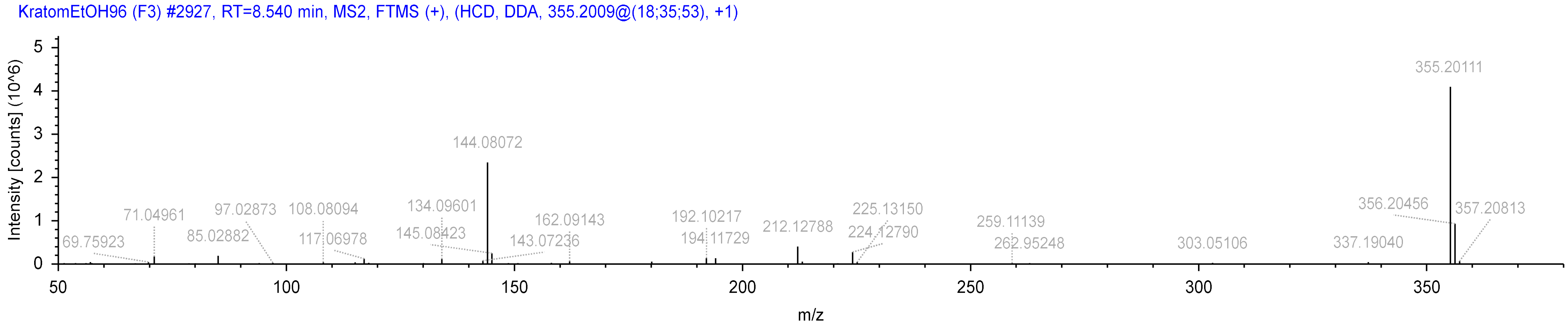

© Reported with Compound Discoverer 3.2

Structure	Name	RT [min]	Formula	Molecular Weight	Group Areas
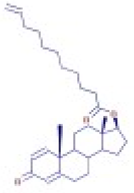	Boldenone Undecylenate	17.93	C30 H44 O3	452.3290	1.97e8

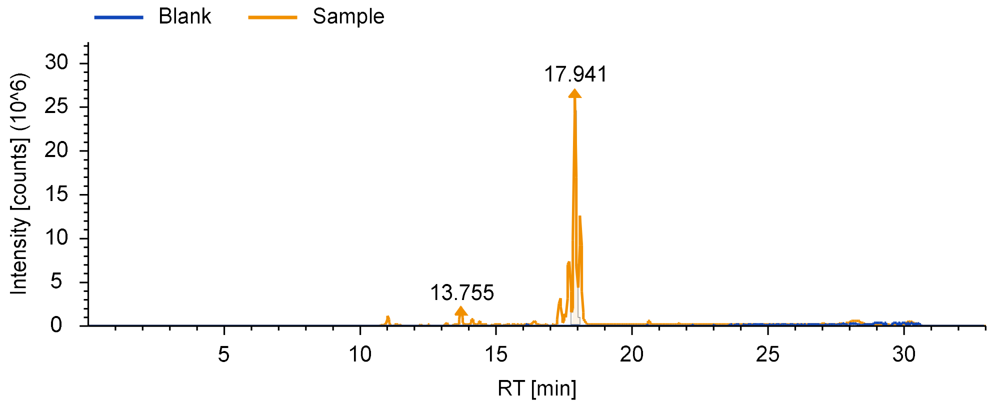

KratomEtOH96 (F3) #6140, RT=17.941 min, MS1, FTMS (+)
C30 H44 O3 as [M+H]+1

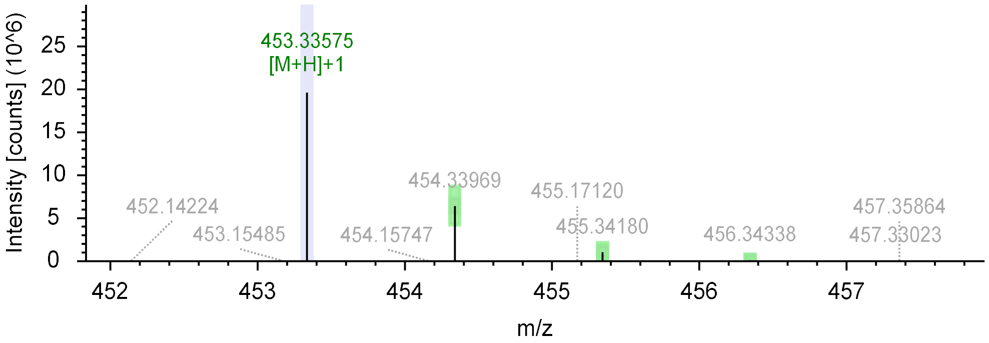

KratomEtOH96 (F3) #6166, RT=18.018 min, MS2, FTMS (+), (HCD, DDA, 453.3361@(18;35;53), +1)

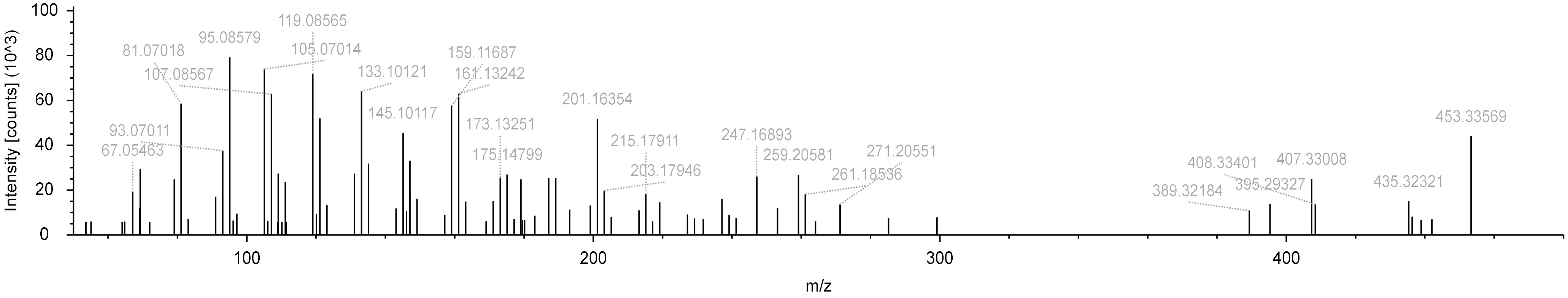

© Reported with Compound Discoverer 3.2

Structure	Name	RT [min]	Formula	Molecular Weight	Group Areas
		12.66	C46 H60 N4 O8	796.4411	1.95e8

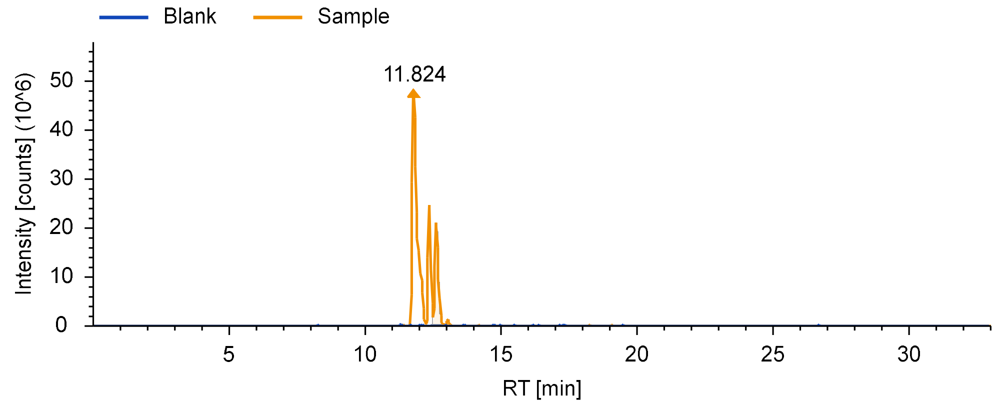

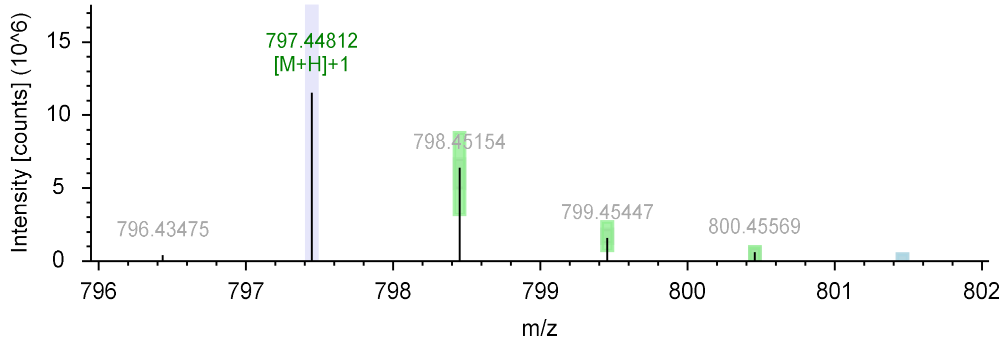

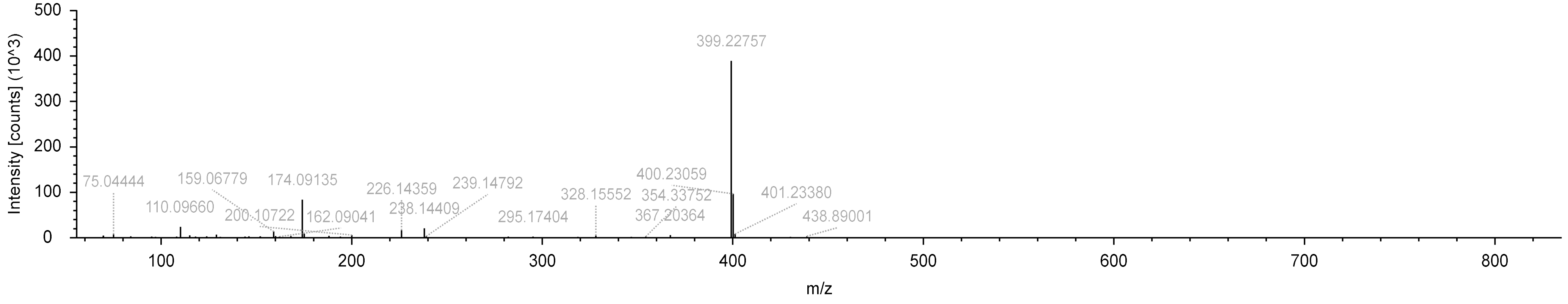

© Reported with Compound Discoverer 3.2

Structure	Name	RT [min]	Formula	Molecular Weight	Group Areas
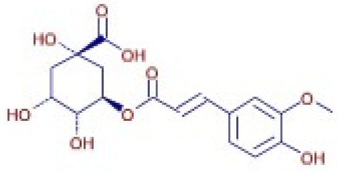	3-O-feruloyl-D-quinic acid	8.17	C17 H20 O9	368.1107	1.95e8

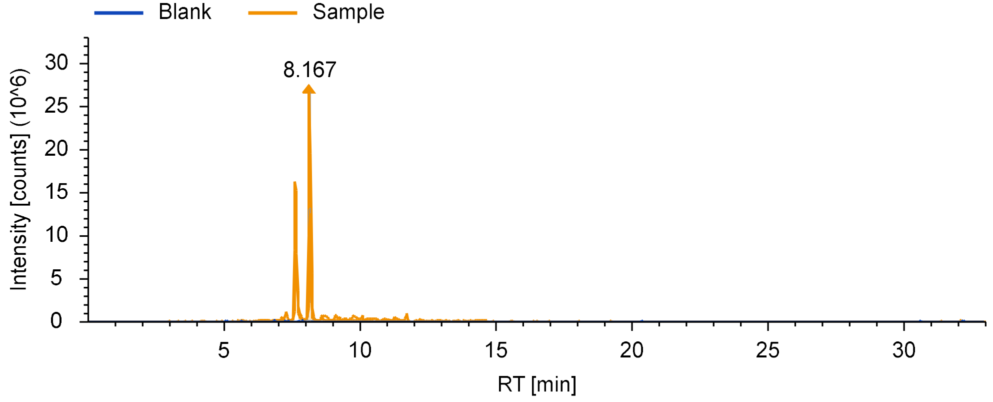

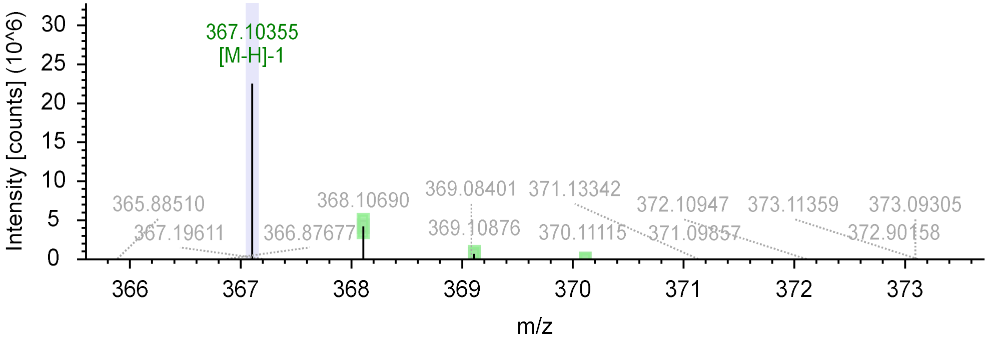

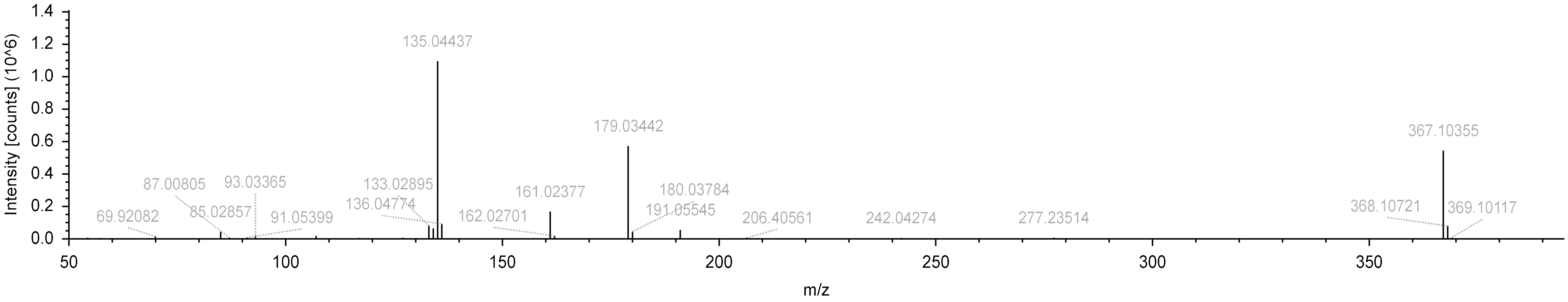

© Reported with Compound Discoverer 3.2

Structure	Name	RT [min]	Formula	Molecular Weight	Group Areas
		10.76	C23 H43 N4 O P	422.3174	1.94e8

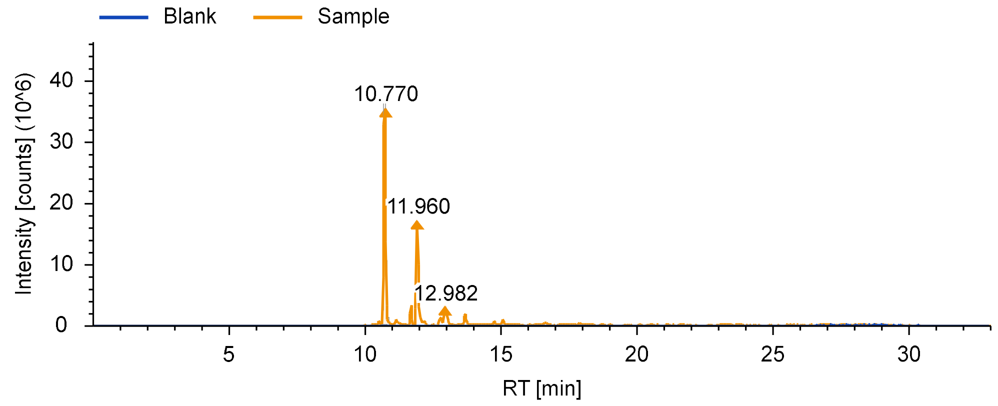

KratomEtOH96 (F3) #3704, RT=10.770 min, MS1, FTMS (+)
C23 H43 N4 O P as [M+H]+1

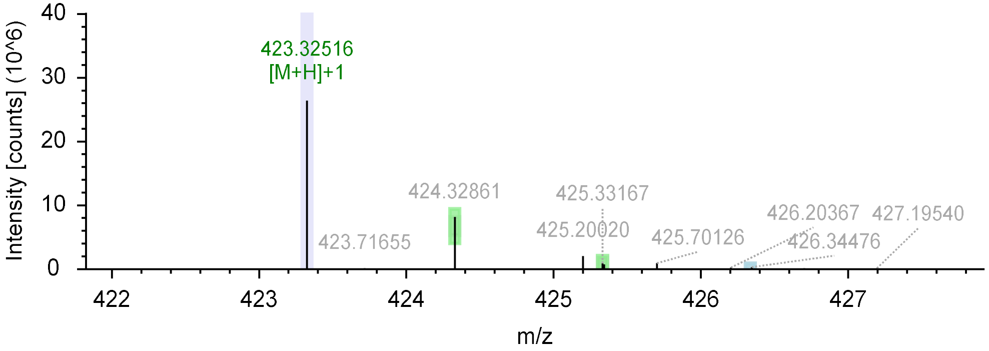

KratomEtOH96 (F3) #3684, RT=10.712 min, MS2, FTMS (+), (HCD, DDA, 423.3255@(18;35;53), +1)

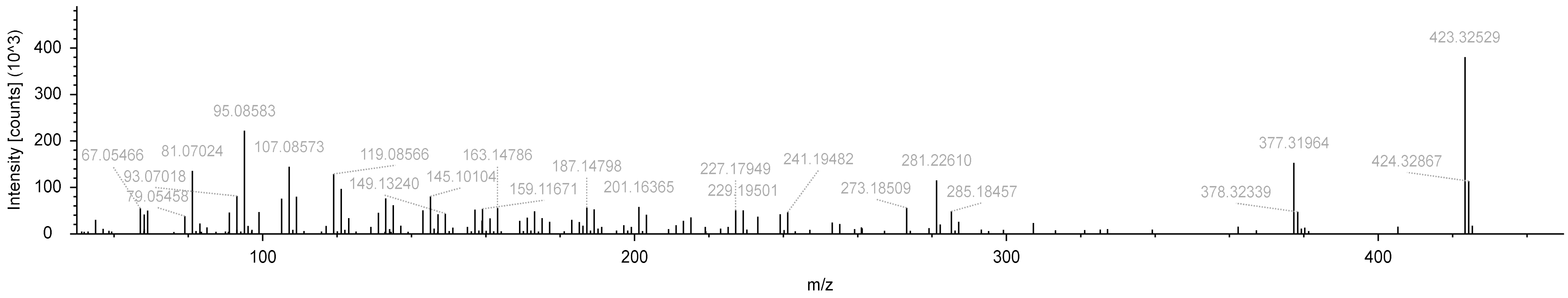

© Reported with Compound Discoverer 3.2

Compounds

Unit Laboratorium Riset Unggulan IPB

Structure	Name	RT [min]	Formula	Molecular Weight	Group Areas
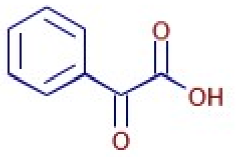	Phenylglyoxylic acid	6.16	C8 H6 O3	150.0317	1.93e8

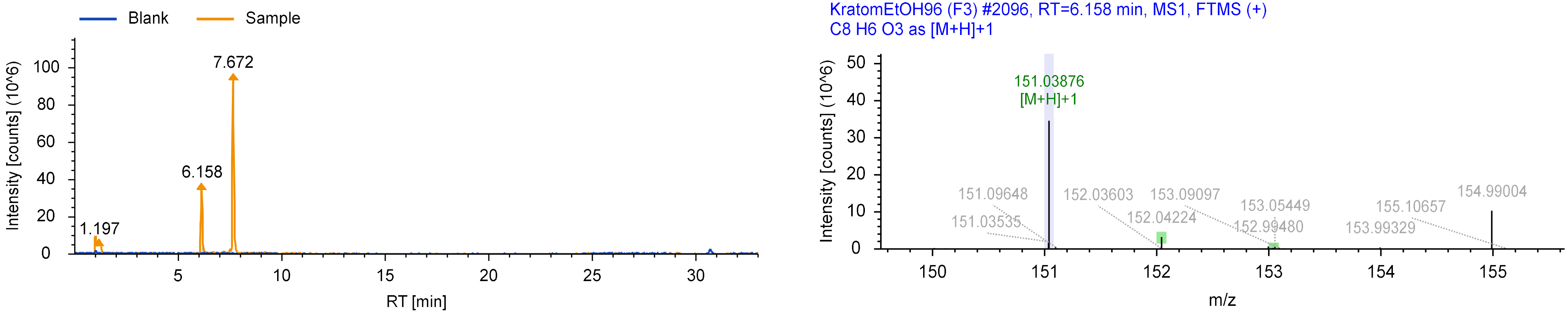

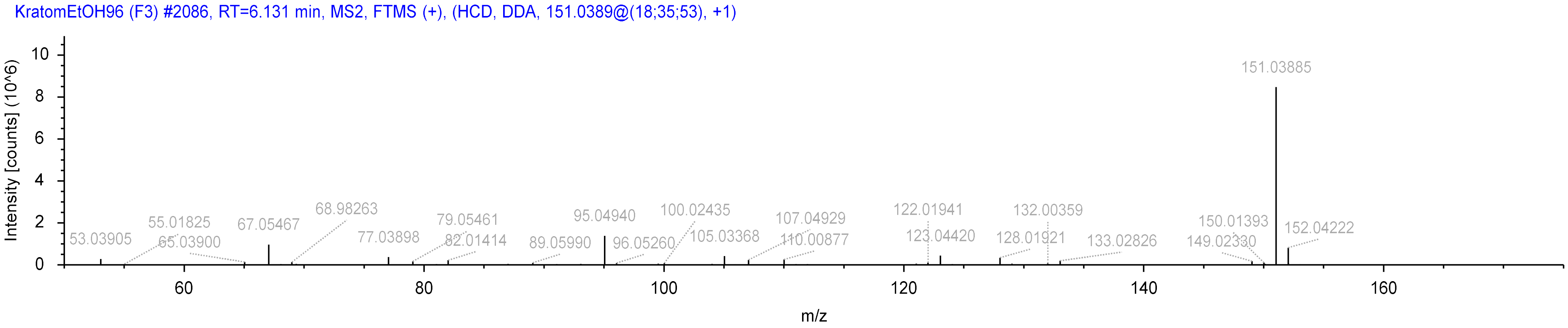

© Reported with Compound Discoverer 3.2

Structure	Name	RT [min]	Formula	Molecular Weight	Group Areas
		1.09	C20 H30 N4 O13	534.1809	1.90e8

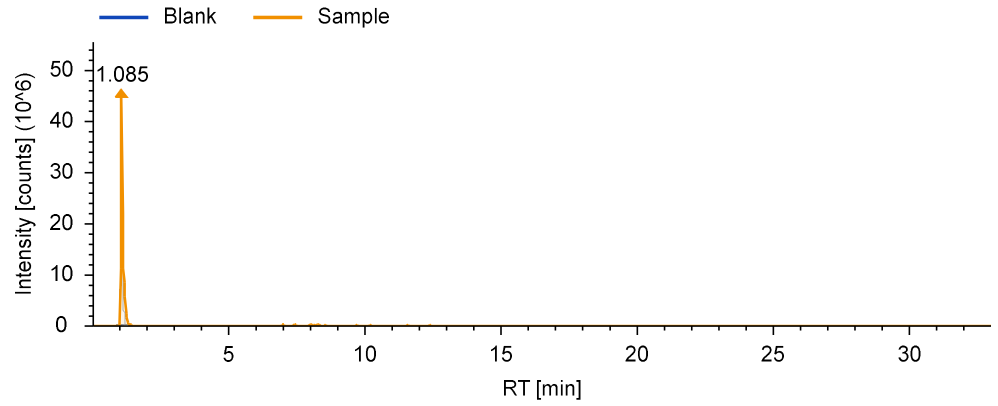

KratomEtOH96 (F3) #374, RT=1.085 min, MS1, FTMS (-)
C20 H30 N4 O13 as [M-H]-1

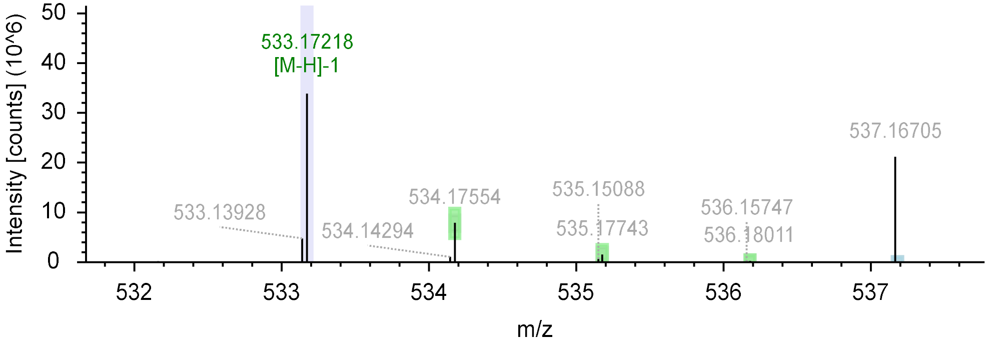

KratomEtOH96 (F3) #378, RT=1.094 min, MS2, FTMS (-), (HCD, DDA, 533.1722@(18;35;53), -1)

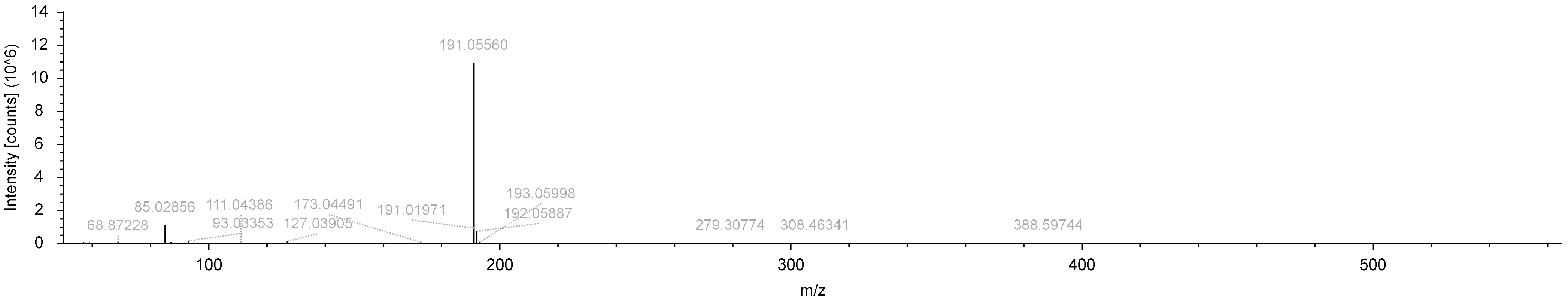

© Reported with Compound Discoverer 3.2

Structure	Name	RT [min]	Formula	Molecular Weight	Group Areas
	D-Glucono-delta-lactone	1.15	C6 H10 O6	178.0477	1.90e8

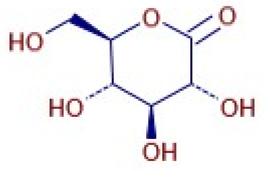

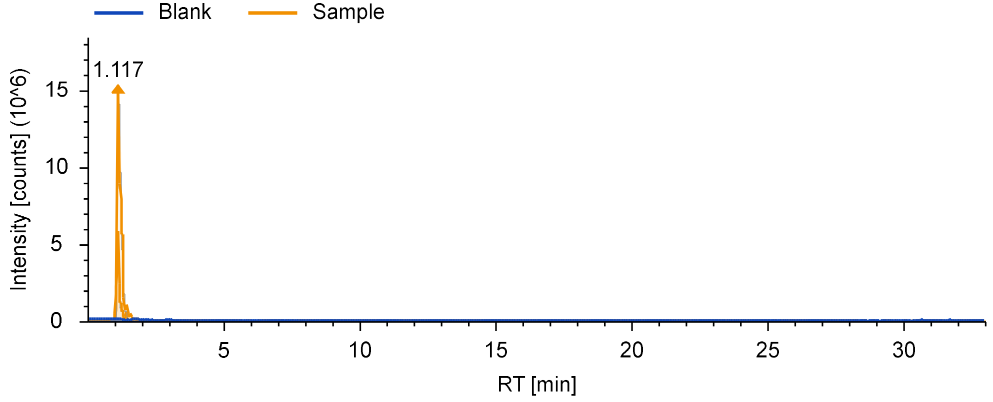

KratomEtOH96 (F3) #398, RT=1.148 min, MS1, FTMS (-)
C6 H10 O6 as [M-H]-1

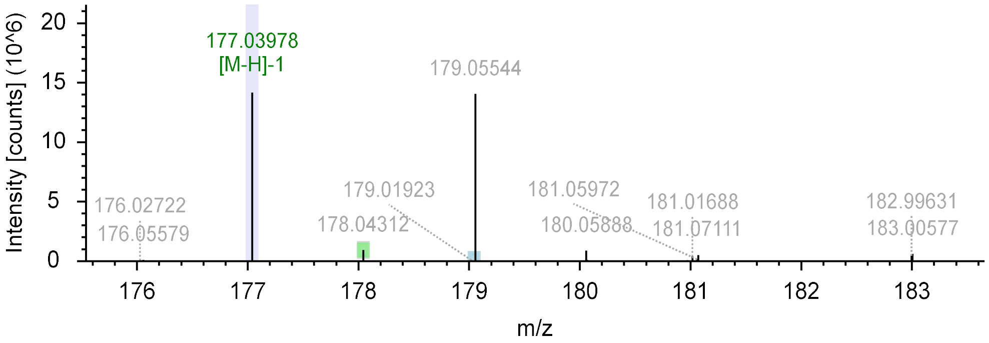

KratomEtOH96 (F3) #402, RT=1.156 min, MS2, FTMS (-), (HCD, DDA, 177.0398@(18;35;53), -1)

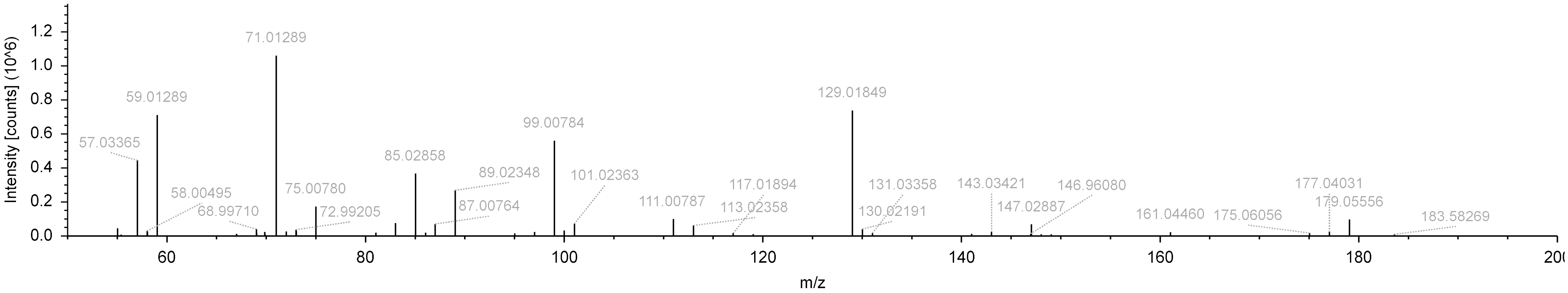

© Reported with Compound Discoverer 3.2

Compounds

Unit Laboratorium Riset Unggulan IPB

Structure	Name	RT [min]	Formula	Molecular Weight	Group Areas
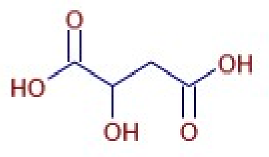	DL-Malic acid	1.15	C4 H6 O5	134.0215	1.85e8

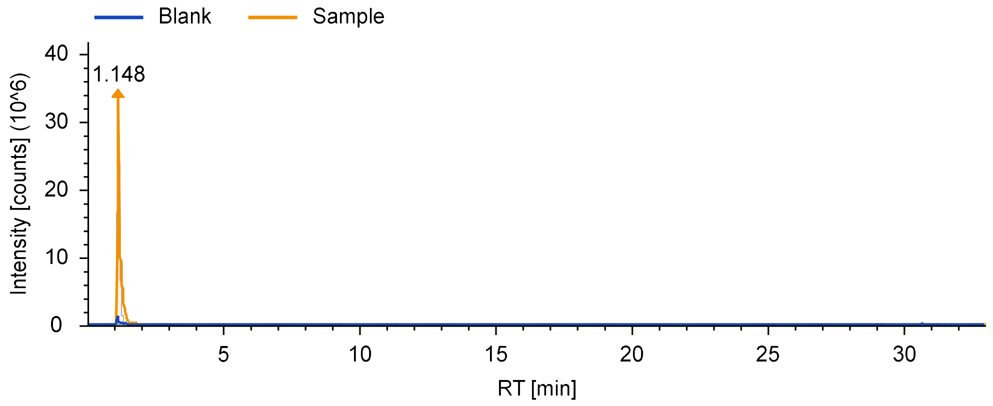

KratomEtOH96 (F3) #398, RT=1.148 min, MS1, FTMS (-)
C4 H6 O5 as [M-H]-1

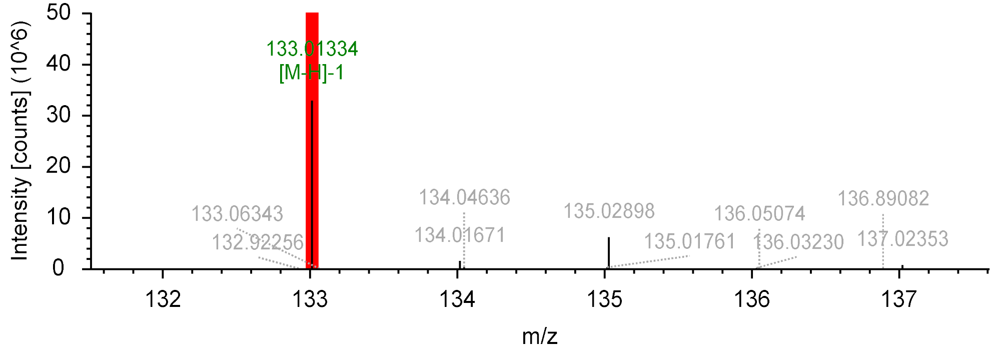

KratomEtOH96 (F3) #401, RT=1.155 min, MS2, FTMS (-), (HCD, DDA, 133.0133@(18;35;53), -1)

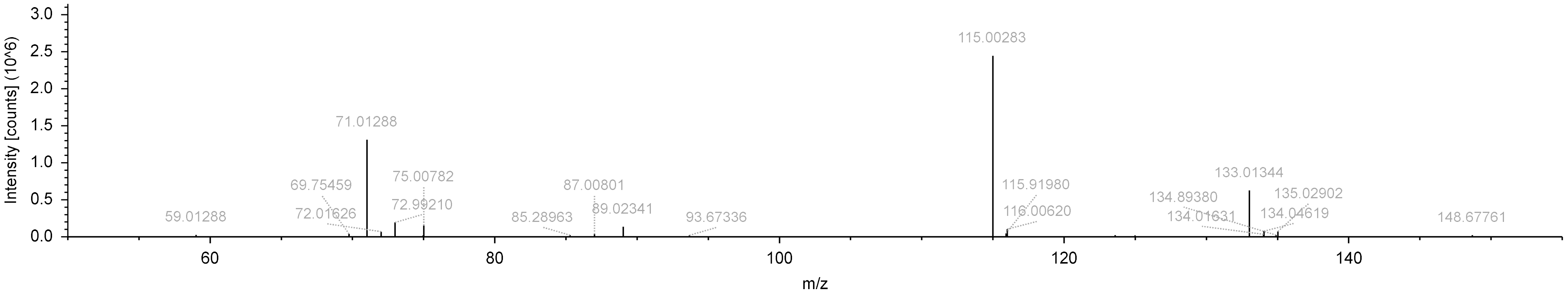

© Reported with Compound Discoverer 3.2

Compounds

Unit Laboratorium Riset Unggulan IPB

Structure	Name	RT [min]	Formula	Molecular Weight	Group Areas
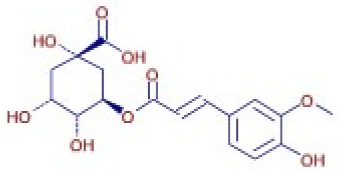	3-O-feruloyl-D-quinic acid	7.66	C17 H20 O9	368.1107	1.85e8

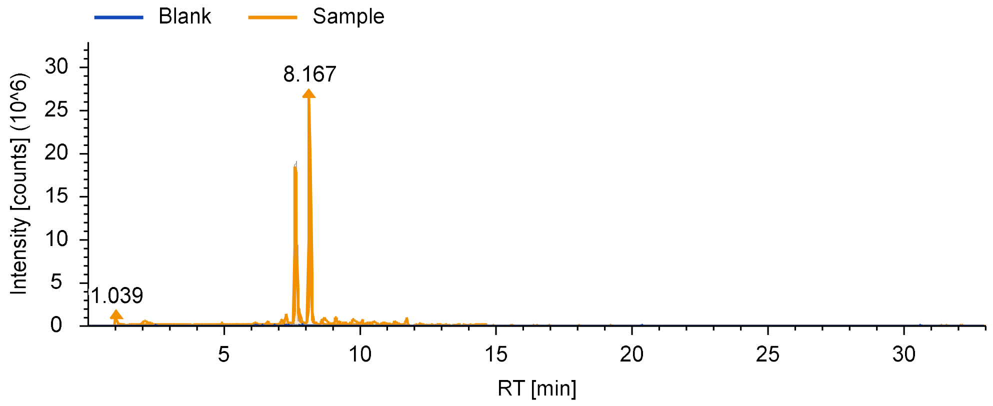

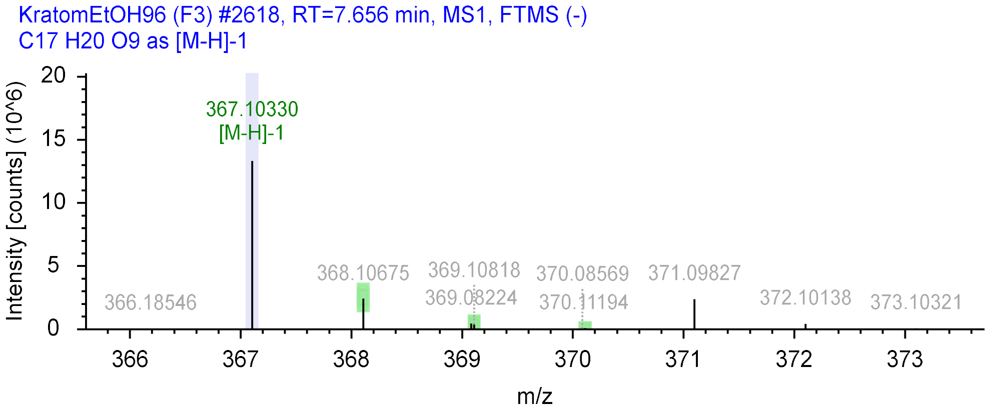

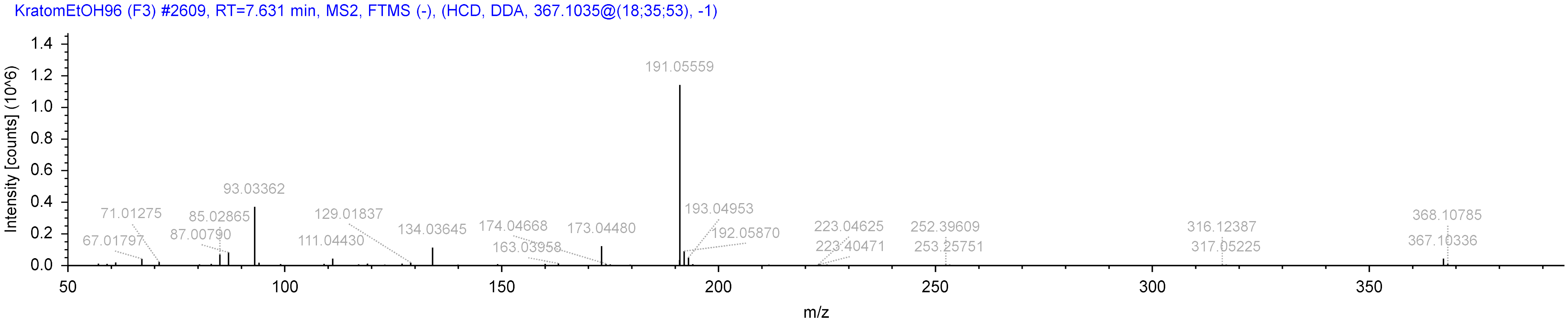

© Reported with Compound Discoverer 3.2

Compounds

Unit Laboratorium Riset Unggulan IPB

Structure	Name	RT [min]	Formula	Molecular Weight	Group Areas
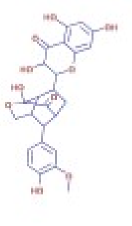	3-Hydroxy-10-(4-hydroxy-3-methoxyphenyl)-8-(3,5,7-trihydroxy-4-oxo-3,4-dihydro-2H-chromen-2-yl)-4-oxatricyclo[4.3.1.0~3,7~]decan-2-one	8.99	C25 H24 O10	484.1369	1.84e8

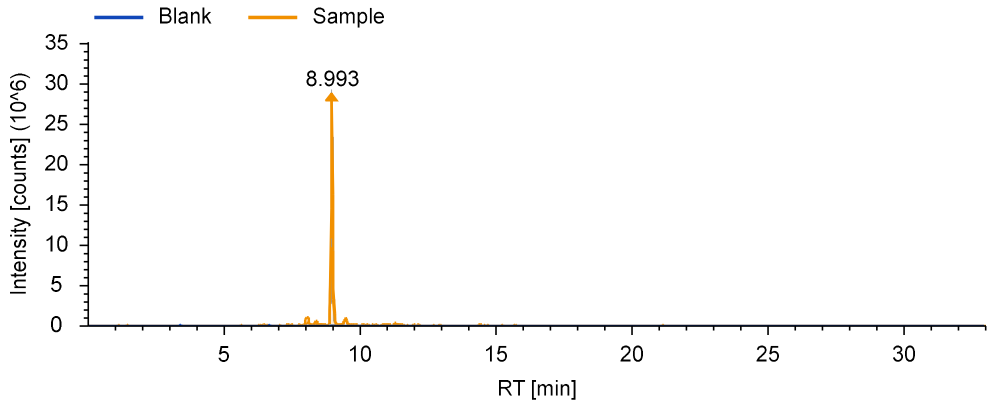

KratomEtOH96 (F3) #3086, RT=8.993 min, MS1, FTMS (-)
C25 H24 O10 as [M-H]-1

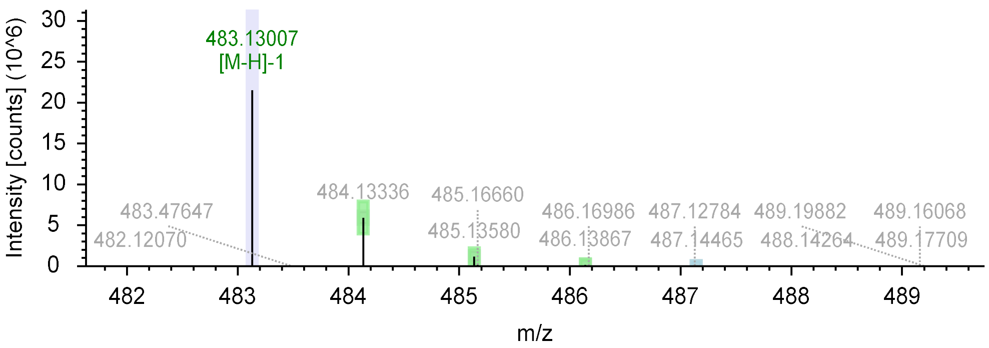

KratomEtOH96 (F3) #3067, RT=8.935 min, MS2, FTMS (-), (HCD, DDA, 483.1300@(18;35;53), -1)

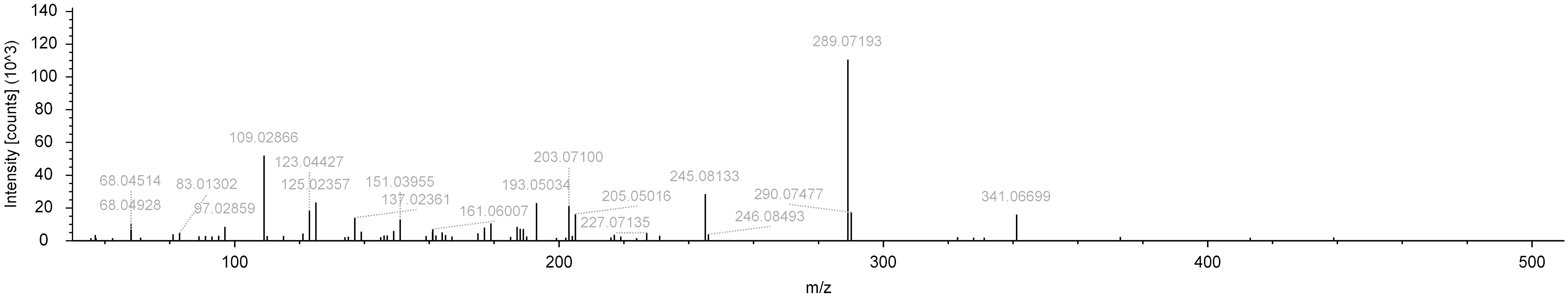

© Reported with Compound Discoverer 3.2

Structure	Name	RT [min]	Formula	Molecular Weight	Group Areas
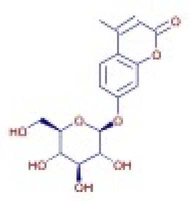	MUD	7.19	C16 H18 O8	338.1002	1.84e8

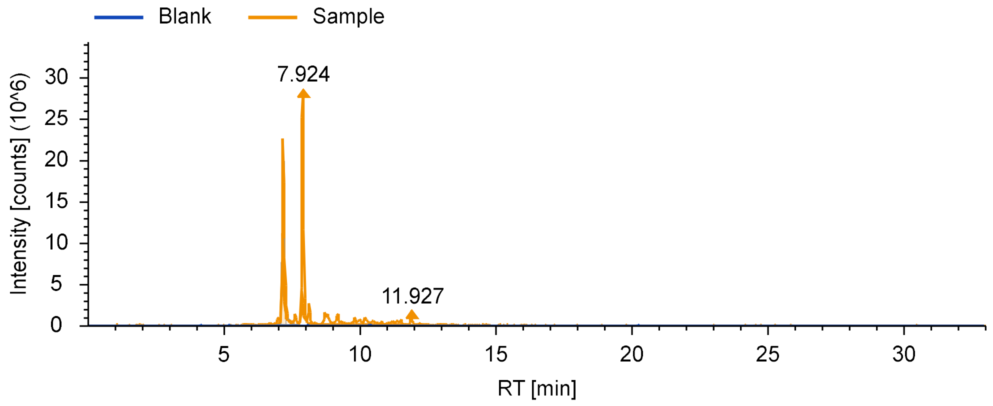

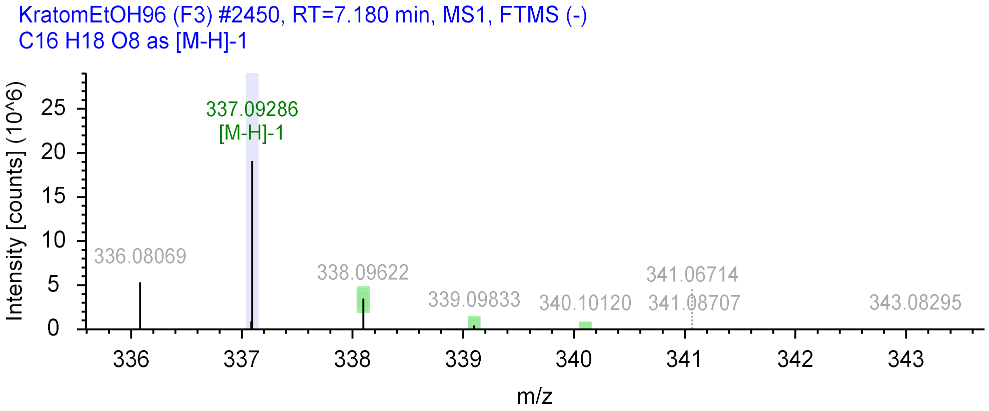

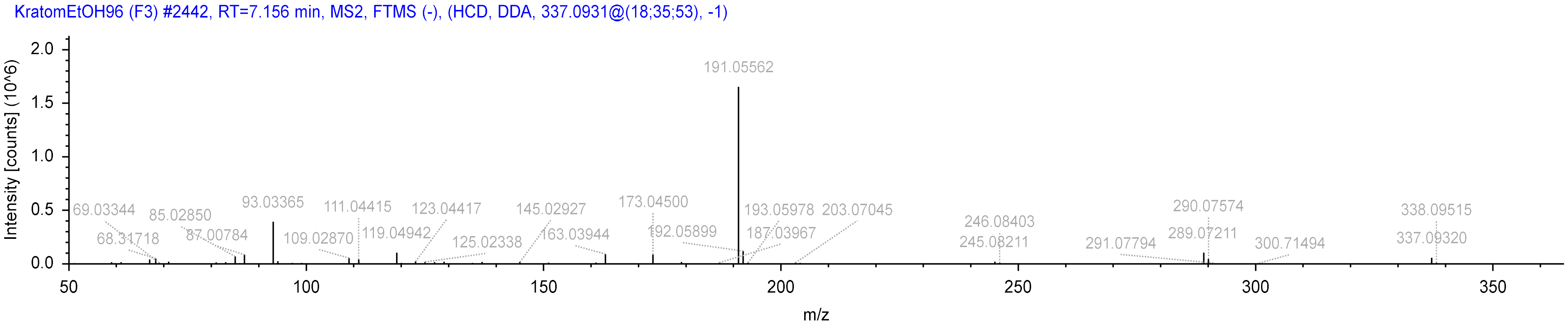

© Reported with Compound Discoverer 3.2
