# Supplementary material for: Kratom (Mitragyna speciosa) as a Phytochemical-Based Natural Product Exhibiting Opioid-like Analgesic Effects with Reduced Tolerance and Dependence Liability via TLR4-Associated Neuroimmune Modulation
Source: Molecules. 2026 Apr 26;31(9):1428. doi: 10.3390/molecules31091428 (PMC13164666; doi:10.3390/molecules31091428)
Supplement: Supplementary file 1 [file molecules-31-01428-s001.zip › Plant Determination.pdf]

KEMENTRIAN PENDIDIKAN, KEBUDAYAAN, RISET DAN TEKNOLOGI
UNIVERSITAS MULAWARMAN FAKULTAS KEHUTANAN
LABORATORIUM EKOLOGI DAN KONSERVASI BIODIVERSITAS HUTAN TROPIS
Alamat: kampus Unmul Gunung Kelua, Jl. Panajam Gd. B11 Lt.1 Samarinda 75123
Telp./Fax (0541) 7273726, Email: lab.ekobio@fahutan.unmul.ac.id

Samarinda, 09 September 2024

Nomor : 197/UN17.4.08/LL/2024
Lampiran : -
Perihal : *Hasil Identifikasi/Determinasi Tumbuhan*

Kepada Yth.
Bpk/Ibu/Sdr(i). Sultan Abdul Waris Husein (2113016027)

Fakultas Farmasi Universitas Mulawarman

di-
Tempat

Dengan Hormat,
Bersama ini kami sampaikan hasil identifikasi/determinasi tumbuhan yang saudara kirimkan ke "Herbarium Mulawarman", Laboratorium Ekologi dan Konservasi Biodiversitas Hutan Tropis Fakultas Kehutanan Universitas Mulawarman Samarinda, adalah sebagai berikut:

Kingdom	:	Plantae
Phyllum	:	Streptophyta
Class	:	Equisetopsida
Order	:	Gentianales
Family	:	Rubiaceae
Species	:	*Mitragyna speciosa* Korth.
Synonyms	:	*Nauclea speciosa* (Korth.) Miq., *Stephegyne speciosa* (Korth.) Korth., *Nauclea korthalsii* Steud., *Nauclea luzoniensis* Blanco.
Common name	:	Kratom

Demikian, semoga berguna bagi saudara.

Kepala,

Prof. Dr.Ir.Paulus Matius, M.Sc
NIP.195504111984031001

Tembusan:
Arsip
