# Supplementary material for: Kratom (Mitragyna speciosa) as a Phytochemical-Based Natural Product Exhibiting Opioid-like Analgesic Effects with Reduced Tolerance and Dependence Liability via TLR4-Associated Neuroimmune Modulation
Source: Molecules. 2026 Apr 26;31(9):1428. doi: 10.3390/molecules31091428 (PMC13164666; doi:10.3390/molecules31091428)
Supplement: Supplementary file 1 [file molecules-31-01428-s001.zip › Solvent Systems_VLC Fractionation.pdf]

Solvent Systems used in VLC fractionation of Kratom Leaf Extracts

A. Methanol Extract

Fraction	Solvent System	Ratio	Note
F1	n-Hexane	100%	Not collected
F2	n-Hexane : Ethyl acetate	75 : 25	Collected
F3	n-Hexane : Ethyl acetate	30 : 70	Collected
F4	Chloroform : Methanol	70 : 30	Collected
F5	Chloroform : Methanol	50 : 50	Collected
F6	Acetone : Methanol	50 : 50	Collected
F7	Chloroform : Methanol	40 : 60	Collected
F8	Methanol	100%	Collected
F9	Methanol	80%	Not collected

B. Ethanol Extract

Fraction	Solvent System	Ratio	Note
F0	n-Hexane	100%	Not collected
F1	n-Hexane : Ethyl acetate	70 : 30	Collected
F2	n-Hexane : Ethyl acetate	50 : 50	Collected
F3	Chloroform : Methanol	70 : 30	Collected
F4	Chloroform : Methanol	50 : 50	Collected
F5	Chloroform : Methanol	30 : 70	Collected
F6	Methanol	100%	Collected
F7	Methanol	80%	Collected
