# Supplementary material for: Kratom (Mitragyna speciosa) as a Phytochemical-Based Natural Product Exhibiting Opioid-like Analgesic Effects with Reduced Tolerance and Dependence Liability via TLR4-Associated Neuroimmune Modulation
Source: Molecules. 2026 Apr 26;31(9):1428. doi: 10.3390/molecules31091428 (PMC13164666; doi:10.3390/molecules31091428)

**Remaining Number of Mice**

1. Normal Control: 4/4
2. Negative Control: 4/6
3. Positive Control: 5/6
4. Fraction 1: 5/7; 2 mice died due to fighting/attack
5. Fraction 2: 5/6
6. Fraction 3: 5/6
7. Fraction 4: 4/6
8. Fraction 5: 6/6; no mortality
9. Fraction 6: 5/8; the majority of mice died
10. Fraction 7: 4/6; the majority of mice died

**Evidence of Counteracting Morphine Effects**
**NEGATIVE CONTROL GROUP**

The morphine-treated negative control group exhibited the following characteristics:

1. Increased blood flow in the face and forelimbs, resulting in a reddish appearance

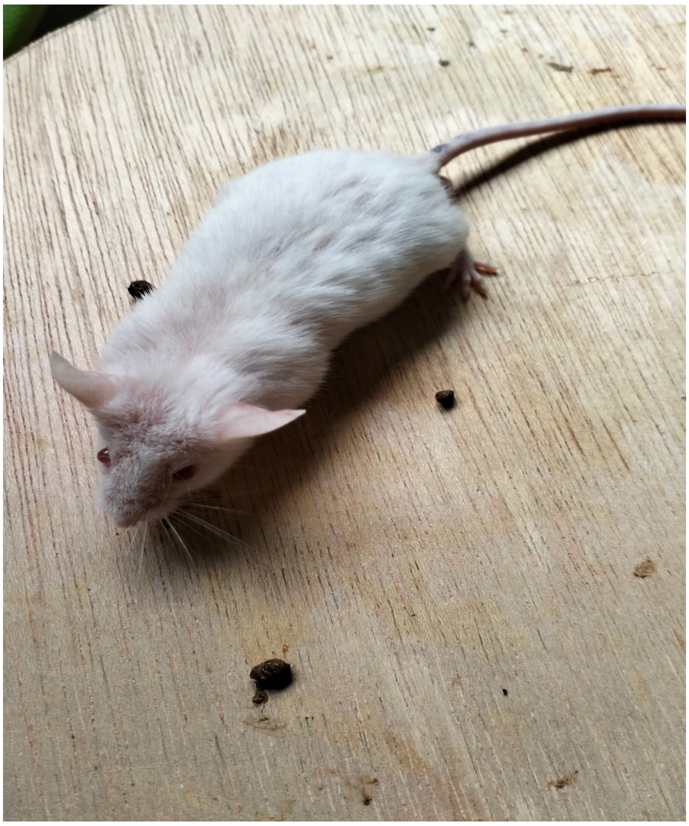

2. Piloerection

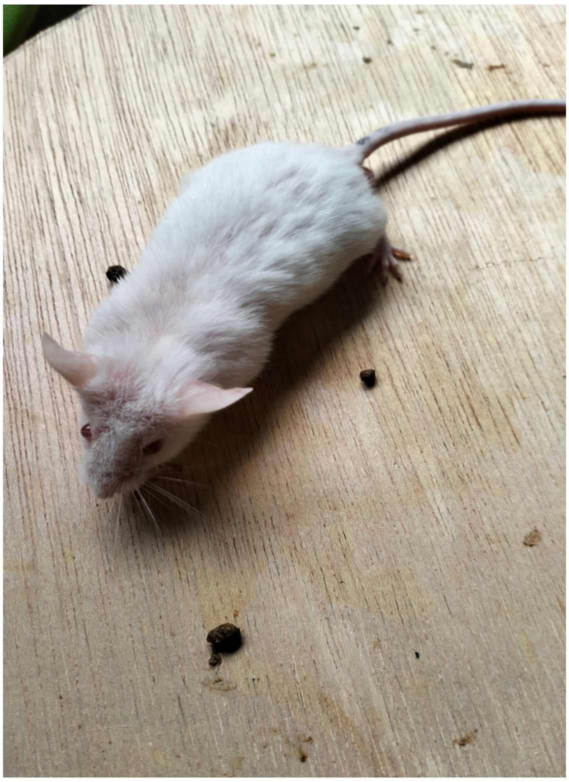

3. Ataxia (impaired motor coordination)

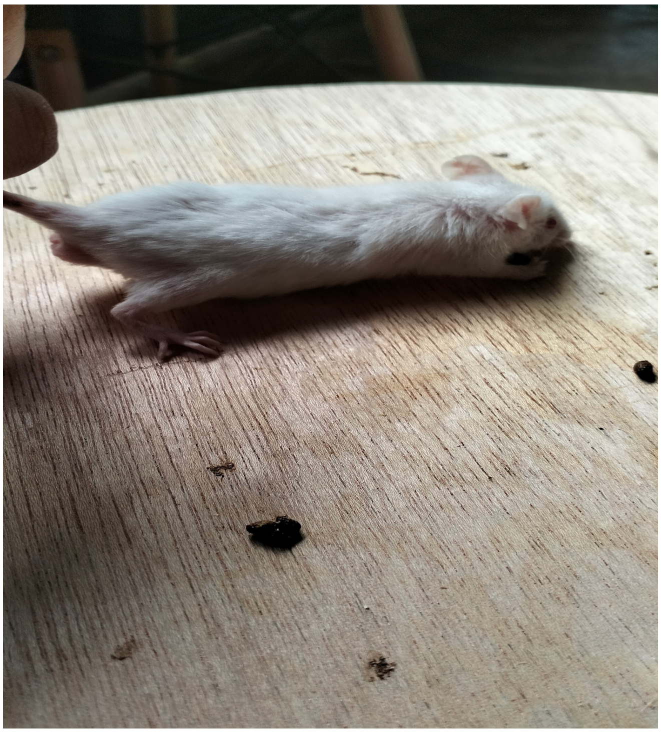

4. Not aggressive (looking downward when placed on the circular board)

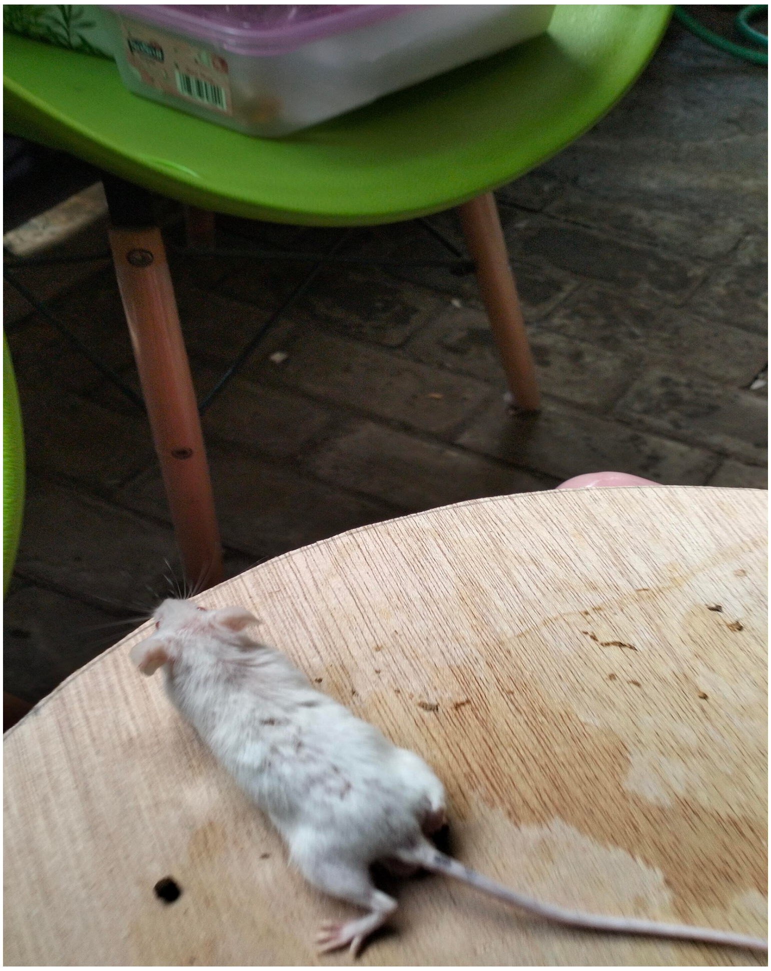

5. No pain response when the tail and ear were pinched

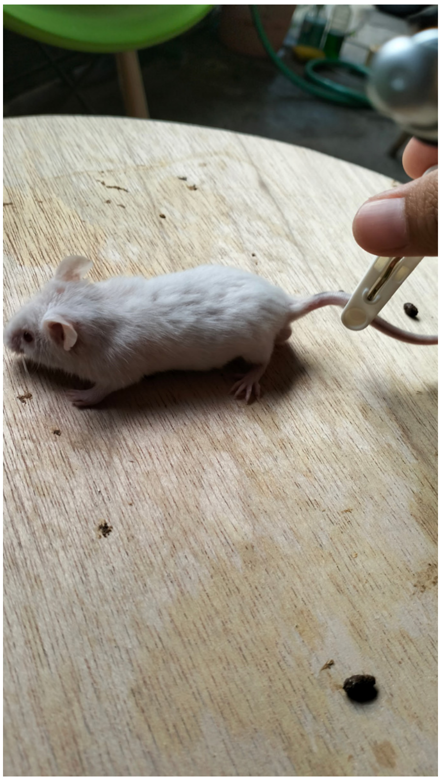

6. Passive behavior, leading to increased catalepsy time

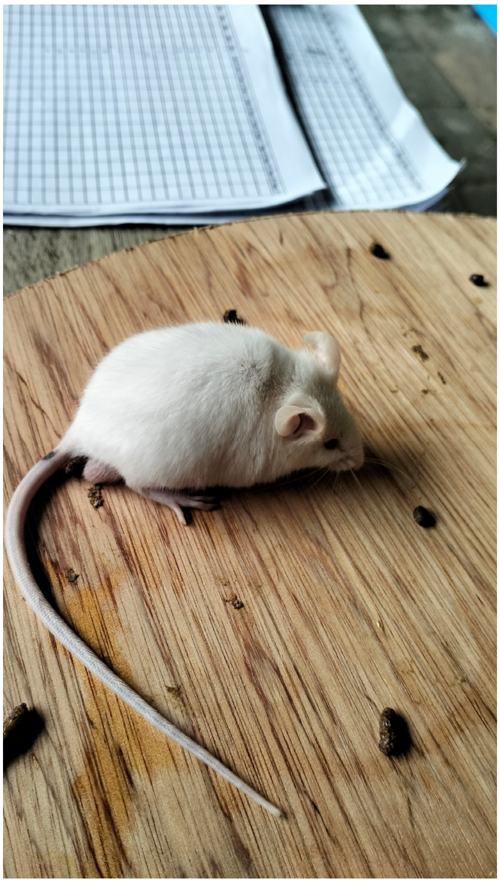

OBSERVATIONS IN GROUP 5 (ASSUMED → SAFE)

Group 5 was assumed to be the safe-fraction group because no animal deaths were observed. Based on behavioral observations over 12 days, Group 5 showed characteristics closely resembling those of the normal control mice (treated only with NaCMC), namely:

1. Aggressive

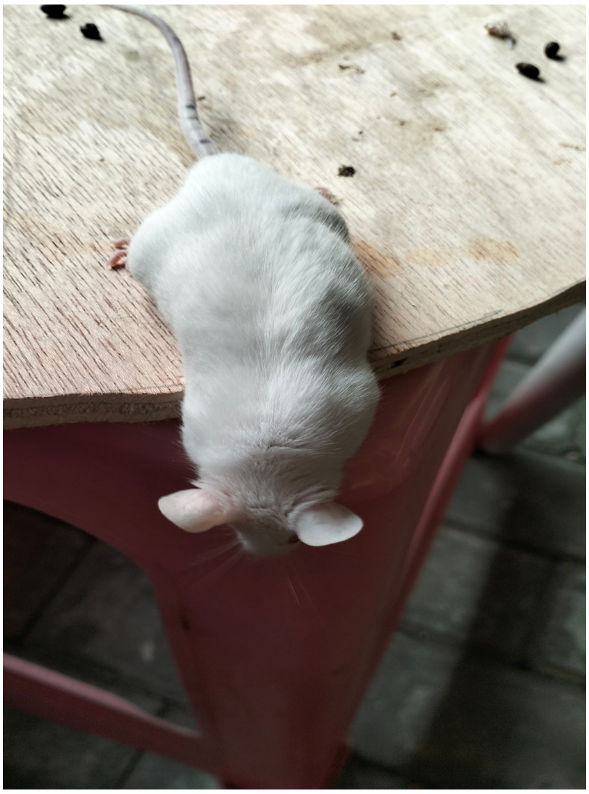

2. Tail erect

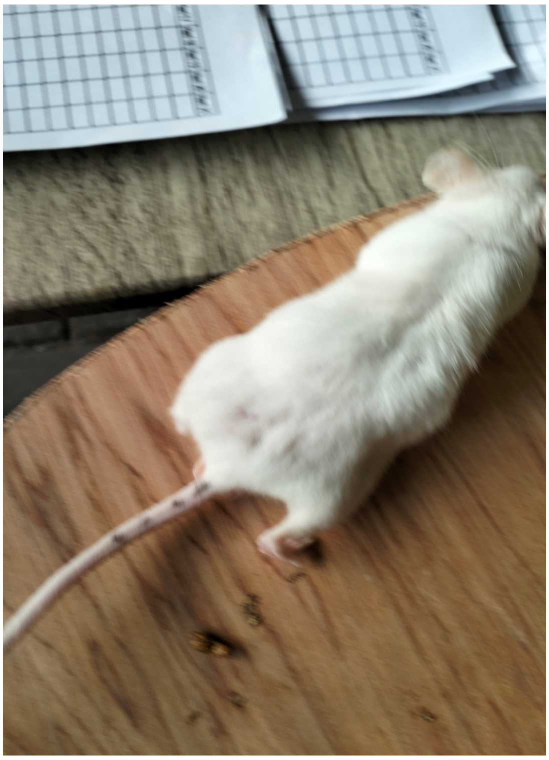

3. Active

5. Responded when the tail and ear were pinched

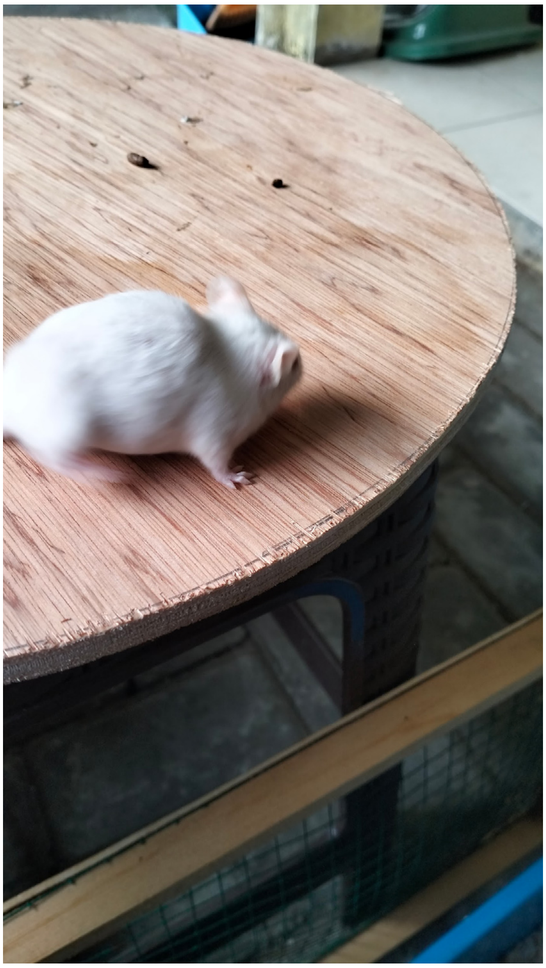

4. Good grip strength

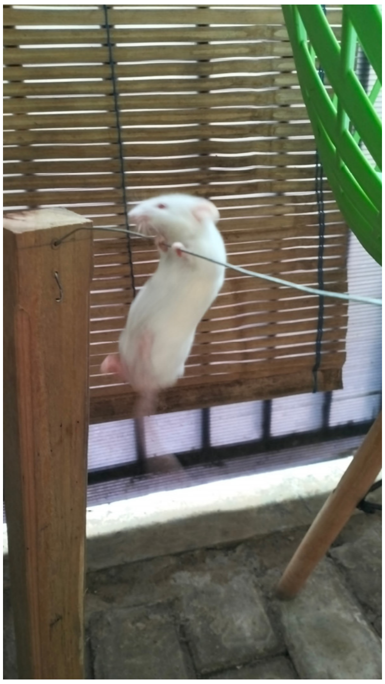

5. Responded when the tail and ear were pinched
6. Normal blood flow, indicated by normal body coloration

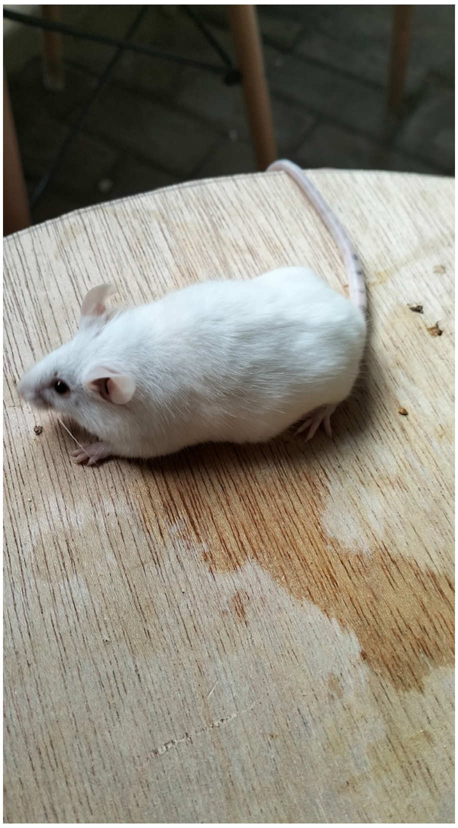

7. Catalepsy time (remaining immobile for less than 1 second)

**OBSERVATIONS IN THE NORMAL CONTROL GROUP**

1. Tail erect
2. Active
3. Aggressive
4. Good grip strength
5. Normal skin color (normal blood flow)
6. Responded when the tail and ear were pinched

## Surgical procedure in mice for blood and organ collection

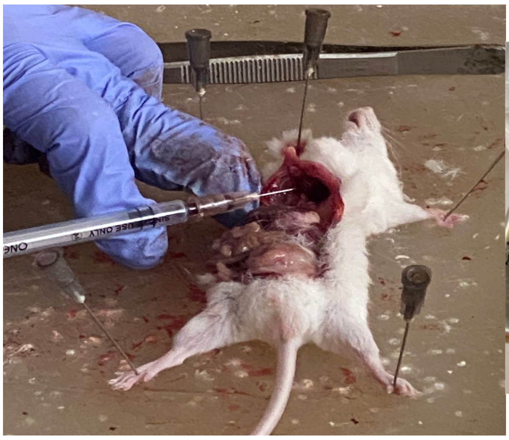

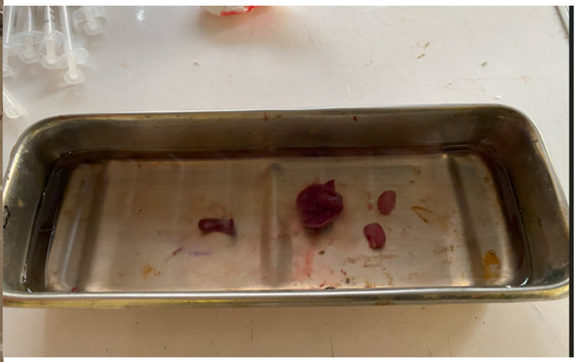

Supplement: Supplementary file 1 [file molecules-31-01428-s001.zip › Survival_Morphine_Evaluation_Surgical.pdf]
