# Supplementary figures and images for: Kratom (Mitragyna speciosa) as a Phytochemical-Based Natural Product Exhibiting Opioid-like Analgesic Effects with Reduced Tolerance and Dependence Liability via TLR4-Associated Neuroimmune Modulation (part 2 of 2)
Source: Molecules. 2026 Apr 26;31(9):1428. doi: 10.3390/molecules31091428 (PMC13164666; doi:10.3390/molecules31091428)

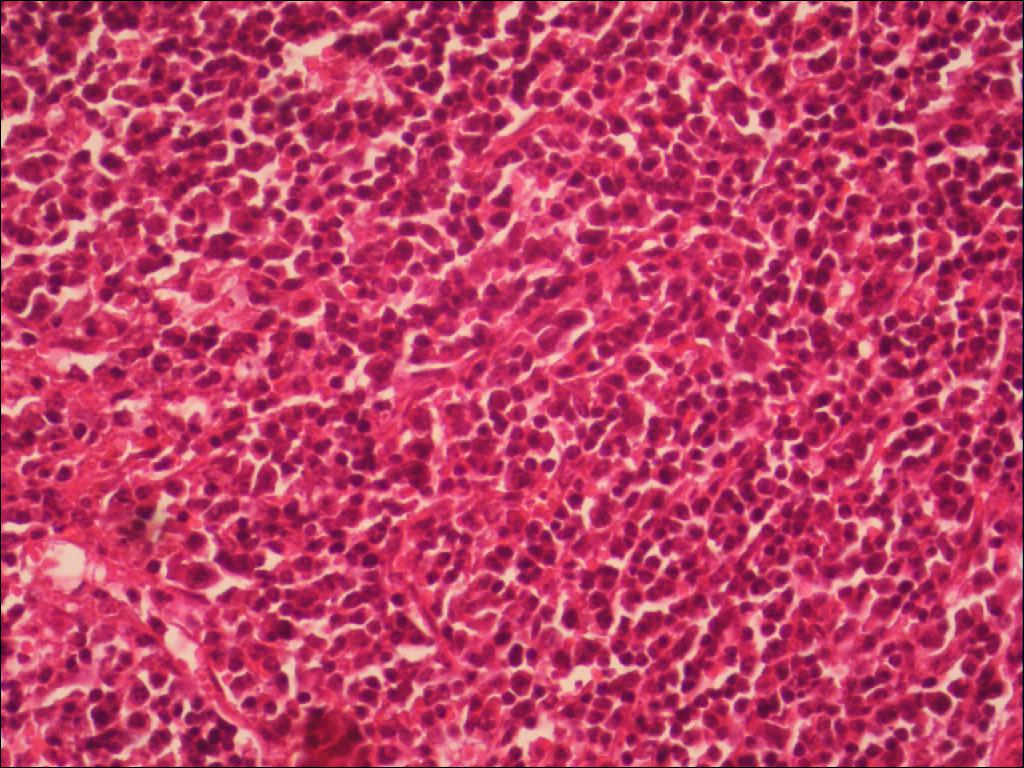

Supplement: Supplementary file 1 [file molecules-31-01428-s001.zip › Splenic Histopathology/Spleen 3.1 (40x).jpg]

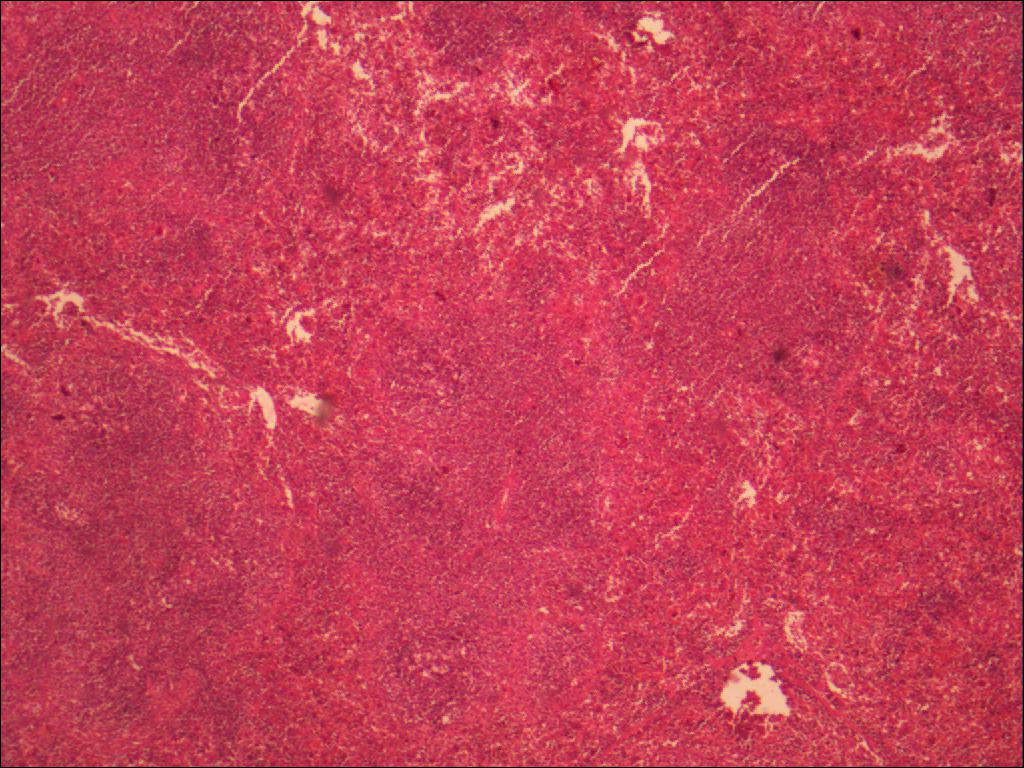

Supplement: Supplementary file 1 [file molecules-31-01428-s001.zip › Splenic Histopathology/Spleen 3.1 (4x).jpg]

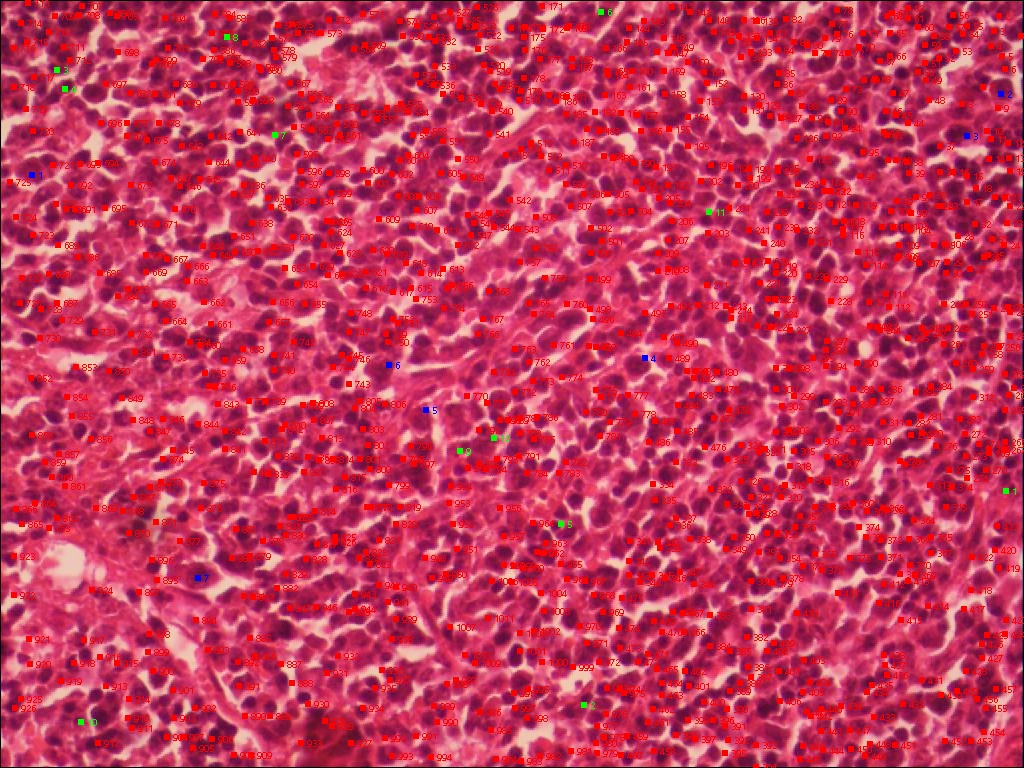

Supplement: Supplementary file 1 [file molecules-31-01428-s001.zip › Splenic Histopathology/Spleen 3.1 Count.jpg]

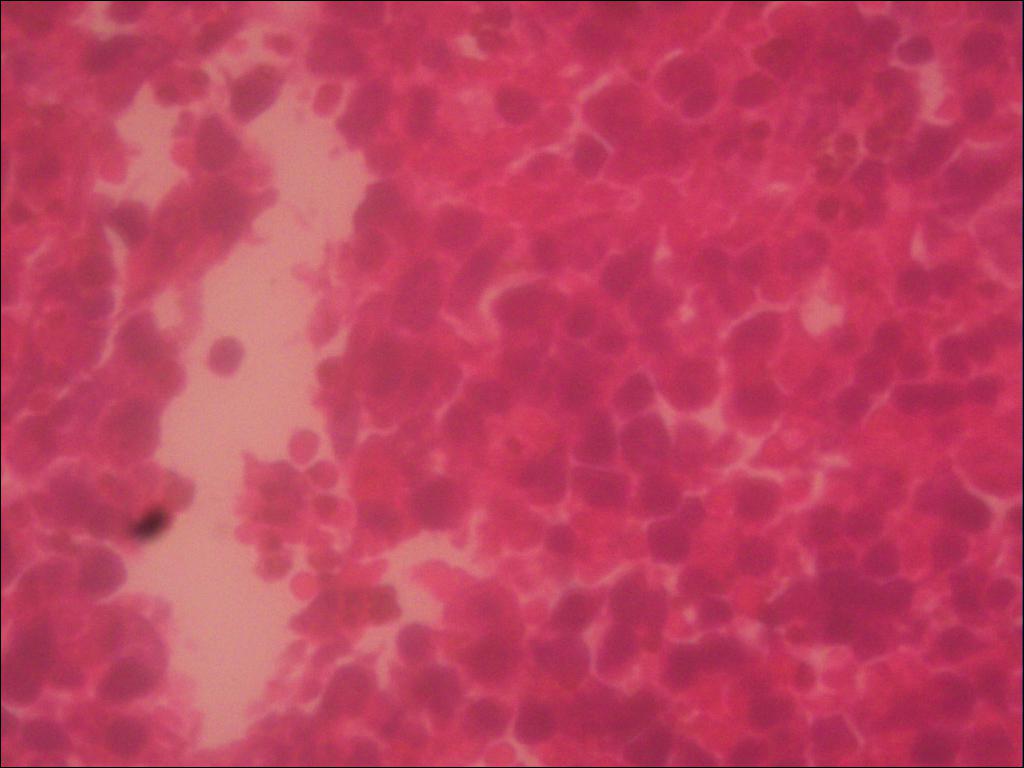

Supplement: Supplementary file 1 [file molecules-31-01428-s001.zip › Splenic Histopathology/Spleen 4.1 (100x).jpg]

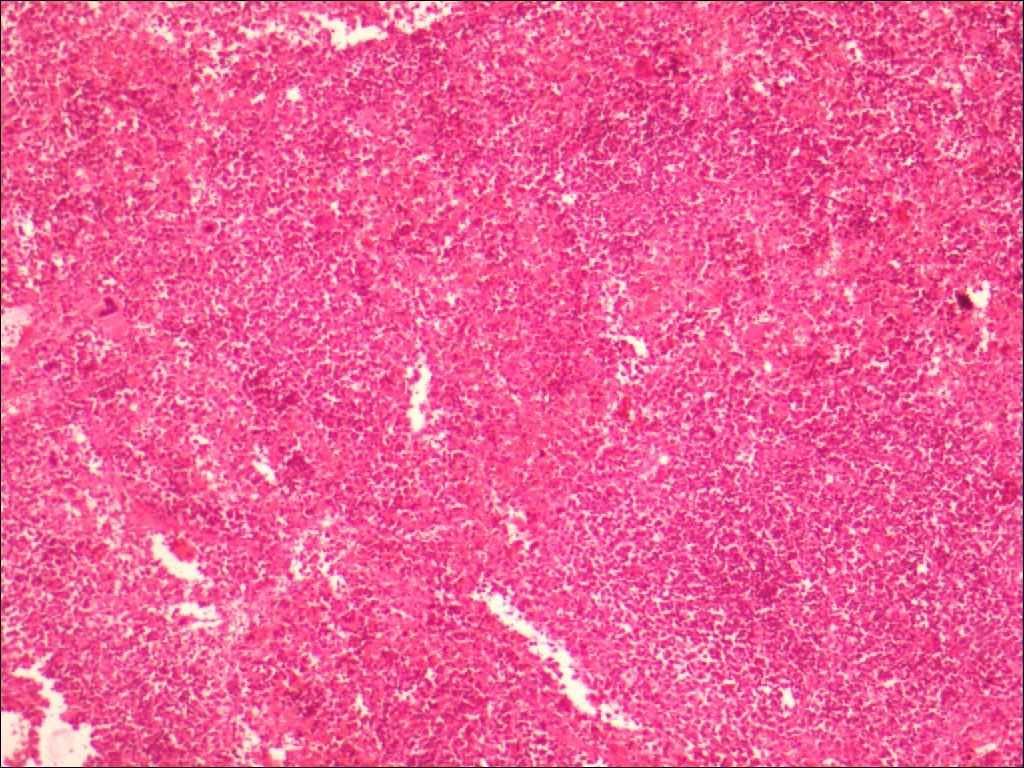

Supplement: Supplementary file 1 [file molecules-31-01428-s001.zip › Splenic Histopathology/Spleen 4.1 (10x).jpg]

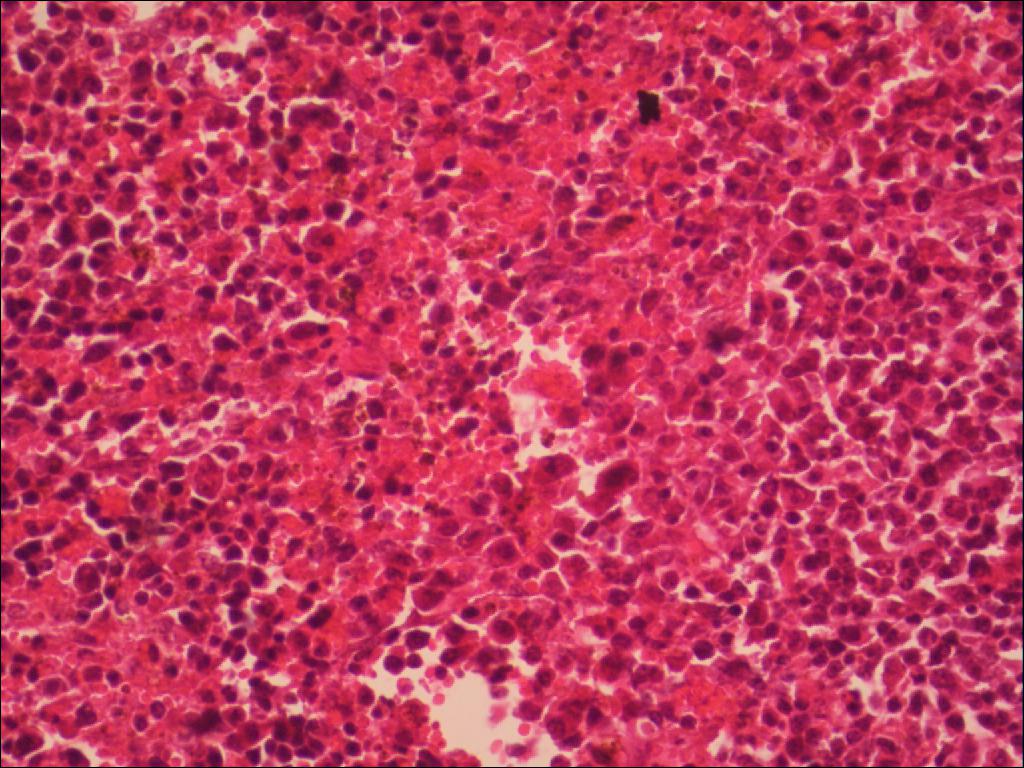

Supplement: Supplementary file 1 [file molecules-31-01428-s001.zip › Splenic Histopathology/Spleen 4.1 (40x).jpg]

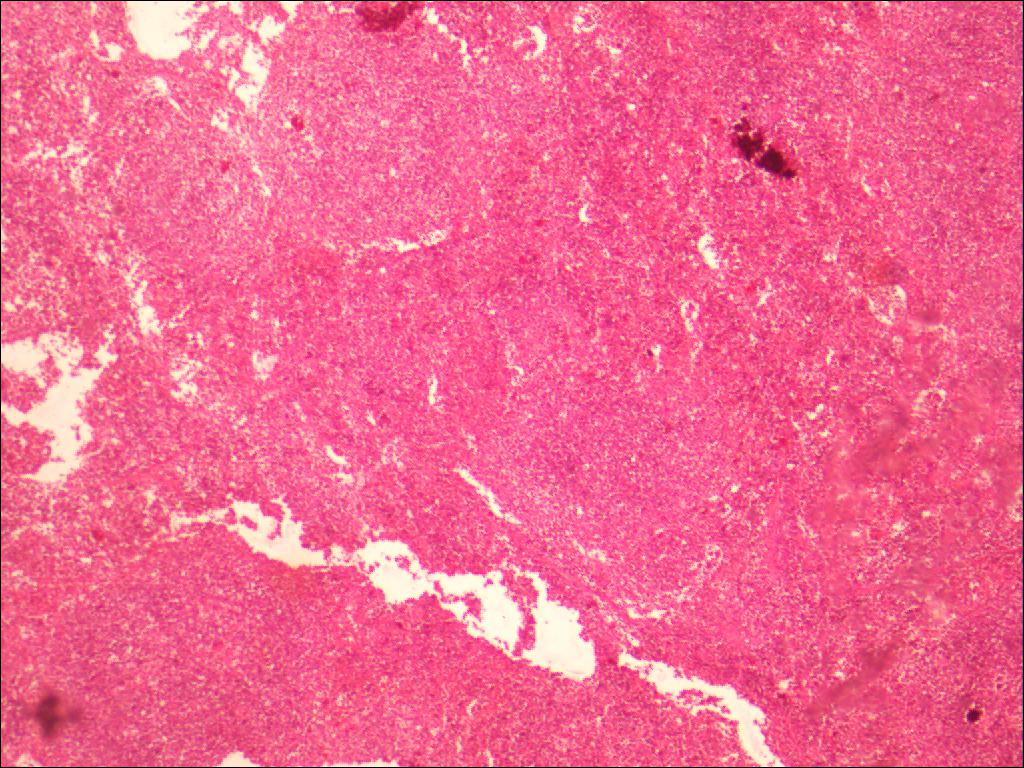

Supplement: Supplementary file 1 [file molecules-31-01428-s001.zip › Splenic Histopathology/Spleen 4.1 (4x).jpg]

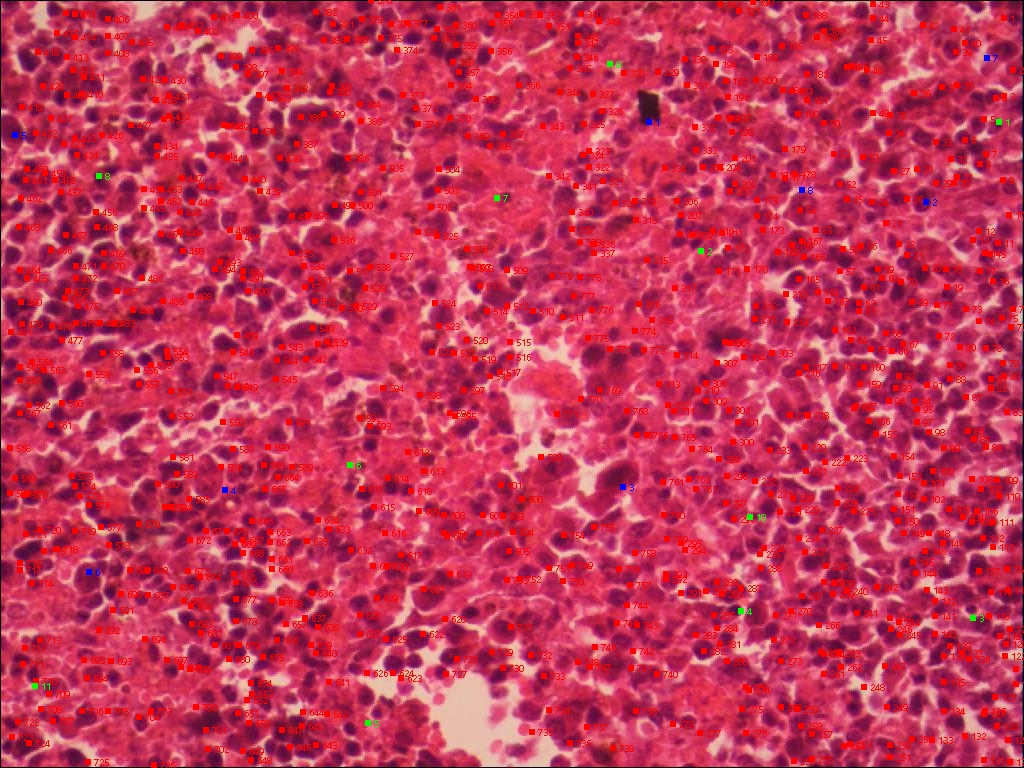

Supplement: Supplementary file 1 [file molecules-31-01428-s001.zip › Splenic Histopathology/Spleen 4.1 Count.jpg]

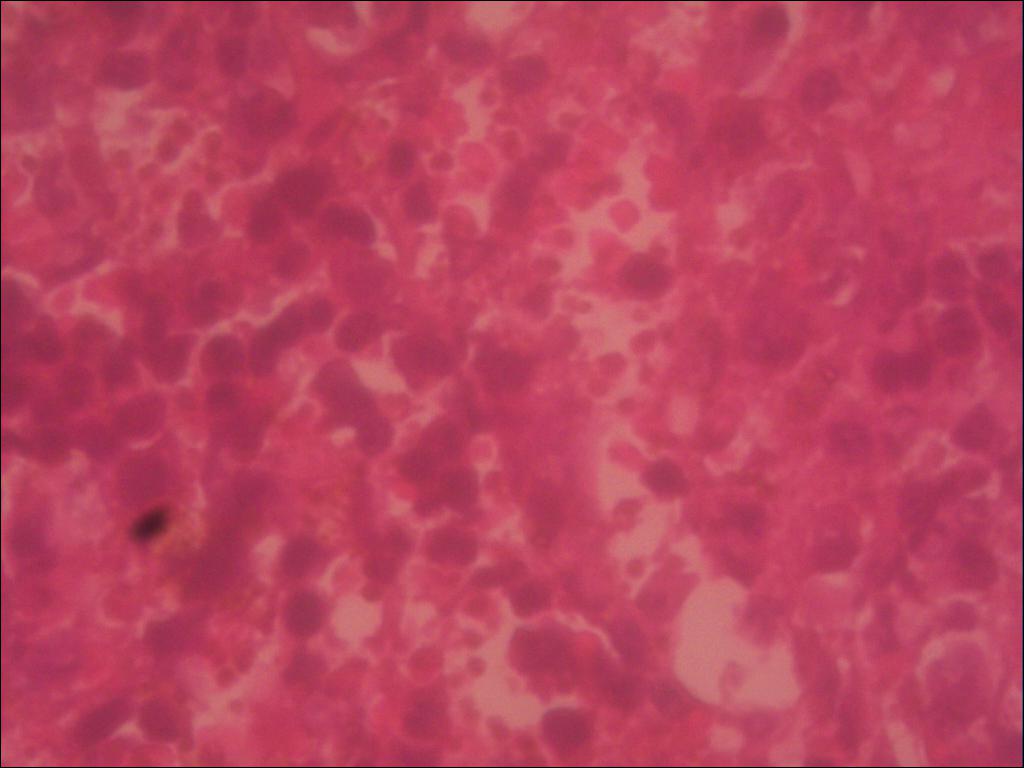

Supplement: Supplementary file 1 [file molecules-31-01428-s001.zip › Splenic Histopathology/Spleen 5.1 (100x).jpg]

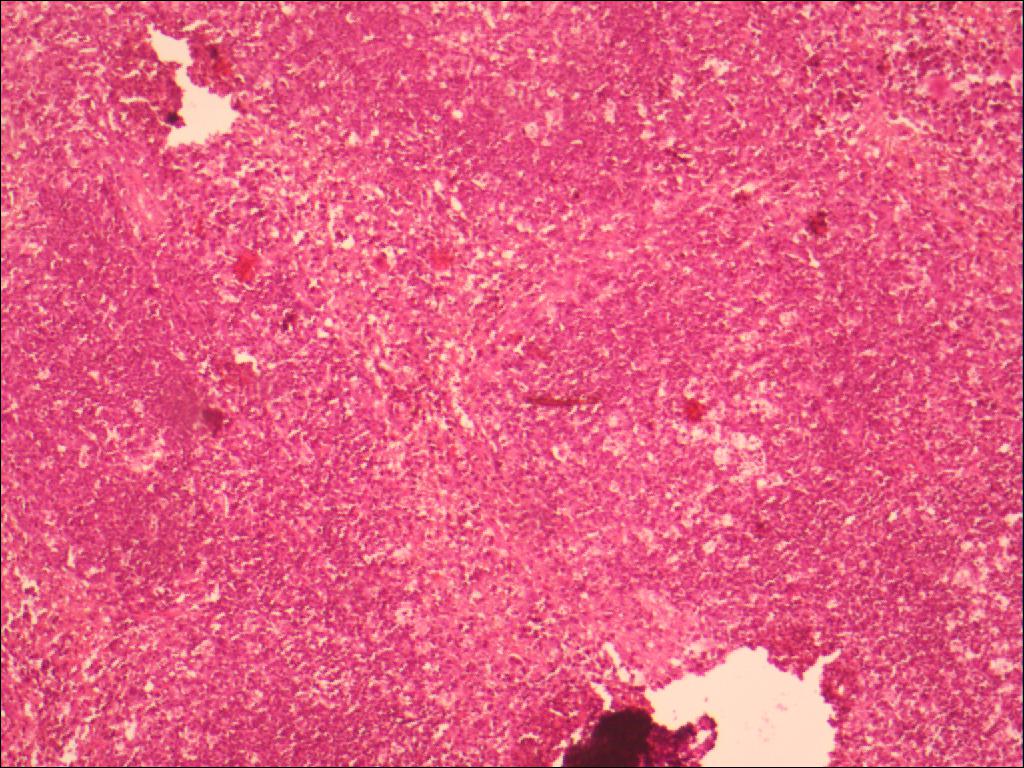

Supplement: Supplementary file 1 [file molecules-31-01428-s001.zip › Splenic Histopathology/Spleen 5.1 (10x).jpg]

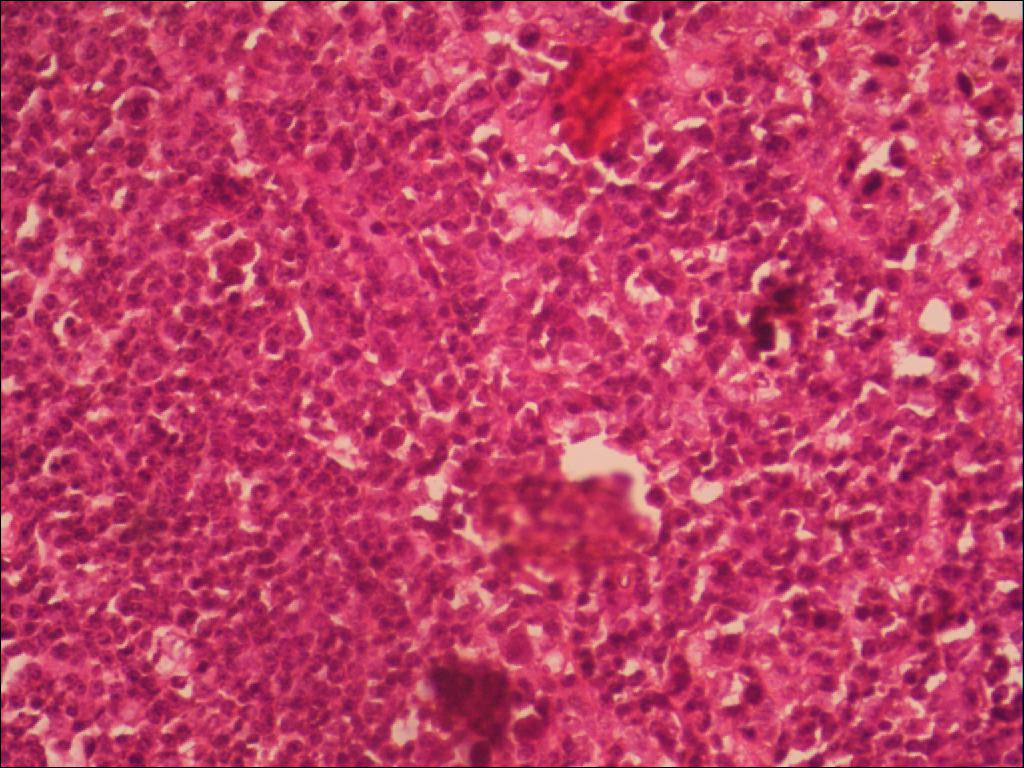

Supplement: Supplementary file 1 [file molecules-31-01428-s001.zip › Splenic Histopathology/Spleen 5.1 (40x).jpg]

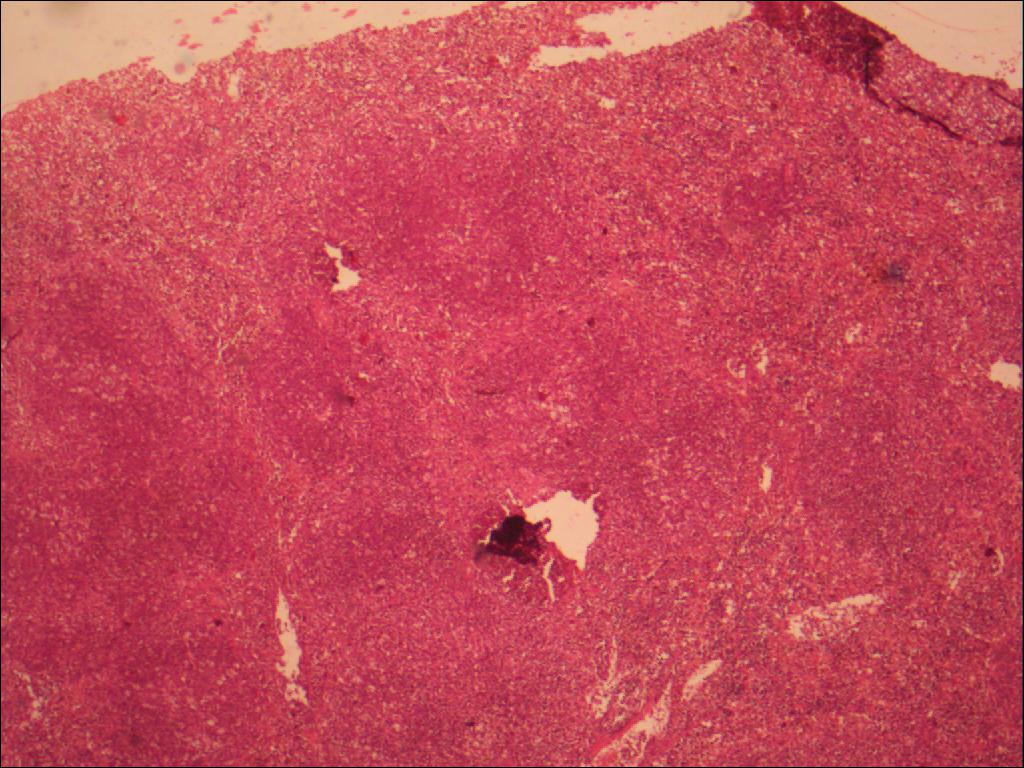

Supplement: Supplementary file 1 [file molecules-31-01428-s001.zip › Splenic Histopathology/Spleen 5.1 (4x).jpg]

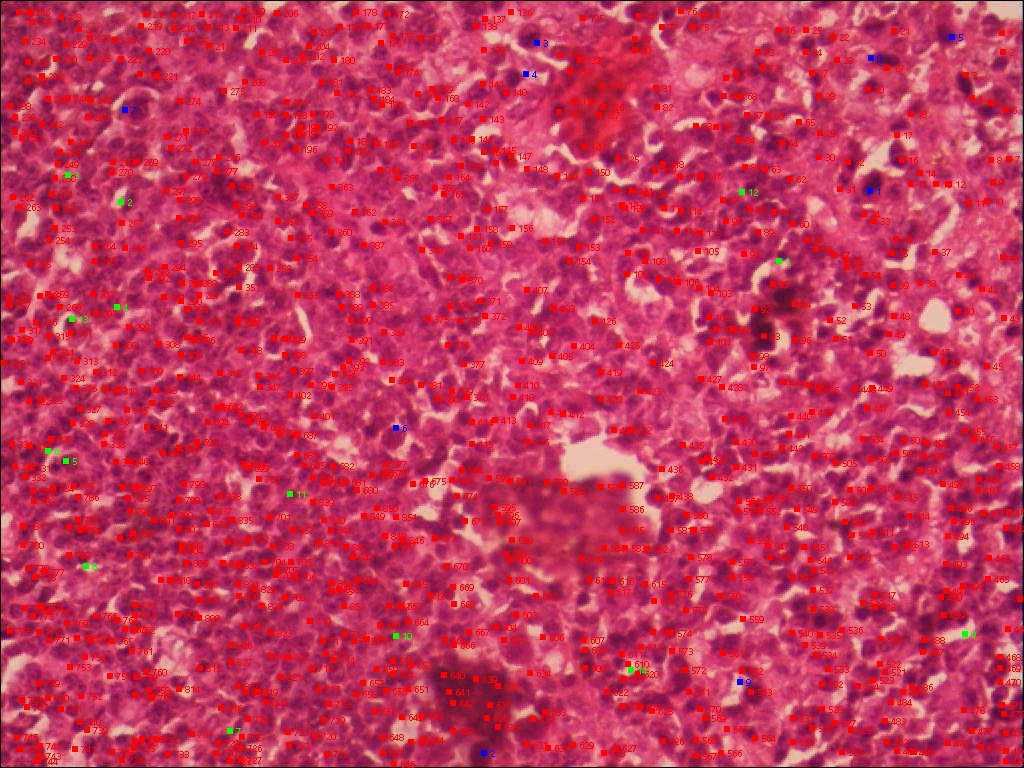

Supplement: Supplementary file 1 [file molecules-31-01428-s001.zip › Splenic Histopathology/Spleen 5.1 Count.jpg]

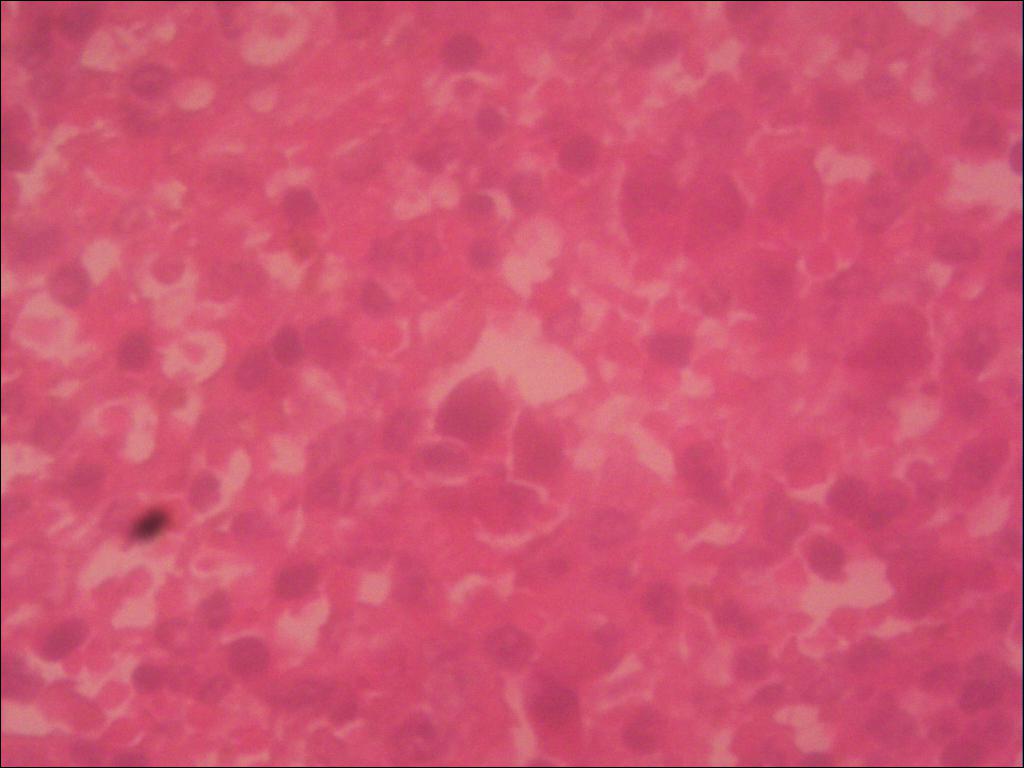

Supplement: Supplementary file 1 [file molecules-31-01428-s001.zip › Splenic Histopathology/Spleen 6.2 (100x).jpg]

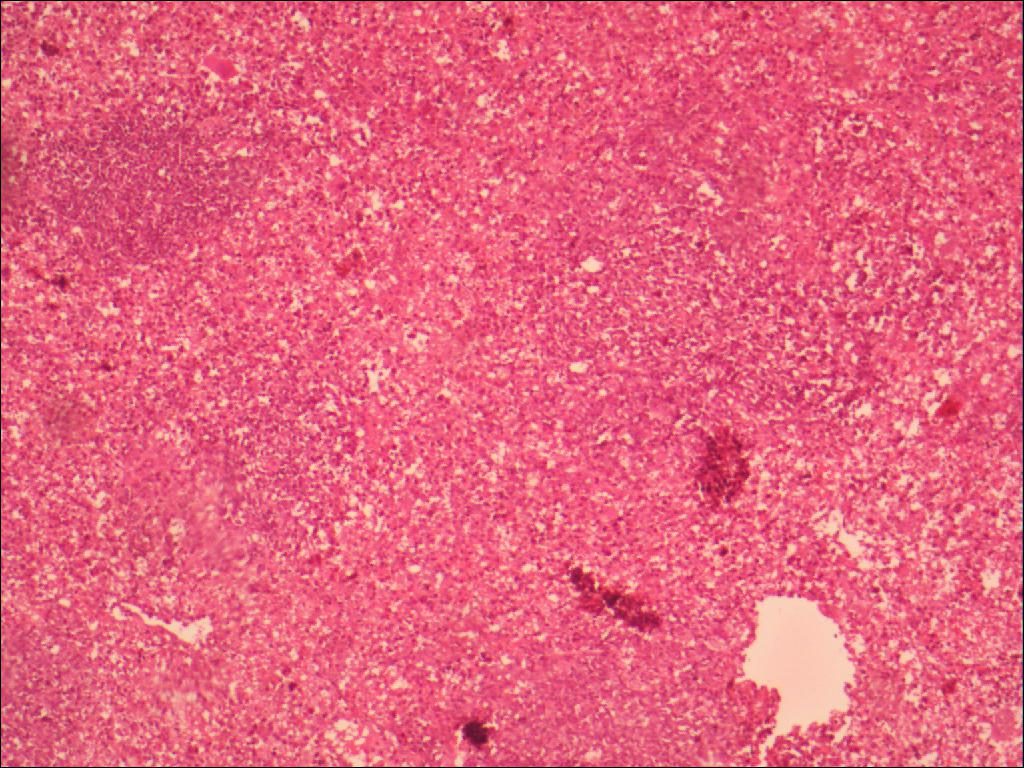

Supplement: Supplementary file 1 [file molecules-31-01428-s001.zip › Splenic Histopathology/Spleen 6.2 (10x).jpg]

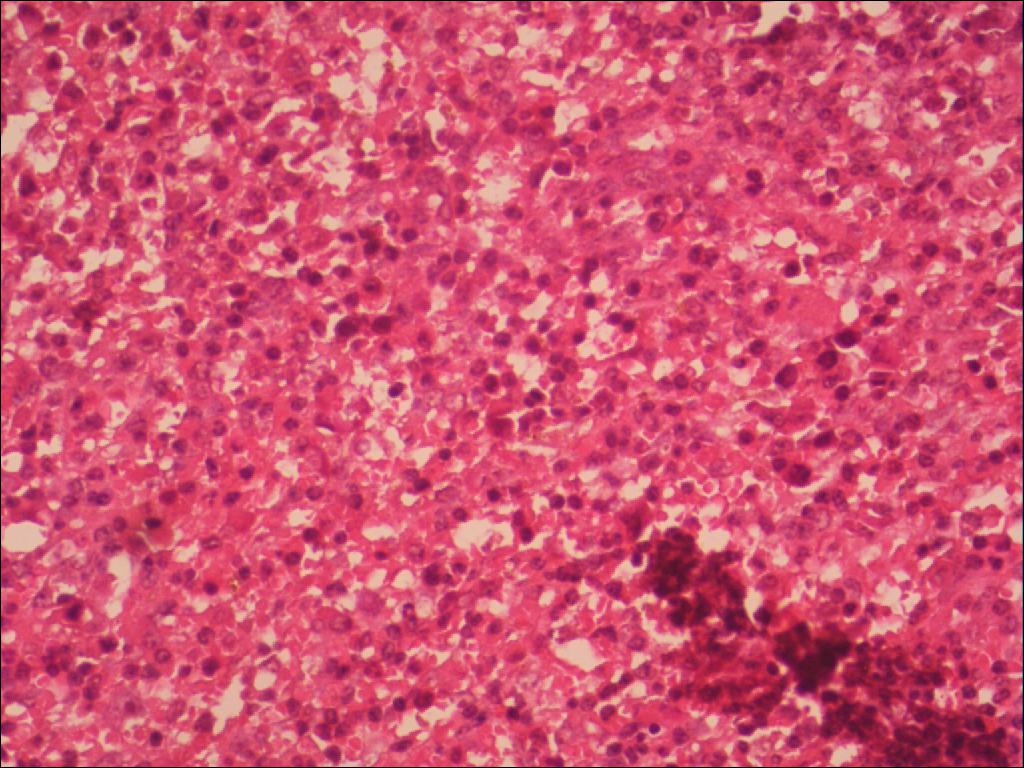

Supplement: Supplementary file 1 [file molecules-31-01428-s001.zip › Splenic Histopathology/Spleen 6.2 (40x).jpg]

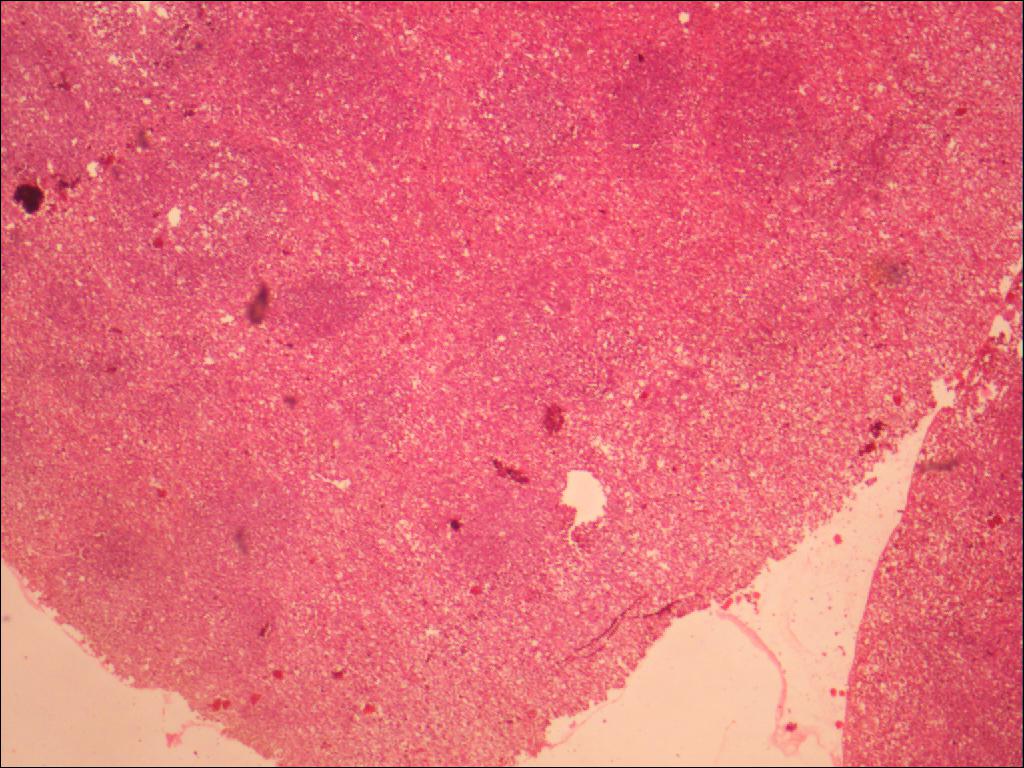

Supplement: Supplementary file 1 [file molecules-31-01428-s001.zip › Splenic Histopathology/Spleen 6.2 (4x).jpg]

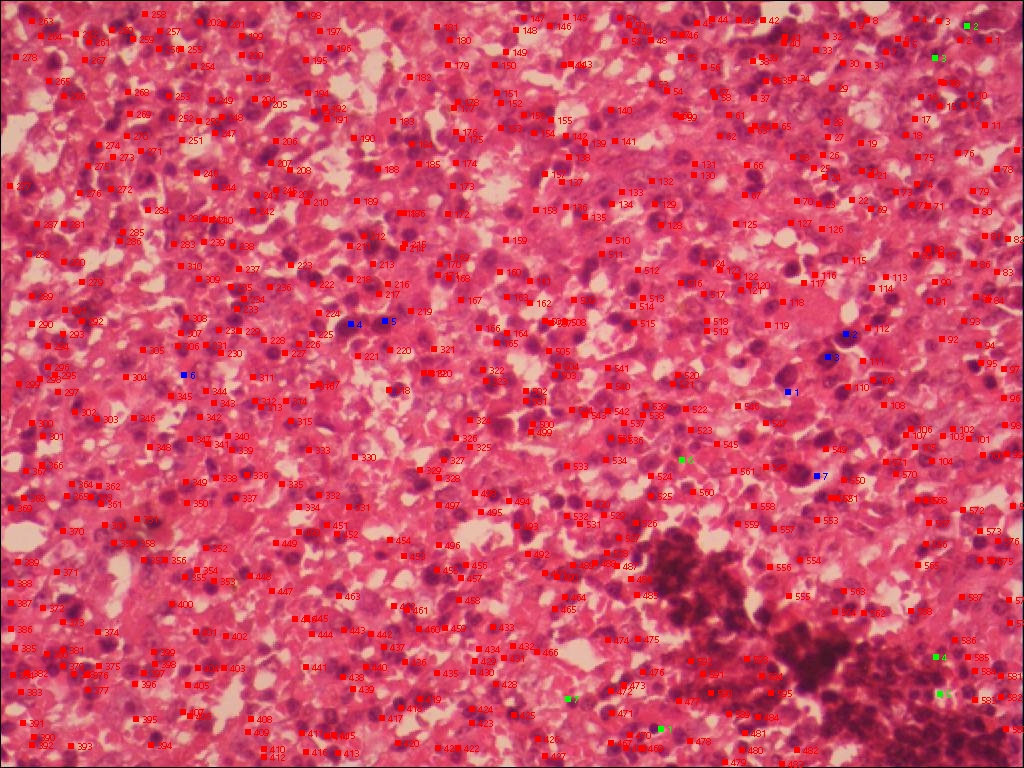

Supplement: Supplementary file 1 [file molecules-31-01428-s001.zip › Splenic Histopathology/Spleen 6.2 Count.jpg]

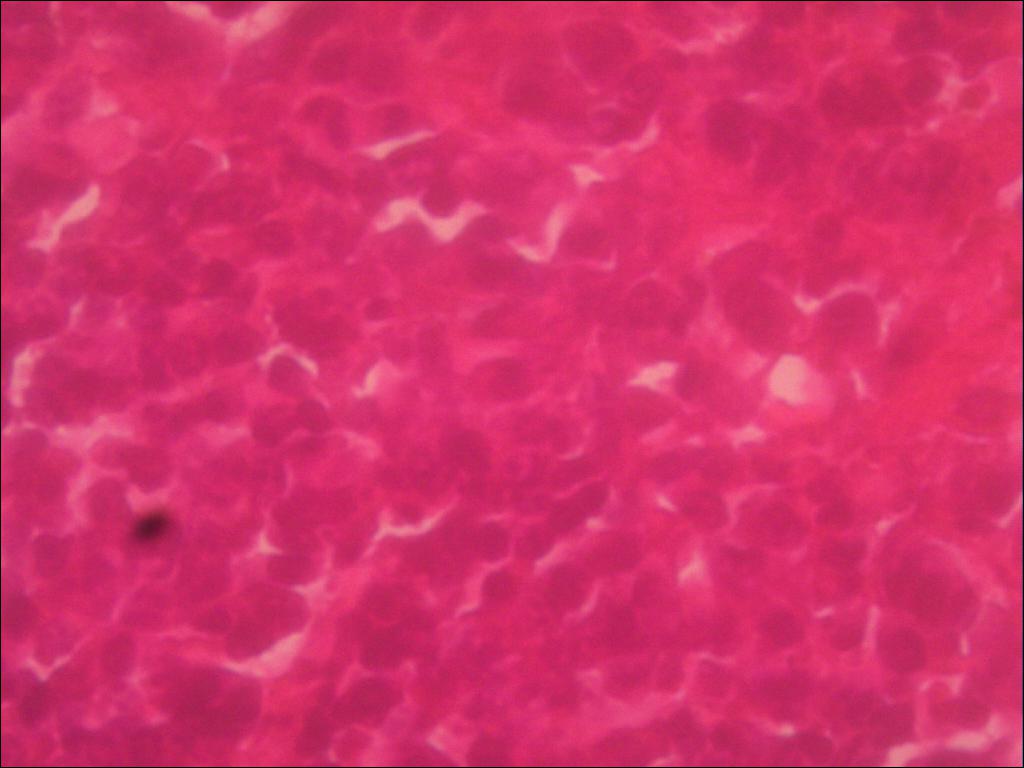

Supplement: Supplementary file 1 [file molecules-31-01428-s001.zip › Splenic Histopathology/Spleen 7.1 (100x).jpg]

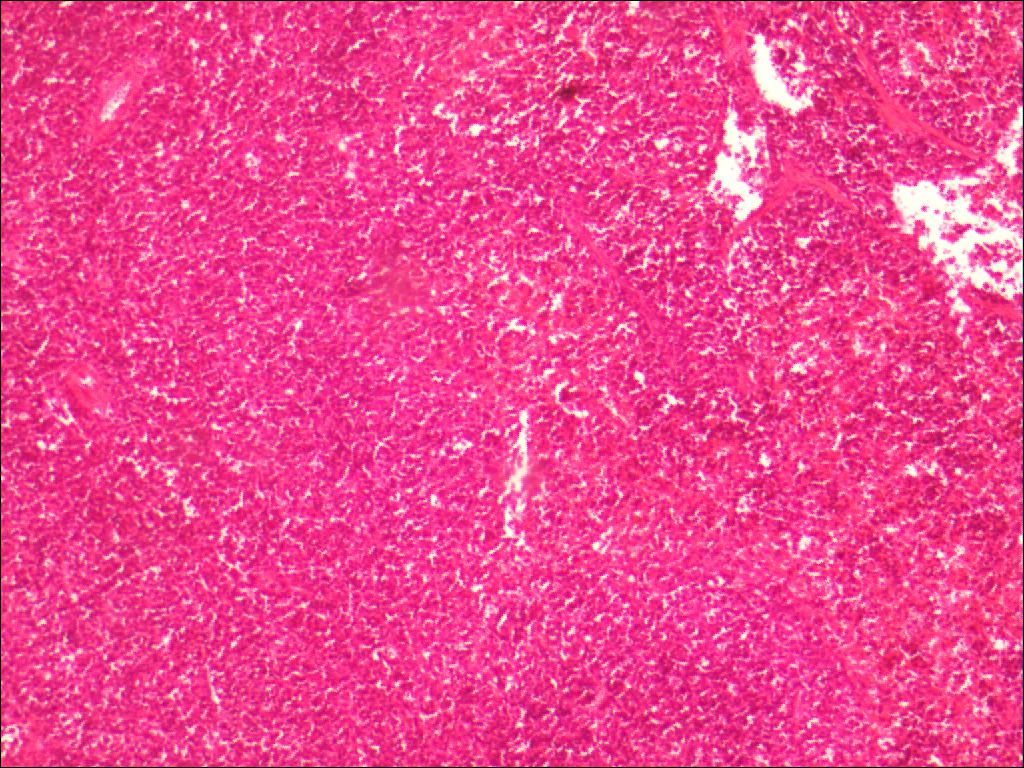

Supplement: Supplementary file 1 [file molecules-31-01428-s001.zip › Splenic Histopathology/Spleen 7.1 (10x).jpg]

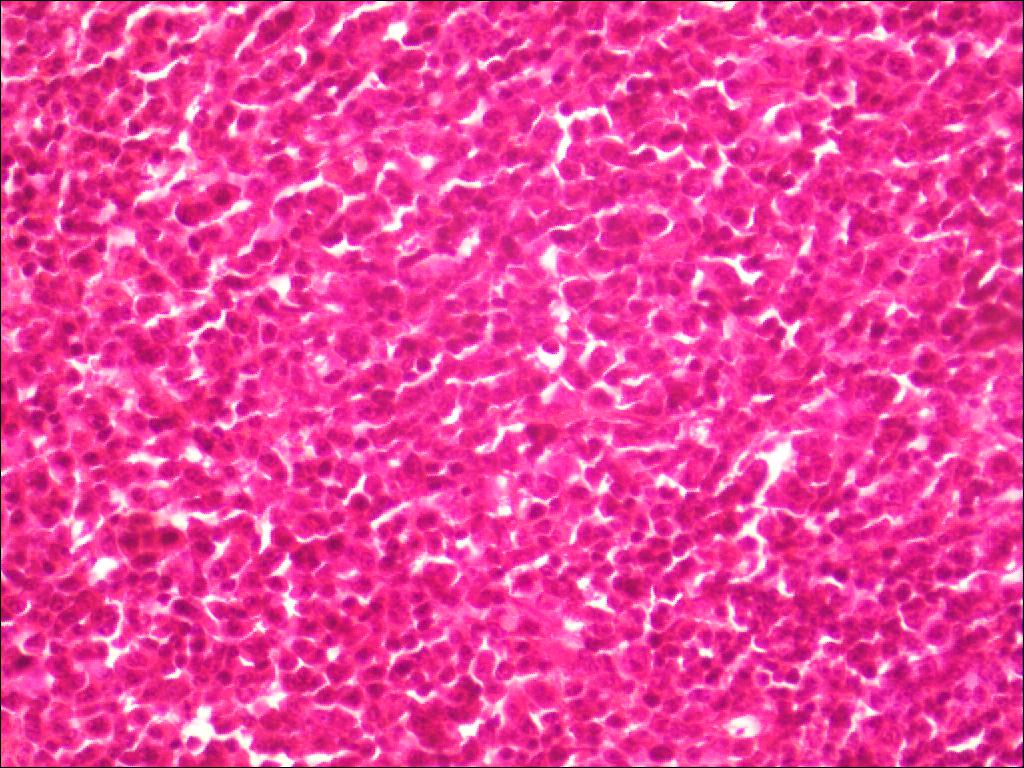

Supplement: Supplementary file 1 [file molecules-31-01428-s001.zip › Splenic Histopathology/Spleen 7.1 (40x).jpg]

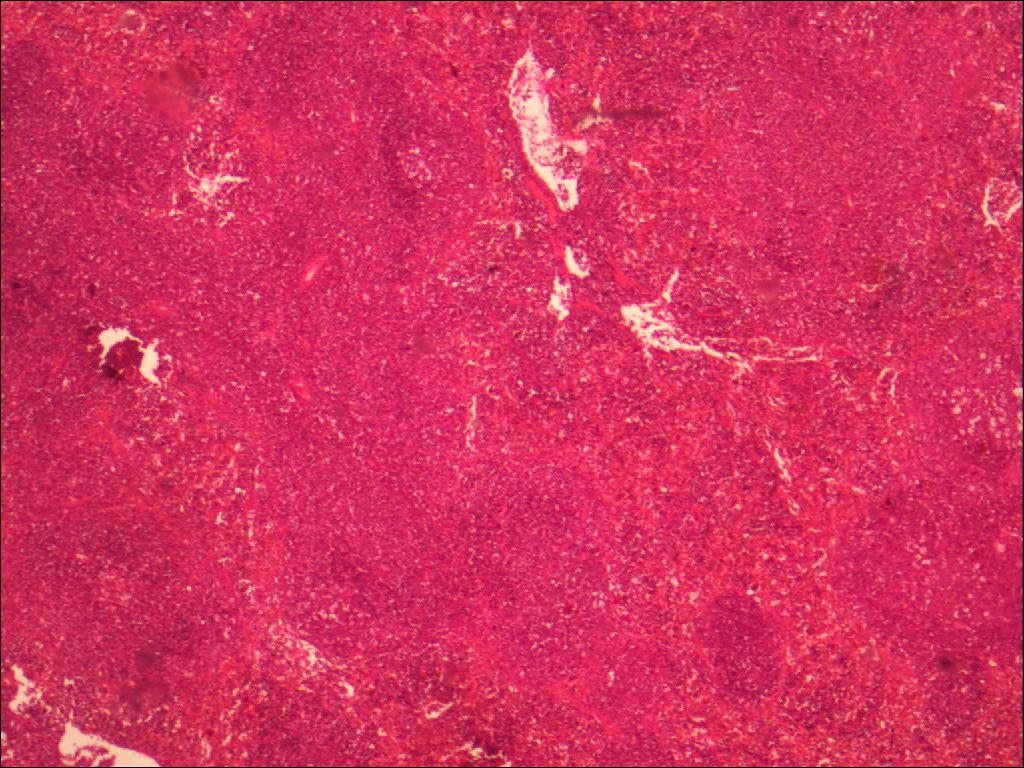

Supplement: Supplementary file 1 [file molecules-31-01428-s001.zip › Splenic Histopathology/Spleen 7.1 (4x).jpg]

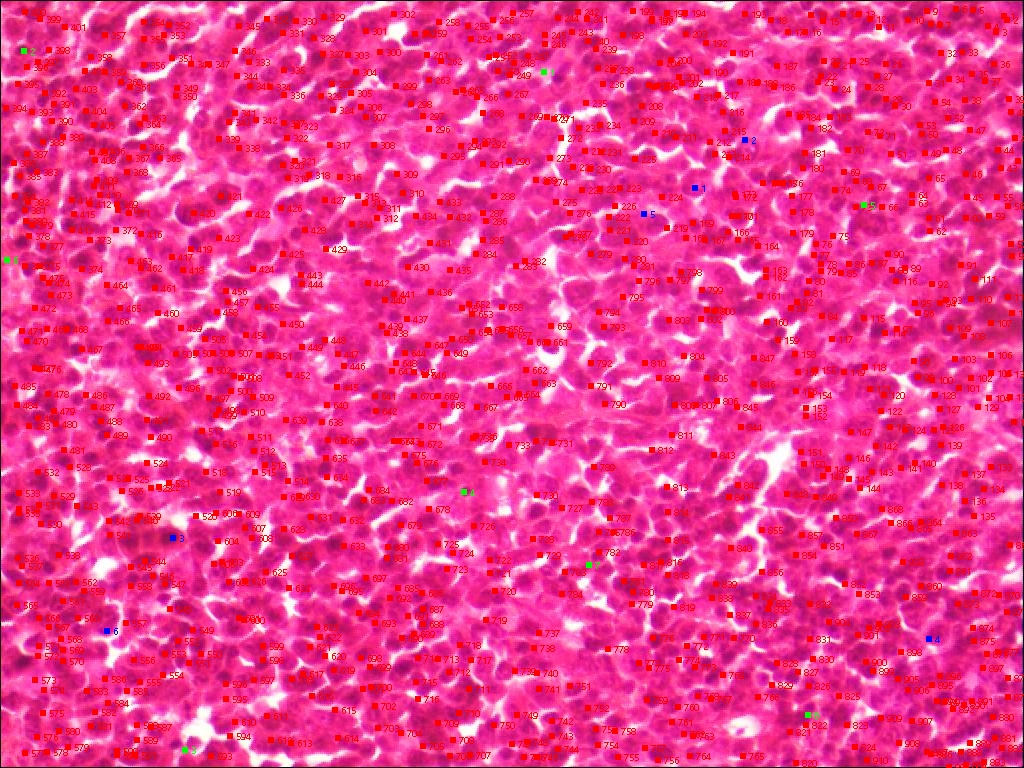

Supplement: Supplementary file 1 [file molecules-31-01428-s001.zip › Splenic Histopathology/Spleen 7.1 Count.jpg]

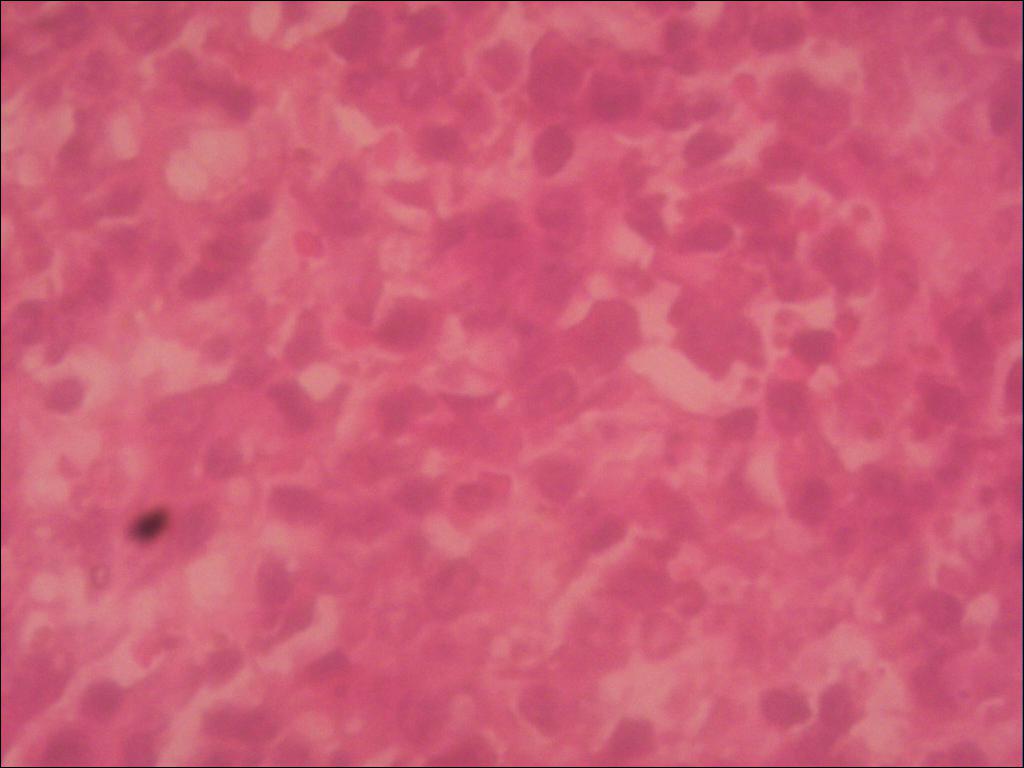

Supplement: Supplementary file 1 [file molecules-31-01428-s001.zip › Splenic Histopathology/Spleen N1 (100x).jpg]

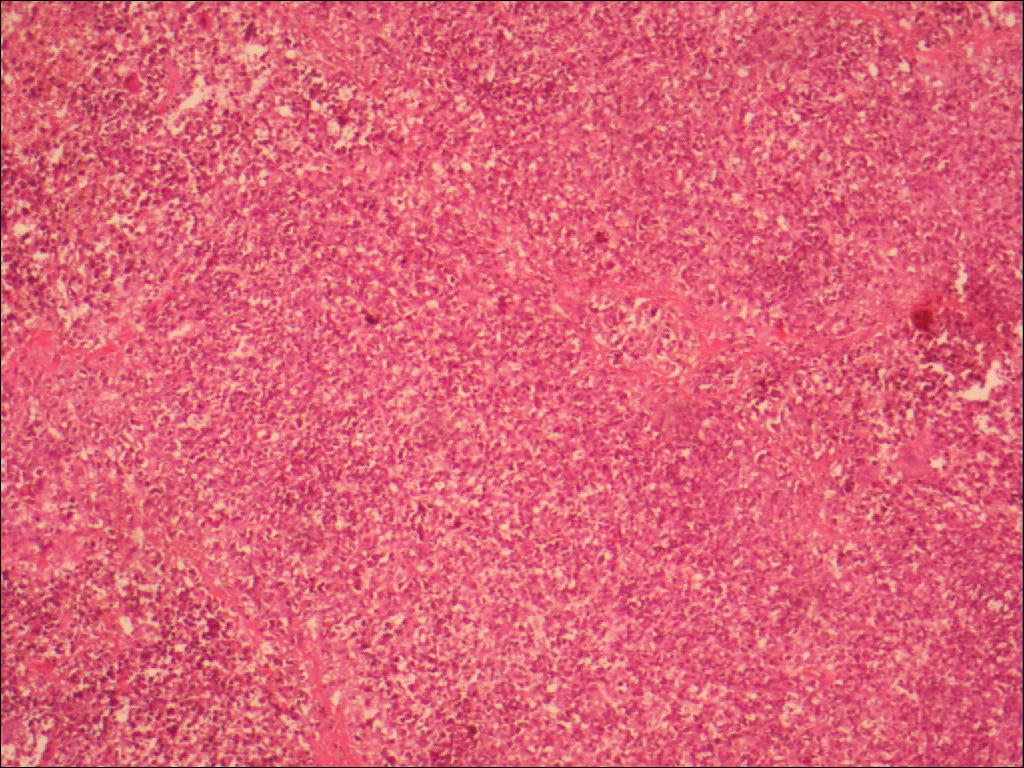

Supplement: Supplementary file 1 [file molecules-31-01428-s001.zip › Splenic Histopathology/Spleen N1 (10x).jpg]

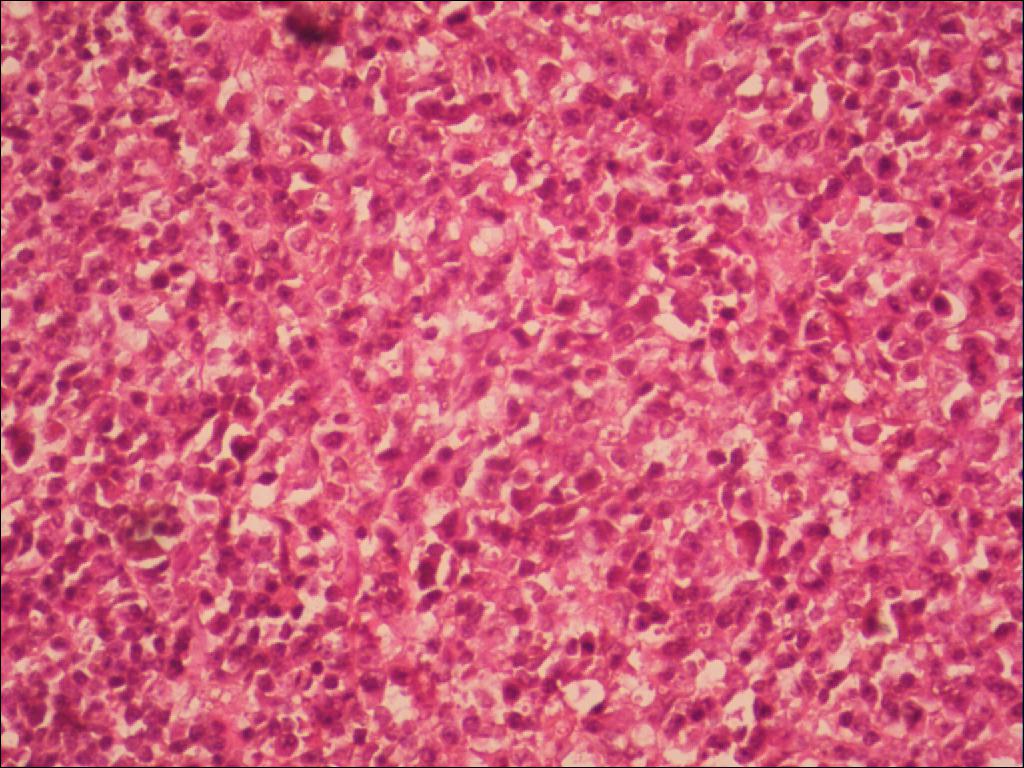

Supplement: Supplementary file 1 [file molecules-31-01428-s001.zip › Splenic Histopathology/Spleen N1 (40x).jpg]

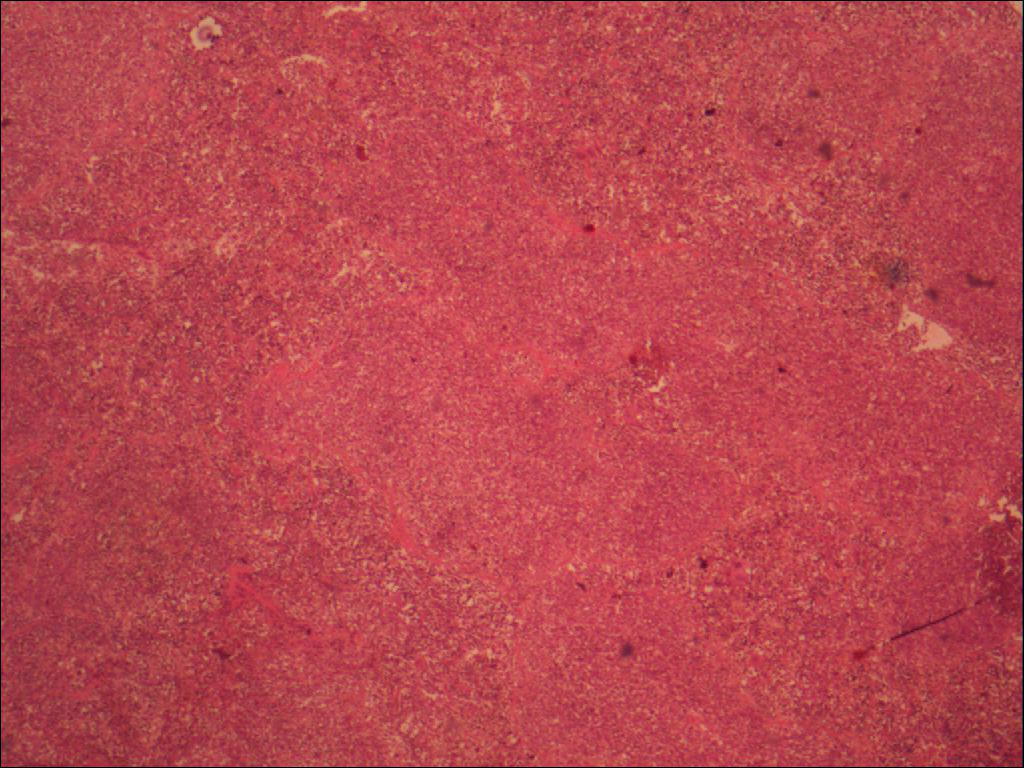

Supplement: Supplementary file 1 [file molecules-31-01428-s001.zip › Splenic Histopathology/Spleen N1 (4x).jpg]

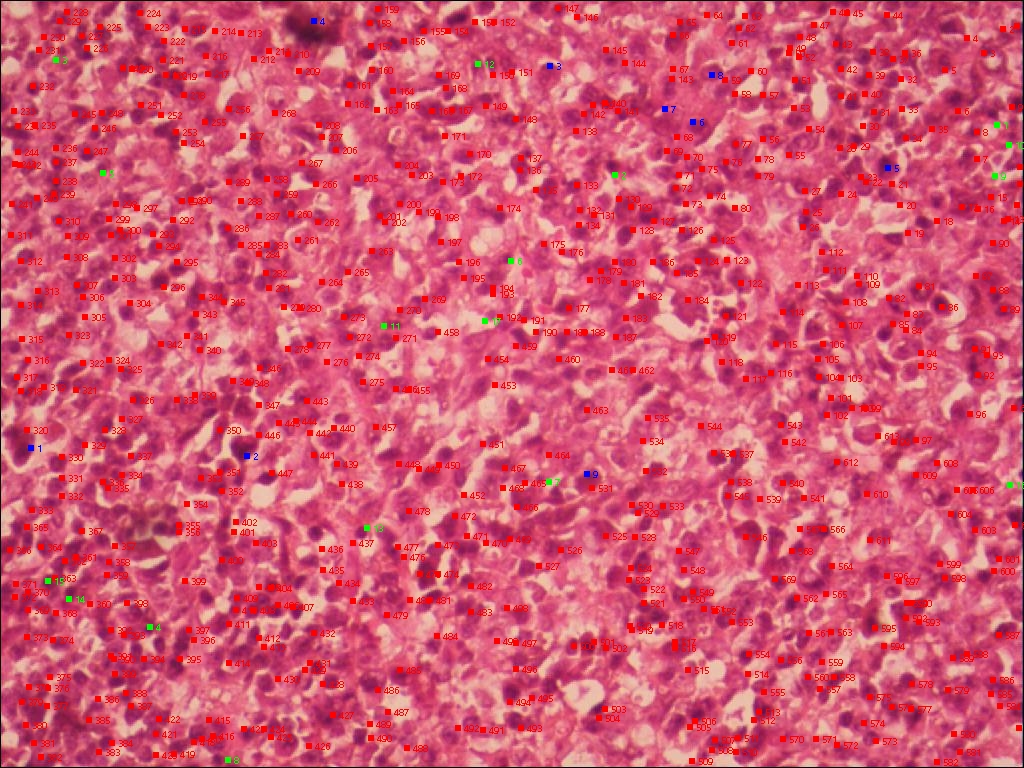

Supplement: Supplementary file 1 [file molecules-31-01428-s001.zip › Splenic Histopathology/Spleen N1 Count.jpg]

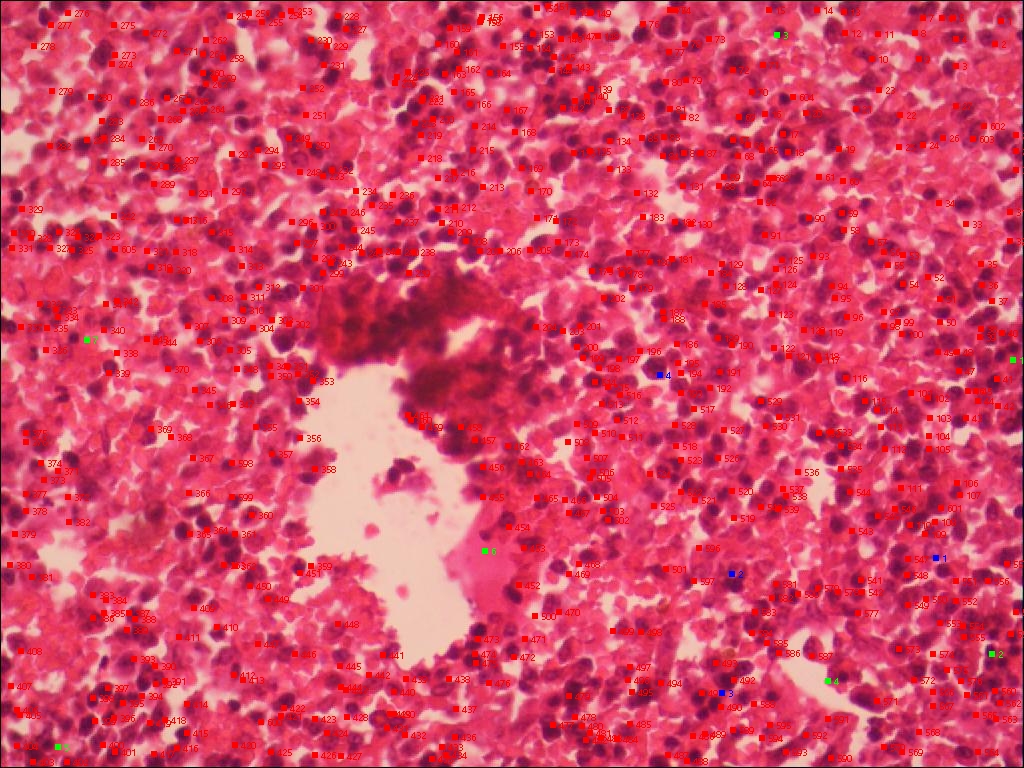

Supplement: Supplementary file 1 [file molecules-31-01428-s001.zip › Splenic Histopathology/Spleen Norma_ Count.jpg]

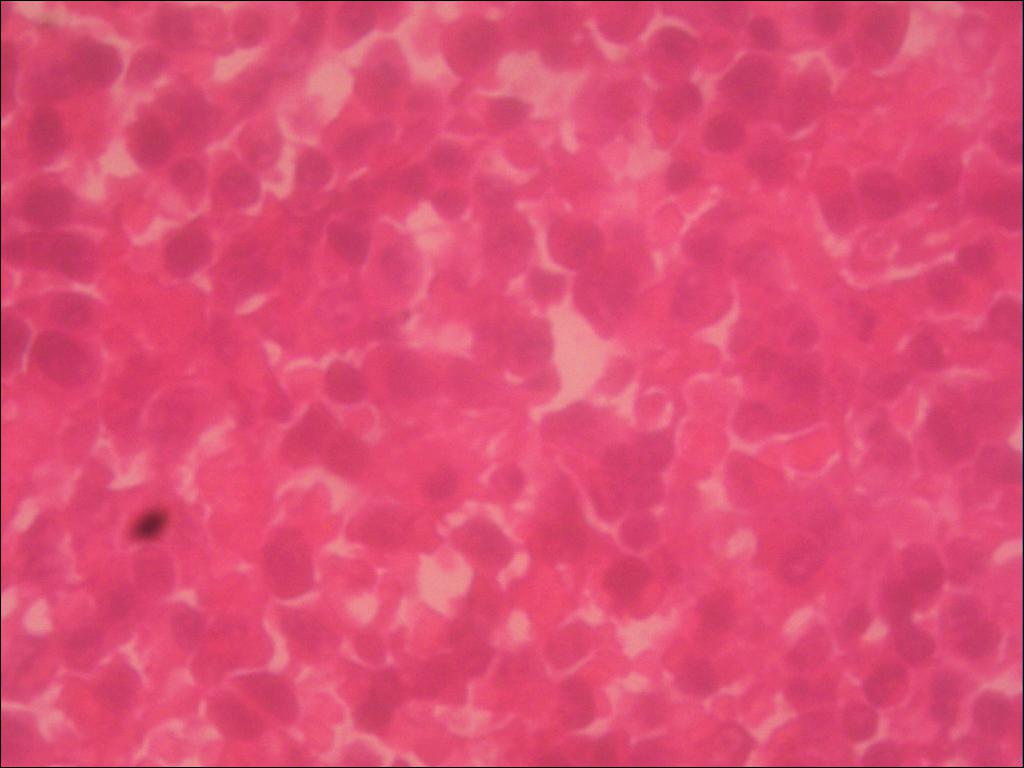

Supplement: Supplementary file 1 [file molecules-31-01428-s001.zip › Splenic Histopathology/Spleen NORMAL 1 (100x).jpg]

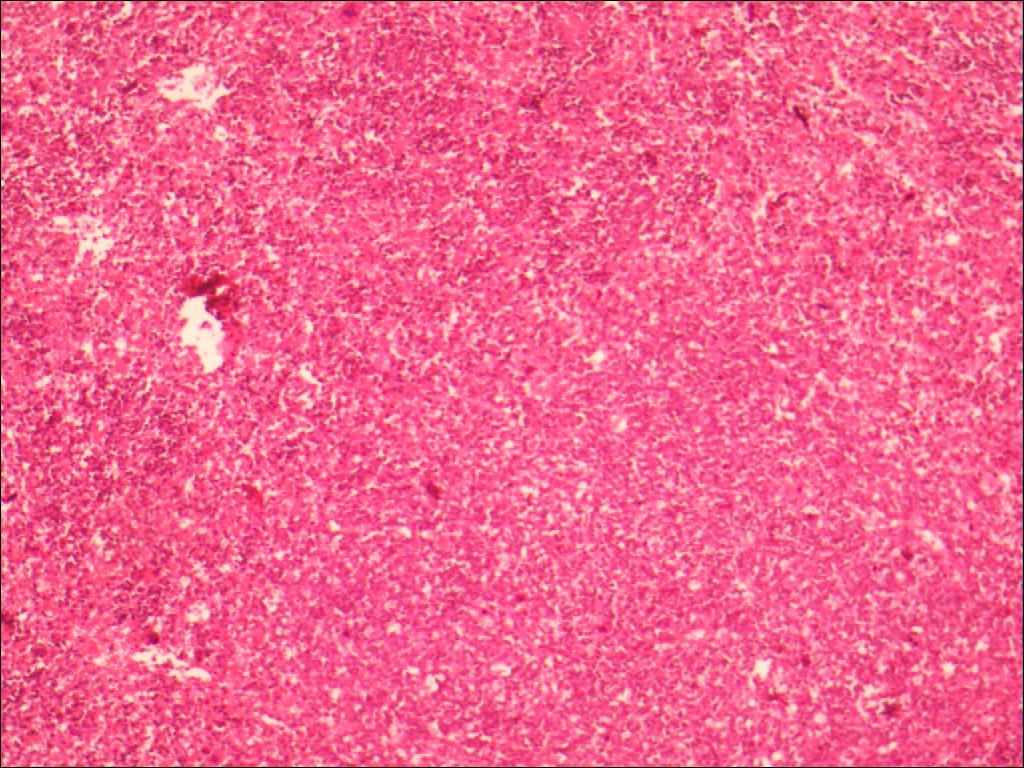

Supplement: Supplementary file 1 [file molecules-31-01428-s001.zip › Splenic Histopathology/Spleen NORMAL 1 (10x).jpg]

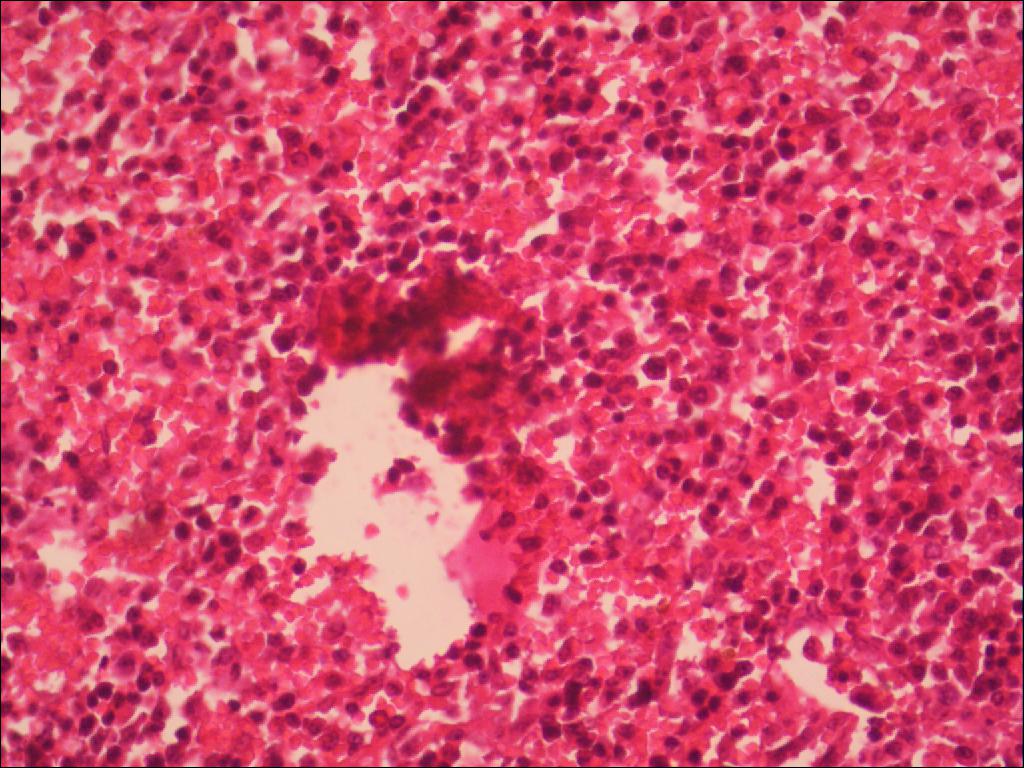

Supplement: Supplementary file 1 [file molecules-31-01428-s001.zip › Splenic Histopathology/Spleen NORMAL 1 (40x).jpg]

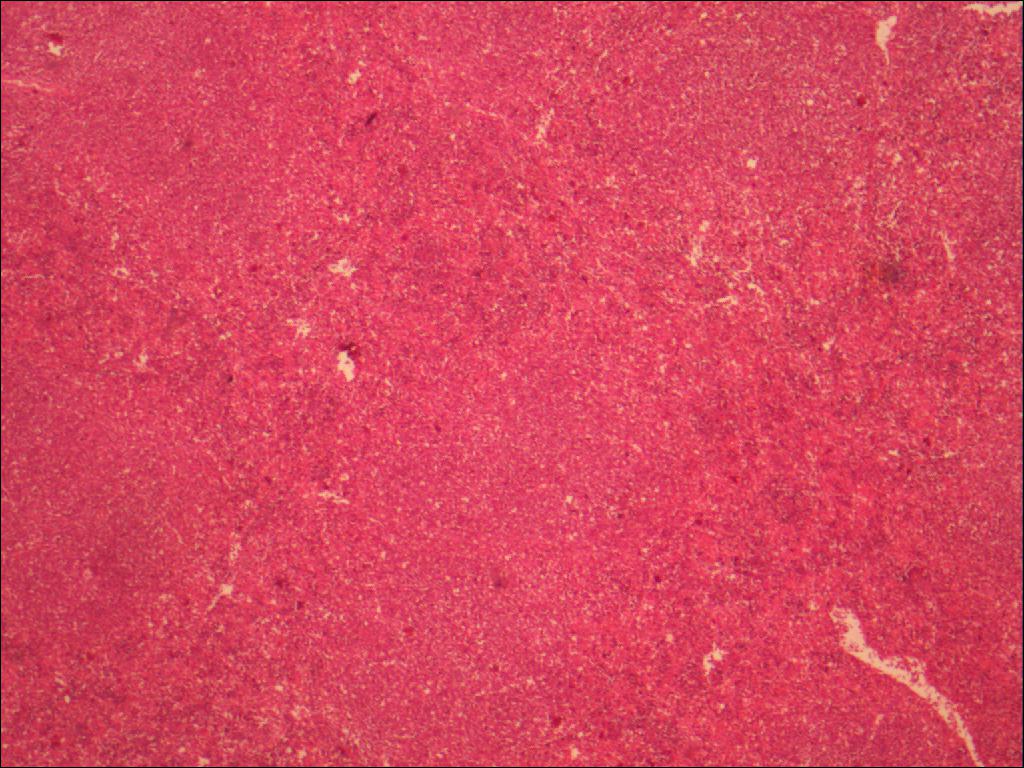

Supplement: Supplementary file 1 [file molecules-31-01428-s001.zip › Splenic Histopathology/Spleen NORMAL 1 (4x).jpg]
